# Metal-free glycosylation with glycosyl fluorides in liquid SO_2_

**DOI:** 10.3762/bjoc.17.78

**Published:** 2021-04-29

**Authors:** Krista Gulbe, Jevgeņija Lugiņina, Edijs Jansons, Artis Kinens, Māris Turks

**Affiliations:** 1 Institute of Technology of Organic Chemistry, Faculty of Materials Science and Applied Chemistry, Riga Technical University, P. Valdena str. 3, Riga, LV-1048, Latvia; 2 Latvian Institute of Organic Synthesis, Aizkraukles str. 21, Riga, LV-1006, Latvia; 3 Department of Chemistry, University of Latvia, Jelgavas str. 1, Riga, LV-1004, Latvia

**Keywords:** fluorosulfite, glycosyl fluoride, Lewis acid, liquid sulfur dioxide, metal-free glycosylation

## Abstract

Liquid SO_2_ is a polar solvent that dissolves both covalent and ionic compounds. Sulfur dioxide possesses also Lewis acid properties, including the ability to covalently bind Lewis basic fluoride ions in a relatively stable fluorosulfite anion (FSO_2_^−^). Herein we report the application of liquid SO_2_ as a promoting solvent for glycosylation with glycosyl fluorides without any external additive. By using various temperature regimes, the method is applied for both armed and disarmed glucose and mannose-derived glycosyl fluorides in moderate to excellent yields. A series of pivaloyl-protected *O*- and *S*-mannosides, as well as one example of a *C*-mannoside, are synthesized to demonstrate the scope of the glycosyl acceptors. The formation of the fluorosulfite species during the glycosylation with glycosyl fluorides in liquid SO_2_ is proved by ^19^F NMR spectroscopy. A sulfur dioxide-assisted glycosylation mechanism that proceeds via solvent separated ion pairs is proposed, whereas the observed α,β-selectivity is substrate-controlled and depends on the thermodynamic equilibrium.

## Introduction

The glycosylation reaction is still one of the most important and basic synthetic strategies in carbohydrate chemistry that provides access to the various types of glycoconjugates [1–4]. Due to the large diversity of glycosyl donors and acceptors there is no general glycosylation method developed so far. To ensure high yielding, as well as regio- and stereoselective glycosidic bond formation, a proper combination of glycosyl donor and acceptor, protecting and leaving groups, promoter, solvent and temperature has to be applied.

In 1981, Mukaiyama et al. introduced glycosyl fluorides [5] as a new class of glycosyl donors [6]. The C–F bond is one of the strongest single bonds in the realm of organic compounds with a bond dissociation energy (BDE) of 570 kJ/mol [7]. Thus, glycosyl fluorides possess a considerably higher thermal and chemical stability compared to the corresponding chlorides (BDE 432 kJ/mol) and bromides (BDE 366 kJ/mol). Due to the advantageous stability during purification, handling and storage, glycosyl fluorides have become widely used glycosyl donors in glycoconjugate synthesis [8–9]. Furthermore, varied reactivity between differentially protected glycosyl fluorides as well as between glycosyl fluorides and other glycosyl donors makes these substrates relevant for more effective glycosylation via orthogonal activation [10–11]. According to the hard–soft acid–base (HSAB) theory the fluoride leaving group is considered to be a hard Lewis base [12–13]. Consequently, a series of fluoride-activating systems containing hard Lewis acidic centers have been published following the first report [7,14–17]. Among these promoters Sn(II) species (SnCl_2_–AgX, X = ClO_4_ or B(C_6_F_5_)_4_) [6,18], group IVB metallocenes (Cp_2_MCl_2_–AgClO_4_, M = Zr, Hf, Ti) [19–21], BF_3_·OEt_2_ [22] and protic acids (TfOH, HClO_4_, HB(C_6_F_5_)_4_) [23] are the most frequently used. During the last decade, apart from reports on novel promoters (Hf(OTf)_4_ [24], InI_3_ [25], In(OTf)_3_ [26], B(C_6_F_5_)_3_ [27]) and coupling partners [28], great attention has been paid to a stereoselective glycosylation by sterically fixed glycosyl fluorides as glycosyl donors [29–31]. The enhanced stability of glycosyl fluorides has also allowed to develop a straightforward protecting-group-free strategy towards oligosaccharides and glycopeptides under basic aqueous conditions [32–33]. Nevertheless, most of the conventional conditions for glycosyl fluoride activation have considerable drawbacks in terms of atom efficiency and environmental impact. These methods generally require (1) stoichiometric amounts of promoters, often heavy metals; (2) multiple additives (co-promoter, molecular sieves, acid scavenger) to facilitate the reaction and/or suppress formation of side-products; (3) low temperatures; (4) complex experimental procedures. Additionally, the majority of the methods reported to date have been applied only for the synthesis of *O*- [4,34–35] and *C*-glycosides [36] and by employing more reactive armed [1] glycosyl fluorides.

In glycosylation reactions the solvent plays a critical role in terms of stabilizing the oxocarbenium ion intermediate and/or affecting the α,β-selectivity [1]. In 2017, Matheu et al. reported a ″green″ glycosylation procedure by employing supercritical CO_2_ (scCO_2_) as a weakly Lewis acidic reaction medium [37]. The method was successfully applied for the synthesis of *O*-glycosides from disarmed glycosyl chlorides and bromides in the absence of additional promoter. Herein we disclose a related concept by applying liquid SO_2_. In contrast to scCO_2_, liquid SO_2_ is a polar Lewis acidic solvent and due to its relatively high boiling point (bp −10 °C) and low vapor pressure (ca. 3 bar at 20 °C) it can be easily liquefied and handled in its liquid state [38]. Advantages of liquid SO_2_ over conventional solvents are: (1) it is aprotic solvent with Lewis acid properties; (2) it dissolves both covalent and ionic compounds [39]; (3) it has good price–quality ratio: ≤5 EUR/kg for the high-purity product (99.98%, H_2_O content ≤50 ppm); (4) it can be easily recycled by changing temperature and/or pressure regimes. The latter approach is used on industrial scale, where processes dealing with a recirculation of SO_2_ in a closed contour are well known. Since the first report by Walden at the beginning of the 20th century [40], a variety of Lewis acid-mediated chemical transformations [41–45], especially those with carbenium ion intermediates [46–56], have benefited from the use of liquid SO_2_ as the reaction medium. To the best of our knowledge, there has been only one example where liquid SO_2_ has been applied as the glycosylation medium to stabilize the oxocarbenium ion formed from glycosyl perchlorate that is generated in situ from glycosyl chloride and AgClO_4_ [56]. Apart from that, SO_2_ has considerable affinity to the Lewis basic halide ions [57–59]. Kuhn et al. [60] and later Eisfield and Regitz [61] have published ab initio studies on the stability of halosulfites HalSO_2_^−^ (Hal = F, Cl, Br or I) that can be formed between halide ions and the SO_2_ molecule. They disclosed that the formation of fluorosulfite anion (FSO_2_^−^) has the highest energy gain and it appears to be stable even in highly polar solvents (ε ≤ 45), while all other halosulfites may dissociate. Thus, we proposed that a plausible formation of the fluorosulfite species and stabilization of the oxocarbenium ion intermediate could facilitate the glycosylation with glycosyl fluorides as glycosyl donors in liquid SO_2_ without the need of external promoter.

## Results and Discussion

We started our study by short screening of the glycosylation conditions in liquid SO_2_ (Table 1). To avoid a potential cleavage of acid-labile protecting groups and to obtain an easily analyzable reaction mixture, pivaloyl-protected mannosyl fluoride α-**1a** as a relatively stable disarmed glycosyl donor was selected as a model substrate. Reactions were carried out in a pressure reactor equipped with a glass tube. By employing a slight excess of 2-phenylethanol (**2a**) as a glycosyl acceptor, the reaction temperature was optimized to 100 °C (Table 1, entry 2). At this temperature full conversion of mannosyl fluoride α-**1a** was achieved and the desired *O*-mannoside **3a** was isolated in a high yield and α-selectivity. Hemiacetal α-**4** was isolated as the only side-product formed via glycosyl donor hydrolysis with the water present in commercial SO_2_ [62]. To note, at lower temperatures (Table 1, entry 1) no reaction was observed and mannosyl fluoride α-**1a** was fully recovered. Recently, Pedersen et al. have studied the vessel effect on the C–F bond activation of glucosyl fluorides [63]. They have proposed an autocatalytic glycosylation by SiF_4_ generated in situ form initially released HF that reacts with silicates of the glassware surface. To clarify the role of a glass vessel in our case, several experiments were carried out in a pressure reactor equipped with a PTFE tube (Table 1, entries 3–5). Under previously optimized reaction conditions (100 °C, Table 1, entry 2), mannoside **3a** was isolated in only 8% yield (Table 1, entry 3). The yield was increased to 23% when acceptor **2a** was added in an excess (3.0 equiv, Table 1, entry 4). Finally, full conversion of fluoride α-**1a** and sufficient yield of desired product **3a** were reached with 3.0 equiv of nucleophile at 150 °C (Table 1, entry 5). Thus, in contrast to the previous report [63], in our case the reaction was not fully stopped by changing the reaction vessel from glass to PTFE tube. At this point, we can confirm the ability of SO_2_ to activate the glycosyl fluoride with a probable co-promoting assistance of a glass vessel. Next, in order to increase the yield of mannoside **3b** formed from a less reactive secondary alcohol **2b**, various additives were tested (Table 1, entries 7–9). Presence of basic molecular sieves (4 Å) as a drying agent led to lower yield and did not suppress the formation of hemiacetal α-**4** (Table 1, entry 7), while no reaction was observed when additives containing a fluorophilic silicon center were used (Table 1, entries 8 and 9). The inhibitory effect of basic molecular sieves may point to the presence and contributory role of protic acid (HF or H_2_SO_3_) in the course of the reaction [64]. Whereas, silyl additives can react with alcohol yielding silyl ether [65–66] that do not react further with glycosyl fluoride α-**1a** under our conditions. The formation of silyl ether was detected in a crude reaction mixture by NMR spectroscopy.

**Table 1:** Screening of conditions for glycosylation in liquid SO_2_.^a^

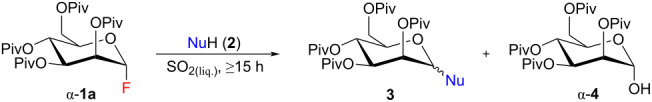							

entry	NuH	(equiv)	*T* (°C)	additive (equiv)	α/β ratio^b^	yield **3** (%)^c^	yield α-**4** (%)^c^

1	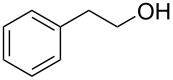 **2a**	1.1	30 to 80	–	NR		
2		1.1	100	–	97:3	**3a**, 87	12
3^d,e^		1.1	100	–	94:6	**3a**, 8	40
4^d,f^		3.0	100	–	96:4	**3a**, 23	35
5^d^		3.0	150	–	97:3	**3a**, 69	30

6	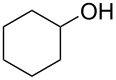 **2b**	1.0	100	–	α-only	**3b**, 67	27
7		1.7	100	4 Å MS	α-only	**3b**, 34	15
8		1.1	100	HMDSO (1.1)	NR		
9		1.7	100	allyl-TMS (2.2)	NR		

^a^Unless otherwise stated, reactions were carried out by using 0.193–0.771 mmol of α-**1a** and 25 ± 5 g of liquid SO_2_ in a pressure reactor containing a glass tube. ^b^Determined by ^1^H NMR analysis of the crude reaction mixture. ^c^Yield of isolated product. ^d^Reaction carried out in a pressure reactor containing a PTFE tube. ^e^53% of α-**1a** was recovered. ^f^48% of α-**1a** was recovered. NR = no reaction; MS = molecular sieves; HMDSO = hexamethyldisiloxane; TMS = trimethylsilyl.

When the optimized model reaction (Table 1, entry 2) between mannosyl fluoride α-**1a** and 2-phenylethanol (**2a**) was carried out in pure conventional solvents (MeCN, THF, toluene or DCM) often used for glycosylation, no reaction was observed (Table 2, entries 1, 4, 6 and 9). Only traces of mannoside **3a** and/or hemiacetal α-**4** were detected by NMR spectroscopy in the presence of H_3_PO_4_ as a protic acid additive having a similar p*K*_a_ value to that of H_2_SO_3_ that is likely to be present in liquid SO_2_ (Table 2, entries 3 and 8) [55]. Thus, the previously considered probable contributory effect of H_2_SO_3_ can be ruled out. Further, in combination with polar aprotic Lewis basic solvents (MeCN, THF) [67] sulfur dioxide was deactivated (Table 2, entries 2 and 5), while in less polar solvents (toluene, DCM) the presence of sulfur dioxide was clearly advantageous and glycoside **3a** was isolated in good yields (Table 2, entries 7 and 10).

**Table 2:** Comparison with conventional solvents.^a^

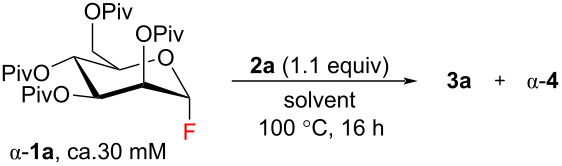		

entry	solvent	yield (%)^b^ (α/β ratio^c^)

0	liquid SO_2_	**3a**, 87 (97:3)

1	MeCN	NR
2^d^	MeCN + SO_2_	NR
3	MeCN + conc.H_3_PO_4_^e^	traces of **3a** & α-**4**

4	THF	NR
5^d^	THF + SO_2_	NR

6	toluene	NR
7^d^	toluene + SO_2_	**3a**, 62% (α-only)
8	toluene + conc.H_3_PO_4_^f^	traces of α-**4**

9	DCM	NR
10^d^	DCM + SO_2_	**3a**, 65% (98:2) α-**4**, 32%

^a^Reactions were carried out in (a) a pressure reactor containing a glass tube for entries 2, 5, 7, 9, and 10; (b) a glass pressure tube for entries 1, 3, 4, 6, and 8. ^b^Yield of isolated product. ^c^Determined by ^1^H NMR analysis of a crude reaction mixture. ^d^Solutions were prepared by bubbling SO_2_ through the selected solvent for 10 min. ^e^1.2 equiv. ^f^1.4 equiv.

Next, the reactivity of various mannosyl halides α-**1a–c** towards *O*- and *S*-nucleophiles were compared under optimized reaction conditions (Table 3). In the case of 2-phenylethanol (**2a**) as an *O*-nucleophile, a similar reactivity, yield of mannoside **3a** and α-selectivity were observed (Table 3, entries 1–3) among all the halides α-**1a–c**, although mannosyl chloride α-**1b** and bromide α-**1c** were not fully consumed. The superior reactivity of glycosyl fluoride α-**1a** in liquid SO_2_ compared to other halides was clearly demonstrated when thiol **2c** was used as an acceptor (Table 3, entries 4–6). *S*-Mannoside **3c** was isolated from mannosyl fluoride α-**1a** in twice as high yield as from the corresponding chloride α-**1b** or bromide α-**1c**. The stability of the latter in liquid SO_2_ at such a high temperature was unexpected due to their generally established labile nature. Additionally, when competitive glycosylation reactions in the presence of both *O*- and *S*-nucleophiles were performed, all mannosyl halides α-**1a–c** gave *O*-mannoside **3a** as the major product in 58 to 71% yield, while overall yield of products **3a,c** varied from 77% for α-**1b** to quant. for α-**1a** (Table S1, Supporting Information File 1).

**Table 3:** Reactivity comparison of mannosyl halides α-**1a–c** in liquid SO_2_.^a^

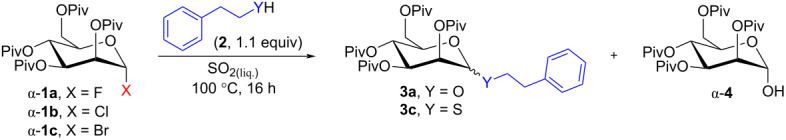								

entry	α-**1**	**2**	composition of a crude reaction mixture (mol %)^b^				α:β ratio^b^	yield **3** (%)^c^

			α-**1**	α-**3**	β-**3**	α-**4**		

1	**a**	**2a** (Y = O)	ND	86	3	11	97:3	**3a**, 87
2	**b**		4	85	2	9	98:2	**3a**, 91
3	**c**		14	80	2	4	98:2	**3a**, 81

4	**a**	**2c** (Y = S)	ND	82	18	ND	82:18	**3c**, 95
5	**b**		46	44	2	8	96:4	**3c**, 46
6	**c**		42	42	10	6	81:19	**3c**, 49

^a^Reactions were carried out by using 0.173–0.771 mmol of α-**1** and 25 ±5 g of liquid SO_2_. ^b^Determined by ^1^H NMR analysis of a crude reaction mixture. ^c^Yield of isolated product.

**Scheme 1:**
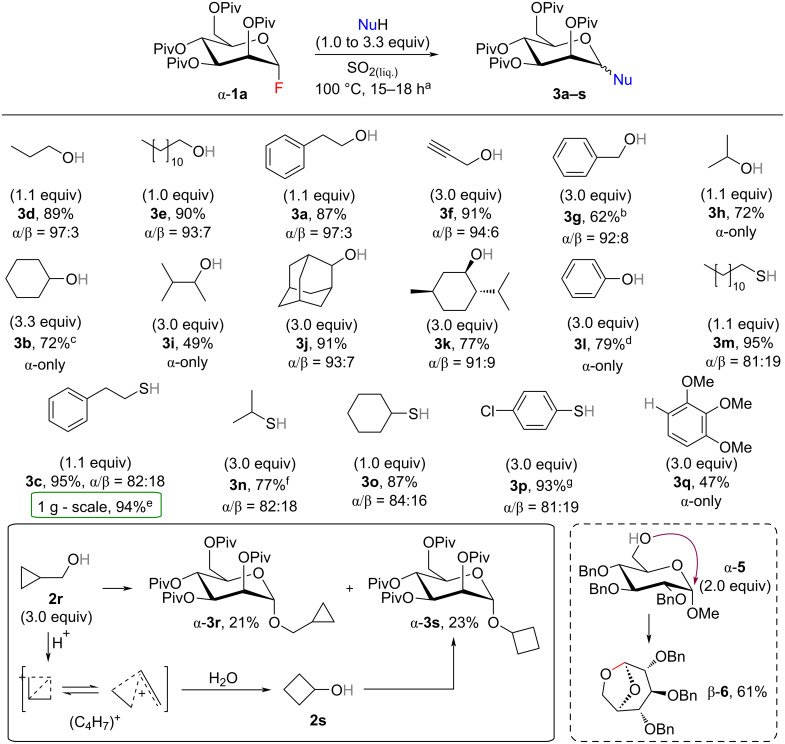
Scope of glycosyl acceptors for glycosylation with pivaloyl-protected mannosyl fluoride α-**1a** in liquid SO_2_. ^a^Unless otherwise stated, reactions were carried out by using 0.193–0.771 mmol of α-**1a** and 25 ± 5 g of liquid SO_2_; α/β ratios were determined by ^1^H NMR analysis of the crude reaction mixture. ^b^56% yield when 1.1 equiv NuH was used. ^c^67% yield when 1.0 equiv NuH was used. ^d^34% yield when 1.0 equiv NuH was used. ^e^Reaction conditions: 1.2 equiv NuH, 43 g liquid SO_2_; α/β = 83:17. ^f^42% yield when 1.1 equiv NuH was used. ^g^59% yield when 1.0 equiv NuH was used.

**Scheme 2:**
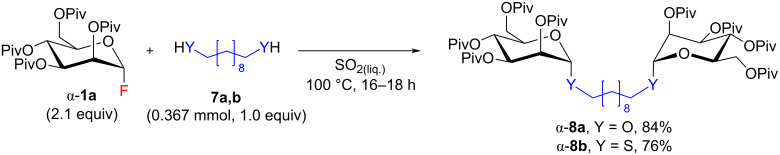
Glycosylation of binucleophiles **7a,b** in liquid SO_2_.

Pivaloyl-protected mannosyl fluoride α-**1a** was further applied for the synthesis of various *O*-, *S*- and *C*-glycosides to demonstrate the scope of acceptors compatible with our glycosylation conditions (Scheme 1). Most of the primary alcohols (**2a**, **2d**–**3f**) were glycosylated in high yields (up to 91%). In the case of less reactive secondary alcohols (**2b**, **2h**, **2j**, **2k**) and phenol (**2l**) better yields were obtained when 3.0 equiv of nucleophile were used. For example, the yield of mannoside **3l** was increased from 34% to 79% when the amount of phenol (**2l**) was changed from 1.0 to 3.0 equiv. Similar reactivity relationships were observed in a series of thiols (**2c**, **2m–p**), but the glycosylation yields comparing to the corresponding alcohols were slightly higher (up to 95%). By employing 2-phenylethanethiol (**2c**), a gram-scale synthesis of mannoside **3c** was successfully demonstrated. Diminished reactivity towards glycosylation of some alcohols (**2g**, **2i**, **2r**) in liquid SO_2_ can be explained by possible formation of stable carbocation species [52]. Thus, in contrast to the other primary alcohols, an excess of 3.0 equiv was required to provide a moderate 62% yield of mannoside **3g** when benzyl alcohol (**2g**) was used as an acceptor. Next, the formation of tertiary carbenium ion from 3-methyl-butan-2-ol (**2i**) via 1,2-hydrogen shift in an initial formed secondary carbocation [52] explains the relatively low yield of mannoside **3i**. The same problem was observed when 1-adamantanol (**S5**) was used as a glycosyl acceptor and the desired mannoside was formed in only 6% NMR yield (Figure S1, Supporting Information File 1). Finally, a mixture of mannosides α-**3r** and α-**3s** was obtained when cyclopropylmethanol (**2r**) was applied. The cyclopropylmethyl carbocation (C_4_H_7_^+^), which is generated in liquid SO_2_ medium, can undergo a rearrangement to form a cyclobutyl carbocation [68]. The latter can be trapped by a water molecule forming cyclobutanol (**2s**) that further reacts with the glycosyl donor. Additionally, our glycosylation approach in liquid SO_2_ was applied for the synthesis of *C*-glycoside **3q** by employing electron-rich 1,2,3-trimethoxybenzene (**2q**). Also binucleophiles **7a** and **7b** were glycosylated with a slight excess of mannosyl fluoride α-**1a** to form bis-mannosides α-**8** in good yields (Scheme 2). In a series of pivaloyl-protected mannosides **3** a substrate-controlled α-selectivity due to the favoring effect of both neighboring ester-type protecting groups and the anomeric effect was observed [3].

On the other hand, mixing of glycosyl donor α-**1a** and 1-*O*-methyl glucoside α-**5** under the developed glycosylation conditions did not provide the expected disaccharide (Scheme 1). Instead, the formation of 1,6-anhydroglucose β-**6** via intramolecular attack [69–70] was detected, while fluoride α-**1a** stayed unchanged. By employing fully protected 1-*O*-methyl glucoside α-**S9** as a glycosyl donor, we have demonstrated that methoxide can act as a mediocre leaving group in liquid SO_2_ (Scheme S1, Supporting Information File 1). Other limitations for the glycosylation with mannosyl fluoride α-**1a** include steric hindrance and the presence of a Lewis basic nitrogen or fluorophilic trimethylsilyl group in the molecule of the glycosyl acceptor (Figure S1, Supporting Information File 1).

To our delight, no cleavage of the pivaloyl protecting groups in liquid SO_2_ medium was observed and the main side-product formed in the series of mannosides **3** was the previously mentioned tetra-*O*-pivaloyl mannopyranose α-**4**. In some experiments traces of 1,1'-mannoside α,α-**S14** formed in the reaction between hemiacetal α-**4** and glycosyl donor *α*-**1a** were detected (see Supporting Information File 1) [71].

**Scheme 3:**
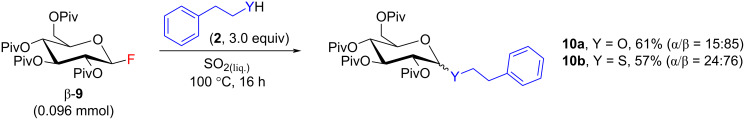
Pivaloyl-protected glucosyl fluoride β-**9** as a glycosyl donor in liquid SO_2_.

Further, we turned our attention to the reactivity of other glycosyl fluorides in liquid SO_2_. We continued with pivaloyl-protected glucosyl fluoride β-**9** (Scheme 3). The reaction conditions were optimized to 100 °C and 3.0 equiv of nucleophile (Table S3, Supporting Information File 1). The target glucosides **10** were obtained in a moderate yield and β-selectivity induced through the neighboring ester type protecting group assistance. At lower temperatures the glycosylation yield was lower, although full conversion of glucosyl fluoride β-**9** was still observed. Compared to the analogue mannose derivative α-**1a**, glucose β-**9** turned out to be less stable and more prone to various side-reactions. A series of side-products formed by hydrolysis and protecting group migrations were detected and their structures are proposed (see Supporting Information File 1).

Next, glycosyl fluorides α-**11** and α-**12** containing more acid-sensitive acetyl protecting groups were applied for the glycosylation of 2-phenylethanol (**2a**) and 2-phenylethanethiol (**2c**) in liquid SO_2_ (Table 4). A temperature screening was performed to identify optimal reaction conditions (Table S4, Supporting Information File 1). The acetyl-protected mannosyl fluoride α-**11** gave the desired mannosides **13** in a moderate yield and α-selectivity. The latter was comparable to the selectivity observed for the pivaloyl-protected mannosides **3**. This time a couple of mono-deprotected side-products was observed (see Supporting Information File 1). The reactivity of acetyl-protected glucosyl fluoride α-**12** was similar to that of mannose α-**11**. Glucosides **14** were isolated in a moderate yield, but without any α,β-selectivity due to the mismatched interaction between the anomeric effect and neighboring protecting group assistance. The diminished selectivity compared to the series of pivaloyl-protected glucosides **10** leads to the conclusion that the Lewis basic carbonyl oxygen of the acetyl group is more coordinated and less nucleophilic in liquid SO_2_ than the carbonyl oxygen of the pivaloyl group. The profile of side-products in this glucose series was similar to that observed for fluoride β-**9** (see Supporting Information File 1).

**Table 4:** Acetyl protected manno- and glucopyranosyl fluorides α-**11** and α-**12** as glycosyl donors in liquid SO_2_.^a^

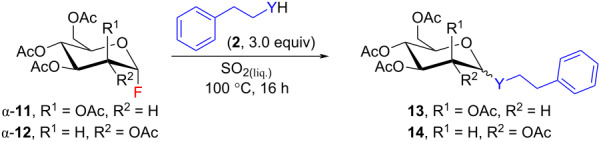				

entry	glycosyl fluoride	Y (**2**)	α/β ratio^b^	yield (%)^c^

1	α-**11**	O	91:9	**13a**, 55
2		S	78:22	**13b**, 67

3	α-**12**	O	54:46	**14a**, 43
4		S	48:52	**14b**, 76

^a^Reactions were carried out in a scale of 0.277–0.300 mmol (α-**11** or α-**12**). ^b^Determined by ^1^H NMR analysis of a crude reaction mixture. ^c^Yield of isolated product.

**Scheme 4:**
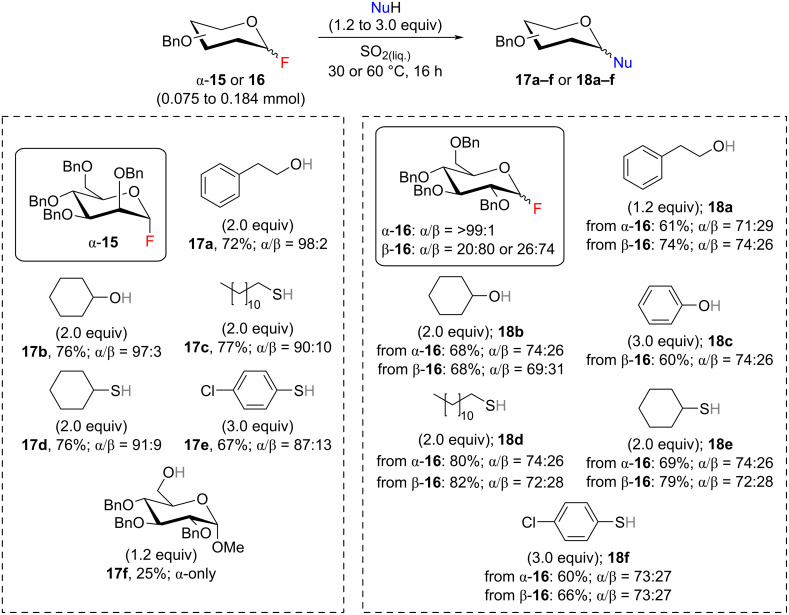
Benzyl protected manno- and glucopyranosyl fluorides α-**15** and **16** as glycosyl donors in liquid SO_2_. Reactions were carried out at 30 °C for mannosyl fluoride α-**15** and glucosyl fluoride α-**16**; at 60 °C for glucosyl fluoride β-**16**. Anomeric ratios were determined by HPLC analysis.

The armed benzyl-protected glycosyl fluorides α-**15** and **16** were more reactive than their acylated analogues and the corresponding glycosides **17** and **18** were obtained at lower temperatures (Scheme 4). The reaction temperature for mannosyl fluoride α-**15** was optimized to 30 °C (Table S5, Supporting Information File 1). At higher temperature desired mannoside **17a** was not observed, whereas at −10 °C its yield was decreased. Under optimal conditions mannosides **17a–e** were obtained in good yields and α-selectivity. Importantly, due to increased reactivity of glycosyl donor α-**15** at lower temperature, we have also managed to obtain disaccharide **17f**, albeit in low yield. Interestingly, a different temperature regime was adopted for benzyl-protected glucosyl fluoride **16** depending on its anomeric ratio (Table S6, Supporting Information File 1). Thus, the glycosylation temperature for the glucosyl fluoride containing an excess of β-anomer β-**16** was optimized to 60 °C, while for the more reactive α-anomer α-**16** it was decreased to 30 °C. Regardless of the anomeric ratio, the desired *O*- and *S*-glucosides **18a–f** were isolated in good yields. Besides, glycosylation of primary nucleophiles with benzyl-protected glucosyl fluoride gave better yields (**18a** and **18d**) than with the corresponding acylated analogues β-**9** and α-**11** described above. It was also found that the glycosylation stereoselectivity with glucosyl fluoride **16** did not depend on the anomeric ratio of glucosyl fluoride **16**: both anomers of **16** yielded glucosides **18** in similar anomeric ratios with excess of the α-anomer. As expected, in the absence of an ester type protecting group at C2 position, for both series of benzyl protected glycosides α-selectivity was observed solely due to the anomeric effect.

**Scheme 5:**
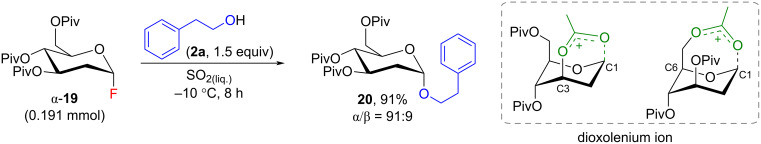
2-Deoxy glycosyl fluoride α-**19** as a glycosyl donor in liquid SO_2_.

The Lewis acidic medium of liquid SO_2_ was also facilitating for the synthesis of 2-deoxy glucoside **20** from corresponding fluoride α-**19** in 91% yield and good α-selectivity at −10 °C (Scheme 5). Due to the absence of a neighboring group at C2 position, the stereoselective synthesis of 2-deoxy glycosides is challenging [72–74]. We hypothesize that the stabilization of the oxocarbenium ion intermediate in a form of a dioxolenium ion by the remote protecting group in C3 or C6 position could be the reason for such a good α-selectivity in liquid SO_2_ [75].

Within this study, several experiments were also carried out to test the reactivity of peracylated manno- and glucopyranoses in liquid SO_2_ (Table S7, Supporting Information File 1). Most of these glycosyl donors were not fully consumed at 100 °C and formed a complex mixture of monosaccharides.

Finally, in order to make our glycosylation procedure more attractive and more accessible to the synthetic community we have demonstrated an application of saturated solutions of SO_2_ in conventional solvents that do not require a specific equipment, but can be performed in widely available glass pressure tubes (Table 5). In this context it has technically a similarity with ammonia solutions in organic solvents. We prepared saturated SO_2_ solutions in toluene and DCM. The concentration of SO_2_ in saturated solutions was determined by iodometric titration. As shown in Table 5, higher yields were obtained in DCM solutions. The yield of *O*-mannoside **3a** was even higher when the glycosylation between mannosyl fluoride α-**1a** and 2-phenylethanol (**2a**) was performed in a gram-scale by applying a solvent/substrate ratio of 10:1 (mL/g) (Table 5, entries 2 and 5). A diminished yield was observed for *S*-mannoside **3c** when the glycosylation was carried out in saturated DCM solution (64%) instead of pure SO_2_ (95%) (Table 5, entry 6). No difference was observed between the yields of thioglucoside **18d** in liquid SO_2_ or its solution in DCM (Table 5, entry 7).

**Table 5:** Glycosylation with mannosyl fluoride α-**1a** and glucosyl fluoride α-**16** in saturated SO_2_ solutions.^a^

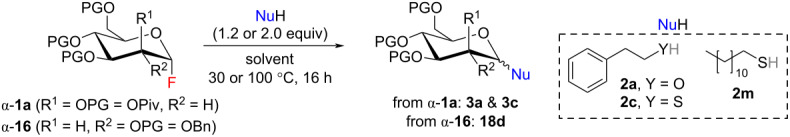							

entry	glycosyl fluoride	scale (g)	solvent	conc. (mL/g)	NuH	α/β ratio^b^	yield (%)^c^

1	α-**1a**	0.2	2.7 M SO_2_ in toluene	75	**2a**	>99:1	**3a**, 66
2		1.5		10	**2a**	95:5	**3a**, 75
3		0.1		75	**2c**	90:10	**3c**, 32

4	α-**1a**	0.2	2.0 M SO_2_ in DCM	75	**2a**	98:2	**3a**, 84
5		1.5		10	**2a**	96:4	**3a**, 94
6		0.1		75	**2c**	86:14	**3c**, 64
7	α-**16**	0.1		20	**2m**	64:36	**18d**, 84

^a^Reactions were carried out in glass pressure tubes; reaction conditions: (entries 1–6) 1.2 equiv NuH, at 100 °C; (entry 7) 2.0 equiv NuH, at 30 °C. ^b^Determined by ^1^H NMR (entries 1–6) or HPLC (entry 7) analysis of a crude reaction mixture. ^c^Yield of isolated product.

By employing benzyl-protected glucosyl fluoride **16** with different anomeric ratios, we have demonstrated that the stereochemical outcome of the glycosylation in liquid SO_2_ does not depend on the configuration of the anomeric center of the glycosyl donor. This observation points to the formation of a solvent-separated ion pair (SSIP) between the oxocarbenium ion and a counteranion, for example, fluorosulfite. At the same time, according to the Lewis base properties characterized by lithium cation basicity (LiCB) liquid SO_2_ (76.3) is similar to DCM (83) [67]. Thus, liquid SO_2_ could be classified as a non-coordinating solvent that unlikely coordinates to the oxocarbenium ion intermediate and affects the glycosylation stereoselectivity [1]. As a result, we can conclude that the stereoselectivity of the glycosylation in liquid SO_2_ is substrate-controlled and approaches a thermodynamic equilibrium determined by the anomeric effect or interference of both the anomeric effect and the assistance of the neighboring ester-type protecting group.

Next, we have also observed that the anomerization of the glycosylated products towards their thermodynamic equilibrium is promoted by the species formed during the glycosylation reaction [76]. Thus, when anomerically pure thiomannoside β-**3c** was subjected to the glycosylation conditions (100 °C, 16 h) in liquid SO_2_ without any additives, no anomerization was observed and the tested substrate β-**3c** was almost fully recovered. In contrast, when the same thiomannoside β-**3c** was added to the glycosylation mixture containing 1-dodecanethiol (**2m**) and mannosyl fluoride α-**1a** or bromide α-**1c** (Table 6), anomerization occurred approaching the anomeric ratio observed initially for mannoside **3c** (α/β = 82:18, Scheme 1).

**Table 6:** Anomerization of thiomannoside β-**3c** under glycosylation conditions.

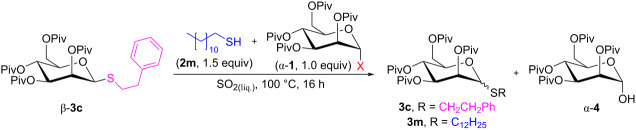							

entry	α-**1**	composition of a crude reaction mixture (mol %)^a^				α/β ratio^a^	
	
		**3c**	**3m**	α*-***1**	α-**4**	**3c**	**3m**

1	**a** (X = F)	48	35	ND	17	71:29	82:18
2	**c** (X = Br)	39	48	13	ND	81:19	82:18

^a^Determined by ^1^H NMR analysis of a crude reaction mixture.

**Figure 1:**
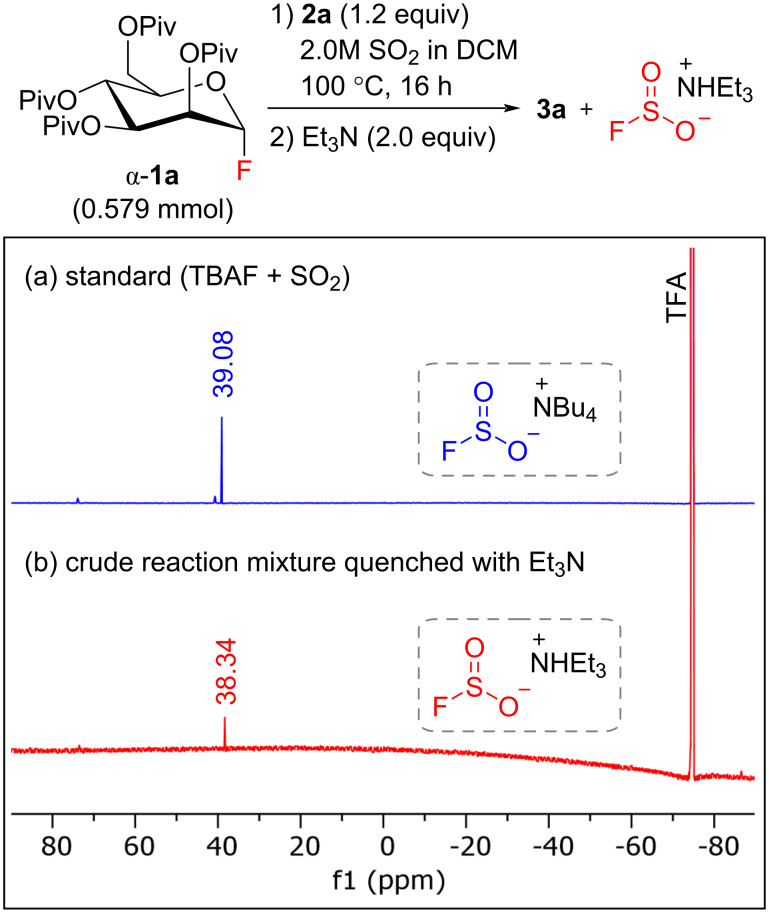
Detection of the FSO_2_^−^ species by ^19^F NMR (471 MHz, D_2_O).

Finally, we proved the formation of the fluorosulfite species by employing ^19^F NMR spectroscopy (Figure 1). Glycosylation of the reaction mixture was treated with Et_3_N to stabilize the possibly formed fluorosulfite anions in form of an ammonium salt. The ^19^F NMR spectra of the water-soluble components was than compared to the standard obtained from the reaction between TBAF and SO_2_. The peak that corresponds to the FSO_2_^−^ anion was observed at 38.34 ppm (TFA as an external standard, −76.55 ppm) [77].

**Figure 2:**
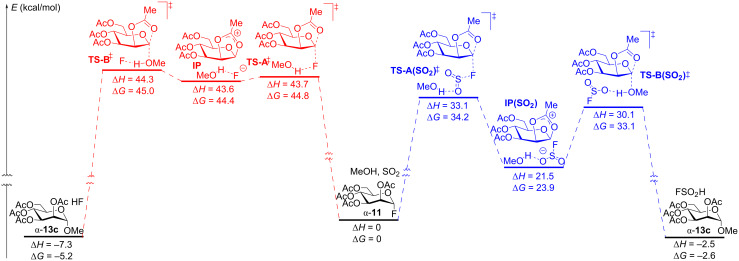
Computational study of reaction mechanism α-**11** + MeOH → α-**13c** in the presence of and in absence of SO_2_ (Gaussian 09, Revision D.01; Gaussian, Inc.; m052x method and the 6-31+g(d) basis set). Enthalpy and Gibbs free energy values referenced against the starting value for the substrates and catalyst are given in kcal/mol.

Also DFT calculations were performed on the model reaction α-**11** + MeOH → α-**13c** to elucidate the influence of SO_2_ on the dissociation of the glycosidic C–F bond [78] (Figure 2). Indeed, it was found that the coordination of the Lewis acidic SO_2_ to the fluoride (transition state TS-A^≠^ versus TS-A(SO_2_)^≠^) decreases the C–F bond dissociation energy (ΔΔG) by 10.6 kcal/mol. The formation of the neighboring group stabilized the oxocarbenium ion (dioxolenium ion) and its reaction with alcohol leads to the experimentally observed glycosides and the FSO_2_H adduct. We assume that due to the formation of the latter also substrates, which do not possess the participating group at C2 position, still react through the oxocarbenium ion intermediate.

## Conclusion

In summary, novel sulfur dioxide-assisted and metal-free glycosylation conditions by employing a combination of glycosyl fluoride as the glycosyl donor and liquid SO_2_ as both solvent and promoter have been developed. Due to the absence of any external additive, the presented method is considered to be an atom efficient and environmentally friendly synthetic approach. The glycosylation conditions in liquid SO_2_ have been optimized for both disarmed and armed mannose- and glucose-derived glycosyl fluorides, and novel conditions have been successfully applied for the synthesis of *O*-, *S*- and *C*-glycosides in moderate to excellent yields. The glycosylation in liquid SO_2_ is proposed to proceed via a solvent-separated ion pair and with stereoselectivity that is substrate-controlled and presents a thermodynamic equilibrium. The latter was clearly demonstrated when the more challenging 2-deoxyglucosyl fluoride was used as a glycosyl donor and the assistance of a remote acyl-protecting group provided good α-selectivity. The initially proposed formation of the fluorosulfite species during the glycosylation in liquid SO_2_ was proved by employing ^19^F NMR spectroscopy and DFT calculations. Finally, a more conventional experimental procedure has been provided for the application of saturated SO_2_ solution in DCM or toluene. This protocol does not require specific equipment and the reactions can be performed in widely available glass pressure tubes.

## Supporting Information

File 1 Experimental procedures; experimental data for synthesized compounds; competitive glycosylation of *O*- and *S*-nucleophiles; problematic glycosyl acceptors; reaction optimization data; reactivity of other glycosyl donors; proposed structures of side-products; detailed description of ^19^F NMR studies; stability tests for various glycosyl donors.

File 2 Copies of NMR spectra.

File 3 DFT calculations.
