# Supplementary material for: Metal-free glycosylation with glycosyl fluorides in liquid SO2
Source: Beilstein J Org Chem. 2021 Apr 29;17:964–76. doi: 10.3762/bjoc.17.78 (PMC8093551; doi:10.3762/bjoc.17.78)
Supplement: File 1 — Experimental procedures; experimental data for synthesized compounds; competitive glycosylation of O- and S-nucleophiles; problematic glycosyl acceptors; reaction optimization data; reactivity of other glycosyl donors; proposed structures of side-products; detailed description of 19F NMR studies; stability tests for various glycosyl donors. [file Beilstein_J_Org_Chem-17-964-s001.pdf]

BEILSTEIN JOURNAL OF ORGANIC CHEMISTRY

Supporting Information

for

Metal-free glycosylation with glycosyl fluorides in liquid SO_2

Krista Gulbe, Jevgeņija Lugiņina, Edijs Jansons, Artis Kinens and Māris Turks

Beilstein J. Org. Chem. **2021,** *17,* 964–976. doi:10.3762/bjoc.17.78

Experimental procedures; experimental data for synthesized compounds; competitive glycosylation of *O*- and *S*-nucleophiles; problematic glycosyl acceptors; reaction optimization data; reactivity of other glycosyl donors; proposed structures of side-products; detailed description of ^{19}F NMR studies; stability tests for various glycosyl donors

License and Terms: This is a supporting information file under the terms of the Creative Commons Attribution License (https://creativecommons.org/licenses/by/4.0). Please note that the reuse, redistribution and reproduction in particular requires that the author(s) and source are credited and that individual graphics may be subject to special legal provisions.
The license is subject to the *Beilstein Journal of Organic Chemistry* terms and conditions: (https://www.beilstein-journals.org/bjoc/terms)

Contents

1. General experimental details ... S1-2
2. Experimental procedures ... S1-3
3. Competitive glycosylation of *O*- and *S*-nucleophiles in liquid SO_2 ... S1-6
4. Problematic glycosyl acceptors ... S1-7
5. Reaction optimization data ... S1-8
6. Reactivity of other glycosyl donors in liquid SO_2 ... S1-10
7. Proposed structures of side-products ... S1-11
8. ^{19}F NMR studies ... S1-12
9. Stability of various glycosyl donors in liquid SO_2 ... S1-14
10. Experimental data for synthesized compounds ... S1-15
11. References ... S1-40

1. General experimental details

Unless otherwise stated, reactions in liquid SO_2 were carried out in (1) a stainless steel pressure reactor equipped with a glass tube at temperatures above –10 °C; (2) a two-neck round-bottom flask equipped with a dry ice condenser at temperature –10 °C. Reactions in conventional solvents or in saturated SO_2 solutions were carried out in (1) a stainless steel pressure reactor equipped with a glass tube; (2) a glass pressure tube sealed with a PTFE screw cap. Inert atmosphere or highly anhydrous conditions were not provided.

Commercially available reagents were used as received. Conventional solvents were distilled prior to use. THF was distilled from sodium under argon atmosphere.

The reaction mixture and chromatographic purification were monitored by thin-layer chromatography (TLC) on E. Merck Kieselgel 60 F254 with detection by UV light (254 nm) or visualization with potassium permanganate or cerium sulfate stain. Column chromatography was performed on ROCC (60 Å, 40–60 µm) or ZEOprep (60 Å, 15–40 µm) silica gel.

NMR spectra were recorded on a Bruker Avance 300 or Bruker Avance 500 spectrometer in $CDCl_3$. ^{1}H NMR spectra were recorded at 300 or 500 MHz. ^{13}C NMR spectra were recorded at 75.5 or 126 MHz. The chemical shifts (δ) are reported in ppm. The residual solvent peak was used as an internal reference (7.26 and 77.16 ppm for ^{1}H NMR and ^{13}C NMR, respectively). The coupling constants (J) are given in hertz (Hz) and reported with the following abbreviations: s (singlet), d (doublet), dd (doublet of doublets), ddd (doublet of doublet of doublets), dddd (doublet of doublet of doublet of doublets), t (triplet), dt (doublet of triplets), td (triplet of doublets), tt (triplet of triplets), quint (quintet), m (multiplet), br (broad signal). For quantitative ^{1}H NMR, the relaxation time was increased (d1 = 10). ^{19}F NMR spectra were recorded on a Bruker Avance 500 spectrometer at 471 MHz in D_2O and referenced to the trifluoroacetic acid (TFA) as an external reference standard (–76.55 ppm vs. $CFCl_3$).

High-performance liquid chromatography (HPLC) was performed on an Agilent 1200 series chromatograph coupled with UV detector (190–370 nm). HPLC analysis were performed using normal-phase HPLC column (Zorbax RX-SIL, 4.6 × 250 mm, 5 µm) and isocratic elution mode (eluent: 89% hexanes and 11% methyl tert-butyl ether (MTBE); flow rate 1.6 mL/min).

Specific optical rotations ([α]) were measured at room temperature (24 °C) on an Anton Paar MCP 500 polarimeter using 10 mm cell and sodium light (D line at 589 nm).

High-resolution mass spectra (HRMS) (electrospray ionization (ESI)) were recorded on an Agilent 1290 Infinity series ultra-high pressure liquid chromatograph connected to an Agilent 6230 time-of-flight mass spectrometer.

Unless otherwise stated, reported anomeric (α:β) ratios demonstrate compound distribution in a crude reaction mixture determined by ^{1}H NMR or HPLC analysis.

2. Experimental procedures

Synthesis of glycosyl donors

Glycosyl donors α-**1a**[1], α-**1c**[2], β-**9**[3], α-**11**[4], α-**12**[4], α-**15**[5], α-**16**[5], β-**16**[6], α-**S9**[7], **S10**[2], β-**S11**[8], **S12**[9] and β-**S13**[10] were synthesized according to the reported procedures. To confirm purity of these compounds, ^{1}H and ^{13}C NMR spectra are provided.

Synthesis of 2,3,4,6-tetra-*O*-pivaloyl-α-D-mannopyranosyl chloride (α-1b). The previously reported reaction conditions were adapted.[11] $SnCl_2$ (1.0 equiv) and $SOCl_2$ (2.0 equiv) were added to a solution of pentapivaloyl mannopyranose **S10** (2.0 g, 3.33 mmol, 1.0 equiv) in anhydrous DCM (30 mL) under an inert atmosphere (Ar). The reaction mixture was stirred at room temperature until complete consumption of the starting material was observed by TLC (7 h). The reaction mixture was then washed with H_2O (30 mL). The organic phase was neutralized with saturated aqueous $NaHCO_3$ solution to pH ≥ 7 and sequentially washed with H_2O (20 mL) and brine (20 mL). The organic layer was dried over anhydrous Na_2SO_4, filtered and concentrated under reduced pressure. The crude residue was fractionated by column chromatography (EtOAc/hexanes) on silica gel that was prior treated with 0.2% Et_3N solution in MeOH. Mannosyl chloride *α*-**1b** was isolated as a white amorphous solid (1.66 g, 93%).

Synthesis of 3,4,6-tri-*O*-pivaloyl-2-deoxy-α-D-glucopyranosyl fluoride (*α*-19). For the synthesis of fluoride *α*-**19** following reaction sequence was applied:

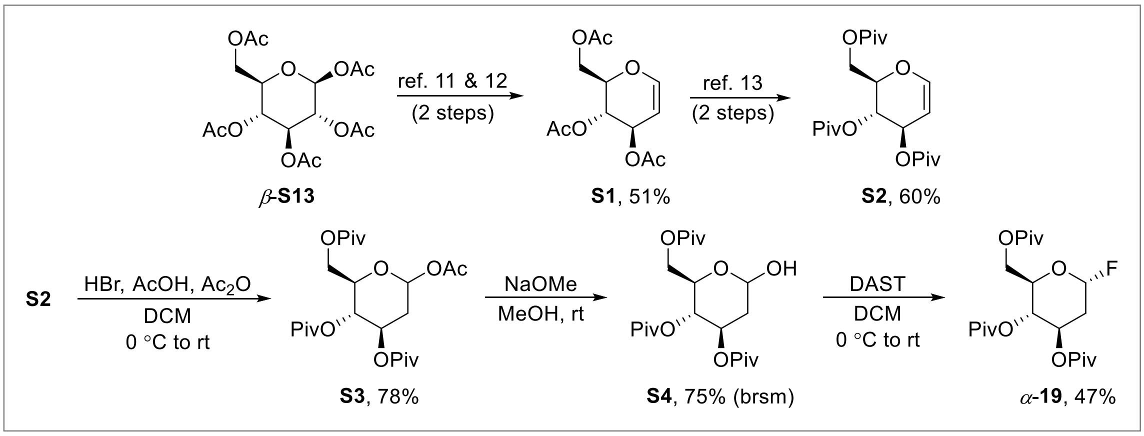

Initially, penta-acetyl glucopyranose *β*-**S13** was transformed to pivaloyl protected glucal **S2** *via* acetyl protected glucal **S1** according to the reported procedures.[11-13]

*1-O-Acetyl-3,4,6-tri-O-pivaloyl-2-deoxy-D-glucopyranose (**S3**).* Previously reported reaction conditions were adapted.[14] Pivaloyl protected glucal **S2** (2.56 g, 6.42 mmol, 1.0 equiv) was dissolved in anhydrous DCM (12 mL). Solution was then purged with nitrogen for 10 min and cooled to 0 °C. AcOH (3.5 mL) and Ac_2O (5 mL) were added. The reaction mixture was left stirring for 15 min. 33% HBr in acetic acid solution (0.3 equiv of HBr) was added. The resulting solution was allowed to warm to room temperature and left stirring for 19 h. The reaction was poured into ice-cold H_2O (60 mL) and diluted with DCM (50 mL). The organic phase was separated and washed with ice-cold H_2O (3 × 30 mL), ice-cold saturated aqueous $NaHCO_3$ solution (1 × 30 mL) and ice-cold brine (1 × 30 mL). DCM solution was dried over anhydrous Na_2SO_4, filtered and evaporated under reduced pressure. The residue was fractionated by column chromatography (EtOAc/hexanes). Compound **S3** was isolated as a yellowish amorphous solid (2.28 g, 78%, α:β = 81:19 (NMR, isolated)).

*3,4,6-Tri-O-pivaloyl-2-deoxy-D-glucopyranose (**S4**).* Previously reported reaction conditions were adapted.[13] 2-Deoxyglucopyranose **S3** (2.28 g, 4.97 mmol, 1.0 equiv) was dissolved in anhydrous MeOH (10 mL) under inert atmosphere (N_2). NaOMe (0.5 equiv) was added and the solution was left stirring at room temperature for 9 h. The reaction mixture was neutralized (pH ~7) with Dowex 50W8 (100–200 mesh) ion exchange resin, filtered and concentrated under reduced pressure. The residue was fractionated by column chromatography (EtOAc/hexanes). Compound **S4** was isolated as a white amorphous solid (1.15 g, 75% (brsm), α:β = 77:23 (NMR, isolated)).

*3,4,6-Tri-O-pivaloyl-2-deoxy-α-D-glucopyranosyl fluoride (α-**19**).* Previously reported reaction conditions were adapted.[6] 2-Deoxyglucose **S4** (1.15 g, 2.76 mmol, 1.0 equiv) was dissolved in anhydrous DCM (11 mL) under inert atmosphere (N_2). The resulting mixture was cooled to 0 °C and solution of DAST (2.7 equiv) in anhydrous DCM (5 mL) was added. The reaction was allowed to warm to room temperature and then left stirring for 3 h. The reaction mixture was poured into a saturated aqueous $NaHCO_3$ solution (100 mL). Biphasic solution was extracted with DCM (3 × 50 mL). Organic layers were collected, dried over anhydrous Na_2SO_4, filtered and concentrated under reduced pressure. The residue was fractionated by column chromatography (EtOAc/hexanes). Compound α-**19** was isolated as a white amorphous solid (550 mg, 47%). ***Note:** another anomer β-**19** that was formed in 41% yield degraded during the storage at –4 °C.*

Glycosylation procedures

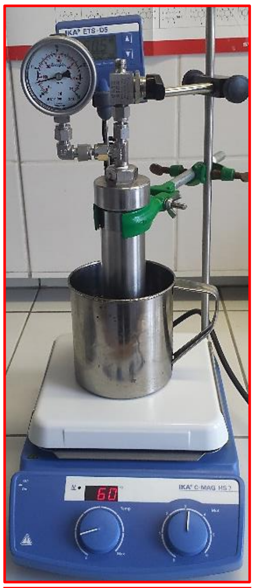

Method A

***Method A:* General procedure for glycosylation in liquid SO_2.** Glycosyl donor and glycosyl acceptor were placed into a stainless steel reactor equipped with a glass tube and a magnetic stirring bar. The reactor was sealed and cooled in a dry ice-ethanol bath to –78 °C. Sulfur dioxide was then transferred from storage cylinder to the reaction vessel by distillation. The reactor was sealed and warmed to room temperature. The reaction was performed under pressure at optimized temperature for 16 h. Then, the reactor was cooled to room temperature (ice bath) and connected to either the storage cylinder of SO_2 for the solvent recycling or a trap containing aqueous $NaHCO_3$ solution for SO_2 removal. The residue was dissolved in DCM and transferred to a flask. After solvent evaporation under reduced pressure, the crude residue was analyzed by ^{1}H NMR or HPLC* to determine anomeric ratio and then fractionated by column chromatography (EtOAc/hexanes) to provide pure product(s).

**Sample for HPLC analysis was prepared in hexanes with concentration approx. 1 mg/1 mL.*

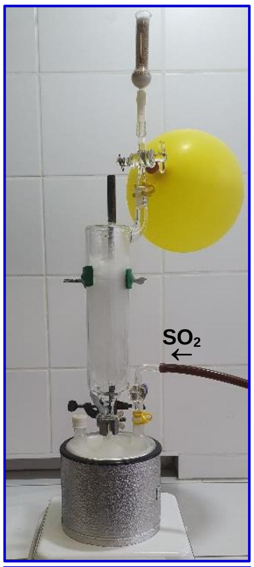

Method B

***Method B:* Glycosylation in liquid SO_2 at –10 °C (for Scheme 5).** 2-Deoxy glucosyl fluoride α-**19** (80 mg, 0.191 mmol, 1.0 equiv) and 2-phenylethanol (**2a**, 1.5 equiv) were placed into an oven-dried two-neck flask (50 mL) equipped with a magnetic stirring bar. The flask was than equipped with a dry ice condenser and placed in a Dewar flask. The system was flushed with nitrogen and cooled to –78 °C by employing dry ice-ethanol mixture as a cooling agent. Sulfur dioxide (~25 mL) was then transferred from storage cylinder to the reaction vessel by distillation. The Dewar flask was removed, and the reaction was stirred at –10 °C for 7 h. Dry ice condenser was removed and SO_2 was evaporated and trapped in aqueous $NaHCO_3$ solution within an hour. The residue was analyzed by ^{1}H NMR to determine anomeric ratio and then fractionated by column chromatography (EtOAc/hexanes) to provide glucoside **20** (90.1 mg, 91%, α:β = 91:9).

***Method C:* General procedure for glycosylation when saturated SO_2 solution in DCM or toluene was used.** Glycosyl donor and glycosyl acceptor were placed into a glass pressure tube equipped with a magnetic stirring bar. Saturated solution of SO_2 in DCM (2.0M) or toluene (2.7M) was added. The glass tube was sealed with a PTFE screw cap. The reaction was left stirring at optimized temperature for 16 h. Then, the reaction was allowed to cool to room temperature and transferred to a flask. After solvent evaporation under reduced pressure, the crude residue was analyzed by ^{1}H NMR or HPLC* to determine anomeric ratio and then fractionated by column chromatography (EtOAc/hexanes) to provide pure product(s).

** Sample for HPLC analysis was prepared in hexanes with concentration approx. 1 mg/1 mL.*

Preparation of saturated SO_2 solutions in DCM and toluene[15]

SO_2 storage cylinder was connected to empty gas wash bottle (**A**) that was connected to the gas wash bottle (**B**) filled with distilled DCM or toluene (~100 mL). This was further connected to the empty gas wash bottle (**C**) and bottle (**D**) filled with diluted aqueous NaOH solution (300 mL). While cooling DCM or toluene containing wash bottle in an ice bath (0 °C), SO_2 gas was slowly run through the system until solvent solution started to bubble vigorously (~15 min). Concentrations were determined by iodometric titration and was found to be 2.0M and 2.7M for DCM and toluene, respectively. Both SO_2 solutions were used further without dilution.

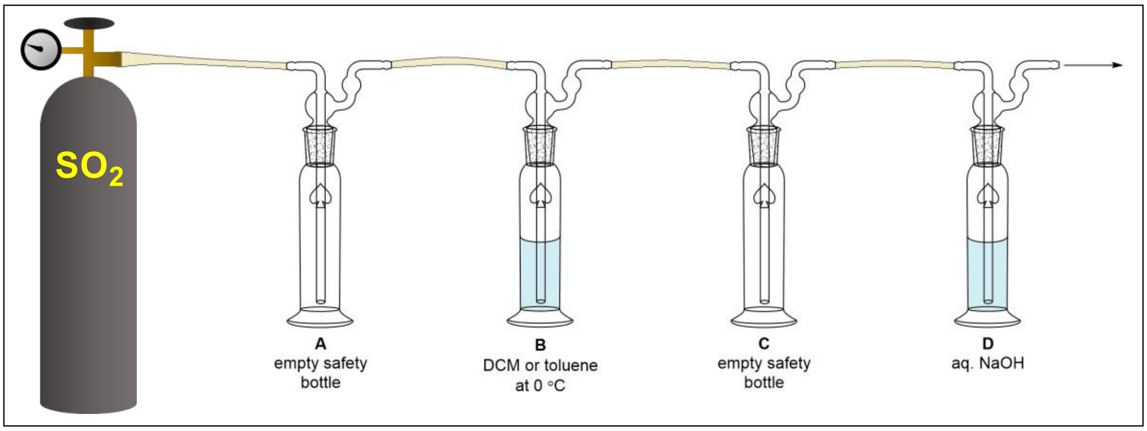

The saturated SO_2 solutions in DCM and toluene were iodometric titrated:

I_2 (0.765–0.916 mmol, 1.0 equiv) and KI (1.4 equiv) were weighed in 100 mL Erlenmeyer flask and diluted with MeCN/H_2O 1:1 (10 mL). Saturated solution of SO_2 in DCM or toluene (1 mL) was diluted in a volumetric flask (10 mL) with MeCN to obtain 1:10 dilution. From the diluted solution 2 mL was added to the Erlenmeyer flask containing I_2 solution. The residual I_2 was back-titrated with standardized aqueous solution of $Na_2S_2O_3$ (0.1N). Color loss of I_2 solution indicated titration endpoint.

The residual I_2 was calculated according to equation (1) and then amount of SO_2 was calculated from equation (2):

$$I_2 + 2Na_2S_2O_3 \rightarrow Na_2S_4O_6 + 2NaI \quad (1)$$

$$SO_2 + I_2 + H_2O \rightarrow SO_3 + 2HI \quad (2)$$

3. Competitive glycosylation of *O*- and *S*-nucleophiles in liquid SO_2

Table S1: Competitive glycosylation of *O*- and *S*-nucleophiles with mannosyl halides *α*-**1a-c** in liquid SO_2.[a]

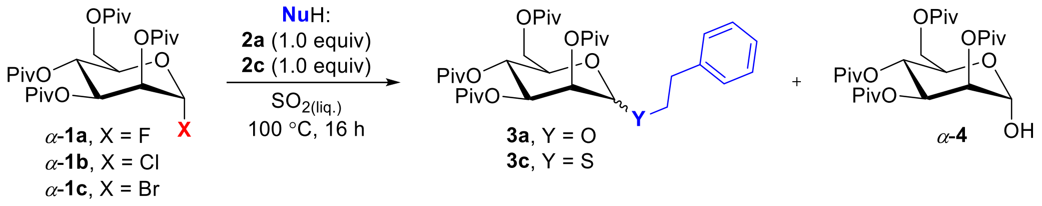

entry	α-1	composition of a crude reaction mixture (mol%)[b]						α:β ratio[b]		3a:3c ratio[b]	yield (%)[b,c]	
		α-1	α-3a	β-3a	α-3c	β-3c	α-4	3a	3c		3a	3c
**1**	**a**	ND	66	3	24	5	2	96:4	83:17	70:30	71	29
**2**	**b**	11	59	<1	19	<1	9	99:1	96:4	75:25	58	19
**3**	**c**	8	68	2	15	4	3	97:3	79:21	79:21	63	17

[a]Reactions were carried out in a scale of 80 to 200 mg (α-**1**). [b]Determined by ^1H NMR analysis of a crude reaction mixture. [c]NMR yield; 1,2,3-trimethoxybenzene as an internal standard. ND = not detected

4. Problematic glycosyl acceptors

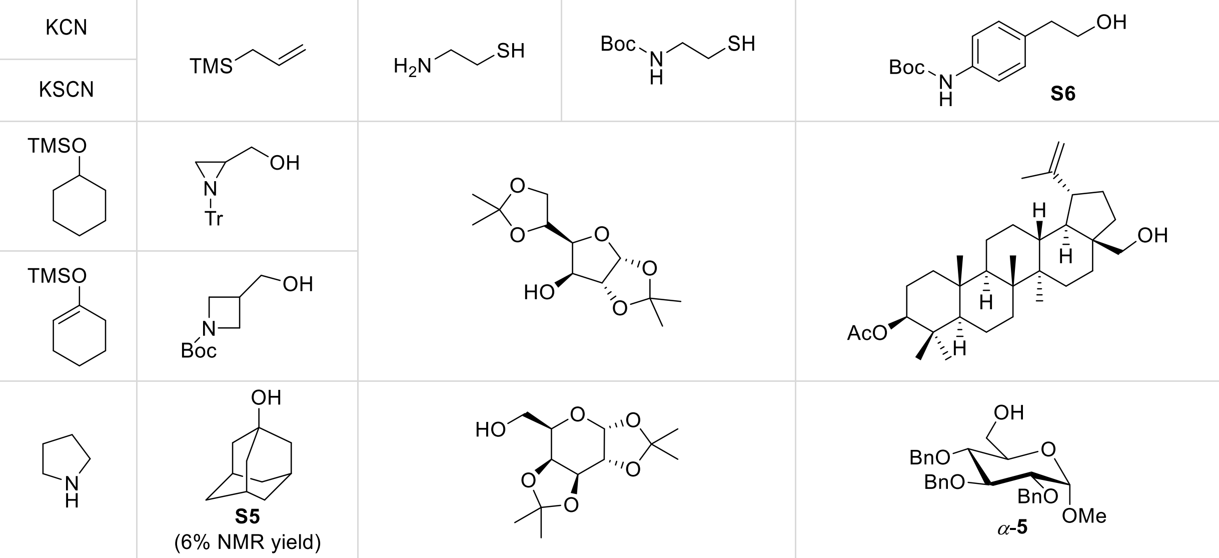

Figure S1: Tested nucleophiles that did not react with mannosyl fluoride α-**1a** in liquid SO_2 (100 °C, ≥14 h, 1.0 to 3.0 equiv of NuH).

Table S2: Glycosylation attempts between mannosyl fluoride α-**1a** and glycosyl acceptor **S6** in liquid SO_2.

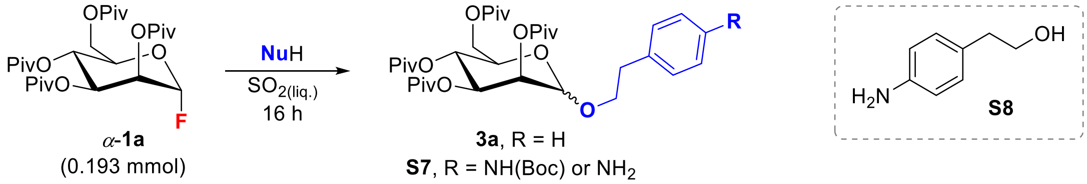

entry	NuH (equiv)		*T* (°C)	yield (%)*	S6:S8 ratio*
	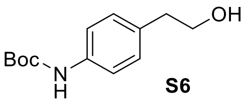 **S6**	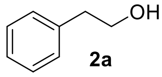 **2a**			
**1**	1.1	-	100	**S7**, ND	88:12
**2**	1.1	-	150	**S7**, ND	51:49
**3**	1.0	1.0	100	**S7**, ND<br>**3a**, ND	68:32

*Determined by ^{1}H NMR analysis of a crude reaction mixture. ND = not detected

5. Reaction optimization data

Table S3: Optimization for pivaloyl protected glucosyl fluoride β-**9** as a glycosyl donor in liquid SO_2.

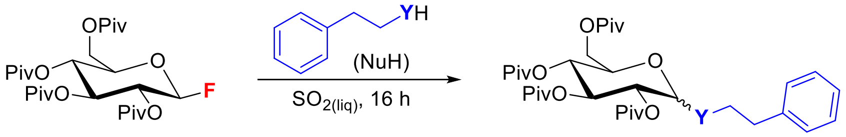

β-**9**
(0.096 mmol)

10a, Y = O
10b, Y = S

entry	NuH		*T* (°C)	α:β ratio[a]	yield (%)[b]
	Y	(equiv)			
**1**	O	1.1	30	10:90	**10a**, 24
**2**		1.1	60	15:85	**10a**, 33
**3**		1.1	100	22:78	**10a**, 41
**4**		2.0	100	17:83	**10a**, 35
**5**		3.0	100	15:85	**10a**, 61
**6**	S	1.1	30	11:89	**10b**, 29
**7**		1.1	60	18:82	**10b**, 39
**8**		1.1	100	18:82	**10b**, 31
**9**		3.0	100	24:76	**10b**, 57

[a]Determined by ^{1}H NMR analysis of a crude reaction mixture. [b]Yield of isolated product.

Table S4: Optimization for acetyl protected manno- and glucopyranosyl fluorides α-**11** and α-**12** as glycosyl donors in liquid SO_2.

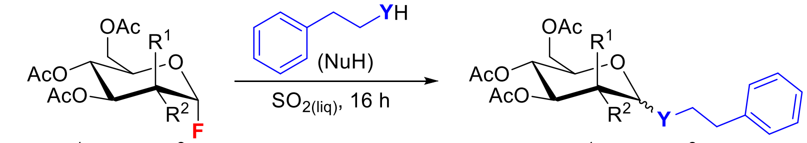

α-**11**, R^1 = OAc, R^2 = H
α-**12**, R^1 = H, R^2 = OAc

13, R^1 = OAc, R^2 = H
14, R^1 = H, R^2 = OAc

entry	glycosyl fluoride	NuH		*T* (°C)	α:β ratio[a]	yield (%)[b]
		Y	(equiv)			
**1**	α-**11**	O	3.0	30	NR	
**2**		O	3.0	60	88:12	**13a**, 41
**3**		O	3.0	100	91:9	**13a**, 55
**4**		S	3.0	100	78:22	**13b**, 67
**5**	α-**12**	O	2.0	30	NR	
**6**		O	2.0	60	30:70	**14a**, 37
**7**		O	2.0	100	66:34	**14a**, 42
**8**		O	3.0	100	54:46	**14a**, 43
**9**		S	3.0	100	48:52	**14b**, 76

[a]Reactions were carried out in a scale of 0.208 to 0.300 mmol (α-**11** or α-**12**). [b]Determined by ^{1}H NMR analysis of a crude reaction mixture. [c]Yield of isolated product. NR = no reaction

Table S5: Optimization for benzyl protected mannosyl fluoride *α*-**15** as a glycosyl donor in liquid SO_2.

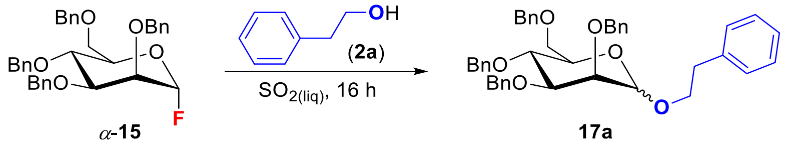

α-**15** (0.111 to 0.184 mmol) **17a**

entry	2a (equiv)	*T* (°C)	α:β ratio[a]	yield 17a (%)[b]
**1**		−10	71:29	31
**2**	1.2	30	98:2	68
**3**		60	not observed	
**4**		100	not observed	
**5**	2.0	30	98:2	72

[a]Determined by HPLC analysis of a crude reaction mixture. [b]Yield of isolated product.

Table S6: Optimization for benzyl protected glucosyl fluoride **16** as a glycosyl donor in liquid SO_2.

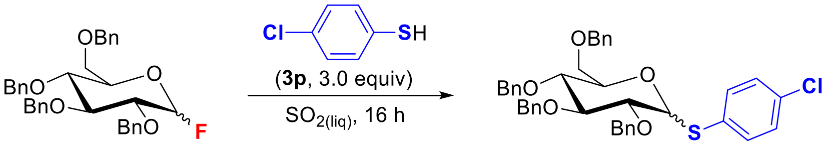

16 (0.092 to 0.184 mmol) **18f**

entry	α:β ratio[a] (16)	T (°C)	α:β ratio[a] (18f)	yield 18f (%)[b]
**1**	20:80	30	73:27	56
**2**	26:74	60	73:27	66
**3**	26:74	100	70:30	49
**4**		-10	74:26	45
**5**	>99:1	30	73:27	60
**6**		60	73:27	47
**7**		100	72:28	19

[a]Determined by HPLC analysis of a crude reaction mixture. [b]Yield of isolated product.

6. Reactivity of other glycosyl donors in liquid SO_2

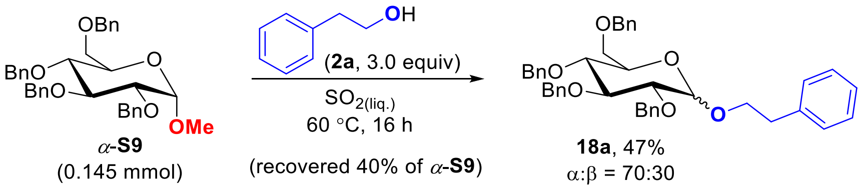

Scheme S1: Benzyl protected 1-*O*-methyl glucoside α-**S9** as a glycosyl donor in liquid SO_2.

Table S7: Glycosylation of 2-phenylethanol (**2a**, 3 equiv) with peracylated manno- and glucopyranoses **S10–13** in liquid SO_2 (16 h).[a]

entry	glycosyl donor		T (°C)	product	yield (%)[b]	recovered glycosyl donor (%)[b]
1	**S10**	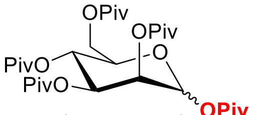 (α:β = 37:63)	100	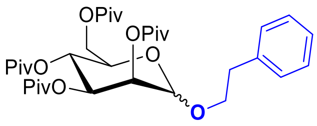	NR	
2			150		**3a**, 2[c]	**S10**, 88[c]
3	*β*-**S11**	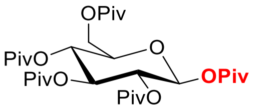	100	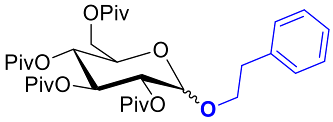	**10a**, 9	*β*-**S11**, 40
4			150		**10a**, 25	*β*-**S11**, ND
5	**S12**	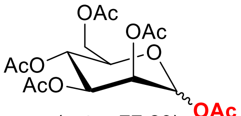 (α:β = 77:23)	100	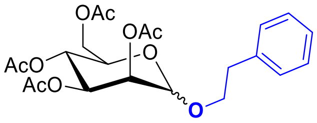	**13a**, 9	**S12**, 40
6			150		**13a**, 19	**S12**, 12
7	*β*-**S13**	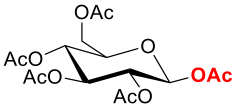	100	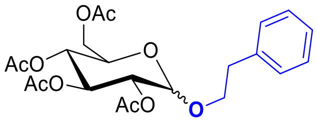	**14a**, 13	*β*-**S13**, ND
8			60		**14a**, 5	*β*-**S13**, 14

[a]Reactions were carried out in a scale of 74 to 100 mg (**S10–S13**). [b]Yield of isolated/recovered compound. [c]Determined by ^{1}H NMR analysis of a crude reaction mixture (mol%); remaining 10 mol% correspond to the product of hydrolysis. NR = reaction; ND = not detected

7. Proposed structures of side-products

*from pivaloyl protected mannosyl fluoride α-**1a**:*

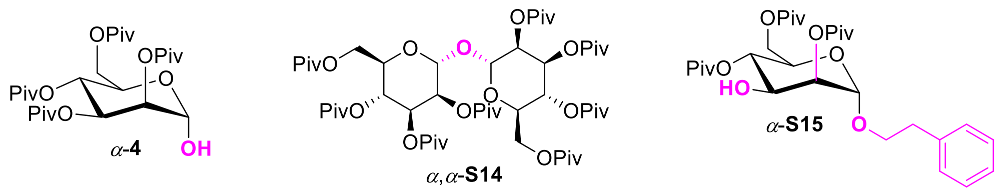

*from pivaloyl protected glucosyl fluoride β-**9** & penta-pivaloyl glucose β-**S11**:*

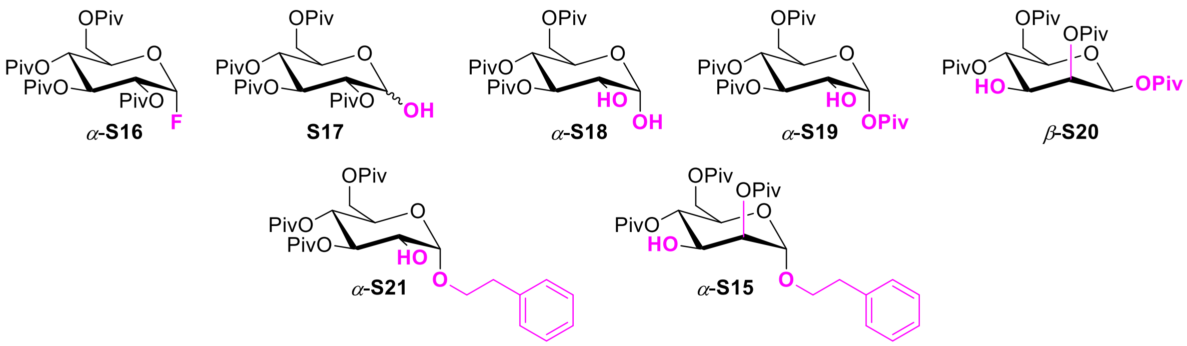

*from acetyl protected mannosyl fluoride α-**11** & penta-acetyl mannose **S12**:*

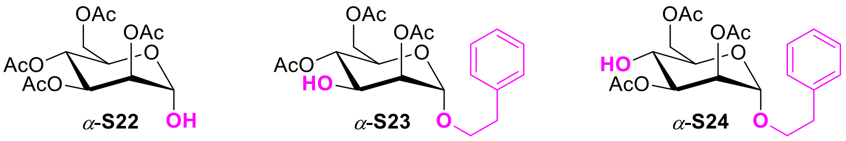

*from acetyl protected glucosyl fluoride α-**12** & penta-acetyl glucose β-**S13**:*

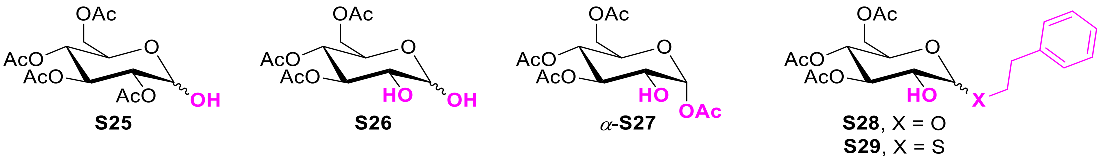

Side-products in disaccharide α-17f synthesis

*from benzyl protected mannosyl fluoride α-**15**:*

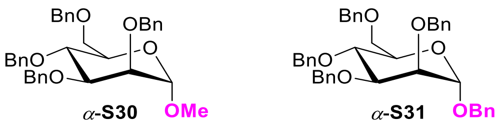

*from glycosyl acceptor α-**5**:*

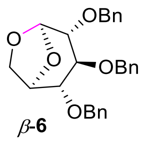

*from benzyl protected glucosyl fluoride **16**:*

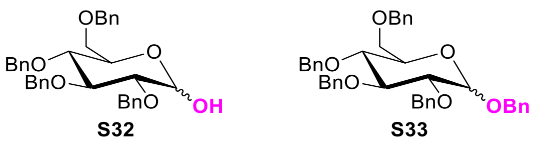

*from 2-deoxy glucosyl fluoride α-**19**:*

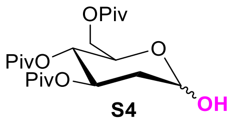

8. ^{19}F NMR studies

In order to detect FSO_2^- species, ^{19}F NMR spectroscopy was employed. Tetrabutyl ammonium fluorosulfite (FSO_2NBu_4) formed in the reaction between TBAF and SO_2 was selected as a standard to identify characteristic chemical shift[16] of fluorine in FSO_2^- anion associated with an ammonium type counter cation. ^{19}F NMR spectra were recorded by employing 0.9M TFA in D_2O as an external standard (–74.58 ppm referenced to the pure TFA as a reference standard (–76.55 ppm vs $CFCl_3$)).

Preparation of standard FSO_2NBu_4. TBAF (~1 mL, 1M sol. in THF) was placed into a glass tube equipped with a magnetic stirring bar. Solution was concentrated under reduced pressure by employing Schlenk line (~1 h). The glass tube was placed into a stainless steel reactor. The reactor was sealed and cooled in a dry ice-ethanol bath to –78 °C. Sulfur dioxide was then transferred from storage cylinder to the reaction vessel by distillation. The reactor was sealed and warmed to room temperature. The reaction was left stirring at 60 °C for 3 h. The reactor was cooled to room temperature (ice bath) and connected to a trap containing aqueous $NaHCO_3$ solution for SO_2 removal. The residue was dissolved in D_2O (2 mL) and analyzed by ^{19}F NMR (Figure S2): (a) full conversion of TBAF (–122.6 ppm) was observed; (b) characteristic chemical shift of FSO_2^- was observed at +39.1 ppm.

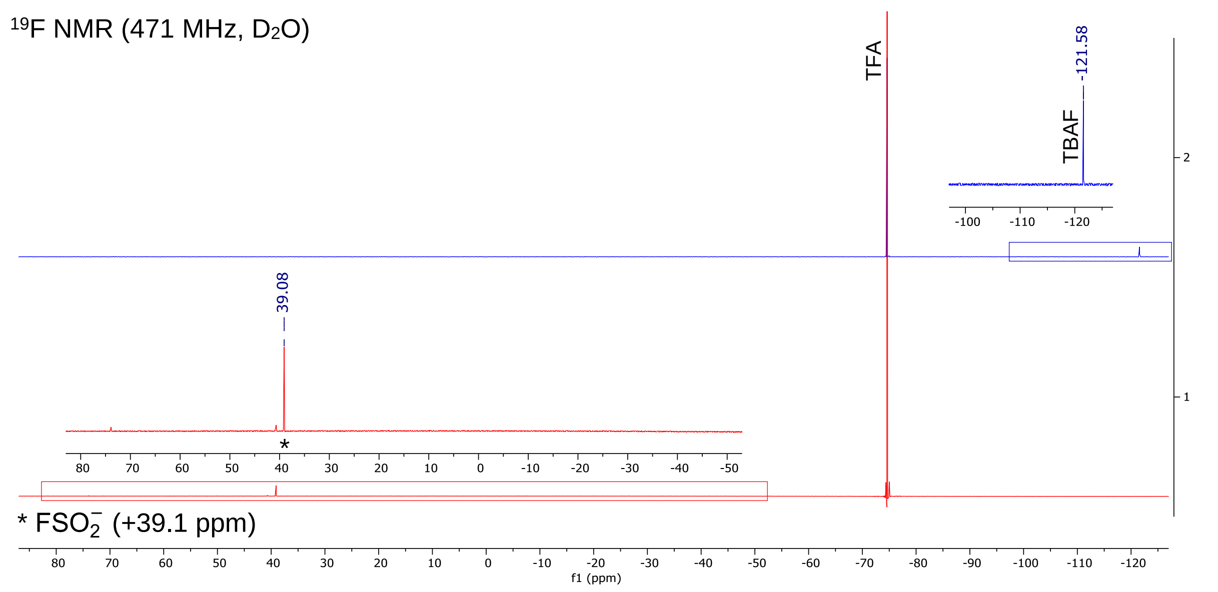

Figure S2: Generation of FSO_2^- species from TBAF and SO_2. (1 - TBAF + SO_2 → FSO_2NBu_4; 2 - TBAF standard)

Glycosylation reaction.

(1) Mannosyl fluoride α-**1a** (300 mg, 0.579 mmol, 1.0 equiv) and 2-phenylethanol (**2a**, 1.2 equiv) were placed into a glass pressure tube equipped with a magnetic stirring bar. 2M SO_2 solution in DCM (3 mL) was added and the glass tube was sealed with a PTFE screw cap. The reaction was left stirring at 100 °C for 6 h. Then, the reaction was allowed to cool to room temperature and Et_3N was added (2.0 equiv). After stirring for 5 min the reaction mixture was transferred to a flask and concentrated under reduced pressure. D_2O (1 mL) was added to the residue and water soluble components were analyzed by ^{19}F NMR (Figure S3, *spectrum 1*).

(2) Glucosyl fluoride (α-**16**, 300 mg, 0.553 mmol, 1.0 equiv) and 1-dodecanethiol (**2m**, 1.2 equiv) were placed into a glass pressure tube equipped with a magnetic stirring bar. 2M SO_2 solution in DCM (3 mL) was added and the glass tube was sealed with a PTFE screw cap. The reaction was left stirring at 30 °C for 6 h. Then, Et_3N was added (2.0 equiv). After stirring for 5 min the reaction mixture was transferred to a flask and concentrated under reduced pressure. D_2O (1 mL) was added to the residue and water soluble components were analyzed by ^{19}F NMR (Figure S3, *spectrum 2*).

Note: reaction was quenched by Et_3N in order to neutralize acidic media and stabilize possibly formed fluorosulfite anion in a form of ammonium salt FSO_2NHEt_3.

In both cases signal at +38 ppm was observed indicating release of FSO_2^- species during the glycosylation reaction in the presence of SO_2.

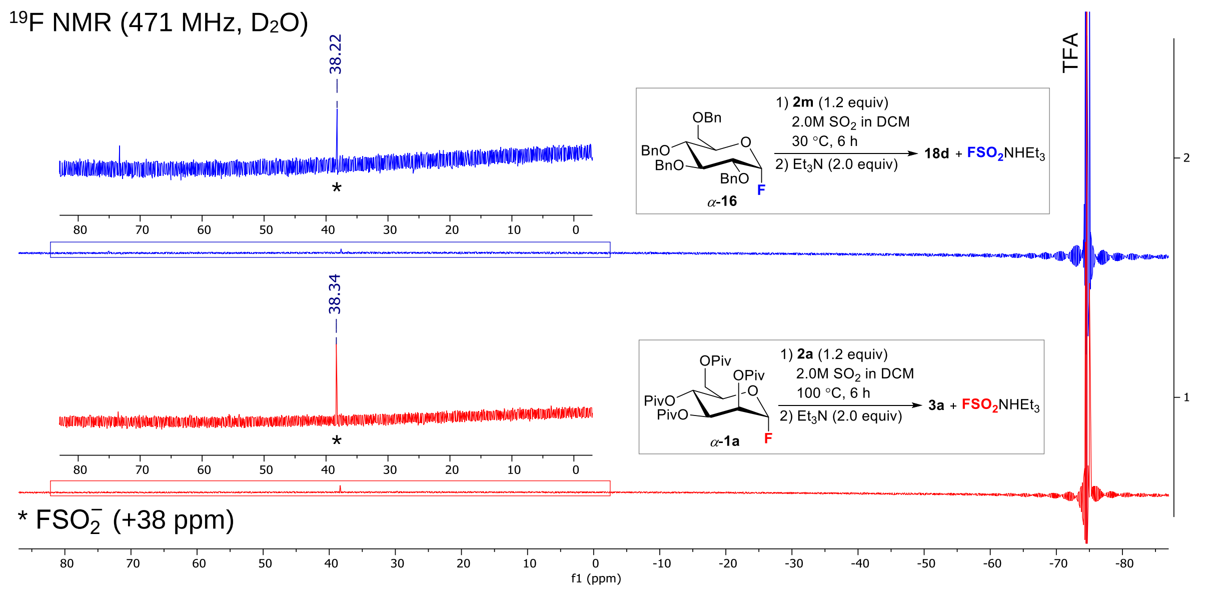

Figure S3: ^{19}F NMR analysis for detection of FSO_2^- species released during the glycosylation in 2M SO_2 solution in DCM.

9. Stability of various glycosyl donors in liquid SO_2

Table S8: Stability of various glycosyl donors in liquid SO_2 (16 h).[a]

entry		glycosyl donor		T (°C)	recovery of glycosyl donor (%)[b]
1	D-MANNOSE	α-**1a**	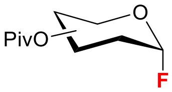	100	>95
2	D-MANNOSE	α-**1b**	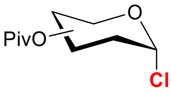	100	48
3	D-MANNOSE	α-**1c**	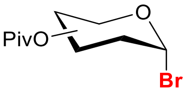	100	9
4	D-MANNOSE	α-**11**	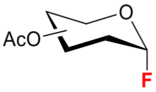	100	not recovered[c]
5	D-MANNOSE	α-**15**	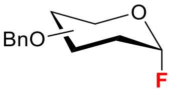	30	not recovered
6	D-MANNOSE	**S10**	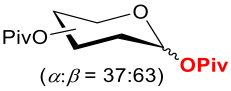 (α:β = 37:63)	100	>95
7	D-MANNOSE	**S12**	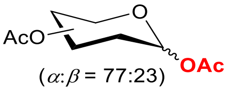 (α:β = 77:23)	100	>95
8	D-GLUCOSE	β-**9**	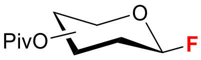	100	not recovered
9	D-GLUCOSE	α-**12**	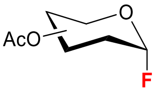	100	not recovered
10	D-GLUCOSE	α-**16**	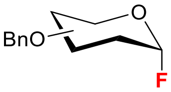	30	not recovered
11	D-GLUCOSE	β-**S11**	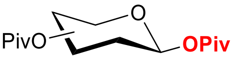	100	>95
12	D-GLUCOSE	β-**S13**	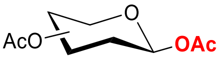	100	>95
13	D-GLUCOSE	α-**S9**	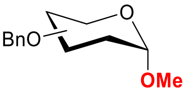	100	>95

[a]Experiments were carried out in a scale of 50 to 100 mg of glycosyl donor. [b]Determined by ^1H NMR analysis of a crude reaction mixture; 1,2,3-trimethoxybenzene as an internal standard. [c]Product of hydrolysis α-**S22** formed in 63% NMR yield.

10. Experimental data for synthesized compounds

Glycosyl donors:

2,3,4,6-Tetra-O-pivaloyl-α-D-mannopyranosyl fluoride (α-1a)[1]

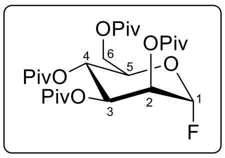

^{1}H NMR (500 MHz, $CDCl_3$): δ 5.55 (t, J = 10.0 Hz, 1H, H-C4), 5.52 (dd, J = 48.8, 1.6 Hz, 1H, H-C1), 5.42–5.36 (m, 2H, H-C2, H-C3), 4.25–4.15 (m, 3H, H-C5, $H_{a,b}$-C6), 1.27 (s, 9H, Piv), 1.24 (s, 9H, Piv), 1.16 (s, 9H, Piv), 1.12 (s, 9H, Piv); ^{13}C NMR (126 MHz, $CDCl_3$): δ 178.13, 177.21, 176.95, 176.66, 105.15 (d, J = 224 Hz), 71.31 (d, J = 3 Hz), 68.67 (d, J = 2 Hz), 67.73 (d, J = 40 Hz), 64.24, 61.38, 39.06, 39.05, 38.96, 38.92, 27.22 (3C), 27.18.

2,3,4,6-Tetra-O-pivaloyl-α-D-mannopyranosyl chloride (α-1b)

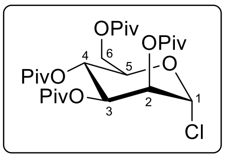

White amorphous solid; R_f = 0.51 (EtOAc/hexanes 1:9); $[\alpha]_D^{24}$ +70.1 (c 1.19, $CHCl_3$); ^{1}H NMR (500 MHz, $CDCl_3$): δ 5.94 (d, J = 1.7 Hz, 1H, H-C1), 5.65 (dd, J = 10.2, 3.3 Hz, 1H, H-C3), 5.55 (t, J = 10.2 Hz, 1H, H-C4), 5.40 (dd, J = 3.3, 1.7 Hz, 1H, H-C2), 4.32 (ddd, J = 10.2, 3.8, 1.6 Hz, 1H, H-C5), 4.24 (dd, J = 12.6, 3.8 Hz, 1H, H_a-C6), 4.16 (dd, J = 12.6, 1.6 Hz, 1H, H_b-C6), 1.27 (s, 9H, Piv), 1.23 (s, 9H, Piv), 1.17 (s, 9H, Piv), 1.12 (s, 9H, Piv); ^{13}C NMR (126 MHz, $CDCl_3$): δ 178.06, 177.22, 176.87, 176.67, 89.38, 71.76, 71.63, 68.25, 64.53, 61.24, 39.08, 39.03, 38.97, 38.90, 27.22, 27.21, 27.19, 27.18; HRMS (ESI) m/z calcd for $C_{26}H_{43}ClO_9Na$ $(M + Na)^+$ 557.2488, found 557.2459.

2,3,4,6-Tetra-O-pivaloyl-α-D-mannopyranosyl bromide (α-1c)[2]

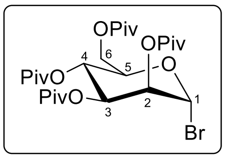

^{1}H NMR (500 MHz, $CDCl_3$): δ 6.25 (d, J = 1.6 Hz, 1H, H-C1), 5.74 (dd, J = 10.4, 3.2 Hz, 1H, H-C3), 5.57 (t, J = 10.4 Hz, 1H, H-C4), 5.45 (dd, J = 3.2, 1.6 Hz, 1H, H-C2), 4.30–4.21 (m, 2H, H-C5, H_a-C6), 4.18–4.12 (m, 1H, H_b-C6), 1.26 (s, 9H, Piv), 1.23 (s, 9H, Piv), 1.17 (s, 9H, Piv), 1.12 (s, 9H, Piv); ^{13}C NMR (126 MHz, $CDCl_3$): δ 178.03, 177.22, 176.78, 176.67, 83.88, 73.31, 72.18, 68.45, 64.48, 61.07, 39.08, 39.03, 38.97, 38.90, 27.21 (2C), 27.18 (2C).

2,3,4,6-Tetra-O-pivaloyl-β-D-glucopyranosyl fluoride (β-9)[3]

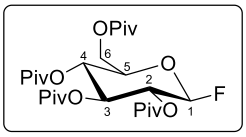

^{1}H NMR (500 MHz, $CDCl_3$): δ 5.32 (dd, J = 52.1, 6.5 Hz, 1H, H-C1), 5.29 (dd, J = 9.1, 8.2 Hz, 1H, H-C3), 5.25 (dd, J = 9.4, 9.1 Hz, 1H, H-C4), 5.12 (ddd, J = 11.5, 8.2, 6.5 Hz, 1H, H-C2), 4.25 (dd, J = 12.6, 1.8 Hz, 1H, H_a-C6), 4.13 (dd, J = 12.6, 4.8 Hz, 1H, H_b-C6), 3.87 (ddd, J = 9.4, 4.8, 1.8 Hz, 1H, H-C5), 1.23 (s, 9H, Piv), 1.18 (s, 9H, Piv), 1.15 (s, 9H, Piv), 1.12 (s, 9H, Piv); ^{13}C NMR (126 MHz, $CDCl_3$): δ 178.19, 177.26, 176.67, 176.41, 106.92 (d, J = 219 Hz), 72.59 (d, J = 5 Hz), 71.49 (d, J = 1 Hz), 71.42 (d, J = 39 Hz), 67.02, 61.48, 39.05, 38.95, 38.90, 38.87, 27.23 (2C), 27.15, 27.13.

2,3,4,6-Tetra-O-acetyl-α-D-mannopyranosyl fluoride (α-11)[5]

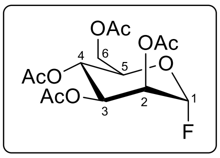

^{1}H NMR (500 MHz, $CDCl_3$): δ 5.58 (dd, J = 48.6, 1.6 Hz, 1H, H-C1), 5.43–5.39 (m, 1H, H-C2), 5.38–5.31 (m, 2H, H-C3, H-C4), 4.30 (dd, J = 12.5, 5.3 Hz, 1H, H_a-C6), 4.20–4.12 (m, 2H, H-C5, H_b-C6), 2.18 (s, 3H, Ac), 2.11 (s, 3H, Ac), 2.06 (s, 3H, Ac), 2.01 (s, 3H, Ac); ^{13}C NMR (126 MHz, $CDCl_3$): δ 170.72, 169.89, 169.82, 169.73, 104.89 (d, J = 221 Hz), 71.04 (d, J = 3 Hz), 68.32 (d, J = 2 Hz), 67.82 (d, J = 40 Hz), 65.16, 62.00, 20.85 (2C), 20.79, 20.72.

2,3,4,6-Tetra-O-acetyl-α-D-glucopyranosyl fluoride (α-12)[5]

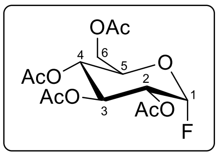

^{1}H NMR (500 MHz, $CDCl_3$): δ 5.75 (dd, J = 52.9, 2.8 Hz, 1H, H-C1), 5.49 (dd, J = 10.2, 9.9 Hz, 1H, H-C3), 5.15 (t, J = 9.9 Hz, 1H, H-C4), 4.96 (ddd, J = 24.2, 10.2, 2.8 Hz, 1H, H-C2), 4.29 (dd, J = 12.4, 4.1 Hz, 1H, H_a-C6), 4.19 (ddd, J = 9.9, 4.1, 2.2 Hz, 1H, H-C5), 4.19 (dd, J = 12.4, 2.2 Hz, 1H, H_b-C6), 2.10 (s, 3H, Ac), 2.10 (s, 3H, Ac), 2.04 (s, 3H, Ac), 2.03 (s, 3H, Ac); ^{13}C NMR (126 MHz, $CDCl_3$): δ 170.68, 170.12, 170.08, 169.57, 103.89 (d, J = 229 Hz), 70.35 (d, J = 25 Hz), 69.92 (d, J = 4 Hz), 69.54, 67.48, 61.34, 20.82, 20.75, 20.68 (2C).

2,3,4,6-Tetra-O-benzyl-α-D-mannopyranosyl fluoride (α-15)[5]

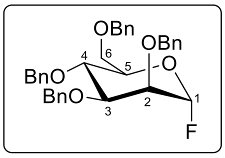

^{1}H NMR (500 MHz, $CDCl_3$): δ 7.39–7.24 (m, 18H, H-Ar), 7.20–7.14 (m, 2H, H-Ar), 5.60 (dd, J = 50.5, 1.6 Hz, 1H, H-C1), 4.87 (d, J = 10.9 Hz, 1H, H-CPh), 4.80 (d, J =12.2 Hz, 1H, H-CPh), 4.74–4.61 (m, 4H, 4×H-CPh), 4.57–4.50 (m, 2H, 2×H-CPh), 4.07 (t, J = 9.4 Hz, 1H, H-C4), 3.96–3.84 (m, 3H, H-C2, H-C3, H-C5), 3.78 (dd, J = 11.0, 4.6 Hz, 1H, H_a-C6), 3.72 (dd, J = 11.0, 1.9 Hz, 1H, H_b-C6); ^{13}C NMR (126 MHz, $CDCl_3$): δ 138.35, 138.31, 138.28, 137.98, 128.60 (2C), 128.50, 128.49, 128.11, 128.03, 128.01 (2C), 127.90, 127.85 (2C), 127.72, 106.60 (d, J = 223 Hz), 79.34 (d, J = 2 Hz), 75.29, 74.33 (d, J = 2 Hz), 74.19, 73.62 (d, J = 34 Hz), 73.57, 73.38, 72.73, 68.76.

2,3,4,6-Tetra-O-benzyl-α-D-glucopyranosyl fluoride (α-16)[5]

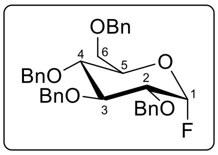

^{1}H NMR (500 MHz, $CDCl_3$): δ 7.40–7.24 (m, 18H, H-Ar), 7.18–7.13 (m, 2H, H-Ar), 5.56 (dd, J = 53.3, 2.7 Hz, 1H, H-C1), 4.96 (d, J = 10.8 Hz, 1H, H-CPh), 4.86 (d, J = 10.8 Hz, 1H, H-CPh), 4.85 (d, J = 10.8 Hz, 1H, H-CPh), 4.81 (d, J = 11.7 Hz, 1H, H-CPh), 4.71 (d, J = 11.7 Hz, 1H, H-CPh), 4.60 (d, J = 12.2 Hz, 1H, H-CPh), 4.52 (d, J = 10.8 Hz, 1H, H-CPh), 4.48 (d, J = 12.2 Hz, 1H, H-CPh), 3.99 (t, J = 9.6 Hz, 1H, H-C3), 3.95 (ddd, J = 9.6, 3.0, 2.0 Hz, 1H, H-C5), 3.76 (dd, J = 10.8, 3.0 Hz, 1H, H_a-C6), 3.74 (t, J = 9.6 Hz, 1H, H-C4), 3.67 (dd, J = 10.8, 2.0 Hz, 1H, H_b-C6), 3.57 (ddd, J = 25.5, 9.6, 2.7 Hz, 1H, H-C2); ^{13}C NMR (126 MHz, $CDCl_3$): δ 138.60, 138.15, 137.83, 137.81, 128.70, 128.58, 128.56, 128.54, 128.33, 128.20, 128.16, 128.09, 127.99, 127.94, 127.93, 127.86, 105.70 (d, J = 227 Hz), 81.58, 79.44 (d, J = 25 Hz), 76.77, 75.97, 75.31, 73.69, 73.66, 72.81 (d, J = 4 Hz), 67.95.

2,3,4,6-Tetra-O-benzyl-β-D-glucopyranosyl fluoride (β-16)[6]

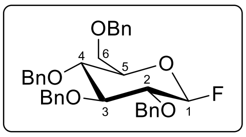

^{1}H NMR (500 MHz, $CDCl_3$): δ 5.27 (dd, J = 52.7, 6.7 Hz, 1H, H-C1), 3.78–3.70 (m, 3H, H-C4, $H_{a,b}$-C6), 3.67 (t, J = 8.7 Hz, 1H, H-C3), 3.64–3.56 (m, 2H, H-C5, H-C2); ^{13}C NMR (126 MHz, $CDCl_3$): δ 110.00 (d, J = 216 Hz), 83.59 (d, J = 12 Hz), 81.61 (d, J = 22 Hz), 77.04, 74.97 (d, J = 5 Hz), 68.51.

3,4,6-Tri-O-pivaloyl-2-deoxy-α-D-glucopyranosyl fluoride (α-19)

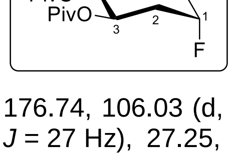

White amorphous solid; R_f = 0.47 (EtOAc/hexanes 3:17); $[\alpha]_D^{24}$ +54.3 (c 1.03, $CHCl_3$); ^{1}H NMR (500 MHz, $CDCl_3$): δ 5.74 (ddd, J = 51.6, 2.9, 1.3 Hz, 1H, H-C1), 5.29 (ddd, J = 11.4, 9.7, 5.2 Hz, 1H, H-C3), 5.15 (t, J = 9.7 Hz, 1H, H-C4), 4.24–4.10 (m, 3H, H-C5, $H_{a,b}$-C6), 2.48 (dddd, J = 13.7, 5.2, 4.9, 1.3 Hz, 1H, H_{eq}-C2), 1.80 (dddd, J = 38.9, 13.7, 11.4, 2.9 Hz, 1H, H_{ax}-C2), 1.23 (s, 9H, Piv), 1.17 (s, 9H, Piv), 1.15 (s, 9H, Piv); ^{13}C NMR (126 MHz, $CDCl_3$): δ 178.20, 177.55, 176.74, 106.03 (d, J = 221 Hz), 70.79 (d, J =3 Hz), 67.95, 67.65, 61.74, 39.04, 38.94, 38.85, 34.82 (d, J = 27 Hz), 27.25, 27.20 (2C); HRMS (ESI) m/z calcd for $C_{21}H_{35}FO_7Na$ $(M + Na)^+$ 441.2259, found 441.2260.

3,4,6-Tri-O-pivaloyl-2-deoxy-β-D-glucopyranosyl fluoride (β-19)

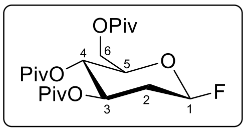

White amorphous solid; R_f = 0.35 (EtOAc/hexanes 3:17); ^{1}H NMR (500 MHz, $CDCl_3$): δ 5.55 (ddd, J = 52.1, 6.1, 2.7 Hz, 1H, H-C1), 5.13 (t, J = 7.6 Hz, 1H, H-C4), 5.00 (td, J = 7.6, 5.2 Hz, 1H, H-C3), 4.30 (dd, J = 12.2, 4.7 Hz, 1H, H_a-C6), 4.27 (dd, J = 12.2, 5.3 Hz, 1H, H_b-C6), 3.89 (ddd, J = 7.6, 5.3, 4.7 Hz, 1H, H-C5), 2.39 (dddd, J = 17.8, 14.2, 5.2, 2.7 Hz, 1H, H_{eq}-C2), 1.96 (dddd, J = 14.2, 9.8, 7.6, 6.1 Hz, 1H, H_{ax}-C2), 1.23 (s, 9H, Piv), 1.18 (s, 9H, Piv), 1.18 (s, 9H, Piv); ^{13}C NMR (126 MHz, $CDCl_3$): δ 178.21, 177.60, 176.71, 105.72 (d, J = 216 Hz), 72.44 (d, J = 3 Hz), 67.60 (d, J = 8 Hz), 66.88, 62.54, 39.00, 38.93, 38.85, 33.41 (d, J = 24 Hz), 27.26, 27.18, 27.14. ***Note:*** *compound degraded during the storage at –4 °C.*

1-O-Methyl-2,3,4,6-tetra-O-benzyl-α-D-glucopyranose (α-S9)[7]

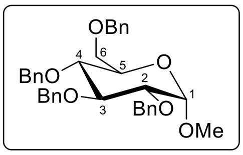

^{1}H NMR (500 MHz, $CDCl_3$): δ 7.38–7.24 (m, 18H, H-Ar), 7.16–7.11 (m, 2H, H-Ar), 4.98 (d, J = 10.7 Hz, 1H, H-CPh), 4.85–4.78 (m, 3H, 3×H-CPh), 4.67 (d, J = 12.4 Hz, 1H, H-CPh), 4.63 (d, J = 3.6 Hz, 1H, H-C1), 4.61 (d, J = 12.2 Hz, 1H, H-CPh), 4.51–4.45 (m, 2H, 2×H-CPh), 3.99 (t, J = 9.5 Hz, 1H, H-C3), 3.77–3.70 (m, 2H, H-C5, H_a-C6), 3.66–3.60 (m, 2H, H-C4, H_b-C6), 3.56 (dd, J = 9.5, 3.6 Hz, 1H, H-C2), 3.38 (s, 3H, OMe); ^{13}C NMR (126 MHz, $CDCl_3$): δ 138.95, 138.41, 138.32, 138.07, 128.59, 128.53, 128.50 (2C), 128.28, 128.11, 128.04 (2C), 127.99, 127.82, 127.80, 127.72, 98.36, 82.28, 79.98, 77.81, 75.90, 75.17, 73.63, 73.54, 70.19, 68.63, 55.31.

1,2,3,4,6-Penta-O-pivaloyl-D-mannopyranose (S10)[2]

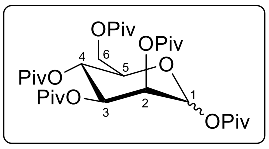

α:β = 37:63 (NMR, isolated); ^{1}H NMR (500 MHz, $CDCl_3$): δ (α) 6.01 (d, J = 1.8 Hz, 1H, H-C1), 5.53 (t, J = 10.2 Hz, 1H, H-C4), 5.34 (dd, J = 10.2, 3.1 Hz, 1H, H-C3), 5.28 (dd, J = 3.1, 1.8 Hz, 1H, H-C2), 4.24–4.09 (m, 2H, $H_{a,b}$-C6), 4.06–4.00 (m, 1H, H-C5); (β) 5.83 (br s, 1H, H-C1), 5.51–5.44 (m, 2H, H-C2, H-C4), 5.16 (dd, J = 10.2, 3.1 Hz, 1H, H-C3), 4.24–4.09 (m, 2H, $H_{a,b}$-C6), 3.88–3.80 (m, 1H, H-C5); ^{13}C NMR (126 MHz, $CDCl_3$): δ (α) 90.87, 71.32, 69.51, 68.35, 64.65, 61.84; (β) 90.84, 73.32, 71.14, 68.29, 64.85, 61.65.

1,2,3,4,6-Penta-O-pivaloyl-β-D-glucopyranose (β-S11)[17]

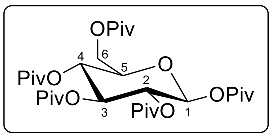

^{1}H NMR (500 MHz, $CDCl_3$): δ 5.70 (d, J = 8.3 Hz, 1H, H-C1), 5.37 (dd, J = 9.8, 9.3 Hz, 1H, H-C3), 5.21 (dd, J = 9.3, 8.3 Hz, 1H, H-C2), 5.16 (t, J = 9.8 Hz, 1H, H-C4), 4.15 (dd, J = 12.4, 2.2 Hz, 1H, H_a-C6), 4.10 (dd, J = 12.4, 5.3 Hz, 1H, H_b-C6), 3.86 (ddd, J = 9.8, 5.3, 2.2 Hz, 1H, H-C5), 1.21 (s, 9H, Piv), 1.17 (s, 9H, Piv), 1.15 (s, 9H, Piv), 1.12 (br s, 18H, 2×Piv); ^{13}C NMR (126 MHz, $CDCl_3$): δ 178.18, 177.20, 176.58 (2C), 176.50, 92.01, 72.94, 72.50, 70.25, 67.89, 61.61, 39.01, 38.91, 38.86 (3C), 27.29, 27.23, 27.20, 27.18, 26.98.

1,2,3,4,6-Penta-O-acetyl-D-mannopyranose (S12)[9]

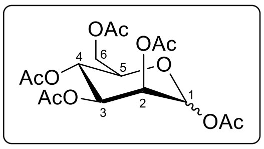

α:β = 77:23 (NMR, isolated); ^{1}H NMR (500 MHz, $CDCl_3$): δ (α) 6.08 (d, J = 1.8 Hz, 1H, H-C1), 5.38–5.32 (m, 2H, H-C3, H-C4), 5.28–5.24 (m, 1H, H-C2), 4.28 (dd, J = 12.4, 5.2 Hz, 1H, H_a-C6), 4.10 (dd, J = 12.4, 2.6 Hz, 1H, H_b-C6), 4.08–4.01 (m, 1H, H-C5); (β) 5.86 (d, J = 1.2 Hz, 1H, H-C1), 5.48 (dd, J = 3.4, 1.2 Hz, 1H, H-C2), 5.13 (dd, J = 10.0, 3.4 Hz, 1H, H-C3), 5.30–5.28 (m, 1H, H-C4), 4.30 (dd, J = 12.4, 5.2 Hz, 1H, H_a-C6), 4.14 (dd, J = 12.4, 2.4 Hz, 1H, H_b-C6), 3.80 (ddd, J = 9.9, 5.2, 2.4 Hz, 1H, H-C5); ^{13}C NMR (126 MHz, $CDCl_3$): δ (α) 90.74, 70.75, 68.87, 68.47, 65.68, 62.24; (β) 90.56, 73.45, 70.79, 68.31, 65.54, 62.20.

1,2,3,4,6-Penta-O-acetyl-β-D-glucopyranose (β-S13)[18]

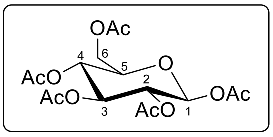

^{1}H NMR (500 MHz, $CDCl_3$): δ 5.71 (d, J = 8.4 Hz, 1H, H-C1), 5.25 (t, J = 9.5 Hz, 1H, H-C3), 5.13 (dd, J = 9.5, 8.4 Hz, 1H, H-C2), 5.13 (dd, J = 9.9, 9.5 Hz, 1H, H-C4), 4.29 (dd, J = 12.5, 4.6 Hz, 1H, H_a-C6), 4.11 (dd, J = 12.5, 2.2 Hz, 1H, H_b-C6), 3.84 (ddd, J = 9.9, 4.6, 2.2 Hz, 1H, H-C5), 2.11 (s, 3H, Ac), 2.08 (s, 3H, Ac), 2.03 (br s, 6H, 2×Ac), 2.01 (s, 3H, Ac); ^{13}C NMR (126 MHz, $CDCl_3$): δ 170.73, 170.23, 169.51, 169.37, 169.09, 91.83, 72.92, 72.86, 70.35, 67.87, 61.57, 20.95, 20.84, 20.70 (2C), 20.69.

Intermediates isolated in the synthesis of 2-deoxy glucosyl fluoride α-19:

3,4,6-Tri-O-acetyl-D-glucal (S1)[12]

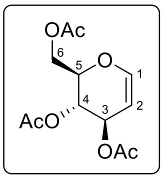

^{1}H NMR (500 MHz, $CDCl_3$): δ 6.46 (d, J = 6.2 Hz, 1H, H-C1), 5.37–5.30 (m, 1H, H-C3), 5.21 (dd, J = 7.4, 6.0 Hz, 1H, H-C4), 4.84 (dd, J = 6.2, 3.2 Hz, 1H, H-C2), 4.39 (dd, J = 12.2, 5.9 Hz, 1H, H_a-C6), 4.28–4.22 (m, 1H, H-C5), 4.19 (dd, J = 12.2, 2.9 Hz, 1H, H_b-C6), 2.08 (s, 3H, Ac), 2.07 (s, 3H, Ac), 2.04 (s, 3H, Ac); ^{13}C NMR (126 MHz, $CDCl_3$): δ 170.73, 170.55, 169.71, 145.76, 99.13, 74.08, 67.56, 67.31, 61.50, 21.13, 20.93, 20.86.

3,4,6-Tri-O-pivaloyl-D-glucal (S2)[13]

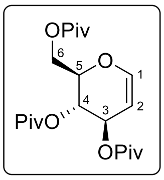

^{1}H NMR (500 MHz, $CDCl_3$): δ 6.45 (dd, J = 6.2, 1.3 Hz, 1H, H-C1), 5.34–5.29 (m, 1H, H-C3), 5.26 (dd, J = 7.3, 5.9 Hz, 1H, H-C4), 4.81 (dd, J = 6.2, 3.2 Hz, 1H, H-C2), 4.32 (dd, J = 11.9, 5.6 Hz, 1H, H_a-C6), 4.29–4.25 (m, 1H, H-C5), 4.21 (dd, J = 11.9, 2.8 Hz, 1H, H_b-C6), 1.22 (s, 9H, Piv), 1.18 (s, 9H, Piv), 1.18 (s, 9H, Piv); ^{13}C NMR (126 MHz, $CDCl_3$): δ 178.29, 177.95, 176.73, 145.77, 99.22, 74.30, 67.66, 66.80, 61.48, 39.01, 38.89, 38.87, 27.26, 27.20, 27.14.

1-O-Acetyl-3,4,6-tri-O-pivaloyl-2-deoxy-D-glucopyranose (S3)

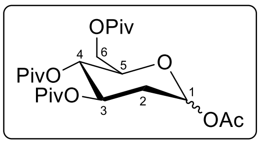

Yellowish amorphous solid; α:β = 81:19 (NMR, isolated); R_f = 0.38 (EtOAc/hexanes 1:4); ^{1}H NMR (500 MHz, $CDCl_3$): δ(α) 6.24 (dd, J = 3.7, 1.5 Hz, 1H, H-C1), 5.28 (ddd, J = 11.5, 9.8, 5.2 Hz, 1H, H-C3), 5.12 (t, J = 9.8 Hz, 1H, H-C4), 4.14 (dd, J = 12.2, 2.0 Hz, 1H, H_a-C6), 4.11 (dd, J = 12.2, 4.5 Hz, 1H, H_b-C6), 4.06 (ddd, J = 9.8, 4.5, 2.0 Hz, 1H, H-C5), 2.26 (ddd, J = 13.6, 5.2, 1.5 Hz, 1H, H_{eq}-C2), 2.12 (s, 3H, Ac), 1.90 (ddd, J = 13.6, 11.5, 3.7 Hz, 1H, H_{ax}-C2), 1.20 (s, 9H, Piv), 1.17 (s, 9H, Piv), 1.14 (s, 9H, Piv); (β) 5.80 (dd, J = 9.8, 2.4 Hz, 1H, H-C1), 5.08–5.01 (m, 2H, H-C3, H-C4), 4.17–4.08 (m, 2H, $H_{a,b}$-C6), 3.80–3.75 (m, 1H, H-C5), 2.36–2.30 (m, 1H, H_{eq}-C2), 1.86–1.77 (m, 1H, H_{ax}-C2); ^{13}C NMR (126 MHz, $CDCl_3$): δ(α) 178.20, 177.78, 176.77, 169.14, 91.04, 70.82, 68.46, 68.18, 61.99, 38.99, 38.94, 38.86, 34.15, 27.21 (2C), 27.19, 21.12; (β) 91.14, 73.29, 69.98, 67.88, 62.17, 34.76; HRMS (ESI) *m/z* calcd for $C_{23}H_{42}NO_9$ $(M + NH_4)^+$ 476.2854, found (α) 476.2834.

3,4,6-Tri-O-pivaloyl-2-deoxy-D-glucopyranose (S4)[13]

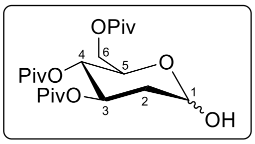

α:β = 77:23 (NMR, isolated); ^{1}H NMR (500 MHz, $CDCl_3$): δ (α) 5.41 (d, J = 3.3 Hz, 1H, H-C1), 5.35 (ddd, J = 11.4, 9.6, 5.3 Hz, 1H, H-C3), 5.09 (t, J = 9.6 Hz, 1H, H-C4), 4.24 (ddd, J = 9.6, 4.2, 1.7 Hz, 1H, H-C5), 4.18 (dd, J = 12.3, 1.7 Hz, 1H, H_a-C6), 4.10 (dd, J = 12.3, 4.2 Hz, 1H, H_b-C6), 2.26 (dd, J = 12.9, 5.3 Hz, 1H, H_{eq}-C2), 1.75 (ddd, J = 12.9, 11.4, 3.3 Hz, 1H, H_{ax}-C2); (β) 5.06 (t, J = 9.6 Hz, 1H, H-C4), 5.02 (td, J = 9.6, 4.9 Hz, 1H, H-C3), 4.95 (dd, J = 9.4, 2.1 Hz, 1H, H-C1), 4.22–4.15 (m, 1H, H_a-C6), 4.14–4.07 (m, 1H, H_b-C6), 3.68 (ddd, J = 9.6, 5.0, 1.8 Hz, 1H, H-C5), 2.39 (ddd, J = 12.4, 4.9, 2.1 Hz, 1H, H_{eq}-C2), 1.64 (ddd, J = 12.4, 9.6, 9.4 Hz, 1H, H_{ax}-C2).

Target glycosides:

1-O-(2-Phenylethyl)-2,3,4,6-tetra-O-pivaloyl-D-mannopyranose (3a)

Prepared according to the **method A** from mannosyl fluoride α-**1a** (200 mg, 0.386 mmol, 1.0 equiv) and 2-phenylethanol (**2a**, 1.1 equiv) at 100 °C; yield 87%; α:β = 97:3 (NMR).

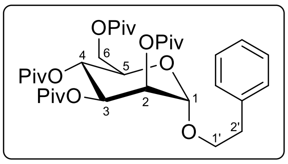

α-**3a**: white solid (206 mg); R_f = 0.62 (EtOAc/hexanes 1:4); $[\alpha]_D^{24}$ +60.1 (*c* 1.00, $CHCl_3$); ^{1}H NMR (300 MHz, $CDCl_3$): *δ* 7.37–7.17 (m, 5H, H-Ar), 5.37 (t, *J* = 10.1 Hz, 1H, H-C4), 5.27 (dd, *J* = 10.1, 3.2 Hz, 1H, H-C3), 5.21 (dd, *J* = 3.2, 1.7 Hz, 1H, H-C2), 4.73 (d, *J* = 1.7 Hz, 1H, H-C1), 3.92 (dd, *J* = 12.5, 3.8 Hz, 1H, H_a-C6), 3.83 (dd, *J* = 12.5, 2.0 Hz, 1H, H_b-C6), 3.84–3.69 (m, 2H, H_2-C1'), 3.19 (ddd, *J* = 10.1, 3.8, 2.0 Hz, 1H, H-C5), 3.01–2.82 (m, 2H, H_2-C2'), 1.24 (s, 9H, Piv), 1.20 (s, 9H, Piv), 1.14 (s, 9H, Piv), 1.12 (s, 9H, Piv); ^{13}C NMR (75.5 MHz, $CDCl_3$): *δ* 178.22, 177.36, 177.29, 176.69, 139.09, 129.29, 128.63, 126.55, 97.20, 69.66, 69.47, 68.56, 68.51, 65.02, 61.88, 39.02, 38.99, 38.89, 38.86, 36.13, 27.25 (3C), 27.18; HRMS (ESI) *m/z* calcd for $C_{34}H_{52}O_{10}Na$ (M + Na)$^+$ 643.3453, found 643.3464.

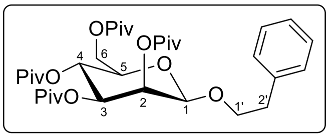

β-**3a**: isolated in 1% calculated yield (3.2 mg); R_f = 0.54 (EtOAc/hexanes 1:4); ^{1}H NMR (500 MHz, $CDCl_3$): *δ* 7.35–7.14 (m, 5H, H-Ar), 5.43 (d, *J* = 3.2 Hz, 1H, H-C2), 5.39 (t, *J* = 10.0 Hz, 1H, H-C4), 5.05 (dd, *J* = 10.0, 3.2 Hz, 1H, H-C3), 4.61 (br s, 1H, H-C1), 4.21 (d, *J* = 12.1 Hz, 1H, H_a-C6), 4.16 (dd, *J* = 12.1, 4.7 Hz, 1H, H_b-C6), 4.10–4.02 (m, 1H, H-C5), 3.71–3.63 (m, 2H, H_2-C1'), 2.91–2.82 (m, 2H, H_2-C2'), 1.24 (s, 9H, Piv), 1.23 (s, 9H, Piv), 1.15 (s, 9H, Piv), 1.11 (s, 9H, Piv); ^{13}C NMR (126 MHz, $CDCl_3$): *δ* 178.31, 177.43, 177.34, 176.78, 138.56, 129.09, 128.47, 126.44, 99.18, 72.69, 71.31, 70.66, 68.62, 65.43, 62.15, 39.11, 39.05, 38.94, 38.90, 36.31, 27.28, 27.26, 27.19 (2C); HRMS (ESI) *m/z* calcd for $C_{34}H_{52}O_{10}Na$ (M + Na)$^+$ 643.3453, found 643.3448.

1-O-Cyclohexyl-2,3,4,6-tetra-O-pivaloyl-α-D-mannopyranose (α-3b)

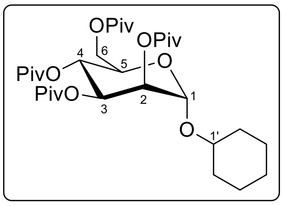

Prepared according to the **method A** from mannosyl fluoride *α*-**1a** (300 mg, 0.579 mmol, 1.0 equiv) and cyclohexanol (**2b**, 3.3 equiv) at 100 °C; yield 72% (250 mg); white solid; R_f = 0.56 (EtOAc/hexanes 1:9); $[\alpha]_D^{24}$ +34.3 (*c* 1.00, $CHCl_3$); ^{1}H NMR (300 MHz, $CDCl_3$): *δ* 5.46 (t, *J* = 10.1 Hz, 1H, H-C4), 5.40 (dd, *J* = 10.1, 2.9 Hz, 1H, H-C3), 5.18 (dd, *J* = 2.9, 1.9 Hz, 1H, H-C2), 4.91 (d, *J* = 1.9 Hz, 1H, H-C1), 4.17 (dd, *J* = 12.3, 4.8 Hz, 1H, H_a-C6), 4.16–4.06 (m, 2H, H-C5, H_b-C6), 3.66–3.53 (m, 1H, H-C1'), 1.98–1.81 (m, 2H), 1.81–1.63 (m, 2H), 1.61–1.19 (m, 6H), 1.26 (s, 9H, Piv), 1.23 (s, 9H, Piv), 1.16 (s, 9H, Piv), 1.11 (s, 9H, Piv); ^{13}C NMR (75.5 MHz, $CDCl_3$): *δ* 178.30, 177.40 (2C), 176.92, 96.04, 76.58, 70.36, 69.67, 69.09, 65.59, 62.46, 39.06, 39.02, 38.95, 38.90, 33.44, 31.54, 27.29 (2C), 27.23 (2C), 25.61, 24.22, 23.96; HRMS (ESI) *m/z* calcd for $C_{32}H_{54}O_{10}Na$ (M + Na)$^+$ 621.3609, found 621.3579.

1-(2-Phenylethylthio)-2,3,4,6-tetra-O-pivaloyl-D-mannopyranose (3c)

Prepared according to the **method A** from mannosyl fluoride *α*-**1a** (406 mg, 0.783 mmol, 1.0 equiv) and 2-phenylethanethiol (**2c**, 1.1 equiv) at 100 °C; yield 95%: α:β = 82:18 (NMR).

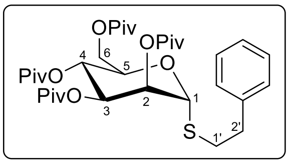

α-**3c**: white solid (396 mg); R_f = 0.39 (EtOAc/hexanes 1:9); $[\alpha]_D^{24}$ +75.9 (*c* 1.00, $CHCl_3$); ^{1}H NMR (300 MHz, $CDCl_3$): *δ* 7.36–7.14 (m, 5H, H-CAr), 5.49 (t, *J* = 10.0 Hz, 1H, H-C4), 5.33 (dd, *J* = 3.3, 1.6 Hz, 1H, H-C2), 5.27 (dd, *J* = 10.0, 3.3 Hz, 1H, H-C3), 5.15 (d, *J* = 1.6 Hz, 1H, H-C1), 4.33 (ddd, *J* = 10.0, 4.2, 1.8 Hz, 1H, H-C5), 4.18 (dd, *J* = 12.5, 4.2 Hz, 1H, H_a-C6), 4.05 (dd, *J* = 12.5, 1.8 Hz, H_b-C6), 3.00–2.76 (m, 4H, H_2-C(1',2')), 1.27 (s, 9H, Piv), 1.21 (s, 9H, Piv), 1.16 (s, 9H, Piv), 1.11 (s, 9H, Piv); ^{13}C NMR (75.5 MHz, $CDCl_3$): *δ* 178.23, 177.25, 177.09, 176.77, 139.88, 128.74, 128.67, 126.69, 82.99, 71.07, 69.95, 69.52, 65.38, 62.18, 39.05, 39.00, 38.93, 38.86, 36.18, 32.68, 27.27 (2C), 27.19 (2C); HRMS (ESI) *m/z* calcd for $C_{34}H_{52}O_9SK$ (M + K)$^+$ 675.2964, found 675.2993.

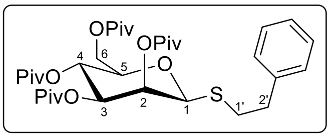

β-**3c**: colorless amorphous solid (78.8 mg); R_f = 0.55 (EtOAc/hexanes 1:4); $[\alpha]_D^{24}$ –38.3 (*c* 1.00, $CHCl_3$); ^1H NMR (300 MHz, $CDCl_3$): *δ* 7.37–7.14 (m, 5H, H-CAr), 5.46 (dd, *J* = 3.3, 1.0 Hz, 1H, H-C2), 5.38 (t, *J* = 10.1 Hz, 1H, H-C4), 5.02 (dd, *J* = 10.1, 3.3 Hz, 1H, H-C3), 4.59 (d, *J* = 1.0 Hz, 1H, H-C1), 4.22 (dd, *J* = 12.3, 1.8 Hz, 1H, H_a-C6), 4.12 (dd, *J* = 12.3, 4.9 Hz, 1H, H_b-C6), 3.62 (ddd, *J* = 10.1, 4.9, 1.8 Hz, 1H, H-C5), 3.03–2.80 (m, 4H, H_2-C(1',2')), 1.28 (s, 9H, Piv), 1.21 (s, 9H, Piv), 1.15 (s, 9H, Piv), 1.11 (s, 9H, Piv); ^{13}C NMR (75.5 MHz, $CDCl_3$): *δ* 178.23, 177.45, 177.08, 176.74, 140.10, 128.74, 128.68, 126.71, 83.25, 76.95, 72.31, 70.23, 65.03, 62.33, 39.21, 38.99, 38.93, 38.89, 36.77, 33.13, 27.33, 27.22, 27.21, 27.18; HRMS (ESI) *m/z* calcd for $C_{34}H_{52}O_9SNa$ $(M + Na)^+$ 659.3224, found 659.3240.

1-O-Propyl-2,3,4,6-tetra-O-pivaloyl-D-mannopyranose (3d)

Prepared according to the **method A** from mannosyl fluoride *α*-**1a** (300 mg, 0.579 mmol, 1.0 equiv) and *n*-propanol (**2d**, 1.1 equiv) at 100 °C; yield 89%; α:β = 97:3 (NMR).

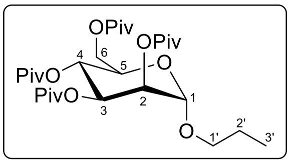

α-**3d**: white solid (275 mg); R_f = 0.63 (EtOAc/hexanes 1:4); $[\alpha]_D^{24}$ +31.4 (*c* 1.00, $CHCl_3$); ^1H NMR (300 MHz, $CDCl_3$): *δ* 5.46 (t, *J* = 10.0 Hz, 1H, H-C4), 5.38 (dd, *J* = 10.0, 3.1 Hz, 1H, H-C3), 5.24 (dd, *J* = 3.1, 1.7 Hz, 1H, H-C2), 4.76 (d, *J* = 1.7 Hz, 1H, H-C1), 4.19 (dd, *J* = 12.4, 4.4 Hz, 1H, H_a-C6), 4.13 (dd, *J* = 12.4, 2.2 Hz, 1H, H_b-C6), 4.03 (ddd, *J* = 10.0, 4.4, 2.2 Hz, 1H, H-C5), 3.65 (dt, *J* = 9.6, 7.0 Hz, 1H, H_a-C1'), 3.41 (dt, *J* = 9.6, 7.0 Hz, H_b-C1'), 1.63 (sextet, *J* = 7.0 Hz, 2H, H_2-C2'), 1.26 (s, 9H, Piv), 1.23 (s, 9H, Piv), 1.16 (s, 9H, Piv), 1.11 (s, 9H, Piv), 0.94 (t, *J* = 7.0 Hz, 3H, H_3-C3'); ^{13}C NMR (75.5 MHz, $CDCl_3$): *δ* 178.25, 177.34 (2C), 176.88, 97.86, 70.11, 69.79, 69.63, 69.02, 65.45, 62.29, 39.04, 39.02, 38.94, 38.89, 27.28 (2C), 27.23 (2C), 22.75, 10.69; HRMS (ESI) *m/z* calcd for $C_{29}H_{51}O_{10}$ $(M + H)^+$ 559.3477, found 559.3468.

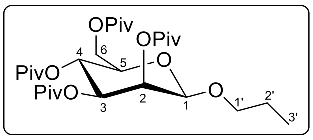

β-**3d**: white amorphous solid (12.3 mg); R_f = 0.48 (EtOAc/hexanes 1:4); $[\alpha]_D^{24}$ –19.1 (*c* 0.57, $CHCl_3$); ^1H NMR (500 MHz, $CDCl_3$): *δ* 5.43 (dd, *J* = 3.2, 1.1 Hz, 1H, H-C2), 5.39 (t, *J* = 10.0 Hz, 1H, H-C4), 5.09 (dd, *J* = 10.0, 3.2 Hz, 1H, H-C3), 4.63 (d, *J* = 1.1 Hz, 1H, H-C1), 4.23 (dd, *J* = 12.2, 1.8 Hz, 1H, H_a-C6), 4.15 (dd, *J* = 12.2, 4.9 Hz, 1H, H_b-C6), 3.75 (dt, *J* = 9.1, 6.9 Hz, 1H, H_a-C1'), 3.69 (ddd, *J* = 10.0, 4.9, 1.8 Hz, 1H, H-C5), 3.43 (dt, *J* = 9.1, 6.9 Hz, 1H, H_b-C1'), 1.56 (sextet, *J* = 6.9 Hz, 2H, H_2-C2'), 1.26 (s, 9H, Piv), 1.24 (s, 9H, Piv), 1.16 (s, 9H, Piv), 1.11 (s, 9H, Piv), 0.87 (t, *J* = 6.9 Hz, 3H, H_3-C3'); ^{13}C NMR (126 MHz, $CDCl_3$): *δ* 178.33, 177.48, 177.38, 176.82, 99.01, 72.64, 71.39, 71.35, 68.67, 65.55, 62.23, 39.12, 39.04, 38.95, 38.91, 27.29, 27.25, 27.20 (2C), 22.95, 10.50; HRMS (ESI) *m/z* calcd for $C_{29}H_{51}O_{10}$ $(M + H)^+$ 559.3477, found 559.3475.

1-O-Dodecyl-2,3,4,6-tetra-O-pivaloyl-D-mannopyranose (3e)

Prepared according to the **method A** from mannosyl fluoride *α*-**1a** (300 mg, 0.579 mmol, 1.0 equiv) and 1-dodecanol (**2e**, 1.0 equiv) at 100 °C; yield 90%; α:β = 93:7 (NMR).

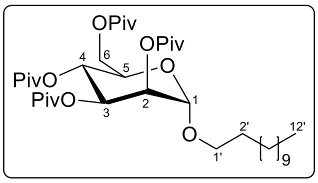

α-**3e**: colorless oil (333 mg); R_f = 0.55 (EtOAc/hexanes 1:9); $[\alpha]_D^{24}$ +29.0 (*c* 1.00, $CHCl_3$); ^1H NMR (300 MHz, $CDCl_3$): *δ* 5.46 (t, *J* = 10.0 Hz, 1H, H-C4), 5.38 (dd, *J* = 10.0, 3.0 Hz, 1H, H-C3), 5.23 (dd, *J* = 3.0, 1.8 Hz, 1H, H-C2), 4.75 (d, *J* = 1.8 Hz, 1H, H-C1), 4.19 (dd, *J* = 12.4, 4.3 Hz, 1H, H_a-C6), 4.13 (dd, *J* = 12.4, 1.9 Hz, 1H, H_b-C6), 4.02 (ddd, *J* = 10.0, 4.3, 1.9 Hz, 1H, H-C5), 3.68 (dt, *J* = 9.6, 6.8 Hz, 1H, H_a-C1'), 3.43 (dt, *J* = 9.6, 6.8 Hz, 1H, H_b-C1'), 1.60 (quint, *J* = 6.8 Hz, 2H, H_2-C2'), 1.40–1.25 (m, 18H), 1.26 (s, 9H, Piv), 1.24 (s, 9H, Piv), 1.16 (s, 9H, Piv), 1.12 (s, 9H, Piv), 0.88 (t, *J* = 6.8 Hz, 3H, H_3-C12'); ^{13}C NMR (75.5 MHz, $CDCl_3$): *δ* 178.24, 177.33, 177.31, 176.86, 97.86, 69.77, 69.63, 68.99, 68.58, 65.44, 62.26, 39.04, 39.02, 38.93, 38.88, 32.05, 29.80, 29.76 (2C), 29.71, 29.53, 29.49, 29.45, 27.28 (2C), 27.23, 27.22, 26.23, 22.82, 14.25; HRMS (ESI) *m/z* calcd for $C_{38}H_{68}O_{10}Na$ $(M + Na)^+$ 707.4705, found 707.4714.

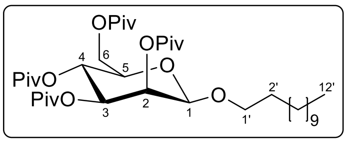

β-**3e**: colorless oil (25.0 mg); R_f = 0.33 (EtOAc/hexanes 1:9); $[\alpha]_D^{24}$ –26.9 (*c* 1.06, $CHCl_3$); ^{1}H NMR (300 MHz, $CDCl_3$): *δ* 5.42 (d, *J* = 3.3 Hz, 1H, H-C2), 5.38 (t, *J* = 10.0 Hz, 1H, H-C4), 5.08 (dd, *J* = 10.0, 3.3 Hz, 1H, H-C3), 4.62 (br s, 1H, H-C1), 4.23 (dd, *J* = 12.3, 1.9 Hz, 1H, H_a-C6), 4.15 (dd, *J* = 12.3, 4.8 Hz, 1H, H_b-C6), 3.78 (dt, *J* = 9.2, 6.5 Hz, 1H, H_a-C1'), 3.68 (ddd, *J* = 10.0, 4.8, 1.9 Hz, 1H, H-C5), 3.46 (dt, *J* = 9.2, 6.5 Hz, 1H, H_b-C1'), 1.53 (quint, *J* = 6.5 Hz, 2H, H_2-C2'), 1.40–1.20 (m, 18H), 1.26 (s, 9H, Piv), 1.23 (s, 9H, Piv), 1.15 (s, 9H, Piv), 1.11 (s, 9H, Piv), 0.88 (t, *J* = 6.5 Hz, 3H, H_3-C12'); ^{13}C NMR (75.5 MHz, $CDCl_3$): *δ* 178.32, 177.47, 177.35, 176.81, 98.98, 72.63, 71.35, 69.82, 68.67, 65.55, 62.23, 39.12, 39.03, 38.94, 38.90, 32.07, 29.81, 29.78, 29.73 (2C), 29.66, 29.49 (2C), 27.30, 27.26, 27.20 (2C), 26.00, 22.83, 14.27; HRMS (ESI) *m/z* calcd for $C_{38}H_{68}O_{10}Na$ $(M + Na)^+$ 707.4705, found 707.4689.

1-O-Propargyl-2,3,4,6-tetra-O-pivaloyl-D-mannopyranose (3f)

Prepared according to the **method A** from mannosyl fluoride *α*-**1a** (100 mg, 0.193 mmol, 1.0 equiv) and propargyl alcohol (**2f**, 3.0 equiv) at 100 °C; yield 91%; α:β = 94:6 (NMR).

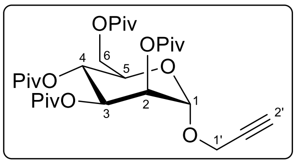

α-**3f**: white amorphous solid (91.6 mg); R_f = 0.56 (EtOAc/hexanes 1:4); $[\alpha]_D^{24}$ +49.1 (*c* 1.02, $CHCl_3$); ^{1}H NMR (500 MHz, $CDCl_3$): *δ* 5.50 (t, *J* = 10.2 Hz, 1H, H-C4), 5.39 (dd, *J* = 10.2, 3.3 Hz, 1H, H-C3), 5.27 (dd, *J* = 3.3, 1.5 Hz, 1H, H-C2), 4.96 (d, *J* = 1.5 Hz, 1H, H-C1), 4.31–4.25 (m, 2H, H_2-C1'), 4.20 (dd, *J* = 12.6, 4.5 Hz, 1H, H_a-C6), 4.13 (dd, *J* = 12.6, 1.7 Hz, 1H, H_b-C6), 4.08 (ddd, *J* = 10.2, 4.5, 1.7 Hz, 1H, H-C5), 2.52–2.43 (m, 1H, H-C2'), 1.27 (s, 9H, Piv), 1.24 (s, 9H, Piv), 1.15 (s, 9H, Piv), 1.11 (s, 9H, Piv); ^{13}C NMR (126 MHz, $CDCl_3$): *δ* 178.24, 177.23, 177.16, 176.80, 96.75, 78.25, 75.56, 69.56, 69.44, 69.36, 65.22, 61.98, 55.13, 39.06, 39.04, 38.95, 38.89, 27.29 (2C), 27.24, 27.21; HRMS (ESI) *m/z* calcd for $C_{29}H_{46}O_{10}Na$ $(M + Na)^+$ 577.2983, found 577.2970.

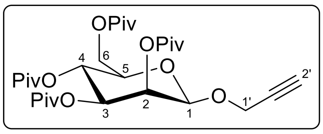

β-**3f**: colorless oil (6.1 mg); R_f = 0.40 (EtOAc/hexanes 1:4); $[\alpha]_D^{24}$ –32.1 (*c* 0.66, $CHCl_3$); ^{1}H NMR (500 MHz, $CDCl_3$): *δ* 5.46 (d, *J* = 3.2 Hz, 1H, H-C2), 5.39 (t, *J* = 9.9 Hz, 1H, H-C4), 5.15 (dd, *J* = 9.9, 3.2 Hz, 1H, H-C3), 4.96 (br s, 1H, H-C1), 4.38 (dd, *J* = 15.9, 2.4 Hz, 1H, H_a-C1'), 4.31 (dd, *J* = 15.9, 2.4 Hz, 1H, H_b-C1'), 4.24 (dd, *J* = 12.1, 1.8 Hz, H_a-C6), 4.16 (dd, *J* = 12.1, 5.1 Hz, 1H, H_b-C6), 3.75 (ddd, *J* = 9.9, 5.1, 1.8 Hz, 1H, H-C5), 2.44 (t, *J* = 2.4 Hz, 1H, H-C2'), 1.26 (s, 9H, Piv), 1.24 (s, 9H, Piv), 1.16 (s, 9H, Piv), 1.12 (s, ,9H, Piv); ^{13}C NMR (126 MHz, $CDCl_3$): *δ* 178.29, 177.42, 177.32, 176.80, 95.87, 78.18, 75.77, 72.83, 71.28, 68.63, 65.46, 62.08, 55.47, 39.17, 39.05, 38.96, 38.91, 27.29, 27.24, 27.19 (2C); HRMS (ESI) *m/z* calcd for $C_{29}H_{50}NO_{10}$ $(M + NH_4)^+$ 572.3429, found 572.3412.

1-O-Benzyl-2,3,4,6-tetra-O-pivaloyl-D-mannopyranose (3g)

Prepared according to the **method A** from mannosyl fluoride *α*-**1a** (100 mg, 0.193 mmol, 1.0 equiv) and benzyl alcohol (**2g**, 3.0 equiv) at 100 °C; yield 62%; α:β = 92:8 (NMR).

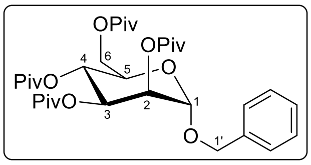

α-**3g**: colorless oil (67.1 mg); R_f = 0.47 (EtOAc/hexanes 1:9); $[\alpha]_D^{24}$ +46.8 (*c* 1.00, $CHCl_3$); ^{1}H NMR (500 MHz, $CDCl_3$): *δ* 7.43–7.28 (m, 5H, H-Ar), 5.49 (t, *J* = 10.0 Hz, 1H, H-C4), 5.43 (dd, *J* = 10.0, 3.1 Hz, 1H, H-C3), 5.32 (dd, *J* = 3.1, 1.7 Hz, 1H, H-C2), 4.87 (d, *J* = 1.7 Hz, 1H, H-C1), 4.74 (d, *J* = 11.8 Hz, 1H, H_a-C1'), 4.75 (d, *J* = 11.8 Hz, 1H, H_b-C1'), 4.18 (dd, *J* = 12.3, 4.5 Hz, 1H, H_a-C6), 4.11 (dd, *J* = 12.3, 1.7 Hz, 1H, H_b-C6), 4.06 (ddd, *J* = 10.0, 4.5, 1.7 Hz, 1H, H-C5), 1.26 (s, 9H, Piv), 1.25 (s, 9H, Piv), 1.16 (s, 9H, Piv), 1.12 (s, 9H, Piv); ^{13}C NMR (126 MHz, $CDCl_3$): *δ* 178.25, 177.33, 177.22, 176.85, 136.64, 128.71, 128.25, 127.96, 97.32, 69.77, 69.63 (2C), 69.29, 65.35, 62.17, 39.04 (2C), 38.94, 38.89, 27.32, 27.28, 27.24, 27.21; HRMS (ESI) *m/z* calcd for $C_{33}H_{50}O_{10}Na$ $(M + Na)^+$ 629.3296, found 629.3291.

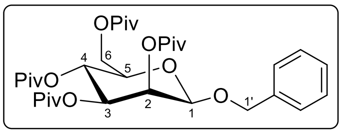

β-**3g**: colorless oil (5.3 mg); R_f = 0.25 (EtOAc/hexanes 1:9); $[\alpha]_D^{24}$ –33.9 (*c* 0.46, $CHCl_3$); ^{1}H NMR (500 MHz, $CDCl_3$): *δ* 7.38–7.27 (m, 5H, H-Ar), 5.45 (d, *J* = 3.2 Hz, 1H, H-C2), 5.42 (t, *J* = 9.9 Hz, 1H, H-C4), 5.06 (dd, *J* = 9.9, 3.2 Hz, 1H, H-C3), 4.85 (d, *J* = 12.2 Hz, 1H, H_a-C1'), 4.65 (br s, 1H, H-C1), 4.62 (d, *J* = 12.2 Hz, 1H, H_b-C1'), 4.25 (dd, *J* = 12.1, 1.8 Hz, 1H, H_a-C6), 4.17 (dd, *J* = 12.1, 4.9 Hz, 1H, H_b-C6), 3.67 (ddd, *J* = 9.9, 4.9, 1.8 Hz, 1H, H-C5), 1.26 (br s, 18H, 2×Piv), 1.15 (s, 9H, Piv), 1.11 (s, 9H, Piv); ^{13}C NMR (126 MHz, $CDCl_3$): *δ* 178.32, 177.44, 177.40, 176.79, 136.87, 128.56, 128.06, 127.91, 97.17, 72.71, 71.31, 70.34, 68.74, 65.48, 62.20, 39.15, 39.07, 38.94, 38.90, 27.31, 27.29, 27.19 (2C); HRMS (ESI) *m/z* calcd for $C_{33}H_{54}NO_{10}$ $(M + NH_4)^+$ 624.3742, found 624.3743.

1-O-Isopropyl-2,3,4,6-tetra-O-pivaloyl-α-D-mannopyranose (α-3h)

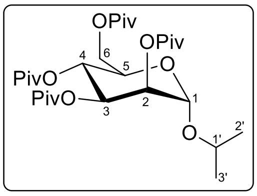

Prepared according to the **method A** from mannosyl fluoride *α*-**1a** (300 mg, 0.579 mmol, 1.0 equiv) and isopropanol (**2h**, 1.1 equiv) at 100 °C; yield 72% (233 mg); white solid; R_f = 0.50 (EtOAc/hexanes 1:9); $[\alpha]_D^{24}$ +30.0 (*c* 1.00, $CHCl_3$); ^{1}H NMR (300 MHz, $CDCl_3$): *δ* 5.46 (t, *J* = 10.0 Hz, 1H, H-C4), 5.38 (dd, *J* = 10.0, 3.0 Hz, 1H, H-C3), 5.16 (dd, *J* = 3.0, 1.9 Hz, 1H, H-C2), 4.86 (d, *J* = 1.9 Hz, 1H, H-C1), 4.18 (dd, *J* = 12.5, 4.6 Hz, 1H, H_a-C6), 4.14–4.04 (m, 2H, H-C5, H_b-C6), 3.91 (septet, *J* = 6.2 Hz, 1H, H-C1'), 1.26 (s, 9H, Piv), 1.24 (d, *J* = 6.2 Hz, 3H, H_3-C2'), 1.23 (s, 9H, Piv), 1.17 (d, *J* = 6.2 Hz, 3H, H_3-C3'), 1.16 (s, 9H, Piv), 1.11 (s, 9H, Piv); ^{13}C NMR (75.5 MHz, $CDCl_3$): *δ* 178.29, 177.39 (2C), 176.90, 96.22, 70.89, 70.34, 69.64, 69.08, 65.56, 62.40, 39.05, 39.02, 38.94, 38.89, 27.29 (2C), 27.24 (2C), 23.37, 21.67; HRMS (ESI) *m/z* calcd for $C_{29}H_{51}O_{10}$ $(M + H)^+$ 559.3477, found 559.3483.

1-O-(1,2-Dimethylpropyl)-2,3,4,6-tetra-O-pivaloyl-α-D-mannopyranose (α-3i)

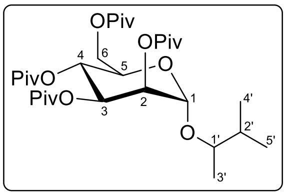

Prepared according to the **method A** from mannosyl fluoride *α*-**1a** (100 mg, 0.193 mmol, 1.0 equiv) and 3-methyl-2-butanol (**2i**, 3.0 equiv) at 100 °C; yield 49% (55.8 mg); diastereomeric mixture (54:46); white amorphous solid; R_f = 0.65 (EtOAc/hexanes 1:9); ^{1}H NMR (500 MHz, $CDCl_3$): *δ* 5.52–5.43 (m, 1H, H-C4), 5.42–5.35 (m, 1H, H-C3), 5.21–5.13 (m, 1H, H-C2), 4.88 (d, *J* = 1.8 Hz, 0.54H, H-C1 (*major*)), 4.85 (d, *J* = 1.8 Hz, 0.46H, H-C1 (*minor*)), 4.21–4.06 (m, 3H, H-C5, $H_{a,b}$-C6), 3.61–3.49 (m, 1H, H-C1'), 1.27 (s, 4.86H, Piv), 1.26 (s, 4.14H, Piv), 1.23 (br s, 9H, Piv). 1.85–1.73 (m, 1H, H-C2'), 1.18 (d, *J* = 6.6 Hz, 1.38H, H_3-C3' (*minor*)), 1.17 (s, 4.14H, Piv), 1.16 (s, 4.86H, Piv), 1.12 (br s, 9H, Piv), 1.10 (d, *J* = 6.4 Hz, 1.62H, H_3-C3' (*major*)), 0.96–0.92 (m, 3H, H_3-C4'), 0.92–0.88 (m, 3H, H_3-C5'); ^{13}C NMR (126 MHz, $CDCl_3$): *δ* 178.33, 178.29, 177.45, 177.43 (3C), 176.90 (2C), 98.19, 95.20, 81.53, 77.87, 70.47, 70.17, 69.74, 69.68, 69.39, 61.17, 65.51, 65.46, 62.48, 62.46, 39.07 (2C), 39.03 (2C), 38.95 (2C), 38.91 (2C), 33.54, 32.89, 27.30 (3C), 27.29, 27.25, 27.23 (3C), 18.62, 18.34, 18.24, 17.62 (2C), 15.15; HRMS (ESI) *m/z* calcd for $C_{31}H_{54}O_{10}Na$ $(M + Na)^+$ 609.3609, found (*major*) 609.3596 and (*minor*) 609.3631.

1-O-(2-Adamantyl)-2,3,4,6-tetra-O-pivaloyl-D-mannopyranose (3j)

Prepared according to the **method A** from mannosyl fluoride *α*-**1a** (100 mg, 0.193 mmol, 1.0 equiv) and 2-adamantanol (**2j**, 3.0 equiv) at 100 °C; yield 91%; α:β = 93:7 (NMR).

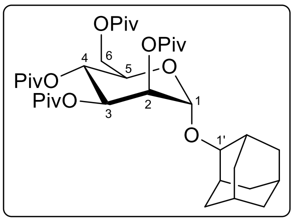

α-**3j**: white amorphous solid (106 mg); R_f = 0.46 (EtOAc/hexanes 1:9); $[\alpha]_D^{24}$ +40.2 (*c* 1.01, $CHCl_3$); ^{1}H NMR (500 MHz, $CDCl_3$): *δ* 5.48 (t, *J* = 10.0 Hz, 1H, H-C4), 5.44 (dd, *J* = 10.0, 3.0 Hz, 1H, H-C3), 5.23 (dd, *J* = 3.0, 1.9 Hz, 1H, H-C2), 4.94 (d, *J* = 1.9 Hz, 1H, H-C1), 4.17 (dd, *J* = 12.5, 4.7 Hz, 1H, H_a-C6), 4.14–4.08 (m, 2H, H-C5, H_b-C6), 3.80 (t, *J* = 3.4 Hz, 1H, H-C1'), 2.17–2.10 (m, 1H), 2.09–2.00 (m, 2H), 2.00–1.95 (m, 1H), 1.89–1.78 (m, 4H), 1.75–1.69 (m, 2H), 1.69–1.58 (m, 2H), 1.56–1.47 (m, 2H), 1.26 (s, 9H, Piv), 1.23 (s, 9H, Piv), 1.17 (s, 9H, Piv), 1.13 (s, 9H, Piv); ^{13}C NMR (126 MHz, $CDCl_3$): *δ* 178.29, 177.49, 177.44, 176.92, 95.79, 80.44, 70.45, 69.82, 69.28, 65.50, 62.48, 39.06, 39.02, 38.96, 38.92, 37.51, 36.78, 36.36, 33.51, 31.91, 31.49, 30.98, 27.39, 27.29 (2C), 27.27, 27.24 (2C); HRMS (ESI) *m/z* calcd for $C_{36}H_{58}O_{10}Na$ $(M + Na)^+$ 673.3922, found 673.3913.

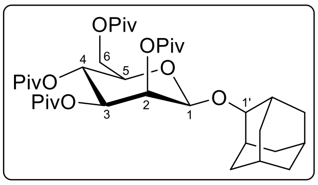

β-**3j**: isolated together with the excess of 2-adamantonol (**2j**) in 7% (8.3 mg) calculated yield; R_f = 0.30 (EtOAc/hexanes 1:9); characteristic chemical shifts reported: ^{1}H NMR (500 MHz, $CDCl_3$): *δ* 5.43 (d, *J* = 3.3 Hz, 1H, H-C2), 5.35 (t, *J* = 10.0 Hz, 1H, H-C4), 5.10 (dd, *J* = 10.0, 3.3 Hz, 1H, H-C3), 4.76 (br s, 1H, H-C1), 4.22 (dd, *J* = 12.1, 1.8 Hz, 1H, H_a-C6), 4.10 (dd, *J* = 12.1, 5.6 Hz, 1H, H_b-C6), 3.82–3.77 (m, 1H, H-C1'), 3.67 (ddd, *J* = 10.0, 5.6, 1.8 Hz, 1H, H-C5); HRMS (ESI) *m/z* calcd for $C_{36}H_{58}O_{10}Na$ $(M + Na)^+$ 673.3922, found 673.3920.

1-O-((1R,2S,5R)-2-Isopropyl-5-methylcyclohexyl)-2,3,4,6-tetra-O-pivaloyl-D-mannopyranose (3k)

Prepared according to the **method A** from mannosyl fluoride *α*-**1a** (100 mg, 0.193 mmol, 1.0 equiv) and (–)-menthol (**2k**, 3.0 equiv) at 100 °C; yield 77%; α:β = 91:9 (NMR).

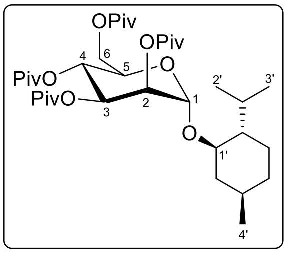

α-**3k**: white amorphous solid (88.2 mg); R_f = 0.54 (EtOAc/hexanes 1:9); $[\alpha]_D^{24}$ +2.0 (*c* 1.00, $CHCl_3$); ^{1}H NMR (500 MHz, $CDCl_3$): *δ* 5.50 (t, *J* = 10.1 Hz, 1H, H-C4), 5.37 (dd, *J* = 10.1, 3.2 Hz, 1H, H-C3), 5.14 (dd, *J* = 3.2, 1.6 Hz, 1H, H-C2), 4.86 (d, *J* = 1.6 Hz, 1H, H-C1), 4.21 (dd, *J* = 12.2, 4.0 Hz, 1H, H_a-C6), 4.19–4.14 (m, 1H, H-C5), 4.07 (dd, *J* = 12.2, 1.1 Hz, H_b-C6), 3.37 (td, *J* = 10.6, 4.3 Hz, 1H, H-C1'), 2.21–2.05 (m, 2H), 1.69–1.60 (m, 2H), 1.43–1.27 (m, 2H), 1.26 (s, 9H, Piv), 1.24 (s, 9H, Piv), 1.16 (s, 9H, Piv), 1.12 (s, 9H, Piv), 1.14–1.02 (m, 1H), 1.01–0.73 (m, 2H), 0.92 (d, *J* = 7.1 Hz, 3H, H_3-C2'), 0.90 (d, *J* = 6.7 Hz, 3H, H_3-C4'), 0.78 (d, *J* = 7.0 Hz, 1H, H_3-C3'); ^{13}C NMR (126 MHz, $CDCl_3$): *δ* 178.40, 177.45, 177.34, 176.83, 99.64, 82.99, 70.26, 69.57, 69.06, 65.48, 62.38, 48.47, 42.87, 39.07, 39.04, 38.94, 38.91, 34.38, 31.82, 27.33, 27.27 (2C), 27.23, 25.87, 23.34, 22.40, 21.16, 16.28; HRMS (ESI) *m/z* calcd for $C_{36}H_{62}O_{10}Na$ $(M + Na)^+$ 677.4235, found 677.4221.

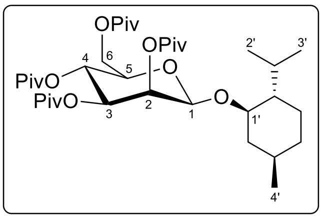

β-**3k**: white amorphous solid (8.6 mg); R_f = 0.41 (EtOAc/hexanes 1:9); $[\alpha]_D^{24}$ –61.7 (*c* 0.85, $CHCl_3$); ^{1}H NMR (500 MHz, $CDCl_3$): *δ* 5.41 (t, *J* = 10.1 Hz, 1H, H-C4), 5.37 (d, *J* = 3.1 Hz, 1H, H-C2), 5.10 (dd, *J* = 10.1, 3.1 Hz, 1H, H-C3), 4.78 (br s, 1H, H-C1), 4.23 (dd, *J* = 12.1, 1.9 Hz, 1H, H_a-C6), 4.06 (dd, *J* = 12.1, 4.3 Hz, 1H, H_b-C6), 3.66 (ddd, *J* = 10.1, 4.3, 1.9 Hz, 1H, H-C5), 3.48 (td, *J* = 10.7, 4.3 Hz, 1H, H-C1'), 2.30–2.19 (m, 1H), 2.00–1.90 (m, 1H), 1.69–1.52 (m, 2H), 1.34–1.20 (m, 1H), 1.25 (s, 9H, Piv), 1.23 (s, 9H, Piv), 1.19–1.07 (m, 1H), 1.15 (s, 9H, Piv), 1.11 (s, 9H, Piv), 1.00–0.76 (m, 3H), 0.90 (d, *J* = 6.6 Hz, 3H, H_3-C4'), 0.81 (d, *J* = 7.0 Hz, 3H, H_3-C2'), 0.70 (d, *J* = 6.8 Hz, 3H, H_3-C3'); ^{13}C NMR (126 MHz, $CDCl_3$): *δ* 178.31, 177.51, 177.26, 176.81, 95.15, 76.19, 72.33, 71.71, 69.51, 65.53, 62.12, 47.84, 39.96, 39.09, 39.00, 38.95, 38.90, 34.39, 31.63, 27.29, 27.22 (3C), 24.94, 23.06, 22.39, 20.99, 15.75; HRMS (ESI) *m/z* calcd for $C_{36}H_{62}O_{10}Na$ $(M + Na)^+$ 677.4235, found 677.4152.

1-O-Phenyl-2,3,4,6-tetra-O-pivaloyl-α-D-mannopyranose (α-3l)

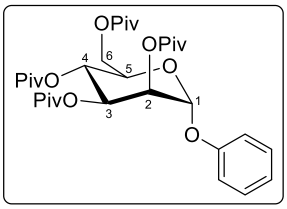

Prepared according to the **method A** from mannosyl fluoride *α*-**1a** (300 mg, 0.579 mmol, 1.0 equiv) and phenol (**2l**, 3.0 equiv) at 100 °C; yield 79% (271 mg); white solid; R_f = 0.43 (EtOAc/hexanes 1:9); $[\alpha]_D^{24}$ +45.5 (*c* 1.00, $CHCl_3$); ^{1}H NMR (500 MHz, $CDCl_3$): *δ* 7.33–7.27 (m, 2H, H-Ar), 7.14–7.09 (m, 2H, H-Ar), 7.08–7.01 (m, 1H, H-Ar), 5.61 (dd, *J* = 10.1, 3.2 Hz, 1H, H-C3), 5.54 (t, *J* = 10.1 Hz, 1H, H-C4), 5.46 (d, *J* = 1.8 Hz, 1H, H-C1), 5.44 (dd, *J* = 3.2, 1.8 Hz, 1H, H-C2), 4.21–4.08 (m, 3H, H-C5, $H_{a,b}$-C6), 1.29 (s, 9H, Piv), 1.18 (s, 9H, Piv), 1.17 (s, 9H, Piv), 1.15 (s, 9H, Piv); ^{13}C NMR (126 MHz, $CDCl_3$): *δ* 178.22, 177.42, 177.23, 176.89, 155.98, 129.77, 123.07, 116.67, 96.21, 69.74, 69.47, 69.37, 65.24, 62.14, 39.11, 38.97 (2C), 38.96, 27.30, 27.26, 27.23, 27.21; HRMS (ESI) *m/z* calcd for $C_{32}H_{48}O_{10}Na$ $(M + Na)^+$ 615.3140, found 615.3133.

1-Dodecylthio-2,3,4,6-tetra-O-pivaloyl-D-mannopyranose (3m)

Prepared according to the **method A** from mannosyl fluoride α-**1a** (400 mg, 0.771 mmol, 1.0 equiv) and 1-dodecanethiol (**2m**, 1.1 equiv) at 100 °C; yield 95%; α:β = 81:19 (NMR).

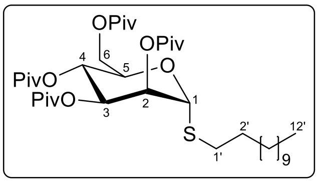

α-**3m**: orange oil (426 mg); R_f = 0.59 (EtOAc/hexanes 1:9); $[\alpha]_D^{24}$ +56.8 (*c* 1.00, $CHCl_3$); ^{1}H NMR (300 MHz, $CDCl_3$): *δ* 5.50 (t, *J* = 10.0 Hz, 1H, H-C4), 5.34 (dd, *J* = 3.2, 1.6 Hz, 1H, H-C2), 5.29 (dd, *J* = 10.0, 3.2 Hz, 1H, H-C3), 5.20 (d, *J* = 1.6 Hz, 1H, H-C1), 4.42 (ddd, *J* = 10.0, 4.5, 1.7 Hz, 1H, H-C5), 4.23 (dd, *J* = 12.5, 4.5 Hz, 1H, H_a-C6), 4.09 (dd, *J* = 12.5, 1.7 Hz, 1H, H_b-C6), 2.73–2.51 (m, 2H, H_2-C1'), 1.61 (quint, *J* = 7.0 Hz, 2H, H_2-C2'), 1.42–1.24 (m, 18H), 1.27 (s, 9H, Piv), 1.23 (s, 9H, Piv), 1.16 (s, 9H, Piv), 1.11 (s, 9H, Piv), 0.87 (t, *J* = 7.0 Hz, 3H, H_3-C12'); ^{13}C NMR (75.5 MHz, $CDCl_3$): *δ* 178.23, 177.25, 177.17, 176.82, 83.01, 71.28, 70.02, 69.49, 65.48, 62.26, 39.08, 39.02, 38.94, 38.87, 32.05, 31.48, 29.78, 29.76, 29.71, 29.66 (2C), 29.48, 29.28, 28.99, 27.29 (2C), 27.21 (2C), 22.82, 14.26; HRMS (ESI) *m/z* calcd for $C_{38}H_{68}O_9SNa$ (M + Na)$^+$ 723.4476, found 723.4454.

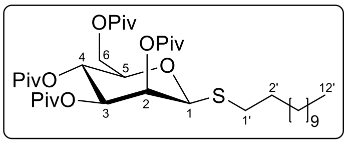

β-**3m**: orange oil (88.8 mg); R_f = 0.44 (EtOAc/hexanes 1:9); $[\alpha]_D^{24}$ –35.7 (*c* 1.00, $CHCl_3$); ^{1}H NMR (300 MHz, $CDCl_3$): *δ* 5.50 (dd, *J* = 3.4, 1.1 Hz, 1H, H-C2), 5.38 (t, *J* = 10.0 Hz, 1H, H-C4), 5.11 (dd, *J* = 10.0, 3.4 Hz, 1H, H-C3), 4.76 (d, *J* = 1.1 Hz, 1H, H-C1), 4.23 (dd, *J* = 12.5, 1.8 Hz, 1H, H_a-C6), 4.12 (dd, *J* = 12.5, 5.1 Hz, 1H, H_b-C6), 3.72 (ddd, *J* = 10.0, 5.1, 1.8 Hz, 1H, H-C5), 2.64 (t, *J* = 7.2 Hz, 2H, H_2-C1'), 1.58 (quint, *J* = 7.2 Hz, 2H, H_2-C2'), 1.40–1.23 (m, 18H), 1.28 (s, 9H, Piv), 1.22 (s, 9H, Piv), 1.14 (s, 9H, Piv), 1.10 (s, 9H, Piv), 0.87 (t, *J* = 7.1 Hz, 3H, H_3-C12'); ^{13}C NMR (75.5 MHz, $CDCl_3$): *δ* 178.24, 177.51, 177.10, 176.77, 83.18, 76.96, 72.39, 70.36, 65.17, 62.41, 39.21, 38.99, 38.93, 38.89, 32.03, 31.76, 29.84, 29.75 (2C), 29.70, 29.62, 29.46, 29.27, 28.91, 27.33, 27.23, 27.20, 27.17, 22.80, 14.24; HRMS (ESI) *m/z* calcd for $C_{38}H_{68}O_9SNa$ (M + Na)$^+$ 723.4476, found 723.4472.

1-Isopropylthio-2,3,4,6-tetra-O-pivaloyl-D-mannopyranose (3n)

Prepared according to the **method A** from mannosyl fluoride α-**1a** (168 mg, 0.324 mmol, 1.0 equiv) and 2-propanethiol (**2n**, 3.0 equiv) at 100 °C; yield 77%; α:β = 82:18 (NMR).

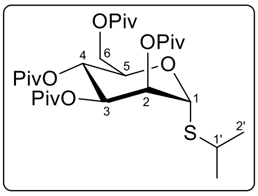

α-**3n**: white solid (115 mg); R_f = 0.53 (EtOAc/hexanes 1:9); $[\alpha]_D^{24}$ +64.9 (*c* 1.00, $CHCl_3$); ^{1}H NMR (300 MHz, $CDCl_3$): *δ* 5.49 (t, *J* = 10.0 Hz, 1H, H-C4), 5.34–5.22 (m, 3H, H-C1, H-C2, H-C3), 4.43 (ddd, *J* = 10.0, 4.4, 1.6 Hz, 1H, H-C5), 4.21 (dd, *J* = 12.4, 4.4 Hz, 1H, H_a-C6), 4.09 (dd, *J* = 12.4, 1.6 Hz, 1H, H_b-C6), 3.09 (septet, *J* = 6.7 Hz, 1H, H-C1'), 1.32 (d, *J* = 6.7 Hz, 6H, 2×H_3-C2'), 1.27 (s, 9H, Piv), 1.23 (s, 9H, Piv), 1.16 (s, 9H, Piv), 1.11 (s, 9H, Piv); ^{13}C NMR (75.5 MHz, $CDCl_3$): *δ* 178.27, 177.27, 177.23, 176.84, 82.28, 71.64, 70.05, 69.60, 65.52, 62.36, 39.10, 39.01, 38.95, 38.86, 36.45, 27.29 (2C), 27.21 (2C), 23.94, 23.67; HRMS (ESI) *m/z* calcd for $C_{29}H_{50}O_9SK$ (M + K)$^+$ 613.2807, found 613.2798.

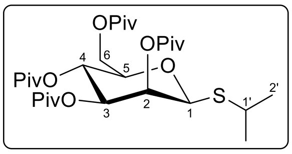

β-**3n**: colorless oil (29.0 mg); R_f = 0.25 (EtOAc/hexanes 1:9); $[\alpha]_D^{24}$ –26.4 (*c* 2.11, $CHCl_3$); ^{1}H NMR (300 MHz, $CDCl_3$): *δ* 5.51 (d, *J* = 3.3 Hz, 1H, H-C2), 5.35 (t, *J* = 10.1 Hz, 1H, H-C4), 5.13 (dd, *J* = 10.1, 3.3 Hz, 1H, H-C3), 4.81 (br s, 1H, H-C1), 4.24 (dd, *J* = 12.3, 1.8 Hz, 1H, H_a-C6), 4.10 (dd, *J* = 12.3, 5.7 Hz, 1H, H_b-C6), 3.74 (ddd, *J* = 10.1, 5.7, 1.8 Hz, 1H, H-C5), 3.14 (septet, *J* = 6.8 Hz, 1H, H-C1'), 1.30 (d, *J* = 6.8 Hz, 6H, 2×H_3-C2'), 1.28 (s, 9H, Piv), 1.22 (s, 9H, Piv), 1.15 (s, 9H, Piv), 1.11 (s, 9H, Piv); ^{13}C NMR (75.5 MHz, $CDCl_3$): *δ* 178.29, 177.53, 177.11, 176.85, 82.24, 76.95, 72.41, 70.80, 65.25, 62.63, 39.22, 38.98, 38.97, 38.91, 35.96, 27.35, 27.23 (2C), 27.21, 23.97, 23.54; HRMS (ESI) *m/z* calcd for $C_{29}H_{54}NO_9S$ (M + NH_4)$^+$ 592.3514, found 592.3507.

1-Cyclohexylthio-2,3,4,6-tetra-O-pivaloyl-D-mannopyranose (3o)

Prepared according to the **method A** from mannosyl fluoride α-**1a** (300 mg, 0.579 mmol, 1.0 equiv) and cyclohexanethiol (**2o**, 1.0 equiv) at 100 °C; yield 87%; α:β = 84:16 (NMR).

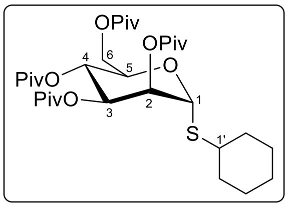

α-**3o**: colorless oil (259 mg); R_f = 0.70 (EtOAc/hexanes 1:4); $[\alpha]_D^{24}$ +65.8 (*c* 1.00, $CHCl_3$); ^{1}H NMR (300 MHz, $CDCl_3$): *δ* 5.49 (t, *J* = 10.0 Hz, 1H, H-C4), 5.34–5.29 (m, 2H, H-C1, H-C2), 5.27 (dd, *J* = 10.0, 2.9 Hz, 1H, H-C3) 4.46 (ddd, *J* = 10.0, 4.4, 1.4 Hz, 1H, H-C5), 4.21 (dd, *J* = 12.5, 4.4 Hz, 1H, H_a-C6), 4.10 (dd, *J* = 12.5, 1.4 Hz, 1H, H_b-C6), 2.95–2.78 (m, 1H, H-C1'), 2.09–1.92 (m, 2H), 1.86–1.67 (m, 2H), 1.65–1.55 (m, 1H), 1.51–1.26 (m, 5H), 1.27 (s, 9H, Piv), 1.24 (s, 9H, Piv), 1.16 (s, 9H, Piv), 1.11 (s, 9H, Piv); ^{13}C NMR (75.5 MHz, $CDCl_3$): *δ* 178.30, 177.27, 177.23, 176.85, 82.22, 71.76, 70.06, 69.56, 65.56, 62.43, 44.82, 39.10, 39.03, 38.95, 38.87, 34.22, 33.66, 27.30 (2C), 27.22 (2C), 26.09, 25.92, 25.72; HRMS (ESI) *m/z* calcd for $C_{32}H_{54}O_9SNa$ (M + Na)$^+$ 637.3381, found 637.3378.

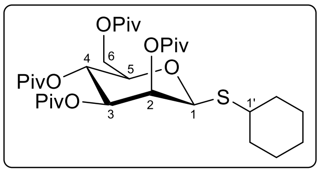

β-**3o**: colorless oil (50.3 mg); R_f = 0.58 (EtOAc/hexanes 1:4); $[\alpha]_D^{24}$ –45.9 (*c* 0.90, $CHCl_3$); ^{1}H NMR (300 MHz, $CDCl_3$): *δ* 5.50 (dd, *J* = 3.4, 1.1 Hz, 1H, H-C2), 5.33 (t, *J* = 10.0 Hz, 1H, H-C4), 5.12 (dd, *J* = 10.0, 3.4 Hz, 1H, H-C3), 4.83 (d, *J* = 1.1 Hz, 1H, H-C1), 4.25 (dd, *J* = 12.2, 1.3 Hz, 1H, H_a-C6), 4.08 (dd, *J* = 12.2, 5.8 Hz, 1H, H_b-C6), 3.74 (ddd, *J* = 10.0, 5.8, 1.3 Hz, 1H, H-C5), 2.94–2.78 (m, 1H, H-C1'), 2.04–1.90 (m, 2H), 1.84–1.68 (m, 2H), 1.66–1.58 (m, 1H), 1.46–1.26 (m, 5H), 1.28 (s, 9H, Piv), 1.22 (s, 9H, Piv), 1.15 (s, 9H, Piv), 1.11 (s, 9H, Piv); ^{13}C NMR (75.5 MHz, $CDCl_3$): *δ* 178.28, 177.53, 177.11, 176.88, 81.84, 76.95, 72.43, 70.90, 65.32, 62.70, 44.26, 39.23, 39.00, 38.97, 38.91, 34.21, 33.68, 27.37, 27.26, 27.24, 27.21, 26.25, 26.17, 25.75; HRMS (ESI) *m/z* calcd for $C_{32}H_{54}O_9SNa$ (M + Na)$^+$ 637.3381, found 637.3379.

1-(4-Chlorophenylthio)-2,3,4,6-tetra-O-pivaloyl-D-mannopyranose (3p)

Prepared according to the **method A** from mannosyl fluoride α-**1a** (300 mg, 0.579 mmol, 1.0 equiv) and 4-chlorothiophenol (**2p**, 3.0 equiv) at 100 °C; yield 93%; α:β = 81:19 (NMR).

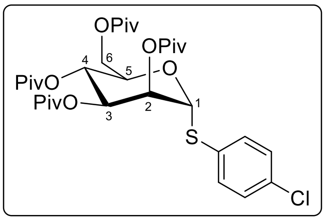

α-**3p**: white solid (281 mg); R_f = 0.46 (EtOAc/hexanes 1:9); $[\alpha]_D^{24}$ +73.7 (*c* 1.00, $CHCl_3$); ^{1}H NMR (500 MHz, $CDCl_3$): *δ* 7.44–7.40 (m, 2H, H-Ar), 7.31–7.27 (m, 2H, H-Ar), 5.54 (t, *J* = 10.1 Hz, 1H, H-C4), 5.49 (dd, *J* = 3.1, 1.6 Hz, 1H, H-C2), 5.40 (d, *J* = 1.6 Hz, 1H, H-C1), 5.30 (dd, *J* = 10.1, 3.1 Hz, 1H, H-C3), 4.53 (ddd, *J* = 10.1, 4.6, 1.6 Hz, 1H, H-C5), 4.23 (dd, *J* = 12.6, 4.6 Hz, 1H, H_a-C6), 4.11 (dd, *J* = 12.6, 1.6 Hz, 1H, H_b-C6), 1.26 (s, 9H, Piv), 1.21 (s, 9H, Piv), 1.18 (s, 9H, Piv), 1.13 (s, 9H, Piv); ^{13}C NMR (126 MHz, $CDCl_3$): *δ* 178.22, 177.35, 177.15, 176.79, 134.53, 133.38, 131.58, 129.56, 86.44, 70.91, 70.33, 69.84, 65.27, 62.21, 39.11, 39.00, 38.99, 38.93, 27.25 (2C), 27.22 (2C); HRMS (ESI) *m/z* calcd for $C_{32}H_{47}ClO_9SNa$ (M + Na)$^+$ 665.2522, found 665.2551.

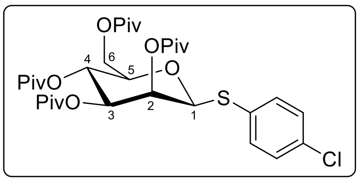

β-**3p**: white solid (64.3 mg); R_f = 0.29 (EtOAc/hexanes 1:9); $[\alpha]_D^{24}$ –27.7 (*c* 1.06, $CHCl_3$); ^{1}H NMR (500 MHz, $CDCl_3$): *δ* 7.46–7.40 (m, 2H, H-Ar), 7.30–7.23 (m, 2H, H-Ar), 5.66 (dd, *J* = 3.4, 1.1 Hz, 1H, H-C2), 5.40 (t, *J* = 10.1 Hz, 1H, H-C4), 5.10 (dd, *J* = 10.1, 3.4 Hz, 1H, H-C3), 4.93 (d, *J* = 1.1 Hz, 1H, H-C1), 4.28 (dd, *J* = 12.4, 1.8 Hz, 1H, H_a-C6), 4.11 (dd, *J* = 12.4, 5.6 Hz, 1H, H_b-C6), 3.75 (ddd, *J* = 10.1, 5.6, 1.8 Hz, 1H, H-C5), 1.33 (s, 9H, Piv), 1.24 (s, 9H, Piv), 1.16 (s, 9H, Piv), 1.12 (s, 9H, Piv); ^{13}C NMR (126 MHz, $CDCl_3$): *δ* 178.23, 177.47, 177.07, 176.82, 134.42, 133.20, 132.33, 129.41, 86.44, 77.02, 72.15, 70.38, 64.98, 62.47, 39.33, 39.02, 38.98, 38.94, 27.36, 27.26, 27.23, 27.20; HRMS (ESI) *m/z* calcd for $C_{32}H_{47}ClO_9SNa$ (M + Na)$^+$ 665.2522, found 665.2518.

1-(2,3,4,6-Tetra-O-pivaloyl-α-D-mannopyranosyl)-2,3,4-trimethoxybenzene (α-3q)

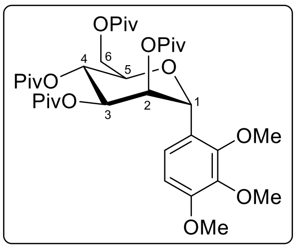

Prepared according to the **method A** from mannosyl fluoride α-**1a** (100 mg, 0.193 mmol, 1.0 equiv) and 1,2,3-trimethoxybenzene (**2q**, 3.0 equiv) at 100 °C; yield 47% (60.8 mg); white amorphous solid; R_f = 0.39 (EtOAc/hexanes 1:4); $[\alpha]_D^{24}$ –2.8 (*c* 1.01, $CHCl_3$); ^1H NMR (500 MHz, $CDCl_3$): δ 7.10 (d, *J* = 8.8 Hz, 1H, H-Ar), 6.59 (d, *J* = 8.8 Hz, 1H, H-Ar), 5.60 (dd, *J* = 3.2, 1.1 Hz, 1H, H-C2), 5.56 (t, *J* = 10.1 Hz, 1H, H-C4), 5.34 (dd, *J* = 10.1, 3.2 Hz, 1H, H-C3), 4.99 (br s, 1H, H-C1), 4.30–4.20 (m, 2H, $H_{a,b}$-C6), 3.95 (s, 3H, OMe), 3.89–3.84 (m, 1H, H-C5), 3.81 (s, 3H, OMe), 3.79 (s, 3H, OMe), 1.27 (s, 9H, Piv), 1.17 (s, 9H, Piv), 1.10 (s, 9H, Piv), 1.05 (s, 9H, Piv); ^{13}C NMR (126 MHz, $CDCl_3$): δ 178.30, 177.55, 176.88, 176.78, 153.65, 150.16, 141.42, 122.32, 121.98, 106.41, 76.56, 74.28, 72.75, 69.74, 65.37, 62.18, 60.96, 60.83, 56.17, 39.07, 38.95, 38.89, 38.87, 27.30 (2C), 27.26, 27.25; HRMS (ESI) *m/z* calcd for $C_{35}H_{55}O_{12}$ $(M + H)^+$ 667.3688, found 667.3676.

1-O-Cyclopropylmethyl-2,3,4,6-tetra-O-pivaloyl-α-D-mannopyranose (α-3r)

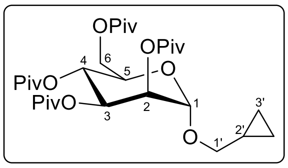

Prepared according to the **method A** from mannosyl fluoride α-**1a** (100 mg, 0.193 mmol, 1.0 equiv) and cyclopropylmethanol (**2r**, 3.0 equiv) at 100 °C; yield 21% (22.7 mg); white amorphous solid; R_f = 0.32; (EtOAc/hexanes 1:9); $[\alpha]_D^{24}$ +30.0 (*c* 0.63, $CHCl_3$); ^1H NMR (500 MHz, $CDCl_3$): δ 5.47 (t, *J* = 10.1 Hz, 1H, H-C4), 5.43 (dd, *J* = 10.1, 3.0 Hz, 1H, H-C3), 5.26 (dd, *J* = 3.0, 1.8 Hz, 1H, H-C2), 4.82 (d, *J* = 1.8 Hz, 1H, H-C1), 4.18 (dd, *J* = 12.5, 4.7 Hz, 1H, H_a-C6), 4.12 (dd, *J* = 12.5, 1.7 Hz, 1H, H_b-C6), 4.06 (ddd, *J* = 10.1, 4.7, 1.7 Hz, 1H, H-C5), 3.49 (dd, *J* = 10.5, 7.0 Hz, 1H, H_a-C1'), 3.35 (dd, *J* = 10.5, 7.0 Hz, 1H, H_b-C1'), 1.27 (s, 9H, Piv), 1.23 (s, 9H, Piv), 1.16 (s, 9H, Piv), 1.12 (s, 9H, Piv), 1.12–1.02 (m, 1H, H-C2'), 0.63–0.50 (m, 2H, H_a-C3'), 0.28–0.16 (m, 2H, H_b-C3'); ^{13}C NMR (126 MHz, $CDCl_3$): δ 178.28, 177.35 (2C), 176.90, 97.51, 73.10, 69.85, 69.63, 69.03, 65.50, 62.32, 39.06, 39.03, 38.96, 38.90, 27.30 (2C), 27.25, 27.23, 10.38, 3.51, 3.13; HRMS (ESI) *m/z* calcd for $C_{30}H_{50}O_{10}Na$ $(M + Na)^+$ 593.3296, found 593.3307.

1-O-Cyclobutyl-2,3,4,6-tetra-O-pivaloyl-α-D-mannopyranose (α-3s)

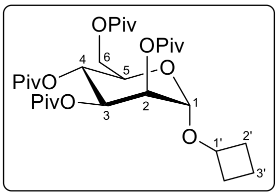

Isolated as a side-product in the synthesis of mannoside α-**3r**; yield 23% (25.4 mg); white solid; R_f = 0.38; (EtOAc/hexanes 1:9); $[\alpha]_D^{24}$ +32.0 (*c* 0.61, $CHCl_3$); ^1H NMR (500 MHz, $CDCl_3$): δ 5.47 (t, *J* = 10.1 Hz, 1H, H-C4), 5.40 (dd, *J* = 10.1, 3.1 Hz, 1H, H-C3), 5.20 (dd, *J* = 3.1, 1.7 Hz, 1H, H-C2), 4.76 (d, *J* = 1.7 Hz, 1H, H-C1), 4.19 (dd, *J* = 12.3, 4.2 Hz, 1H, H_a-C6), 4.19–4.12 (m, 1H, H-C1'), 4.10 (dd, *J* = 12.3, 1.7 Hz, 1H, H_b-C6), 4.07 (ddd, *J* = 10.1, 4.2, 1.7 Hz, 1H, H-C5), 2.28–2.15 (m, 2H, H_a-C2'), 2.13–1.93 (m, 2H, H_b-C2'), 1.77–1.66 (m, 1H, H_a-C3'), 1.54–1.44 (m, 1H, H_b-C3'), 1.26 (s, 9H, Piv), 1.24 (s, 9H, Piv), 1.16 (s, 9H, Piv), 1.12 (s, 9H, Piv); ^{13}C NMR (126 MHz, $CDCl_3$): δ 178.31, 177.35 (2C), 176.85, 96.23, 71.75, 69.92, 69.57, 69.20, 65.41, 62.24, 39.06, 39.04, 38.92, 38.91, 31.00, 30.25, 27.30, 27.28, 27.25, 27.22, 12.76; HRMS (ESI) *m/z* calcd for $C_{30}H_{50}O_{10}Na$ $(M + Na)^+$ 593.3296, found 593.3275.

Bis-α-D-mannopyranoside α-8a

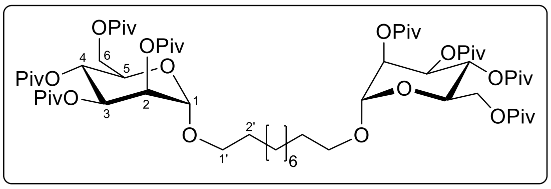

Prepared according to the **method A** from mannosyl fluoride α-**1a** (400 mg, 0.771 mmol, 2.1 equiv) and 1,10-decanediol (**7a**, 1.0 equiv) at 100 °C; yield 84% (359 mg); colorless oil; R_f = 0.66 (EtOAc/hexanes 1:4); $[\alpha]_D^{24}$ +33.6 (*c* 1.00, $CHCl_3$); ^1H NMR (300 MHz, $CDCl_3$): δ 5.46 (t, *J* = 9.9 Hz, 1H, H-C4), 5.38 (dd, *J* = 9.9, 3.0 Hz, 1H, H-C3), 5.23 (dd, *J* = 3.0, 1.8 Hz, 1H, H-C2), 4.75 (d, *J* = 1.8 Hz, 1H, H-C1), 4.19 (dd, *J* = 12.3, 4.2 Hz, 1H, H_a-C6), 4.13 (dd, *J* = 12.3, 1.7 Hz, 1H, H_b-C6), 4.02 (ddd, *J* = 9.9, 4.2, 1.7 Hz, 1H, H-C5), 3.69 (dt, *J* = 9.5, 6.7 Hz, 1H, H_a-C1'), 3.43 (dt, *J* = 9.5, 6.7 Hz, 1H, H_b-C1'), 1.61 (quint, *J* = 6.7 Hz, 2H, H_2-C2'), 1.42–1.28 (m, 6H), 1.26 (s, 9H, Piv), 1.24 (s, 9H, Piv), 1.16 (s, 9H, Piv), 1.12 (s, 9H, Piv); ^{13}C NMR (75.5 MHz, $CDCl_3$): δ 178.25, 177.33, 177.32, 176.88, 97.88, 69.79, 69.64, 69.01, 68.61, 65.46, 62.27, 39.05, 39.04, 38.95, 38.90, 29.69, 29.55, 29.48, 27.30 (2C), 27.24, 27.23, 26.23. HRMS (ESI) *m/z* calcd for $C_{62}H_{110}NO_{20}$ $(M + NH_4)^+$ 1188.7616, found 1188.7603.

Bis-α-D-mannopyranoside α-8b

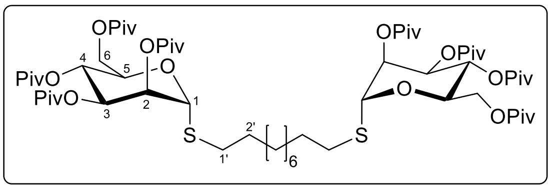

Prepared according to the **method A** from mannosyl fluoride α-**1a** (400 mg, 0.771 mmol, 2.1 equiv) and 1,10-decanedithiol (**7b**, 1.0 equiv) at 100 °C; yield 76% (336 mg); colorless oil; R_f = 0.74 (EtOAc/hexanes 1:4); $[\alpha]_D^{24}$ +48.3 (*c* 1.00, $CHCl_3$); ^1H NMR (300 MHz, $CDCl_3$): δ 5.50 (t, *J* = 10.0 Hz, 1H, H-C4), 5.35–5.26 (m, 2H, H-C2, H-C3), 5.20 (d, *J* = 1.6 Hz, 1H, H-C1), 4.43 (ddd, *J* = 10.0, 4.4, 1.5 Hz, 1H, H-C5), 4.23 (dd, *J* = 12.5, 4.4 Hz, 1H, H_a-C6), 4.10 (dd, *J* = 12.5, 1.5 Hz, 1H, H_b-C6), 2.73–2.51 (m, 2H, H_2-C1'), 1.62 (quint, *J* = 7.3 Hz, 2H, H_2-C2'), 1.43–1.25 (m, 6H), 1.27 (s, 9H, Piv), 1.24 (s, 9H, Piv), 1.16 (s, 9H, Piv), 1.11 (s, 9H, Piv); ^{13}C NMR (75.5 MHz, $CDCl_3$): δ 178.24, 177.26, 177.18, 176.82, 83.02, 71.29, 70.03, 69.51, 65.50, 62.27, 39.09, 39.03, 38.95, 38.88, 31.48, 29.67, 29.61, 29.29, 29.00, 27.30 (2C), 27.22 (2C). HRMS (ESI) *m/z* calcd for $C_{62}H_{107}O_{18}S_2$ $(M + H)^+$ 1203.6893, found 1203.6896.

1-O-(2-Phenylethyl)-2,3,4,6-tetra-O-pivaloyl-D-glucopyranose (10a)

Prepared according to the **method A** from glucosyl fluoride β-**9** (50 mg, 0.096 mmol, 1.0 equiv) and 2-phenylethanol (**2a**, 3.0 equiv) at 100 °C; yield 61% (36.2 mg); α:β = 15:85 (NMR).

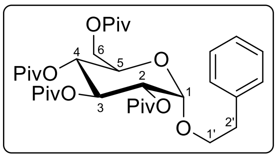

α-**10a**: colorless oil; R_f = 0.38 (EtOAc/hexanes 1:9); $[\alpha]_D^{24}$ +57.9 (*c* 0.47, $CHCl_3$); ^1H NMR (500 MHz, $CDCl_3$): δ 7.33–7.28 (m, 2H, H-Ar), 7.26–7.18 (m, 3H, H-Ar), 5.51 (t, *J* = 9.9 Hz, 1H, H-C4), 5.06 (d, *J* = 3.8 Hz, 1H, H-C1), 5.03 (t, *J* = 9.9 Hz, 1H, H-C3), 4.76 (dd, *J* = 9.9, 3.8 Hz, 1H, H-C2), 3.93 (dd, *J* = 12.4, 1.9 Hz, 1H, H_a-C6), 3.88 (dd, *J* = 12.4, 4.7 Hz, 1H, H_b-C6), 3.85–3.78 (m, 1H, H_a-C1'), 3.68–3.60 (m, 1H, H_b-C1'), 3.46 (ddd, *J* = 9.9, 4.7, 1.9 Hz, 1H, H-C5), 2.97–2.83 (m, 2H, H_2-C2'), 1.19 (s, 9H, Piv), 1.14 (s, 9H, Piv), 1.14 (s, 9H, Piv), 1.12 (s, 9H, Piv); ^{13}C NMR (126 MHz, $CDCl_3$): δ 178.20, 177.90, 177.17, 176.59, 138.78, 129.20, 128.61, 126.56, 95.17, 71.35, 69.73, 68.88, 67.77, 67.54, 61.83, 39.00, 38.88, 38.86 (2C), 36.16, 27.33, 27.27, 27.20, 27.13; HRMS (ESI) *m/z* calcd for $C_{34}H_{56}NO_{10}$ $(M + NH_4)^+$ 638.3899, found 638.3898.

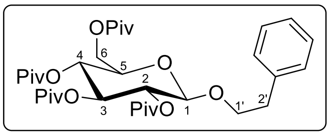

β-**10a**: white amorphous solid; R_f = 0.31 (EtOAc/hexanes 1:9); $[\alpha]_D^{24}$ +2.2 (*c* 1.57, $CHCl_3$); ^1H NMR (500 MHz, $CDCl_3$): δ 7.29–7.24 (m, 2H, H-Ar), 7.23–7.14 (m, 3H, H-Ar), 5.30 (dd, *J* = 9.7, 9.4 Hz, 1H, H-C3), 5.10 (dd, *J* = 9.9, 9.7 Hz, 1H, H-C4), 5.03 (dd, *J* = 9.4, 8.2 Hz, 1H, H-C2), 4.53 (d, *J* = 8.2 Hz, 1H, H-C1), 4.21 (dd, *J* = 10.4, 1.8 Hz, 1H, H_a-C6), 4.10–3.99 (m, 2H, H_b-C6, H_a-C1'), 3.72 (ddd, *J* = 9.9, 5.6, 1.8 Hz, 1H, H-C5), 3.71–3.63 (m, 1H, H_b-C1'), 2.95–2.81 (m, 2H, H_2-C2'), 1.21 (s, 9H, Piv), 1.15 (s, 9H, Piv), 1.11 (s, 9H, Piv), 1.09 (s, 9H, Piv); ^{13}C NMR (126 MHz, $CDCl_3$): δ 178.23, 177.37, 176.62, 176.58, 138.25, 129.03, 128.55, 126.50, 101.16, 72.45, 72.38, 71.22, 70.66, 68.22, 62.16, 39.01, 38.90, 38.85, 38.80, 36.32, 27.28, 27.25, 27.18, 27.12; HRMS (ESI) *m/z* calcd for $C_{34}H_{56}NO_{10}$ $(M + NH_4)^+$ 638.3899, found 638.3891.

1-(2-Phenylethylthio)-2,3,4,6-tetra-O-pivaloyl-D-glucopyranose (10b)

Prepared according to the **method A** from glucosyl fluoride β-**9** (50 mg, 0.096 mmol, 1.0 equiv) and 2-phenylethanethiol (**2c**, 3.0 equiv) at 100 °C; yield 57% (34.9 mg); α:β = 24:76 (NMR).

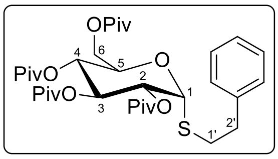

α-**10b**: white amorphous solid; R_f = 0.59 (EtOAc/hexanes 3:17); $[\alpha]_D^{24}$ +53.7 (*c* 1.17, $CHCl_3$); ^1H NMR (500 MHz, $CDCl_3$): δ 7.31–7.26 (m, 2H, H-Ar), 7.23–7.15 (m, 3H, H-Ar), 5.69 (d, *J* = 5.9 Hz, 1H, H-C1), 5.44 (t, *J* = 9.8 Hz, 1H, H-C3), 5.09 (t, *J* = 9.8 Hz, 1H, H-C4), 4.98 (dd, *J* = 9.8, 5.9 Hz, 1H, H-C2), 4.37 (ddd, *J* = 9.8, 4.5, 2.3 Hz, 1H, H-C5), 4.12–4.03 (m, 2H, $H_{a,b}$-C6), 2.92–2.80 (m, 3H, H_a-C1', H_2-C2'), 2.78–2.69 (m, 1H, H_b-C1'), 1.18 (s, 9H, Piv), 1.16 (s, 9H, Piv), 1.15 (s, 9H, Piv), 1.12 (s, 9H, Piv); ^{13}C NMR (126 MHz, $CDCl_3$): δ 178.22, 177.55, 176.98, 176.68, 140.03, 128.68, 128.63, 126.62, 81.60, 71.17, 70.10, 68.13, 67.98, 62.19, 38.98, 38.91, 38.84, 38.81, 35.86, 30.99, 27.32, 27.27, 27.21, 27.19; HRMS (ESI) *m/z* calcd for $C_{34}H_{56}NO_9S$ $(M + NH_4)^+$ 654.3670, found 654.3645.

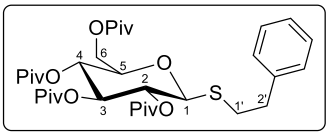

β-**10b**: colorless oil; R_f = 0.54 (EtOAc/hexanes 3:17); $[\alpha]_D^{24}$ –8.2 (*c* 1.10, $CHCl_3$); ^1H NMR (500 MHz, $CDCl_3$): δ 7.31–7.25 (m, 2H, H-Ar), 7.23–7.16 (m, 3H, H-Ar), 5.31 (t, *J* = 9.8 Hz, 1H, H-C3), 5.12 (t, *J* = 9.8 Hz, 1H, H-C4), 5.07 (t, *J* = 9.8 Hz, 1H, H-C2), 4.46 (d, *J* = 9.8 Hz, 1H, H-C1), 4.22 (dd, *J* = 12.4, 1.9 Hz, 1H, H_a-C6), 4.06 (dd, *J* = 12.4, 5.4 Hz, 1H, H_b-C6), 3.70 (ddd, *J* = 9.8, 5.4, 1.9 Hz, 1H, H-C5), 3.02–2.86 (m, 4H, H_2-C(1',2')), 1.19 (s, 9H, Piv), 1.15 (s, 9H, Piv), 1.14 (s, 9H, Piv), 1.11 (s, 9H, Piv); ^{13}C NMR (126 MHz, $CDCl_3$): δ 178.17, 177.28, 176.69, 176.54, 140.11, 128.67, 128.59, 126.59, 83.52, 76.62, 73.35, 69.62, 67.89, 62.22, 38.98, 38.90, 38.86, 38.80, 36.29, 30.82, 27.29, 27.25, 27.23, 27.19; HRMS (ESI) *m/z* calcd for $C_{34}H_{56}NO_9S$ $(M + NH_4)^+$ 654.3670, found 654.3667.

1-O-(2-Phenylethyl)-2,3,4,6-tetra-O-acetyl-D-mannopyranose (13a)

Prepared according to the **method A** from mannosyl fluoride α-**11** (97.1 mg, 0.277 mmol, 1.0 equiv) and 2-phenylethanol (**2a**, 3.0 equiv) at 100 °C; yield 55% (69.3 mg); α:β = 91:9 (NMR).

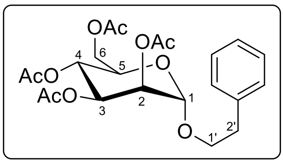

α-**13a**: colorless oil; R_f = 0.62 (EtOAc/hexanes 1:1); $[\alpha]_D^{24}$ +55.2 (*c* 1.04, $CHCl_3$); ^1H NMR (500 MHz, $CDCl_3$): δ 7.33–7.28 (m, 2H, H-Ar), 7.25–7.19 (m, 3H, H-Ar), 5.28 (dd, *J* = 9.9, 3.2 Hz, 1H, H-C3), 5.22 (dd, *J* = 3.2, 1.8 Hz, 1H, H-C2), 5.20 (t, *J* = 9.9 Hz, 1H, H-C4), 4.79 (d, *J* = 1.8 Hz, 1H, H-C1), 4.11 (dd, *J* = 12.4, 5.0 Hz, 1H, H_a-C6), 3.92 (dd, *J* = 12.4, 2.4 Hz, 1H, H_b-C6), 3.83 (dt, *J* = 9.4, 7.4 Hz, 1H, H_a-C1'), 3.72 (dt, *J* = 9.4, 6.2 Hz, 1H, H_b-C1'), 3.47 (ddd, *J* = 9.9, 5.0, 2.4 Hz, 1H, H-C5), 2.91 (dd, *J* = 7.4, 6.2 Hz, 2H, H_2-C2'), 2.13 (s, 3H, Ac), 2.07 (s, 3H, Ac), 2.02 (s, 3H, Ac), 1.99 (s, 3H, Ac); ^{13}C NMR (126 MHz, $CDCl_3$): δ 170.76, 170.20, 170.04, 169.82, 138.64, 129.14, 128.65, 126.57, 97.31, 69.75, 69.20, 68.94, 68.41, 66.08, 62.38, 36.07, 21.03, 20.86, 20.84, 20.82; HRMS (ESI) *m/z* calcd for $C_{22}H_{32}NO_{10}$ $(M + NH_4)^+$ 470.2021, found 470.2013.

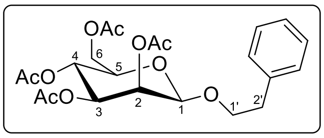

β-**13a**: colorless oil; R_f = 0.59 (EtOAc/hexanes 1:1); $[\alpha]_D^{24}$ –16.4 (*c* 0.52, $CHCl_3$); ^1H NMR (500 MHz, $CDCl_3$): δ 7.32–7.26 (m, 2H, H-Ar), 7.24–7.16 (m, 3H, H-Ar), 5.46 (d, *J* = 3.3 Hz, 1H, H-C2), 5.25 (t, *J* = 10.0 Hz, 1H, H-C4), 5.01 (dd, *J* = 10.0, 3.3 Hz, 1H, H-C3), 4.59 (br s, 1H, H-C1), 4.29 (dd, *J* = 12.3, 5.6 Hz, 1H, H_a-C6), 4.14 (dd, *J* = 12.3, 2.7 Hz, 1H, H_b-C6), 4.10 (dt, *J* = 9.4, 7.1 Hz, 1H, H_a-C1'), 3.69 (dt, *J* = 9.4, 7.7 Hz, 1H, H_b-C1'), 3.62 (ddd, *J* = 10.0, 5.6, 2.7 Hz, 1H, H-C5), 2.90 (dd, *J* = 7.7, 7.1 Hz, 2H, H_2-C2'), 2.18 (s, 3H, Ac), 2.08 (s, 3H, Ac), 2.04 (s, 3H, Ac), 1.99 (s, 3H, Ac); ^{13}C NMR (126 MHz, $CDCl_3$): δ 170.88, 170.49, 170.18, 169.74, 138.29, 129.15, 128.57, 126.60, 98.97, 72.56, 71.30, 71.21, 68.88, 66.24, 62.70, 36.25, 21.01, 20.94, 20.85, 20.74; HRMS (ESI) *m/z* calcd for $C_{22}H_{28}O_{10}Na$ $(M + Na)^+$ 475.1575, found 475.1598.

1-(2-Phenylethylthio)-2,3,4,6-tetra-O-acetyl-D-mannopyranose (13b)

Prepared according to the **method A** from mannosyl fluoride α-**11** (105 mg, 0.300 mmol, 1.0 equiv) and 2-phenylethanethiol (**2c**, 3.0 equiv) at 100 °C; yield 67% (93.8 mg); α:β = 78:22 (NMR).

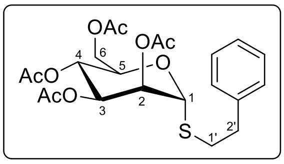

α-**13b**[19]: colorless oil; R_f = 0.62 (EtOAc/hexanes 1:1); ^{1}H NMR (500 MHz, $CDCl_3$): δ 7.32–7.27 (m, 2H, H-Ar), 7.24–7.17 (m, 3H, H-Ar), 5.34 (dd, J = 3.3, 1.5 Hz, 1H, H-C2), 5.30 (t, J = 10.0 Hz, 1H, H-C4), 5.27 (br s, 1H, H-C1), 5.25 (dd, J = 10.0, 3.3 Hz, 1H, H-C3), 4.35 (ddd, J = 10.0, 5.4, 2.1 Hz, 1H, H-C5), 4.30 (dd, J = 12.2, 5.4 Hz, 1H, H_a-C6), 4.07 (dd, J = 12.2, 2.1 Hz, 1H, H_b-C6), 2.97–2.87 (m, 3H, H_a-C1', H_2-C2'), 2.87–2.78 (m, 1H, H_b-C1'), 2.16 (s, 3H, Ac), 2.04 (s, 3H, Ac), 2.04 (s, 3H, Ac), 1.99 (s, 3H, Ac); ^{13}C NMR (126 MHz, $CDCl_3$): δ 170.72, 170.07, 169.91, 169.85, 139.93, 128.72, 128.63, 126.72, 82.70, 71.18, 69.55, 69.14, 66.43, 62.57, 36.15, 32.84, 21.05, 20.83 (2C), 20.76.

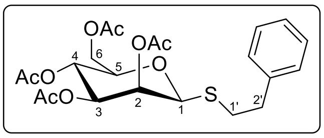

β-**13b**: white amorphous solid; R_f = 0.55 (EtOAc/hexanes 1:1); $[\alpha]_D^{24}$ –53.5 (*c* 1.05, $CHCl_3$); ^{1}H NMR (500 MHz, $CDCl_3$): δ 7.35–7.29 (m, 2H, H-Ar), 7.26–7.18 (m, 3H, H-Ar), 5.46 (dd, J = 3.6, 1.1 Hz, 1H, H-C2), 5.23 (t, J = 10.0 Hz, 1H, H-C4), 4.97 (dd, J = 10.0, 3.6 Hz, 1H, H-C3), 4.57 (d, J = 1.1 Hz, 1H, H-C1), 4.26 (dd, J = 12.2, 6.0 Hz, 1H, H_a-C6), 4.13 (dd, J = 12.2, 2.5 Hz, 1H, H_b-C6), 3.58 (ddd, J = 10.0, 6.0, 2.5 Hz, 1H, H-C5), 3.05–2.93 (m, 3H, H_a-C1', H_2-C2'), 2.93–2.85 (m, 1H, H_b-C1'), 2.18 (s, 3H, Ac), 2.05 (s, 3H, Ac), 2.04 (s, 3H, Ac), 1.97 (s, 3H, Ac); ^{13}C NMR (126 MHz, $CDCl_3$): δ 170.80, 170.34, 170.18, 169.72, 140.10, 128.76, 128.68, 126.79, 82.81, 76.70, 72.01, 70.45, 65.99, 63.01, 36.86, 33.05, 20.90, 20.83, 20.77, 20.70; HRMS (ESI) *m/z* calcd for $C_{22}H_{28}O_9SNa$ (M + Na)$^+$ 491.1346, found 491.1356.

1-O-(2-Phenylethyl)-2,3,4,6-tetra-O-acetyl-D-glucopyranose (14a)[20]

Prepared according to the **method A** from glucosyl fluoride *α*-**12** (100 mg, 0.286 mmol, 1.0 equiv) and 2-phenylethanol (**2a**, 3.0 equiv) at 100 °C; yield 43% (56.1 mg); α:β = 54:46 (NMR).

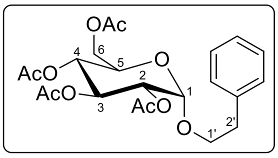

α-**14a**: white amorphous solid; R_f = 0.68 (EtOAc/hexanes 1:1); ^{1}H NMR (500 MHz, $CDCl_3$): δ 7.33–7.27 (m, 2H, H-Ar), 7.25–7.18 (m, 3H, H-Ar), 5.45 (t, J = 9.8 Hz, 1H, H-C3), 5.04 (d, J = 3.7 Hz, 1H, H-C1), 4.99 (t, J = 9.8 Hz, 1H, H-C4), 4.82 (dd, J = 9.8, 3.7 Hz, 1H, H-C2), 4.10 (dd, J = 12.4, 4.4 Hz, 1H, H_a-C6), 3.92 (dd, J = 12.4, 2.2 Hz, 1H, H_b-C6), 3.86 (dt, J = 9.7, 6.7 Hz, 1H, H_a-C1'), 3.69 (dt, J = 9.7, 6.3 Hz, 1H, H_b-C1'), 3.59 (ddd, J = 9.8, 4.4, 2.2 Hz, 1H, H-C5), 2.91 (dd, J = 6.7, 6.3 Hz, 2H, H_2-C2'), 2.06 (s, 3H, Ac), 2.01 (s, 3H, Ac), 2.01 (s, 3H, Ac), 1.99 (s, 3H, Ac); ^{13}C NMR (126 MHz, $CDCl_3$): δ 170.78, 170.32, 170.26, 169.71, 138.75, 129.14, 128.57, 126.53, 95.51, 70.94, 70.28, 69.07, 68.51, 67.23, 61.84, 35.92, 20.85 (2C), 20.78, 20.74.

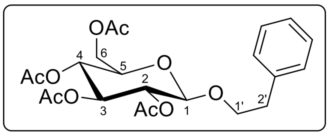

β-**14a**: colorless oil; R_f = 0.62 (EtOAc/hexanes 1:1); ^{1}H NMR (500 MHz, $CDCl_3$): δ 7.30–7.24 (m, 2H, H-Ar), 7.22–7.17 (m, 3H, H-Ar), 5.17 (t, J = 9.5 Hz, 1H, H-C3), 5.08 (t, J = 9.5 Hz, 1H, H-C4), 4.99 (dd, J = 9.5, 8.0 Hz, 1H, H-C2), 4.48 (d, J = 8.0 Hz, 1H, H-C1), 4.26 (dd, J = 12.4, 5.0 Hz, 1H, H_a-C6), 4.18–4.10 (m, 2H, H_b-C6, H_a-C1'), 3.72–3.63 (m, 2H, H-C5, H_b-C1'), 2.95–2.83 (m, 2H, H_2-C2'), 2.08 (s, 3H, Ac), 2.01 (s, 3H, Ac), 1.99 (s, 3H, Ac), 1.89 (s, 3H, Ac); ^{13}C NMR (126 MHz, $CDCl_3$): δ 170.83, 170.43, 169.54, 169.41, 138.57, 129.09, 128.48, 126.45, 100.91, 72.93, 71.94, 71.26, 70.79, 68.56, 62.08, 36.05, 20.89, 20.75, 20.73, 20.69.

1-(2-Phenylethylthio)-2,3,4,6-tetra-O-acetyl-D-glucopyranose (14b)

Prepared according to the **method A** from glucosyl fluoride α-**12** (100 mg, 0.286 mmol, 1.0 equiv) and 2-phenylethanethiol (**2c**, 3.0 equiv) at 100 °C; yield 76% (102 mg); α:β = 48:52 (NMR).

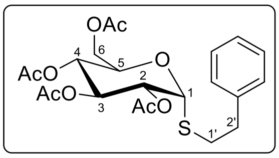

α-**14b**: colorless oil; R_f = 0.48 (EtOAc/hexanes 2:3); $[\alpha]_D^{24}$ +162.7 (*c* 1.41, $CHCl_3$); ^1H NMR (500 MHz, $CDCl_3$): δ 7.32–7.27 (m, 2H, H-Ar), 7.25–7.15 (m, 3H, H-Ar), 5.69 (d, *J* = 5.7 Hz, 1H, H-C1), 5.36 (t, *J* = 9.9 Hz, 1H, H-C3), 5.03 (t, *J* = 9.9 Hz, 1H, H-C4), 5.02 (dd, *J* = 9.9, 5.7 Hz, 1H, H-C2), 4.38 (ddd, *J* = 9.9, 5.0, 2.4 Hz, 1H, H-C5), 4.27(dd, *J* = 12.4, 5.0 Hz, 1H, H_a-C6), 4.04 (dd, *J* = 12.4, 2.4 Hz, 1H, H_b-C6), 2.95–2.80 (m, 3H, H_a-C1', H_2-C2'), 2.78–2.71 (m, 1H, H_b-C1'), 2.05 (s, 3H, Ac), 2.03 (s, 3H, Ac), 2.03 (s, 3H, Ac), 2.01 (s, 3H, Ac); ^{13}C NMR (126 MHz, $CDCl_3$): δ 170.73, 170.07, 170.04, 169.77, 140.07, 128.67, 128.61, 126.68, 81.98, 70.84, 70.60, 68.70, 67.73, 62.11, 35.93, 31.50, 20.88, 20.81 (2C), 20.75; HRMS (ESI) *m/z* calcd for $C_{22}H_{28}O_9S$ $(M + Na)^+$ 491.1346, found 491.1350.

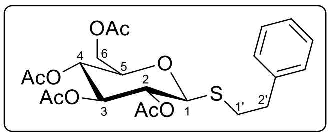

β-**14b**[21]: white amorphous solid; R_f = 0.41 (EtOAc/hexanes 2:3); ^1H NMR (500 MHz, $CDCl_3$): δ 7.32–7.27 (m, 2H, H-Ar), 7.25–7.18 (m, 3H, H-Ar), 5.20 (t, *J* = 9.9 Hz, 1H, H-C3), 5.07 (t, *J* = 9.9 Hz, 1H, H-C4), 5.04 (t, *J* = 9.9 Hz, 1H, H-C2), 4.44 (d, *J* = 9.9 Hz, 1H, H-C1), 4.24 (dd, *J* = 12.4, 5.0 Hz, 1H, H_a-C6), 4.14 (dd, *J* = 12.4, 2.3 Hz, 1H, H_b-C6), 3.66 (ddd, *J* = 9.9, 5.0, 2.3 Hz, 1H, H-C5), 3.02–2.94 (m, 1H, H_a-C1'), 2.94–2.87 (m, 3H, H_b-C1', H_2-C2'), 2.06 (s, 3H, Ac), 2.04 (s, 3H, Ac), 2.02 (s, 3H, Ac), 2.00 (s, 3H, Ac); ^{13}C NMR (126 MHz, $CDCl_3$): δ 170.78, 170.32, 169.54, 169.53, 140.17, 128.67, 128.64, 126.66, 83.63, 76.07, 73.98, 69.96, 68.44, 62.30, 36.44, 31.37, 20.87, 20.83, 20.75, 20.72.

1-O-(2-Phenylethyl)-2,3,4,6-tetra-O-benzyl-D-mannopyranose (17a)

Prepared according to the **method A** from mannosyl fluoride α-**15** (100 mg, 0.184 mmol, 1.0 equiv) and 2-phenylethanol (**2a**, 2.0 equiv) at 30 °C; yield 72% (85.1 mg); α:β = 98:2 (HPLC; $t_R(\alpha)$ = 6.349 min; $t_R(\beta)$ = 7.020 min).

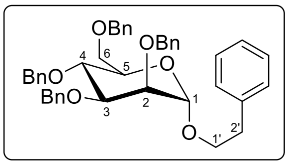

α-**17a**[22]: colorless oil; R_f = 0.53 (EtOAc/hexanes 1:4); ^1H NMR (500 MHz, $CDCl_3$): δ 7.41–7.11 (m, 25H, H-Ar), 4.88 (d, *J* = 10.8 Hz, 1H, H-CPh), 4.86 (br s, 1H, H-C1), 4.72 (d, *J* = 12.3 Hz, 1H, H-CPh), 4.68 (d, *J* = 12.3 Hz, 1H, H-CPh), 4.65 (d, *J* = 12.0 Hz, 1H, H-CPh), 4.65–4.61 (m, 2H, 2×H-CPh), 4.52 (d, *J* = 12.0 Hz, 1H, H-CPh), 4.50 (d, *J* = 10.8 Hz, 1H, H-CPh), 3.97 (t, *J* = 9.5 Hz, 1H, H-C4), 3.89 (dd, *J* = 9.5, 2.8 Hz, 1H, H-C3), 3.91–3.84 (m, 1H, H_a-C1'), 3.75 (d, *J* = 2.8 Hz, 1H, H-C2), 3.74 (dd, *J* = 10.7, 5.1 Hz, 1H, H_a-C6), 3.68 (dd, *J* = 10.7, 2.1 Hz, 1H, H_b-C6), 3.66–3.58 (m, 2H, H-C5, H_b-C1'), 2.83 (t, *J* = 7.4 Hz, 2H, H_2-C2'); ^{13}C NMR (126 MHz, $CDCl_3$): δ 138.85, 138.72, 138.70, 138.58, 138.55, 129.07, 128.50, 128.48, 128.43, 128.41, 128.39, 128.01, 127.93, 127.84 (2C), 127.68, 127.67, 127.64, 127.54, 126.40, 97.86, 80.13, 75.06, 75.05, 74.90, 73.43, 72.65, 72.24, 71.95, 69.42, 68.25, 36.21.

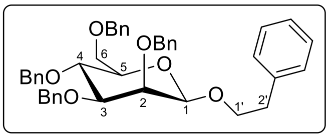

β-**17a**: isolated in a mixture with α-**17a**; R_f = 0.48 (EtOAc/hexanes 1:4); several characteristic chemical shifts are reported: ^1H NMR (500 MHz, $CDCl_3$): δ 4.34 (br s, 1H, H-C1), 3.80 (dd, *J* = 10.7, 1.9 Hz, 1H, H_a-C6), 3.45 (dd, *J* = 9.6, 3.2 Hz, 1H, H-C3), 3.43 (ddd, *J* = 9.6, 5.8, 1.9 Hz, 1H, H-C5), 2.97 (t, *J* = 7.1 Hz, 2H, H_2-C2'); ^{13}C NMR (126 MHz, $CDCl_3$): δ 101.69 (C1), 82.34 (C3), 76.07 (C5), 69.83 (C6), 36.50 (C2'); HRMS (ESI) *m/z* calcd for $C_{42}H_{48}NO_6$ $(M + NH_4)^+$ 662.3476, found 662.3462.

1-O-Cyclohexyl-2,3,4,6-tetra-O-benzyl-D-mannopyranose (17b)[23]

Prepared according to the **method A** from mannosyl fluoride α-**15** (100 mg, 0.184 mmol, 1.0 equiv) and cyclohexanol (**2b**, 2.0 equiv) at 30 °C; yield 76% (87.6 mg); α:β = 97:3 (HPLC; $t_R(\alpha)$ = 5.173 min; $t_R(\beta)$ = 5.707 min).

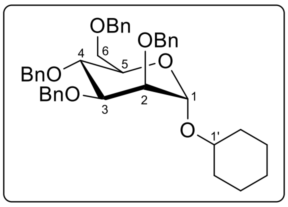

α-**17b**: colorless oil; R_f = 0.46 (EtOAc/hexanes 3:17); ^{1}H NMR (500 MHz, $CDCl_3$): δ 7.33–7.14 (m, 18H, H-Ar), 7.12–7.07 (m, 2H, H-Ar), 4.92 (d, *J* = 1.8 Hz, 1H, H-C1), 4.80 (d, *J* = 10.7 Hz, 1H, H-CPh), 4.69 (d, *J* = 12.4 Hz, 1H, H-CPh), 4.62 (d, *J* = 12.4 Hz, 1H, H-CPh), 4.59 (d, *J* = 12.1 Hz, 1H, H-CPh), 4.59–4.52 (m, 2H, 2×H-CPh), 4.45 (d, *J* = 12.1 Hz, 1H, H-CPh), 4.43 (d, *J* = 10.7 Hz, 1H, H-CPh), 3.90 (t, *J* = 9.4 Hz, 1H, H-C4), 3.85 (dd, *J* = 9.4, 3.0 Hz, 1H, H-C3), 3.78 (ddd, *J* = 9.4, 5.1, 1.9 Hz, 1H, H-C5), 3.71 (dd, *J* = 10.8, 5.1 Hz, 1H, H_a-C6), 3.65 (dd, *J* = 10.8, 1.9 Hz, 1H, H_b-C6), 3.65 (dd, *J* = 3.0, 1.8 Hz, 1H, H-C2), 3.53–3.46 (m, 1H, H-C1'), 1.79–1.71 (m, 1H), 1.71–1.63 (m, 1H), 1.63–1.53 (m, 2H), 1.45–1.36 (m, 1H), 1.29–1.04 (m, 5H); ^{13}C NMR (126 MHz, $CDCl_3$): δ 138.80, 138.67, 138.65, 138.63, 128.46 (2C), 128.43, 128.38, 128.22, 127.98, 127.82, 127.79, 127.73, 127.66, 127.63, 127.52, 95.85, 80.46, 75.49, 75.35, 75.32, 74.87, 73.41, 72.70, 72.30, 71.91, 69.55, 33.39, 31.41, 25.78, 24.17, 23.91.

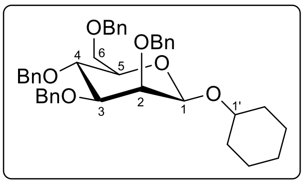

β-**17b**: isolated in a mixture with α-**17b**; R_f = 0.39 (EtOAc/hexanes 3:17); characteristic chemical shifts used for compound identification: ^{1}H NMR (500 MHz, $CDCl_3$): δ 3.43 (dd, *J* = 9.4, 3.3 Hz, 1H, H-C3), 3.37 (ddd, *J* = 9.6, 6.1, 1.7 Hz, 1H, H-C5); ^{13}C NMR (126 MHz, $CDCl_3$): δ 99.66, 82.75.

1-Dodecylthio-2,3,4,6-tetra-O-benzyl-D-mannopyranose (17c)

Prepared according to the **method A** from mannosyl fluoride α-**15** (100 mg, 0.184 mmol, 1.0 equiv) and 1-dodecanethiol (**2m**, 2.0 equiv) at 30 °C; yield 77% (103 mg); α:β = 90:10 (HPLC; $t_R(\alpha)$ = 2.877 min; $t_R(\beta)$ = 3.280 min).

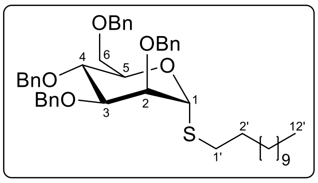

α-**17c**: colorless oil; R_f = 0.56 (EtOAc/hexanes 3:17); $[\alpha]_D^{24}$ +62.5 (*c* 1.03, $CHCl_3$); ^{1}H NMR (500 MHz, $CDCl_3$): δ 7.41–7.22 (m, 18H, H-Ar), 7.20–7.15 (m, 2H, H-Ar), 5.37 (d, *J* = 1.3 Hz, 1H, H-C1), 4.88 (d, *J* = 10.7 Hz, 1H, H-CPh), 4.73 (d, *J* = 12.5 Hz, 1H, H-CPh), 4.67 (d, *J* = 12.5 Hz, 1H, H-CPh), 4.67 (d, *J* = 12.1 Hz, 1H, H-CPh), 4.59 (d, *J* = 11.8 Hz, 1H, H-CPh), 4.56 (d, *J* = 11.8 Hz, 1H, H-CPh), 4.51 (d, *J* = 12.1 Hz, 1H, H-CPh), 4.51 (d, *J* = 10.7 Hz, 1H, H-CPh), 4.13 (ddd, *J* = 10.0, 5.0, 2.0 Hz, 1H, H-C5), 4.03 (t, *J* = 10.0 Hz, 1H, H-C4), 3.88–3.82 (m, 2H, H-C2, H-C3), 3.82 (dd, *J* = 11.0, 5.0 Hz, 1H, H_a-C6), 3.71 (dd, *J* = 11.0, 2.0 Hz, 1H, H_b-C6), 2.61 (dt, *J* = 12.8, 7.4 Hz, 1H, H_a-C1'), 2.53 (dt, *J* = 12.8, 7.4 Hz, 1H, H_b-C1'), 1.56 (quint, *J* = 7.4 Hz, 2H, H_2-C2'), 1.37–1.21 (m, 18H), 0.89 (t, *J* = 7.0 Hz, 3H, H_3-C12'); ^{13}C NMR (126 MHz, $CDCl_3$): δ 138.67, 138.54, 138.44, 138.29, 128.50, 128.49, 128.44, 128.40, 128.09, 128.02, 127.93, 127.86, 127.77, 127.76, 127.70, 127.58, 82.37, 80.55, 76.51, 75.28, 75.21, 73.43, 72.19, 72.14, 72.06, 69.31, 32.07, 31.53, 29.86, 29.82, 29.79, 29.77, 29.70, 29.51, 29.35, 29.00, 22.84, 14.27; HRMS (ESI) *m/z* calcd for $C_{46}H_{60}O_5SNa$ $(M + Na)^+$ 747.4054, found 747.4029.

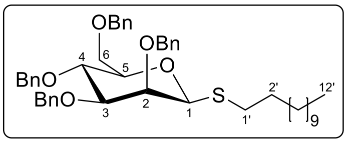

β-**17c**: isolated in a mixture with *α*-**17c**; R_f = 0.50 (EtOAc/hexanes 3:17); several characteristic chemical shifts reported: ^{1}H NMR (500 MHz, $CDCl_3$): δ 4.53[§] (br s, 1H, H-C1), 3.61 (dd, *J* = 9.6, 2.7 Hz, 1H, H-C3), 3.49 (ddd, *J* = 9.6, 6.1, 1.8 Hz, 1H, H-C5); ^{13}C NMR (126 MHz, $CDCl_3$): δ 84.85 (C1), 84.61 (C3), 80.47 (C5); HRMS (ESI) *m/z* calcd for $C_{46}H_{60}O_5SNa$ $(M + Na)^+$ 747.4054, found 747.4041.

[§]Chemical shift determined from ^{1}H-^{13}C HSQC spectrum.

1-Cyclohexylthio-2,3,4,6-tetra-O-benzyl-D-mannopyranose (17d)

Prepared according to the **method A** from mannosyl fluoride α-**15** (100 mg, 0.184 mmol, 1.0 equiv) and cyclohexanethiol (**2o**, 2.0 equiv) at 30 °C; yield 76% (89.0 mg); α:β = 91:9 (HPLC; $t_R(\alpha)$ = 3.560 min; $t_R(\beta)$ = 4.151 min).

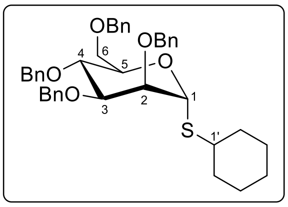

α-**17d**: colorless oil; R_f = 0.47 (EtOAc/hexanes 3:17); $[\alpha]_D^{24}$ +75.9 (*c* 1.03, $CHCl_3$); ^{1}H NMR (500 MHz, $CDCl_3$): *δ* 7.42–7.23 (m, 18H, H-Ar), 7.22–7.16 (m, 2H, H-Ar), 5.48 (d, *J* = 1.5 Hz, 1H, H-C1), 4.89 (d, *J* = 10.7 Hz, 1H, H-CPh), 4.74 (d, *J* = 12.2 Hz, 1H, H-CPh), 4.68 (d, *J* = 12.2 Hz, 1H, H-CPh), 4.68 (d, *J* = 12.2 Hz, 1H, H-CPh), 4.61–4.55 (m, 2H, 2×H-CPh), 4.51 (d, *J* = 10.7 Hz, 1H, H-CPh), 4.51 (d, *J* = 12.2 Hz, 1H, H-CPh), 4.17 (ddd, *J* = 9.5, 5.0, 1.7 Hz, 1H, H-C5), 4.02 (t, *J* = 9.5 Hz, 1H, H-C4), 3.87 (dd, *J* = 3.1, 1.5 Hz, 1H, H-C2), 3.83 (dd, *J* = 9.5, 3.1 Hz, 1H, H-C3), 3.83 (dd, *J* = 10.9, 5.0 Hz, 1H, H_a-C6), 3.72 (dd, *J* = 10.9, 1.7 Hz, 1H, H_b-C6), 2.88–2.74 (m, 1H, H-C1'), 2.01–1.89 (m, 2H), 1.78–1.64 (m, 2H), 1.63–1.51 (m, 1H), 1.41–1.18 (m, 5H); ^{13}C NMR (126 MHz, $CDCl_3$): *δ* 138.71, 138.58, 138.46, 138.34, 128.49, 128.48, 128.42, 128.37, 128.06, 128.02, 127.89, 127.81, 127.74, 127.73, 127.67, 127.54, 81.31, 80.68, 76.74, 75.27, 75.25, 73.35, 72.12, 72.05 (2C), 69.39, 44.39, 34.16, 33.94, 26.15, 26.03, 25.81; HRMS (ESI) *m/z* calcd for $C_{40}H_{50}NO_5S$ $(M + NH_4)^+$ 656.3404, found 656.3397.

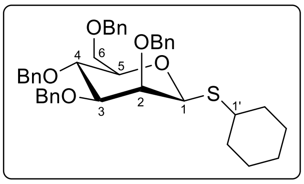

β-**17d**: isolated in a mixture with *α*-**17d**; R_f = 0.42 (EtOAc/hexanes 3:17); several characteristic chemical shifts are reported: ^{1}H NMR (500 MHz, $CDCl_3$): *δ* 4.62 (br s, 1H, H-C1), 3.61 (dd, *J* = 9.5, 2.8 Hz, 1H, H-C3), 3.50 (ddd, *J* = 9.5, 6.6, 2.0 Hz, 1H, H-C5), 2.97–2.86 (m, 1H, H-C1'); ^{13}C NMR (126 MHz, $CDCl_3$): *δ* 84.68 (C3), 83.53 (C1), 80.32 (C5); HRMS (ESI) *m/z* calcd for $C_{40}H_{46}O_5SNa$ $(M + Na)^+$ 661.2958, found 661.2956.

1-(4-Chlorophenylthio)-2,3,4,6-tetra-O-benzyl-D-mannopyranose (17e)

Prepared according to the **method A** from mannosyl fluoride α-**15** (100 mg, 0.184 mmol, 1.0 equiv) and 4-chlorothiophenol (**2p**, 3.0 equiv) at 30 °C; yield 67% (82.6 mg); α:β = 87:13 (HPLC; $t_R(\alpha)$ = 3.556 min; $t_R(\beta)$ = 3.940 min).

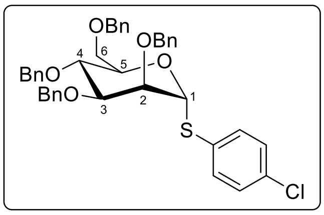

α-**17e**: colorless oil; R_f = 0.44 (EtOAc/hexanes 3:17); $[\alpha]_D^{24}$ +88.7 (*c* 1.02, $CHCl_3$); ^{1}H NMR (500 MHz, $CDCl_3$): *δ* 7.40–7.25 (m, 20H, H-Ar), 7.23–7.19 (m, 2H, H-Ar), 7.19–7.15 (m, 2H, H-Ar), 5.53 (d, *J* = 1.7 Hz, 1H, H-C1), 4.90 (d, *J* = 10.8 Hz, 1H, H-CPh), 4.72 (d, *J* = 12.3 Hz, 1H, H-CPh), 4.64 (d, *J* = 12.3 Hz, 1H, H-CPh), 4.63 (d, *J* = 12.0 Hz, 1H, H-CPh), 4.63 (d, *J* = 11.8 Hz, 1H, H-CPh), 4.60 (d, *J* = 11.8 Hz, 1H, H-CPh), 4.53 (d, *J* = 10.8 Hz, 1H, H-CPh), 4.49 (d, *J* = 12.0 Hz, 1H, H-CPh), 4.26 (ddd, *J* = 9.7, 5.3, 1.7 Hz, 1H, H-C5), 4.03 (t, *J* = 9.7 Hz, 1H, H-C4), 3.96 (dd, *J* = 3.0, 1.7 Hz, 1H, H-C2), 3.84 (dd, *J* = 9.7, 3.0 Hz, 1H, H-C3), 3.82 (dd, *J* = 10.8, 5.3 Hz, 1H, H_a-C6), 3.75 (dd, *J* = 10.8, 1.7 Hz, 1H, H_b-C6); ^{13}C NMR (126 MHz, $CDCl_3$): *δ* 138.49, 138.41, 138.24, 137.96, 133.81, 133.24 (2C), 132.80, 129.24, 128.58, 128.54, 128.51, 128.45, 128.12, 128.07, 128.00, 127.90 (2C), 127.82, 127.68, 85.95, 80.20, 76.28, 75.34, 75.14, 73.47, 72.93, 72.34, 72.20, 69.38; HRMS (ESI) *m/z* calcd for $C_{40}H_{43}ClNO_5S$ $(M + NH_4)^+$ 684.2545, found 684.2530.

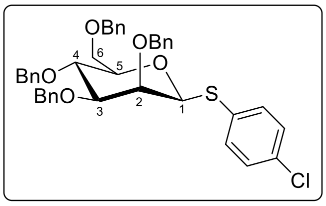

β-**17e**: isolated in a mixture with *α*-**17e**; R_f = 0.38 (EtOAc/hexanes 3:17); several characteristic chemical shifts are reported: ^{1}H NMR (500 MHz, $CDCl_3$): *δ* 4.72§ (br s, 1H, H-C1), 4.13 (d, *J* = 3.0 Hz, 1H, H-C2), 3.64 (dd, *J* = 9.5, 3.0 Hz, 1H, H-C3), 3.52 (ddd, *J* = 9.5, 6.7, 1.9 Hz, 1H, H-C5); ^{13}C NMR (126 MHz, $CDCl_3$): *δ* 87.62 (C1), 84.49 (C3), 80.23 (C5), 77.51§ (C2); HRMS (ESI) *m/z* calcd for $C_{40}H_{43}ClNO_5S$ $(M + NH_4)^+$ 684.2545, found 684.2523.

1-O-Methyl-2,3,4-tri-O-benzyl-6-O-(2,3,4,6-tetra-O-benzyl-α-D-mannopyranosyl)-α-D-glucopyranose (17f). [24,25]

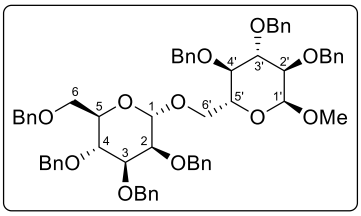

Prepared according to the **method A** from mannosyl fluoride α-**15** (100 mg, 0.184 mmol, 1.0 equiv) and 1-*O*-methyl 2,3,4-tri-*O*-benzyl-*α*-D-glucopyranose (α-**5**, 1.2 equiv); yield 25% (44.5 mg); white amorphous solid; R_f = 0.48 (EtOAc/hexanes 3:7); ^{1}H NMR (500 MHz, $CDCl_3$): *δ* 7.40–7.19 (m, 33H, H-Ar), 7.17–7.13 (m, 2H, H-Ar), 4.99 (d, *J* = 10.8 Hz, 1H, H-CPh), 4.97 (d, *J* = 1.7 Hz, 1H, H-C1), 4.89 (d, *J* = 10.3 Hz, 1H, H-CPh), 4.86 (d, *J* = 10.3 Hz, 1H, H-CPh), 4.80 (d, *J* = 10.8 Hz, 1H, H-CPh), 4.79 (d, *J* = 12.3 Hz, 1H, H-CPh), 4.74 (d, *J* = 12.4 Hz, 1H, H-CPh), 4.69 (d, *J* = 12.4 Hz, 1H, H-CPh), 4.69 (d, *J* = 12.3 Hz, 1H, H-CPh), 4.64 (d, *J* = 12.0 Hz, 1H, H-CPh), 4.62 (d, *J* = 12.0 Hz, 1H, H-CPh), 4.61 (d, *J* = 12.0 Hz, 1H, H-CPh), 4.57 (d, *J* = 3.5 Hz, 1H, H-C1'), 4.50 (d, *J* = 10.8 Hz, 1H, H-CPh), 4.49 (d, *J* = 10.8 Hz, 1H, H-CPh), 4.45 (d, *J* = 12.0 Hz, 1H, H-CPh), 4.00 (t, *J* = 9.5 Hz, 1H, H-C4), 3.98 (t, *J* = 9.3 Hz. 1H, H-C3'), 3.86 (dd, *J* = 9.5, 3.3 Hz, 1H, H-C3), 3.84 (dd, *J* = 11.8, 4.4 Hz, 1H, H_a-C6), 3.79 (dd, *J* = 3.3, 1.7 Hz, 1H, H-C2), 3.75–3.65 (m, 3H, H-C5, H-C5', H_a-C6'), 3.64–3.58 (m, 2H, H_b-C6, H_b-C6'), 3.46 (dd, *J* = 9.3, 3.5 Hz, 1H, H-C2'), 3.40 (t, *J* = 9.3 Hz, 1H, H-C4'), 3.31 (s, 3H, Me); ^{13}C NMR (126 MHz, $CDCl_3$): *δ* 138.80, 138.78, 138.60, 138.55, 138.50, 138.32, 138.27, 128.63, 128.55, 128.50 (2C), 128.41, 128.37 (2C), 128.22, 128.16, 128.14, 128.08, 127.99, 127.90, 127.86, 127.80, 127.76, 127.74, 127.70, 127.64, 127.59, 127.53, 98.38, 97.95, 82.27, 80.12, 79.70, 77.76, 75.94, 75.15, 75.07, 74.99, 74.77, 73.40 (2C), 72.57, 72.13, 72.06, 69.94, 69.24, 65.93, 55.21.

1-O-(2-Phenylethyl)-2,3,4,6-tetra-O-benzyl-D-glucopyranose (18a) [26]

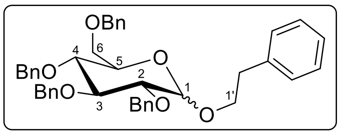

Prepared according to the **method A** from (a) α-**16** (100 mg, 0.184 mmol, 1.0 equiv) and 2-phenylethanol (**2a**, 1.2 equiv) at 30 °C; yield 61% (72.8 mg); α:β = 71:29 (HPLC); (b) *β*-**16** (92.5 mg, 0.171 mmol, 1.0 equiv) and 2-phenylethanol (**2a**, 1.2 equiv) at 60 °C; yield 74% (81.8 mg); α:β = 74:26 (HPLC); t_R(*α*) = 8.026 min; t_R(*β*) = 7.413 min; anomeric mixture was isolated as a colorless oil; R_f = 0.48 (EtOAc/hexanes 1:4); ^{1}H NMR (500 MHz, $CDCl_3$): *δ* (*α*) 4.77 (d, *J* = 3.6 Hz, 1H, H-C1), 4.02–3.95 (m, 1H, H-C3), 3.82 (dt, *J* = 9.8, 7.5 Hz, 1H, H_a-C1'), 3.71–3.66 (m, 1H, H_b-C1'), 3.66–3.57 (m, 3H, H-C4, H-C5, H_a-C6), 3.55 (dd, *J* = 9.6, 3.6 Hz, 1H, H-C2), 3.55–3.51 (m, 1H, H_b-C6); (*β*) 4.41 (d, *J* = 7.8 Hz, 1H, H-C1), 4.21 (dt, *J* = 9.5, 6.7 Hz, 1H, H_a-C1'), 3.78–3.71 (m, 2H, H_a-C6, H_b-C1'), 3.71–3.66 (m, 1H, H_b-C6), 3.66–3.57 (m, 2H, H-C3, H-C4), 3.48–3.41 (m, 1H, H-C5), 3.44 (t, *J* = 8.4 Hz, 1H, H-C2); ^{13}C NMR (126 MHz, $CDCl_3$): *δ* (*α*) 96.97, 82.11, 80.14, 77.79, 70.30, 68.58; (*β*) 103.76, 84.79, 82.36, 77.99, 74.98, 69.07.

1-O-Cyclohexyl-2,3,4,6-tetra-O-benzyl-D-glucopyranose (18b) [26]

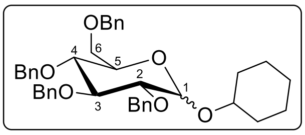

Prepared according to the **method A** from (a) α-**16** (100 mg, 0.184 mmol, 1.0 equiv) and cyclohexanol (**2b**, 2.0 equiv) at 30 °C; yield 68% (78.0 mg); α:β = 74:26 (HPLC); (b) β-**16** (50.5 mg, 0.093 mmol, 1.0 equiv) and cyclohexanol (**2b**, 2.0 equiv) at 60 °C; yield 68% (39.6 mg); α:β = 69:31 (HPLC); t_R(*α*) = 5.513 min; t_R(*β*) = 4.620 min; anomeric mixture was isolated as a colorless oil; R_f = 0.58 (EtOAc/hexanes 1:4); ^{1}H NMR (500 MHz, $CDCl_3$): *δ* (*α*) 4.97 (d, *J* = 3.7 Hz, 1H, H-C1), 4.01 (t, *J* = 9.7 Hz, 1H, H-C3), 3.89 (ddd, *J* = 9.7, 3.6, 2.0 Hz, 1H, H-C5), 3.78–3.69 (m, 1H, H_a-C6), 3.69–3.61 (m, 2H, H-C4, H_b-C6), 3.60–3.52 (m, 1H, H-C2); (*β*) 4.52 (d, *J* = 8.1 Hz, 1H, H-C1), 3.78–3.69 (m, 1H, H_a-C6), 3.69–3.61 (m, 2H, H-C3, H_b-C6), 3.60–3.52 (m, 1H, H-C4), 3.50–3.43 (m, 1H, H-C5), 3.46 (t, *J* = 8.4 Hz, 1H, H-C2); ^{13}C NMR (126 MHz, $CDCl_3$): *δ* (*α*) 94.83, 82.25, 80.14, 78.05, 70.21, 68.76; (*β*) 102.10, 85.00, 82.44, 78.18, 74.97, 69.34.

*1-O-Phenyl-2,3,4,6-tetra-O-benzyl-D-glucopyranose (**18c**).*[27]

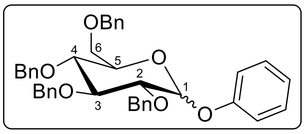

Prepared according to the **method A** from *β*-**16** (47.8 mg, 0.088 mmol, 1.0 equiv) and phenol (**2l**, 3.0 equiv) at 60 °C; yield 60% (32.6 mg); α:β = 74:26 (HPLC); $t_R(\alpha)$ = 5.372 min; $t_R(\beta)$ = 4.572 min; anomeric mixture was isolated as a colorless oil; R_f = 0.46 (EtOAc/hexanes 1:4); ^1H NMR (500 MHz, $CDCl_3$): δ (α) 5.49 (d, J = 3.5 Hz, 1H, H-C1), 4.22 (t, J = 9.5 Hz, 1H, H-C3), 3.93–3.87 (m, 1H, H-C5), 3.80 (t, J = 9.5 Hz, 1H, H-C4), 3.78–3.67 (m, 2H, H-C2, H_a-C6), 3.61–3.56 (m, 1H, H_b-C6); (β) 5.03 (d, J = 6.8 Hz, 1H, H-C1), 3.84–3.78 (m, 1H, H_a-C6), 3.78–3.67 (m, 4H, H-C2, H-C3, H-C4, H_b-C6), 3.66–3.61 (m, 1H, H-C5); ^{13}C NMR (126 MHz, $CDCl_3$): δ (α) 95.58, 82.14, 79.87, 77.54, 70.93, 68.37; (β) 101.82, 84.82, 82.16, 77.85, 75.20, 69.00.

1-Dodecylthio-2,3,4,6-tetra-O-benzyl-D-glucopyranose (18d)

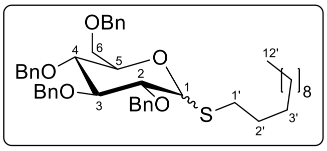

Prepared according to the **method A** from (a) α-**16** (100 mg, 0.184 mmol, 1.0 equiv) and 1-dodecanethiol (**2m**, 2.0 equiv) at 30 °C; yield 80% (107 mg); α:β = 74:26 (HPLC); (b) β-**16** (40.8 mg, 0.075 mmol, 1.0 equiv) and 1-dodecanethiol (**2m**, 2.0 equiv) at 60 °C; yield 82% (44.3 mg); α:β = 72:28 (HPLC); $t_R(\alpha)$ = 7.710 min; $t_R(\beta)$ = 6.481 min; anomeric mixture was isolated as a colorless oil; R_f = 0.49 (EtOAc/hexanes 3:17); ^1H NMR (500 MHz, $CDCl_3$): δ 7.42–7.38 (m, H-Ar), 7.37–7.27 (m, H-Ar), 7.21–7.13 (m, H-Ar), 5.39 (d, J = 5.1 Hz, 1H, α-H-C1), 5.00–4.91 (m, 1H-α, 2H-β, H-CPh), 4.89–4.81 (m, 1H-α, 2H-β, H-CPh), 4.80–4.72 (m, 2H-α, 1H-β, H-CPh), 4.67 (d, J = 11.9 Hz, 1H, α-H-CPh), 4.65–4.61 (m, 1H-α, 1H-β, H-CPh), 4.60–4.55 (m, 2H-β, H-CPh), 4.49 (d, J = 10.8 Hz, 1H, α-H-CPh), 4.47 (d, J = 12.0 Hz, 1H, α-H-CPh), 4.45 (d, J = 9.6 Hz, 1H, β-H-C1), 4.21 (ddd, J = 9.8, 3.8, 2.1 Hz, 1H, α-H-C5), 3.88 (t, J = 9.6 Hz, 1H, α-H-C3), 3.84 (dd, J = 9.6, 5.1 Hz, 1H, α-H-C2), 3.78–3.74 (m, 1H, β-H_a-C6), 3.78 (dd, J = 10.7, 3.8 Hz, 1H, α-H_a-C6), 3.73–3.68 (m, 2H, β-H-C3, β-H_b-C6), 3.68–3.60 (m, 3H, α-H-C4, α-H_b-C6, β-H-C4), 3.48 (ddd, J = 9.5, 4.8, 1.9 Hz, 1H, β-H-C5), 3.46 (t, J = 9.5 Hz, 1H, β-H-C2), 2.83–2.67 (m, 2H, β-H_2-C1'), 2.58 (dt, J = 12.9, 7.3 Hz, 1H, α-H_a-C1'), 2.49 (dt, J = 12.9, 7.3 Hz, 1H, α-H_b-C1'), 1.68 (quint, J = 7.5 Hz, 2H, β-H_2-C2'), 1.61 (quint, J = 7.3 Hz, 2H, α-H_2-C2'), 1.46–1.36 (m, H_2-C3'), 1.35–1.21 (m, H_2-C(4'-11')), 0.97–0.83 (m, H_3-C12'); ^{13}C NMR (126 MHz, $CDCl_3$): δ 138.90, 138.66, 138.45, 138.38, 138.19, 138.14, 138.06, 138.04, 128.56, 128.54, 128.51, 128.47, 128.24, 128.14, 128.10, 127.98, 127.96, 127.90, 127.82, 127.79, 127.77, 127.69, 86.80 (β), 85.39 (β), 83.60 (α), 82.73 (α), 81.97 (β), 79.73 (α), 79.27 (β), 78.12 (β), 77.65 (α), 75.88, 75.84, 75.63, 75.15, 73.57, 72.51, 70.57 (α), 69.27 (β), 68.72 (α), 32.06, 31.03, 30.05, 29.92, 29.81, 29.78, 29.77, 29.71, 29.69, 29.49, 29.38, 29.08, 22.83, 14.26; HRMS (ESI) *m/z* calcd for $C_{46}H_{64}NO_5S$ $(M + NH_4)^+$ 742.4500, found 742.4480.

1-Cyclohexylthio-2,3,4,6-tetra-O-benzyl-D-glucopyranose (18e)[28]

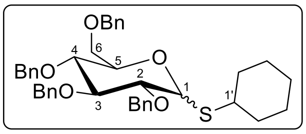

Prepared according to the **method A** from (a) α-**16** (100 mg, 0.184 mmol, 1.0 equiv) and cyclohexanethiol (**2o**, 2.0 equiv) at 30 °C; yield 69% (80.6 mg); α:β = 74:26 (HPLC); (b) β-**16** (97.2 mg, 0.179 mmol, 1.0 equiv) and cyclohexanethiol (**2o**, 2.0 equiv) at 60 °C; yield 79% (90.1 mg); α:β = 72:28 (HPLC); $t_R(\alpha)$ = 4.493 min; $t_R(\beta)$ = 3.930 min); anomeric mixture was isolated as a white amorphous solid; R_f = 0.56 (EtOAc/hexanes 1:4); ^1H NMR (500 MHz, $CDCl_3$): δ (α) 5.46 (d, J = 4.9 Hz, 1H, H-C1), 4.26 (ddd, J = 9.8, 3.9, 1.7 Hz, 1H, H-C5), 3.89–3.80 (m, 2H, H-C2, H-C3), 3.78 (dd, J = 10.5, 3.9 Hz, 1H, H_a-C6), 3.67–3.42 (m, 2H, H-C4, H_b-C6), 2.77 (tt, J = 10.3, 3.9 Hz, 1H, H-C1'); (β) 4.55 (d, J = 9.7 Hz, 1H, H-C1), 3.79–3.74 (m, 1H, H_a-C6), 3.72–3.63 (m, 2H, H-C3, H_b-C6), 3.59 (t, J = 9.5 Hz, 1H, H-C4), 3.48 (ddd, J = 9.5, 5.4, 1.8 Hz, 1H, H-C5), 3.44 (t, J = 9.5 Hz, 1H, H-C2), 3.01 (tt, J = 10.6, 3.9 Hz, 1H, H-C1'); ^{13}C NMR (126 MHz, $CDCl_3$): δ (α) 82.80, 82.66, 79.73, 77.71, 70.54, 68.76; (β) 86.88, 84.57, 82.21, 79.14, 78.20, 69.46.

1-(4-Chlorophenylthio)-2,3,4,6-tetra-O-benzyl-D-glucopyranose (18f)

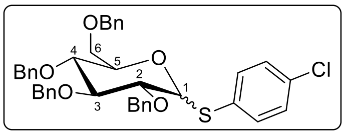

Prepared according to the **method A** from (a) α-**16** (100 mg, 0.184 mmol, 1.0 equiv) and 4-chlorothiophenol (**2p**, 3.0 equiv) at 30 °C; yield 60% (73.2 mg); α:β = 73:27 (HPLC); (b) β-**16** (57.9 mg, 0.107 mmol, 1.0 equiv) and 4-chlorothiophenol (**2p**, 3.0 equiv) at 60 °C; yield 66% (46.9 mg); α:β = 73:27 (HPLC); $t_R(\alpha)$ = 5.471 min; $t_R(\beta)$ = 4.586 min; anomeric mixture was isolated as a white solid; R_f = 0.38 (EtOAc/hexanes 3:17); ^{1}H NMR (500 MHz, $CDCl_3$): δ 7.55–7.50 (m, H-Ar), 7.44–7.26 (m, H-Ar), 7.25–7.15 (m, H-Ar), 5.59 (d, *J* = 4.5 Hz, 1H, α-H-C1), 5.01 (d, *J* = 10.8 Hz, 1H, α-H-CPh), 4.91 (d, *J* = 11.2 Hz, 1H, β-H-CPh), 4.89–4.81 (m, 2H-α, 3H-β, H-CPh), 4.79–4.69 (m, 2H-α, 1H-β, H-CPh), 4.64 (d, *J* = 9.5 Hz, 1H, β-H-C1), 4.63–4.57 (m, 2H, β-H-CPh), 4.59 (d, *J* = 11.8 Hz, 1H, α-H-CPh), 4.55 (d, *J* = 11.8 Hz, 1H, β-H-CPh), 4.51 (d, *J* = 10.8 Hz, 1H, α-H-CPh), 4.43 (d, *J* = 11.8 Hz, 1H, α-H-CPh), 4.31 (ddd, *J* = 10.0, 4.1, 1.9 Hz, 1H, α-H-C5), 3.94–3.90 (m, 1H, α-H-C2), 3.90 (t, *J* = 10.0 Hz, 1H, α-H-C3), 3.82–3.65 (m, 4H, β-H-C3, β-H-C4, β-$H_{a,b}$-C6), 3.76 (dd, *J* = 10.7, 4.7 Hz, 1H, α-H_a-C6), 3.68 (t, *J* = 10.0 Hz, 1H, α-H-C4), 3.62 (dd, *J* = 10.7, 1.9 Hz, 1H, α-H_b-C6), 3.55–3.49 (m, 1H, β-H-C5), 3.50 (t, *J* = 9.5 Hz, 1H, β-H-C2); ^{13}C NMR (126 MHz, $CDCl_3$): δ 138.73, 138.45, 138.29, 138.25, 138.08, 138.03, 137.92, 137.72, 133.82, 133.50, 133.38, 133.05, 132.97, 132.24, 129.15, 128.61, 128.58, 128.53, 128.50, 128.30, 128.28, 128.13, 128.08, 128.03, 128.01, 127.99, 127.89, 127.87, 127.81, 127.80, 87.33 (β), 87.17 (α), 86.80 (β), 82.61 (α), 80.89 (β), 79.83 (α), 79.14 (β), 77.87 (β), 77.51 (α), 75.96, 75.93, 75.58, 75.28, 75.21, 73.56, 72.83, 71.37 (α), 69.09 (α), 68.68 (α); HRMS (ESI) *m/z* calcd for (α) $C_{40}H_{39}ClO_5SK$ $(M + K)^+$ 705.1838, found 705.1865 and (β) for $C_{40}H_{39}ClO_5SNa$ $(M + Na)^+$ 689.2099, found 689.2061.

1-O-(2-Phenylethyl)-3,4,6-tri-O-pivaloyl-2-deoxy-D-glucopyranose (20)

Prepared according to the **method B**; yield 91% (90.1 mg); α:β = 91:9 (NMR).

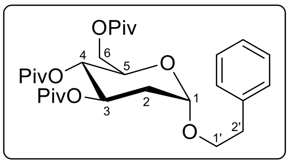

α-**20**: isolated in a mixture with β-**20**; major component (91%); R_f = 0.42 (EtOAc/hexanes 3:17); ^{1}H NMR (500 MHz, $CDCl_3$): δ 7.33–7.28 (m, 2H, H-Ar), 7.26–7.18 (m, 3H, H-Ar), 5.22 (ddd, *J* = 11.4, 9.8, 5.3 Hz, 1H, H-C3), 4.95 (t, *J* = 9.8 Hz, 1H, H-C4), 4.88 (dd, *J* = 3.7, 1.3 Hz, 1H, H-C1), 3.95–3.86 (m, 2H, $H_{a,b}$-C6), 3.83–3.75 (m, 1H, H-C5), 3.64 (dt, *J* = 9.9, 5.9 Hz, 1H, H_a-C1'), 3.36 (dt, *J* = 9.9, 3.5 Hz, 1H, H_b-C1'), 2.94–2.84 (m, 2H, H_2-C2'), 2.20 (ddd. *J* = 12.7, 5.3, 1.3 Hz, 1H, H_{eq}-C2), 1.70 (ddd, *J* = 12.7, 11.4, 3.7 Hz, 1H, H_{ax}-C2), 1.19 (s, 9H, Piv), 1.15 (s, 9H, Piv), 1.14 (s, 9H, Piv); ^{13}C NMR (126 MHz, $CDCl_3$) δ 178.24, 177.66, 176.82, 139.29, 129.27, 128.52, 126.43, 96.52, 69.01, 68.64, 68.12, 68.03, 62.41, 38.98, 38.87, 38.81, 36.23, 35.25, 27.28, 27.23, 27.22; HRMS (ESI) *m/z* calcd for $C_{29}H_{48}NO_8$ $(M + NH_4)^+$ 538.3374, found 538.3385.

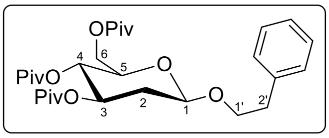

β-**20**: isolated in a mixture with α-**20**; minor component (9%); R_f = 0.42 (EtOAc/hexanes 3:17); several characteristic chemical shifts are reported: ^{1}H NMR (500 MHz, $CDCl_3$) δ 5.01–4.98 (m, 1H, H-C3), 4.56 (dd, *J* = 9.7, 1.8 Hz, 1H, H-C1), 4.20 (dd, *J* = 12.1, 1.7 Hz, 1H, H_a-C6), 4.15–4.06 (m, 2H, H-C5, H_b-C6), 2.32–2.24 (m, 1H, H_{eq}-C2); ^{13}C NMR (126 MHz, $CDCl_3$) δ 99.63, 72.51, 70.53, 70.37, 68.71, 62.72; HRMS (ESI) *m/z* calcd for $C_{29}H_{44}O_8Na$ $(M + Na)^+$ 543.2928, found 543.2910.

Side-products:

2,3,4,6-Tetra-O-pivaloyl-α-D-mannopyranose (α-4)[29]

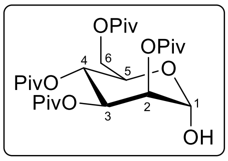

^{1}H NMR (500 MHz, $CDCl_3$): δ 5.53 (t, *J* = 10.2 Hz, 1H, H-C4), 5.46 (dd, *J* = 10.2, 3.2 Hz, 1H, H-C3), 5.28 (dd, *J* = 3.2, 1.7 Hz, 1H, H-C2), 5.19 (dd, *J* = 4.0, 1.7 Hz, 1H, H-C1), 4.29 (ddd, *J* = 10.2, 3.3, 2.0 Hz, 1H, H-C5), 4.19 (dd, *J* = 12.5, 3.3 Hz, 1H, H_a-C6), 4.15 (dd, *J* = 12.5, 2.0 Hz, 1H, H_b-C6), 3.11 (d, *J* = 4.0 Hz, 1H, OH), 1.27 (s, 9H, Piv), 1.24 (s, 9H, Piv), 1.16 (s, 9H, Piv), 1.12 (s, 9H, Piv); ^{13}C NMR (75.5 MHz, $CDCl_3$): δ 178.49, 177.43 (2C), 176.79, 92.61, 70.02, 69.25, 68.98, 65.25, 62.03, 39.07, 39.05, 38.94, 38.91, 27.27 (2C), 27.24, 27.21.

1,6-Anhydro-2,3,4-tri-O-benzyl-β-D-glucopyranose (β-6)[30]

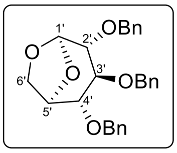

^{1}H NMR (500 MHz, $CDCl_3$) δ 7.38–7.23 (m, 15H, H-Ar), 5.47 (br s, 1H, H-C1), 4.66–4.52 (m, 5H, H-C5, 4×H-CPh), 4.47 (d, *J* = 12.2 Hz, 1H, H-CPh), 4.42 (d, *J* = 12.2 Hz, 1H, H-CPh), 3.92 (d, *J* = 7.20 Hz, 1H, H_a-C6), 3.69 (dd, *J* = 7.20, 6.2 Hz, 1H, H_b-C6), 3.61 (br s, 1H, H-C4), 3.40–3.32 (m, 2H, H-C2, H-C3); ^{13}C NMR (126 MHz, $CDCl_3$) δ 138.07, 138.02, 137.99, 128.60, 128.57, 128.57, 128.10, 127.97 (2C), 127.96 (2C), 127.87, 100.76, 76.97, 76.30, 76.22, 74.53, 72.16, 71.91, 71.32, 65.56.

1,1'-α,α-D-mannoside α,α-S14

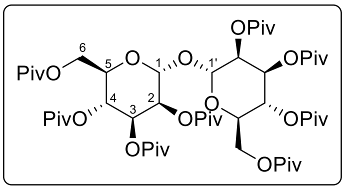

^{1}H NMR (500 MHz, $CDCl_3$): δ 5.55 (t, *J* = 10.1 Hz, 1H, H-C4), 5.35 (dd, *J* = 10.1, 3.3 Hz, 1H, H-C3), 5.32 (dd, *J* = 3.3, 1.7 Hz, 1H, H-C2), 5.09 (d, *J* = 1.7 Hz, 1H, H-C1), 4.21 (dd, *J* = 12.5, 4.1 Hz, 1H, H_a-C6), 4.11 (dd, *J* = 12.5, 1.8 Hz, 1H, H_b-C6), 4.09 (ddd, *J* = 10.1, 4.1, 1.8 Hz, 1H, H-C5), 1.26 (s, 9H, Piv), 1.21 (s, 9H, Piv), 1.19 (s, 9H, Piv), 1.13 (s, 9H, Piv); ^{13}C NMR (126 MHz, $CDCl_3$): δ 178.09, 177.24, 177.20, 176.88, 93.37, 70.04, 69.35, 69.26, 64.85, 61.86, 39.08, 39.01 (2C), 38.91, 27.27, 27.26 (2C), 27.24; HRMS (ESI) *m/z* calcd for $C_{52}H_{90}NO_{19}$ $(M + NH_4)^+$ 1032.6102, found 1032.6102.
Note: *cross peak between H-C1 and C1 was clearly observed in ^{1}H-^{13}C HMBC spectrum.*

1-O-(2-Phenylethyl)-2,4,6-tri-O-pivaloyl-α-D-mannopyranose (α-S15)

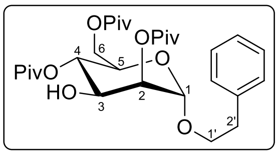

^{1}H NMR (500 MHz, $CDCl_3$): δ 7.33–7.19 (m, 5H, H-Ar), 5.05 (t, *J* = 10.0 Hz, 1H, H-C4), 5.02 (dd, *J* = 3.5, 1.6 Hz, 1H, H-C2), 4.79 (d, *J* = 1.6 Hz, 1H, H-C1), 3.99 (dd, *J* = 12.4, 4.0 Hz, 1H, H_a-C6), 3.95 (dd, *J* = 10.0, 3.5 Hz, 1H, H-C3), 3.88 (dd, *J* = 12.4, 1.5 Hz, 1H, H_b-C6), 3.81 (dt, *J* = 9.4, 7.5 Hz, 1H, H_a-C1'), 3.74 (dt, *J* = 9.4, 5.7 Hz, 1H, H_b-C1'), 3.20 (ddd, *J* = 10.0, 4.0, 1.5 Hz, 1H, H-C5), 2.95–2.85 (m, 2H, H_2-C2'), 1.24 (s, 9H, Piv), 1.20 (s, 9H, Piv), 1.18 (s, 9H, Piv); ^{13}C NMR (126 MHz, $CDCl_3$): δ 179.19, 178.18, 177.93, 139.10, 129.22, 128.57, 126.55, 96.87, 72.38, 69.54, 68.73, 68.40, 68.07, 62.15, 39.35, 39.06, 39.00, 36.10, 27.27, 27.26, 27.13; HRMS (ESI) *m/z* calcd for $C_{29}H_{44}O_9Na$ $(M + Na)^+$ 559.2878, found 559.2871.

2,3,4,6-Tetra-O-pivaloyl-α-D-glucopyranosyl fluoride (α-S16)[31]

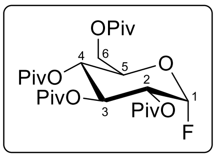

^{1}H NMR (500 MHz, $CDCl_3$): δ 5.72 (dd, *J* = 53.6, 2.7 Hz, 1H, H-C1), 5.59 (t, *J* = 9.8 Hz, 1H, H-C3 or H-C4), 5.21 (t, *J* = 9.8 Hz, 1H, H-C3 or H-C4), 4.91 (ddd, *J* = 24.2, 9.8, 2.7 Hz, 1H, H-C2), 4.23–4.17 (m, 2H, H-C5, H_a-C6), 4.12 (dd, *J* = 12.7, 4.5 Hz, 1H, H_b-C6), 1.23 (s, 9H, Piv), 1.18 (s, 9H, Piv), 1.16 (s, 9H, Piv), 1.13 (s, 9H, Piv); ^{13}C NMR (126 MHz, $CDCl_3$): δ 178.14, 177.73, 177.05, 176.48, 103.77 (d, *J* = 230 Hz), 70.70 (d, *J* = 25 Hz), 70.21 (d, *J* = 4 Hz), 69.06, 66.80, 61.18, 39.06, 38.97, 38.93, 38.89, 27.31, 27.23, 27.18, 27.12.

2,3,4,6-Tetra-O-pivaloyl-D-glucopyranose (S17)[32]

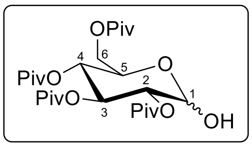

^{1}H NMR (500 MHz, $CDCl_3$): δ (α) 5.61 (t, *J* = 10.0 Hz, 1H, H-C3), 5.45 (d, *J* = 3.7 Hz, 1H, H-C1), 5.15 (t, *J* = 10.0 Hz, 1H, H-C4), 4.83 (dd, *J* = 10.0, 3.7 Hz, 1H, H-C2), 4.27 (ddd, *J* = 10.0, 4.2, 1.8 Hz, 1H, H-C5), 4.23–4.16 (m, 1H, H_a-C6), 4.07 (dd, *J* = 12.5, 4.2 Hz, 1H, H_b-C6); (β) 5.36 (t, *J* = 9.5 Hz, 1H, H-C3), 5.20–5.12 (m, 1H, H-C4), 4.89 (dd, *J* = 9.5, 8.0 Hz, 1H, H-C2), 4.72 (d, *J* = 8.0 Hz, 1H, H-C1), 4.23–4.16 (m, 1H, H_a-C6), 4.11 (dd, *J* = 12.3, 4.7 Hz, 1H, H_b-C6), 3.76 (ddd, *J* = 10.0, 4.7, 1.8 Hz, 1H, H-C5); ^{13}C NMR (126 MHz, $CDCl_3$): δ (α) 90.13, 71.57, 69.49, 67.79, 67.67, 61.73; (β) 96.15, 73.56, 72.72, 71.69, 67.85, 61.81.

3,4,6-Tri-O-pivaloyl-α-D-glucopyranose (α-S18)

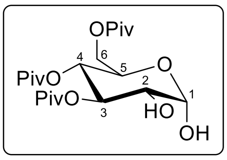

^{1}H NMR (500 MHz, $CDCl_3$): *δ* 5.31 (d, *J* = 3.8 Hz, 1H, H-C1), 5.30 (t, *J* = 9.9 Hz, 1H, H-C3), 5.10 (t, *J* = 9.9 Hz, 1H, H-C4), 4.26–4.17 (m, 2H, H-C5, H_a-C6), 4.07 (dd, *J* = 12.4, 4.4 Hz, 1H, H_b-C6), 3.66 (dd, *J* = 9.9, 3.8 Hz, 1H, H-C2), 1.22 (s, 9H, Piv), 1.19 (s, 9H, Piv), 1.16 (s, 9H, Piv); ^{13}C NMR (126 MHz, $CDCl_3$): *δ* 92.74 (C1), 73.09 (C3), 71.80 (C2), 68.25 (C5), 67.17 (C4), 61.89 (C6); HRMS (ESI) *m/z* calcd for $C_{21}H_{40}NO_9$ $(M + NH_4)^+$ 450.2698, found 450.2670.

1,3,4,6-Tetra-O-pivaloyl-α-D-glucopyranose (α-S19)[32]

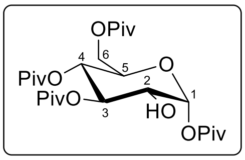

^{1}H NMR (500 MHz, $CDCl_3$): *δ* 6.20 (d, *J* = 3.8 Hz, 1H, H-C1), 5.26 (t, *J* = 9.9 Hz, 1H, H-C3), 5.13 (t, *J* = 9.9 Hz, 1H, H-C4), 4.12 (dd, *J* = 12.2, 2.0 Hz, 1H, H_a-C6), 4.08 (dd, *J* = 12.2, 5.2 Hz, 1H, H_b-C6), 3.99 (ddd, *J* = 9.9, 5.2, 2.0 Hz, 1H, H-C5), 3.92–3.83 (m, 1H, H-C2); ^{13}C NMR (126 MHz, $CDCl_3$): *δ* 91.33, 73.08, 71.04, 70.42, 66.83, 61.87.

1,2,4,6-Tetra-O-pivaloyl-β-D-mannopyranose (β-S20)[33]

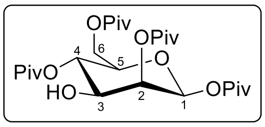

^{1}H NMR (500 MHz, $CDCl_3$): *δ* 6.05 (d, *J* = 1.8 Hz, 1H, H-C1), 5.21 (t, *J* = 10.0 Hz, 1H, H-C4), 5.10 (dd, *J* = 3.6, 1.8 Hz, 1H, H-C2), 4.22 (dd, *J* = 12.2, 4.5 Hz, 1H, H_a-C6), 4.14 (dd, *J* = 12.2, 1.7 Hz, 1H, H_b-C6), 4.12–4.05 (m, 1H, H-C3), 4.03–3.94 (m, 1H, H-C5); ^{13}C NMR (126 MHz, $CDCl_3$): *δ* 90.63, 71.09, 70.69, 69.80, 68.48, 62.05.

1-O-(2-Phenylethyl)-3,4,6-tri-O-pivaloyl-α-D-glucopyranose (α-S21)

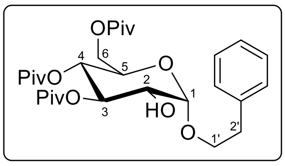

^{1}H NMR (500 MHz, $CDCl_3$): *δ* 7.36–7.18 (m, 5H, H-Ar), 5.17 (t, *J* = 9.9 Hz, 1H, H-C3), 4.95 (t, *J* = 9.9 Hz, 1H, H-C4), 4.86 (d, *J* = 3.7 Hz, 1H, H-C1), 3.97–3.84 (m, 3H, $H_{a,b}$-C6, H_a-C1'), 3.75 (dt, *J* = 9.4, 5.8 Hz, 1H, H_b-C1'), 3.62–3.54 (m, 1H, H-C2), 3.35 (ddd, *J* = 9.9, 4.6, 1.6 Hz, 1H, H-C5), 3.00–2.85 (m, 2H, H_2-C2'), 1.91 (d, *J* = 11.3 Hz, 1H, OH), 1.19 (s, 9H, Piv), 1.18 (s, 9H, Piv), 1.14 (s, 9H, Piv); ^{13}C NMR (126 MHz, $CDCl_3$): *δ* 178.70, 178.17, 176.58, 138.76, 129.17, 128.70, 126.67, 97.88, 73.01, 71.55, 68.78, 68.08, 67.12, 61.92, 39.34, 38.99, 38.84, 36.09, 27.26 (2C), 27.18; HRMS (ESI) *m/z* calcd for $C_{29}H_{44}O_9Na$ $(M + Na)^+$ 559.2878, found 559.2875.

2,3,4,6-Tetra-O-acetyl-α-D-mannopyranose (α-S22)[34]

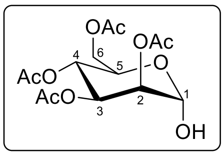

^{1}H NMR (500 MHz, $CDCl_3$): *δ* 5.41 (dd, *J* = 10.0, 3.6 Hz, 1H, H-C3), 5.29 (t, *J* = 10.0 Hz, 1H, H-C4), 5.26 (dd, *J* = 3.6, 1.8 Hz, 1H, H-C2), 5.23 (br s, 1H, H-C1), 4.29–4.20 (m, 2H, H-C5, H_a-C6), 4.18–4.10 (m, 1H, H_b-C6), 3.73 (br s, 1H, OH), 2.15 (s, 3H, Ac), 2.09 (s, 3H, Ac), 2.04 (s, 3H, Ac), 1.99 (s, 3H, Ac); ^{13}C NMR (126 MHz, $CDCl_3$): *δ* 171.01, 170.38, 170.21, 169.97, 92.30, 70.16, 68.91, 68.59, 66.30, 62.71, 21.04, 20.90, 20.84, 20.82.

1-O-(2-Phenylethyl)-2,4,6-tri-O-acetyl-α-D-mannopyranose (α-S23)

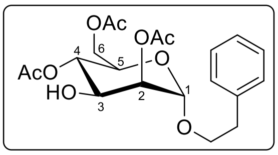

^{1}H NMR (500 MHz, $CDCl_3$): *δ* 5.04 (dd, *J* = 3.6, 1.6 Hz, 1H, H-C2), 4.98 (t, *J* = 10.0 Hz, 1H, H-C4), 4.86 (d, *J* = 1.6 Hz, 1H, H-C1), 4.14 (dd, *J* = 12.3, 5.0 Hz, 1H, H_a-C6), 4.02–3.97 (m, 1H, H-C3), 3.94 (dd, *J* = 12.3, 2.2 Hz, 1H, H_b-C6), 3.42 (ddd, *J* = 10.0, 5.0, 2.2 Hz, 1H, H-C5); ^{13}C NMR (126 MHz, $CDCl_3$): *δ* 97.01, 72.60, 69.32, 68.79, 68.07, 62.47; HRMS (ESI) *m/z* calcd for $C_{20}H_{30}NO_9$ $(M + NH_4)^+$ 428.1915, found 428.1923.

1-O-(2-Phenylethyl)-2,3,6-tri-O-acetyl-α-D-mannopyranose (α-S24)

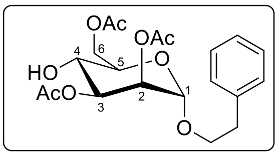

^{1}H NMR (500 MHz, $CDCl_3$): δ 5.20 (dd, J = 3.5, 1.8 Hz, 1H, H-C2), 5.13 (dd, J = 9.9, 3.5 Hz, 1H, H-C3), 4.77 (d, J = 1.8 Hz, 1H, H-C1), 4.37 (dd, J = 12.3, 4.6 Hz, 1H, H_a-C6), 4.11 (dd, J = 12.3, 2.1 Hz, 1H, H_b-C6), 3.78–3.72 (m, 1H, H-C4), 3.32 (ddd, J = 9.9, 4.6, 2.1 Hz, 1H, H-C5); ^{13}C NMR (126 MHz, $CDCl_3$): δ 97.40, 71.65, 70.92, 70.03, 65.80, 63.25; HRMS (ESI) *m/z* calcd for $C_{20}H_{26}O_9Na$ (M + Na)$^+$ 433.1469, found 433.1481.

2,3,4,6-Tetra-O-acetyl-D-glucopyranose (S25)[34]

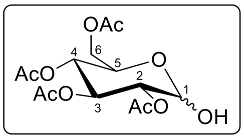

^{1}H NMR (500 MHz, $CDCl_3$): δ (α) 5.53 (t, J = 9.8 Hz, 1H, H-C3), 5.46 (t, J = 3.1 Hz, 1H, H-C1), 5.08 (t, J = 9.8 Hz, 1H, H-C4), 4.93–4.85 (m, 1H, H-C2), 4.29–4.21 (m, 2H, H-C5, H_a-C6), 4.13 (dd, J = 12.3, 1.8 Hz, 1H, H_b-C6); (β) 5.25 (t, J = 9.4 Hz, 1H, H-C3), 5.12–5.05 (m, 1H, H-C4), 4.88 (dd, J = 9.4, 8.4 Hz, 1H, H-C2), 4.74 (t, J = 8.4 Hz, 1H, H-C1), 4.29–4.21 (m, 1H, H_a-C6), 4.17–4.10 (m, 1H, H_b-C6), 3.75 (ddd, J = 10.3, 4.8, 2.2 Hz, 1H, H-C5); ^{13}C NMR (126 MHz, $CDCl_3$): δ (α) 90.32, 71.18, 69.96, 68.60, 67.41, 62.08; (β) 95.73, 73.39, 72.28, 72.23, 68.55, 62.08.

3,4,6-Tri-O-acetyl-D-glucopyranose (S26)[35]

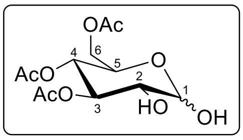

^{1}H NMR (500 MHz, $CDCl_3$): δ (α) 5.33 (d, J = 3.7 Hz, 1H, H-C1), 5.28 (t, J = 9.7 Hz, 1H, H-C3), 5.03 (t, J = 9.7 Hz, 1H, H-C4), 4.29–4.19 (m, 2H, H-C5, H_a-C6), 4.17–4.07 (m, 1H, H_b-C6), 3.72–3.66 (m, 1H, H-C2); (β) 5.11 (t, J = 9.5 Hz, 1H, H-C3), 5.03 (t, J = 9.5 Hz, 1H, H-C4), 4.71 (d, J = 7.8 Hz, 1H, H-C1), 4.29–4.19 (m, 1H, H_a-C6), 4.17–4.07 (m, 1H, H_b-C6), 3.77–3.73 (m, 1H, H-C5), 3.54 (dd, J = 9.5, 7.8 Hz, 1H, H-C2); ^{13}C NMR (126 MHz, $CDCl_3$): δ (α) 92.61, 73.43, 71.18, 68.11, 67.70, 62.24; (β) 97.02, 74.73, 73.51, 72.18, 68.46, 62.30.

1,3,4,6-Tetra-O-acetyl-α-D-glucopyranose (α-S27)[36]

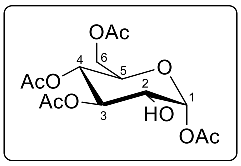

^{1}H NMR (500 MHz, $CDCl_3$): δ 6.24 (d, J = 3.9 Hz, 1H, H-C1), 5.26 (t, J = 9.9 Hz, 1H, H-C3), 5.10 (t, J = 9.9 Hz, 1H, H-C4), 4.32–4.22 (m, 2H, H-C5, H_a-C6), 4.08–4.03 (m, 1H, H_b-C6), 3.93–3.86 (m, 1H, H-C2), 2.20 (s, 3H, Ac), 2.10 (s, 3H, Ac), 2.08 (s, 3H, Ac), 2.05 (s, 3H, Ac); ^{13}C NMR (126 MHz, $CDCl_3$): δ 171.66, 170.80, 169.59, 169.25, 91.47, 73.35, 70.06, 69.82, 67.49, 61.73, 21.09, 20.98, 20.84, 20.74.

1-O-(2-Phenylethyl)-3,4,6-tri-O-acetyl-D-glucopyranose (S28)

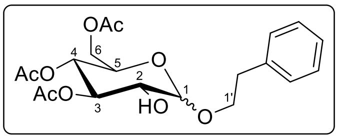

^{1}H NMR (500 MHz, $CDCl_3$): δ (α) 5.16 (t, J = 9.8 Hz, 1H, H-C3), 4.94 (t, J = 9.8 Hz, 1H, H-C4), 4.88 (d, J = 3.8 Hz, 1H, H-C1), 4.11 (dd, J = 12.4, 4.4 Hz, 1H, H_a-C6), 3.94 (dt, J = 9.7, 7.0 Hz, 1H, H_a-C1'), 3.90 (dd, J = 12.4, 2.0 Hz, 1H, H_b-C6), 3.76 (dt, J = 9.7, 6.5 Hz, 1H, H_b-C1'), 3.61 (dd, J = 9.8, 3.8 Hz, 1H, H-C2), 3.53 (ddd, J = 9.8, 4.4, 2.0 Hz, 1H, H-C5); (β) 5.09 (t, J = 9.5 Hz, 1H, H-C3), 5.02 (t, J = 9.5 Hz, 1H, H-C4), 4.33 (d, J = 7.9 Hz, 1H, H-C1), 4.27 (dd, J = 12.3, 4.8 Hz, 1H, H_a-C6), 4.18 (dt, J = 9.6, 6.6 Hz, 1H, H_a-C1'), 4.14–4.08 (m, 1H, H_b-C6), 3.80–3.72 (m, 1H, H_b-C1'), 3.66 (ddd, J = 9.5, 4.8, 2.4 Hz, 1H, H-C5), 3.54 (dd, J = 9.5, 7.9 Hz, 1H, H-C2); ^{13}C NMR (126 MHz, $CDCl_3$): δ (α) 171.22, 170.78, 169.69, 138.48, 129.04, 128.75, 126.73, 98.16, 73.54, 71.01, 69.36, 67.88, 67.74, 61.91, 36.08, 21.03, 20.85, 20.76 (β) 170.83, 170.78, 169.79, 138.27, 128.98, 128.66, 126.69, 103.12, 74.37, 72.29, 72.05, 71.23, 68.53, 62.23, 36.15, 20.93, 20.89, 20.77; HRMS (ESI) *m/z* calcd for $C_{20}H_{26}O_9Na$ (M + Na)$^+$ 433.1469, found (α) 433.1489 and (β) 433.1462.

1-(2-Phenylethylthio)-3,4,6-tri-O-acetyl-D-glucopyranose (S29)

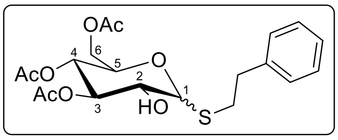

^{1}H NMR (500 MHz, CDCl$_3$): δ (α) 5.41 (d, J = 5.5 Hz, 1H, H-C1), 5.05–4.98 (m, 2H, H-C3, H-C4), 4.34–4.29 (m, 1H, H-C5), 4.29 (dd, J = 11.9, 4.8 Hz, 1H, H$_a$-C6), 4.04 (dd, J = 11.9, 1.9 Hz, 1H, H$_b$-C6), 3.99–3.91 (m, 1H, H-C2); (β) 5.08 (t, J = 9.5 Hz, 1H, H-C3), 5.05–4.98 (m, 1H, H-C4), 4.36 (d, J = 9.5 Hz, 1H, H-C1), 4.24 (dd, J = 12.1, 5.0 Hz, 1H, H$_a$-C6), 4.12 (dd, J = 12.1, 2.3 Hz, 1H, H$_b$-C6), 3.66 (ddd, J = 9.5, 5.0, 2.3 Hz, 1H, H-C5), 3.55 (t, J = 9.5 Hz, 1H, H-2); ^{13}C NMR (126 MHz, CDCl$_3$): δ 171.27, 170.89, 170.80, 170.77, 169.76, 169.75, 139.95, 139.79, 128.75, 128.73, 128.70, 128.64, 126.80, 87.73 (α), 86.66 (β), 76.09 (β), 75.88 (β), 74.17 (α), 71.22 (β), 70.68 (α), 68.76 (α), 68.34 (β), 68.01 (α), 62.48 (β), 62.13 (α), 36.74, 36.54, 33.40, 31.97, 20.97, 20.95, 20.89, 20.85, 20.78, 20.76; HRMS (ESI) *m/z* calcd for $C_{20}H_{26}O_8SNa$ (M + Na)$^+$ 449.1241, found 449.1228 and 449.1264.

1-O-Methyl-2,3,4,6-tetra-O-benzyl-α-D-mannopyranose (α-S30)[37]

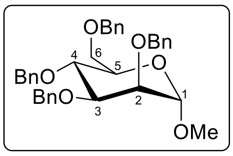

^{1}H NMR (500 MHz, CDCl$_3$): δ 7.42–7.23 (m, 18H, H-Ar), 7.20–7.16 (m, 2H, H-Ar), 4.89 (d, J = 10.9 Hz, 1H, H-CPh), 4.79 (d, J = 1.9 Hz, 1H, H-C1), 4.76 (d, J = 12.4 Hz, 1H, H-CPh), 4.73 (d, J = 12.4 Hz, 1H, H-CPh), 4.68 (d, J =12.0 Hz, 1H, H-CPh), 4.65–4.60 (br s, 2H, 2×H-CPh), 4.57 (d, J = 12.0 Hz, 1H, H-CPh), 4.52 (d, J = 10.9 Hz, 1H, H-CPh), 3.99 (t, J = 9.4 Hz, 1H, H-C4), 3.90 (dd, J = 9.4, 3.0 Hz, 1H, H-C3), 3.82–3.73 (m, 4H, H-C2, H-C4, H$_{a,b}$-C6), 3.34 (s, 3H, Me); ^{13}C NMR (126 MHz, CDCl$_3$): δ 138.68, 138.65, 138.56, 138.50, 128.47, 128.45, 128.42 (2C), 128.06, 127.95, 127.88, 127.73, 127.70, 127.67, 127.65, 127.58, 99.10, 80.37, 75.20, 75.07, 74.69, 73.50, 72.72, 72.24, 71.84, 69.45, 54.87.

1,2,3,4,6-Penta-O-benzyl-α-D-mannopyranose (α-S31)[38]

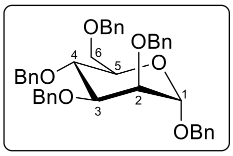

^{1}H NMR (500 MHz, CDCl$_3$): δ 7.40–7.14 (m, 20H, H-Ar), 4.98 (d, J = 1.8 Hz, 1H, H-C1), 4.88 (d, J = 10.9 Hz, 1H, H-CPh), 4.76–4.65 (m, 4H, 4×H-CPh), 4.64–4.60 (m, 2H, 2×H-CPh), 4.57 (d, J = 12.5 Hz, 1H, H-CPh), 4.52 (d, J = 10.9 Hz, 1H, H-CPh), 4.46 (d, J = 12.1 Hz, 1H, H-CPh), 4.02 (t, J = 9.5 Hz, 1H, H-C4), 4.00–3.92 (m, 1H, H-C3), 3.87–3.70 (m, 4H, H-C2, H-C5, H$_{a,b}$-C6); ^{13}C NMR (126 MHz, CDCl$_3$): δ 138.67, 138.60, 138.57, 138.43, 137.49, 128.54, 128.48, 128.46, 128.45, 128.43, 128.17, 127.98, 127.90, 127.88, 127.80, 127.74, 127.71, 127.67, 127.60, 97.32, 80.39, 75.32, 75.13, 74.80, 73.52, 72.70, 72.34, 72.22, 69.41, 69.08.

2,3,4,6-Tetra-O-benzyl-D-glucopyranose (S32)[39]

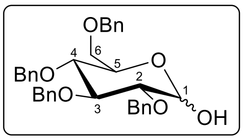

^{1}H NMR (500 MHz, CDCl$_3$): δ (α) 5.23 (d, J = 3.6 Hz, 1H, H-C1), 4.04 (ddd, J = 9.9, 4.0, 2.0 Hz, 1H, H-C5), 3.97 (t, J = 9.3 Hz, 1H, H-C3), 3.71 (dd, J = 10.6, 4.0 Hz, 1H, H$_a$-C6), 3.65 (dd, J = 10.6, 2.0 Hz, 1H, H$_b$-C6), 3.63 (dd, J = 9.9, 9.3 Hz, 1H, H-C4), 3.59 (dd, J = 9.3, 3.6 Hz, 1H, H-C2); (β) 4.72 (d, J = 7.8 Hz, 1H, H-C1), 3.74–3.69 (m, 1H, H$_a$-C6), 3.68–3.50 (m, 4H, H-C3, H-C4, H-C5, H$_b$-C6), 3.40 (dd, J = 8.8, 7.8 Hz, 1H, H-C2); ^{13}C NMR (126 MHz, CDCl$_3$): δ (α) 91.48, 81.89, 80.13, 77.80, 70.48, 68.70; (β) 97.63, 84.71, 83.26, 77.93, 74.92, 69.03.

1,2,3,4,6-Penta-O-benzyl-D-glucopyranose (S33)[40]

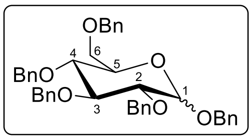

^{1}H NMR (500 MHz, CDCl$_3$): δ (α) 4.85 (d, J = 3.7 Hz, 1H, H-C1), 4.04 (t, J = 9.4 Hz, 1H, H-C3), 3.83–3.79 (m, 1H, H-C5), 3.74–3.69 (m, 1H, H$_a$-C6), 3.68–3.61 (m, 1H, H-C4), 3.61–3.54 (m, 2H, H-C2, H$_b$-C6); (β) 4.52 (d, J = 7.8 Hz, 1H H-C1), 3.77 (dd, J= 10.7, 2.0 Hz, 1H, H$_a$-C6), 3.71 (dd, J = 10.7, 5.0 Hz, 1H, H$_b$-C6), 3.65 (t, J = 8.7 Hz, 1H, H-C4), 3.63 (t, J = 8.7 Hz, 1H, H-C3), 3.55 (t, J = 8.7 Hz, 1H, H-C2), 3.47 (ddd, J = 8.7, 5.0, 2.0 Hz, 1H, H-C5); ^{13}C NMR (126 MHz, CDCl$_3$): δ (α) 95.78, 82.30, 80.05, 77.87, 70.53, 68.57; (β) 102.76, 84.90, 82.46, 78.05, 75.07, 69.11.

11. References

1. Fushimi, M.; Ishii, A.; Tsuruta, H.; Yamazaki, T. Industrial method for producing high-purity 2,3,4,6-tetra-*O*-acyl-D-mannopyranosyl fluoride. Japan patent JP2012017306A, January 26, 2012.
2. Nishino, T.; Ohya, Y.; Murai, R.; Shirahata, T.; Yamamoto, D.; Makino, K.; Kaji, E. *Heterocycles* **2012,** *84,* 1123–1140. doi: 10.3987/COM-11-S(P)94
3. Böhm, G.; Waldmann, H. *Liebigs Ann.* **1996,** *1996,* 621–625. doi: 10.1002/jlac.199619960423
4. Steinmann, A.; Thimm, J.; Matwiejuk, M.; Thiem, J. *Macromolecules* **2010,** *43,* 3606–3612. doi: 10.1021/ma100191d
5. Wen, P.; Crich, D. *Org. Lett.* **2017,** *19,* 2402–2405. doi: 10.1021/acs.orglett.7b00932
6. Bachmann, T.; Rychlik, M. *Carbohydr. Res.* **2020,** *489,* 107929. doi: 10.1016/j.carres.2020.107929
7. Fernández-Herrera, M. A.; Mohan, S.; López-Muñoz, H.; Hernández-Vázquez, J. M. V.; Pérez-Cervantes, E.; Escobar-Sánchez, M. L.; Sánchez-Sánchez, L.; Regla, I.; Pinto, B. M.; Sandoval-Ramírez, J. *Eur. J. Med. Chem.* **2010,** *45,* 4827–4837. doi: 10.1016/j.ejmech.2010.07.051
8. Kunz, H.; Harreus, A. *Liebigs Ann. Chem.* **1982,** *1982,* 41–48. doi: 10.1002/jlac.198219820105
9. Charville, H.; Jin, J.; Evans, C. W.; Brimble, M. A.; Williams, D. E. *RSC Adv.* **2013,** *3,* 15435–15441. doi: 10.1039/C3RA42781A
10. Rauschenberg, M.; Fritz, E.-C.; Schulz, C.; Kaufmann, T.; Ravoo, B. J. *Beilstein J. Org. Chem.* **2014,** *10,* 1354–1364. doi: 10.3762/bjoc.10.138
11. Cardona, A.; Boutureira, O.; Castillón, S.; Díaz, Y.; Matheu, M. I. *Green Chem.* **2017,** *19,* 2687–2694. doi: 10.1039/C7GC00722A
12. Chen, H.; Xian, T.; Zhang, W.; Si, W.; Luo, X.; Zhang, B.; Zhang, M.; Wang, Z.; Zhang, J. *Carbohydr. Res.* **2016,** *431,* 42–46. doi: 10.1016/j.carres.2016.05.013
13. Bucher, C.; Gilmour, R. *Angew. Chem. Int. Ed.* **2010,** *49,* 8724–8728. doi: 10.1002/anie.201004467
14. Beale, T. M.; Moon, P. J.; Taylor, M. S. *Org. Lett.* **2014,** *16,* 3604–3607. doi: 10.1021/ol501711v
15. Leškovskis, K.; Gulbe, K.; Mishnev, A.; Turks, M. *Tetrahedron Lett.* **2020,** *61,* 152528. doi: 10.1016/j.tetlet.2020.152528
16. Characteristic chemical shift of fluorine in FSO_2^- anion associated with phosphonium type counter cation is reported in: Zhu, S.-Z.; Huang, Q.-C.; Wu, K. *Inorg. Chem.* **1994,** *33,* 4584–4585. doi: 10.1021/ic00098a028
17. Becker, D.; Galili, N. *Tetrahedron Lett.* **1992,** *33,* 4775–4778. doi: 10.1016/S0040-4039(00)61283-2
18. Débieux, J.-L.; Cosandey, A.; Helgen, C.; Bochet, C. G. *Eur. J. Org. Chem.* **2007,** *2007,* 2073–2077. doi: 10.1002/ejoc.200600790
19. Poláková, M.; Šesták, S.; Lattová, E.; Petruš, L.; Mucha, J.; Tvaroška, I.; Kóňa, J. *Eur. J. Med. Chem.* **2011,** *46,* 944–952. doi: 10.1016/j.ejmech.2011.01.012
20. Weng, S.-S.; Li, C.-L.; Liao, C.-S.; Chen, T.-A.; Huang, C.-C.; Hung, K.-T. *J. Carbohydr. Chem.* **2010,** *29,* 429–440. doi: 10.1080/07328303.2011.565894
21. Limnios, D.; Kokotos, C. G. *Adv. Synth. Catal.* **2017,** *359,* 323–328. doi: 10.1002/adsc.201600977
22. Lv, W.; Chen, Y.; Wen, S.; Ba, D.; Cheng, G. *J. Am. Chem. Soc.* **2020,** *142,* 14864–14870. doi: 10.1021/jacs.0c07634
23. Liu, J.; Yin, S.; Wang, H.; Li, H.; Ni, G. *Carbohydr. Res.* **2020,** *490,* 107963. doi: 10.1016/j.carres.2020.107963
24. Toshima, K.; Nagai, H.; Kasumi, K.; Kawahara, K.; Matsumura, S. *Tetrahedron* **2004,** *60,* 5331–5339. doi: 10.1016/j.tet.2004.04.071
25. Hotha, S.; Kashyap, S. *J. Am. Chem. Soc.* **2006,** *128,* 9620–9621. doi: 10.1021/ja062425c

26. Iwata, R.; Uda, K.; Takahashi, D.; Toshima, K. *Chem. Commun.* **2014,** *50,* 10695–10698. doi: 10.1039/C4CC04753B
27. Verdelet, T.; Benmahdjoub, S.; Benmerad, B.; Alami, M.; Messaoudi, S. *J. Org. Chem.* **2019,** *84,* 9226–9238. doi: 10.1021/acs.joc.9b01218
28. Griffin, F. K.; Paterson, D. E.; Murphy, P. V; Taylor, R. J. K. *Eur. J. Org. Chem.* **2002,** *2002,* 1305–1322. doi: 10.1002/1099-0690(200204)2002:7<1305::AID-EJOC1305>3.0.CO;2-0
29. Rutschow, S.; Thiem, J.; Kranz, C.; Marquardt, T. *Bioorg. Med. Chem.* **2002,** *10,* 4043–4049. doi: 10.1016/S0968-0896(02)00269-9
30. Yamanoi, T.; Inoue, R.; Matsuda, S.; Iwao, K.; Oda, Y.; Yoshida, A.; Hamasaki, K. *Heterocycles* **2009,** *77,* 445–460. doi: 10.3987/COM-08-S(F)41
31. Kunz, H.; Sager, W. *Helv. Chim. Acta* **1985,** *68,* 283–287. doi: 10.1002/hlca.19850680134
32. Triantakonstanti, V. V; Toskas, A.; Iordanidis, N. S.; Andreou, T.; Koftis, T. V; Gallos, J. K. *Tetrahedron Lett.* **2020,** *61,* 152173. doi: 10.1016/j.tetlet.2020.152173
33. Santoyo-González, F.; Uriel, C.; Calvo-Asín, J. A. *Synthesis* **1998,** *1998,* 1787–1792. doi: 10.1055/s-1998-2224
34. Ikeda, K.; Morimoto, T.; Kakiuchi, K. *J. Org. Chem.* **2010,** *75,* 6279–6282. doi: 10.1021/jo1012288
35. Hanessian, S.; Kagotani, M. *Carbohydr. Res.* **1990,** *202,* 67–79. doi: 10.1016/0008-6215(90)84071-2
36. Yu, H.; Ensley, H. E. *Tetrahedron Lett.* **2003,** *44,* 9363–9366. doi: 10.1016/j.tetlet.2003.09.230
37. Miller, G. J.; Gardiner, J. M. *Org. Lett.* **2010,** *12,* 5262–5265. doi: 10.1021/ol102310x
38. Wang, Y.; Zhang, X.; Wang, P. *Org. Biomol. Chem.* **2010,** *8,* 4322–4328. doi: 10.1039/C002865G
39. Zeng, J.; Vedachalam, S.; Xiang, S.; Liu, X.-W. *Org. Lett.* **2011,** *13,* 42–45. doi: 10.1021/ol102473k
40. Saehlim, N.; Athipornchai, A.; Sirion, U.; Saeeng, R. *Bioorg. Med. Chem. Lett.* **2020,** *30,* 127276. doi: 10.1016/j.bmcl.2020.127276
