# Supplementary material for: Metal-free glycosylation with glycosyl fluorides in liquid SO2
Source: Beilstein J Org Chem. 2021 Apr 29;17:964–76. doi: 10.3762/bjoc.17.78 (PMC8093551; doi:10.3762/bjoc.17.78)

BEILSTEIN JOURNAL OF ORGANIC CHEMISTRY

Supporting Information

for

Metal-free glycosylation with glycosyl fluorides in liquid SO_2

Krista Gulbe, Jevgeņija Lugiņina, Edijs Jansons, Artis Kinens and Māris Turks

Beilstein J. Org. Chem. **2021,** *17,* 964–976. doi:10.3762/bjoc.17.78

Copies of NMR spectra

License and Terms: This is a supporting information file under the terms of the Creative Commons Attribution License (https://creativecommons.org/licenses/by/4.0). Please note that the reuse, redistribution and reproduction in particular requires that the author(s) and source are credited and that individual graphics may be subject to special legal provisions.
The license is subject to the *Beilstein Journal of Organic Chemistry* terms and conditions: (https://www.beilstein-journals.org/bjoc/terms)

1. Glycosyl donors S2-2
2. Intermediates isolated in the synthesis of 2-deoxy glucosyl fluoride α-**19** S2-19
3. Target glycosides S2-24
4. Side-products S2-88

1. GLYCOSYL DONORS

α-**1a**

^{1}H NMR (500 MHz, $CDCl_3$)

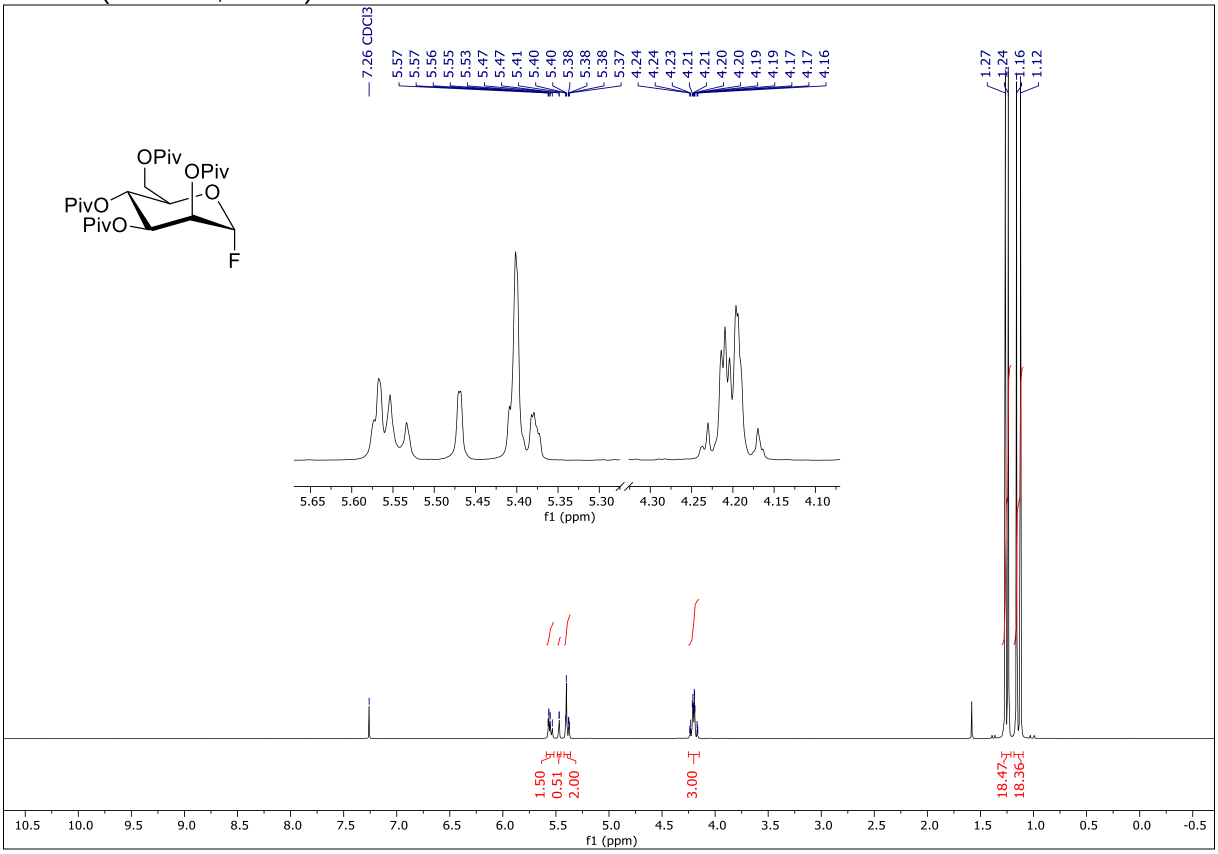

^{13}C NMR (126 MHz, $CDCl_3$)

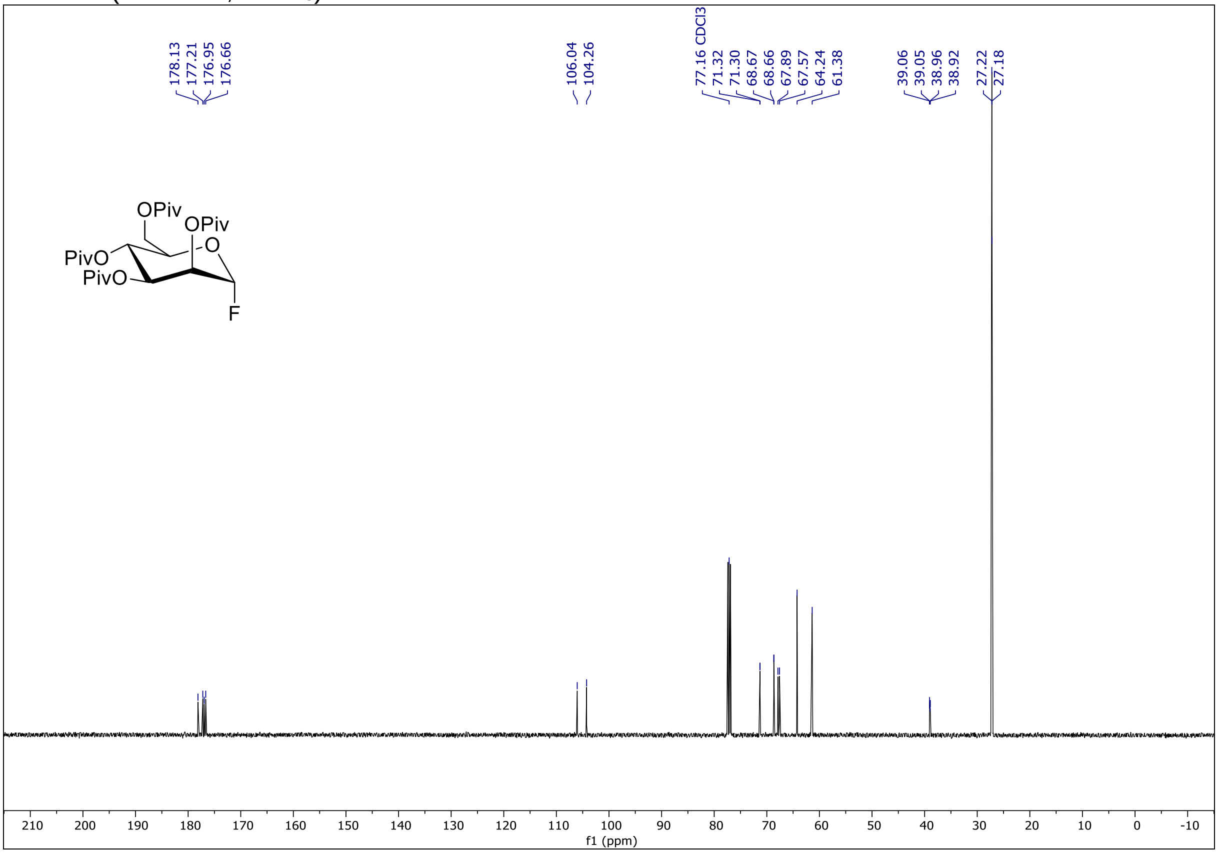

α-**1b**

^{1}H NMR (500 MHz, $CDCl_3$)

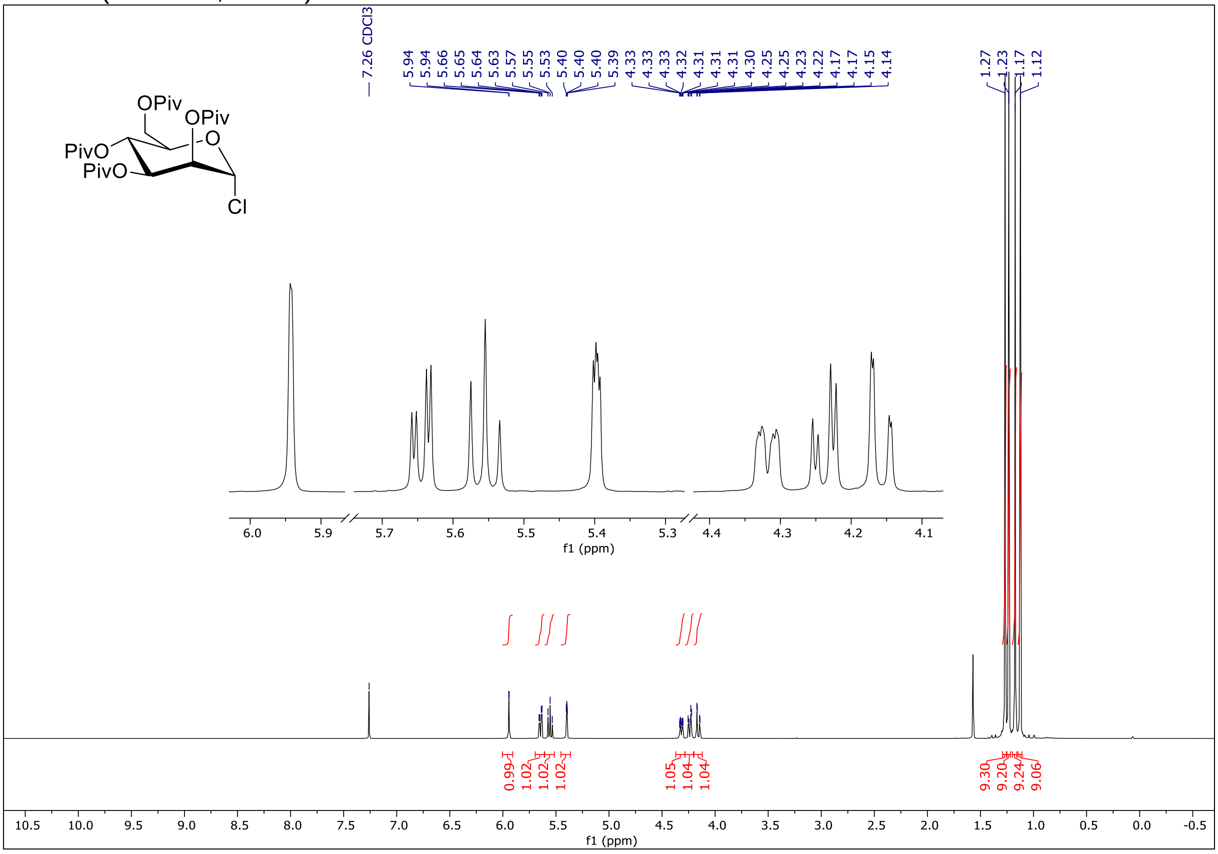

^{13}C NMR (126 MHz, $CDCl_3$)

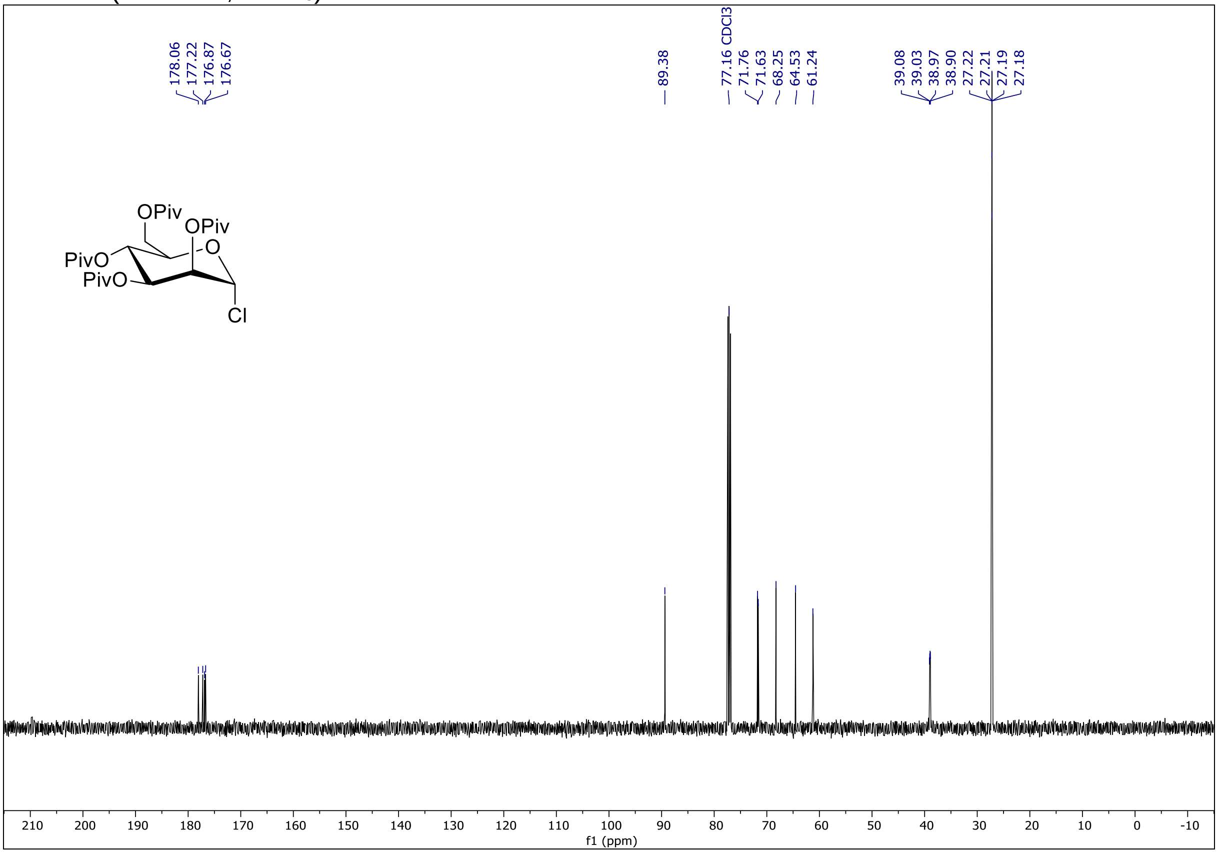

α-**1c**

^{1}H NMR (500 MHz, $CDCl_3$)

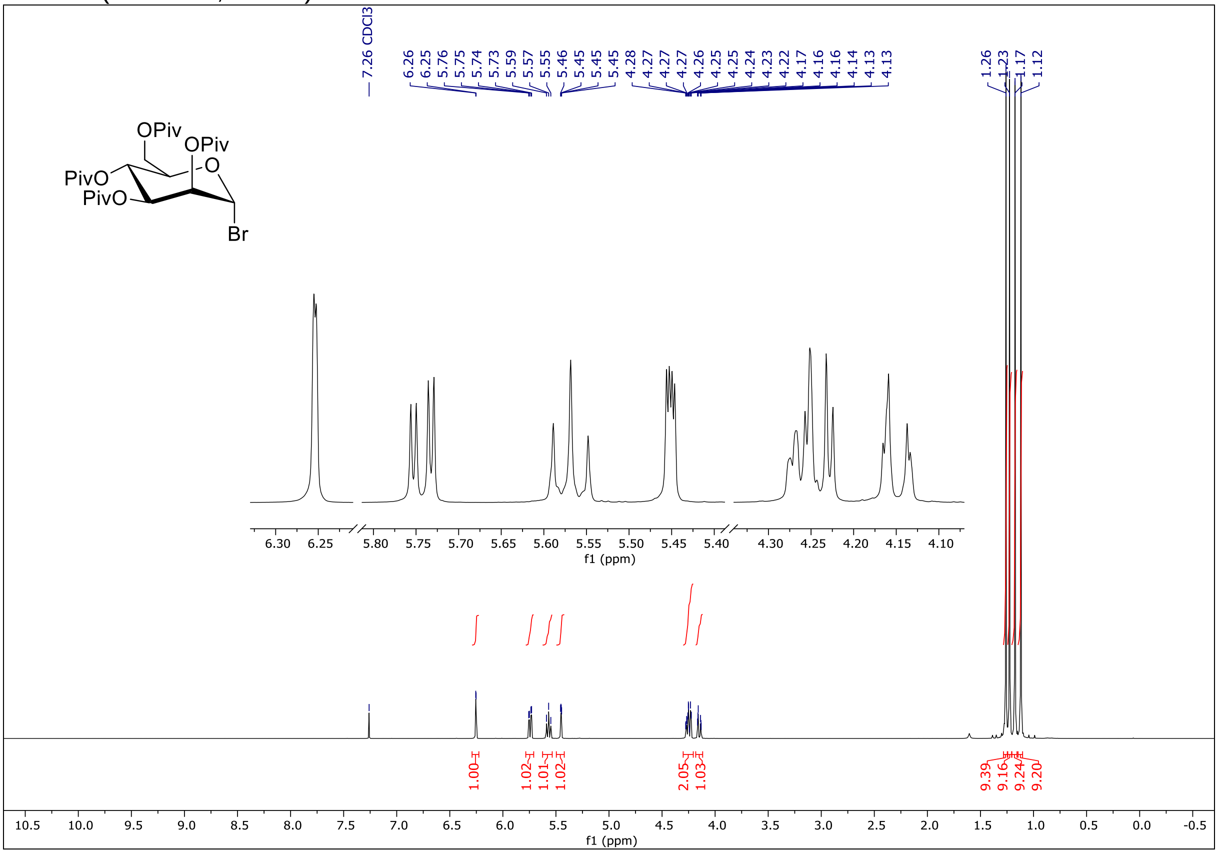

^{13}C NMR (126 MHz, $CDCl_3$)

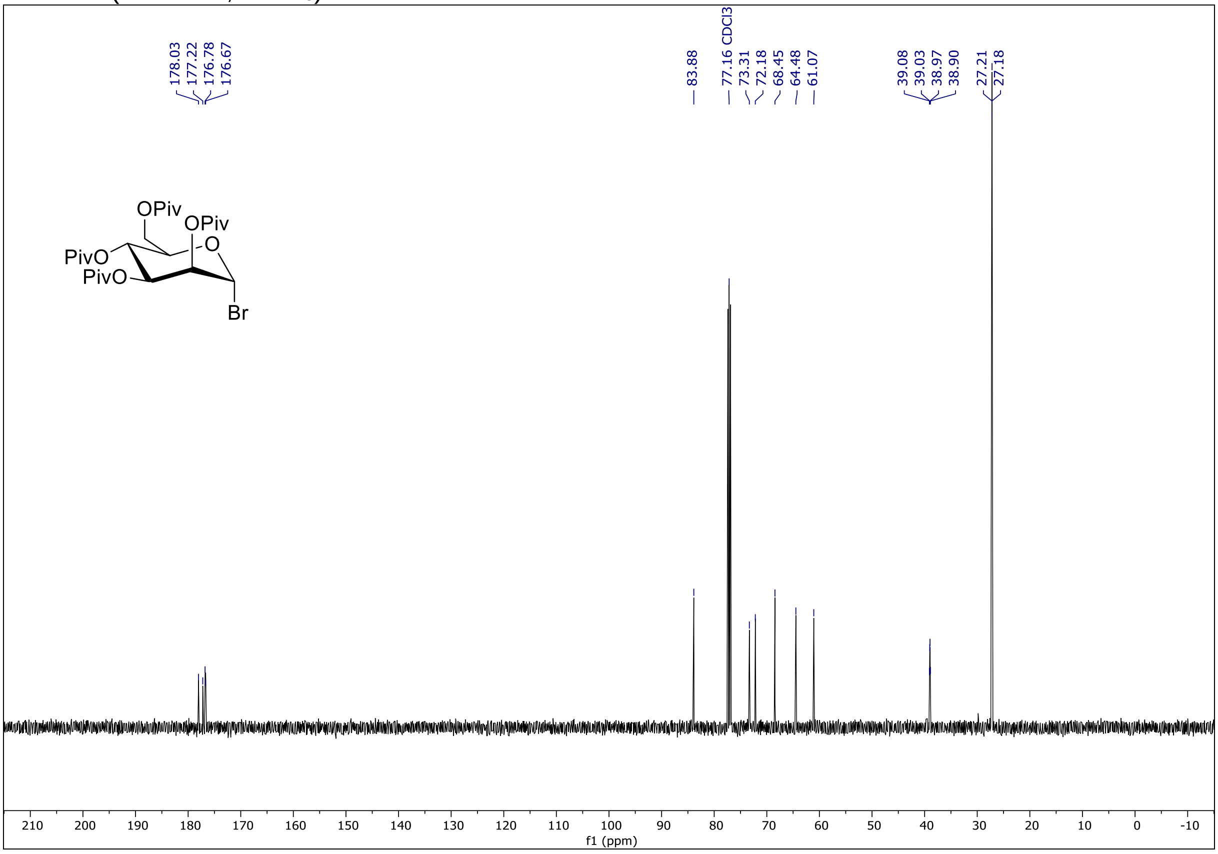

β-**9**

^{1}H NMR (500 MHz, CDCl$_3$)

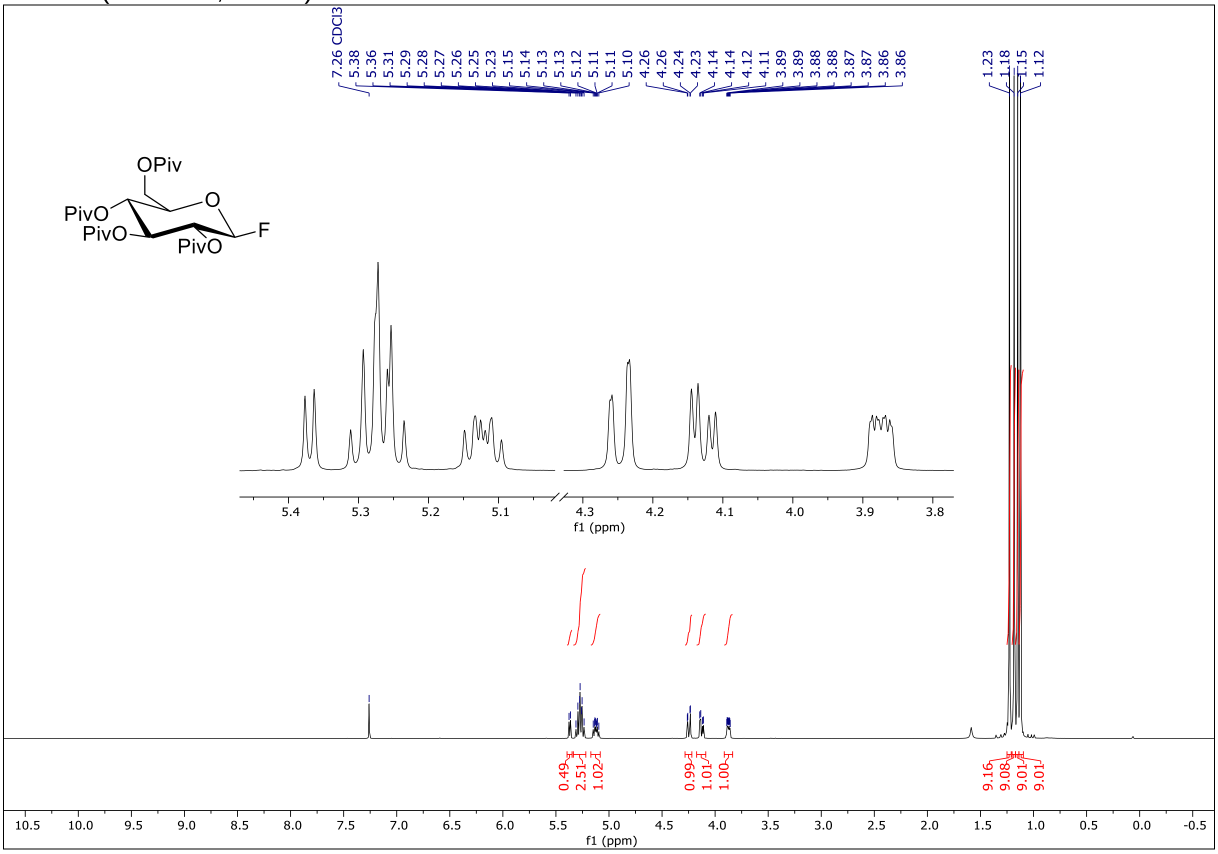

^{13}C NMR (126 MHz, CDCl$_3$)

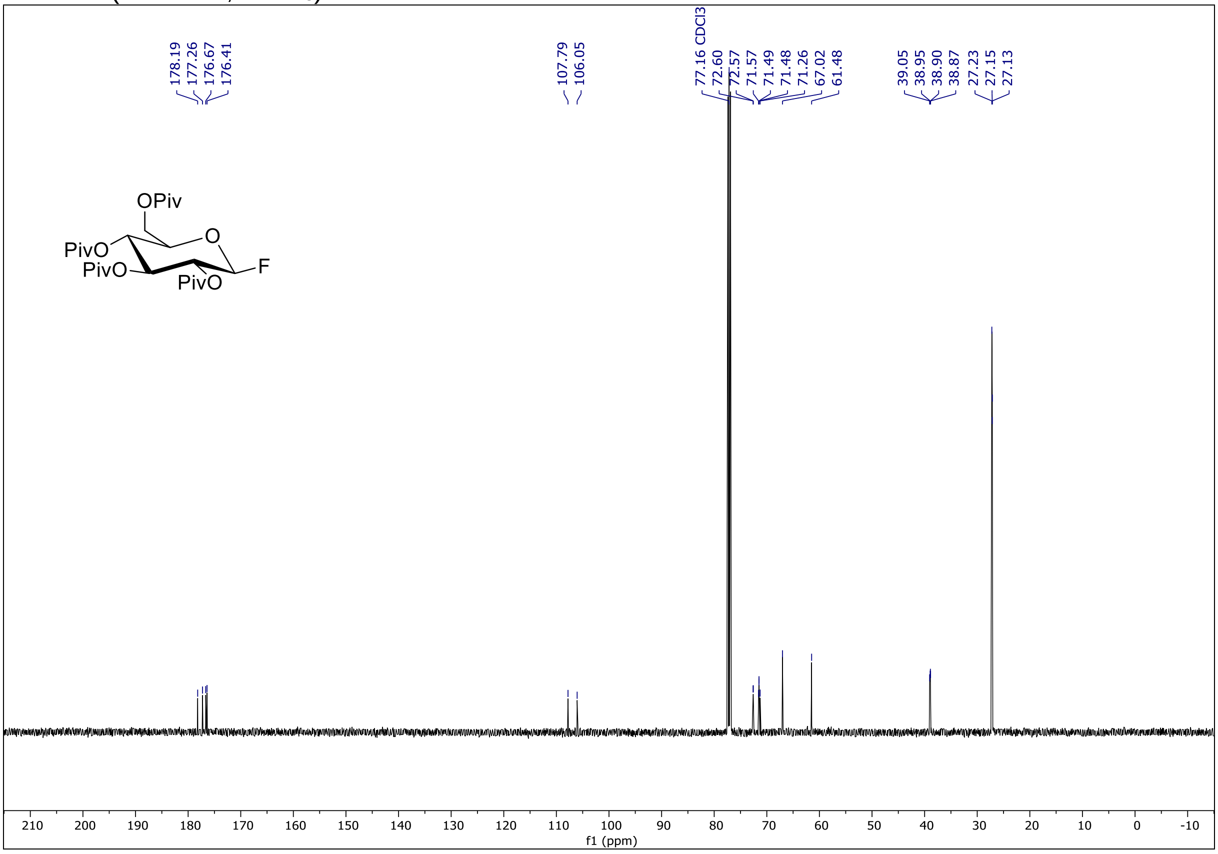

α-**11**

^{1}H NMR (500 MHz, CDCl$_3$)

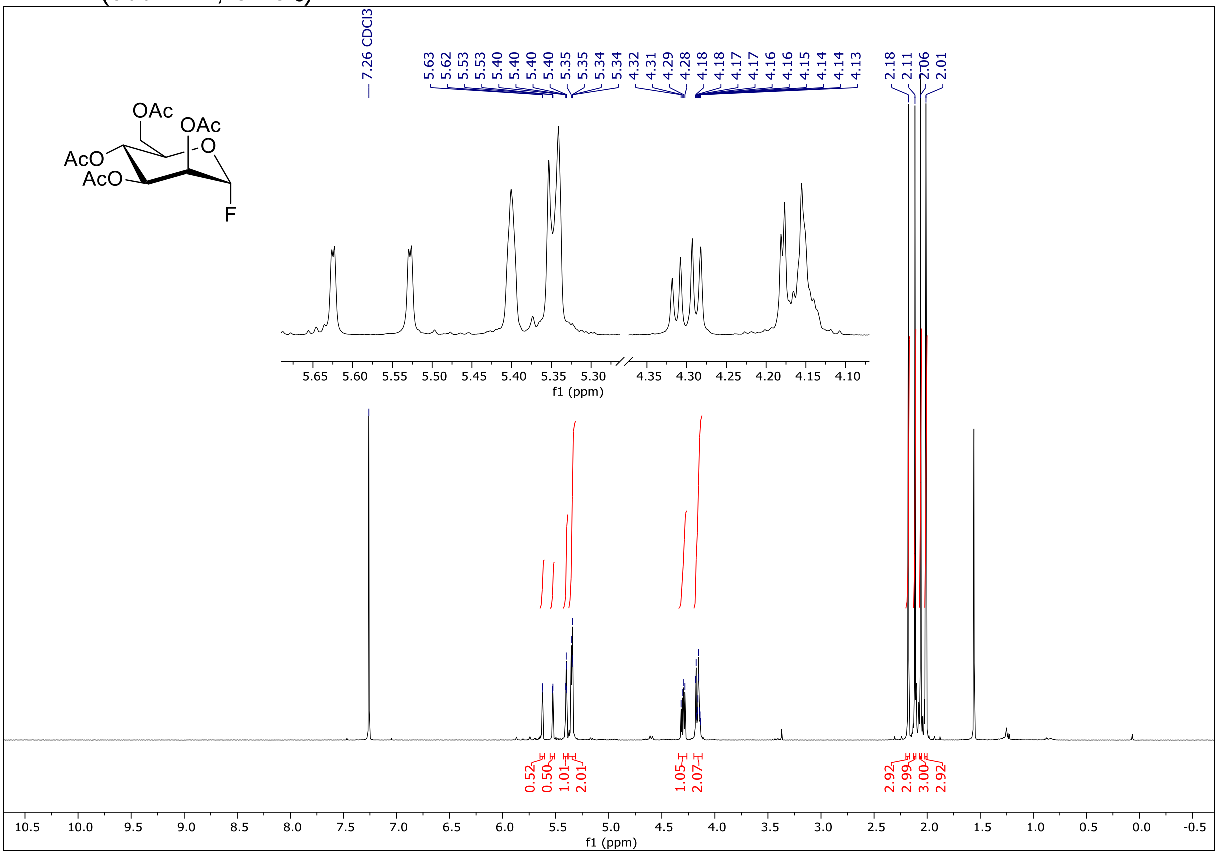

^{13}C NMR (126 MHz, CDCl$_3$)

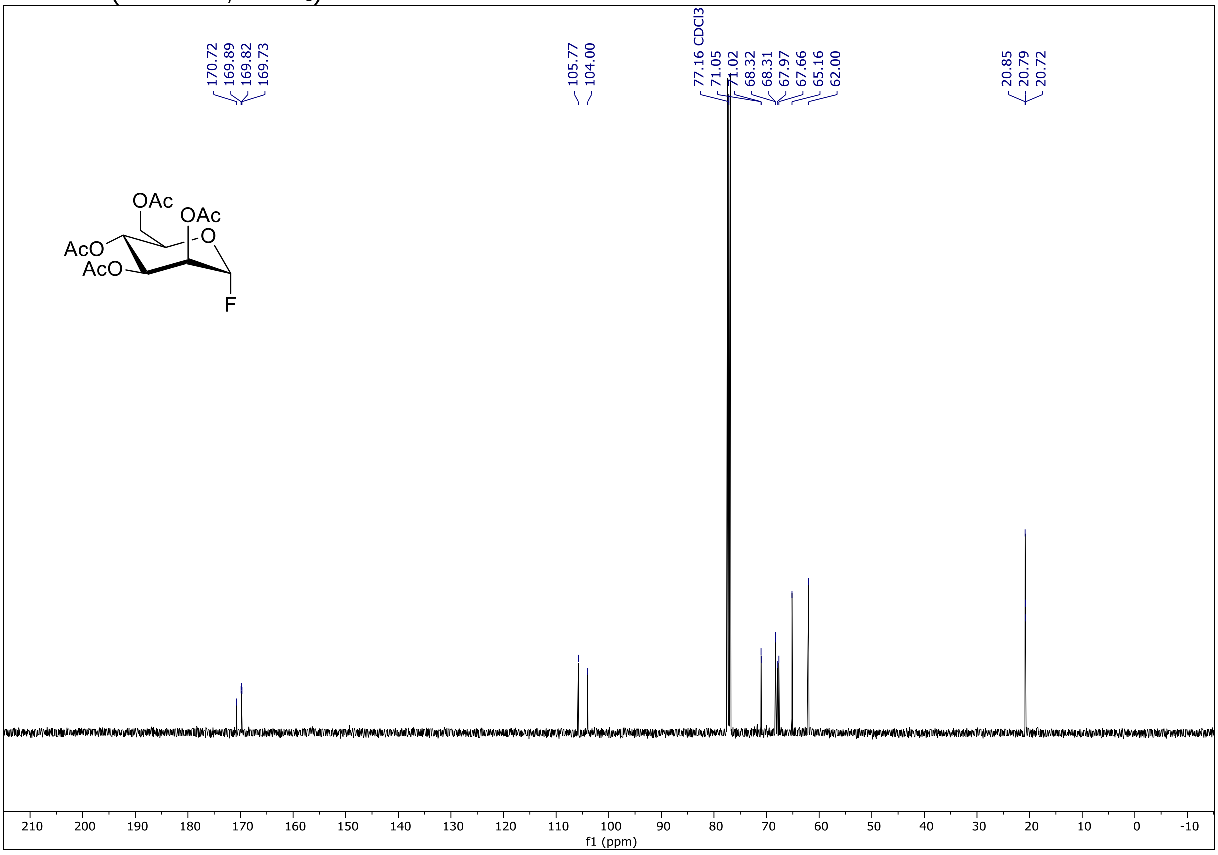

α-12

^{1}H NMR (500 MHz, $CDCl_3$)

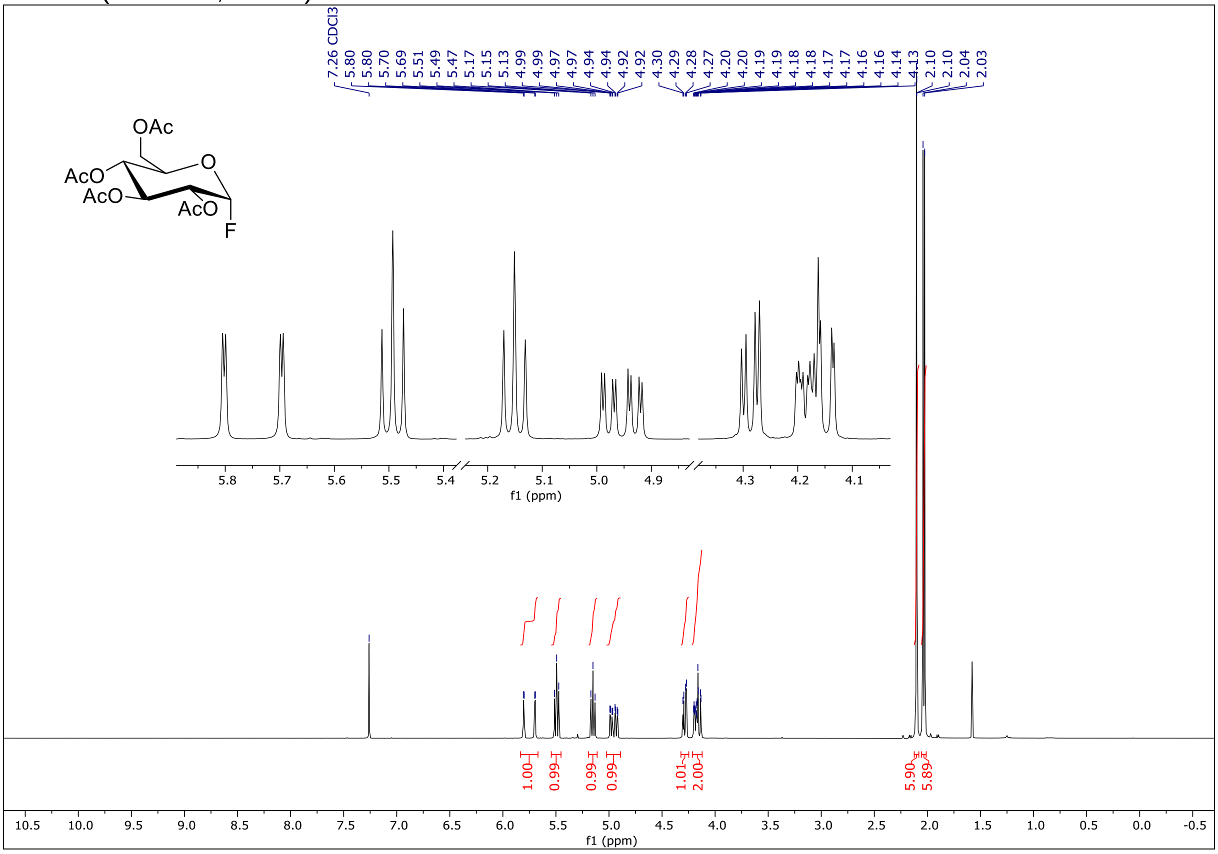

^{13}C NMR (126 MHz, $CDCl_3$)

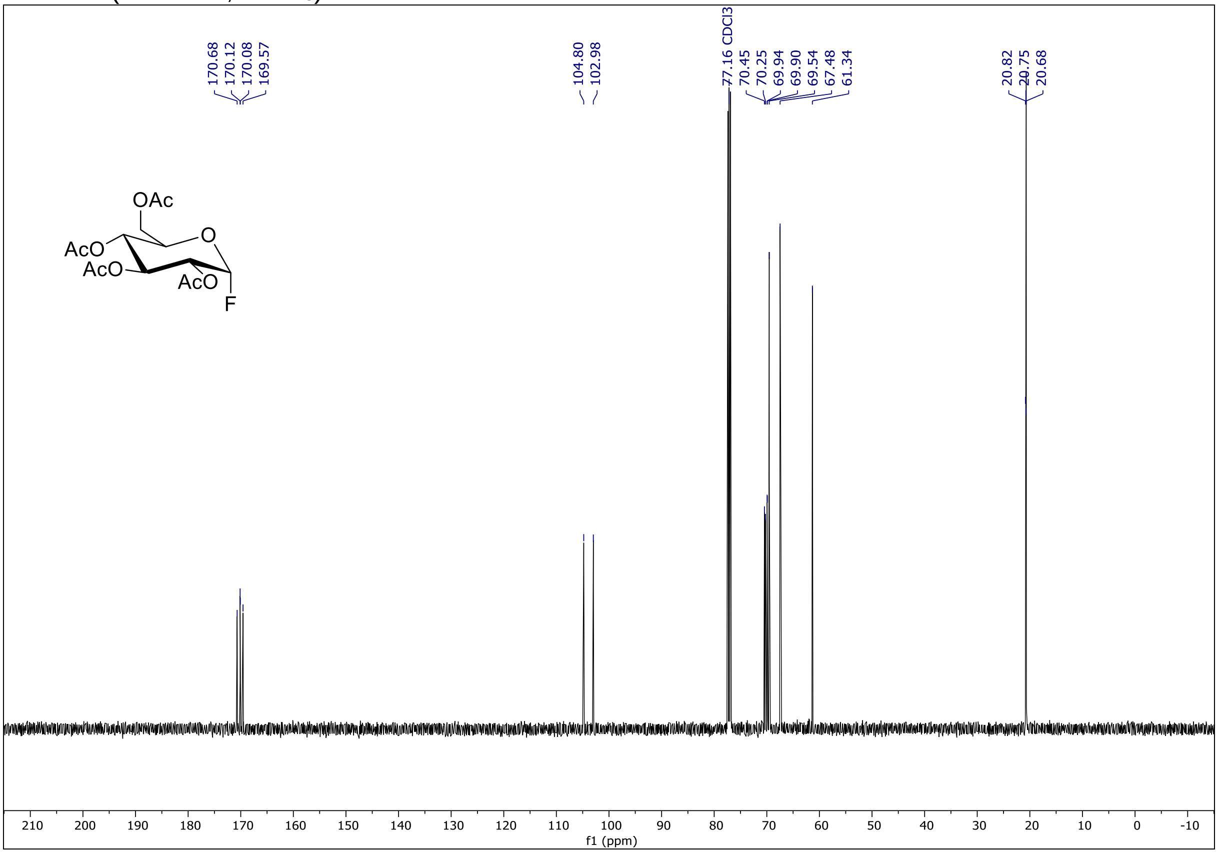

α-**15**

^{1}H NMR (500 MHz, $CDCl_3$)

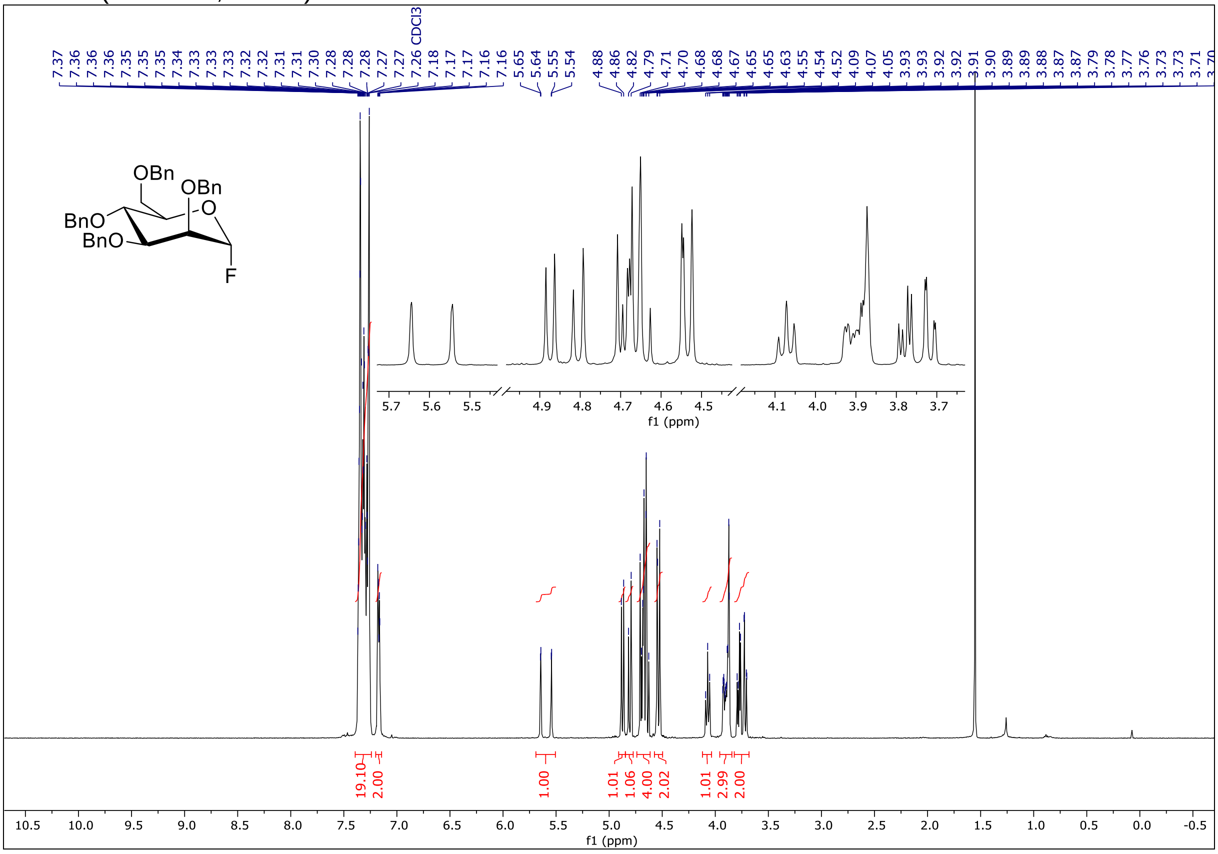

^{13}C NMR (126 MHz, $CDCl_3$)

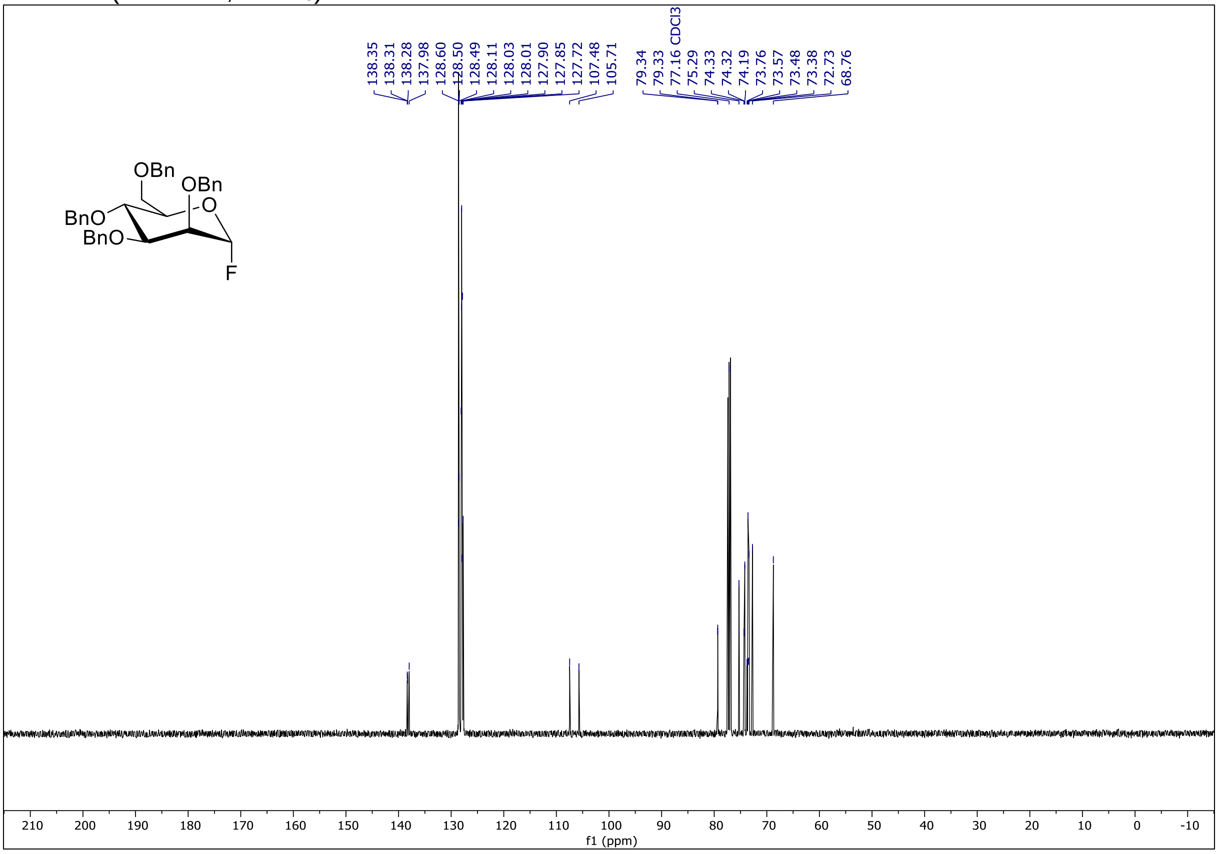

α-**16**

^{1}H NMR (500 MHz, $CDCl_3$)

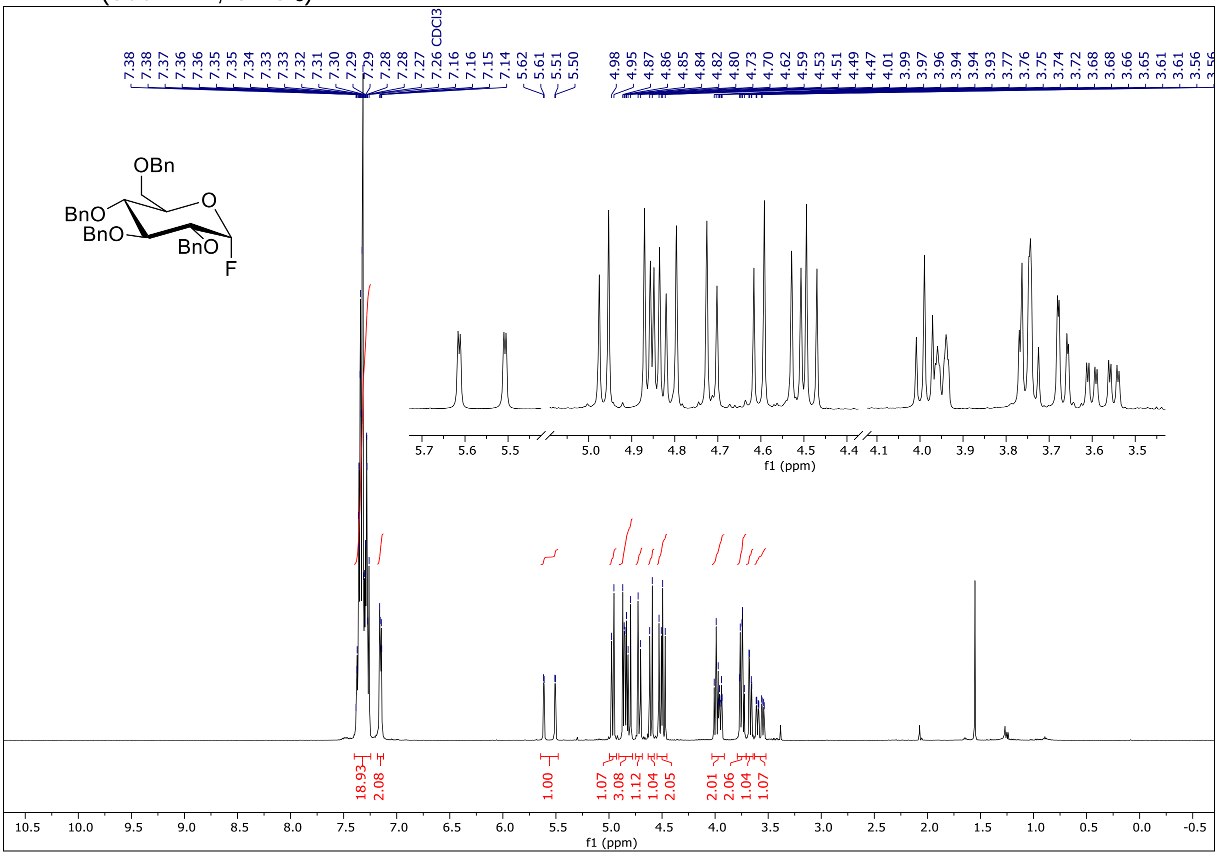

^{13}C NMR (126 MHz, $CDCl_3$)

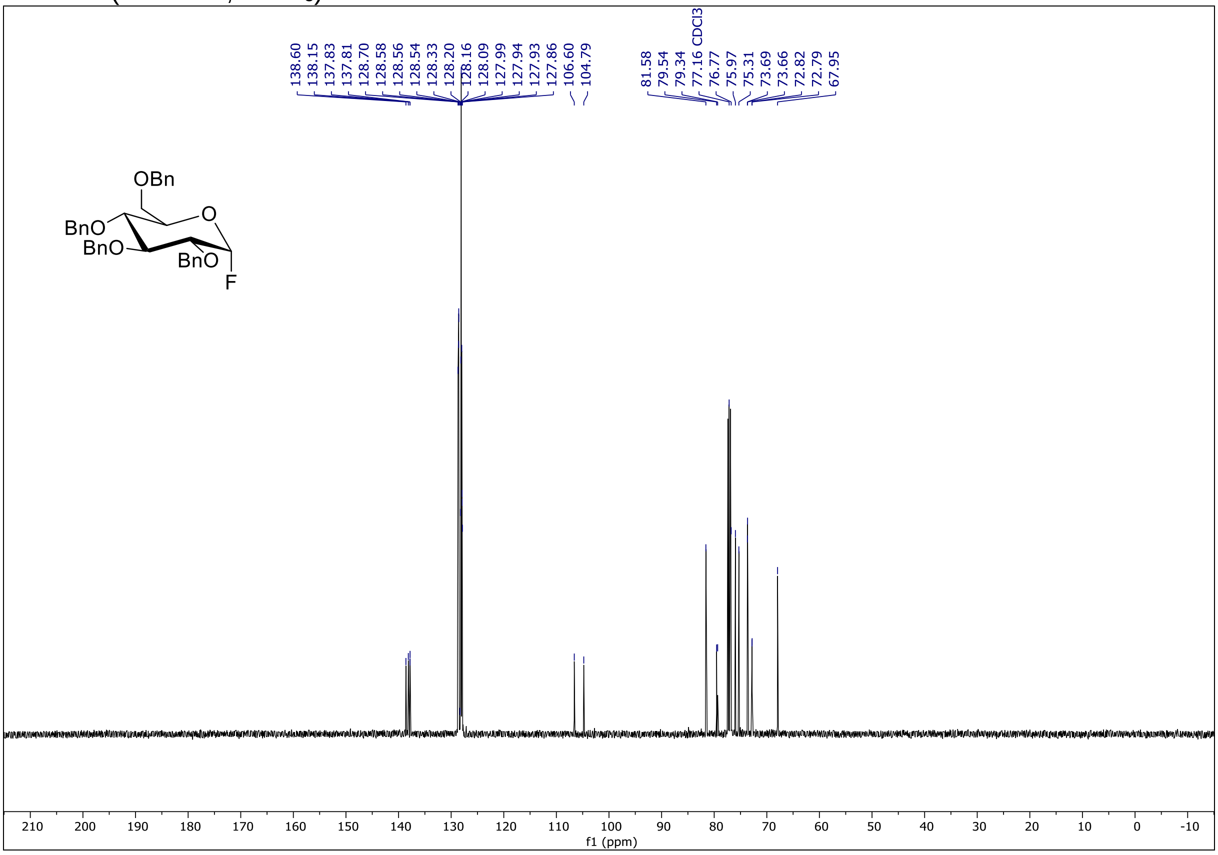

β-**16**

^{1}H NMR (500 MHz, $CDCl_3$)

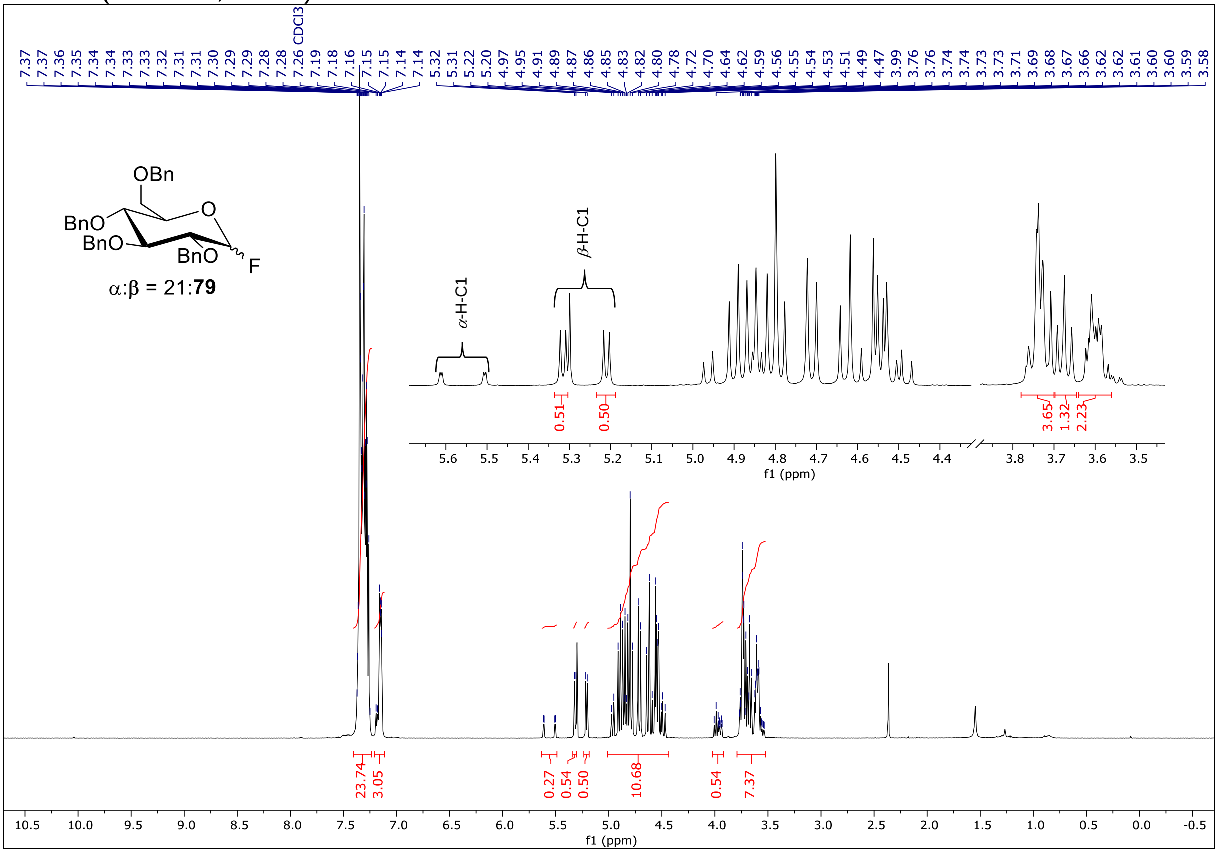

^{13}C NMR (126 MHz, $CDCl_3$)

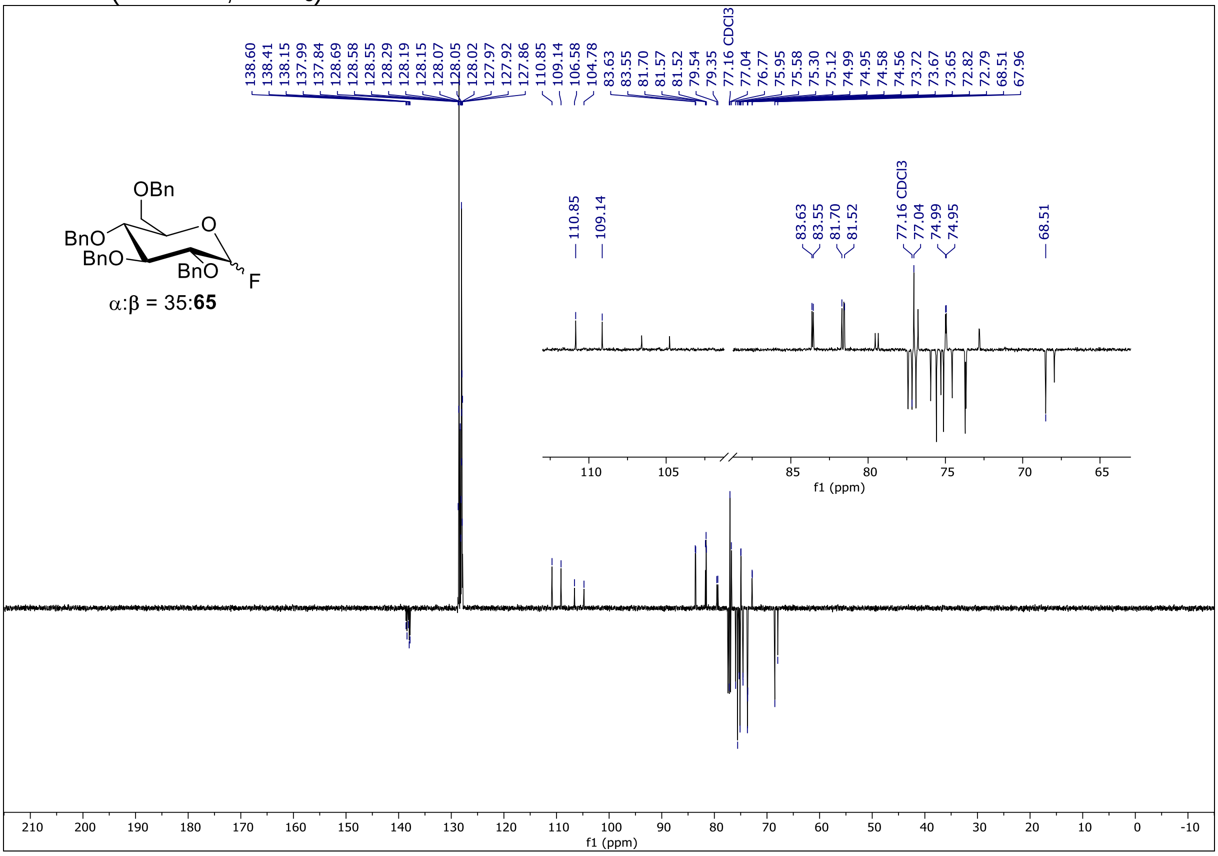

α-19

^{1}H NMR (500 MHz, $CDCl_3$)

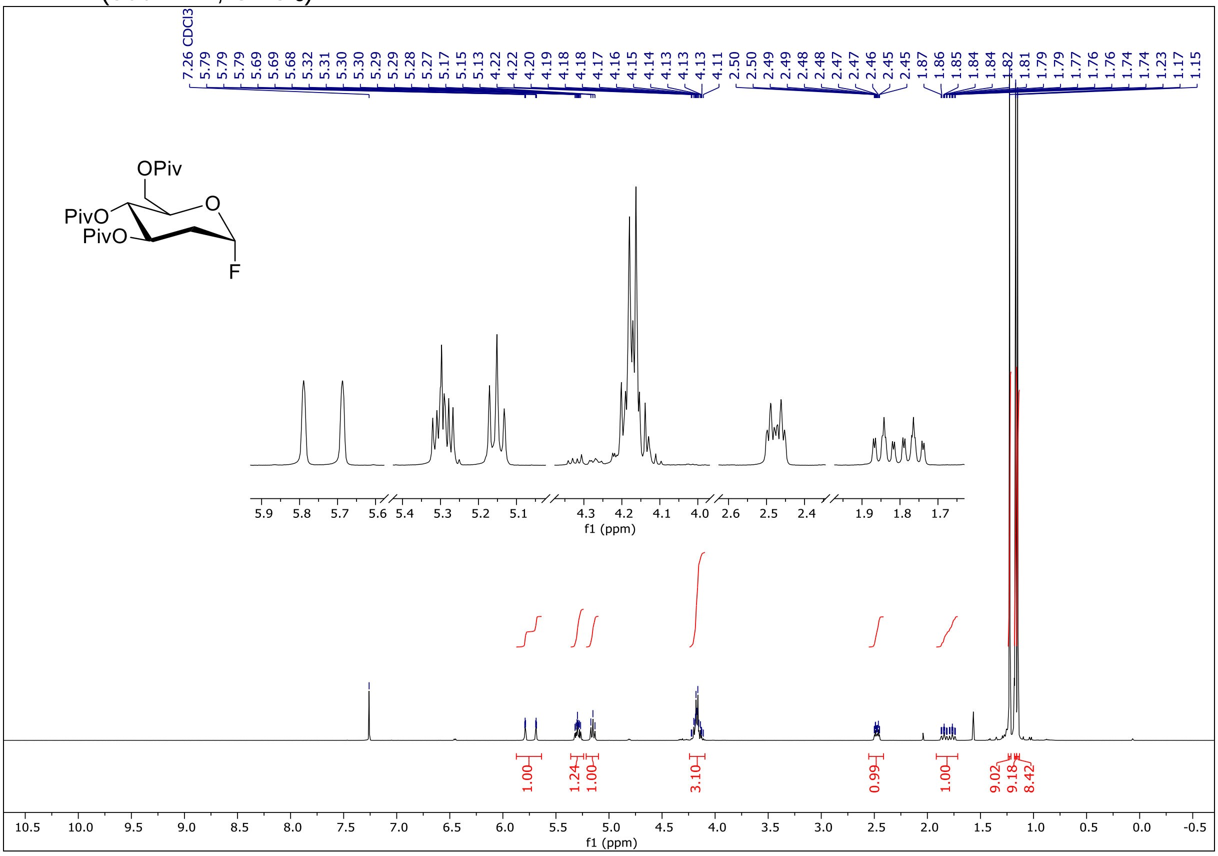

^{13}C NMR (126 MHz, $CDCl_3$)

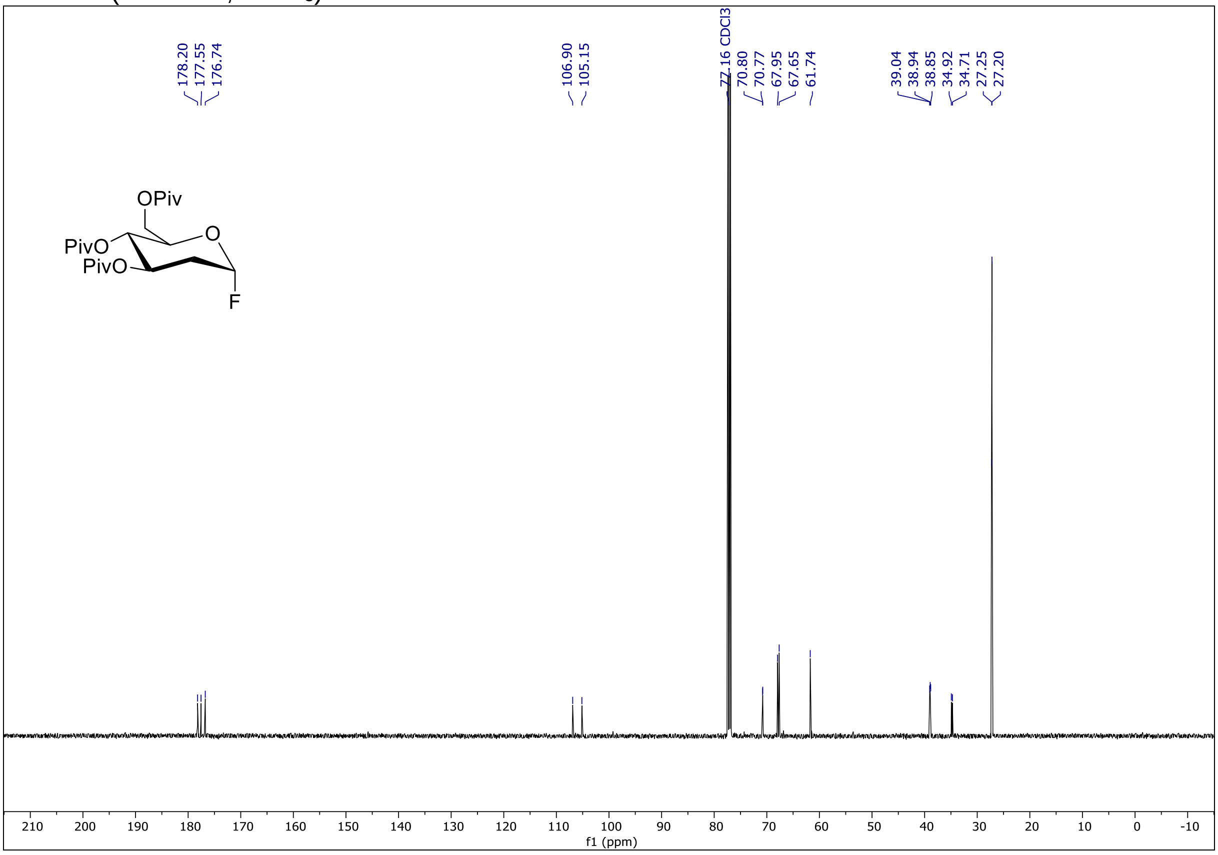

β-**19**

^{1}H NMR (500 MHz, $CDCl_3$)

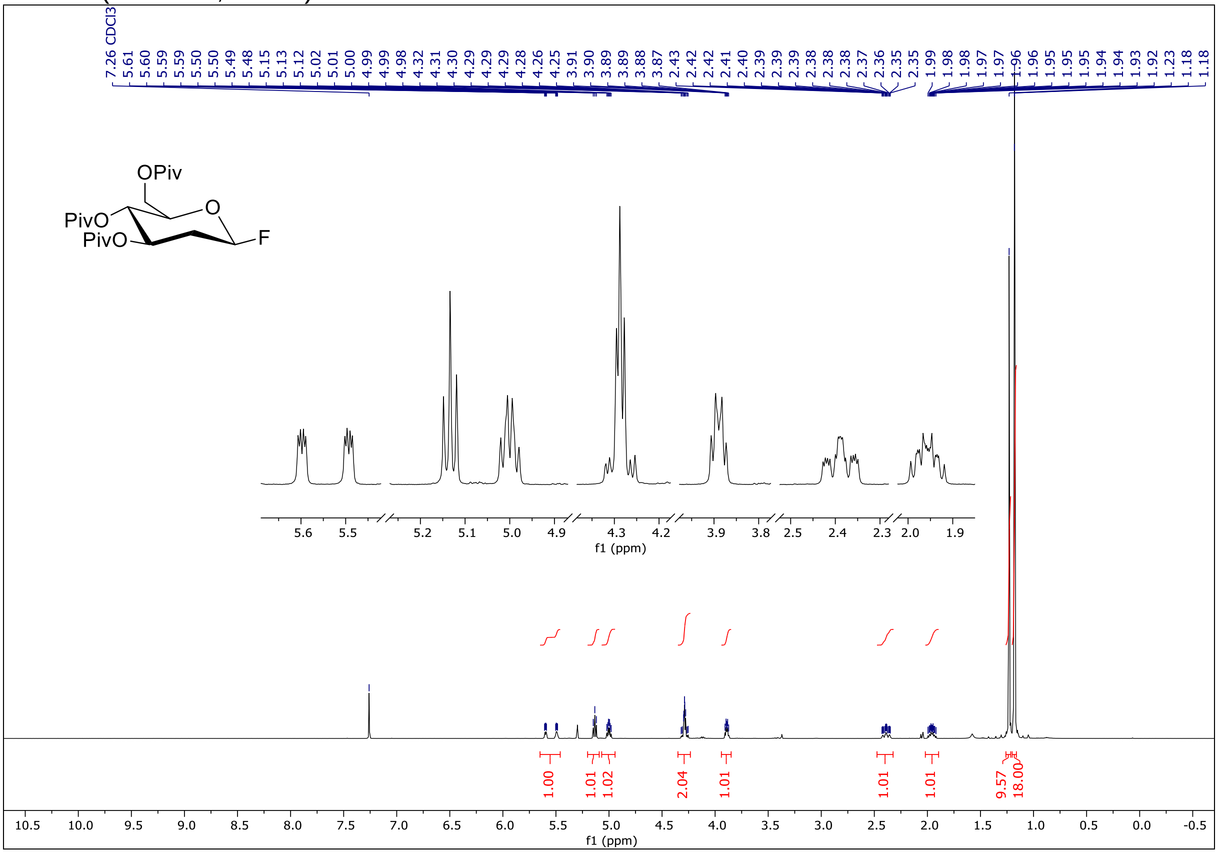

^{13}C NMR (126 MHz, $CDCl_3$)

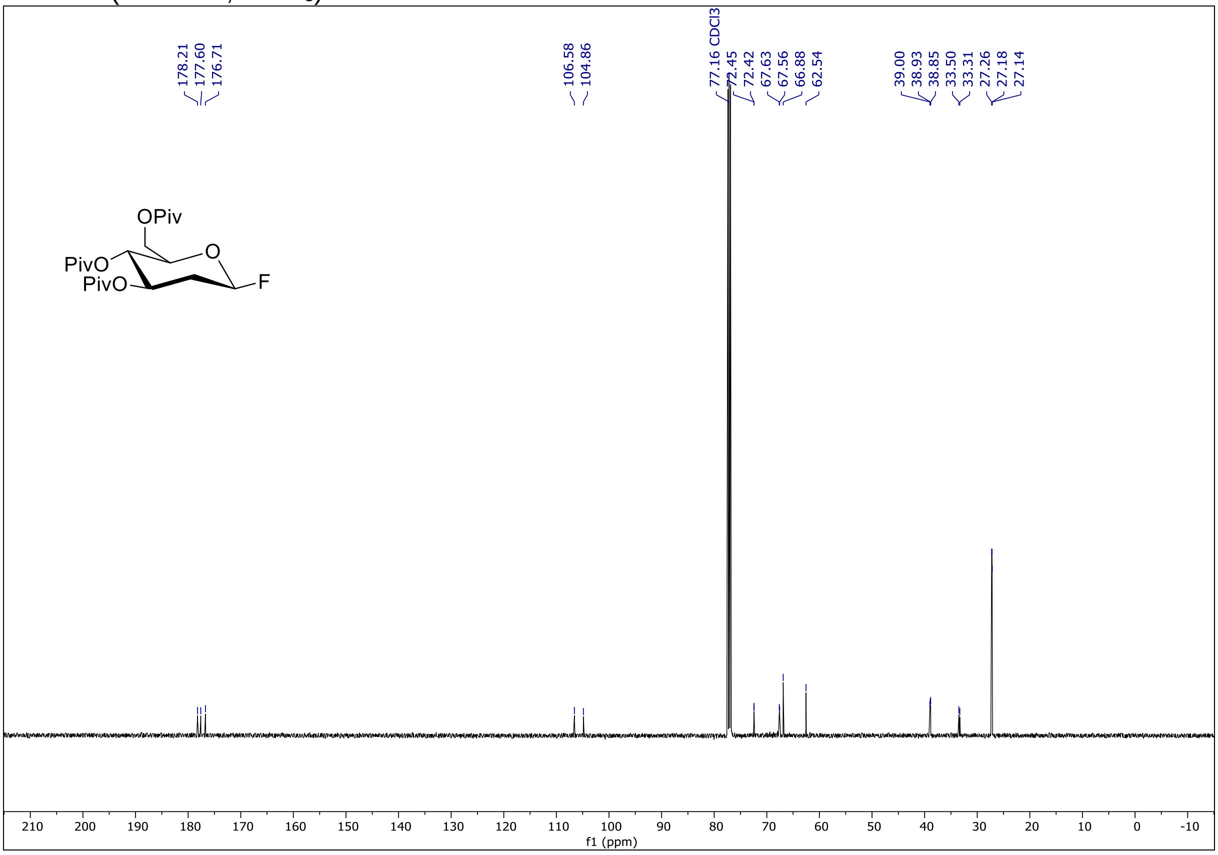

α-S9

^{1}H NMR (500 MHz, $CDCl_3$)

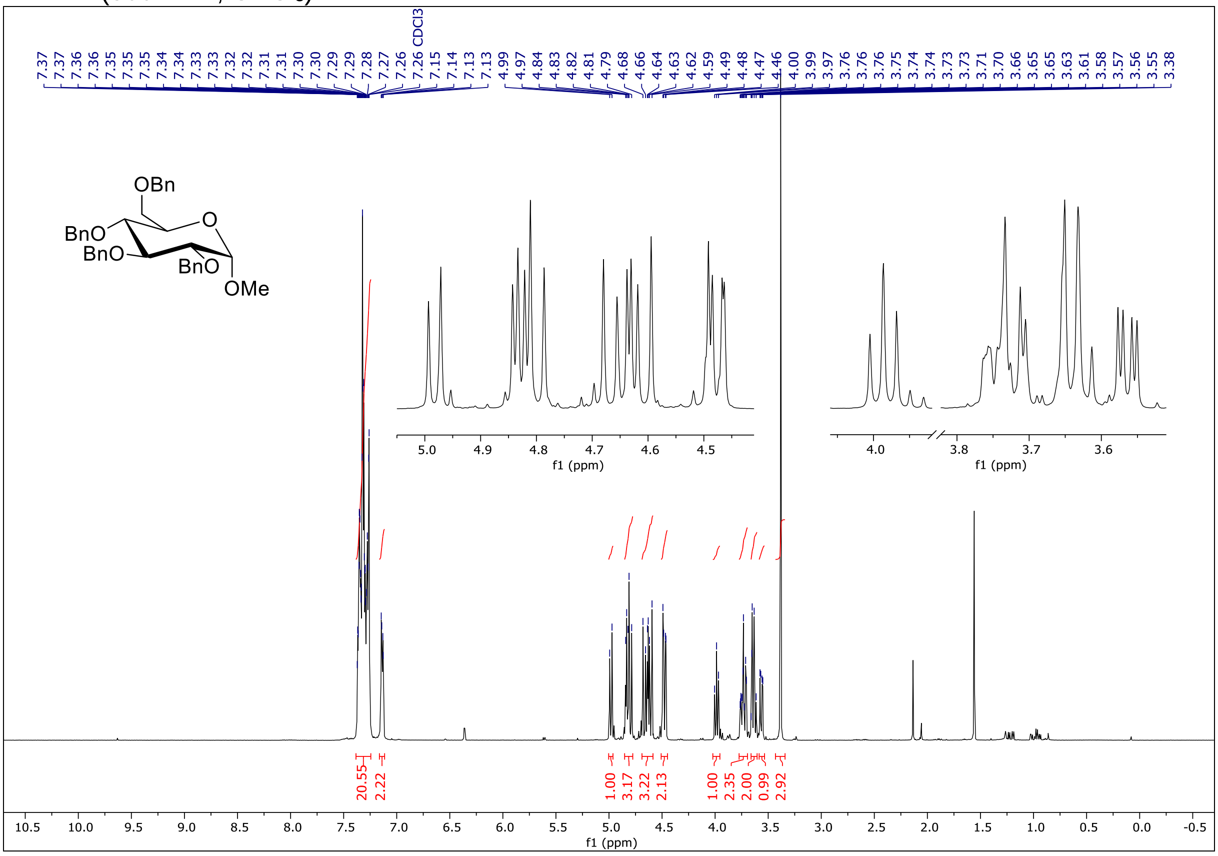

^{13}C NMR (126 MHz, $CDCl_3$)

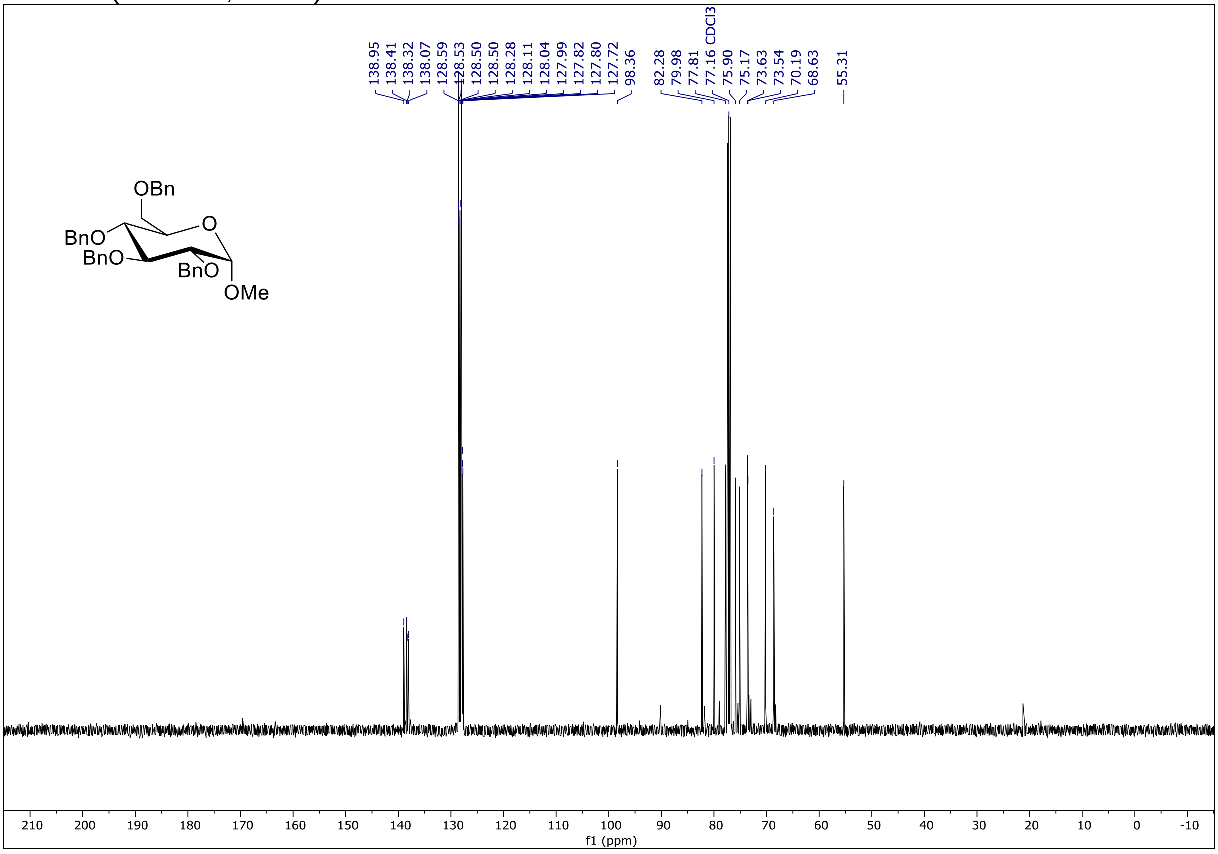

S10

^{1}H NMR (500 MHz, $CDCl_3$)

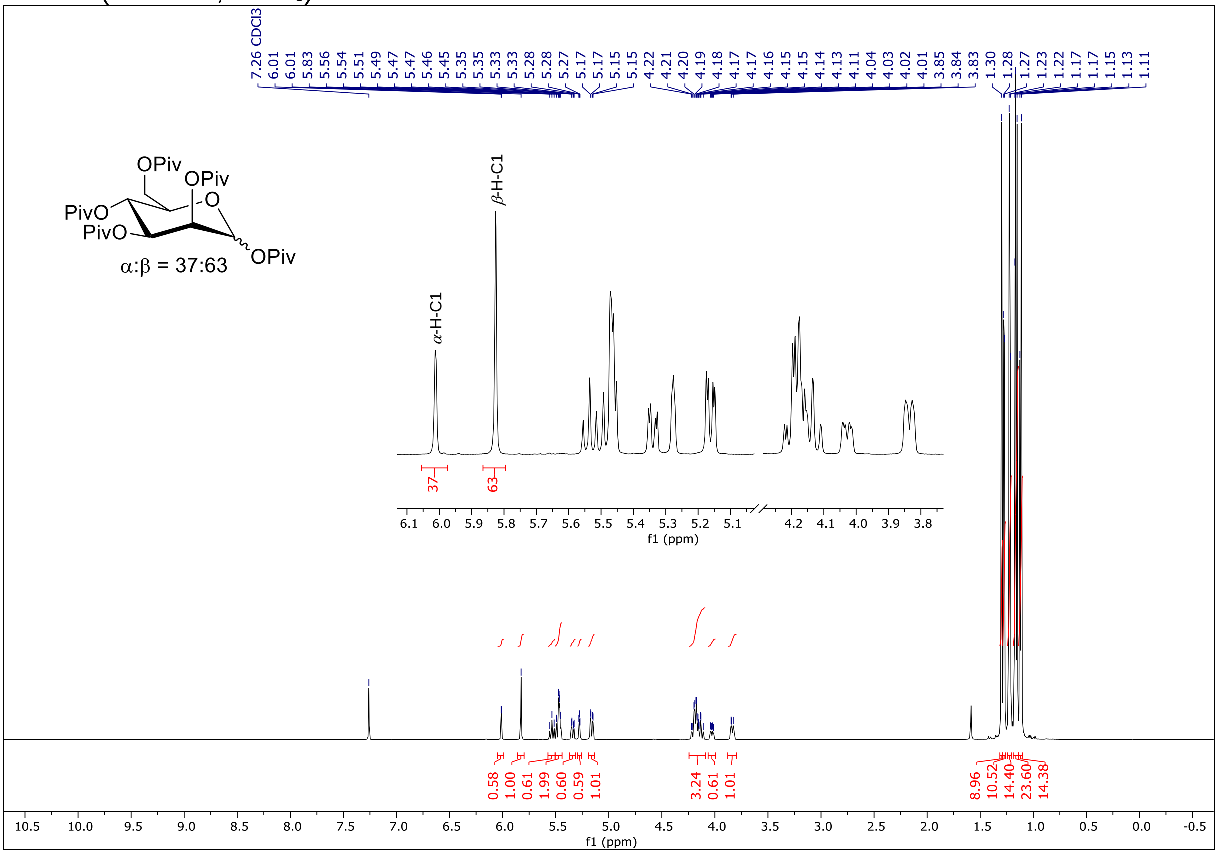

^{13}C NMR (126 MHz, $CDCl_3$)

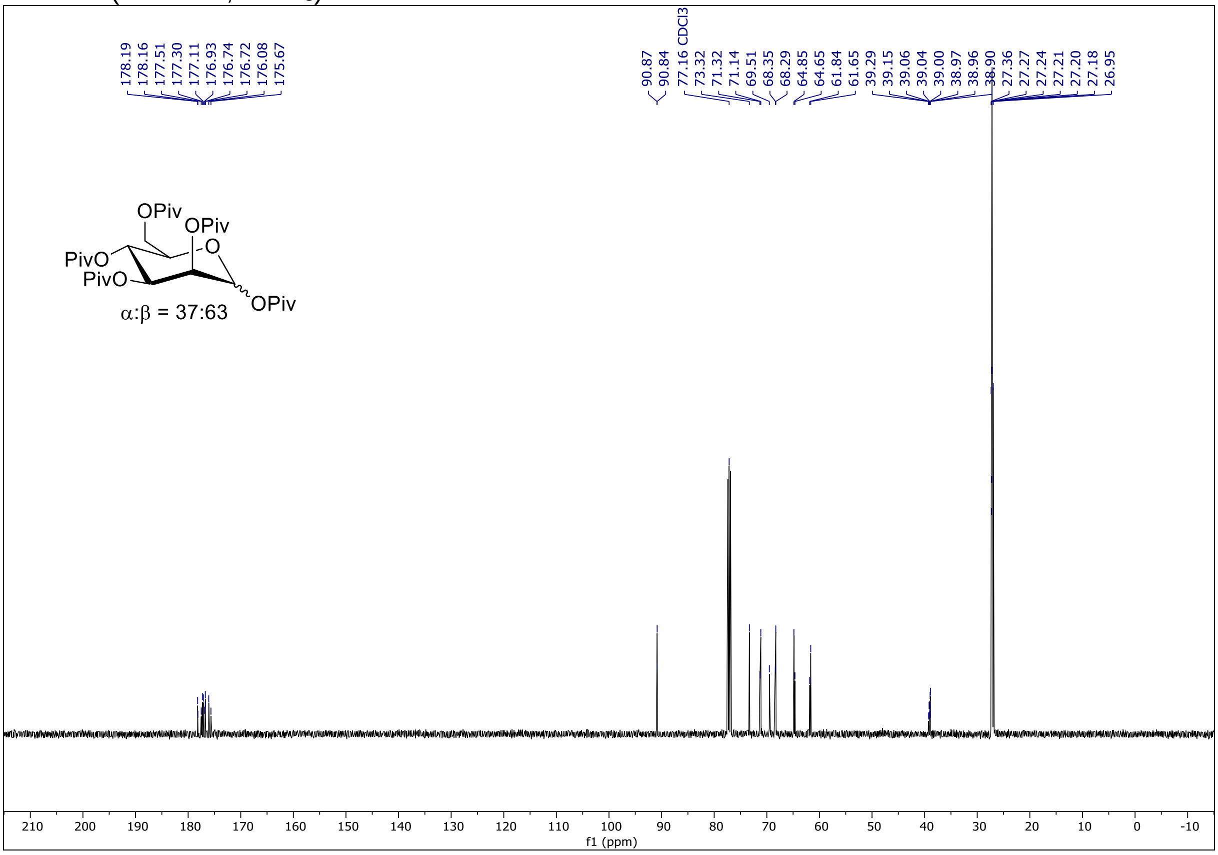

β-**S11**

^{1}H NMR (500 MHz, $CDCl_3$)

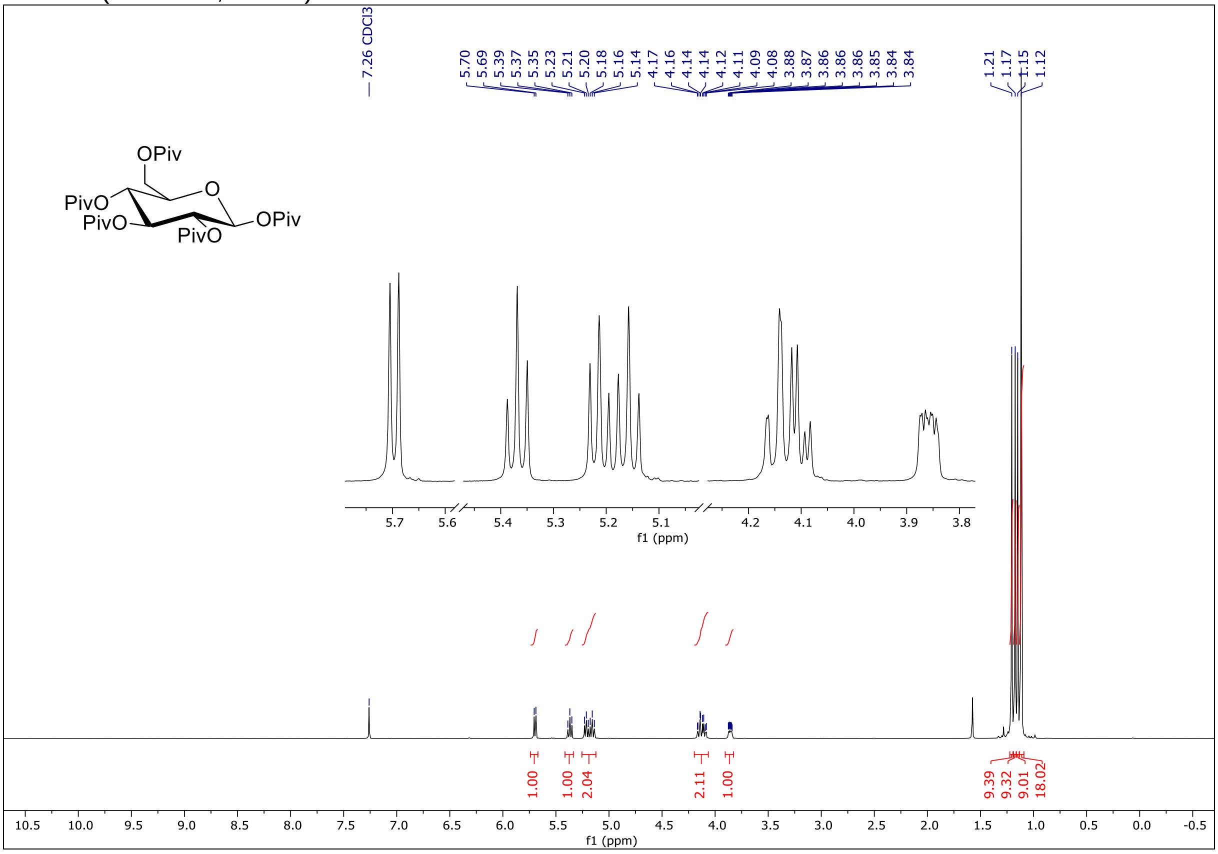

^{13}C NMR (126 MHz, $CDCl_3$)

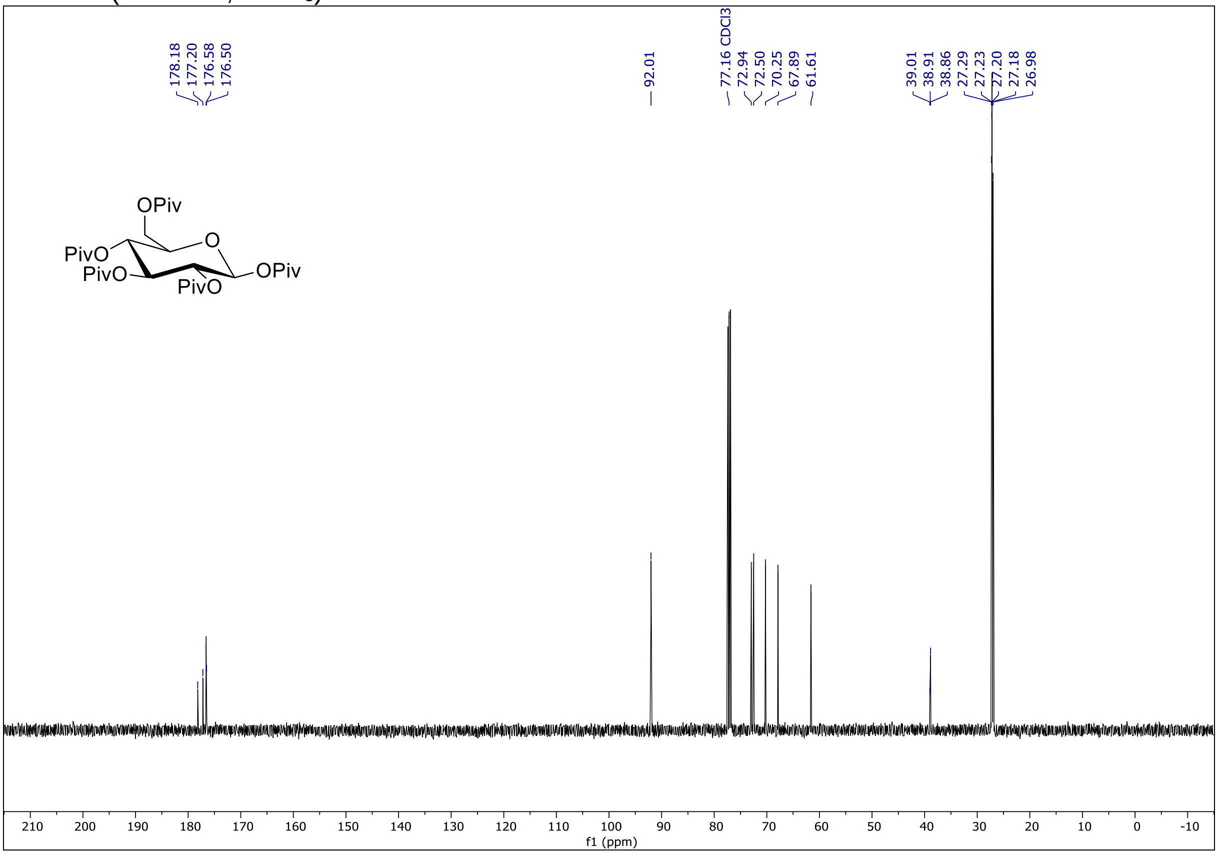

S12

^{1}H NMR (500 MHz, $CDCl_3$)

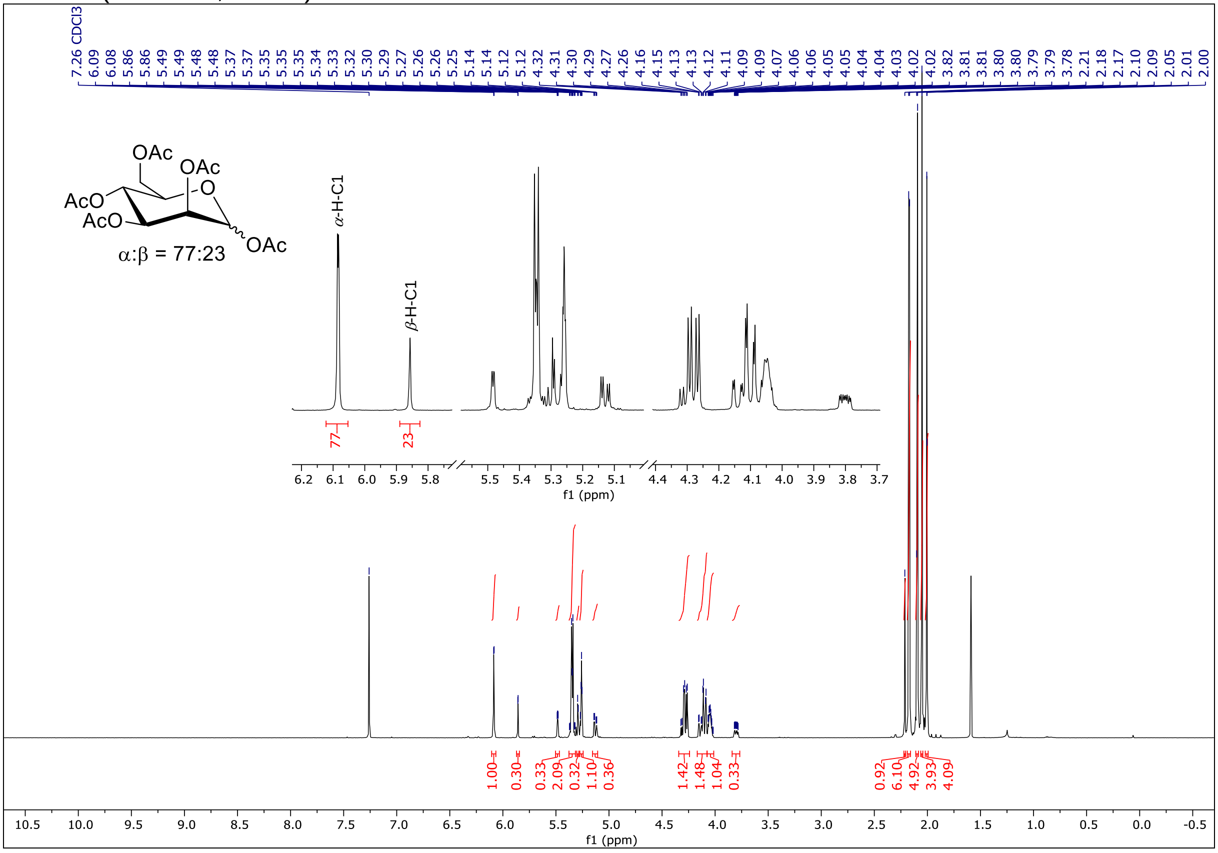

^{13}C NMR (126 MHz, $CDCl_3$)

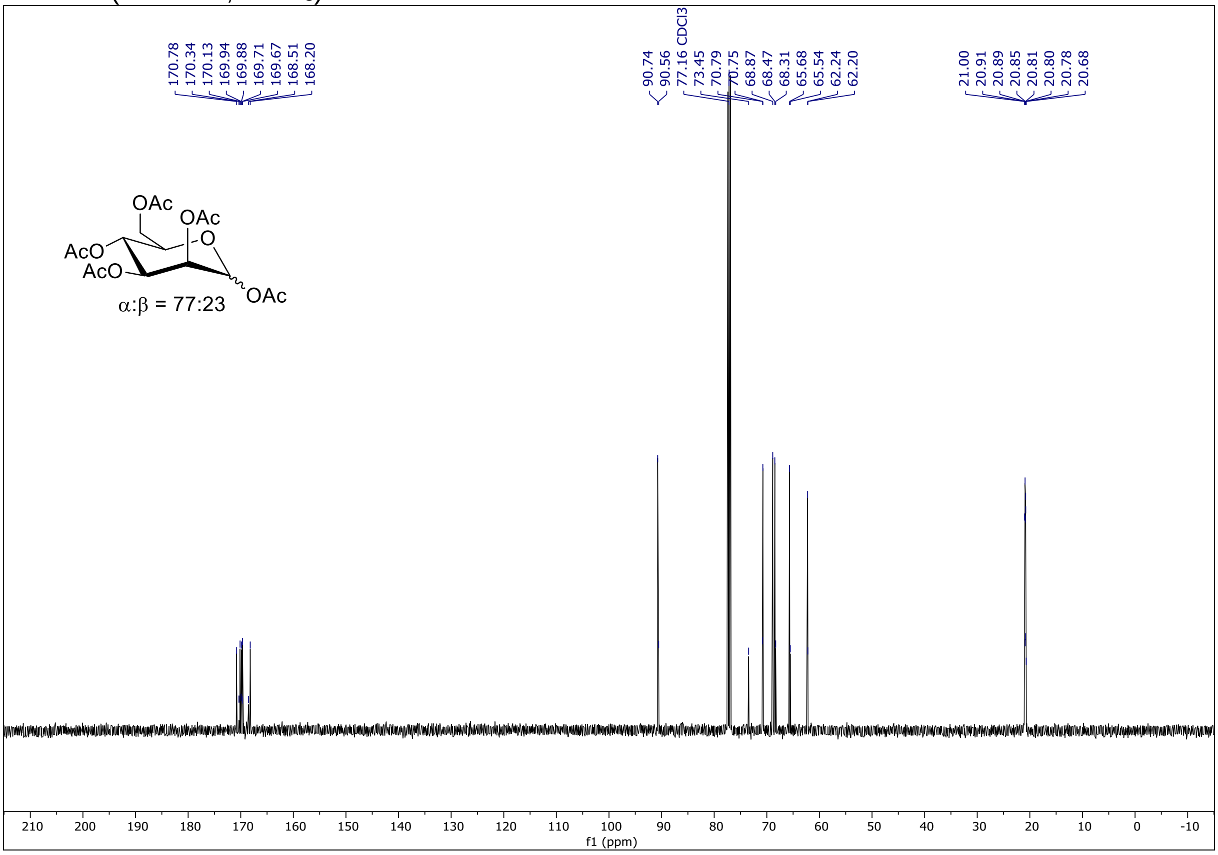

β-**S13**

^{1}H NMR (500 MHz, CDCl$_3$)

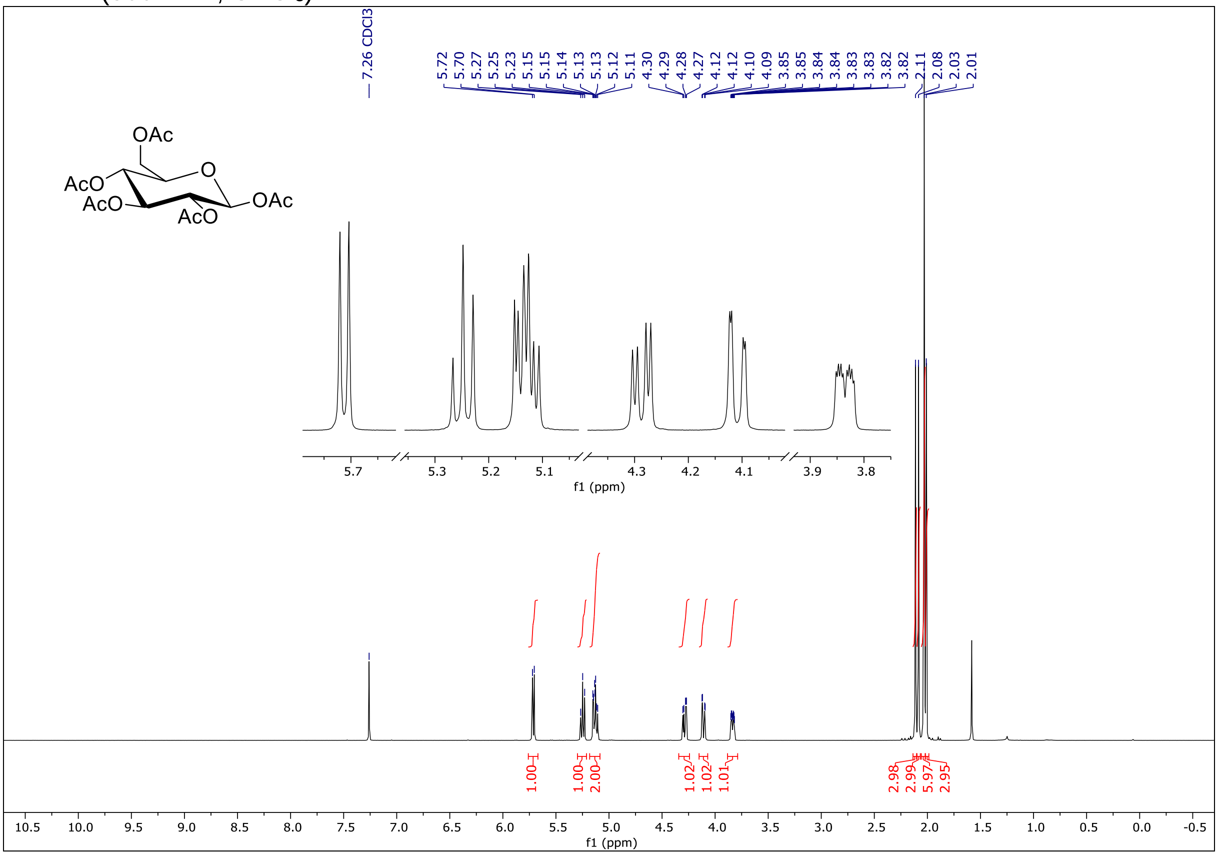

^{13}C NMR (126 MHz, CDCl$_3$)

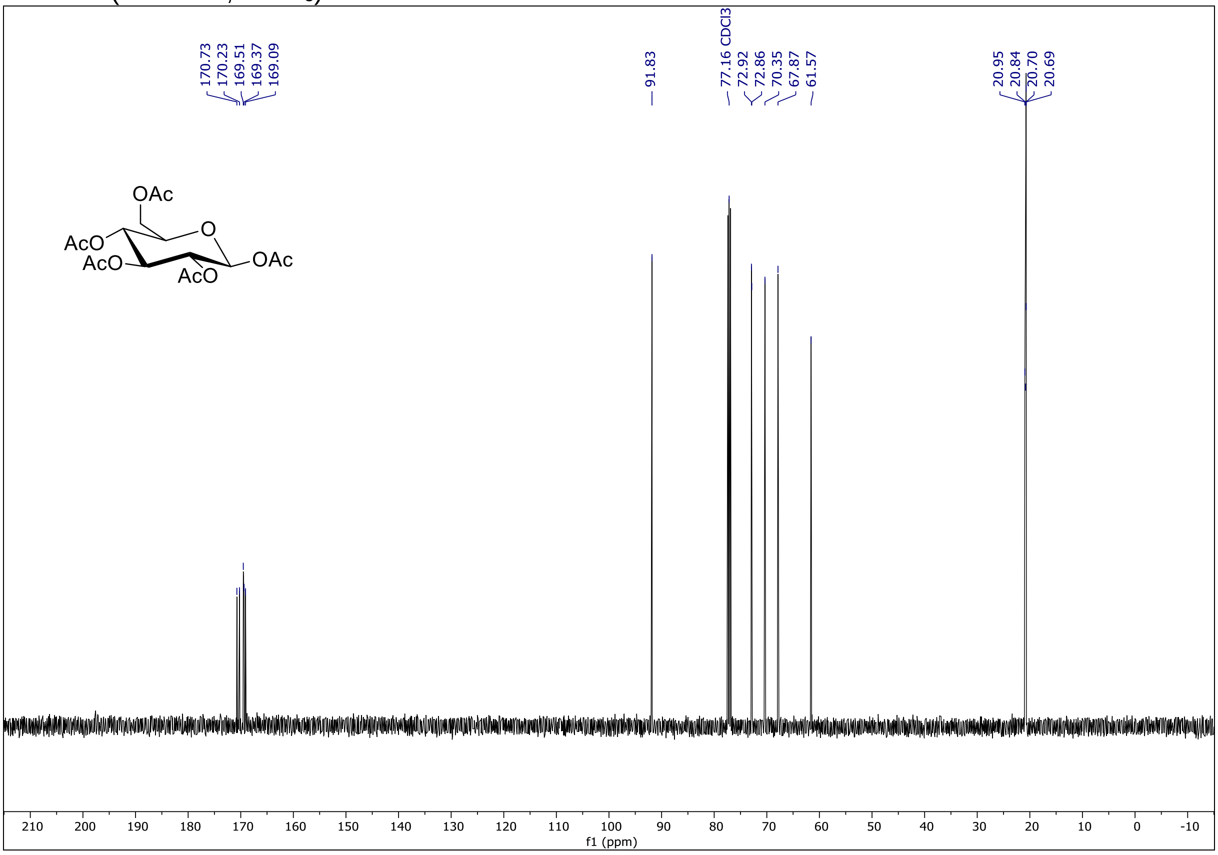

2. INTERMEDIATES ISOLATED IN THE SYNTHESIS OF 2-DEOXY GLUCOSYL FLUORIDE α-19

S1

^{1}H NMR (500 MHz, $CDCl_3$)

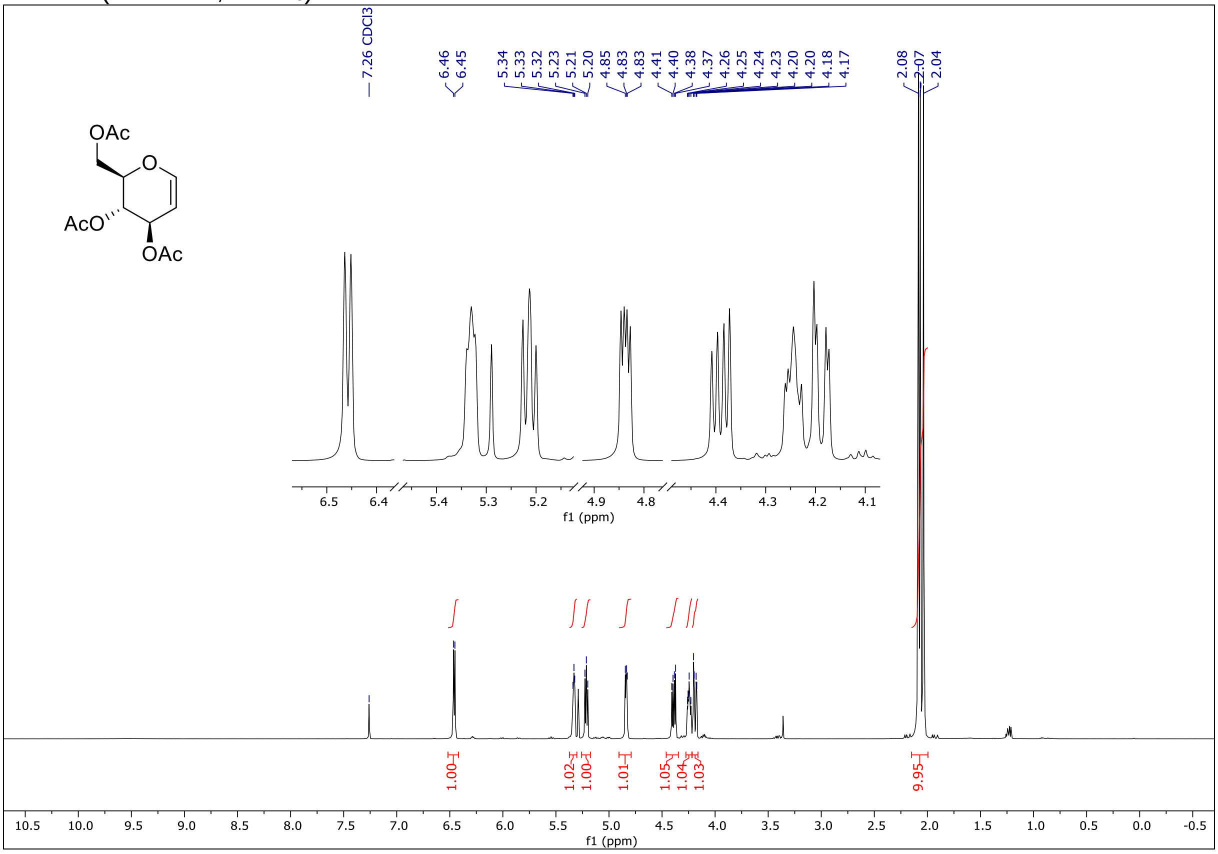

^{13}C NMR (126 MHz, $CDCl_3$)

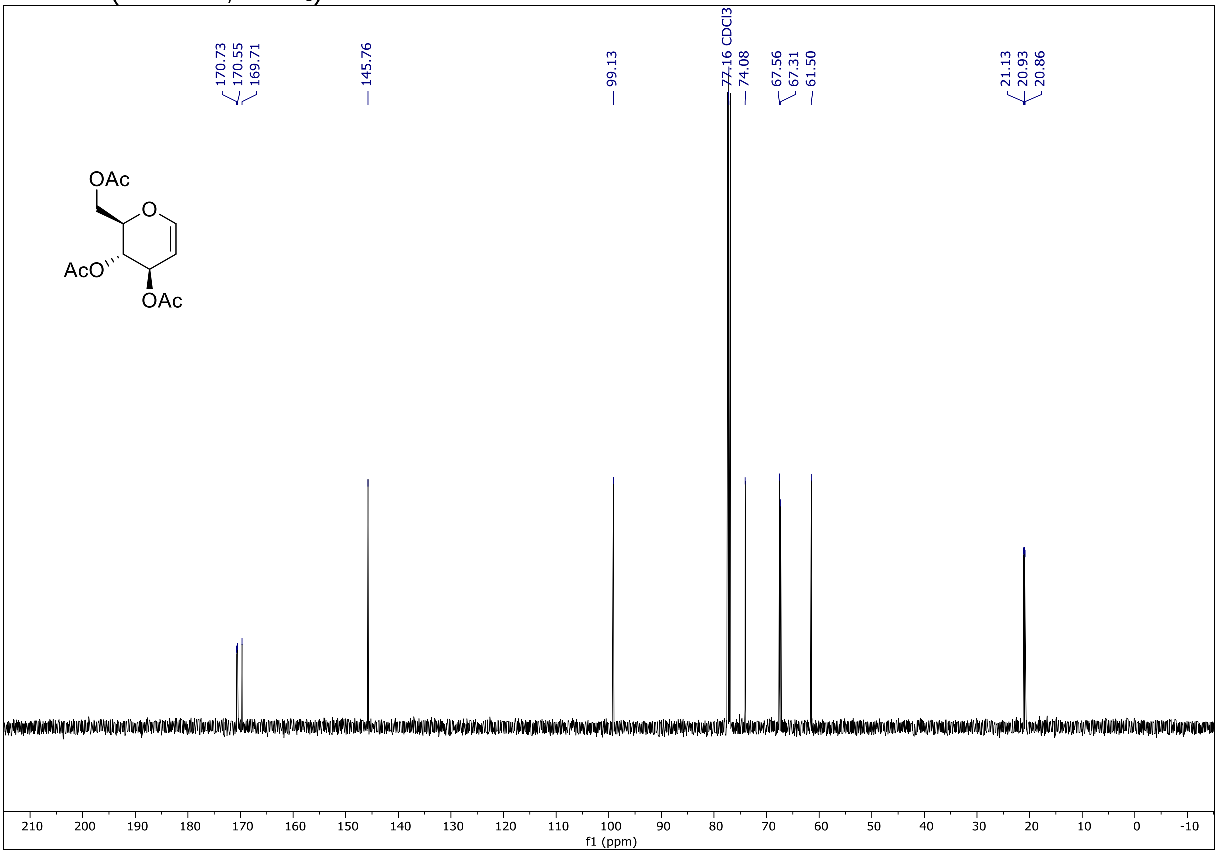

S2

^{1}H NMR (500 MHz, $CDCl_3$)

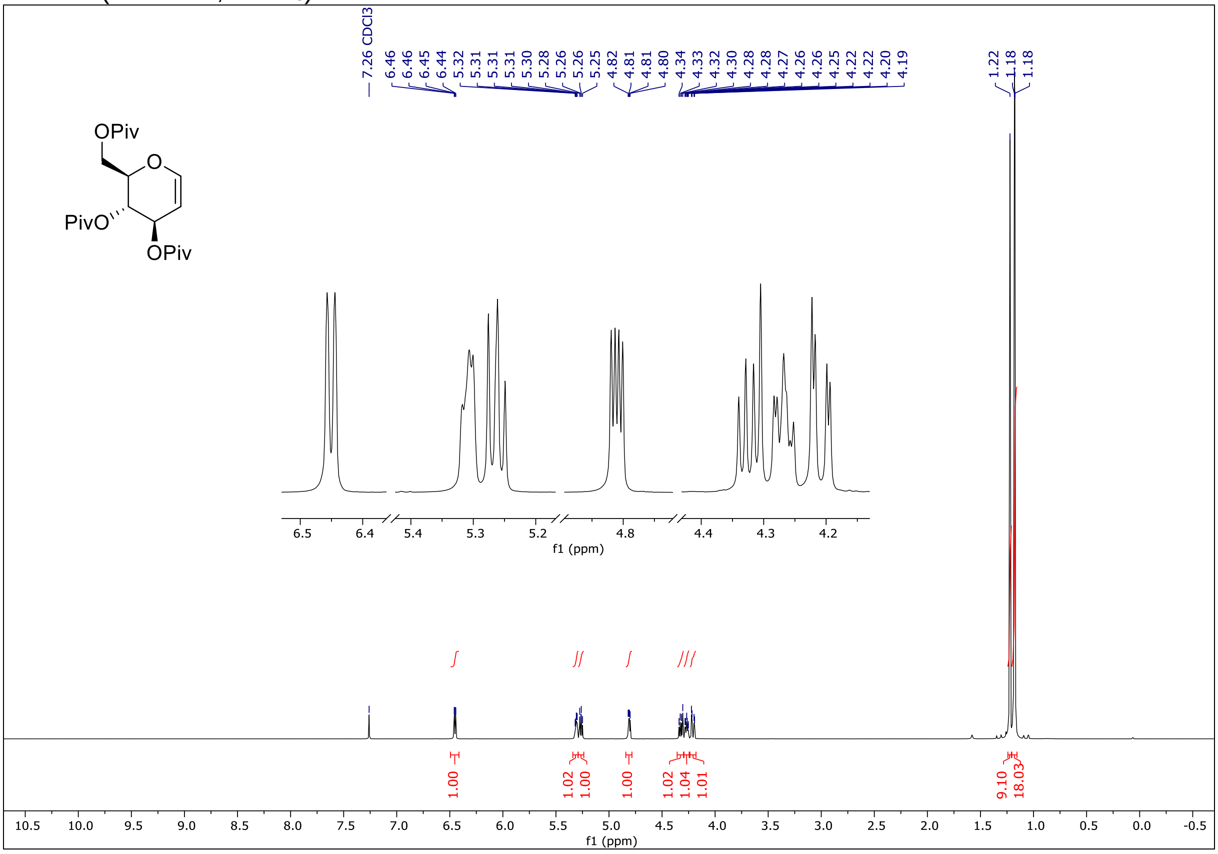

^{13}C NMR (126 MHz, $CDCl_3$)

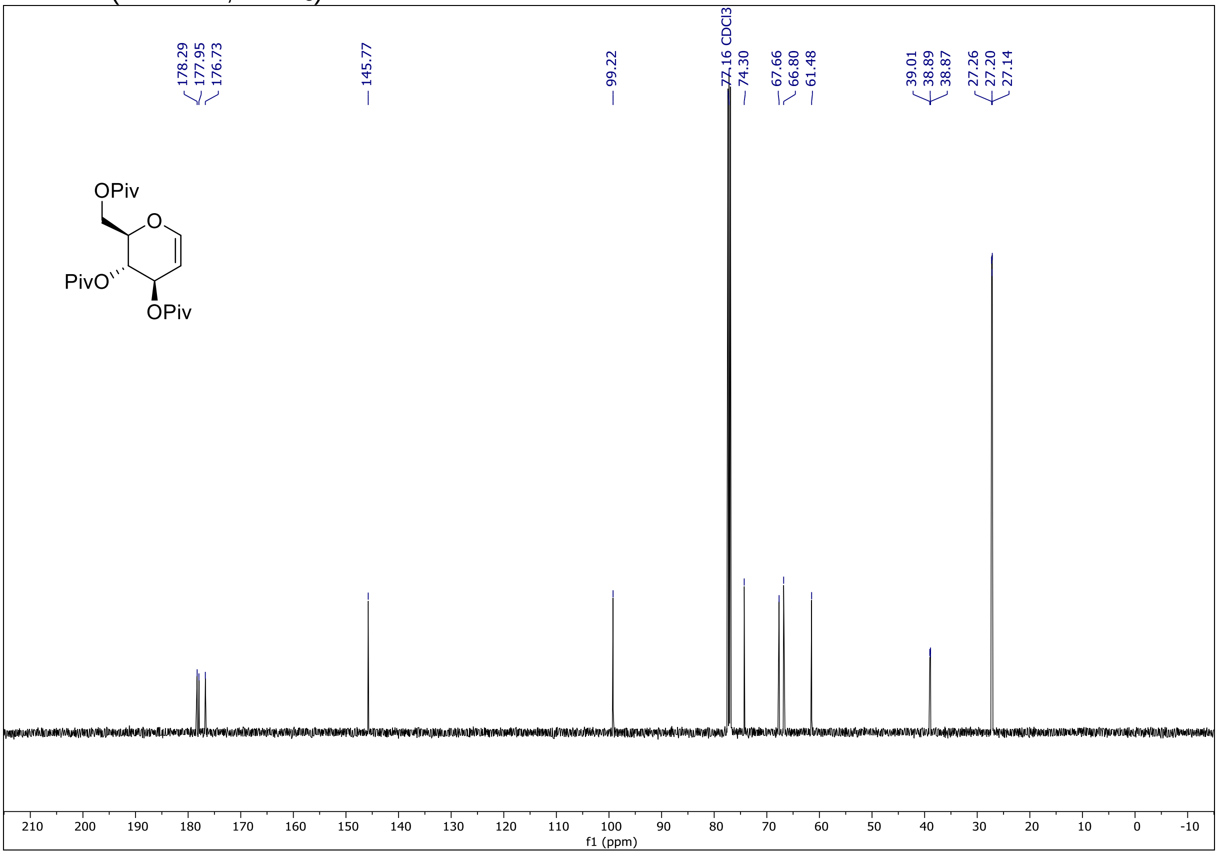

S3

^1H NMR (500 MHz, $CDCl_3$)

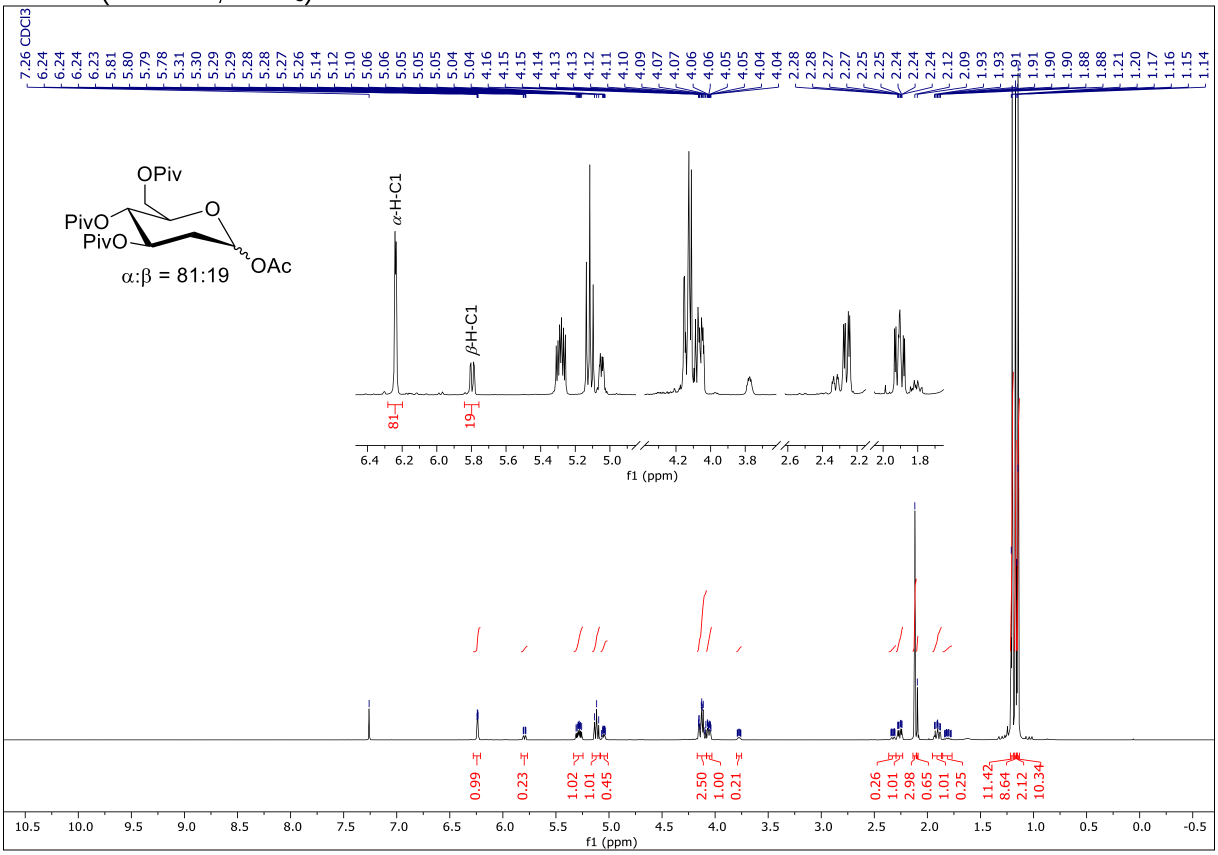

^{13}C NMR (126 MHz, $CDCl_3$)

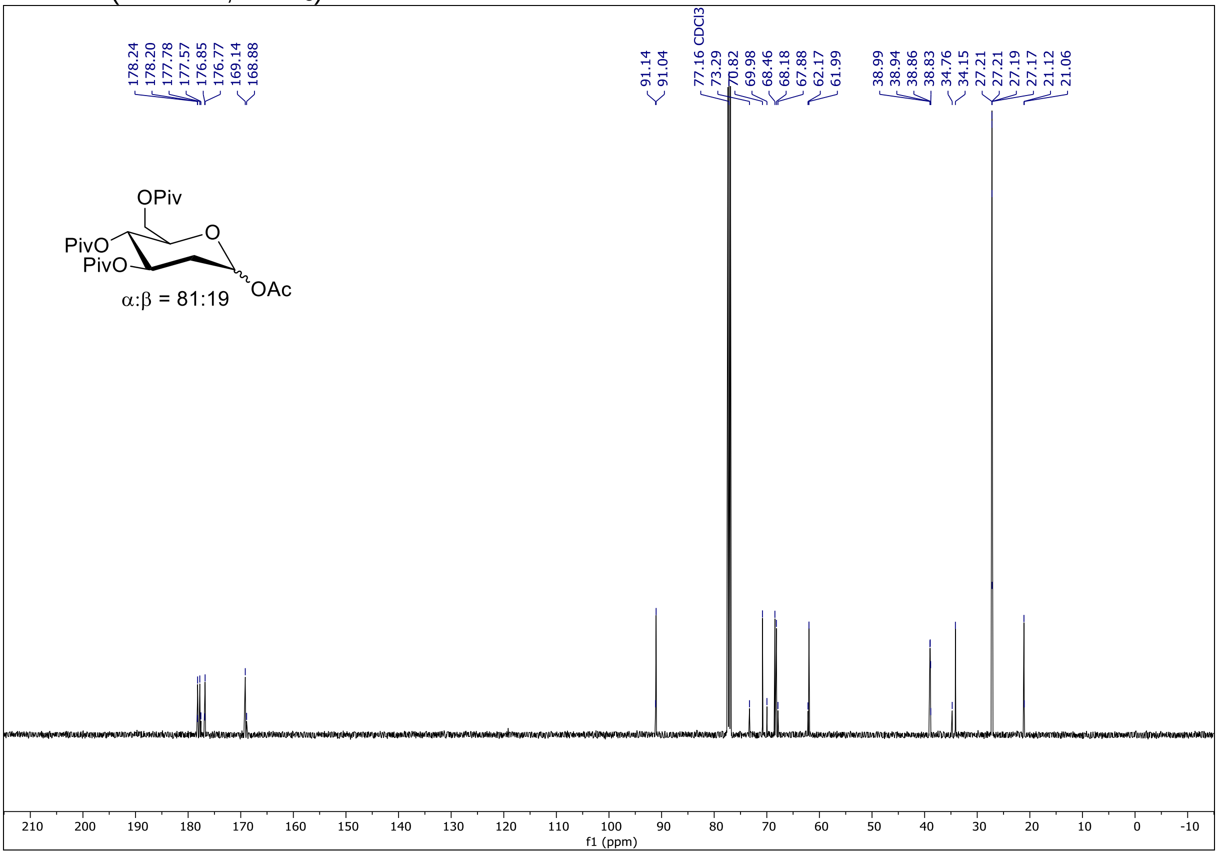

S4

^{1}H NMR (500 MHz, $CDCl_3$)

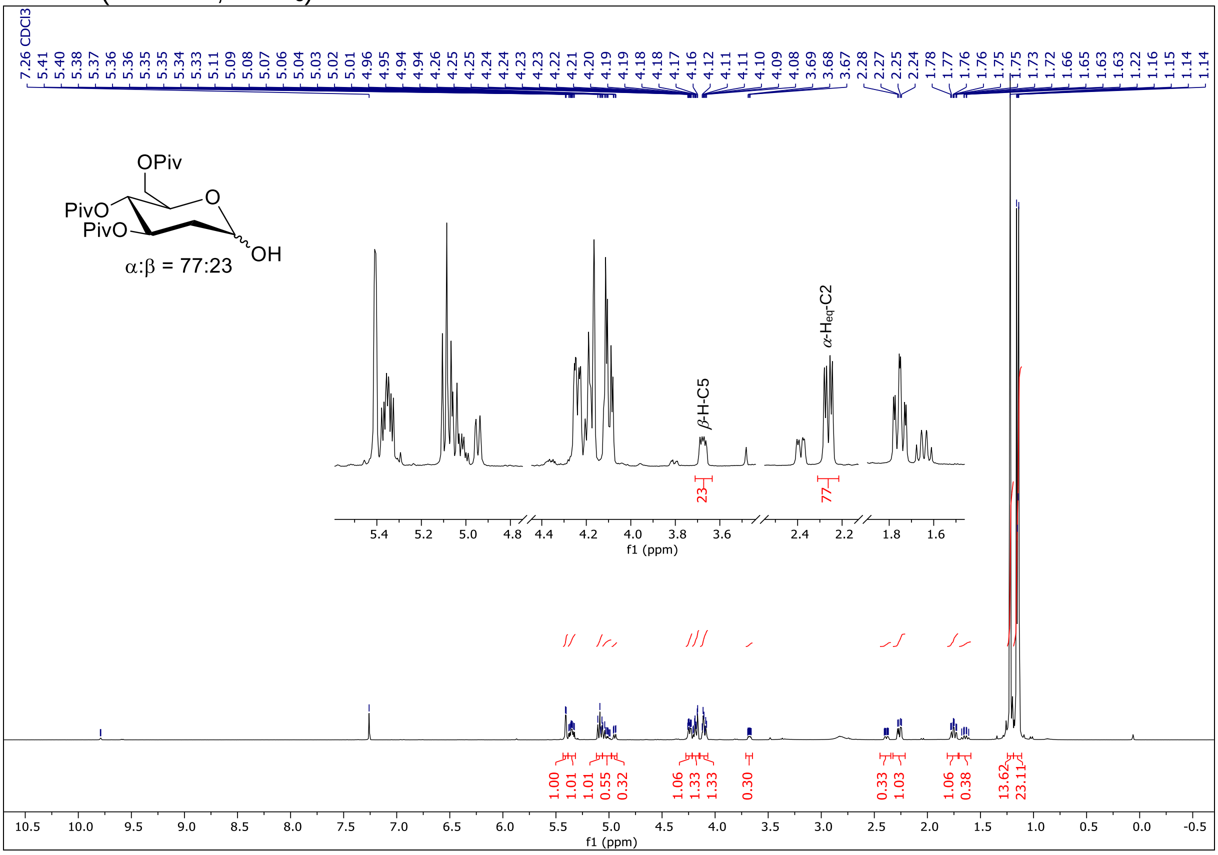

3. TARGET GLYCOSIDES

α-**3a**

^1H NMR (300 MHz, $CDCl_3$)

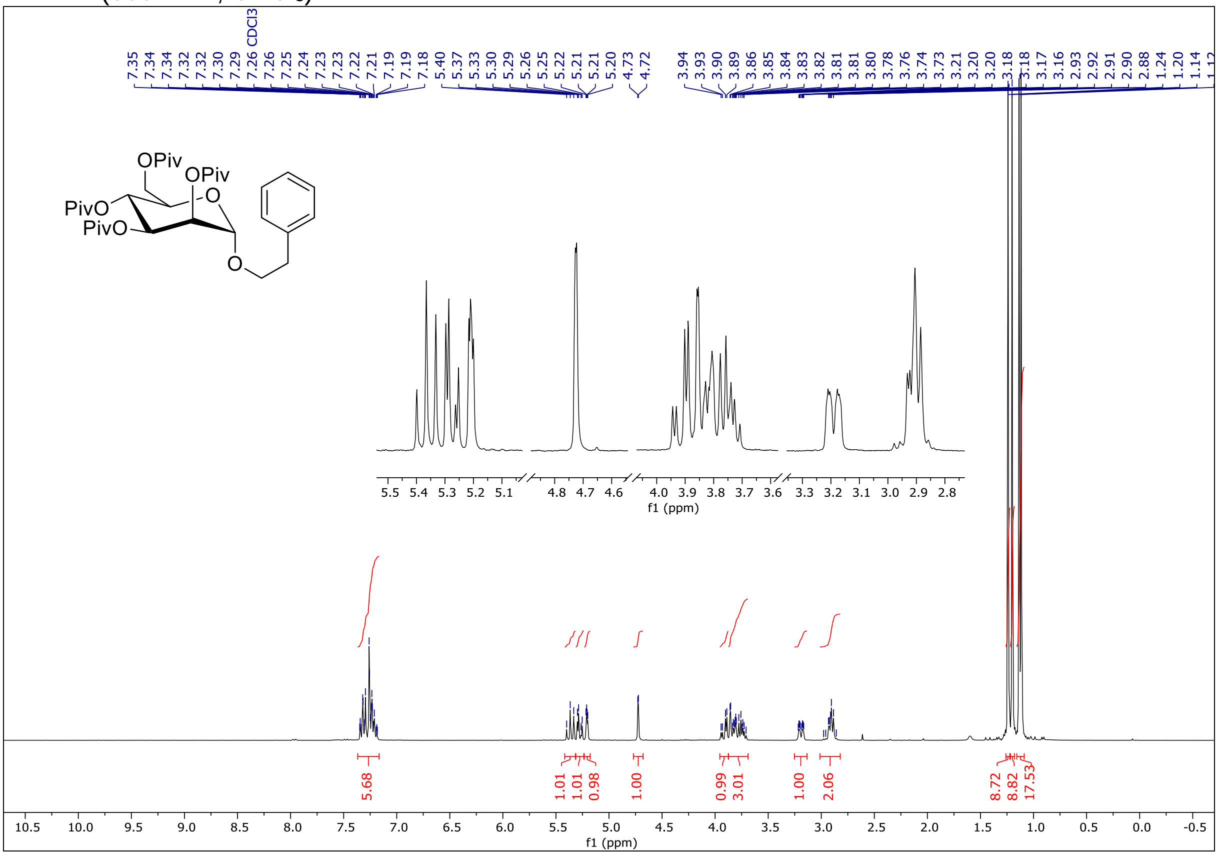

^{13}C NMR (75.5 MHz, $CDCl_3$)

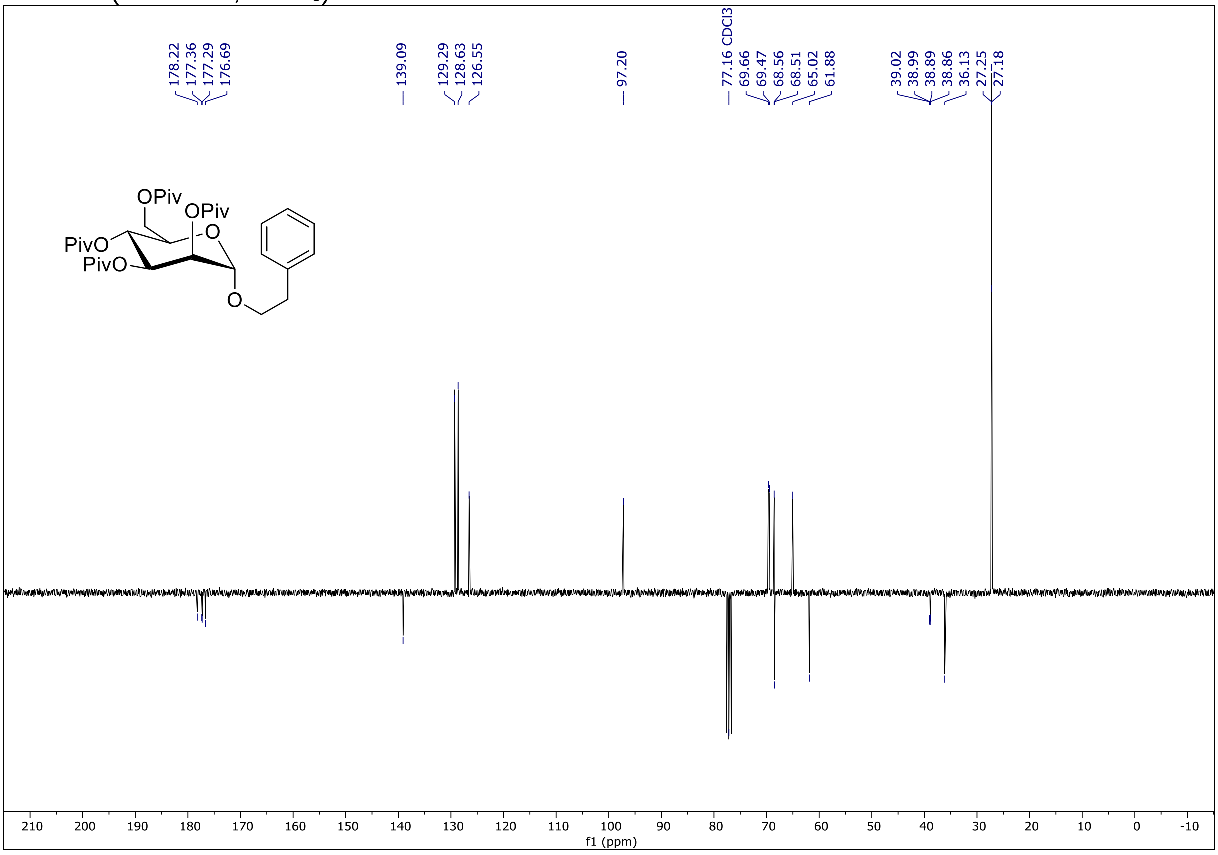

β-**3a**

^{1}H NMR (500 MHz, CDCl$_3$)

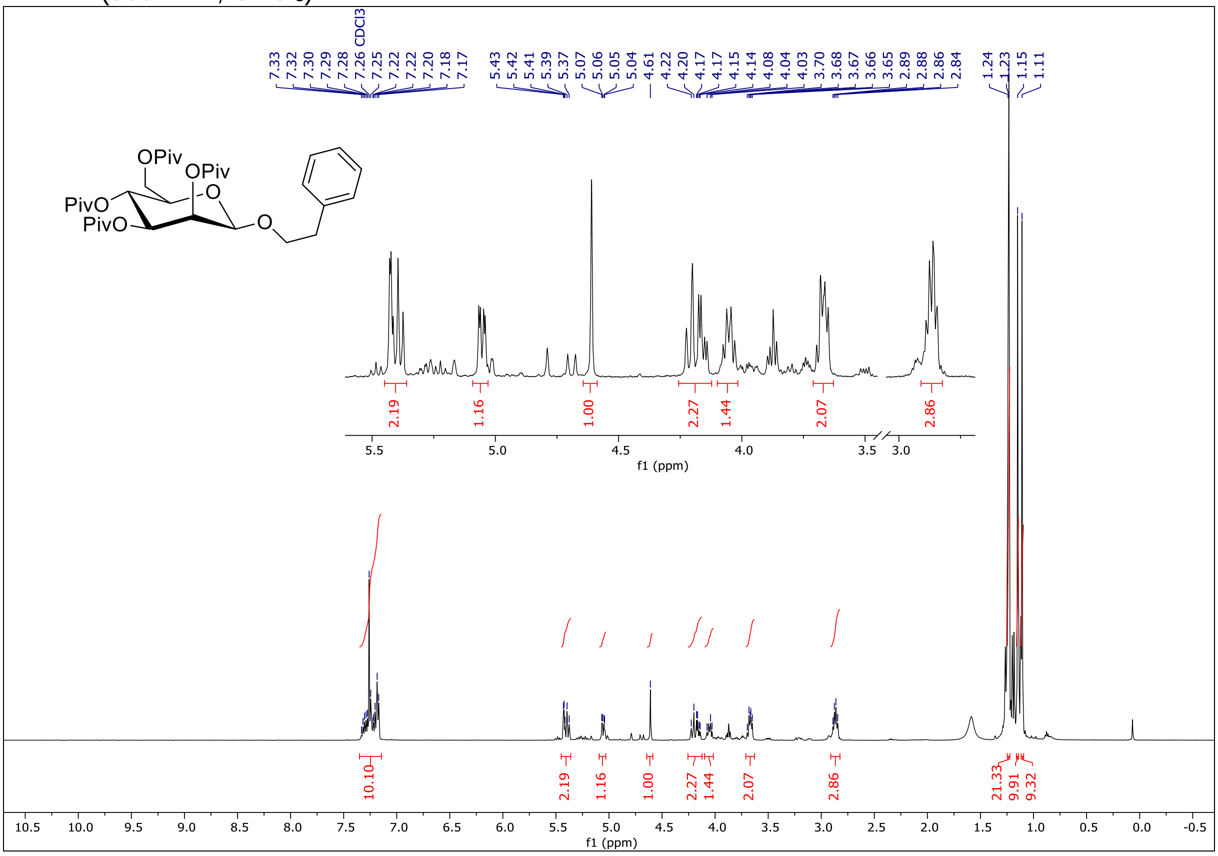

^{13}C NMR (126 MHz, CDCl$_3$)

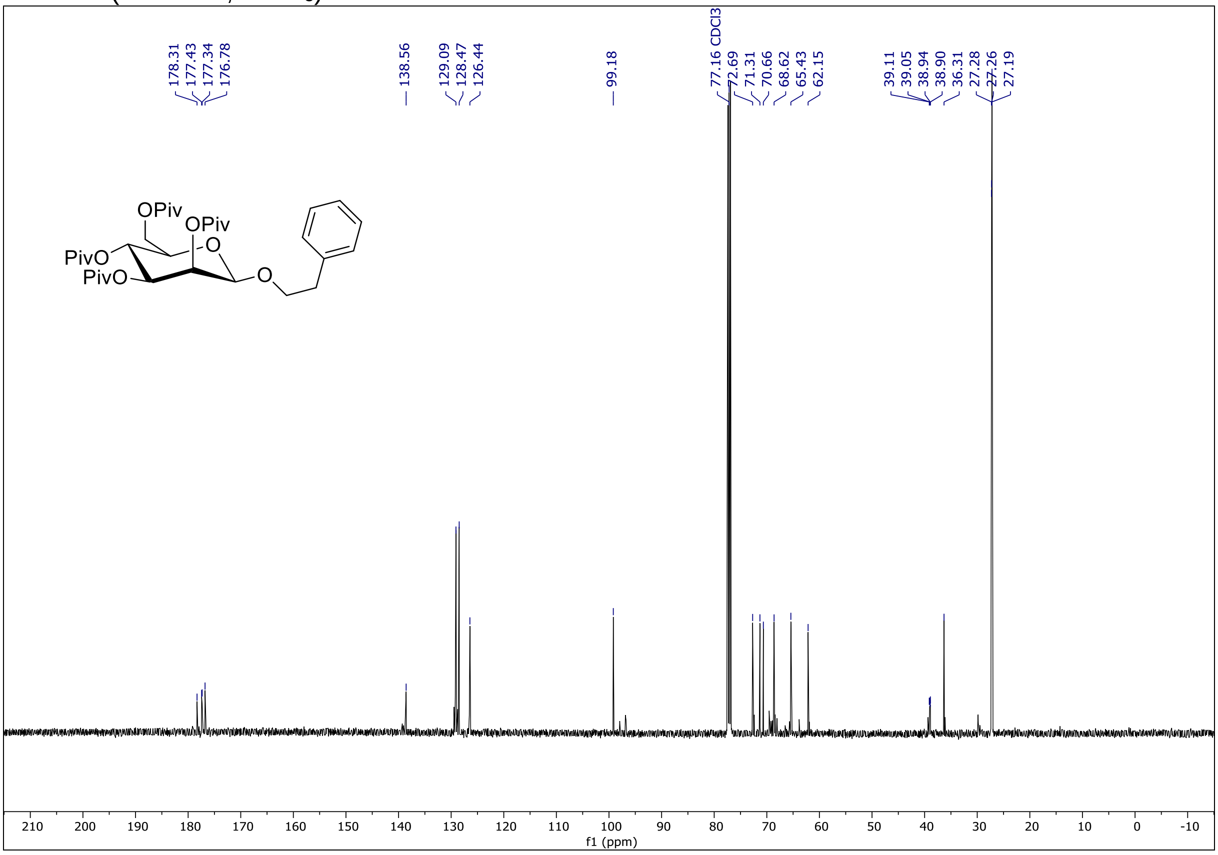

α-**3b**

^{1}H NMR (300 MHz, $CDCl_3$)

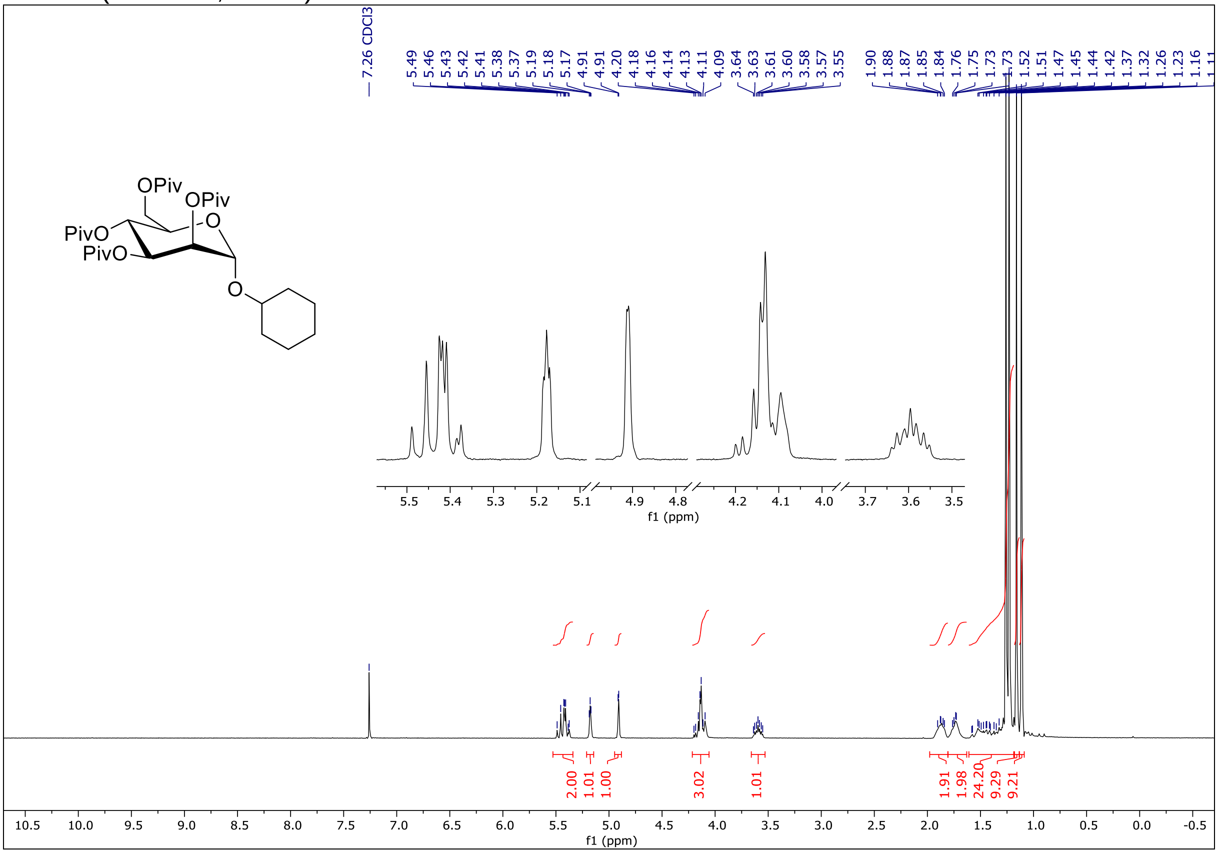

^{13}C NMR (75.5 MHz, $CDCl_3$)

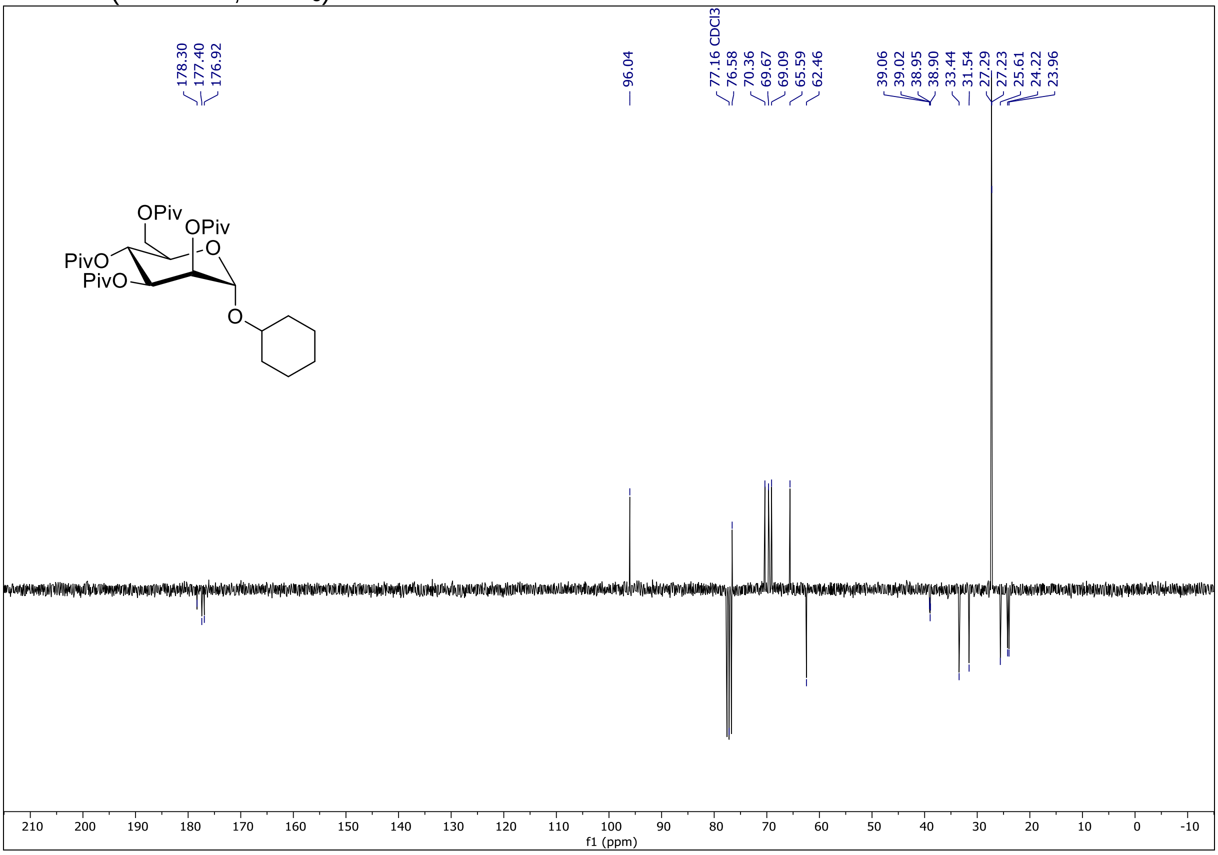

α-3c

^{1}H NMR (300 MHz, $CDCl_3$)

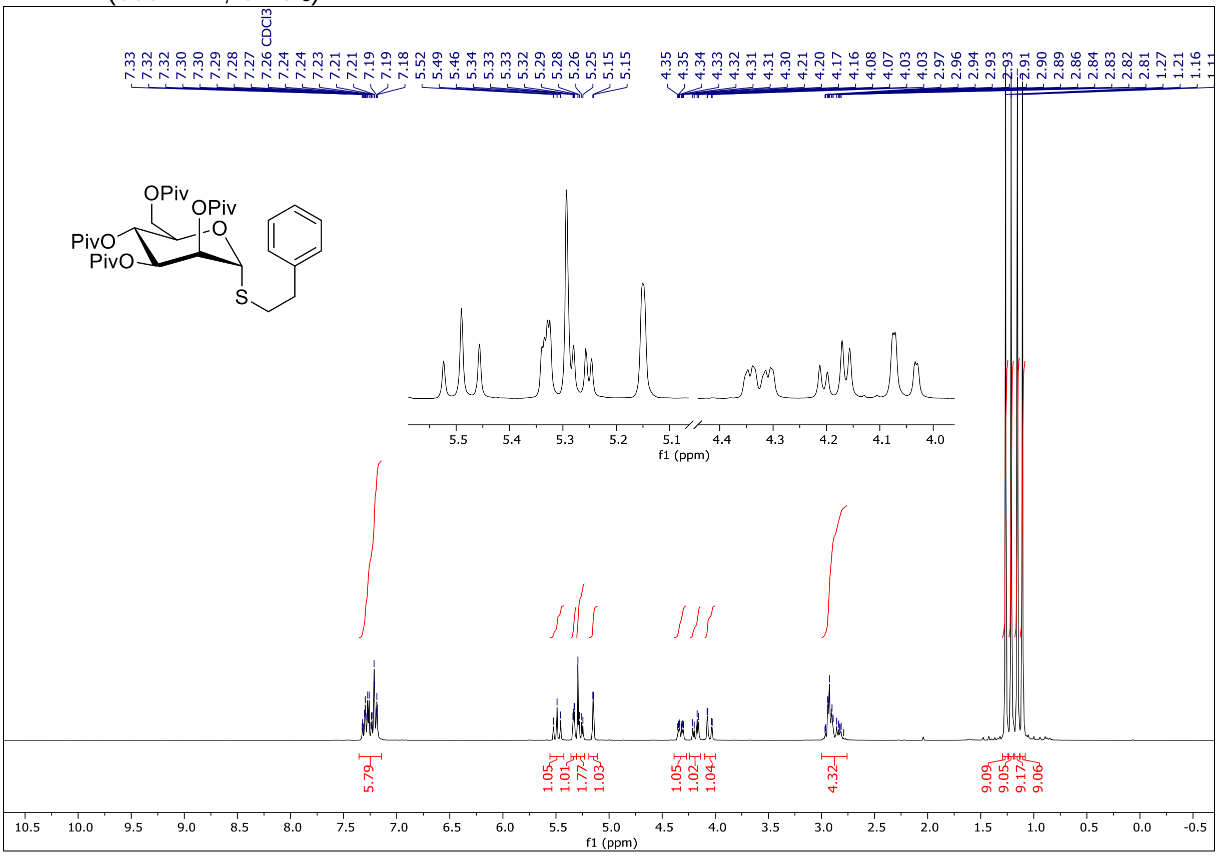

^{13}C NMR (75.5 MHz, $CDCl_3$)

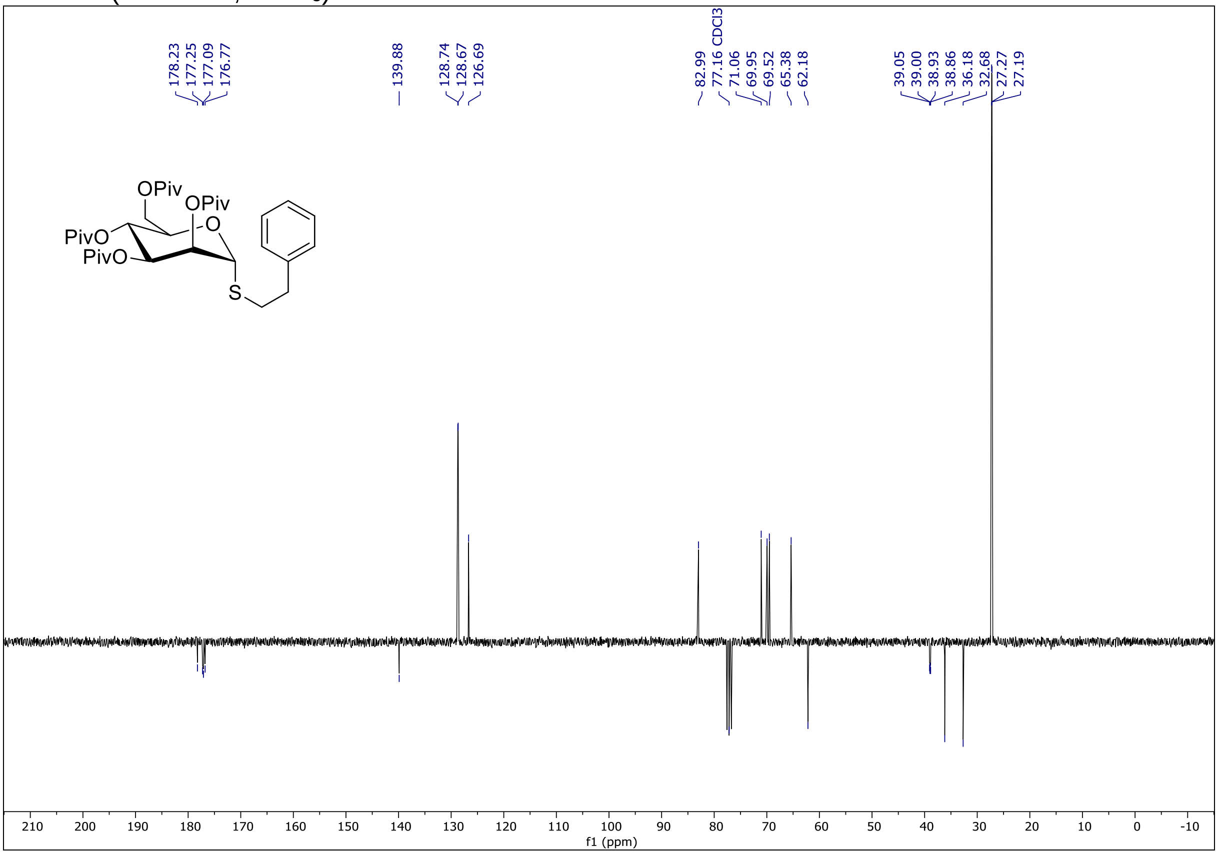

β-**3c**

^{1}H NMR (300 MHz, $CDCl_3$)

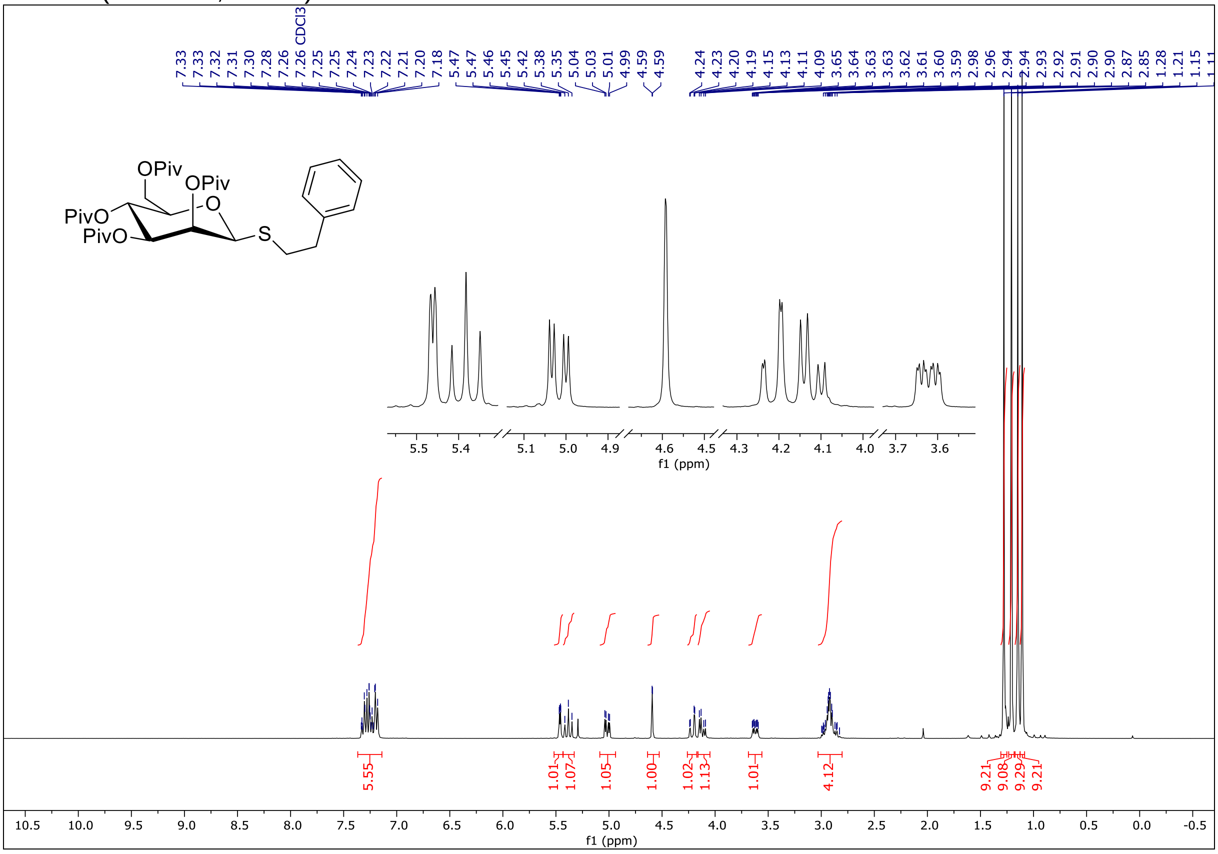

^{13}C NMR (75.5 MHz, $CDCl_3$)

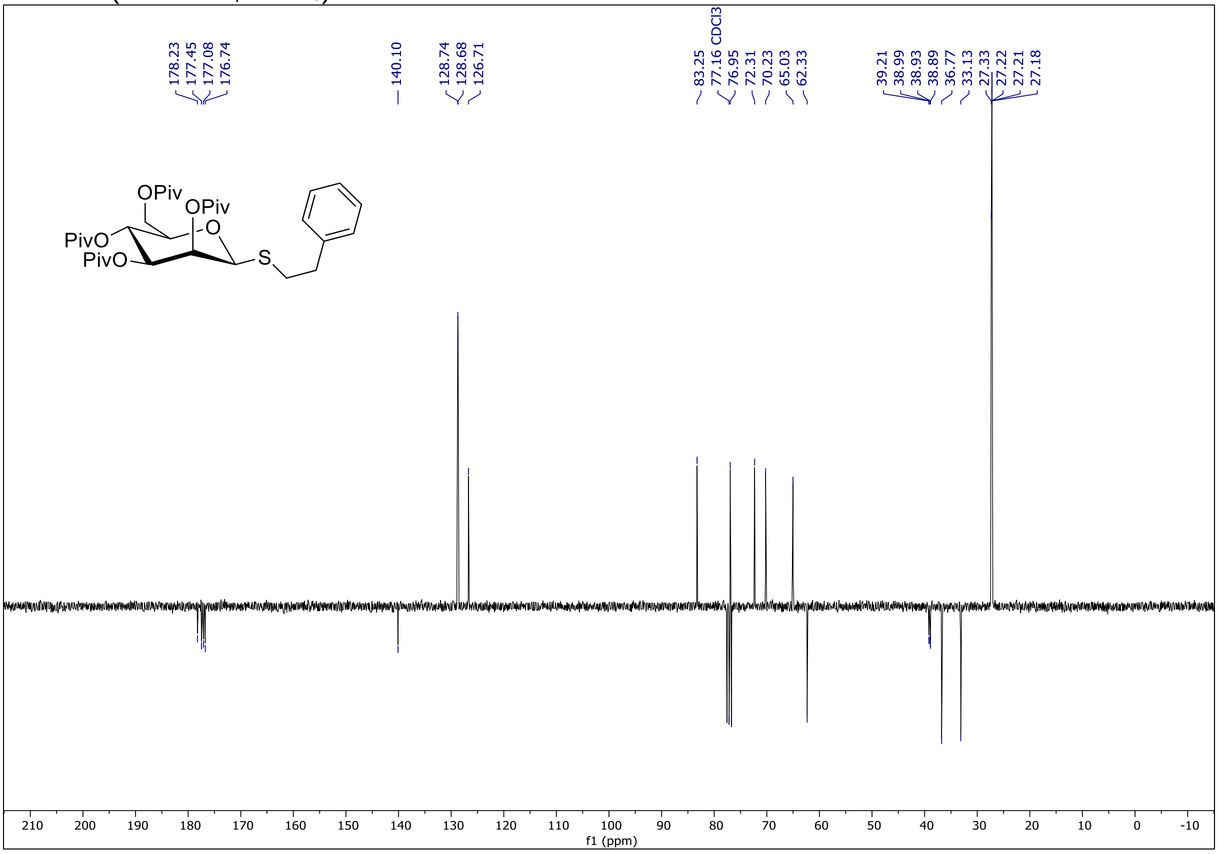

α-**3d**

^{1}H NMR (300 MHz, $CDCl_3$)

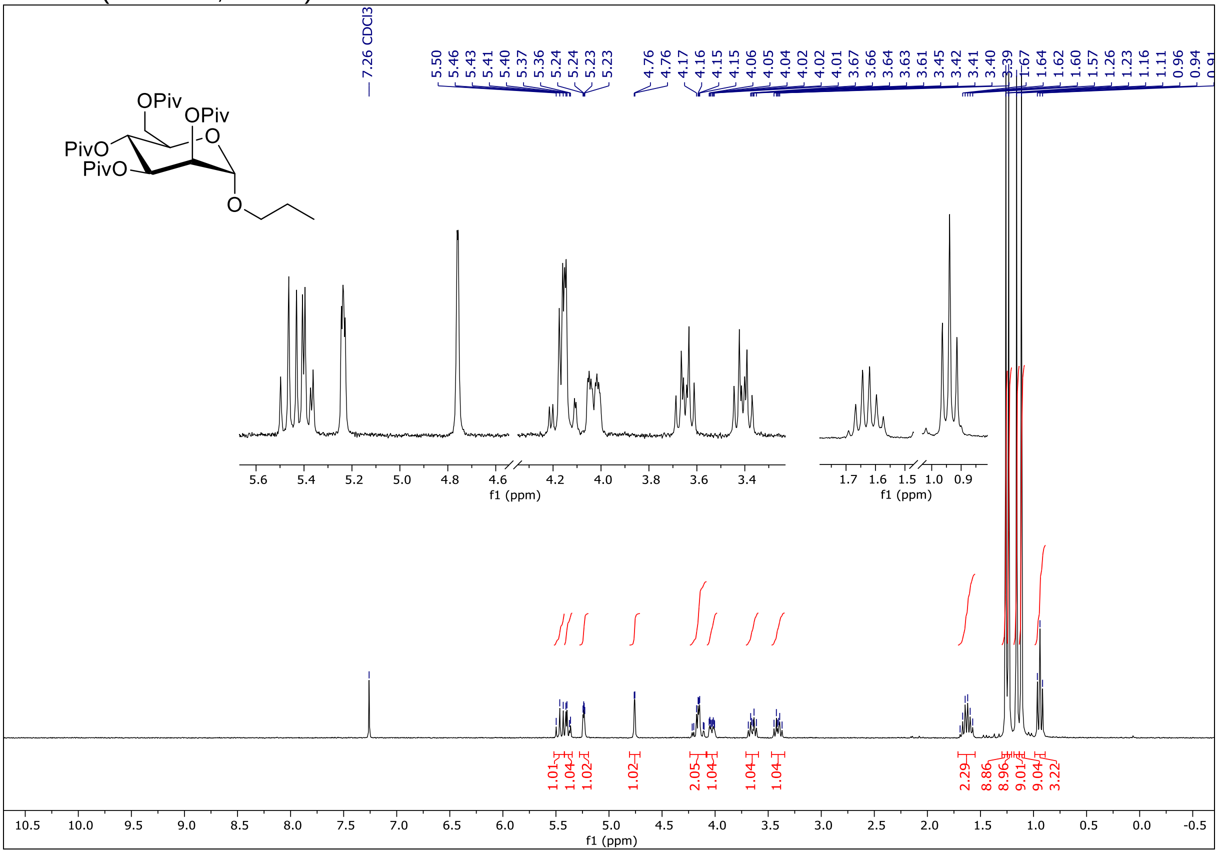

^{13}C NMR (75.5 MHz, $CDCl_3$)

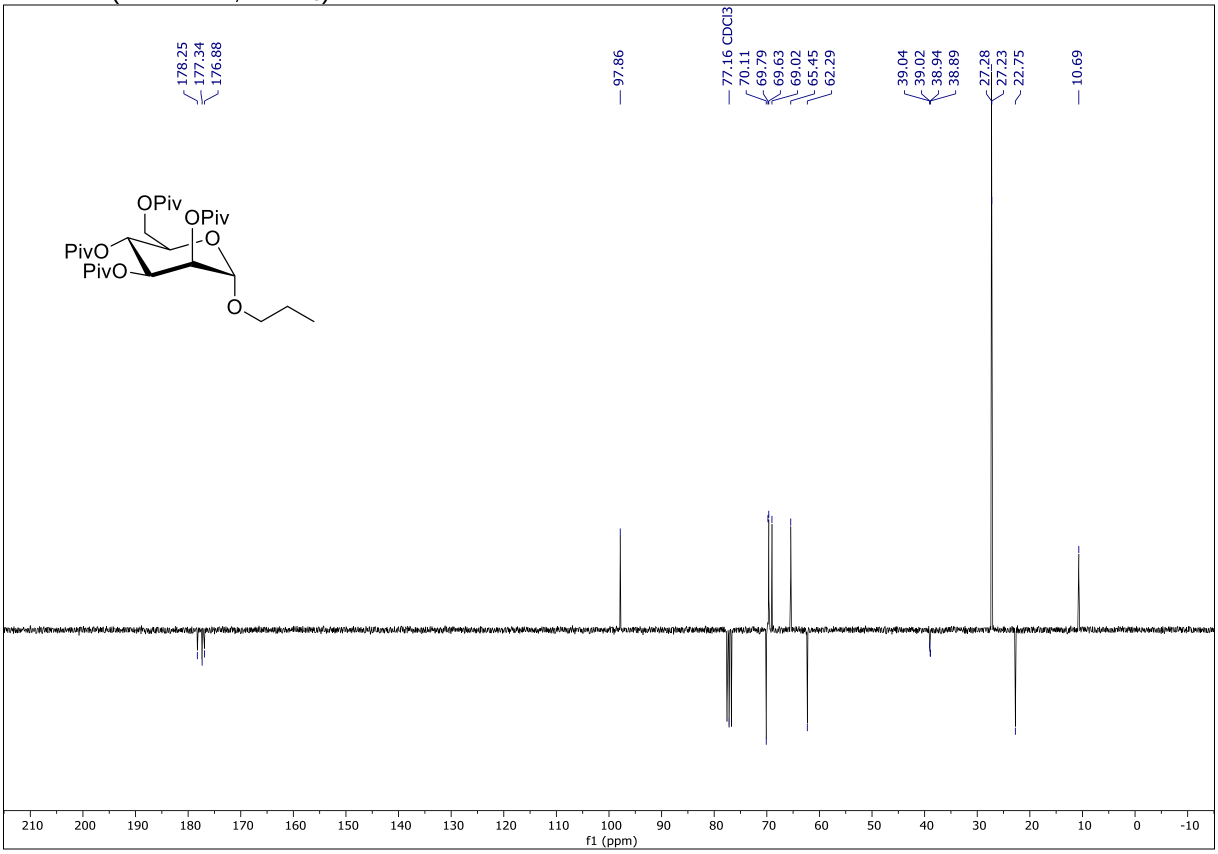

β-3d

^{1}H NMR (500 MHz, $CDCl_3$)

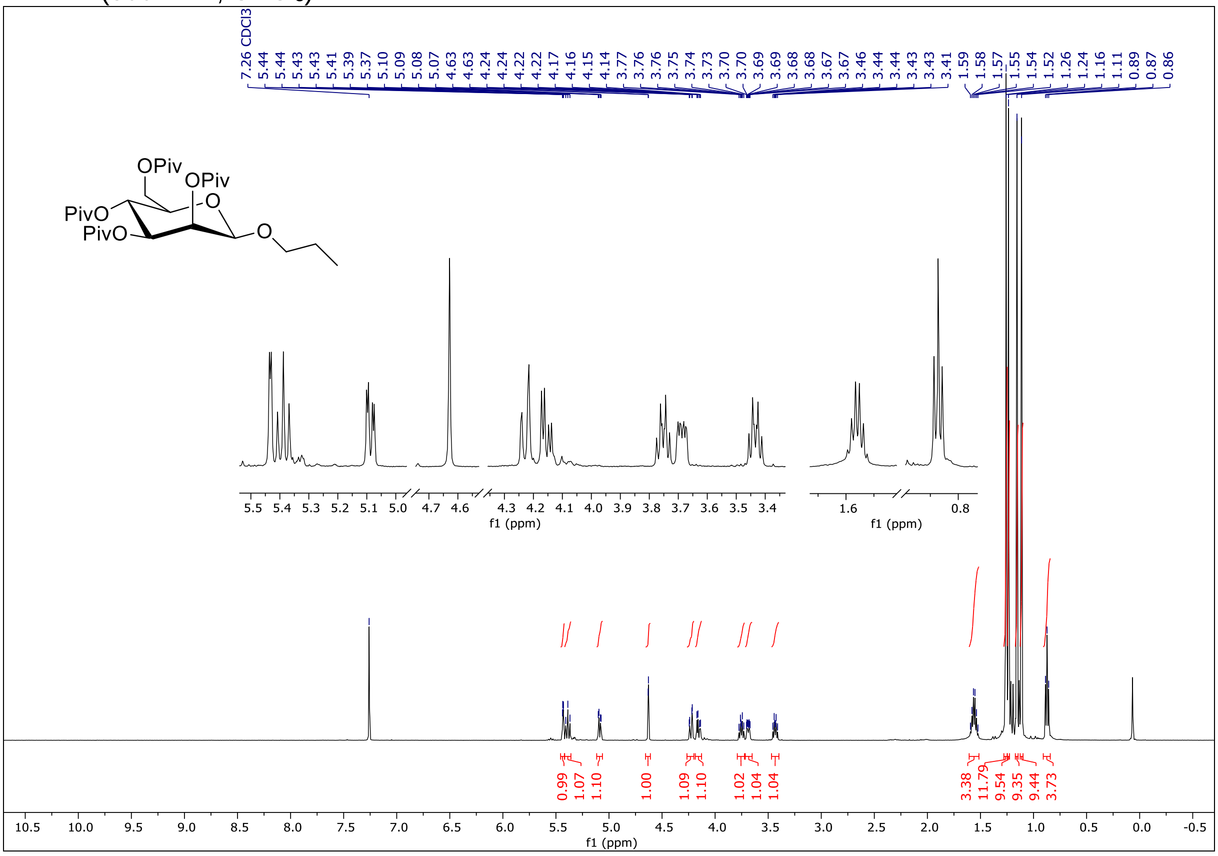

^{13}C NMR (126 MHz, $CDCl_3$)

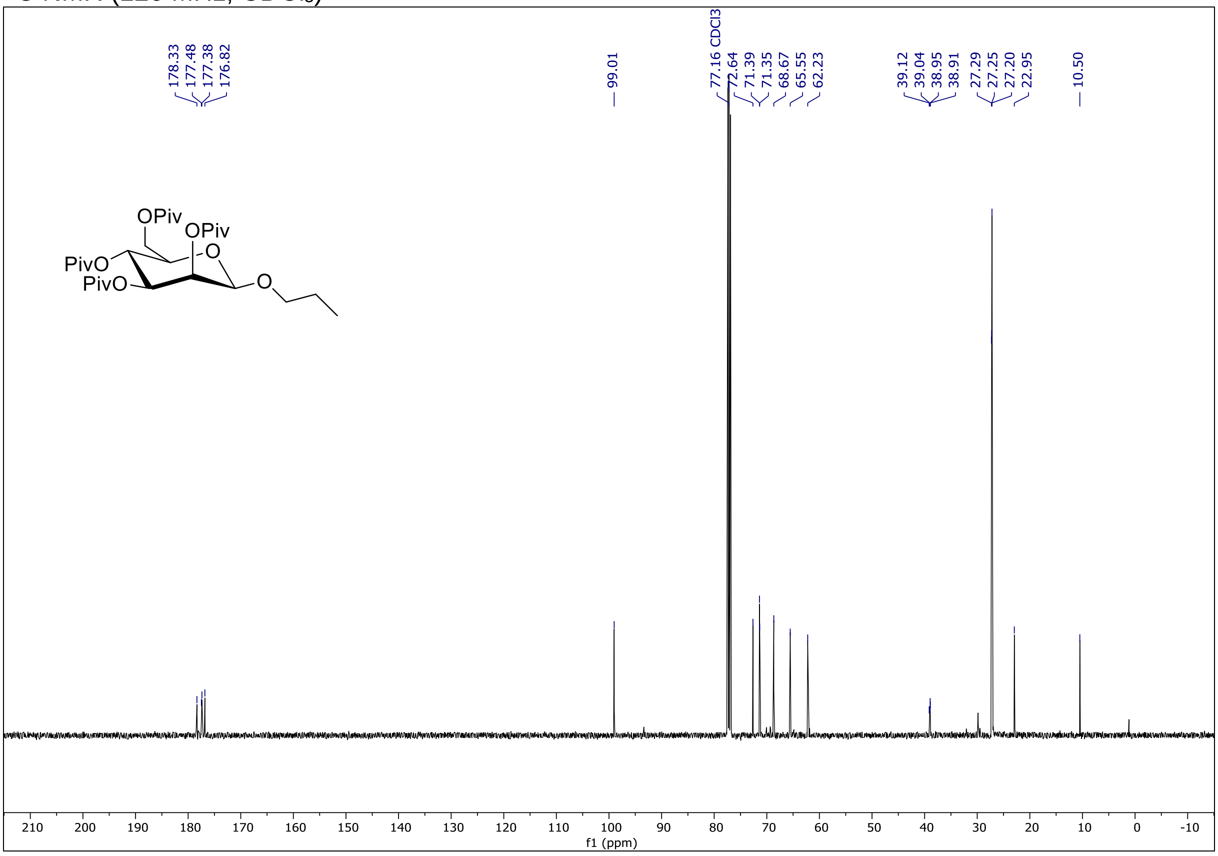

α-**3e**

^{1}H NMR (300 MHz, $CDCl_3$)

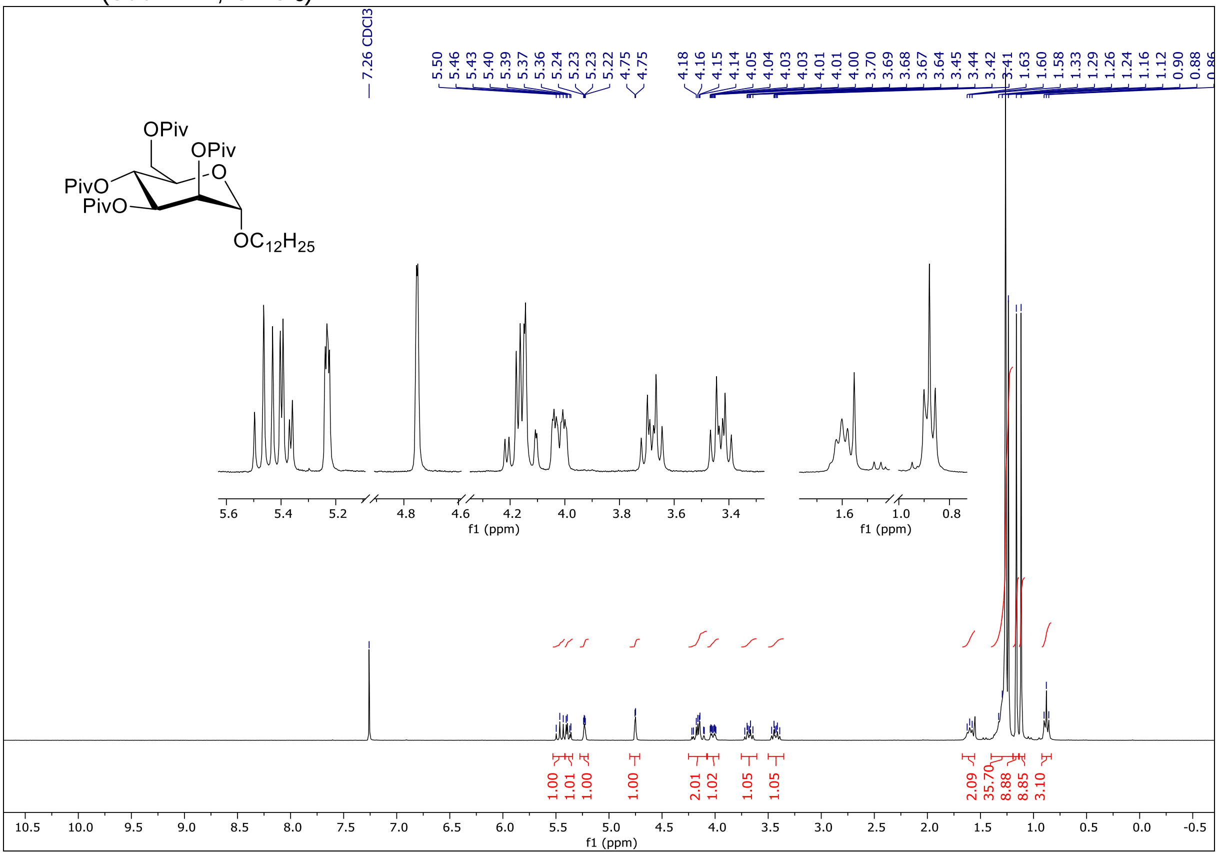

^{13}C NMR (75.5 MHz, $CDCl_3$)

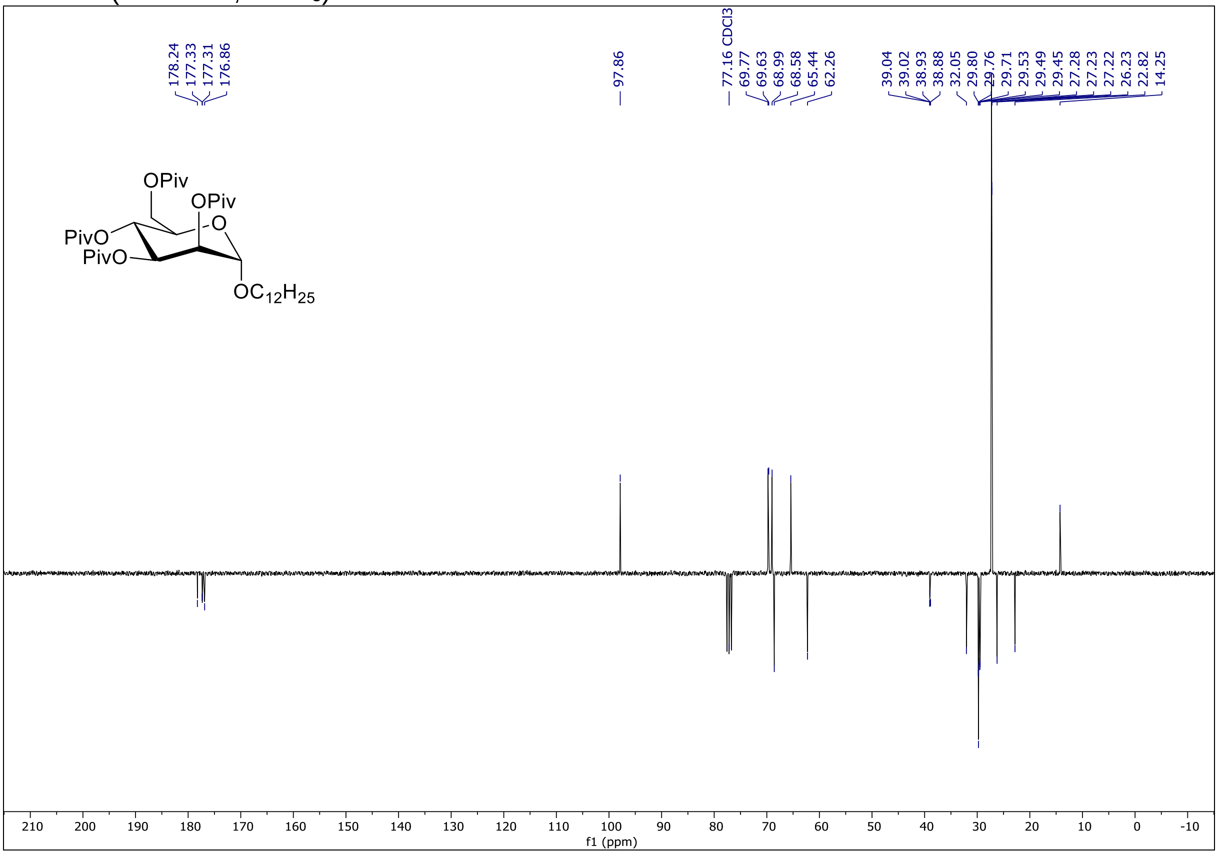

β-**3e**

^{1}H NMR (300 MHz, $CDCl_3$)

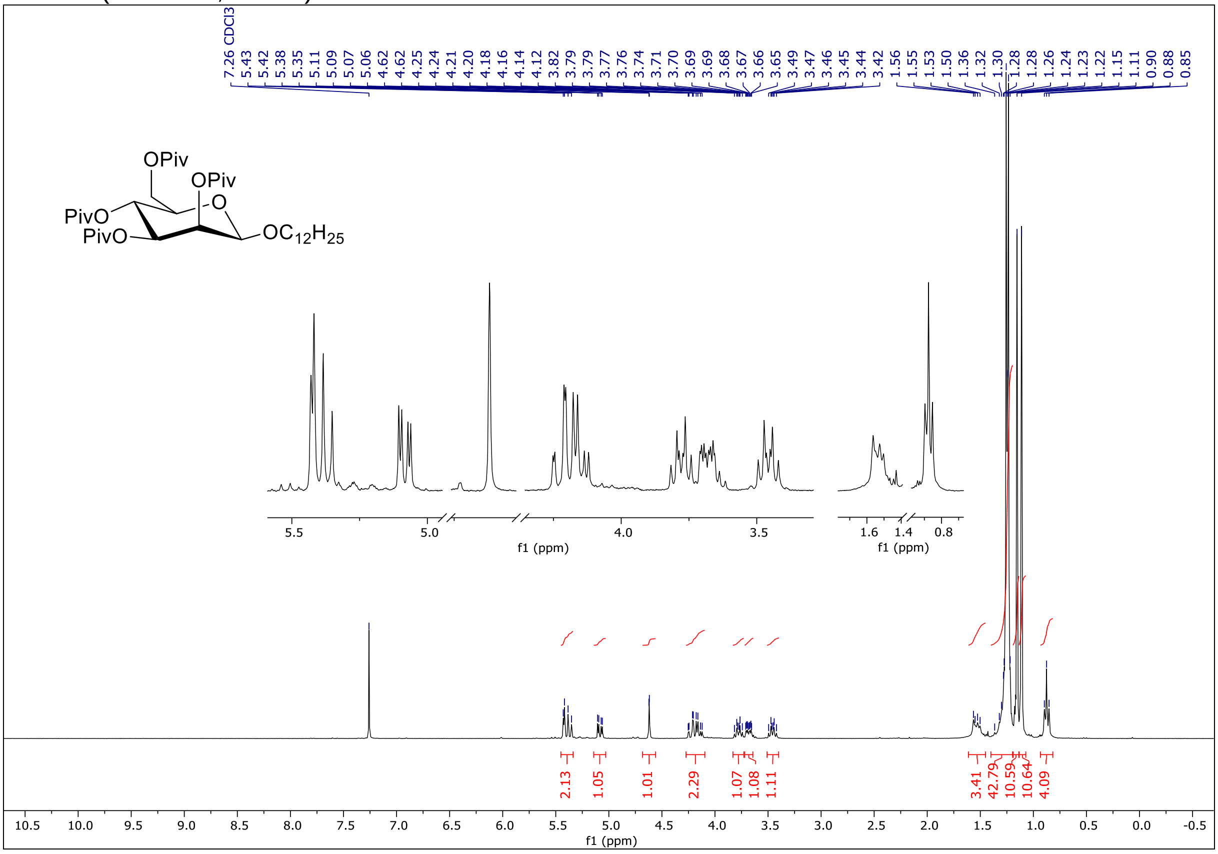

^{13}C NMR (75.5 MHz, $CDCl_3$)

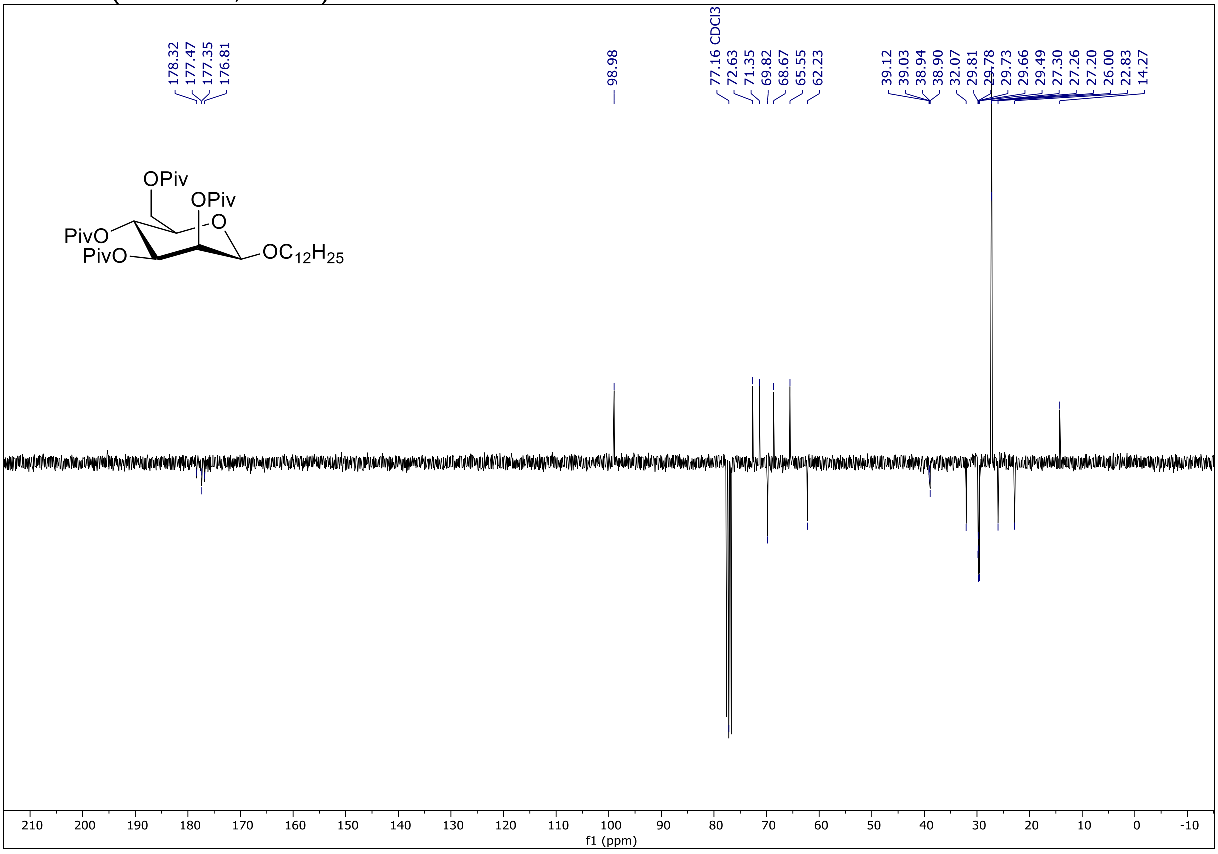

α-3f

^{1}H NMR (500 MHz, CDCl$_3$)

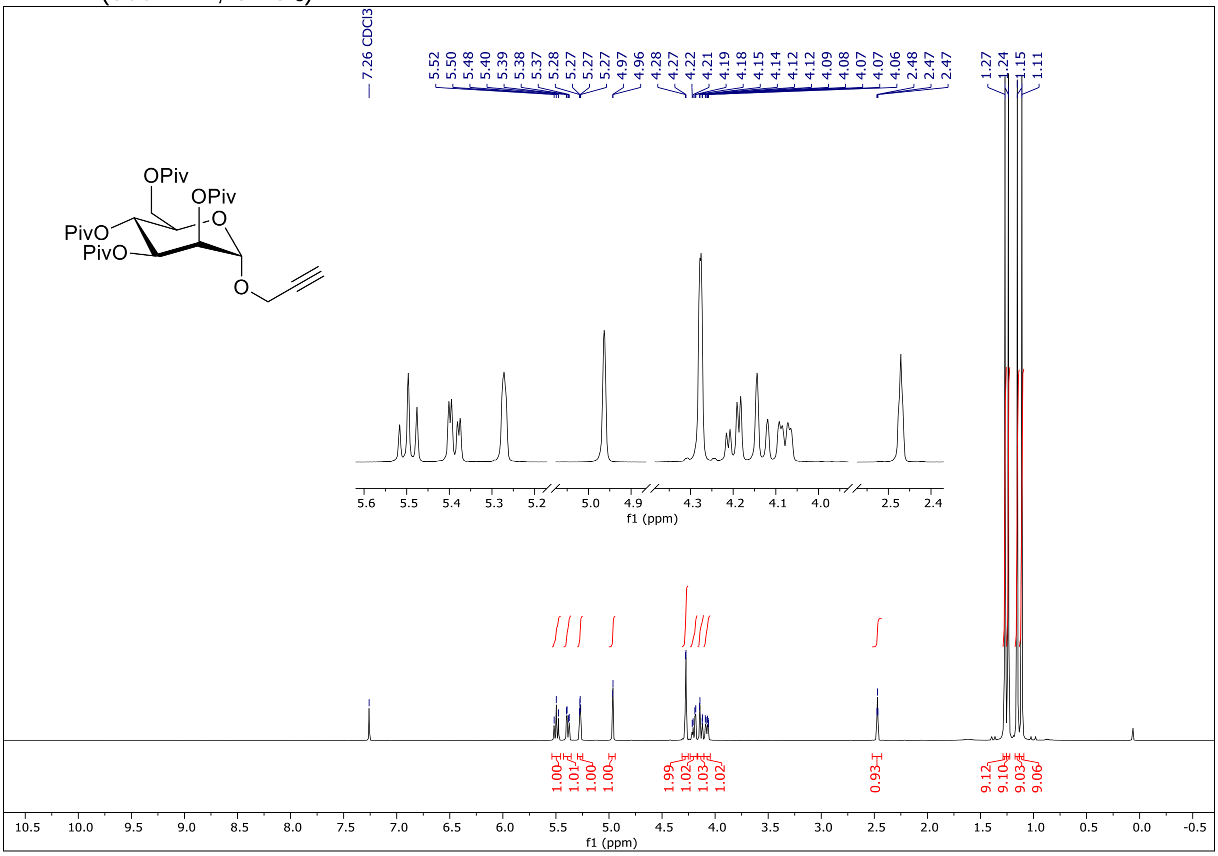

^{13}C NMR (126 MHz, CDCl$_3$)

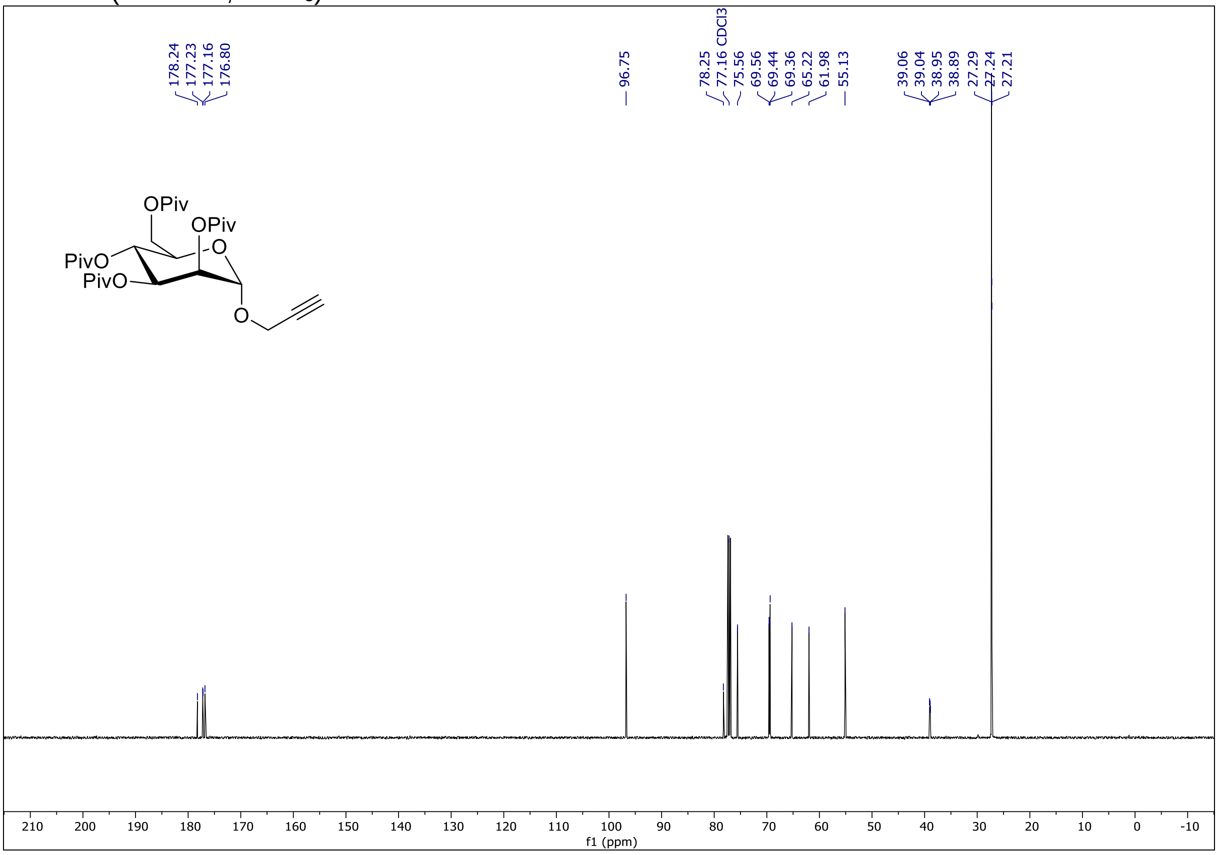

β-3f

^{1}H NMR (500 MHz, $CDCl_3$)

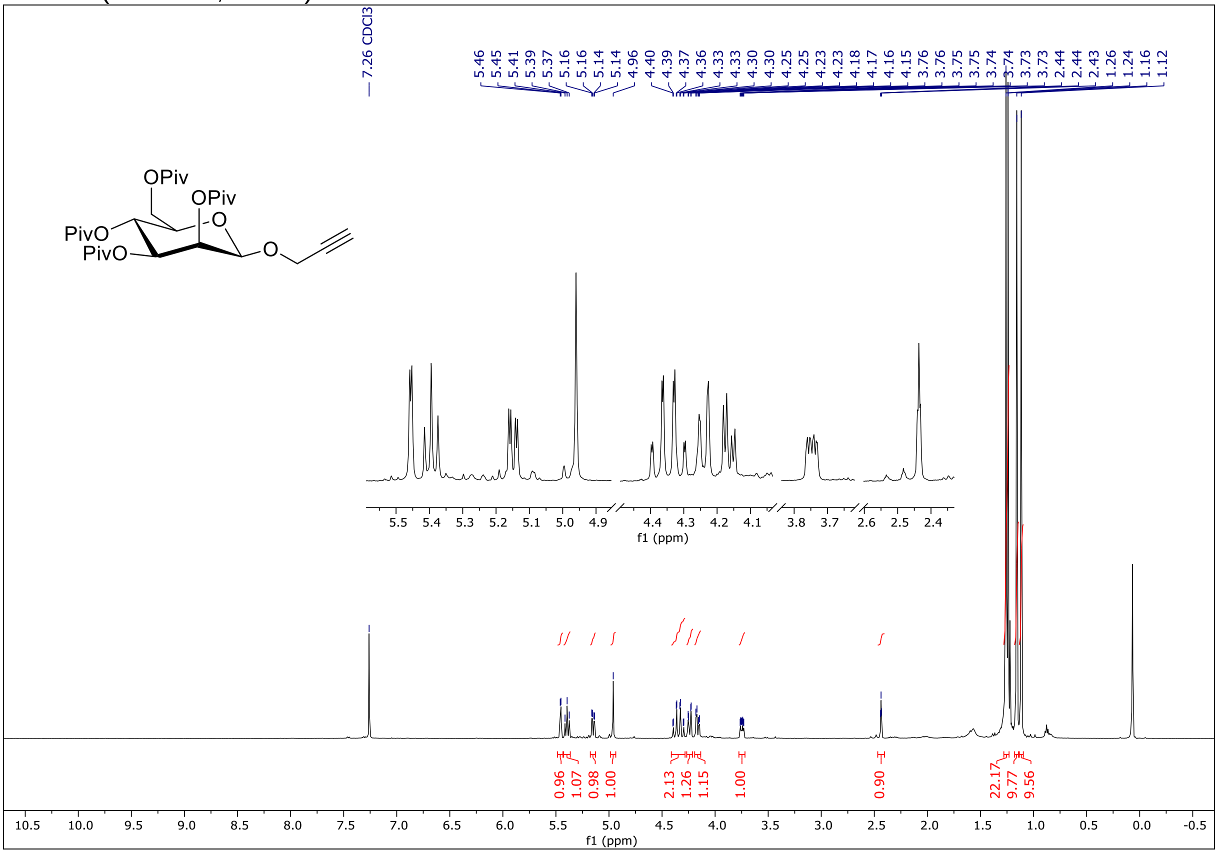

^{13}C NMR (126 MHz, $CDCl_3$)

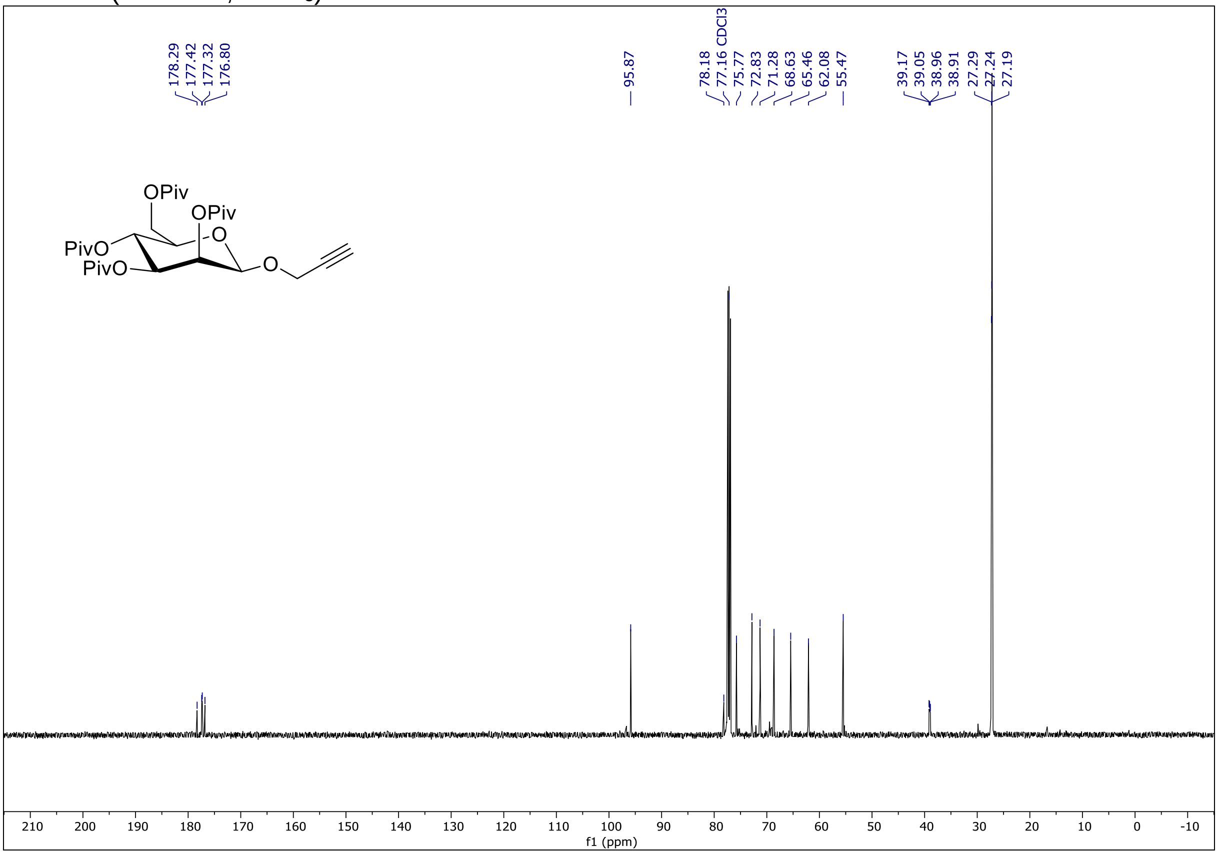

α-**3g**

^{1}H NMR (500 MHz, $CDCl_3$)

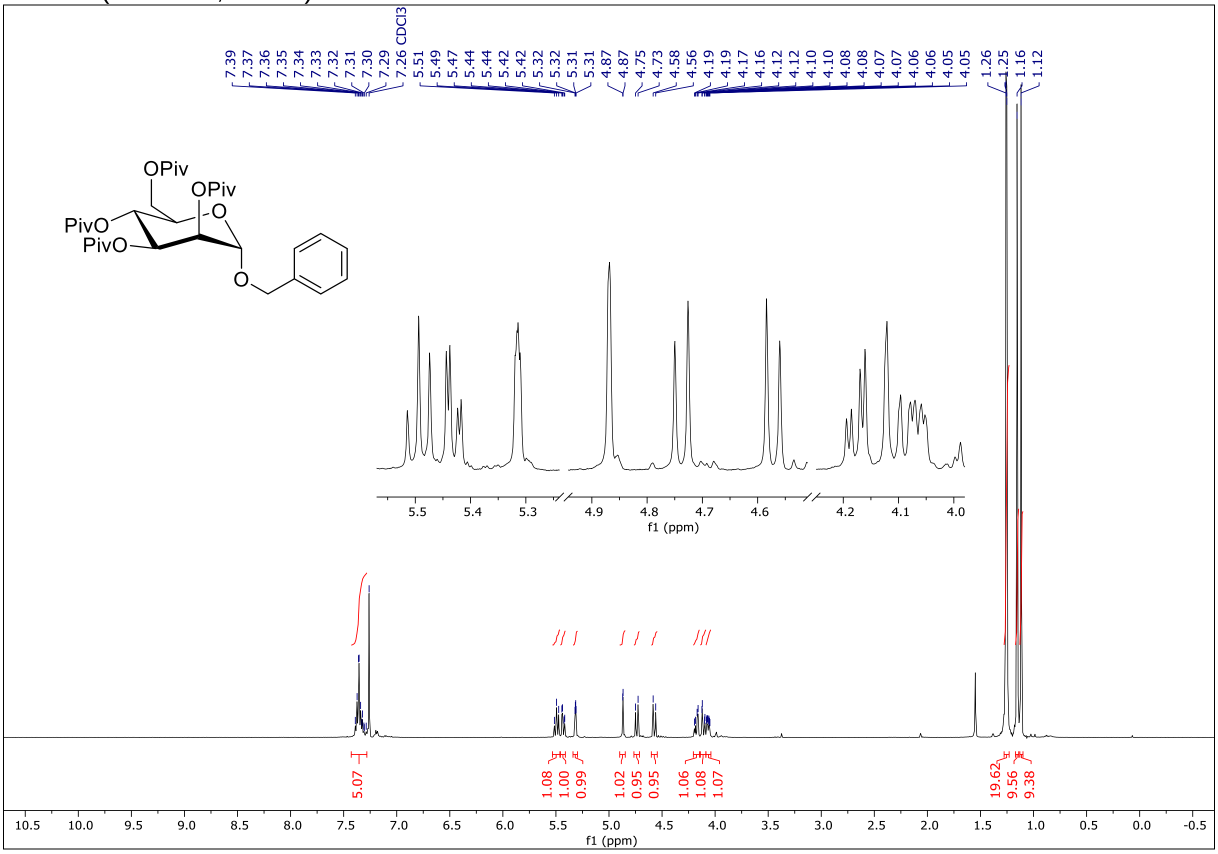

^{13}C NMR (126 MHz, $CDCl_3$)

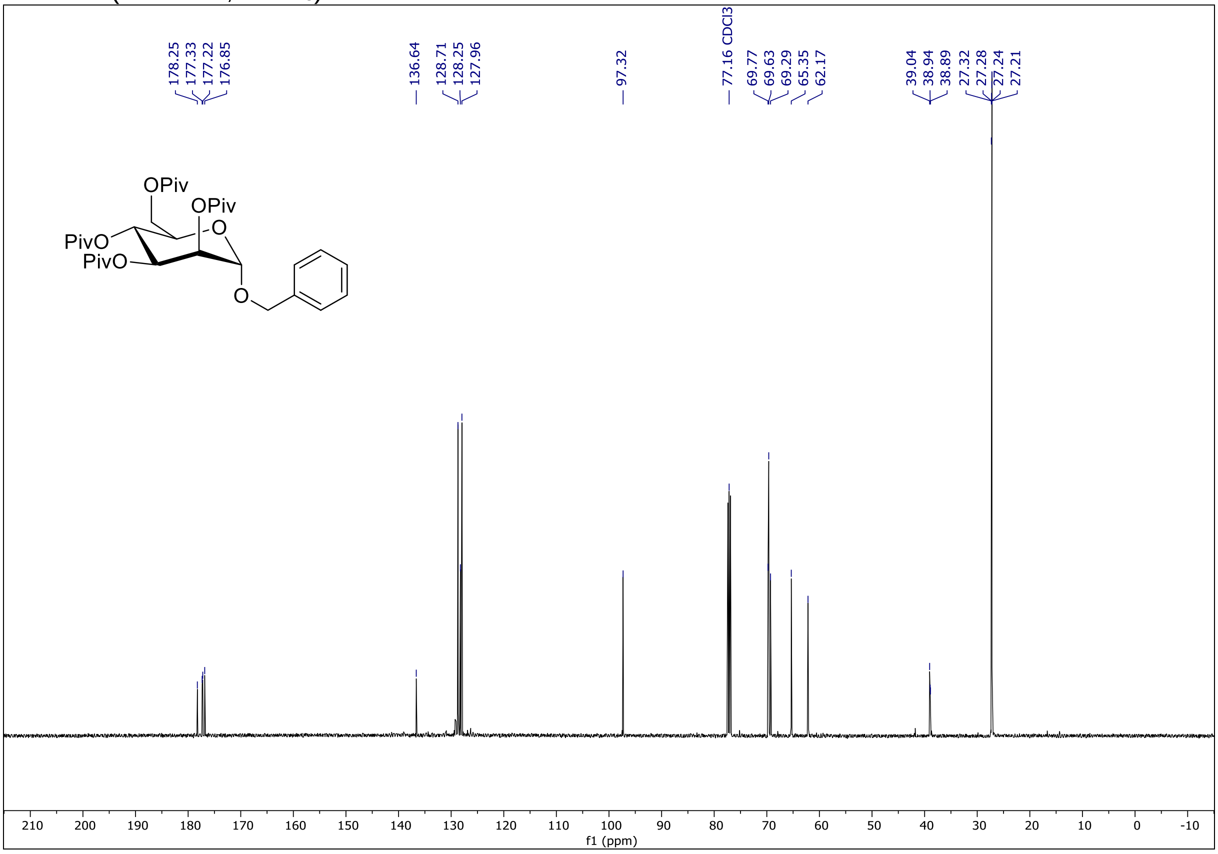

β-3g

^{1}H NMR (500 MHz, $CDCl_3$)

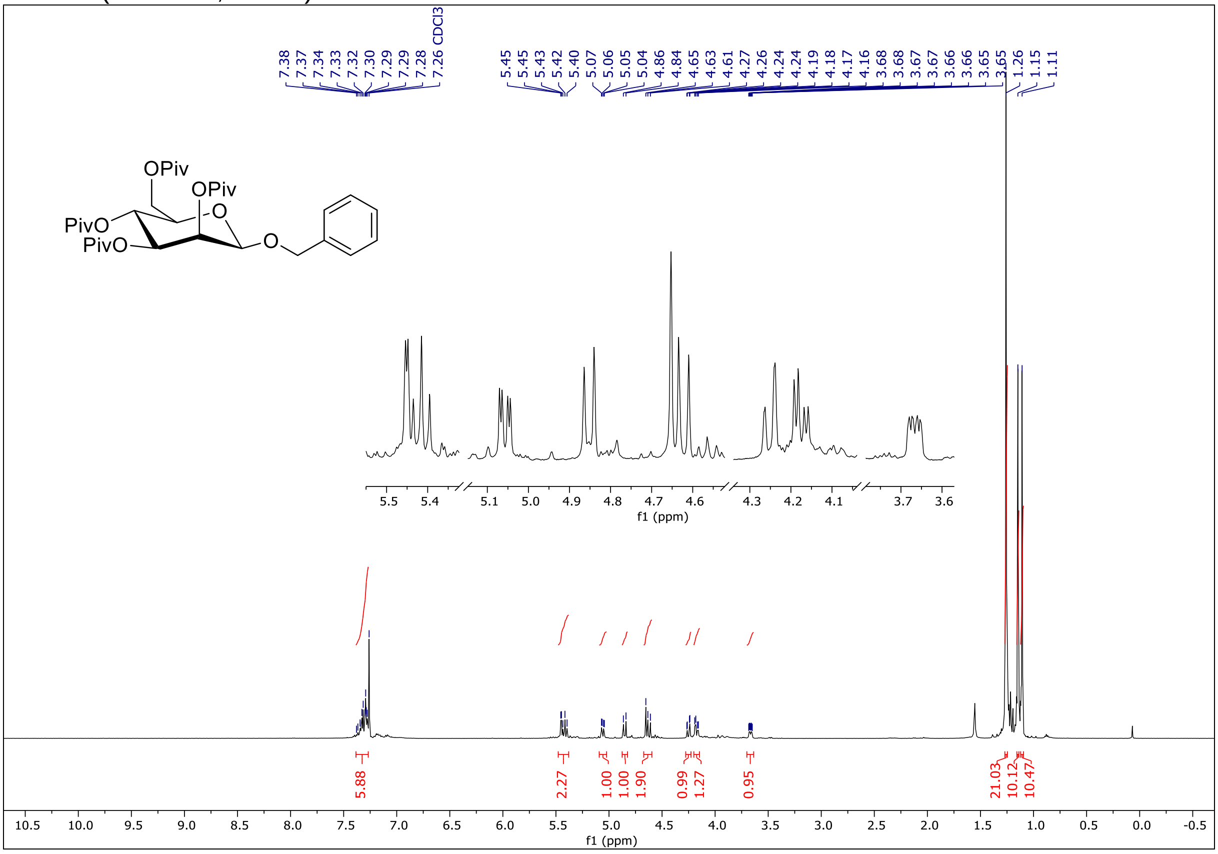

^{13}C NMR (126 MHz, $CDCl_3$)

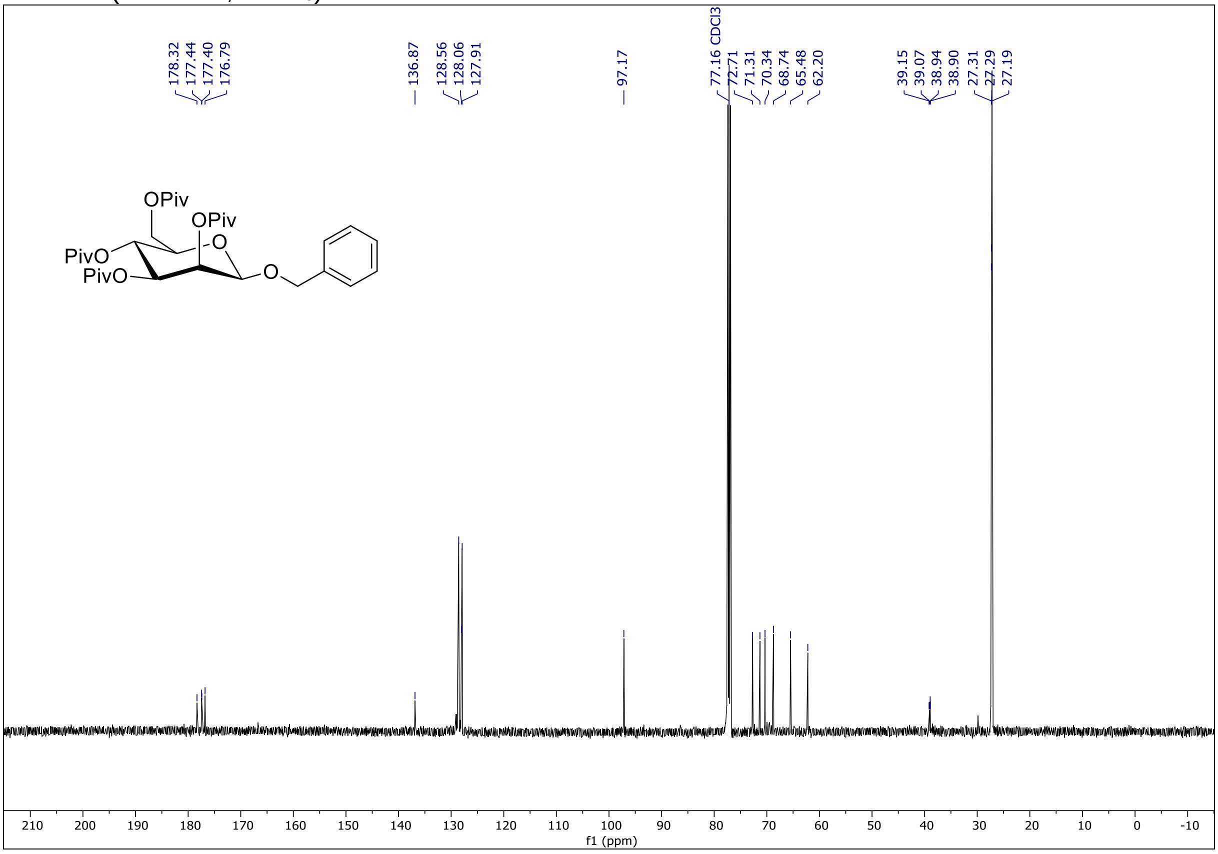

α-3h

^{1}H NMR (300 MHz, CDCl$_3$)

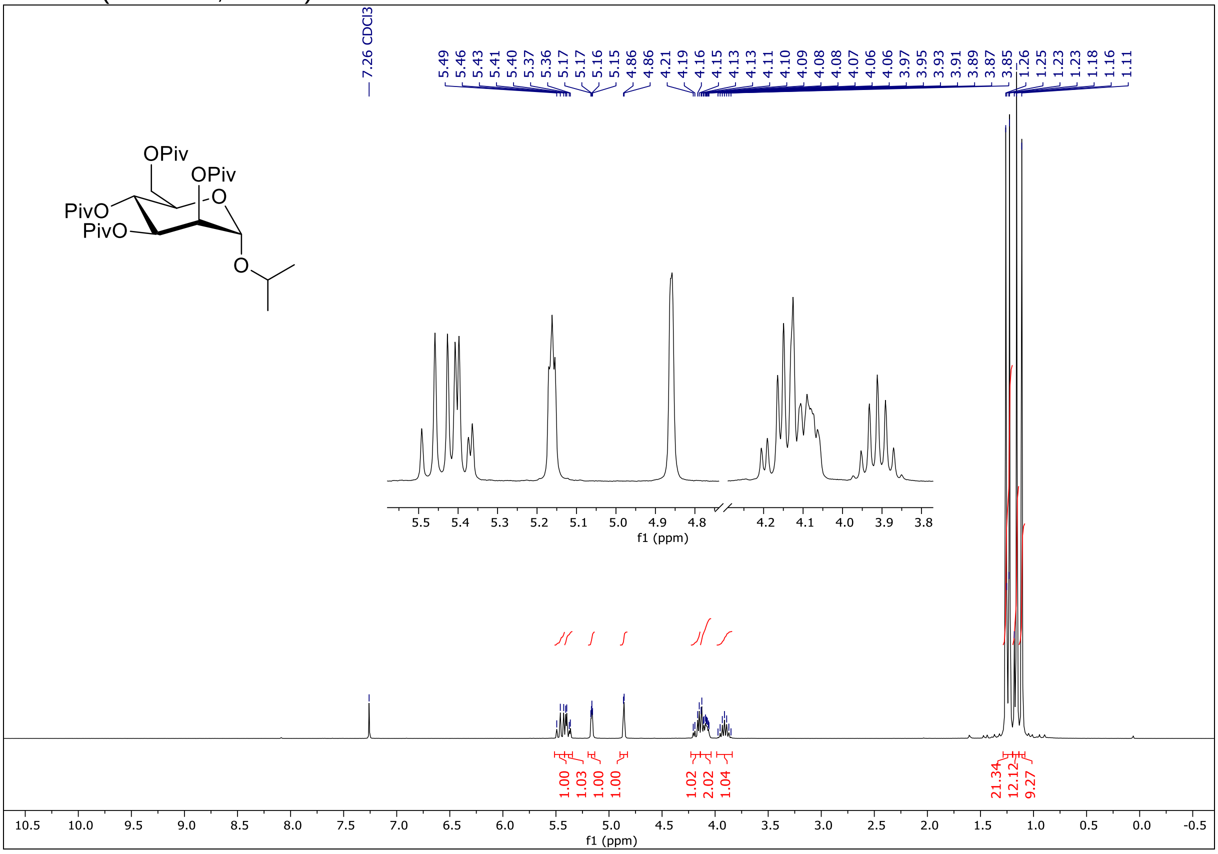

^{13}C NMR (75.5 MHz, CDCl$_3$)

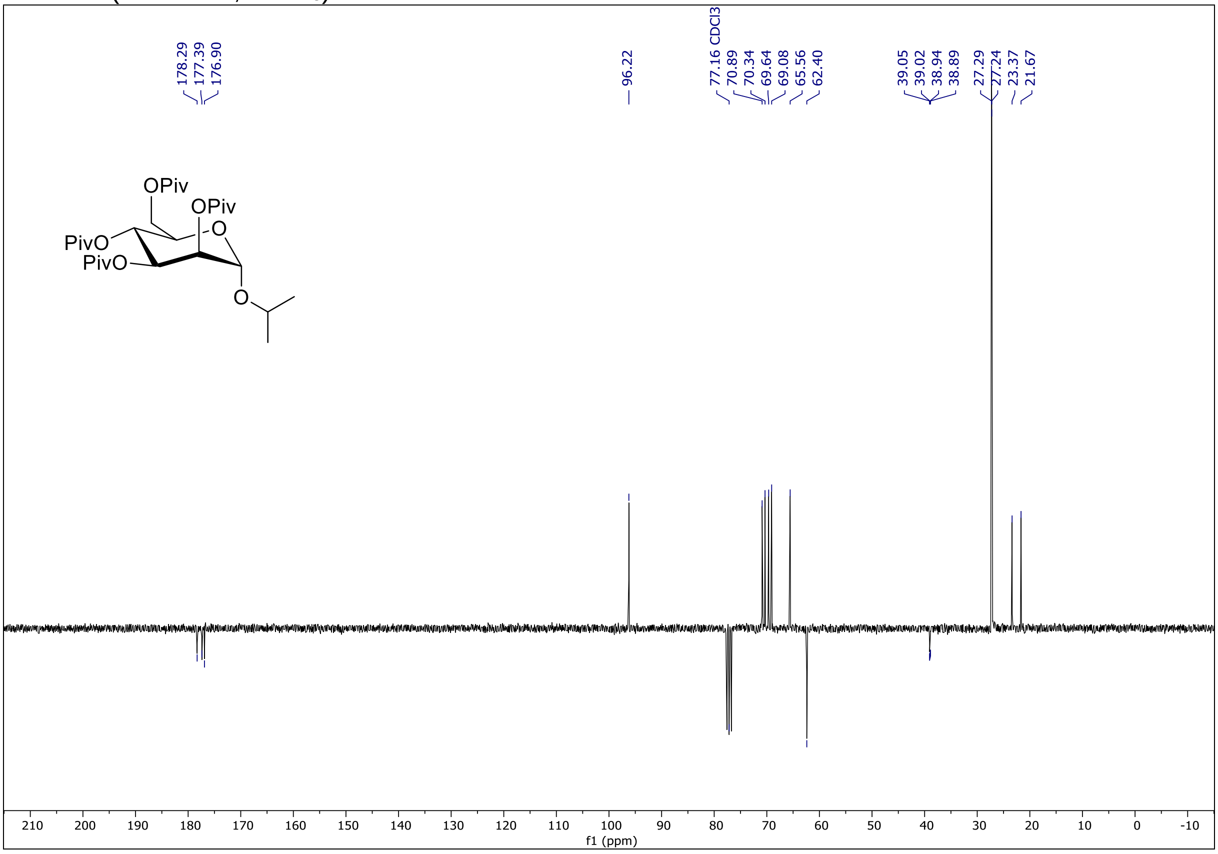

α-3i

^{1}H NMR (500 MHz, $CDCl_3$)

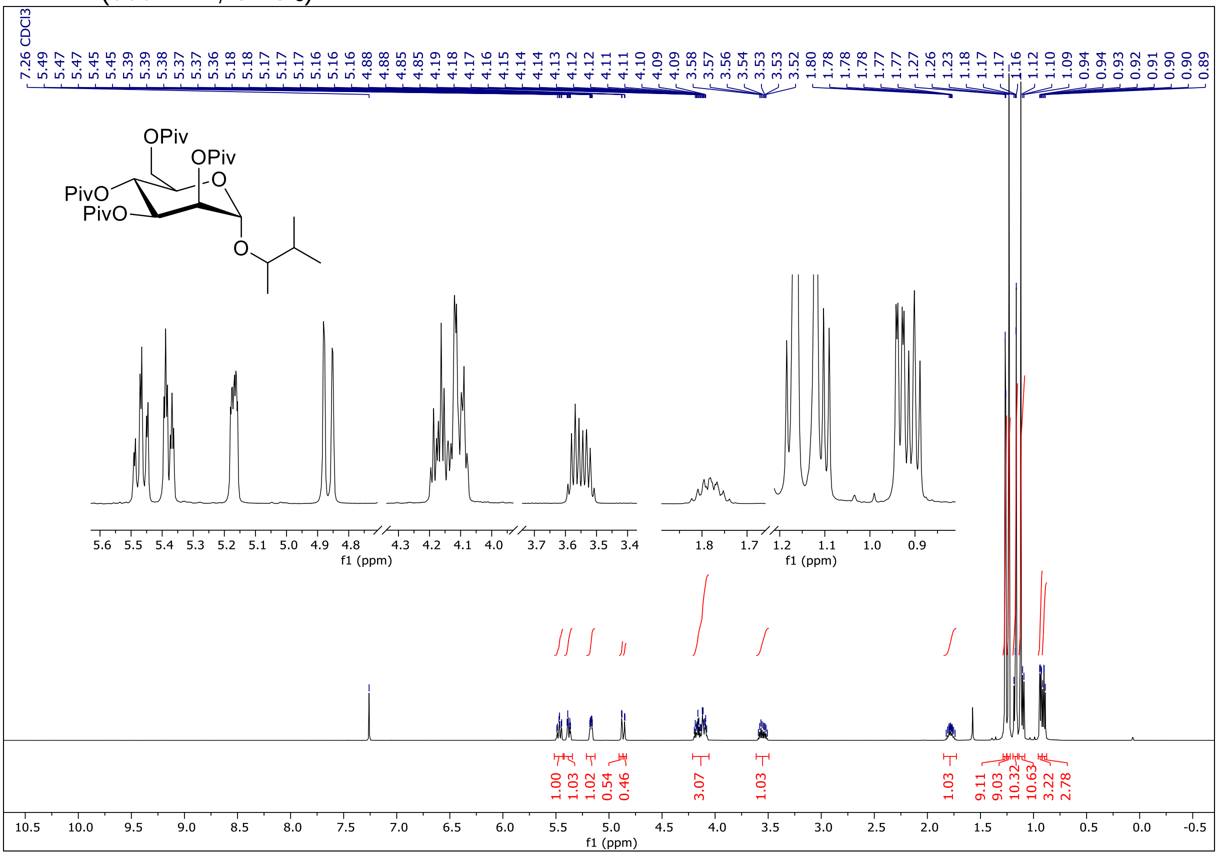

^{13}C NMR (126 MHz, $CDCl_3$)

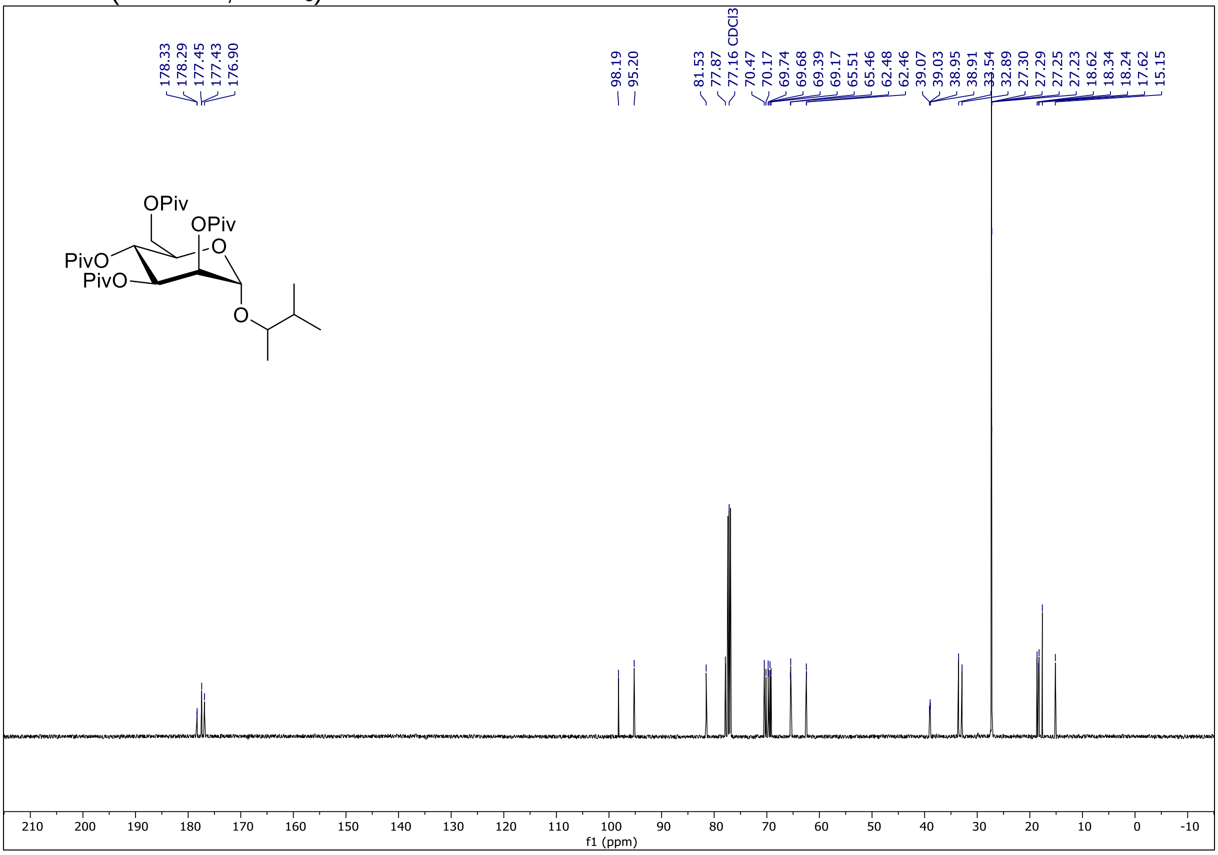

α-**3j**

^{1}H NMR (500 MHz, $CDCl_3$)

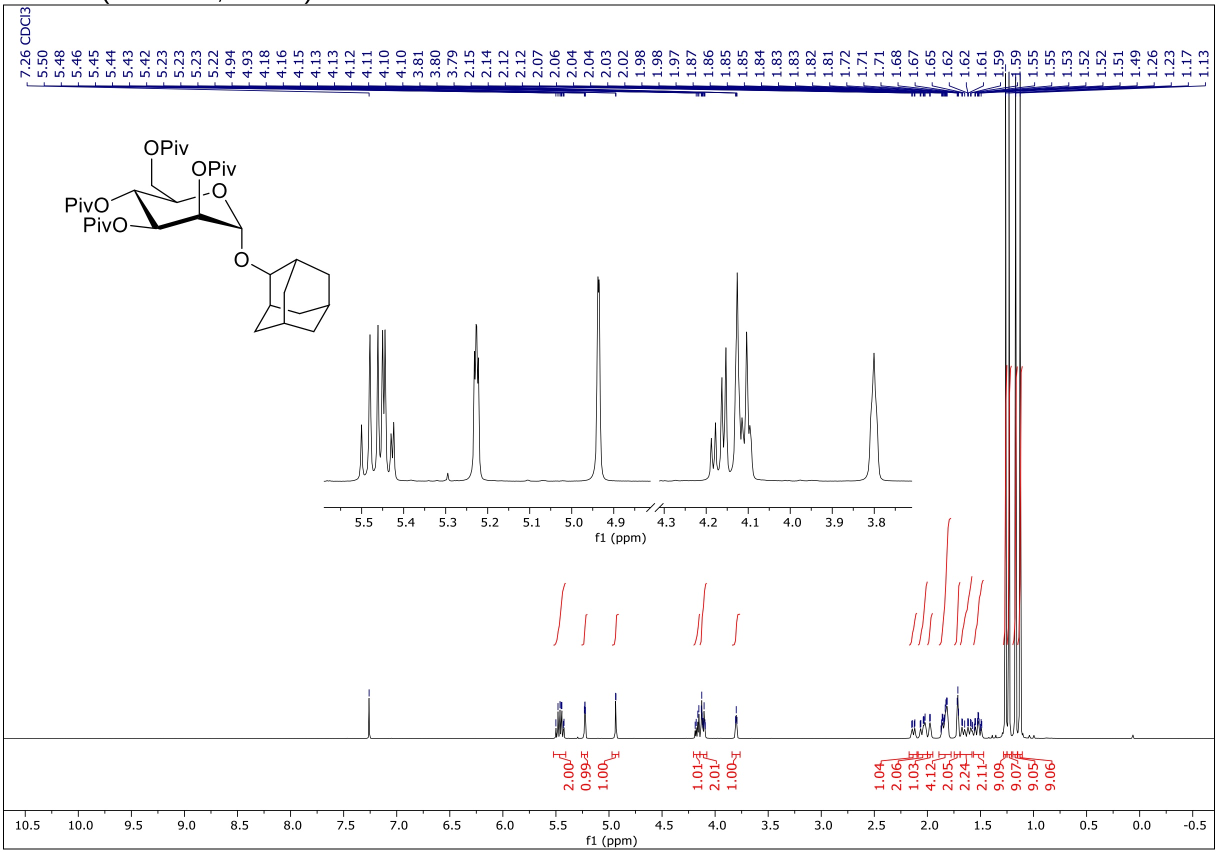

^{13}C NMR (126 MHz, $CDCl_3$)

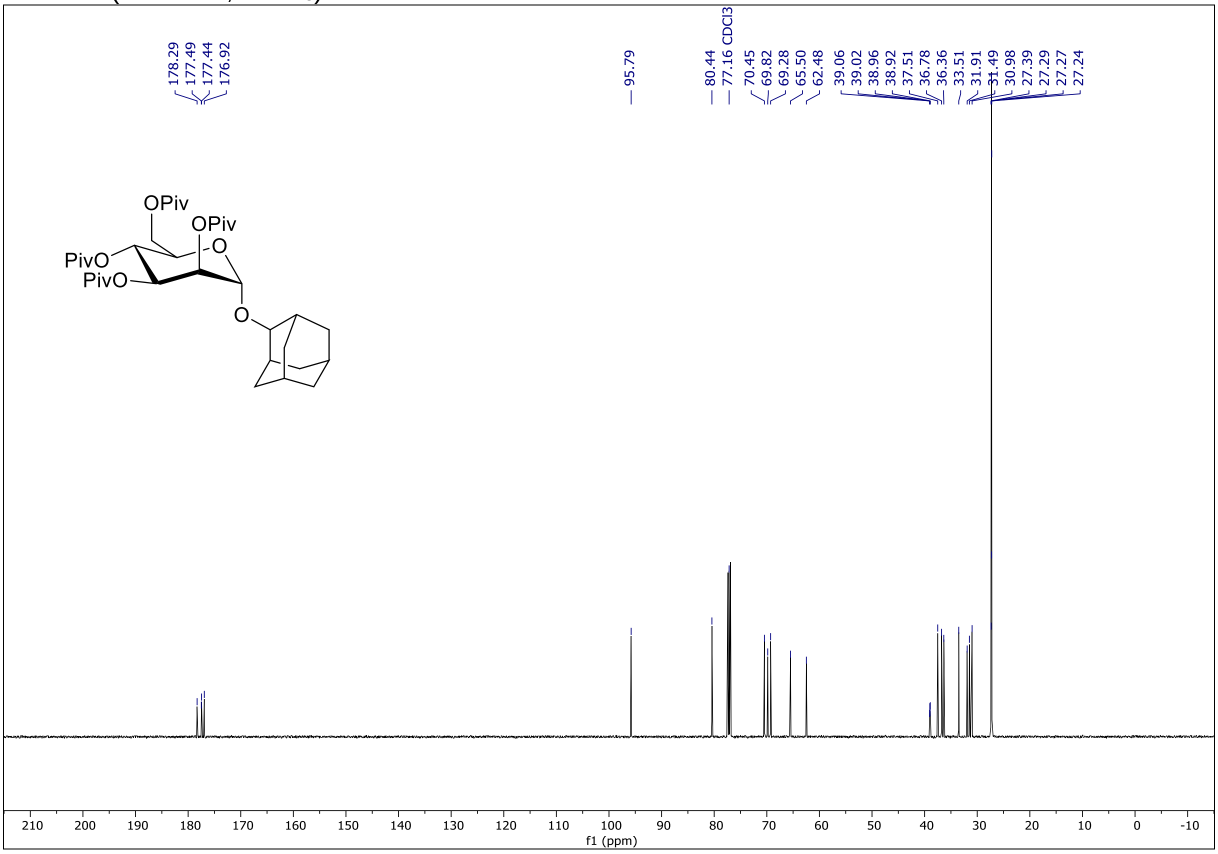

β-**3j**

^{1}H NMR (500 MHz, $CDCl_3$)

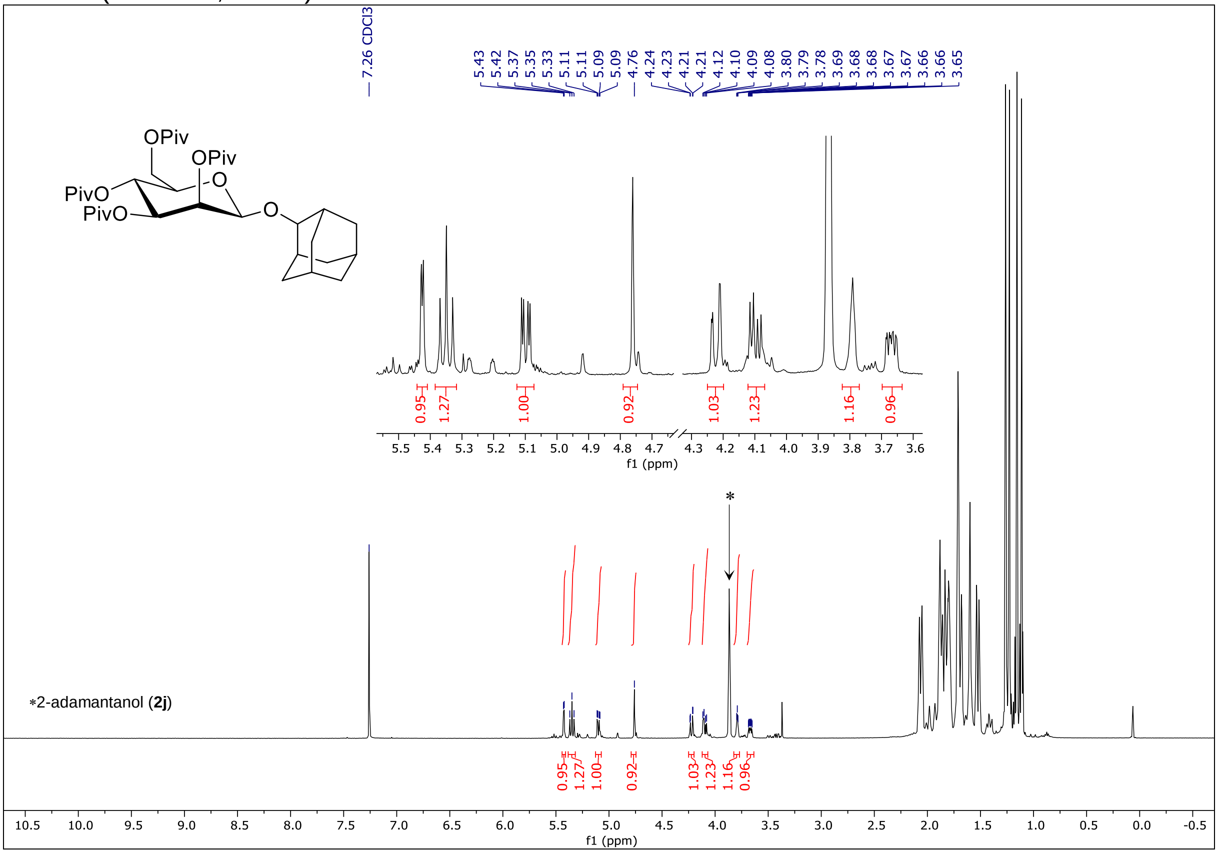

α-3k

^{1}H NMR (500 MHz, $CDCl_3$)

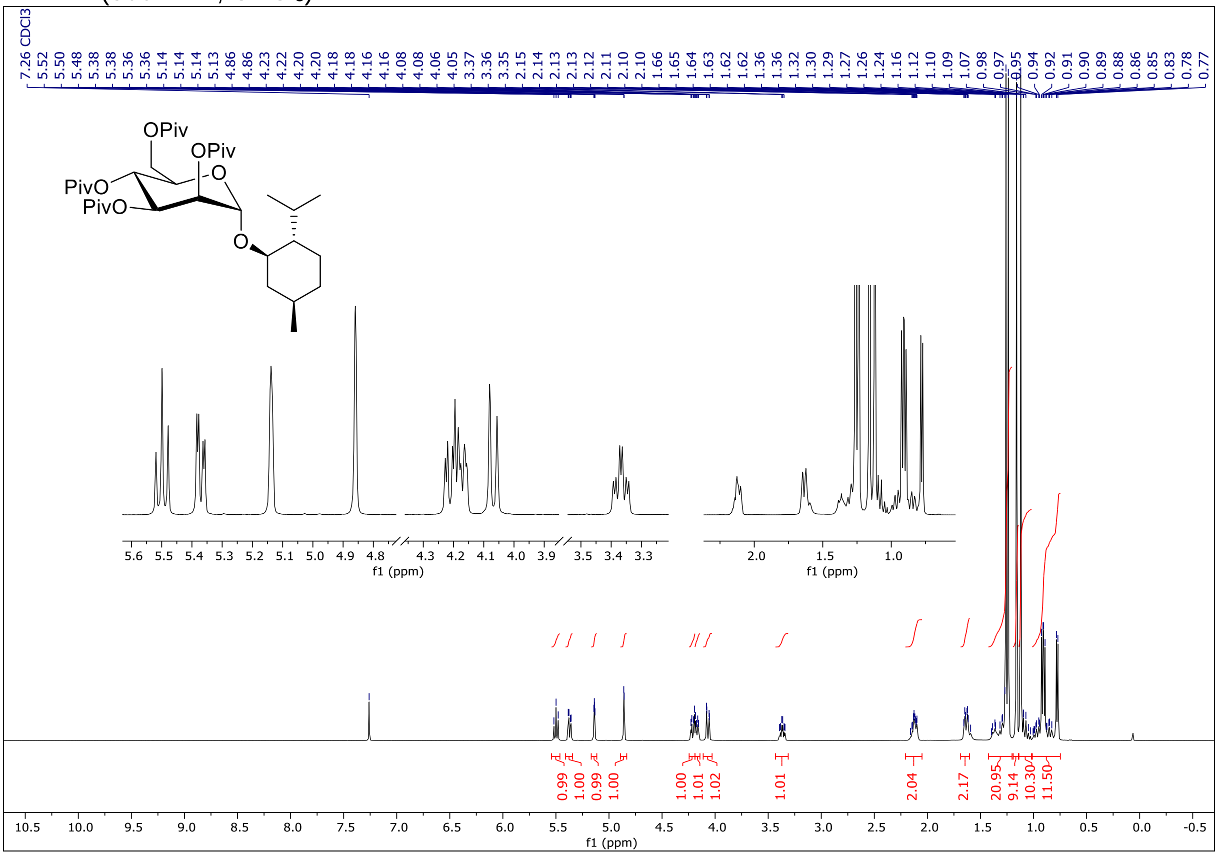

^{13}C NMR (126 MHz, $CDCl_3$)

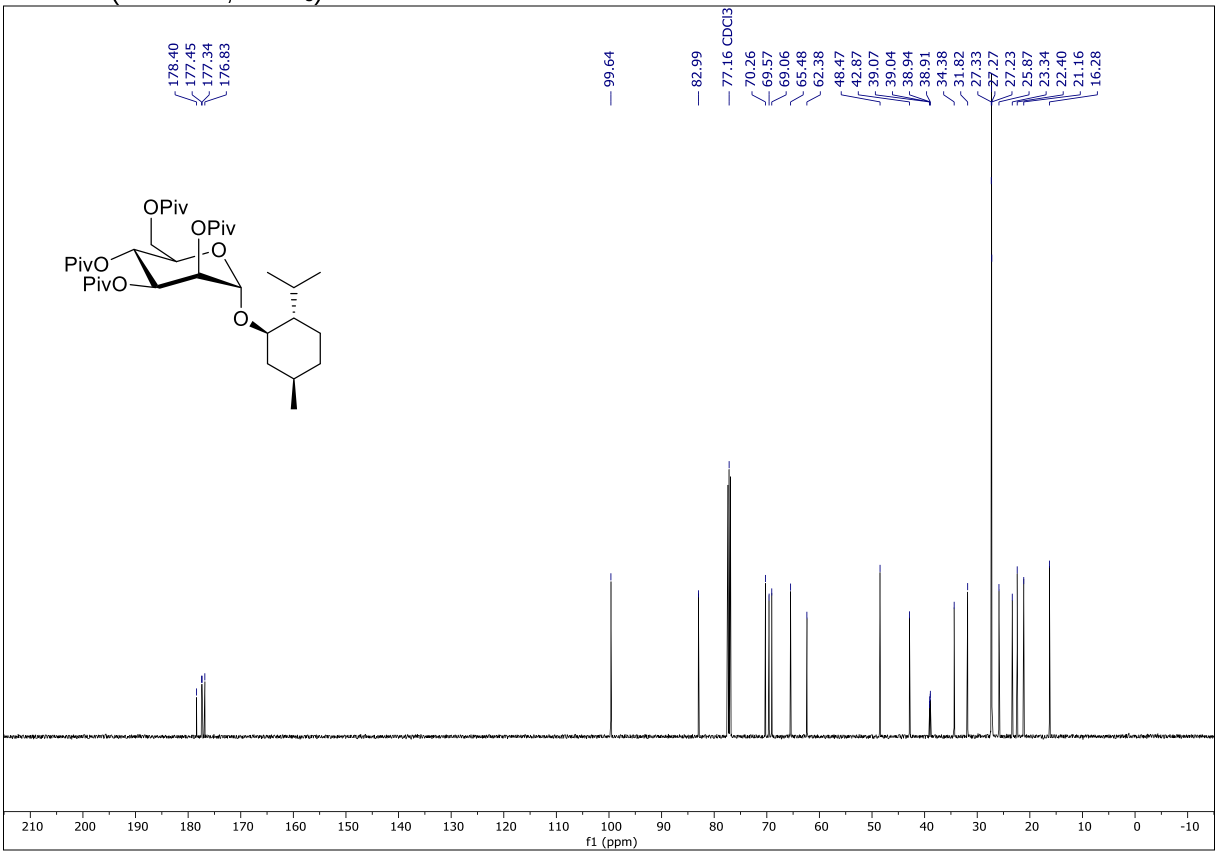

β-3k

^{1}H NMR (500 MHz, $CDCl_3$)

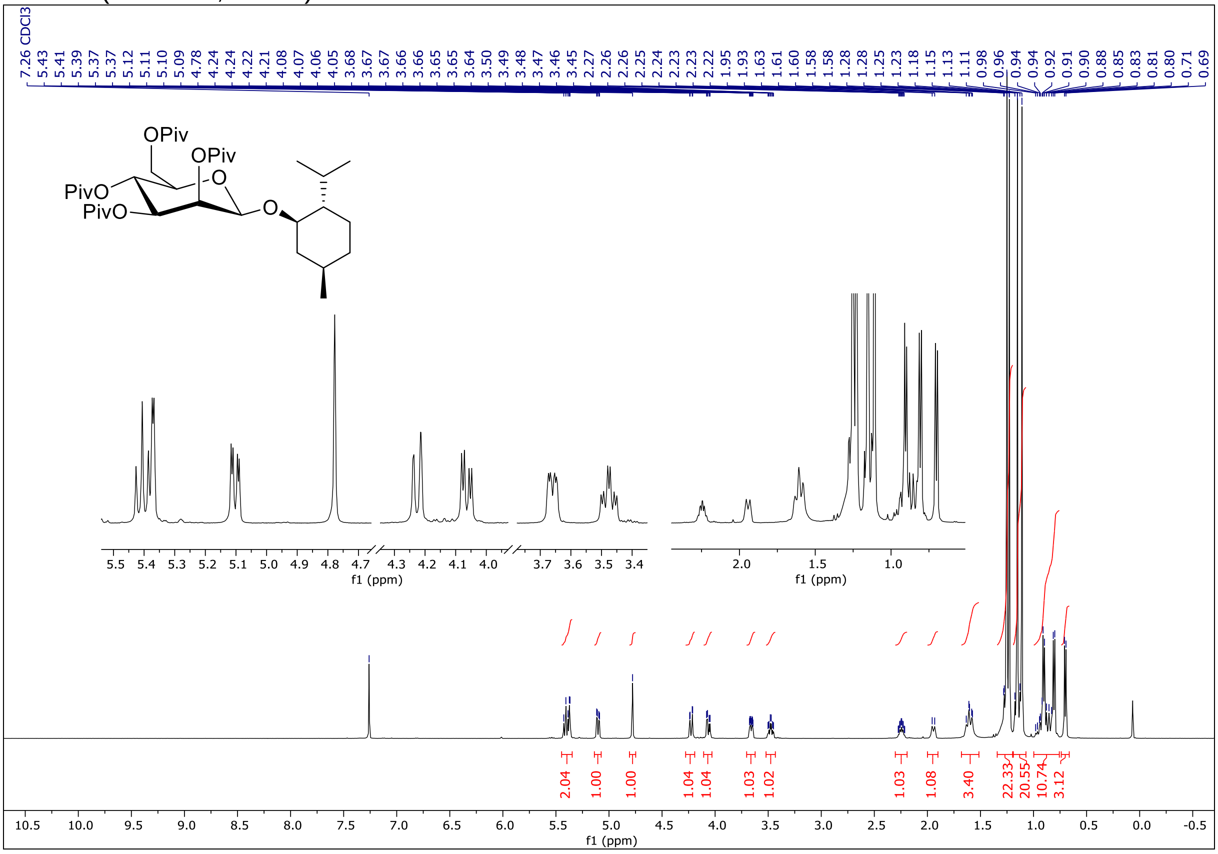

^{13}C NMR (126 MHz, $CDCl_3$)

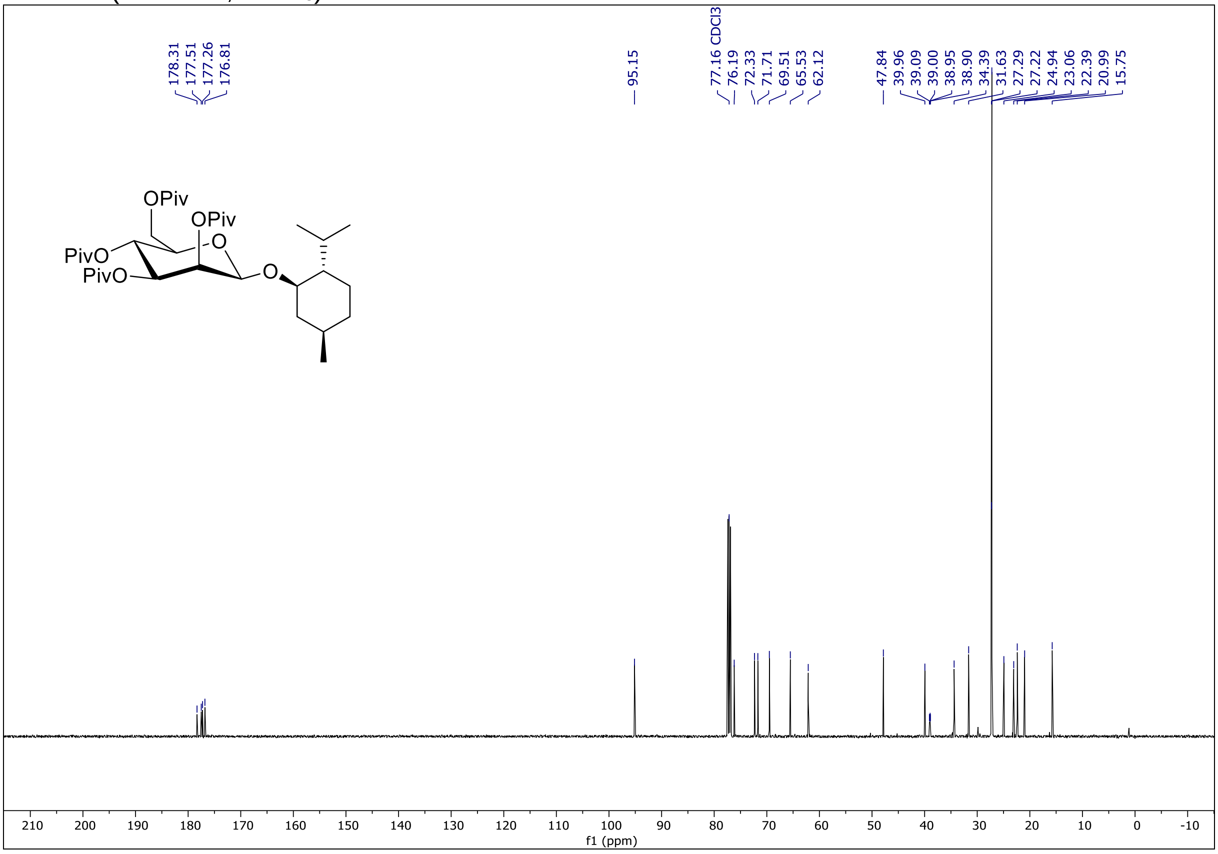

α-**3l**

^{1}H NMR (500 MHz, CDCl$_3$)

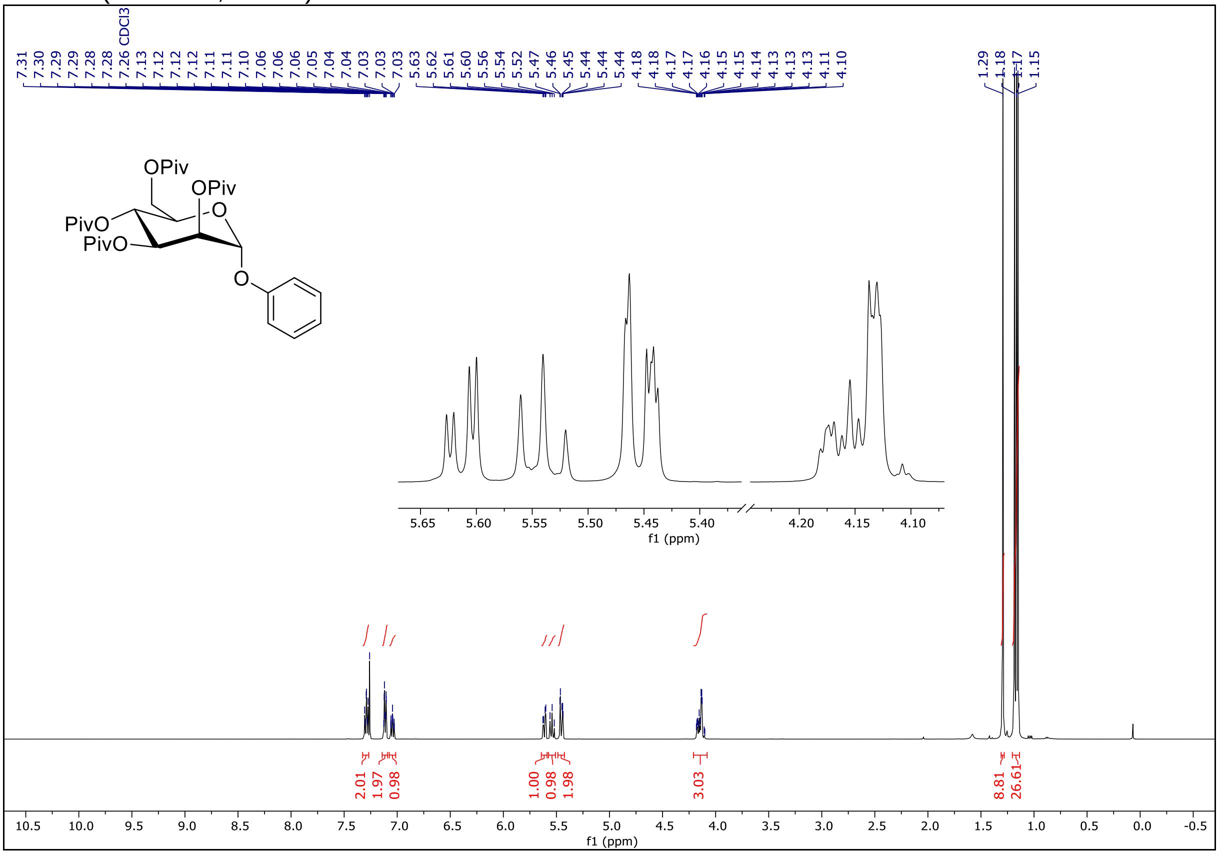

^{13}C NMR (126 MHz, CDCl$_3$)

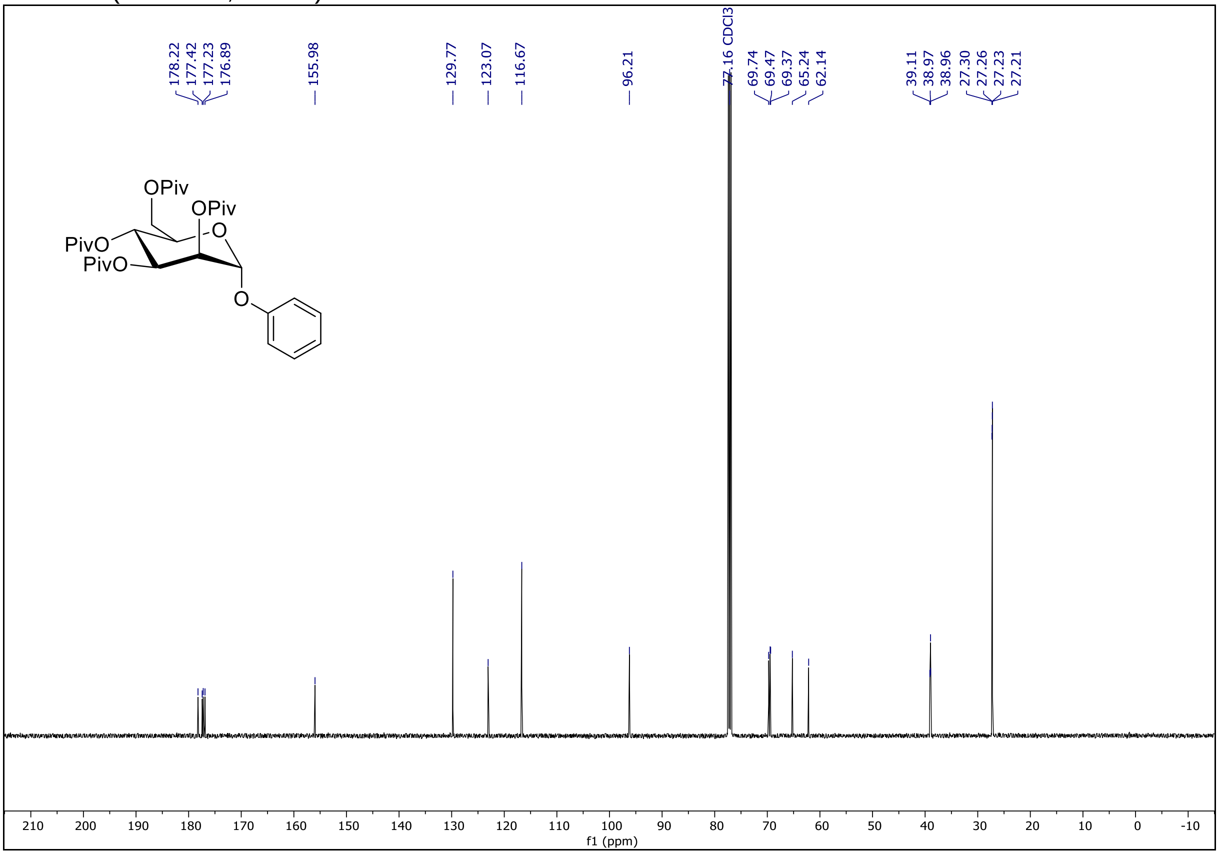

α-3m

^1H NMR (300 MHz, $CDCl_3$)

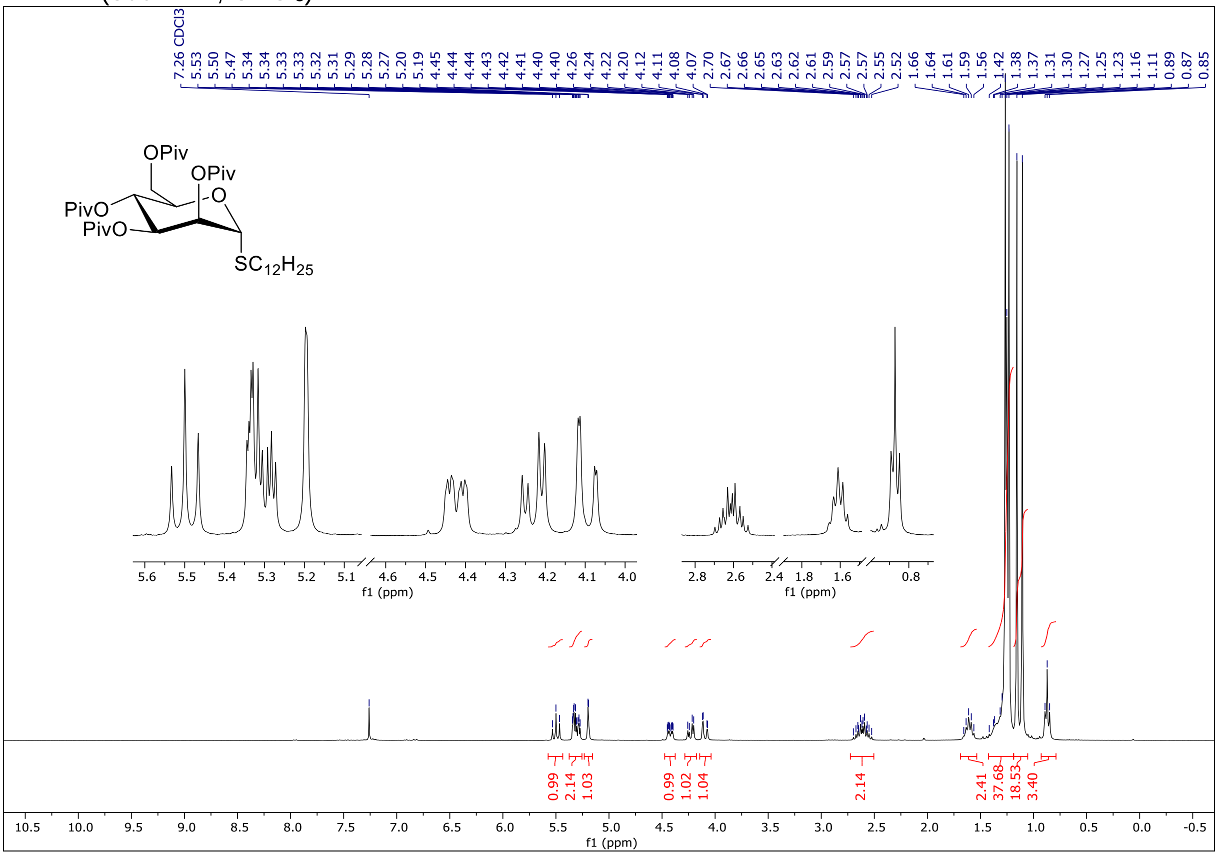

^{13}C NMR (75.5 MHz, $CDCl_3$)

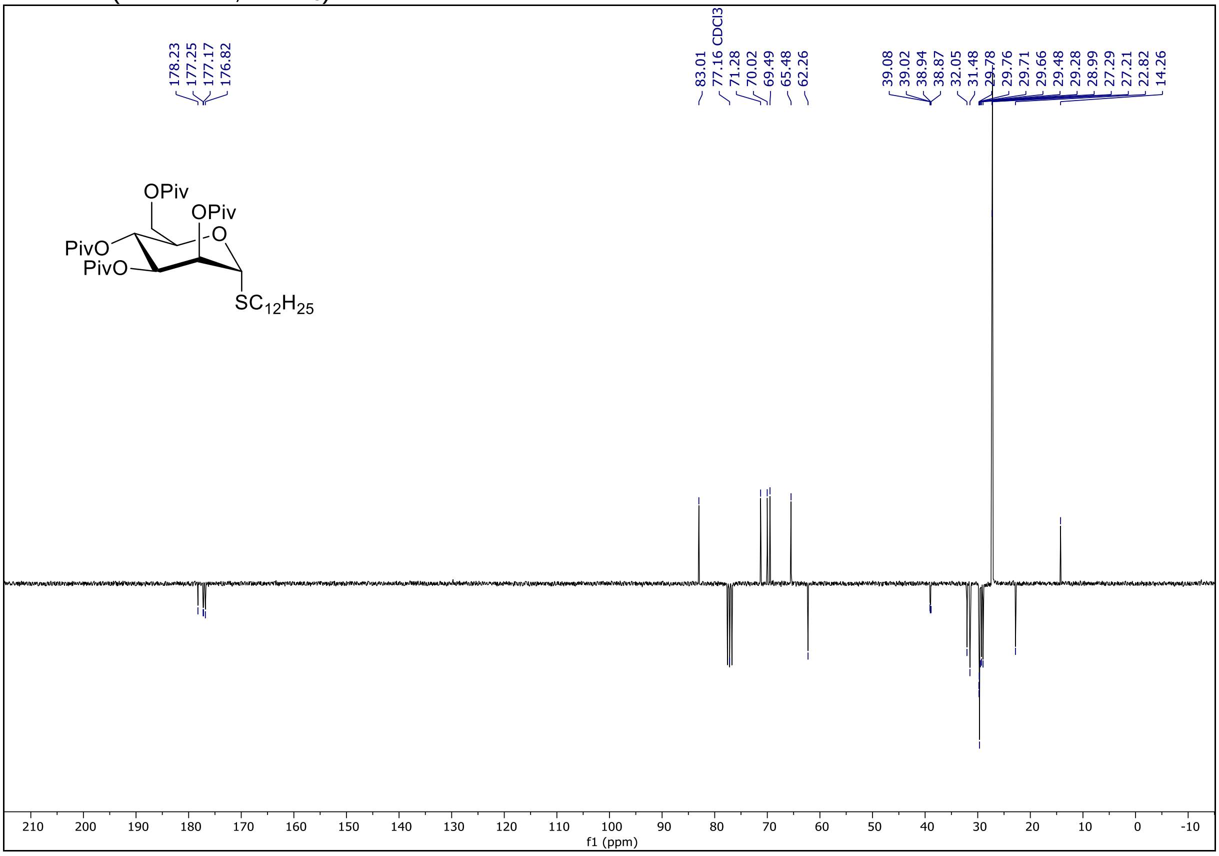

β-3m

^{1}H NMR (300 MHz, $CDCl_3$)

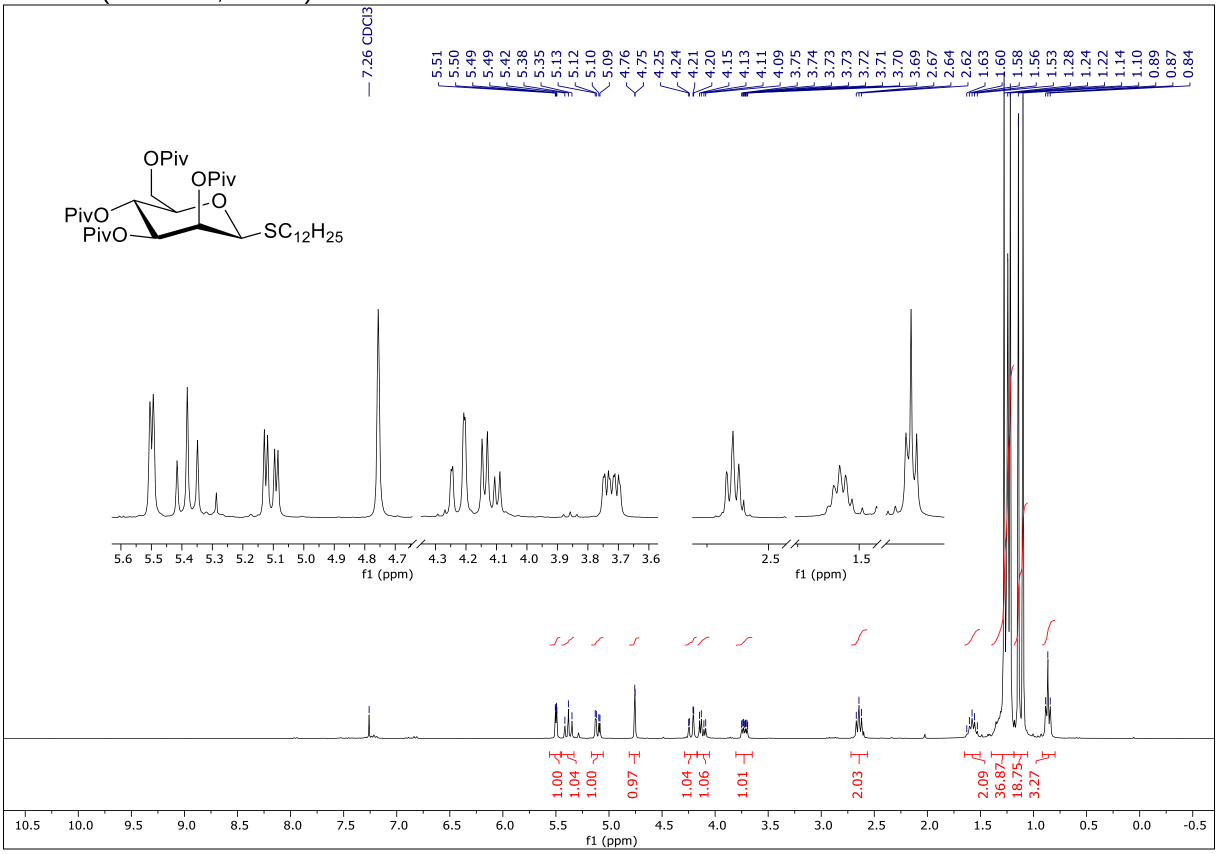

^{13}C NMR (75.5 MHz, $CDCl_3$)

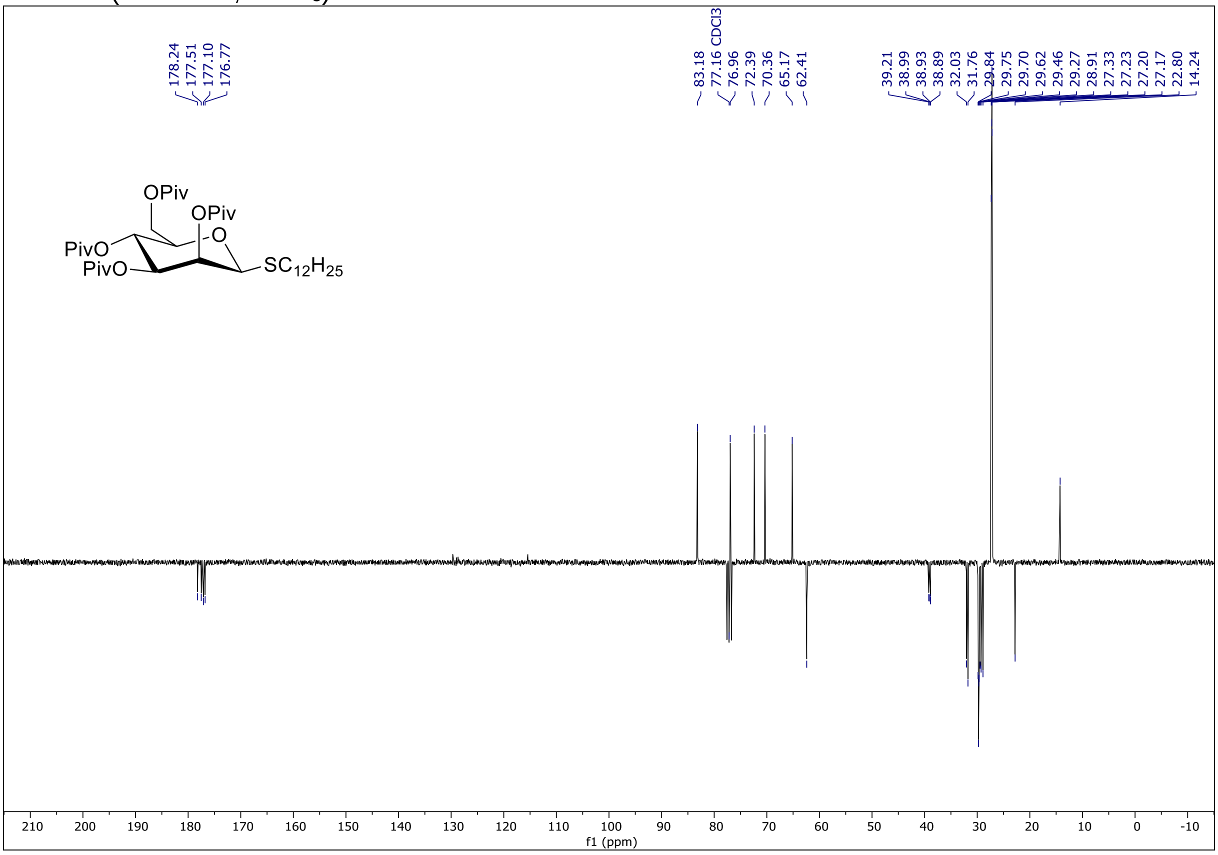

α-**3n**

^{1}H NMR (300 MHz, $CDCl_3$)

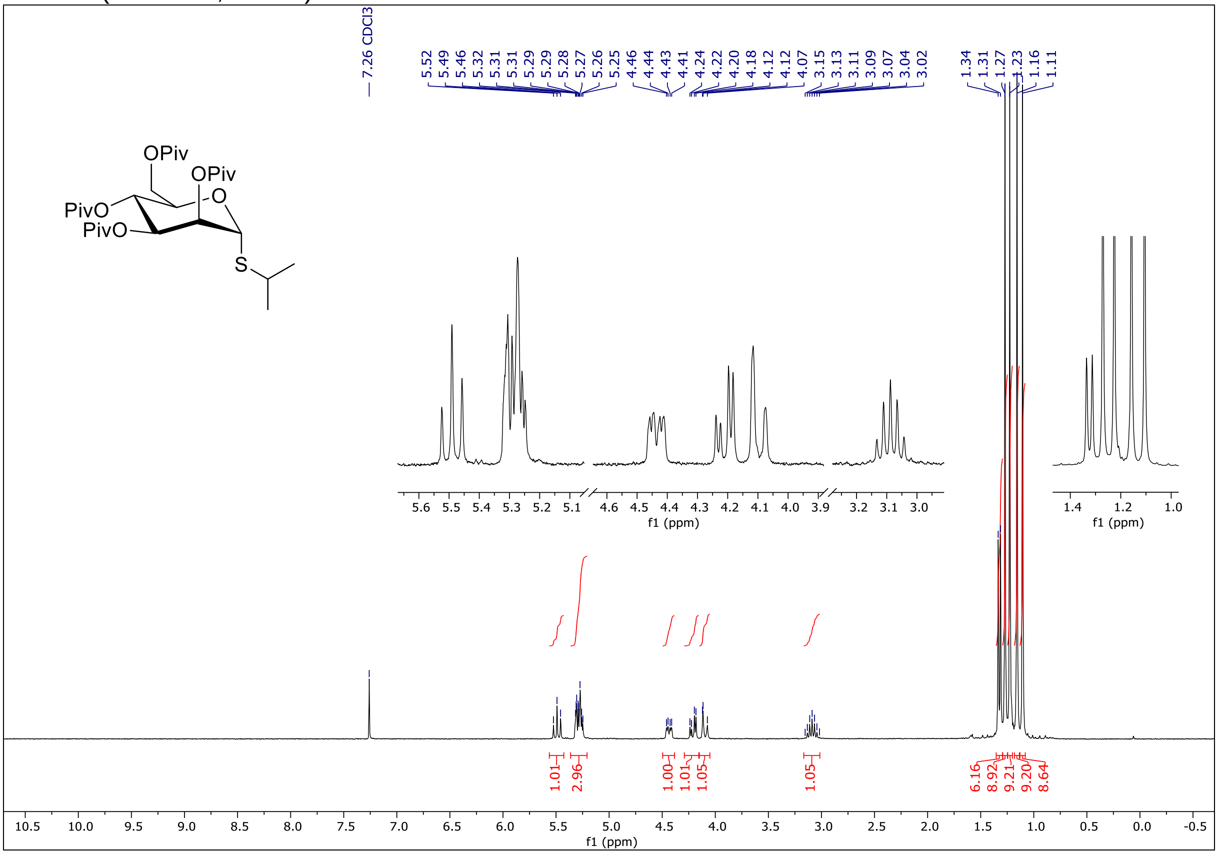

^{13}C NMR (75.5 MHz, $CDCl_3$)

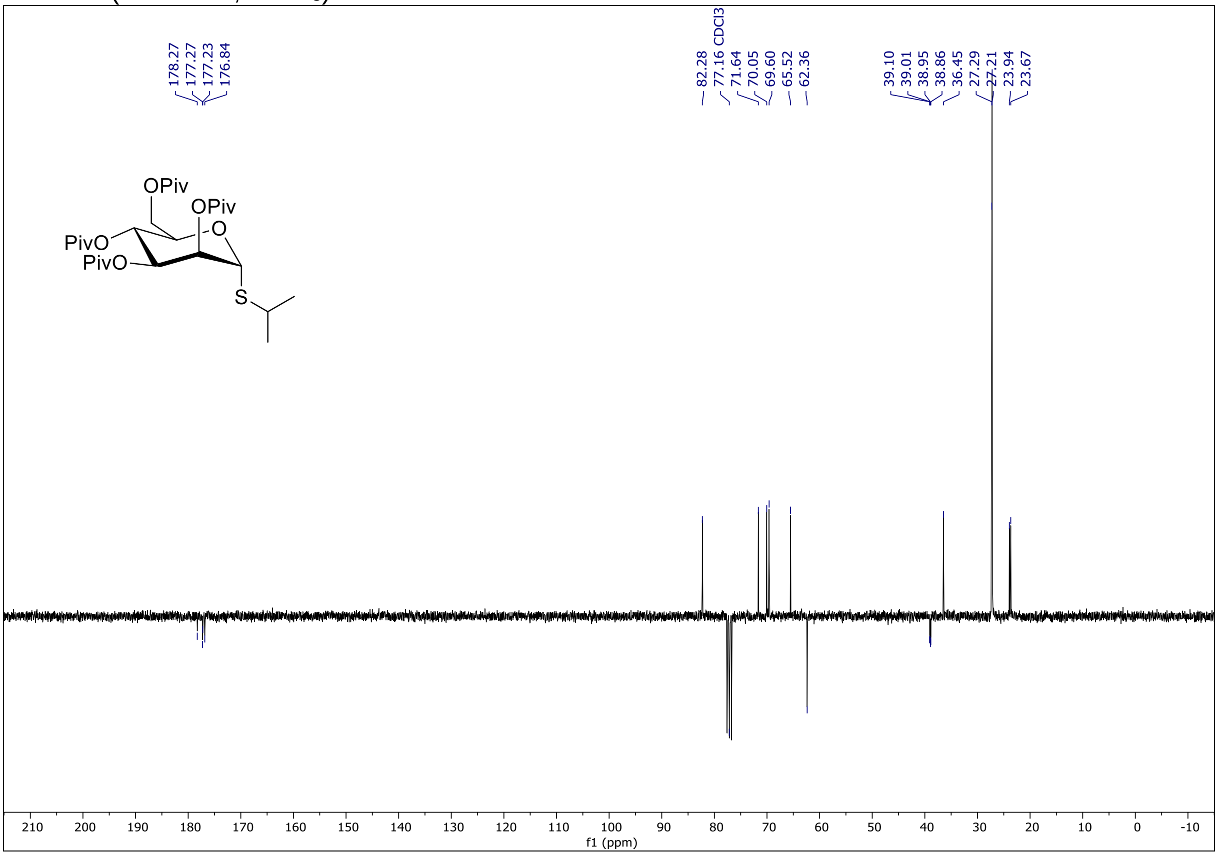

β-3n

^{1}H NMR (300 MHz, $CDCl_3$)

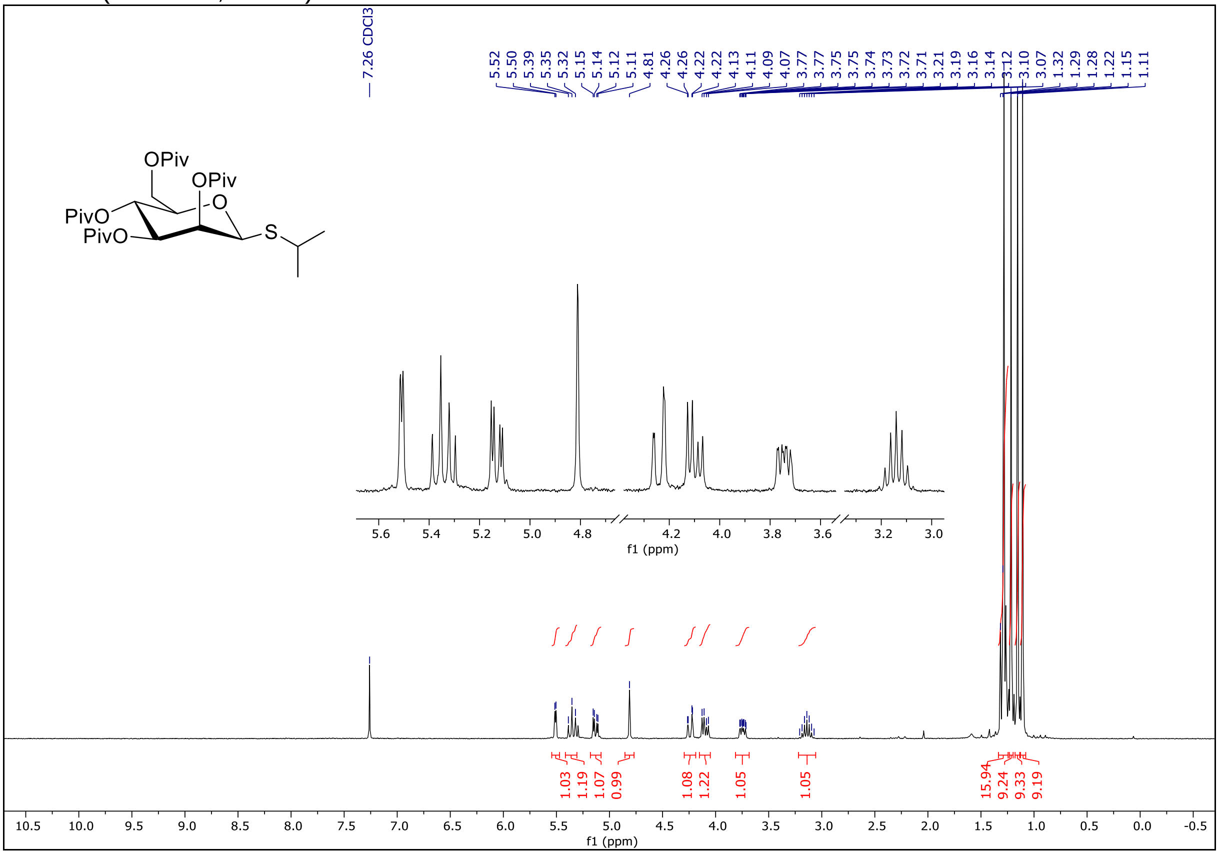

^{13}C NMR (75.5 MHz, $CDCl_3$)

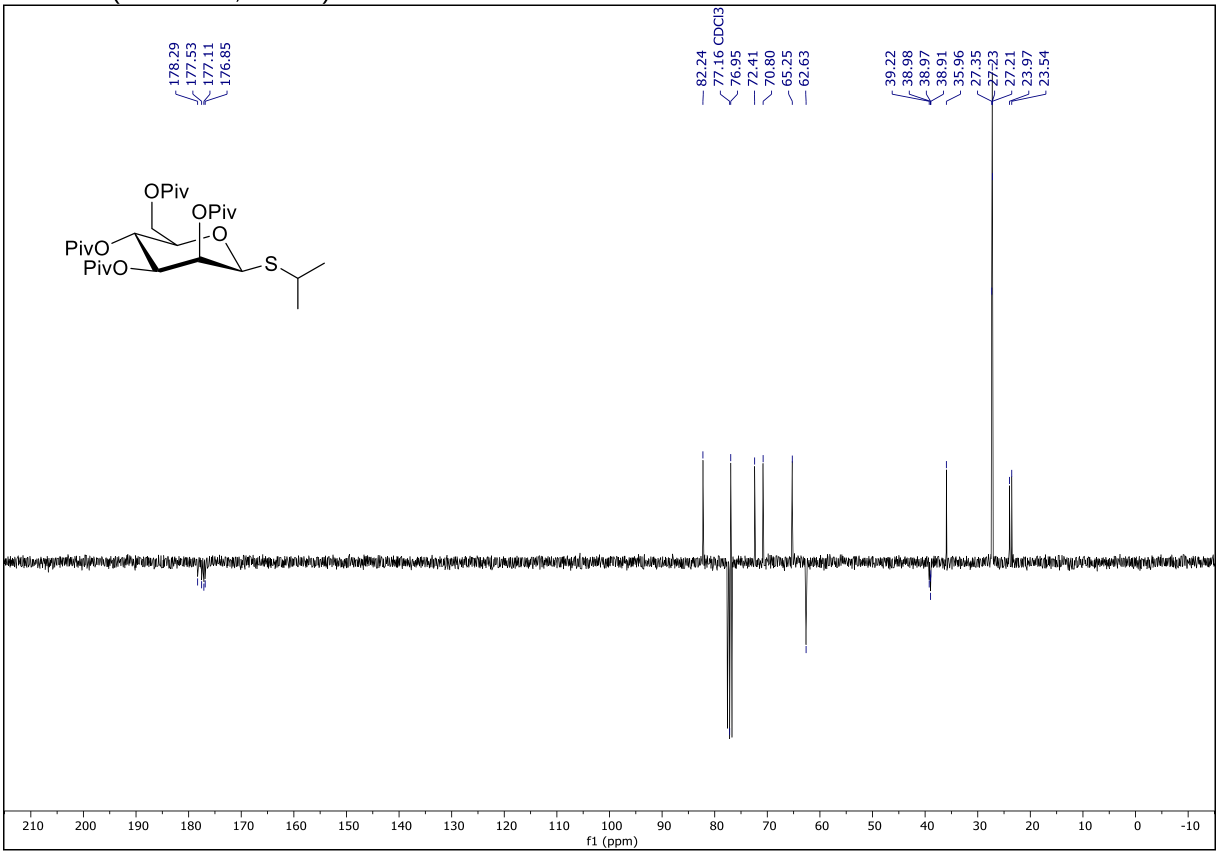

α-3o

^{1}H NMR (300 MHz, $CDCl_3$)

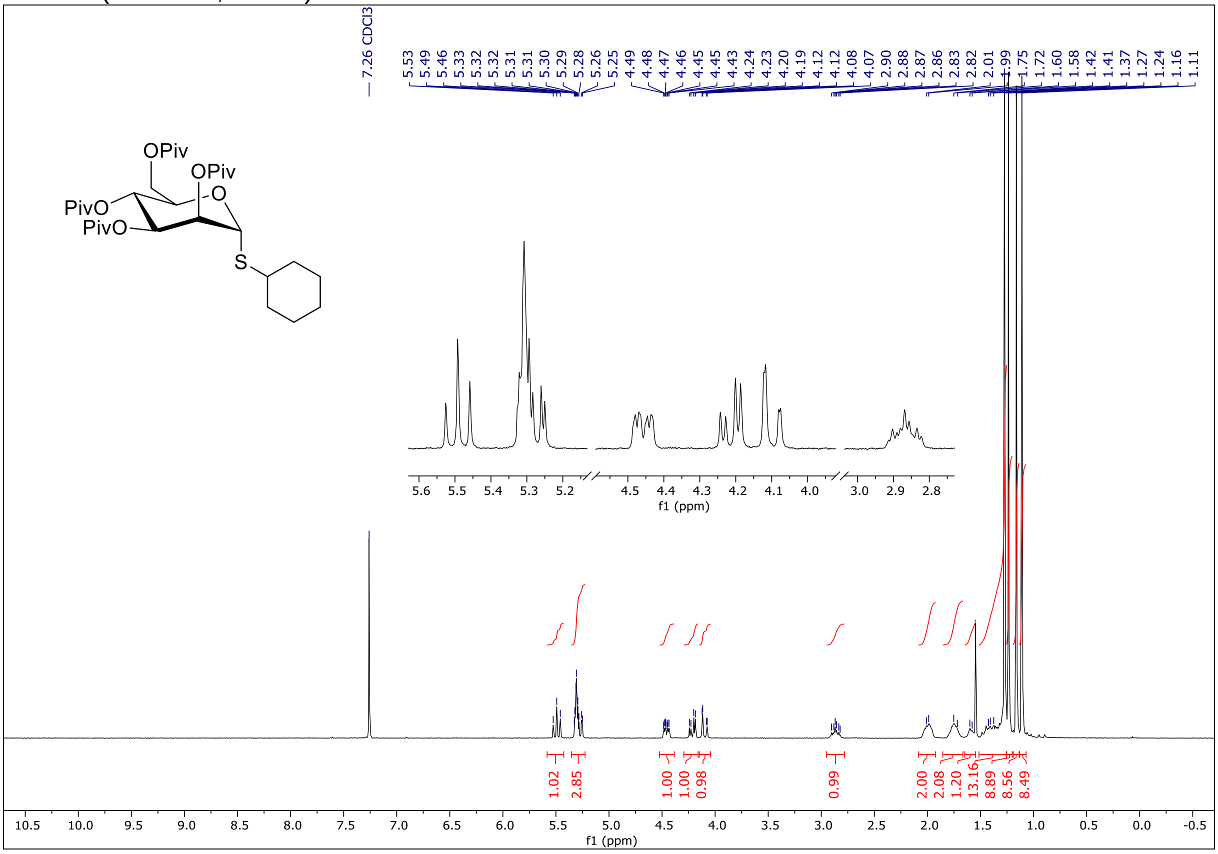

^{13}C NMR (75.5 MHz, $CDCl_3$)

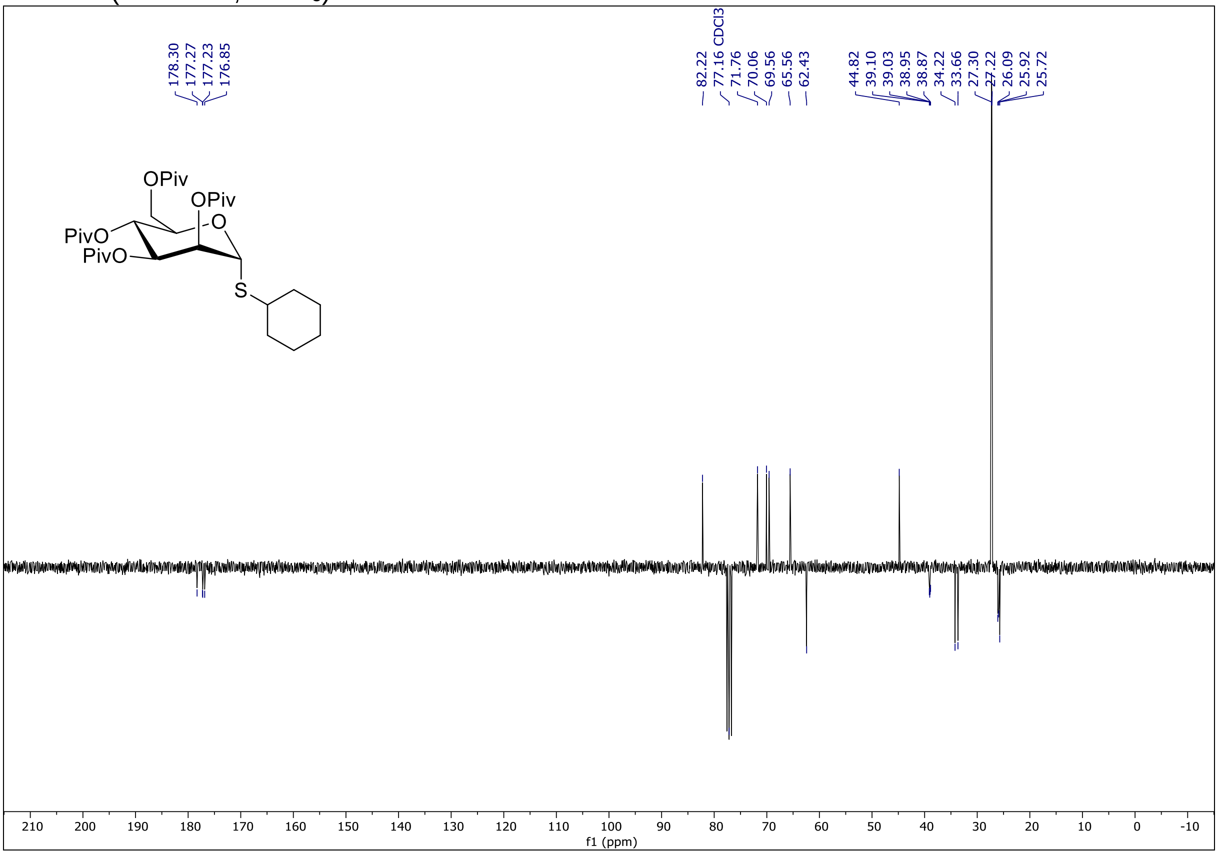

β-3o

¹H NMR (300 MHz, $CDCl_3$)

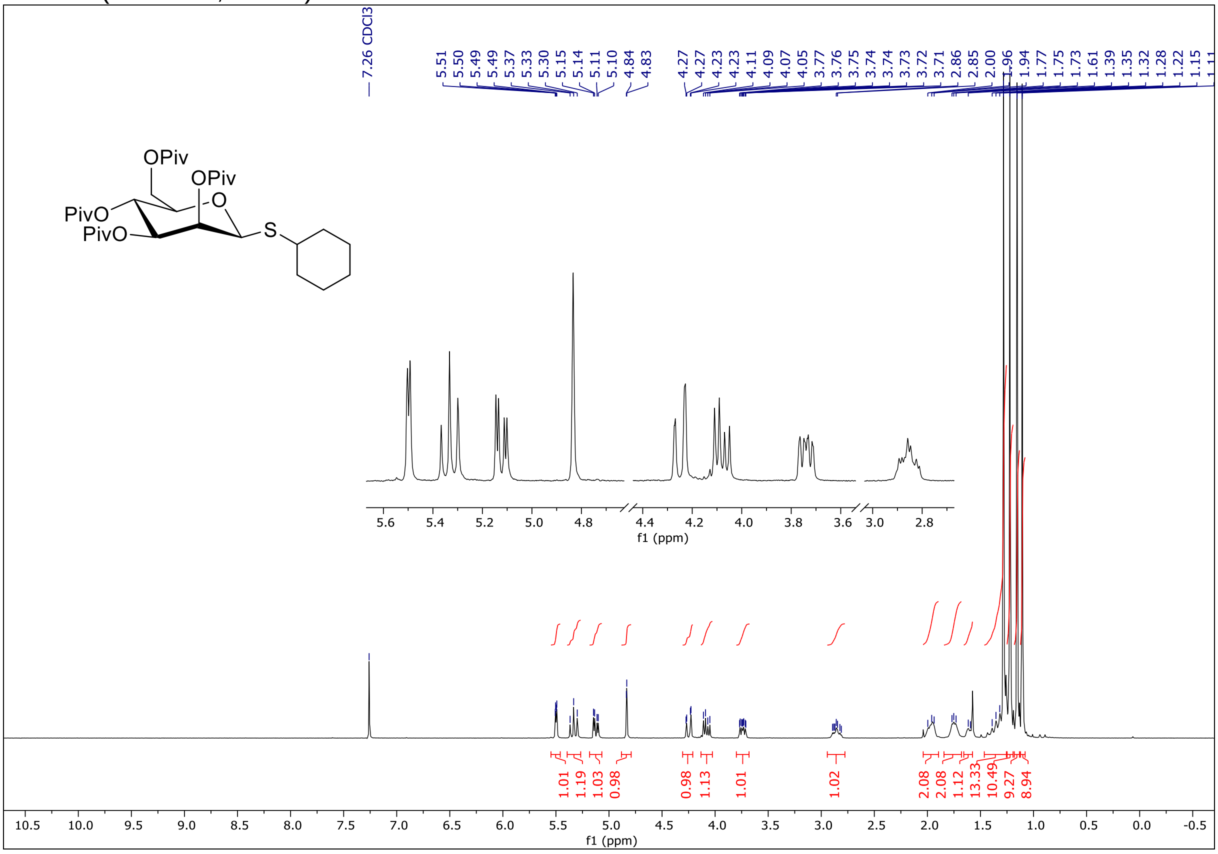

¹³C NMR (75.5 MHz, $CDCl_3$)

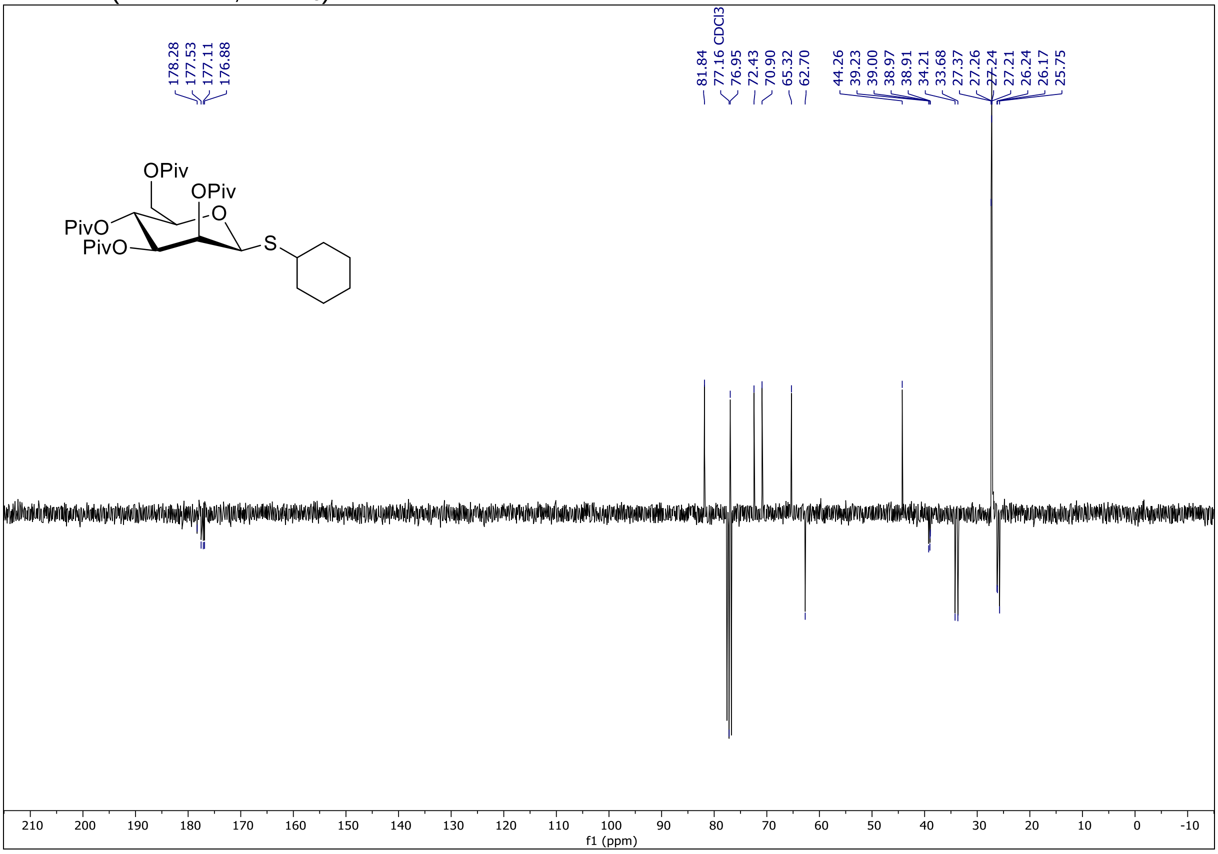

α-**3p**

^{1}H NMR (500 MHz, $CDCl_3$)

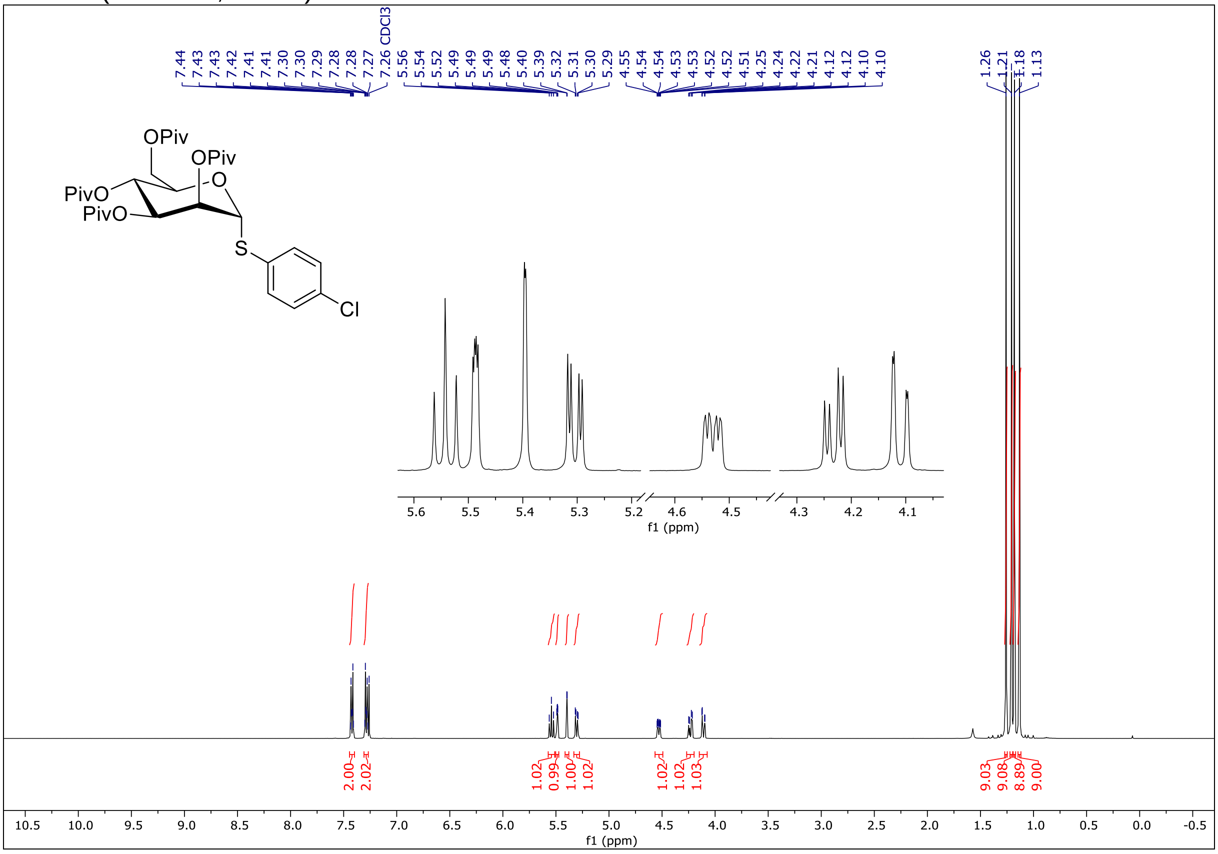

^{13}C NMR (126 MHz, $CDCl_3$)

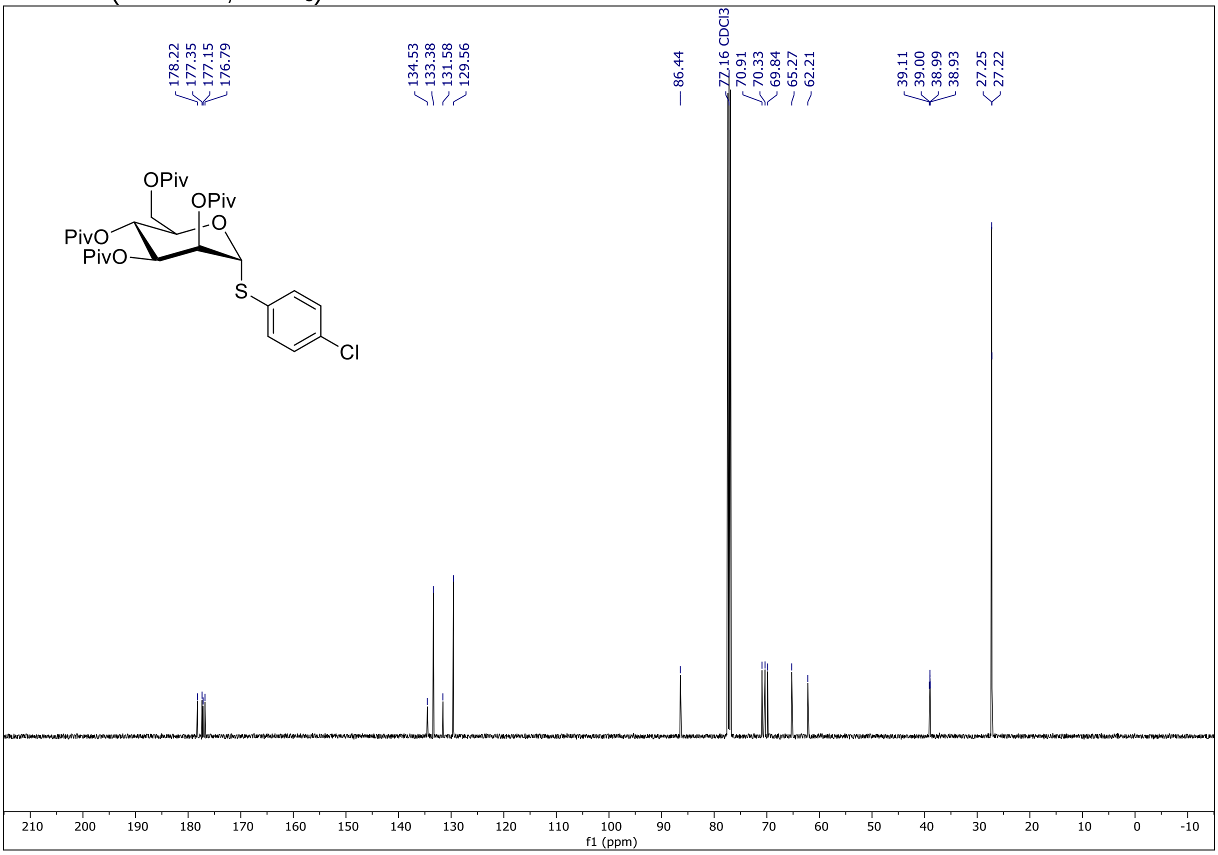

β-3p

¹H NMR (500 MHz, CDCl₃)

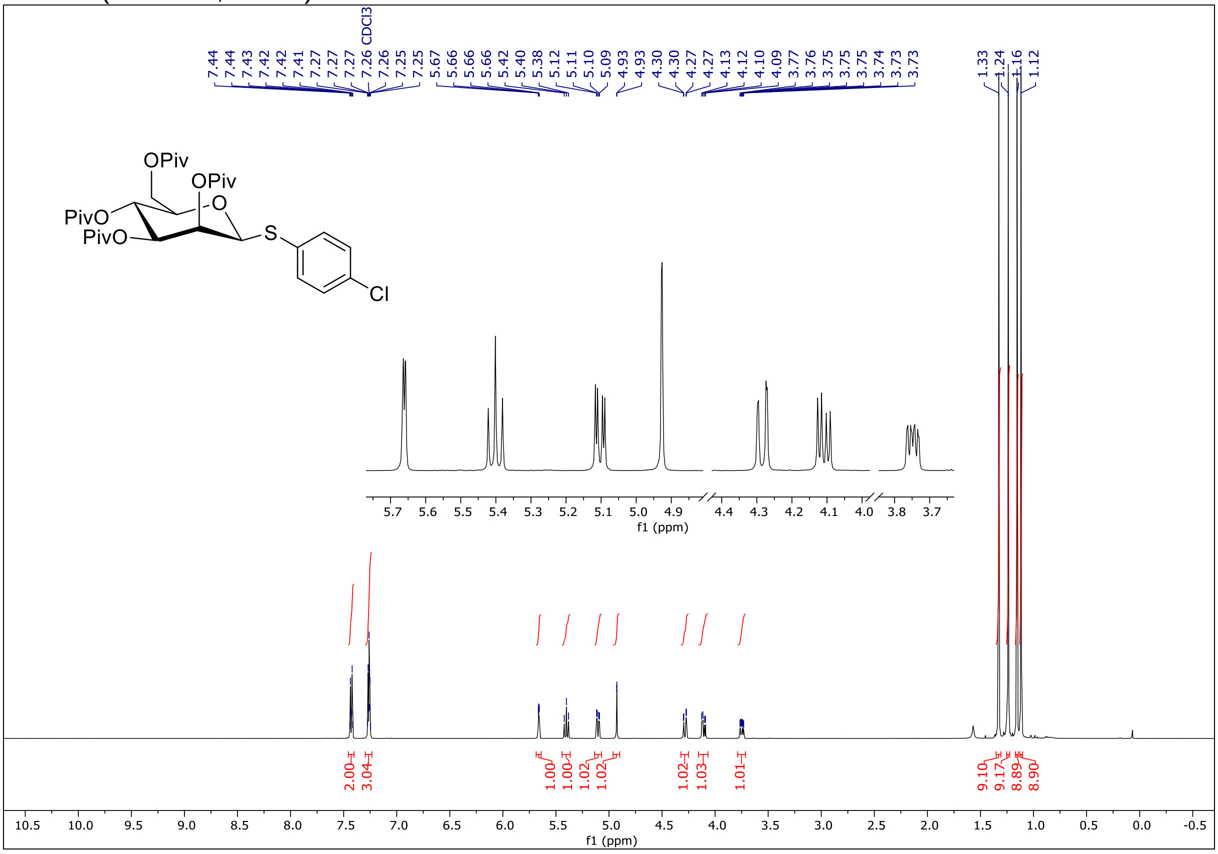

¹³C NMR (126 MHz, CDCl₃)

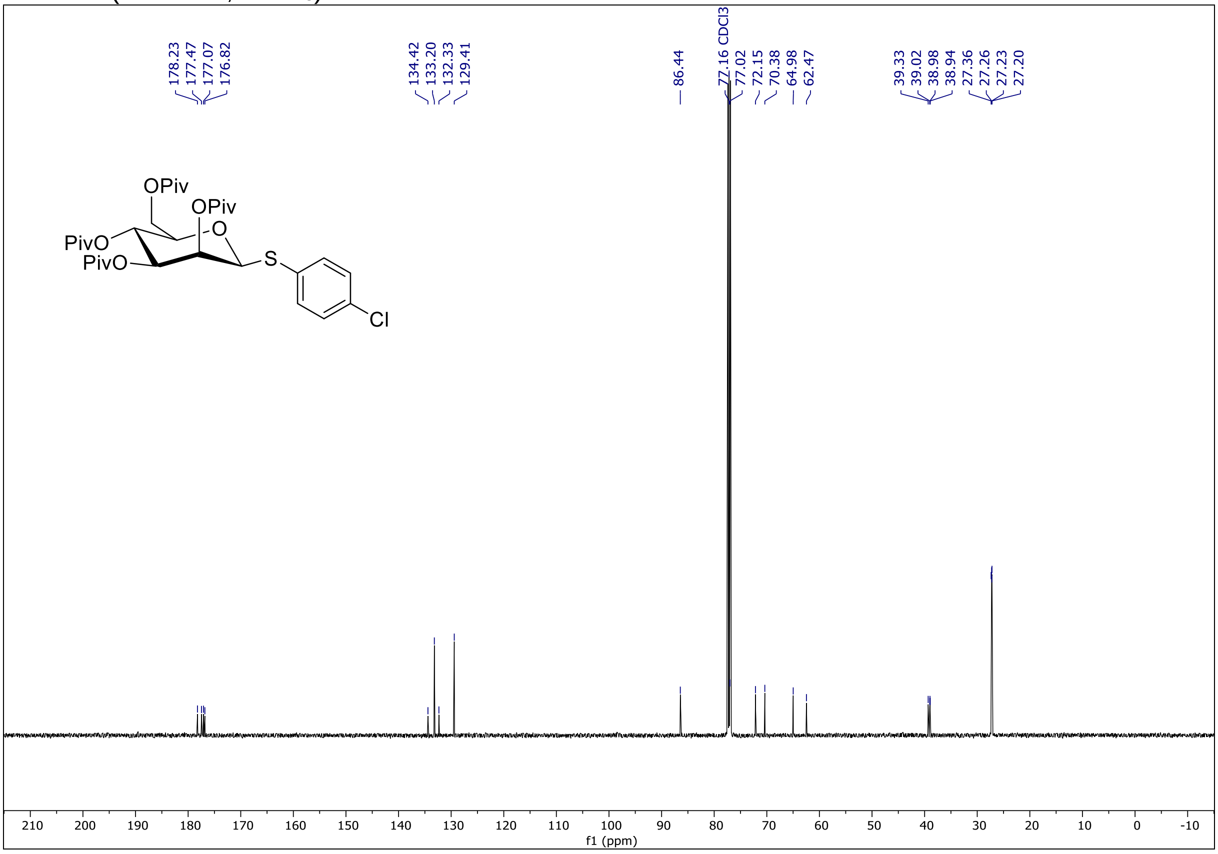

α-**3q**

^{1}H NMR (500 MHz, $CDCl_3$)

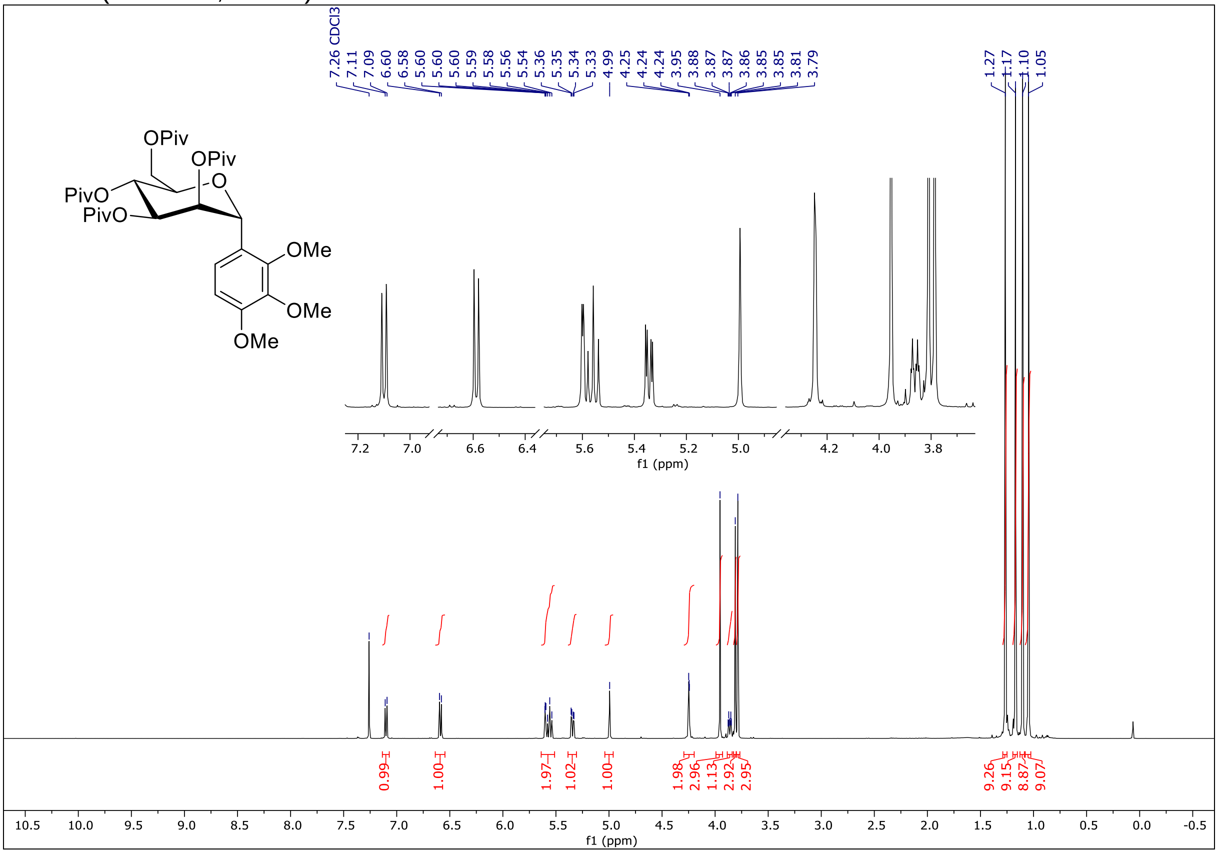

^{13}C NMR (126 MHz, $CDCl_3$)

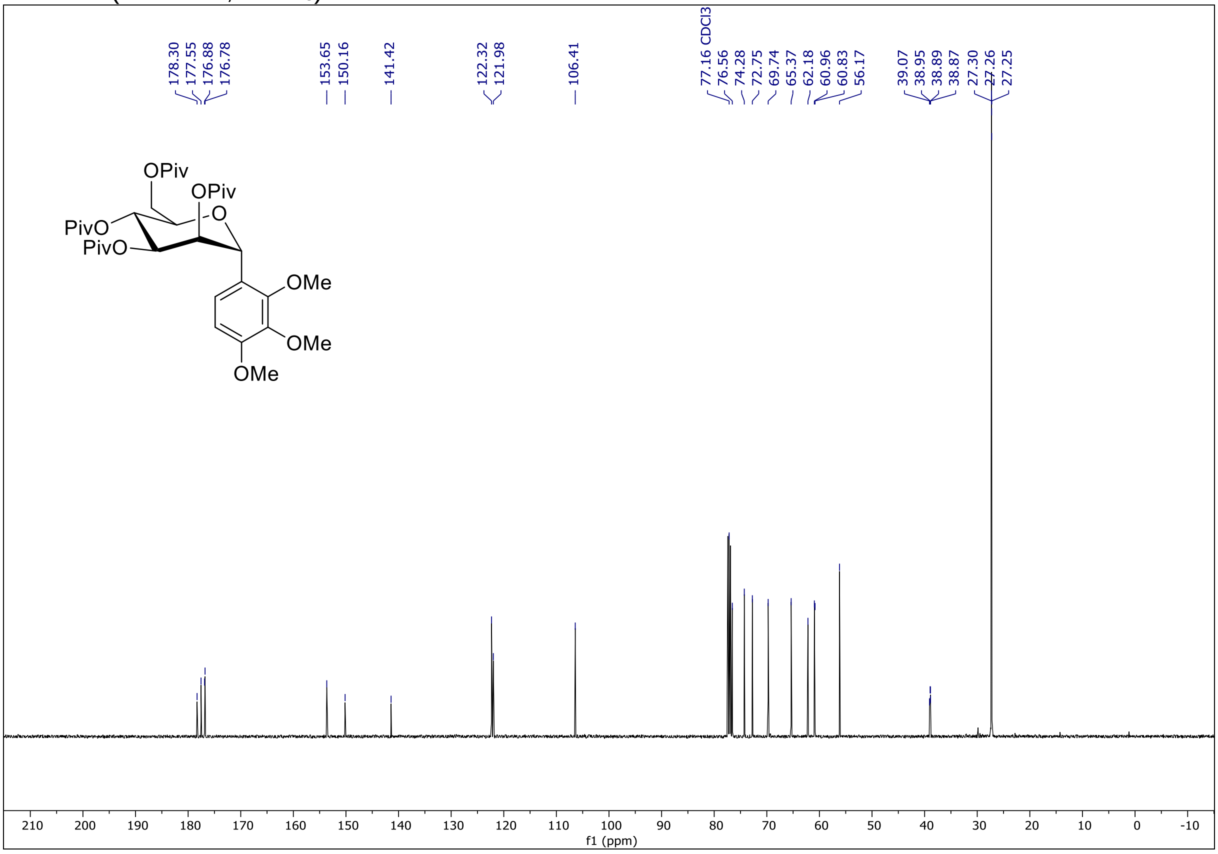

α-**3r**

^{1}H NMR (500 MHz, $CDCl_3$)

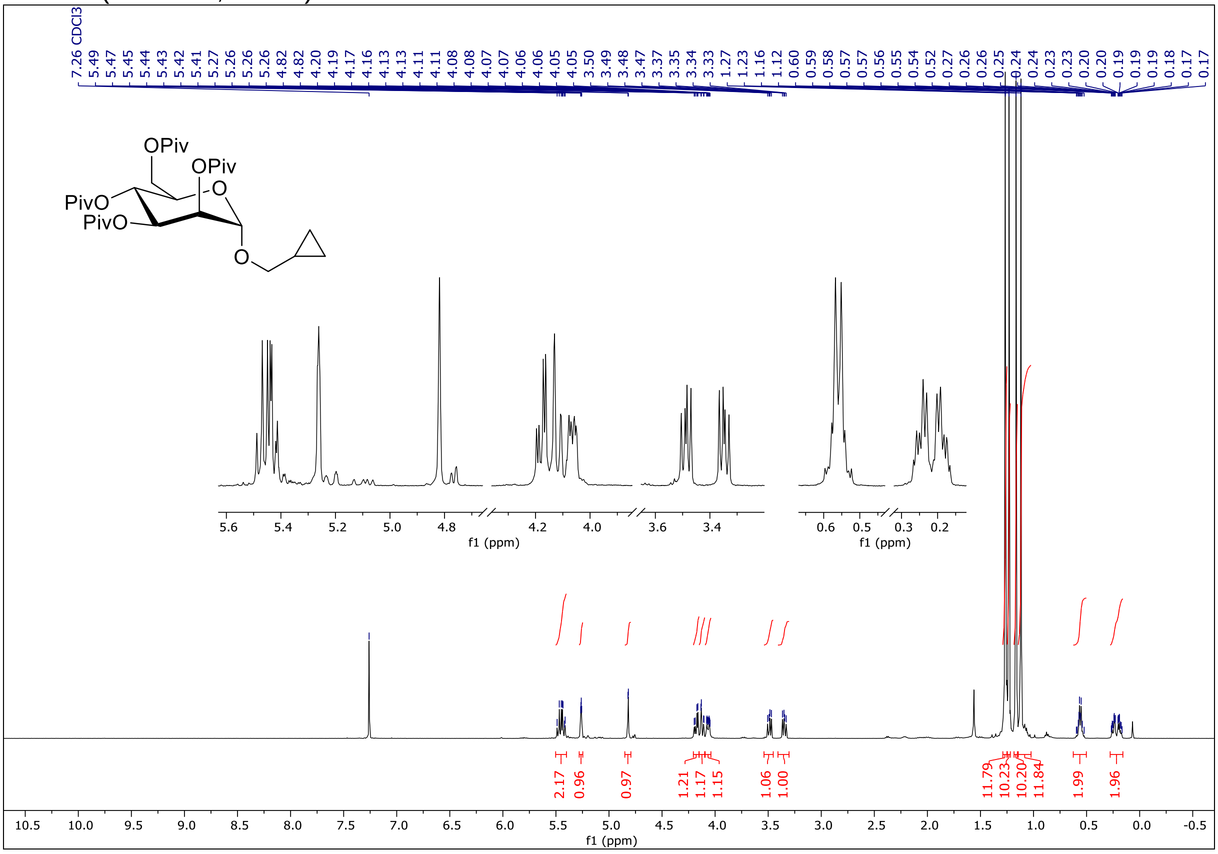

^{13}C NMR (126 MHz, $CDCl_3$)

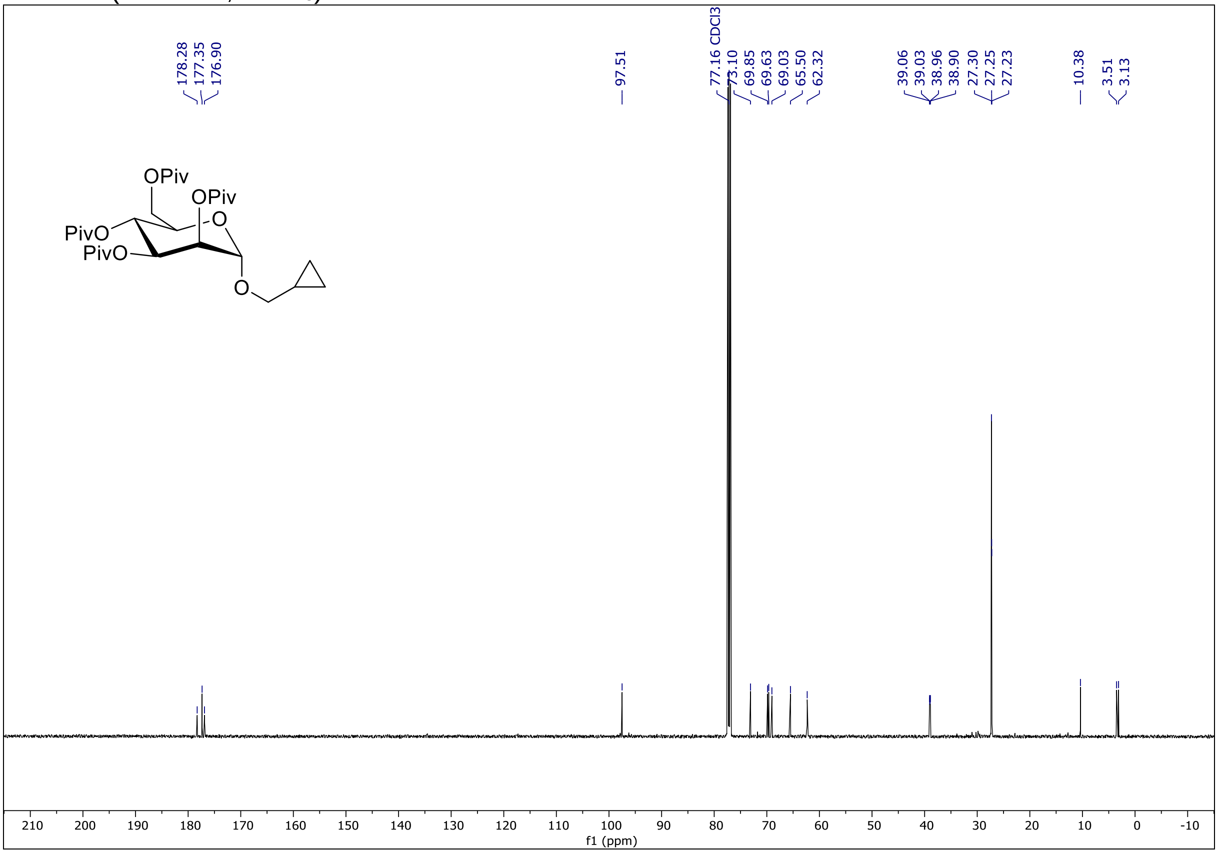

α-**3s**

^{1}H NMR (500 MHz, $CDCl_3$)

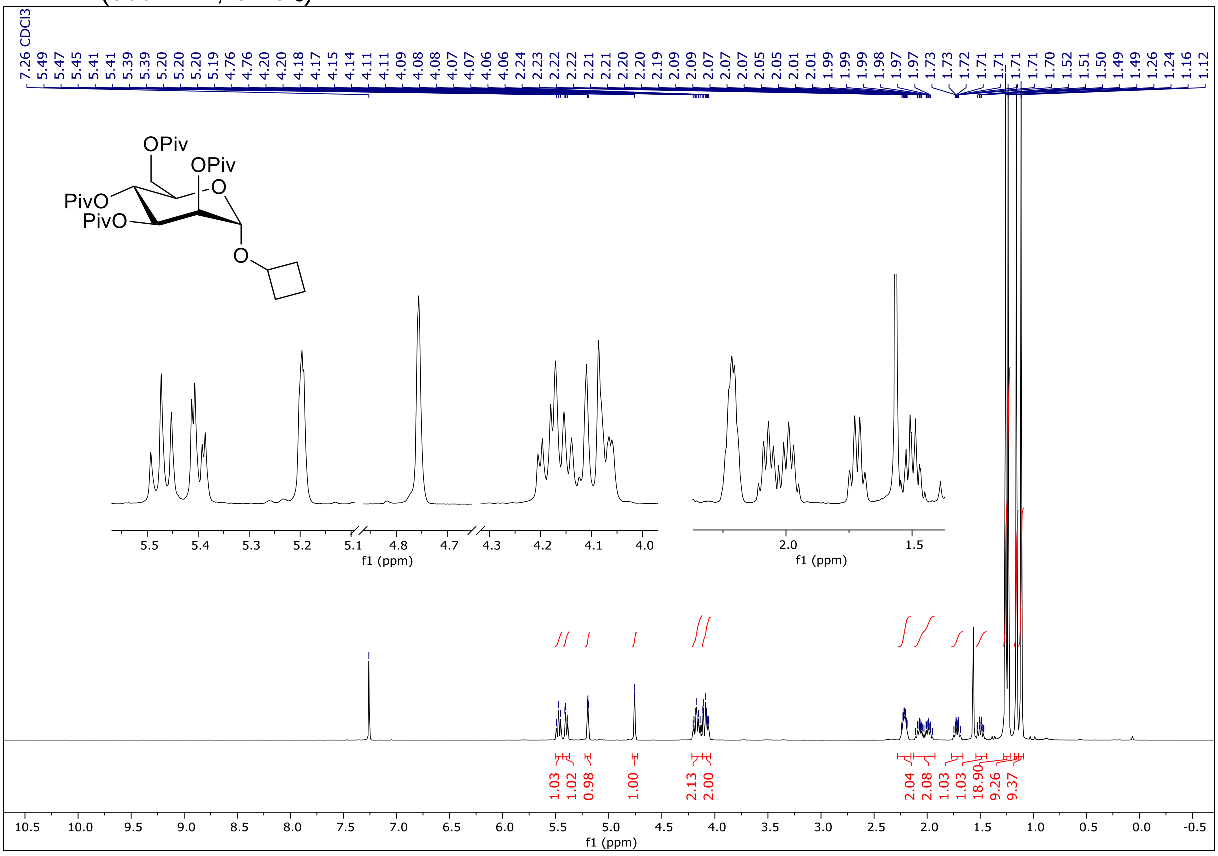

^{13}C NMR (126 MHz, $CDCl_3$)

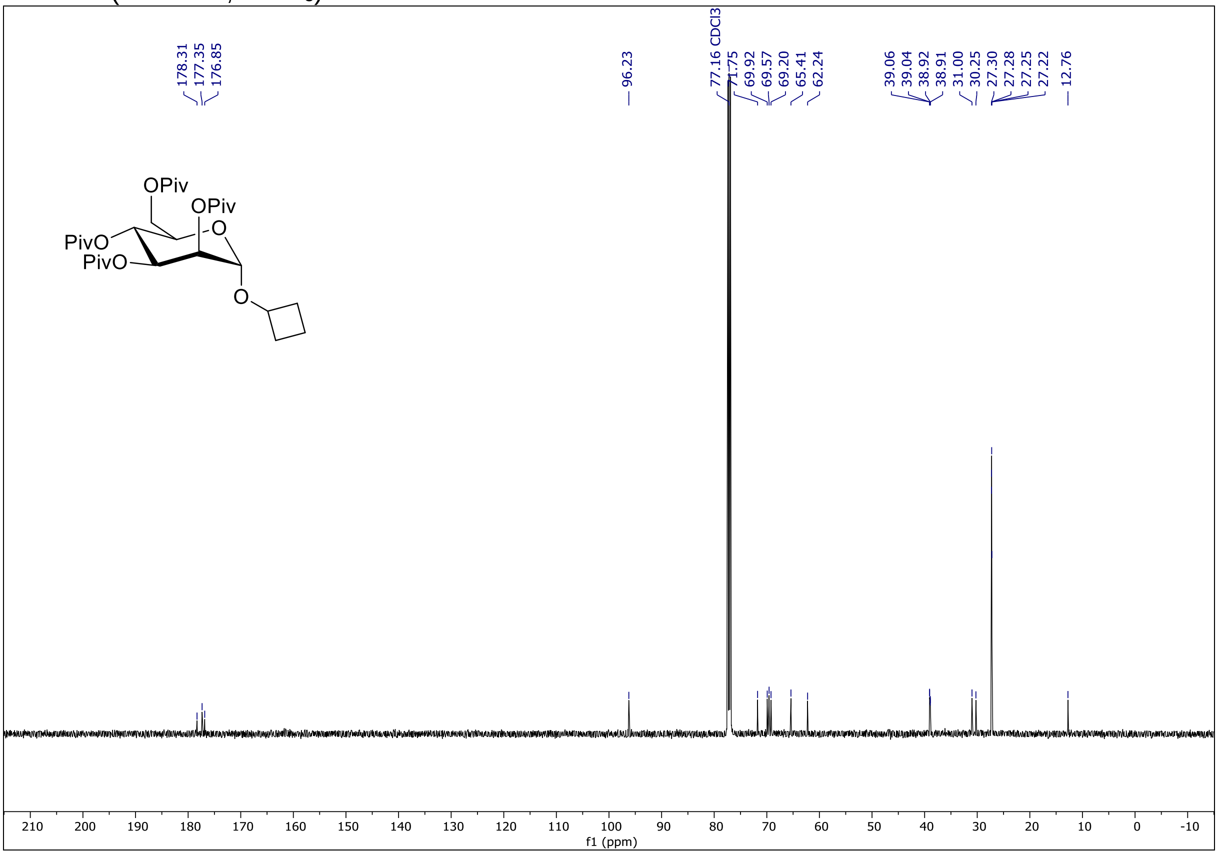

α-**8a**

^{1}H NMR (300 MHz, $CDCl_3$)

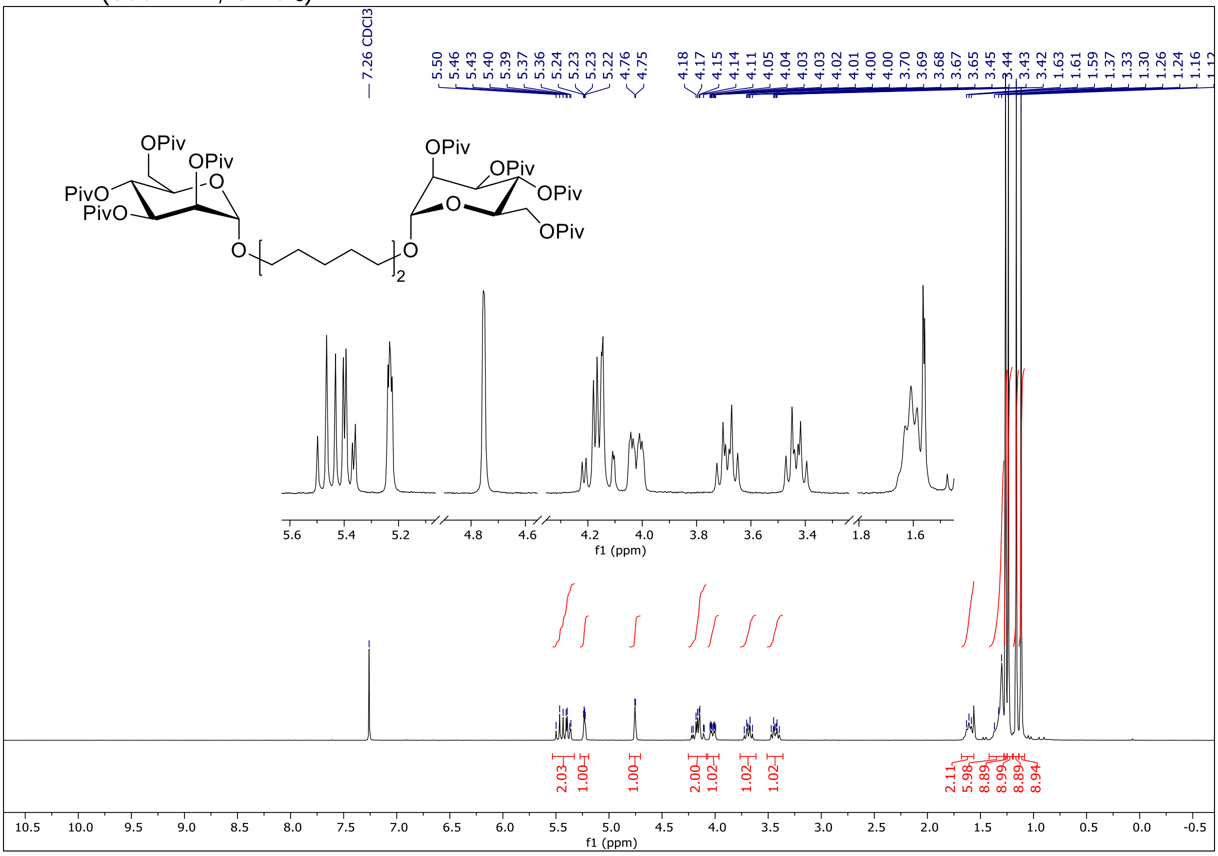

^{13}C NMR (75.5 MHz, $CDCl_3$)

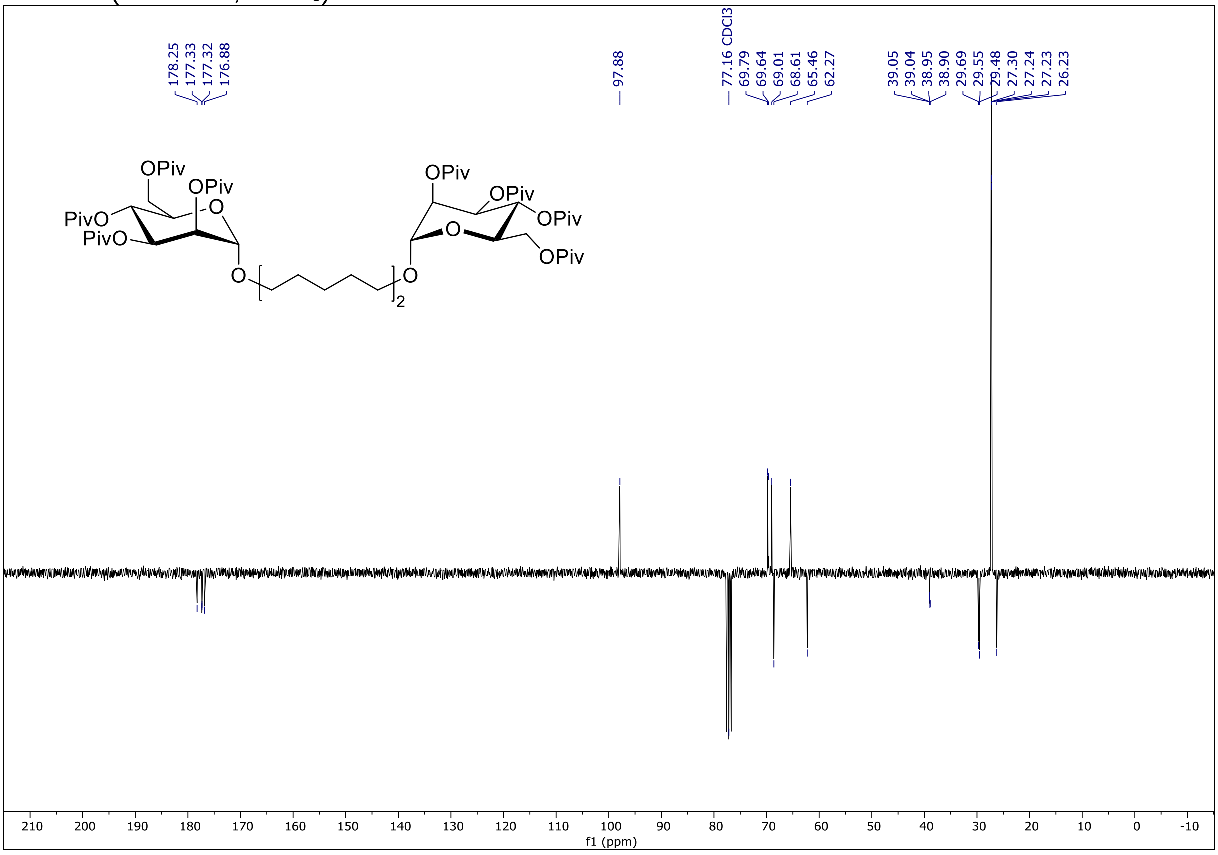

α-8b

^{1}H NMR (300 MHz, $CDCl_3$)

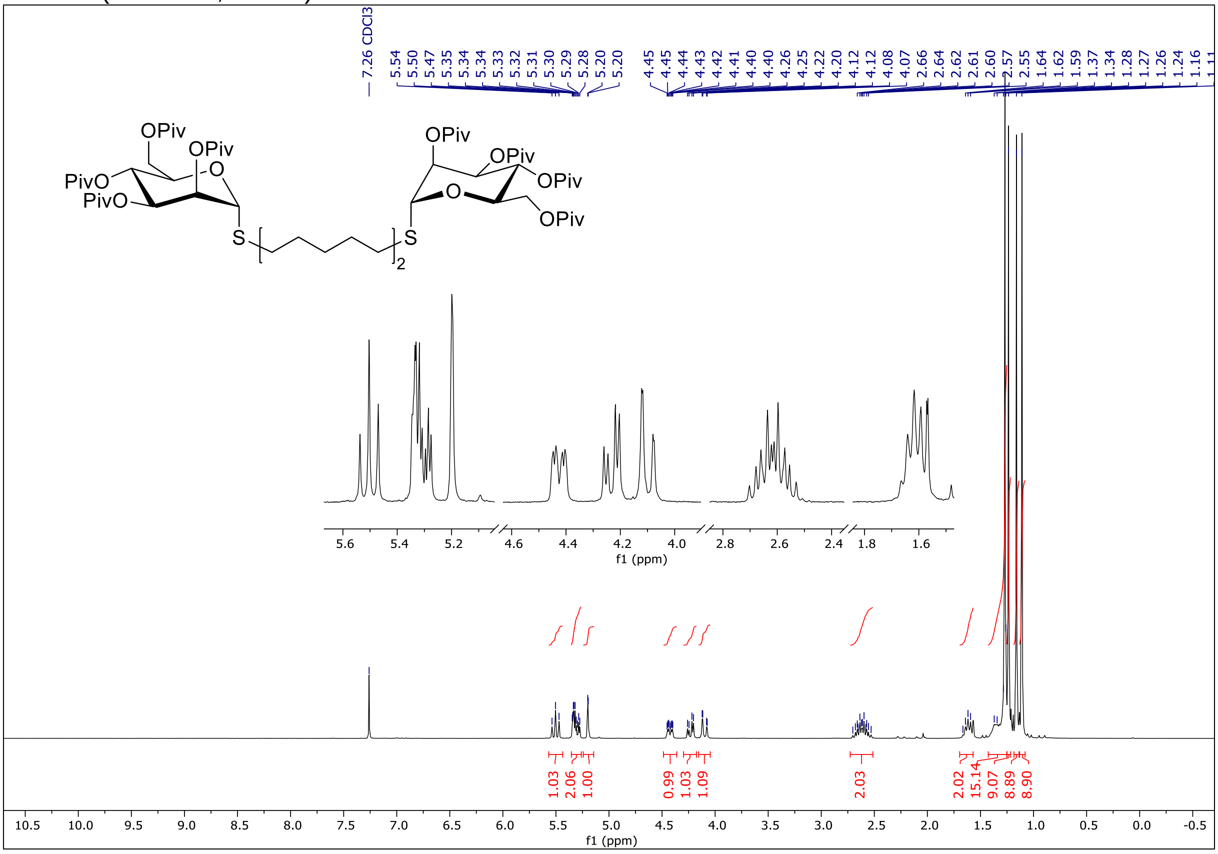

^{13}C NMR (75.5 MHz, $CDCl_3$)

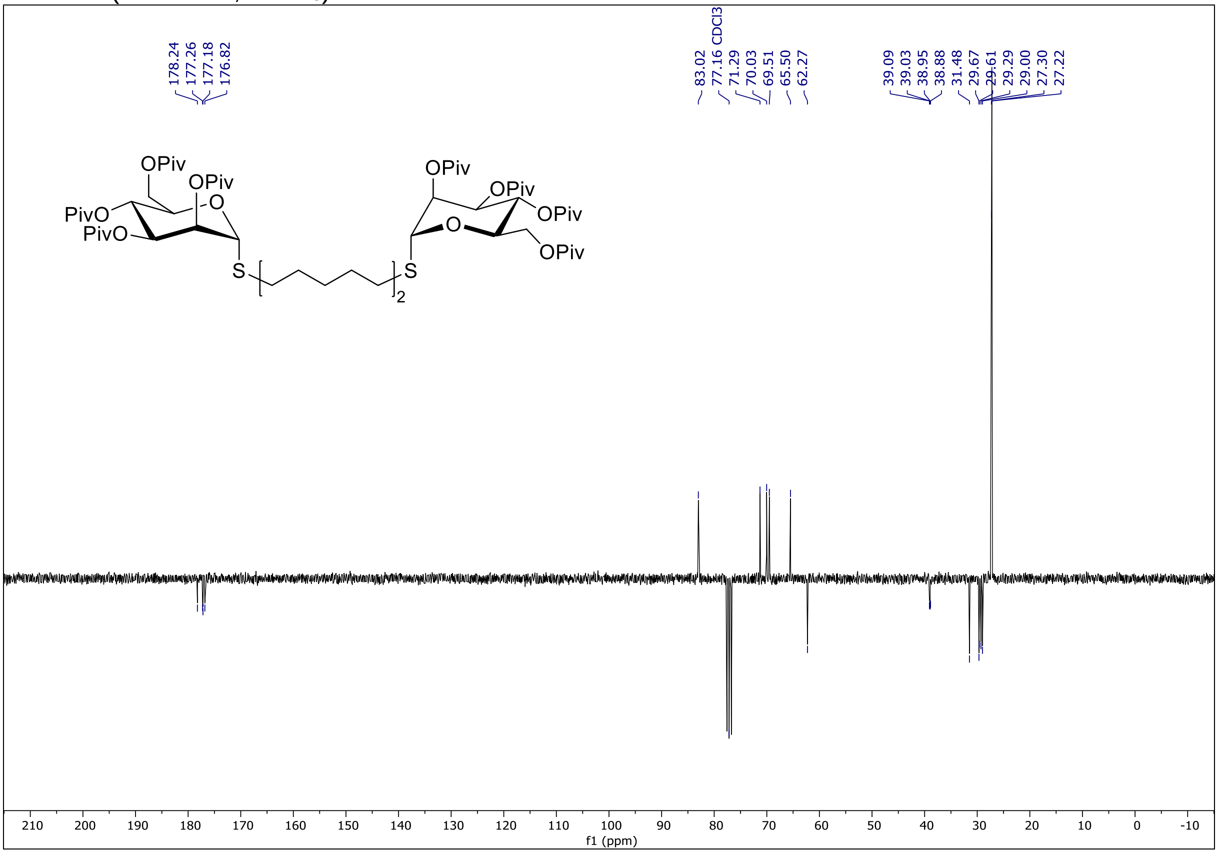

α-**10a**

^{1}H NMR (500 MHz, $CDCl_3$)

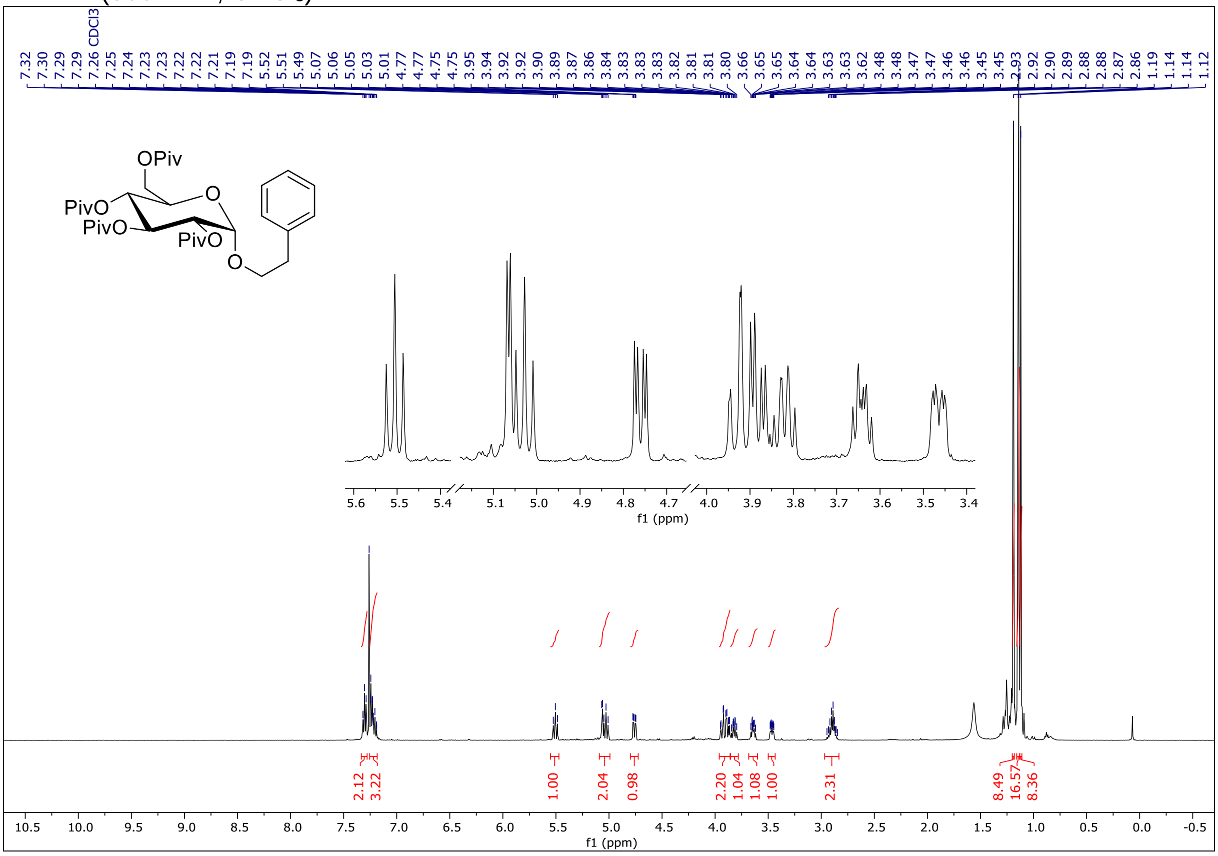

^{13}C NMR (126 MHz, $CDCl_3$)

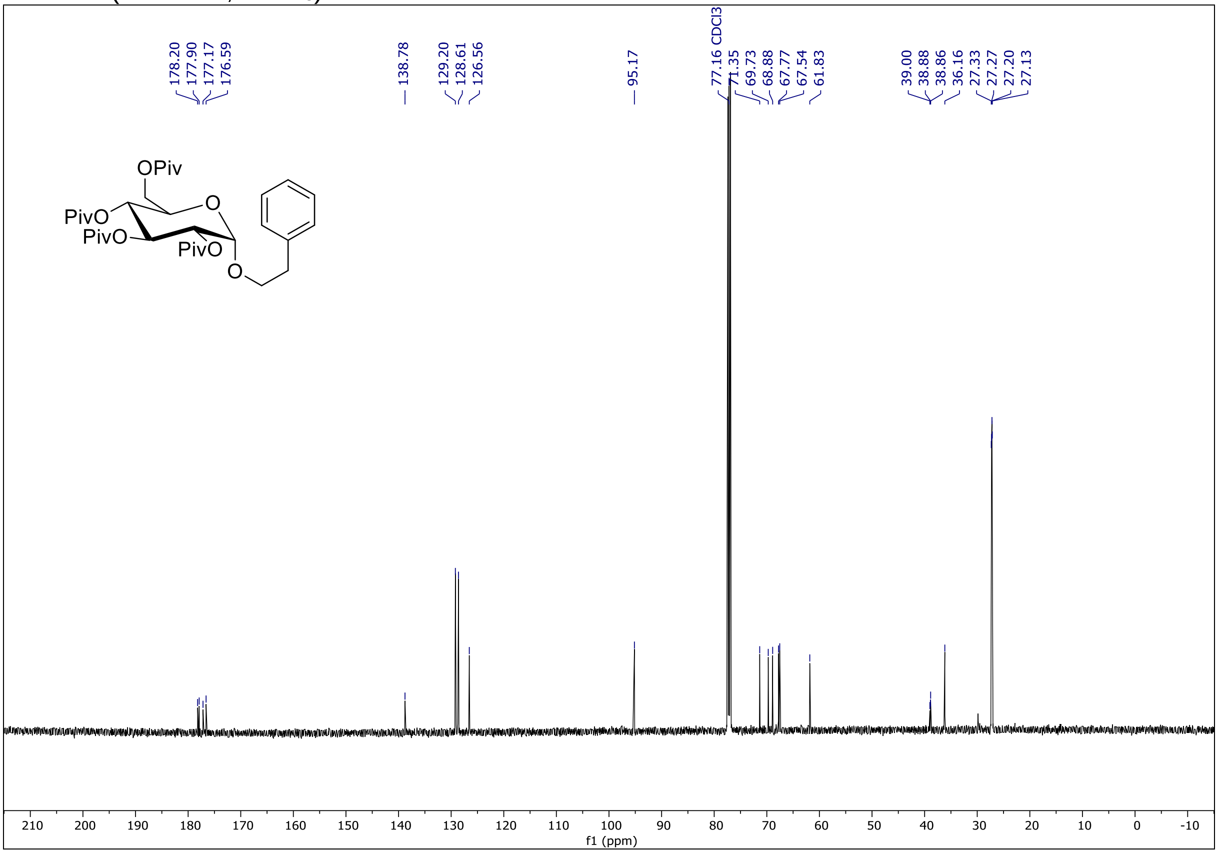

β-10a

^1H NMR (500 MHz, $CDCl_3$)

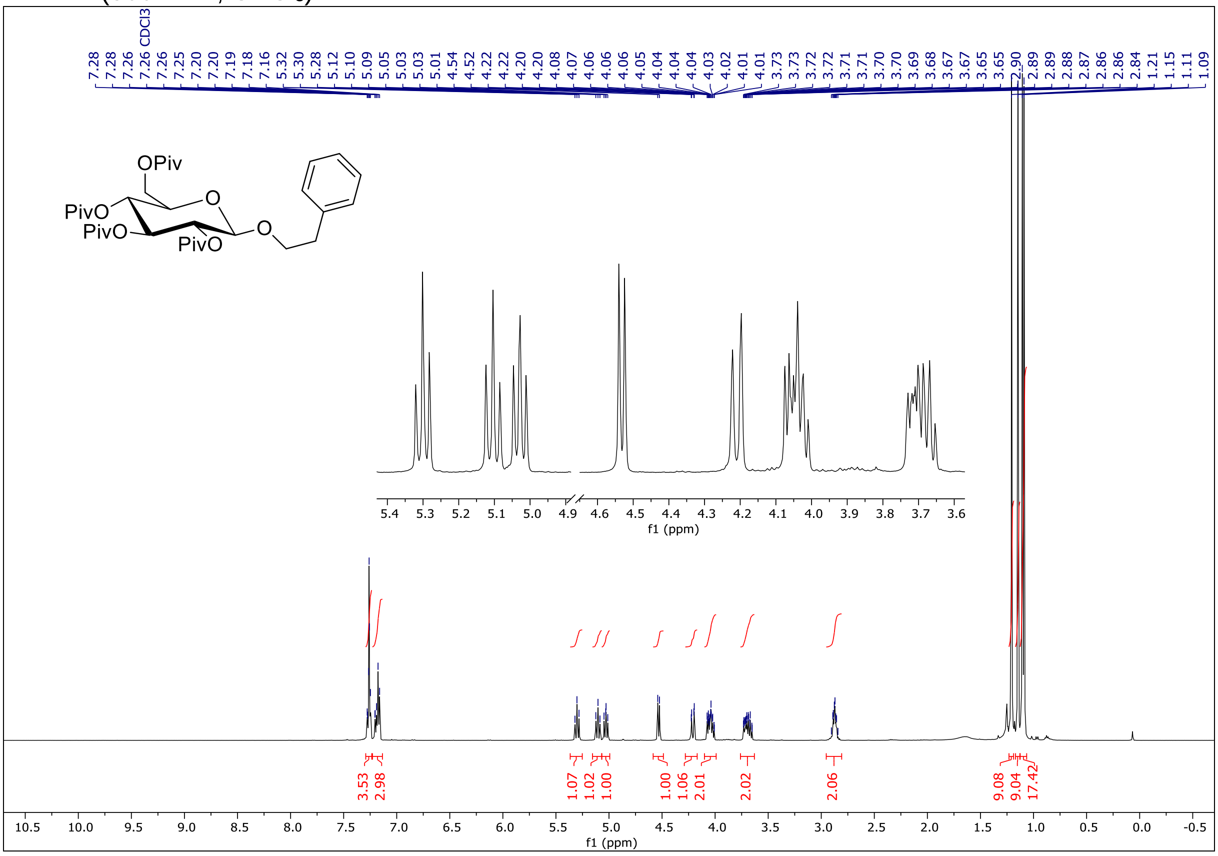

^{13}C NMR (126 MHz, $CDCl_3$)

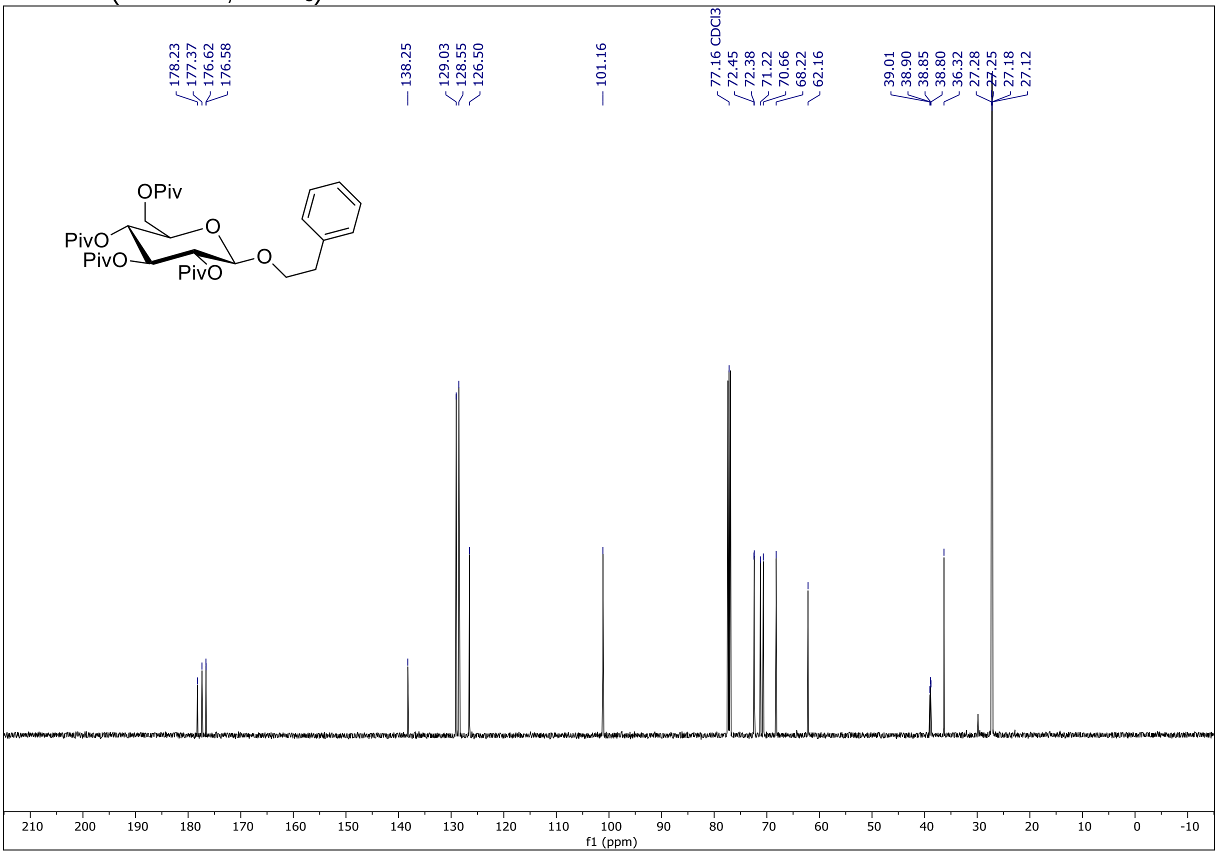

α-**10b**

^{1}H NMR (500 MHz, $CDCl_3$)

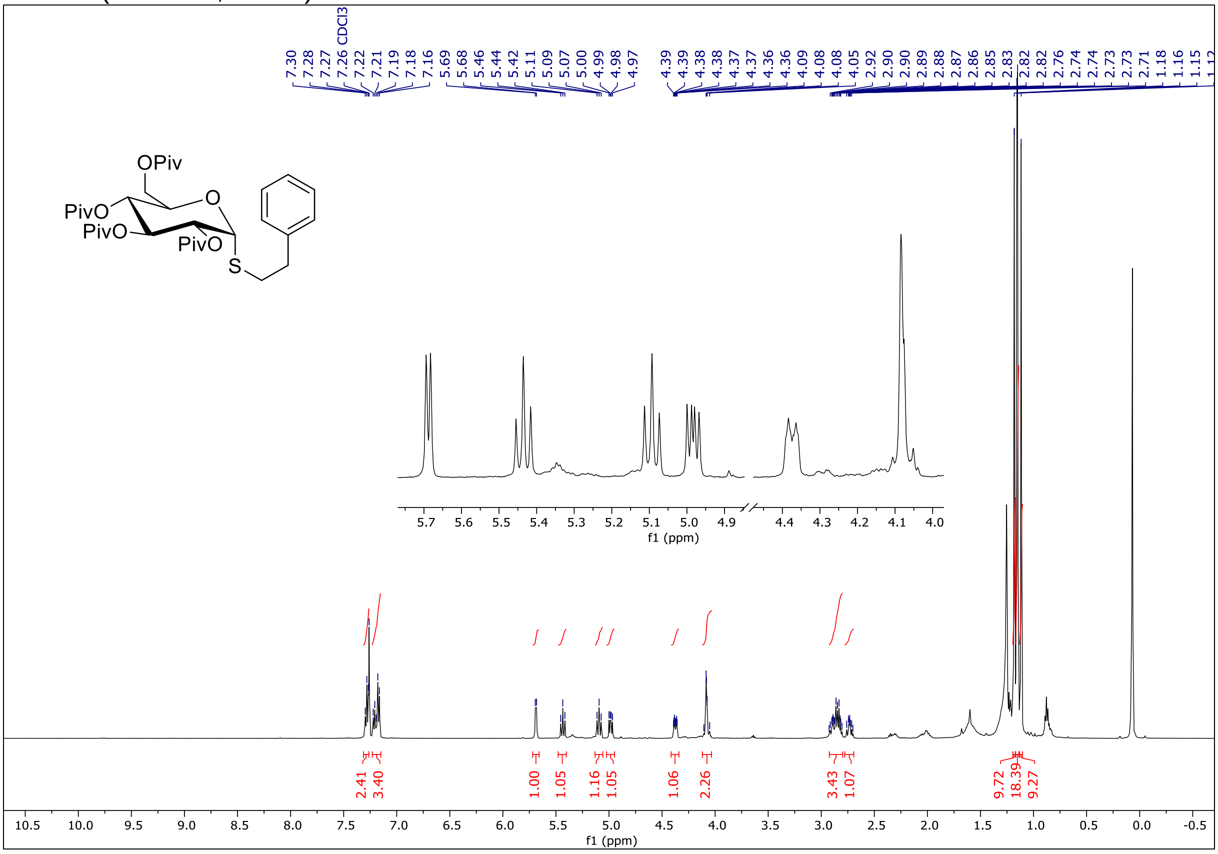

^{13}C NMR (126 MHz, $CDCl_3$)

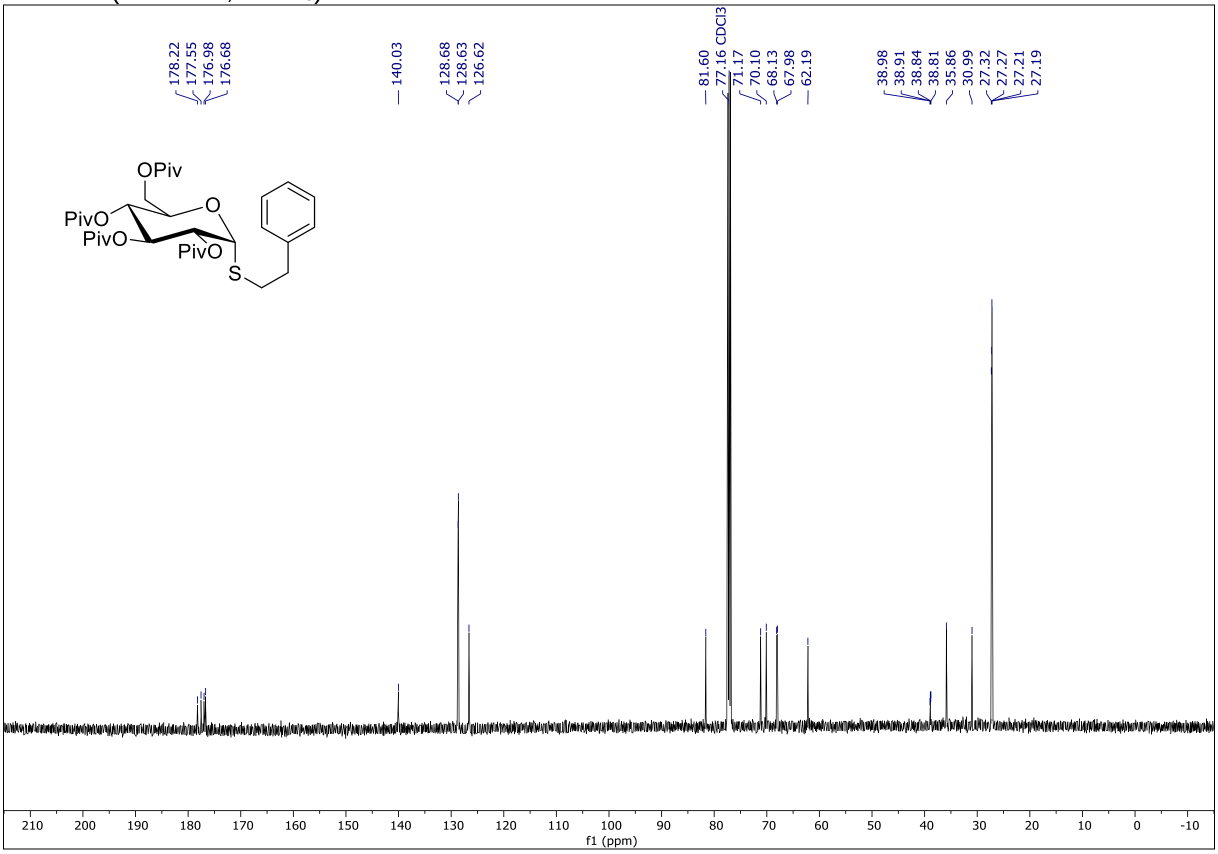

β-10b

^{1}H NMR (500 MHz, $CDCl_3$)

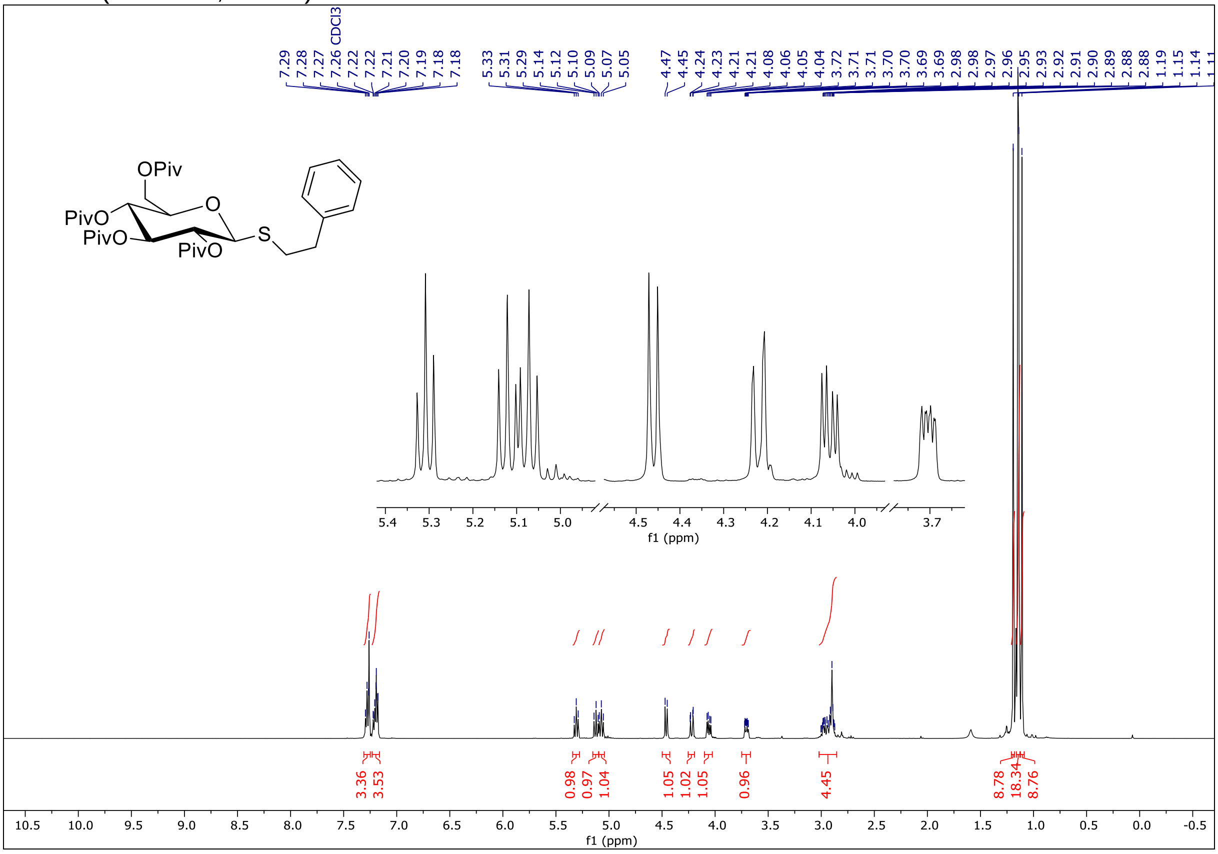

^{13}C NMR (126 MHz, $CDCl_3$)

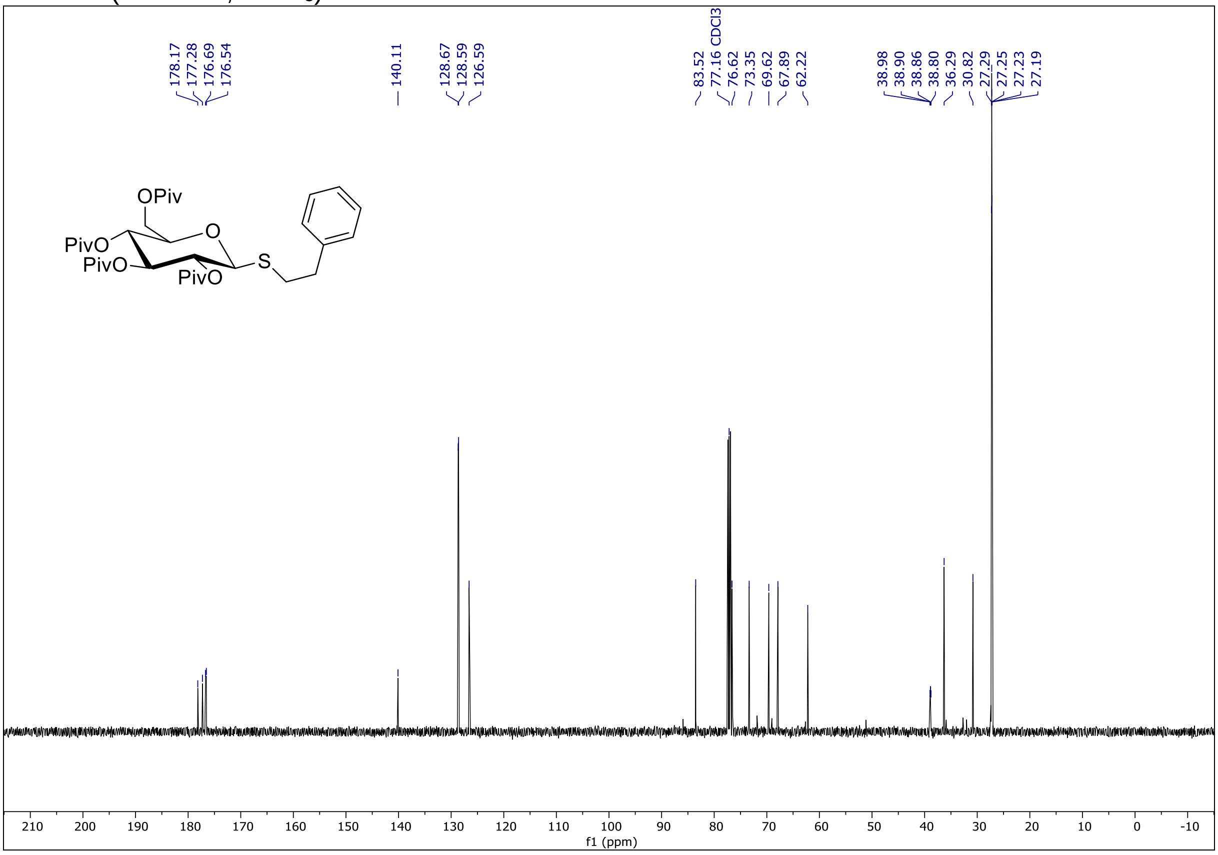

α-**13a**

^{1}H NMR (500 MHz, $CDCl_3$)

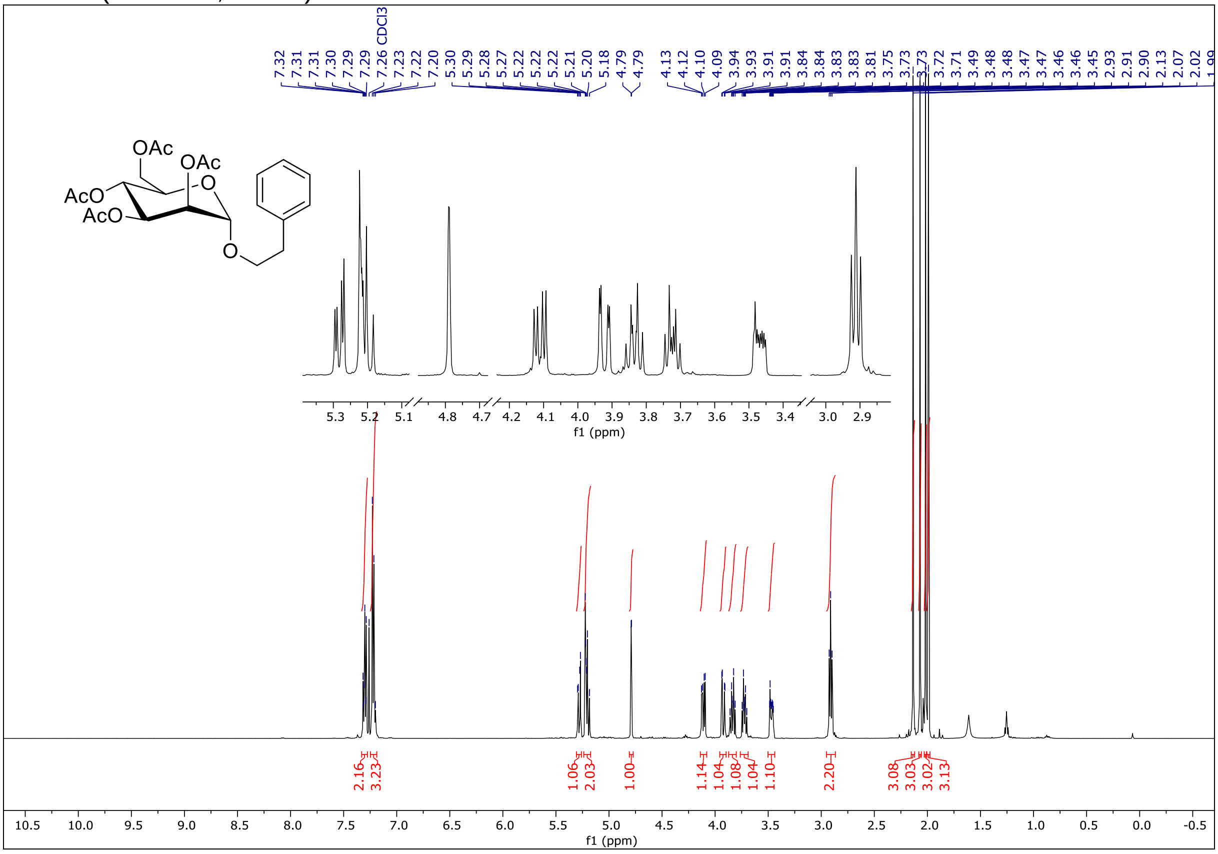

^{13}C NMR (126 MHz, $CDCl_3$)

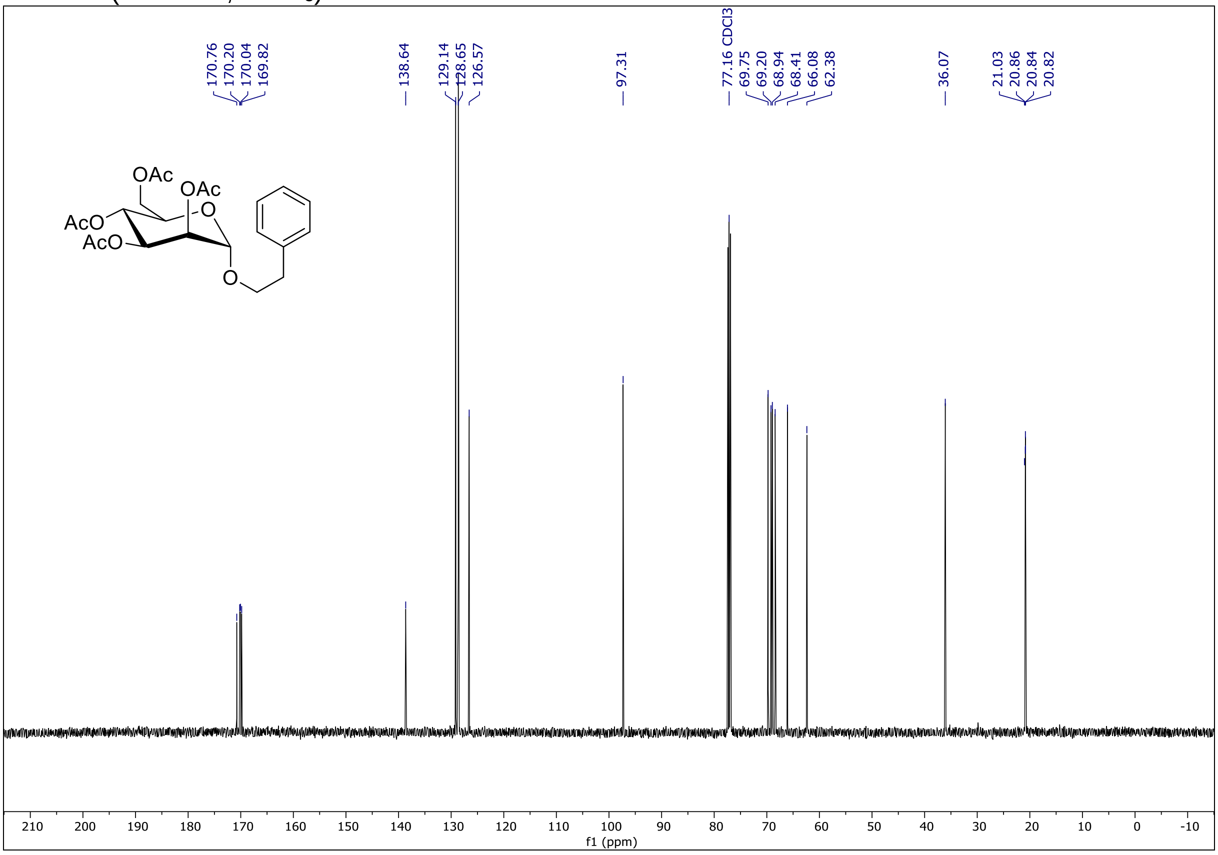

β-**13a**

^{1}H NMR (500 MHz, $CDCl_3$)

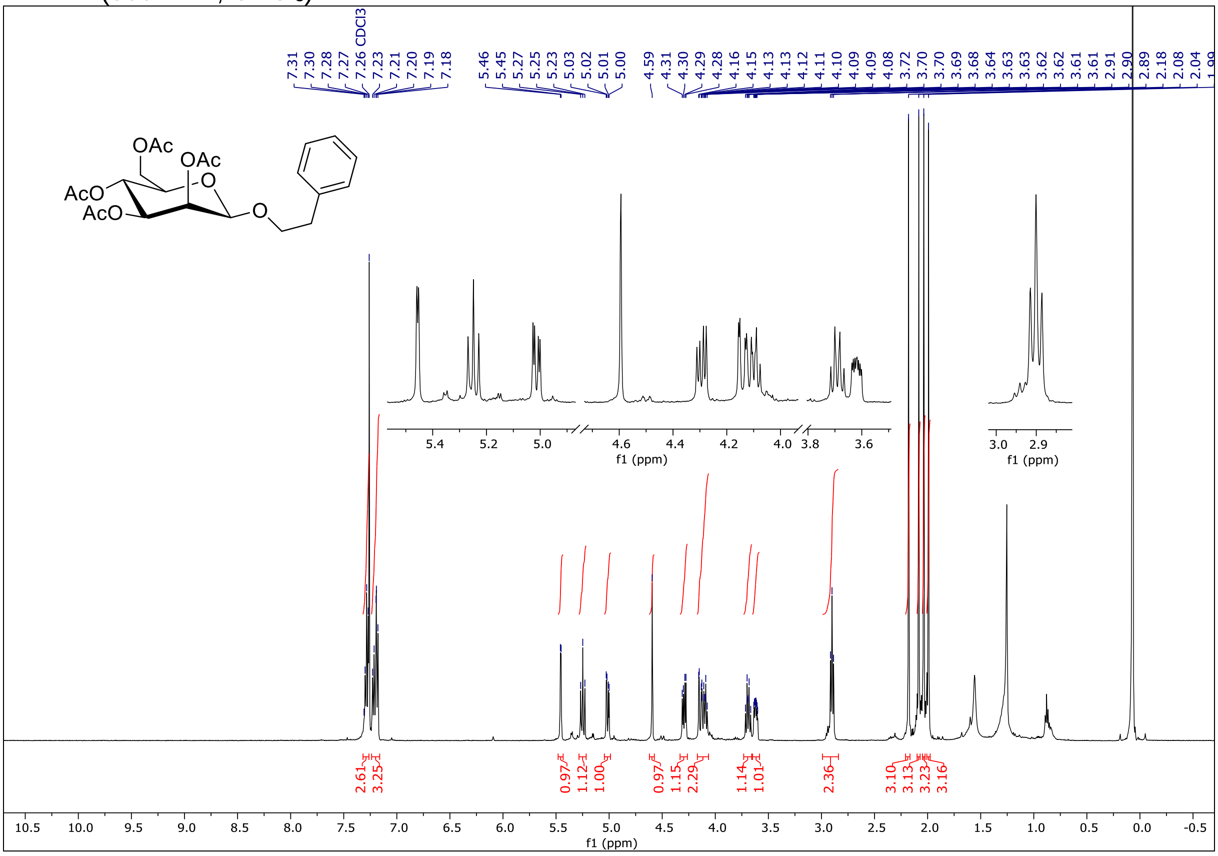

^{13}C NMR (126 MHz, $CDCl_3$)

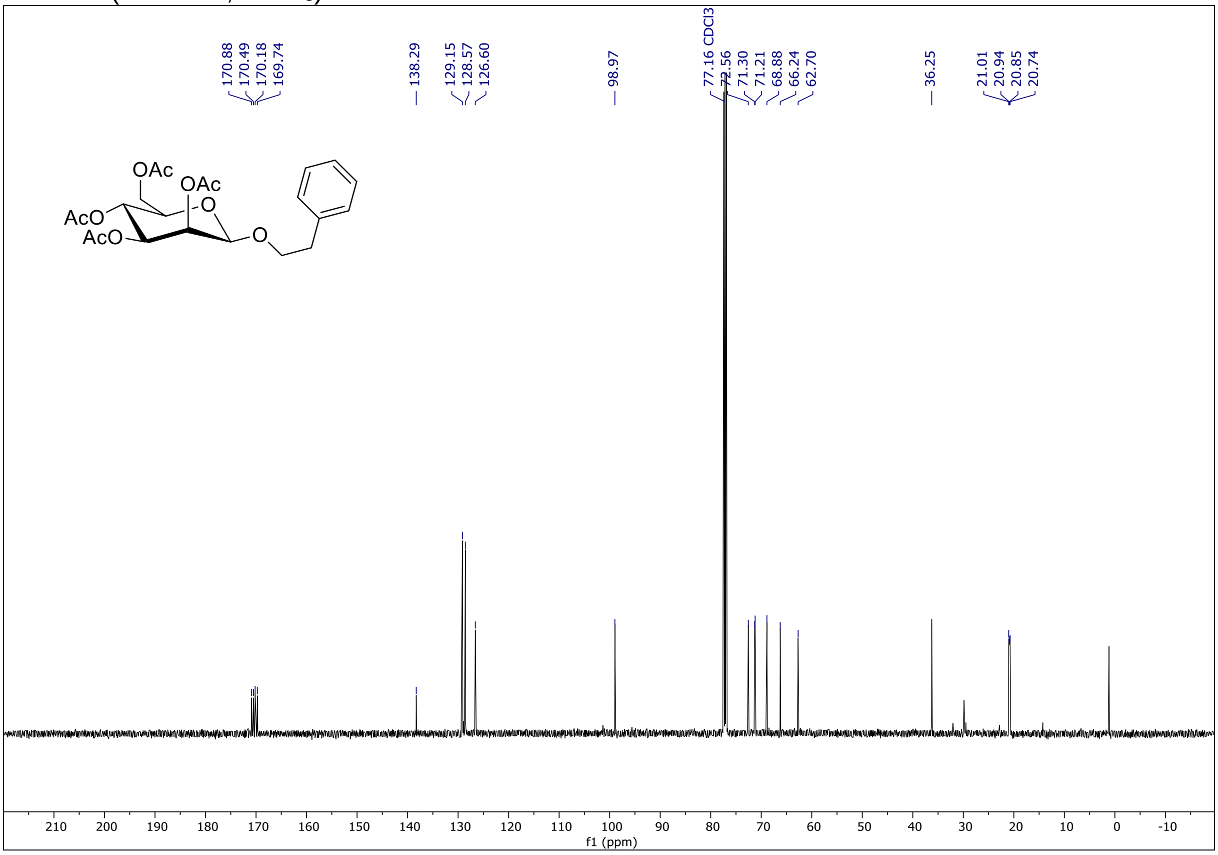

α-**13b**

^{1}H NMR (500 MHz, $CDCl_3$)

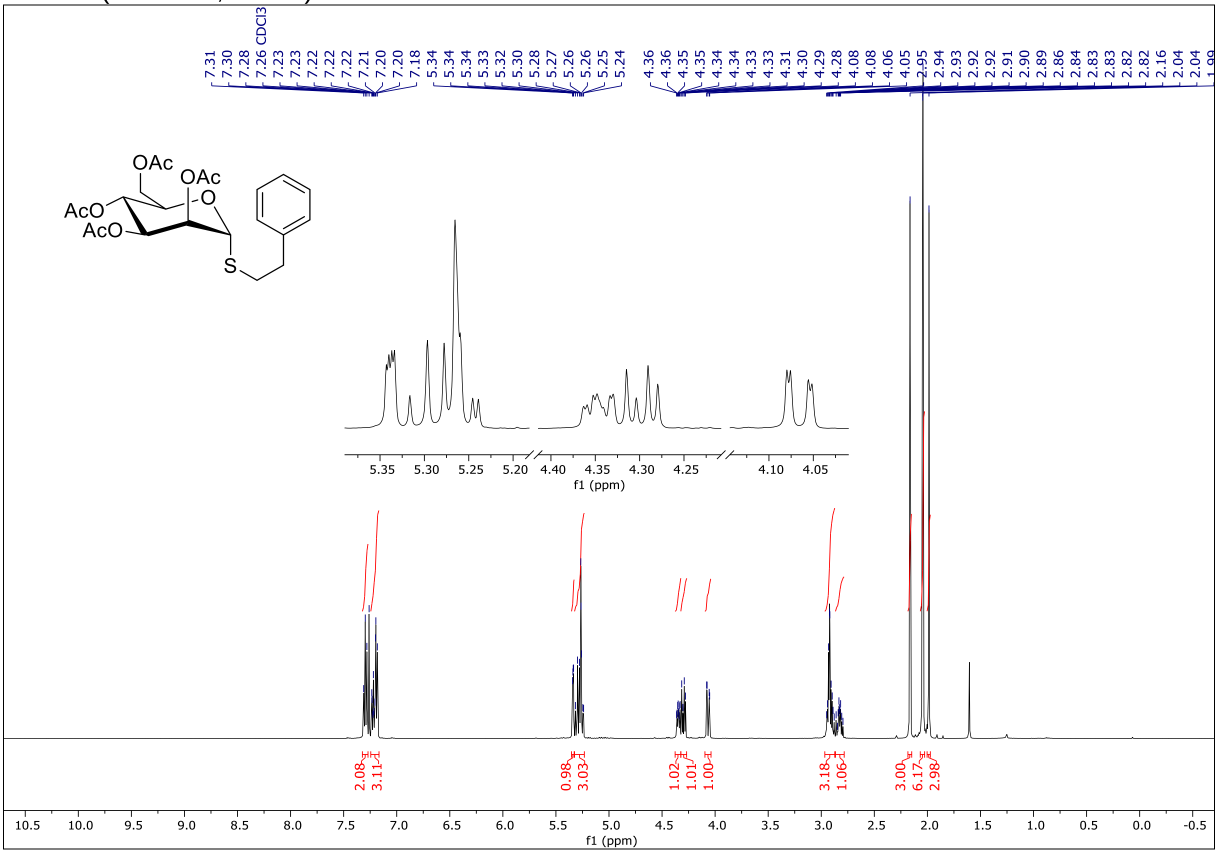

^{13}C NMR (126 MHz, $CDCl_3$)

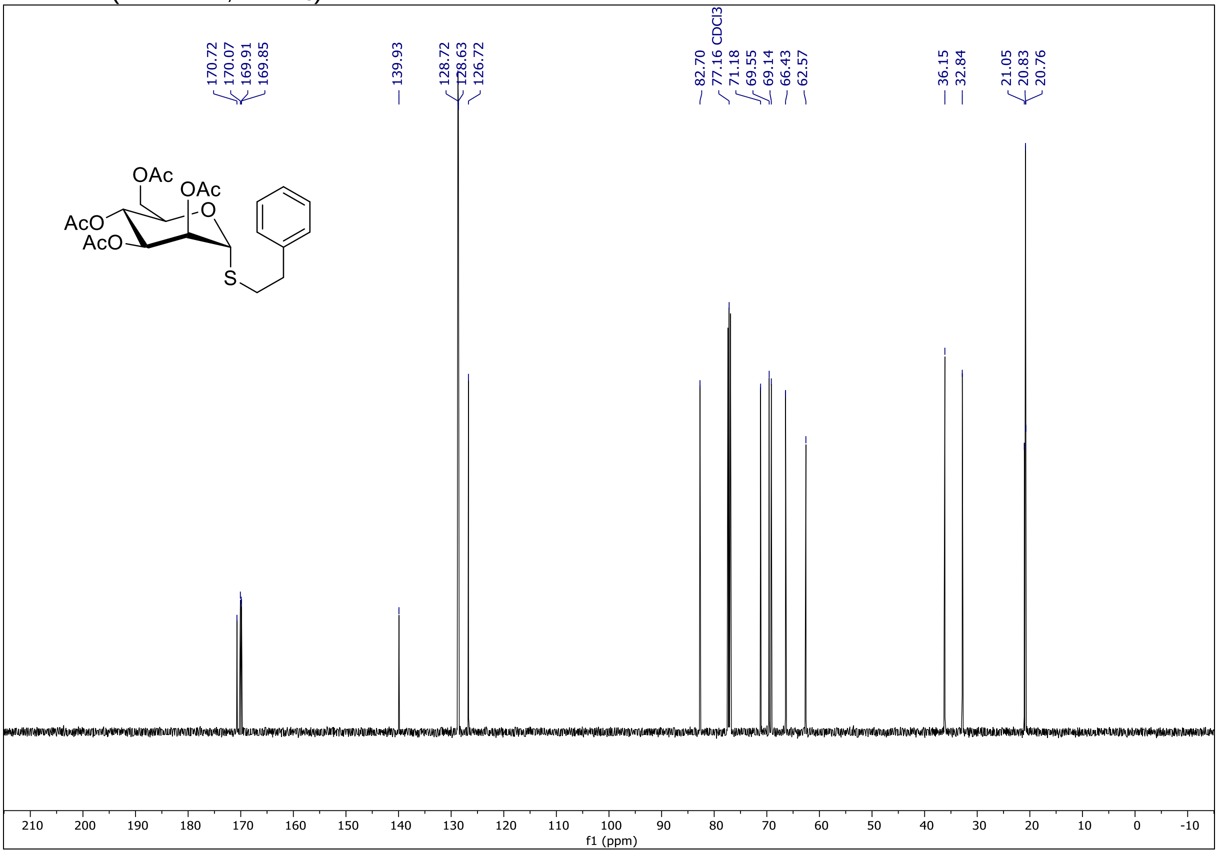

β-13b

^{1}H NMR (500 MHz, $CDCl_3$)

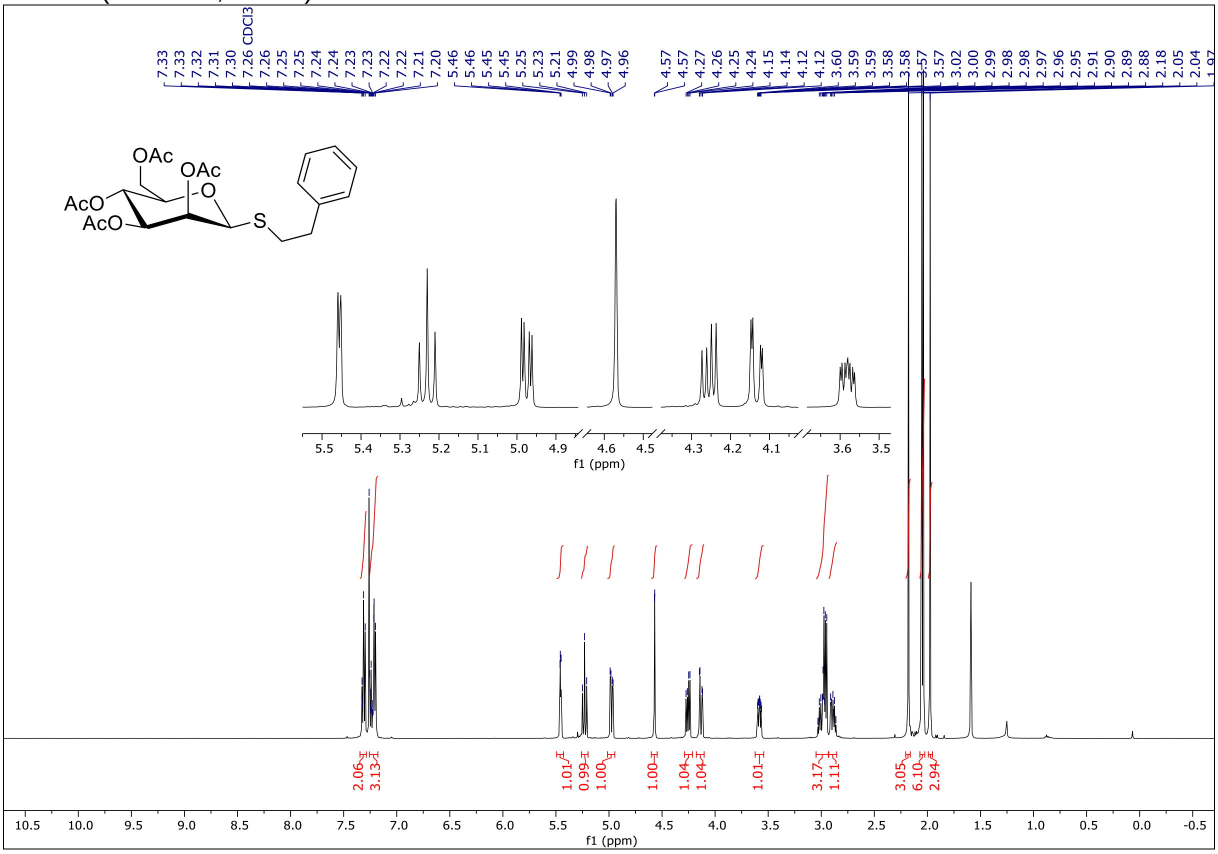

^{13}C NMR (126 MHz, $CDCl_3$)

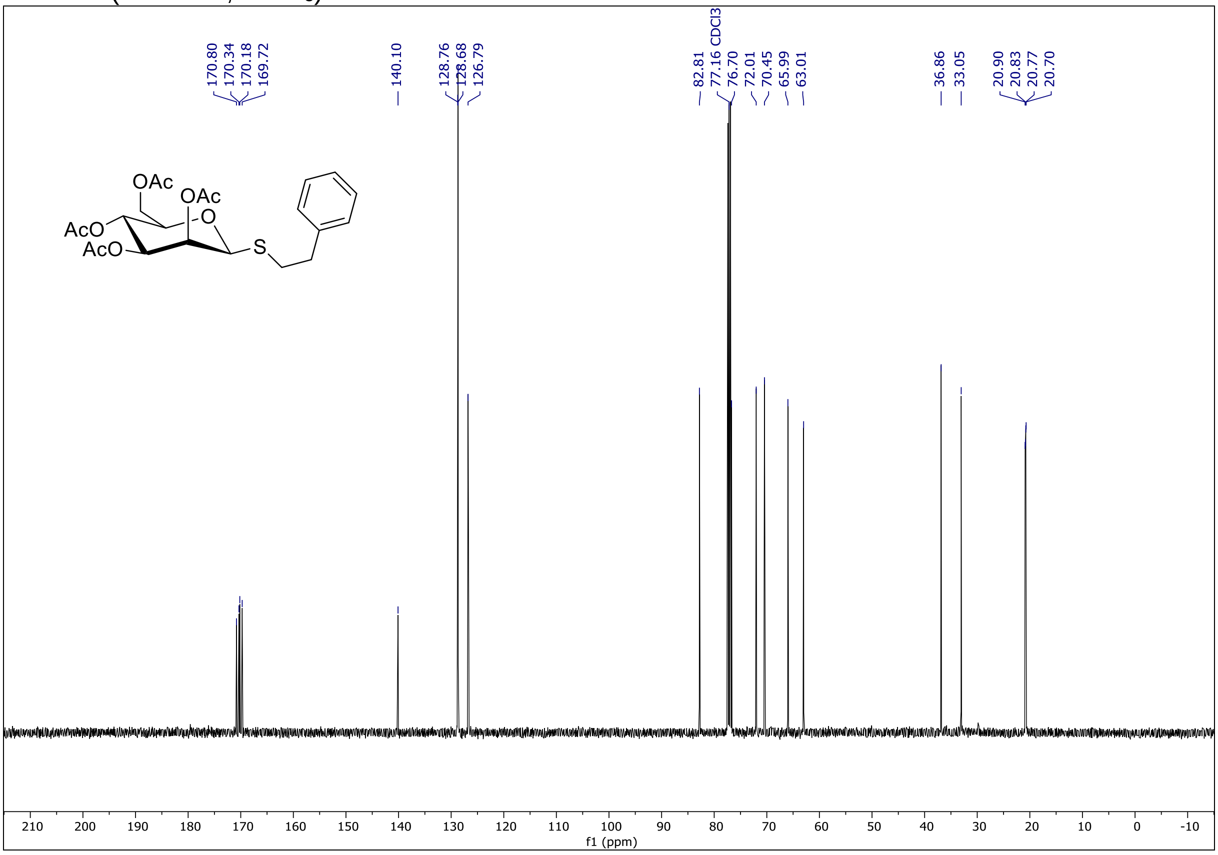

α-**14a**

^{1}H NMR (500 MHz, $CDCl_3$)

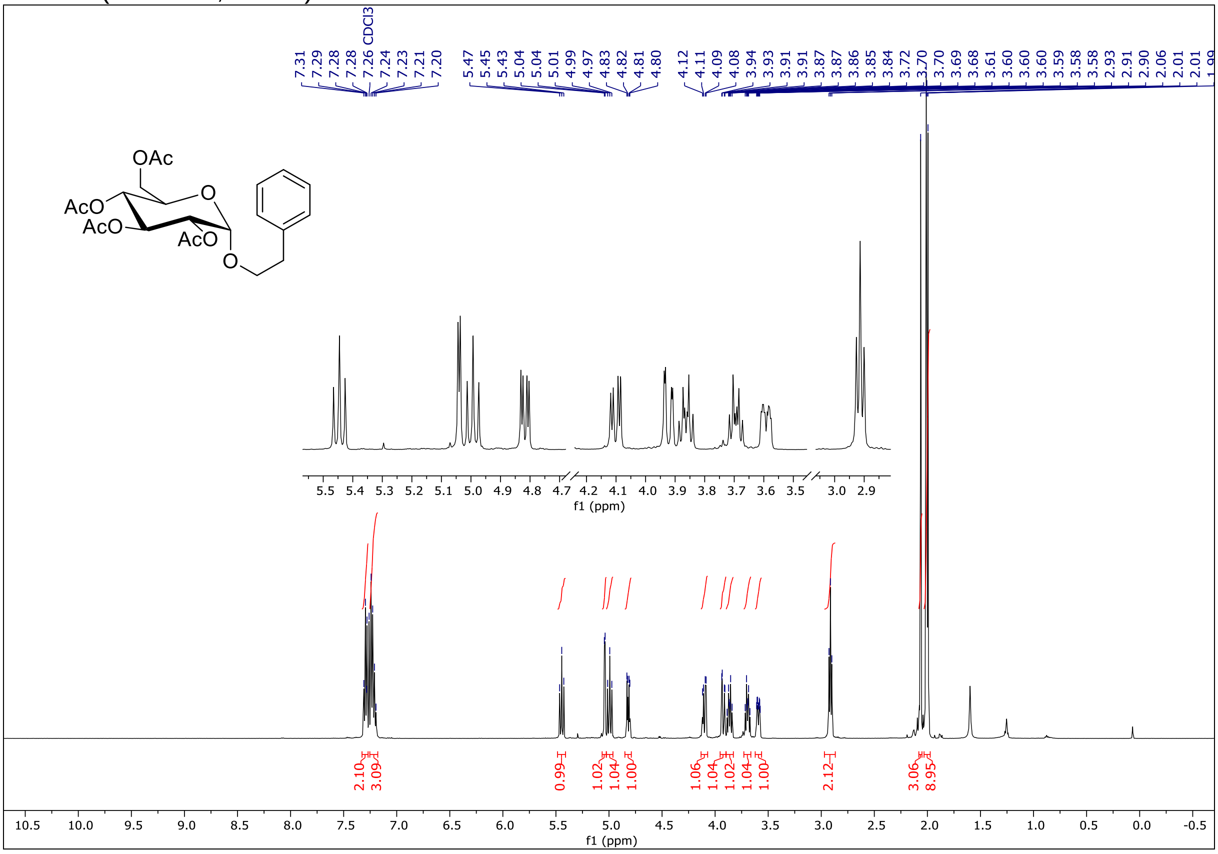

^{13}C NMR (126 MHz, $CDCl_3$)

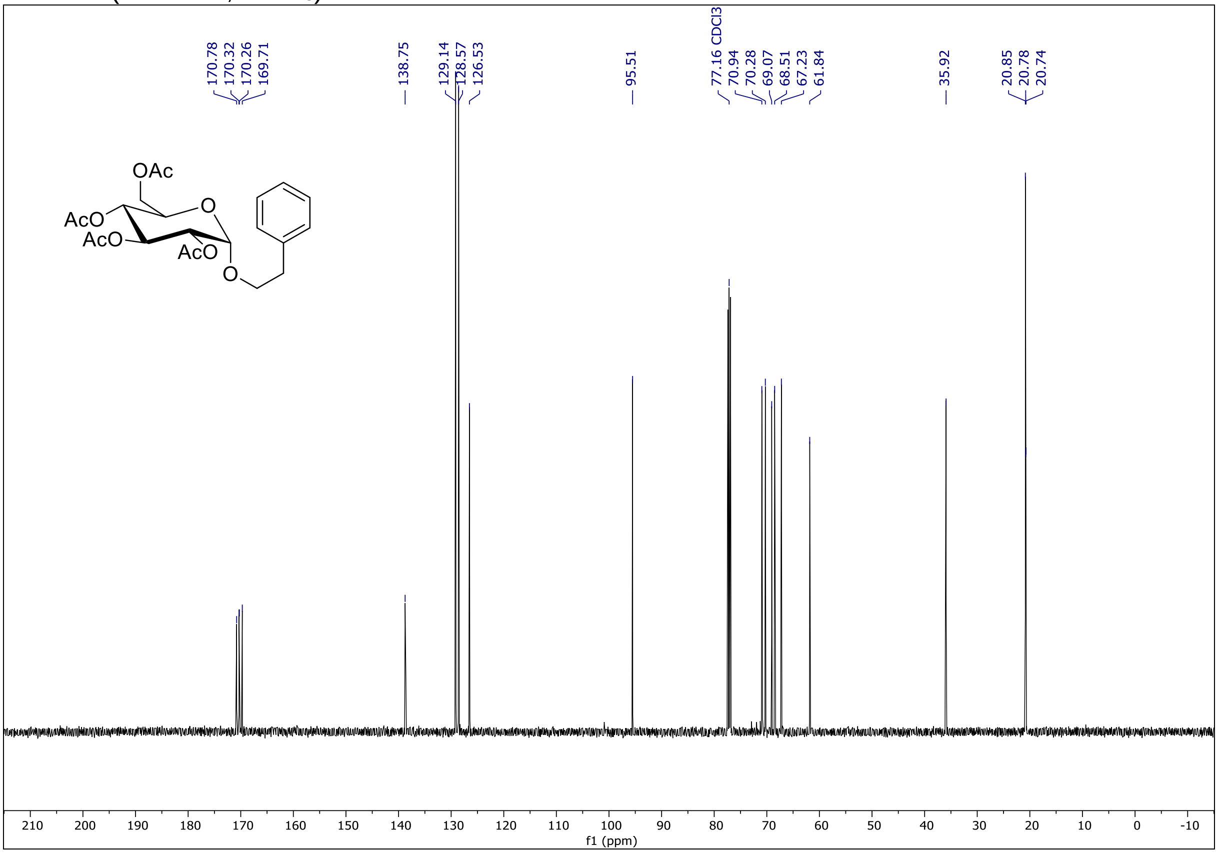

β-**14a**

^{1}H NMR (500 MHz, $CDCl_3$)

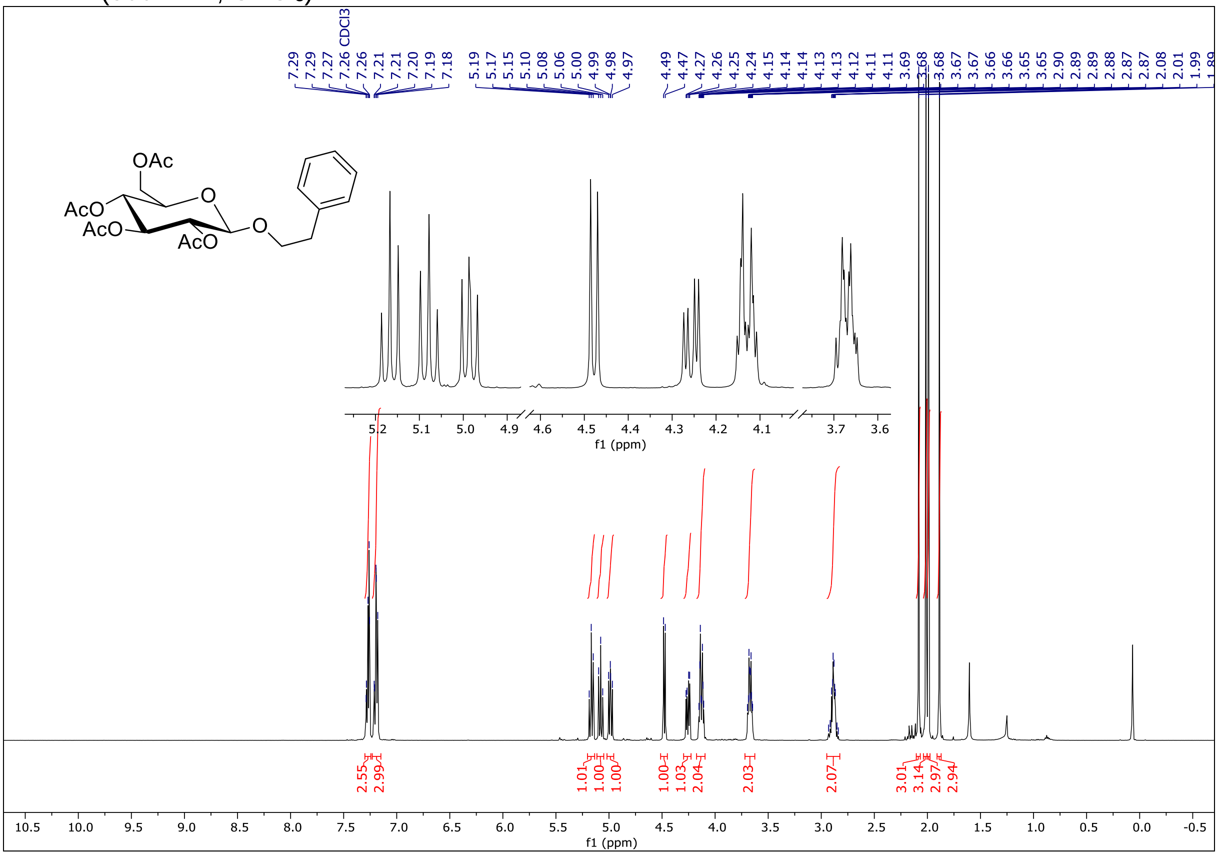

^{13}C NMR (126 MHz, $CDCl_3$)

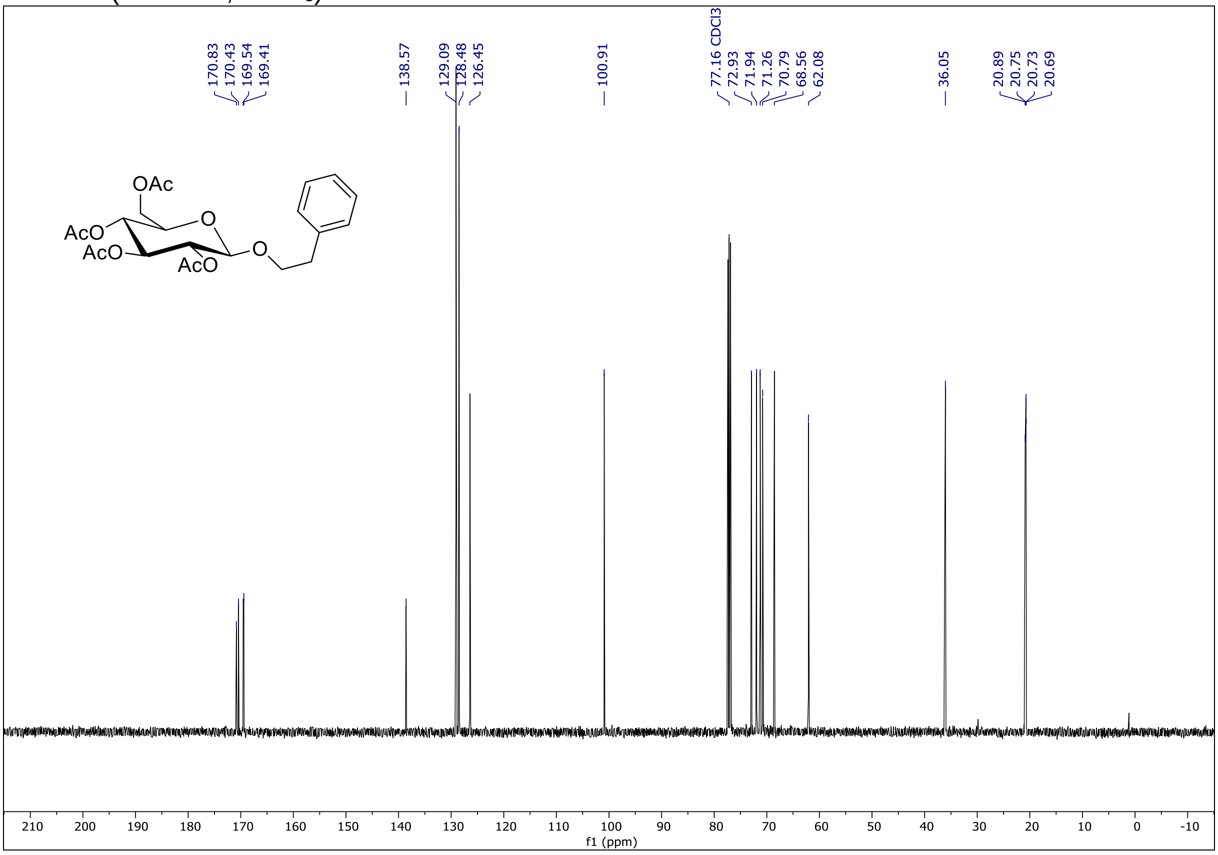

α-**14b**

^{1}H NMR (500 MHz, $CDCl_3$)

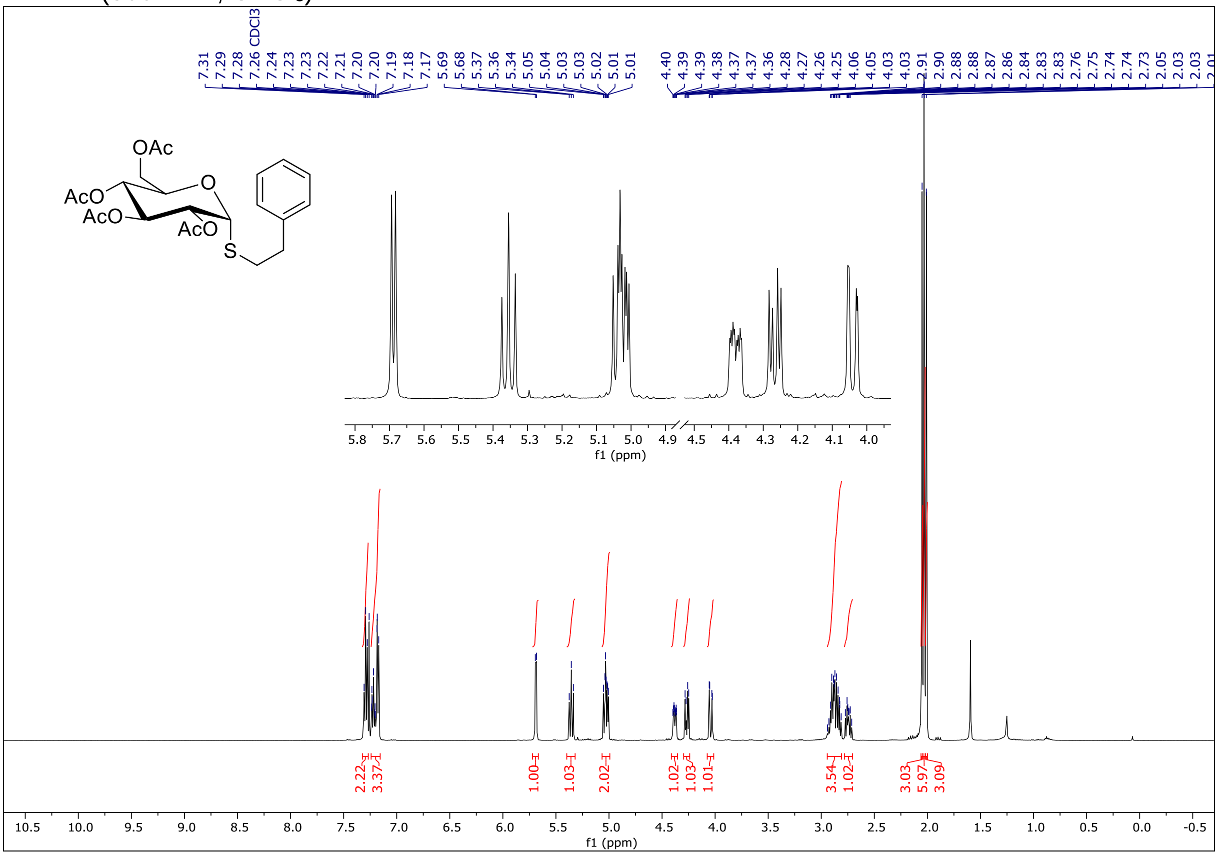

^{13}C NMR (126 MHz, $CDCl_3$)

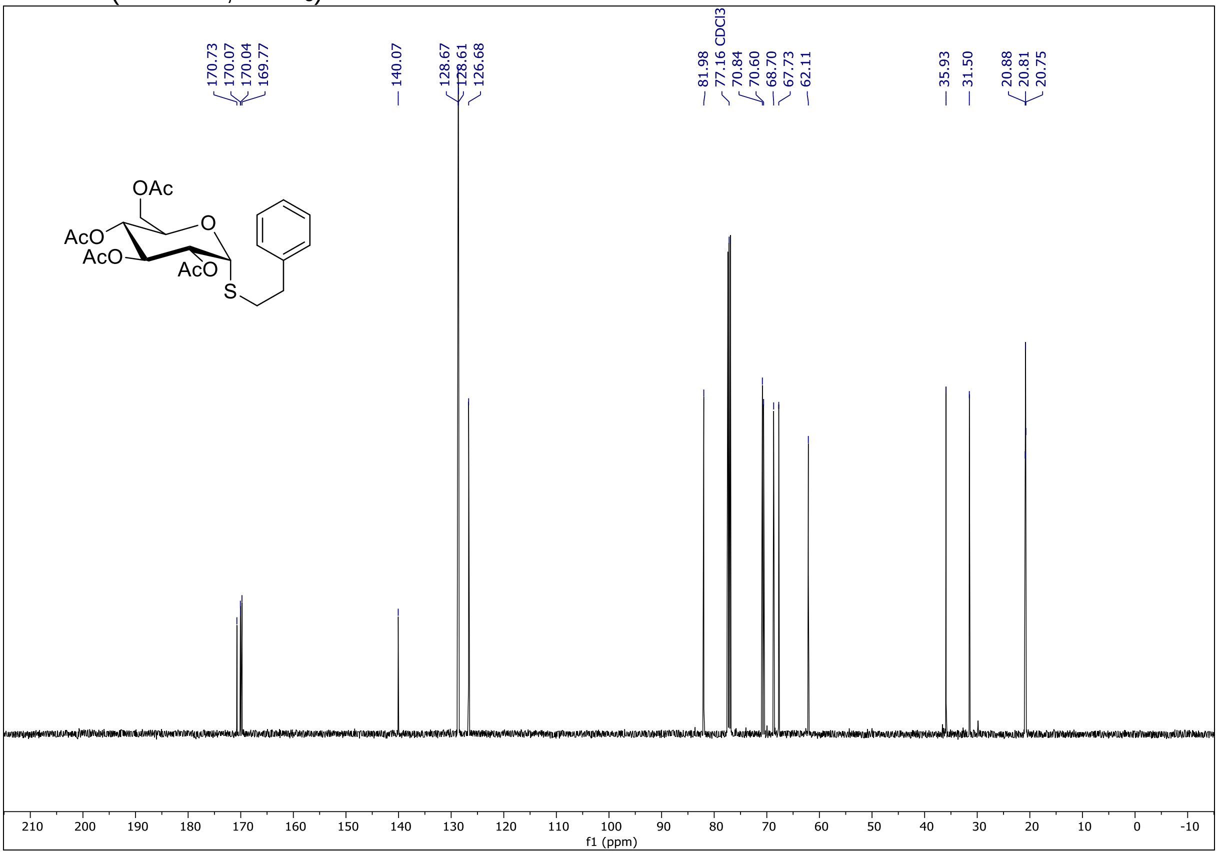

β-**14b**

^{1}H NMR (500 MHz, $CDCl_3$)

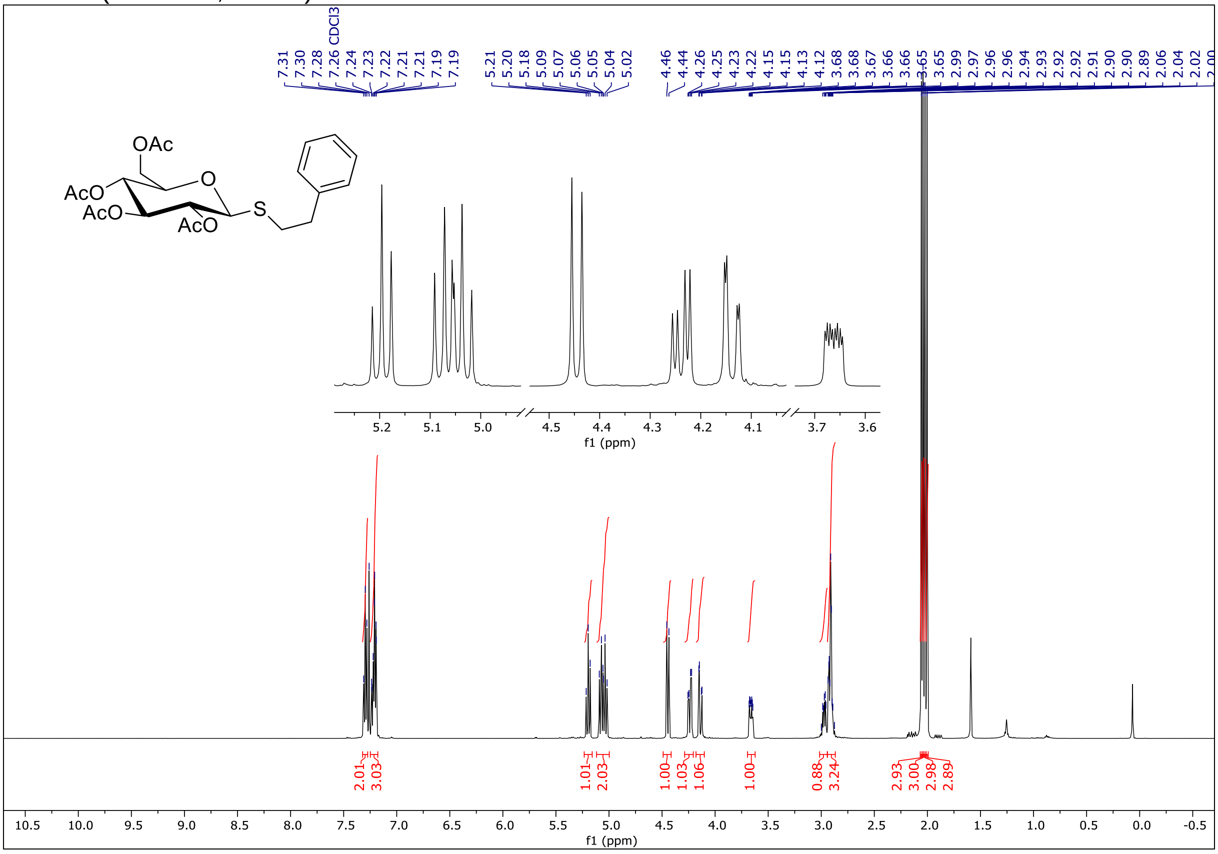

^{13}C NMR (126 MHz, $CDCl_3$)

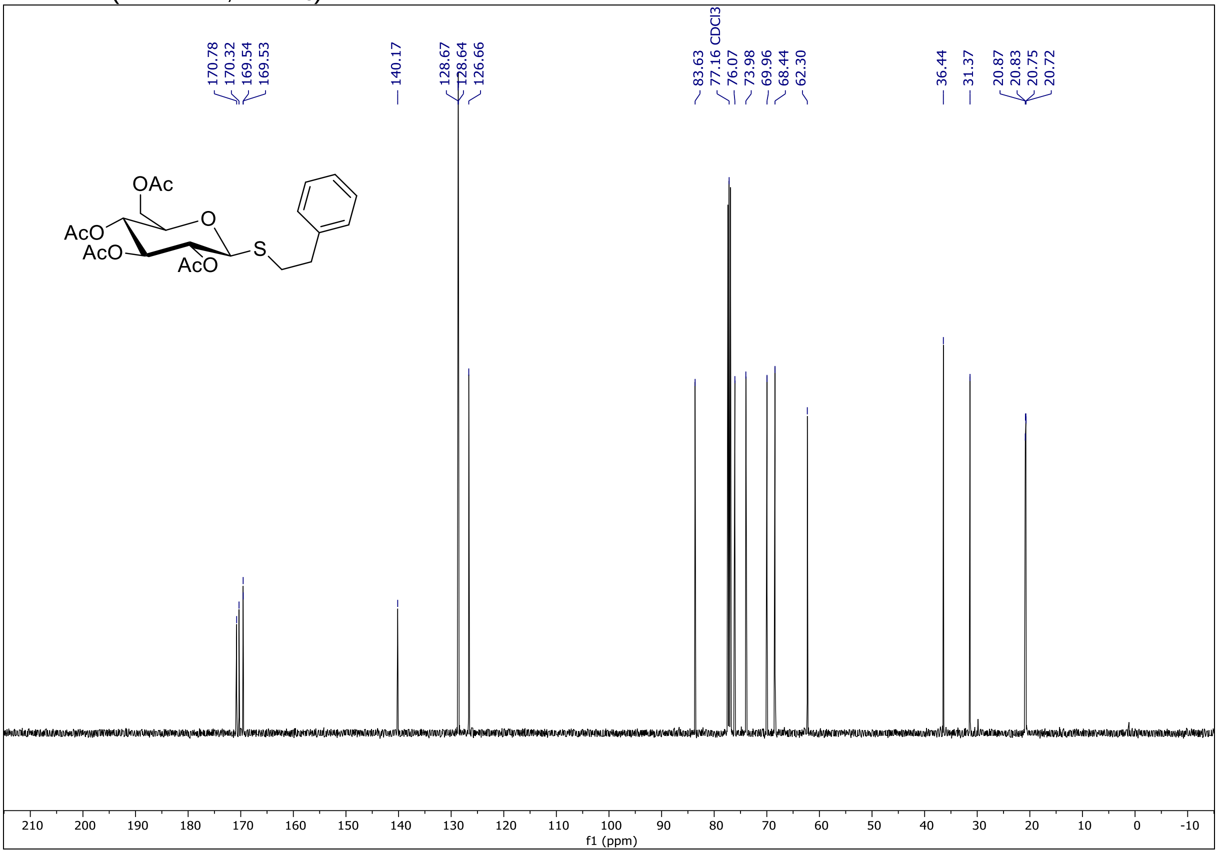

α-**17a**

^{1}H NMR (500 MHz, $CDCl_3$)

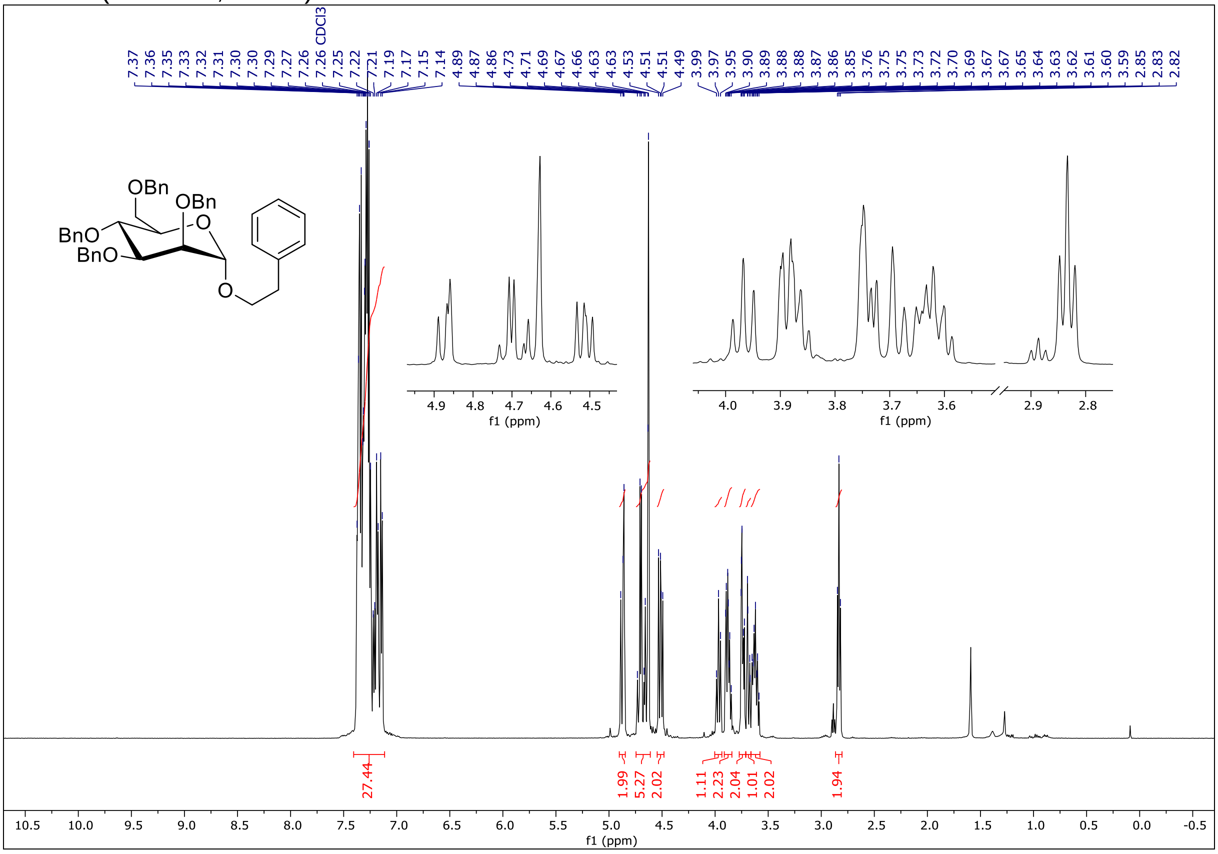

^{13}C NMR (126 MHz, $CDCl_3$)

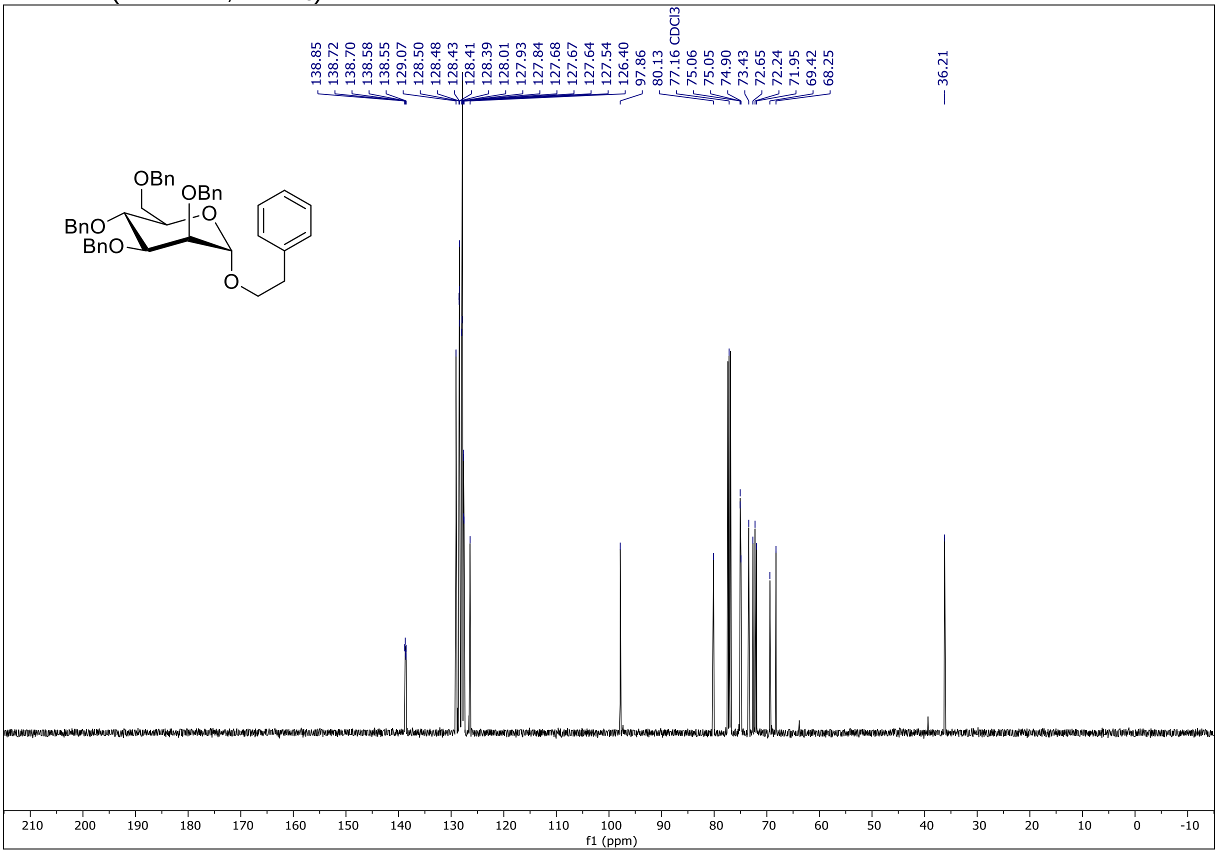

β-17a

^{1}H NMR (500 MHz, $CDCl_3$)

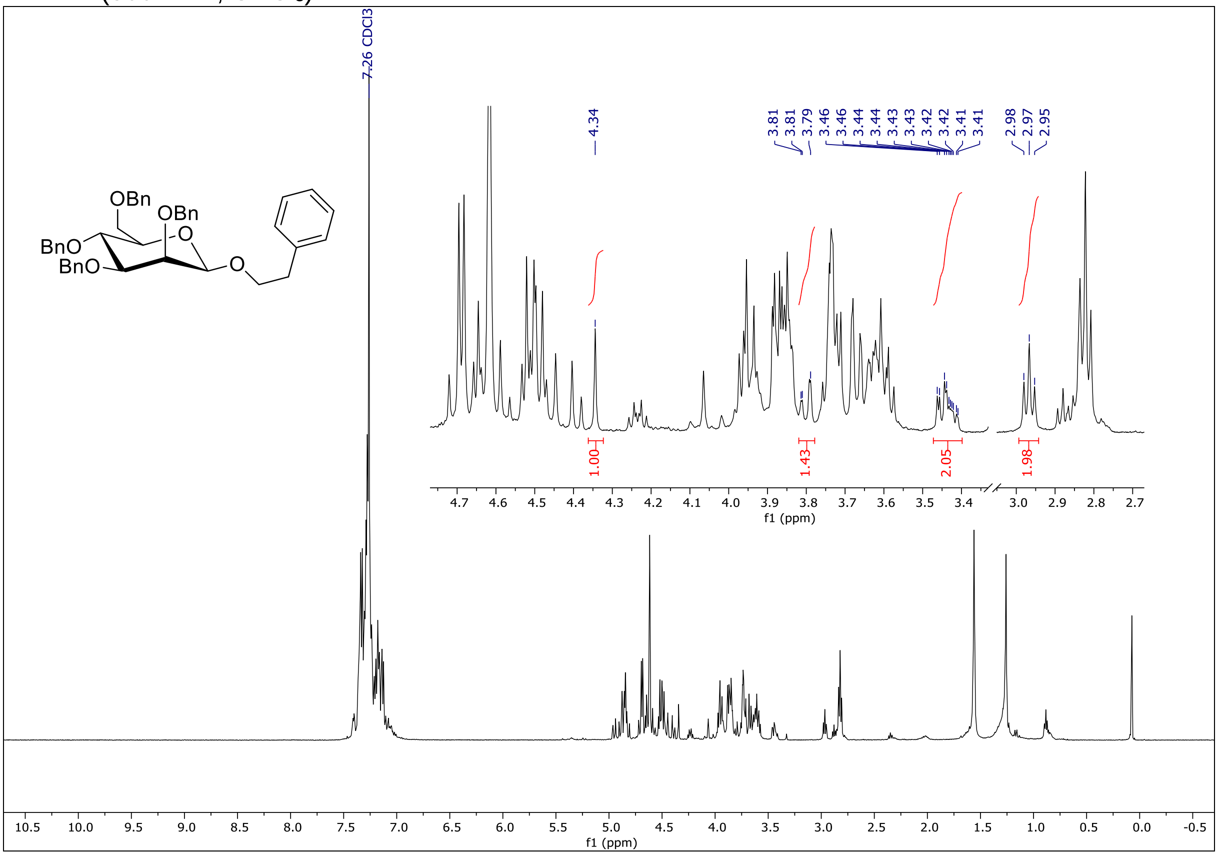

^{13}C NMR (126 MHz, $CDCl_3$)

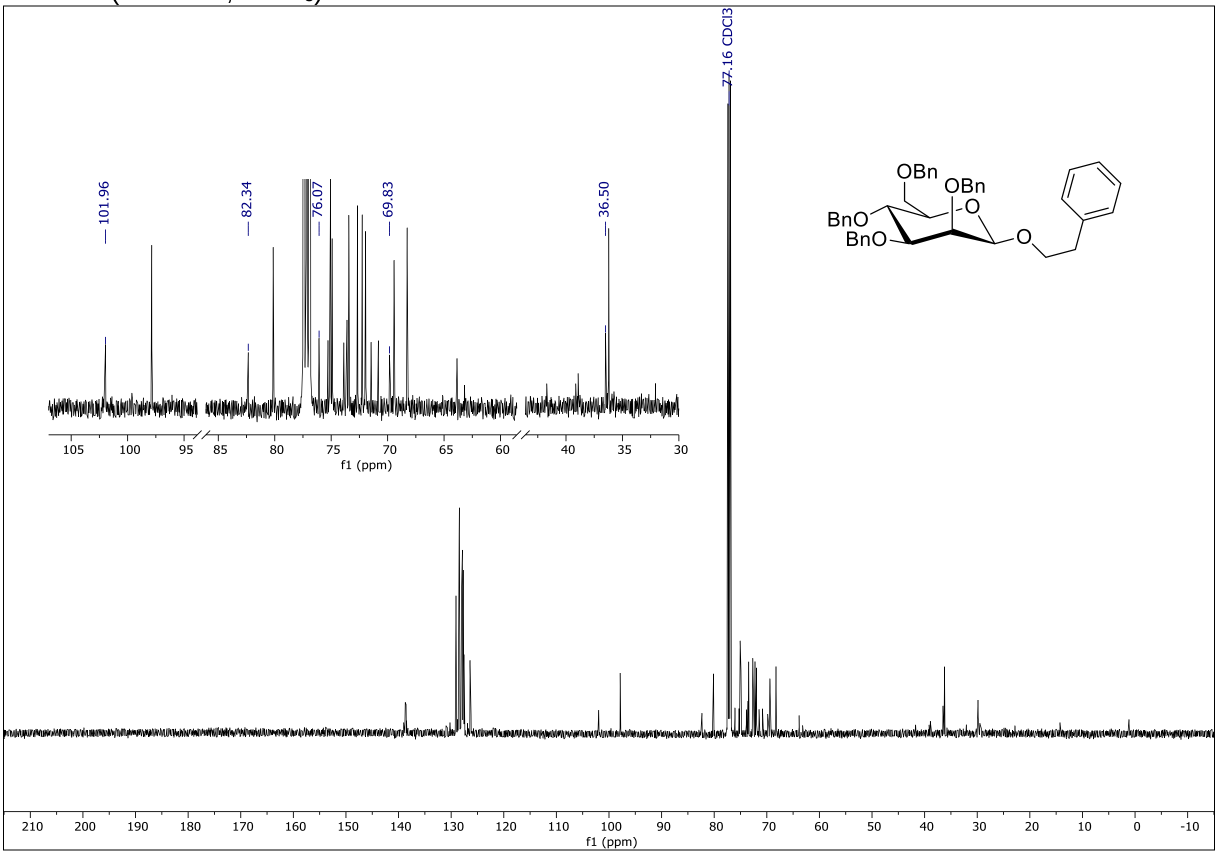

α-17b

^{1}H NMR (500 MHz, CDCl$_3$)

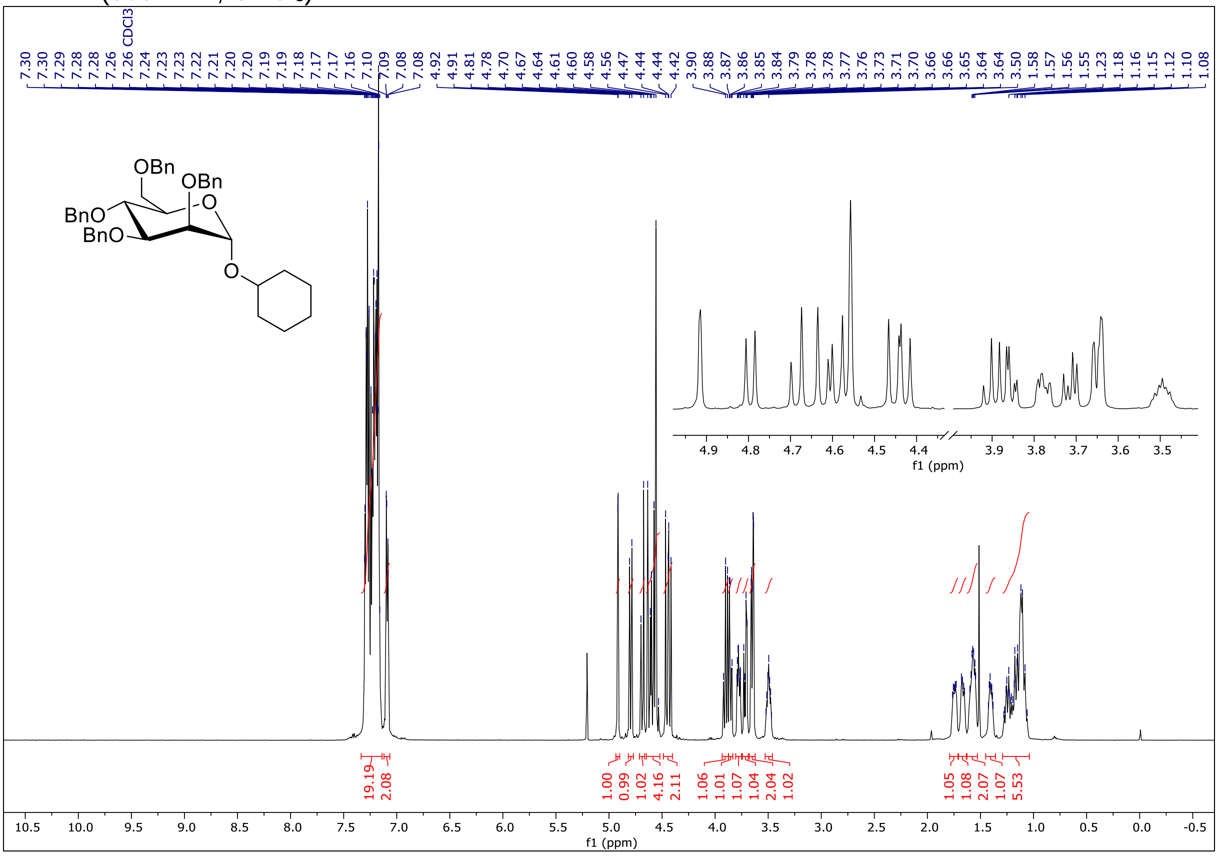

^{13}C NMR (126 MHz, CDCl$_3$)

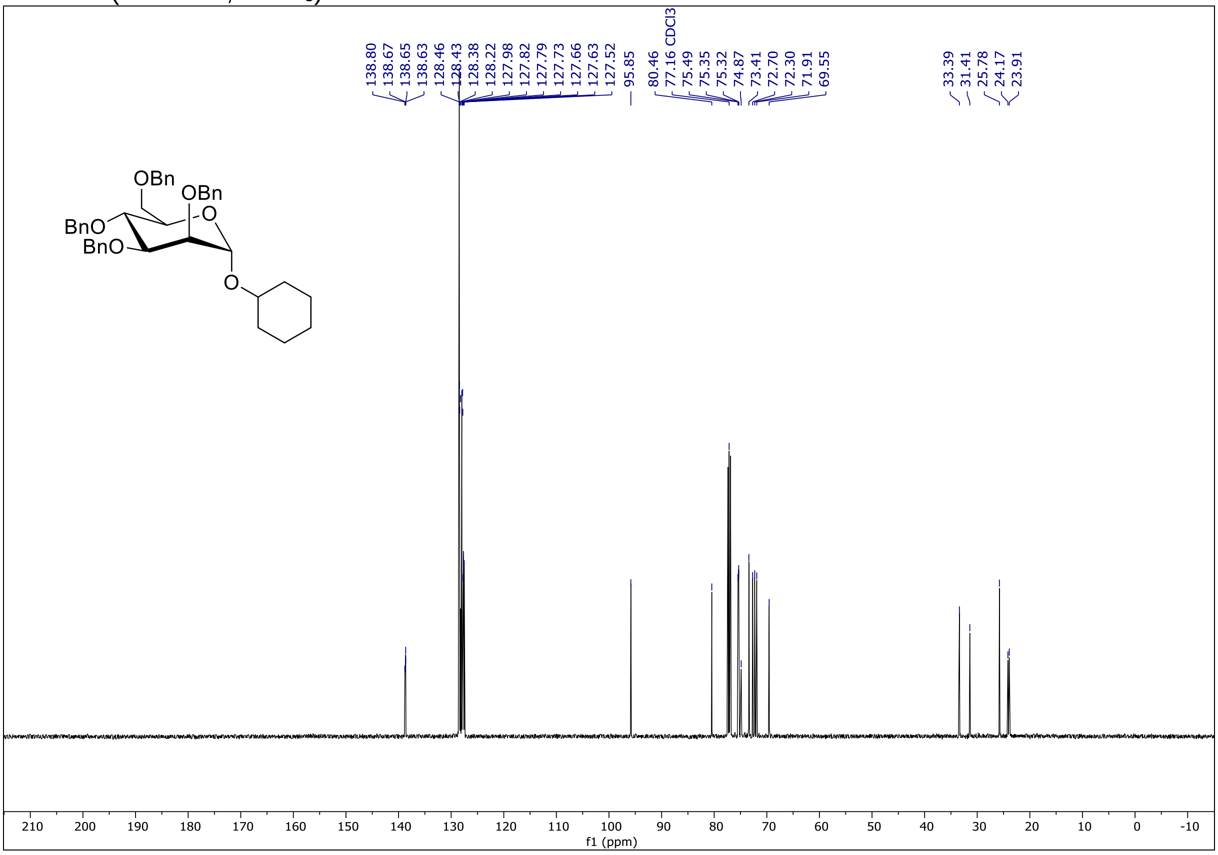

α-**17c**

^{1}H NMR (500 MHz, $CDCl_3$)

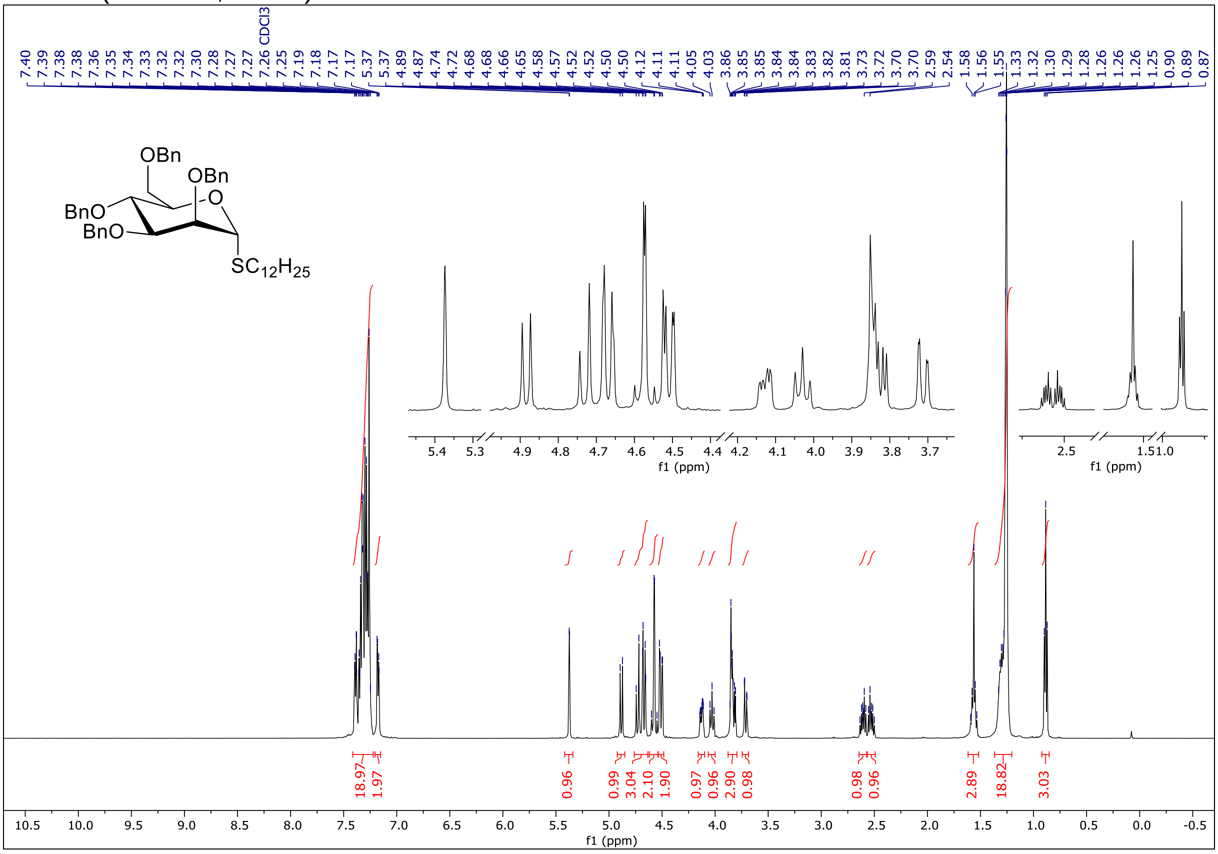

^{13}C NMR (126 MHz, $CDCl_3$)

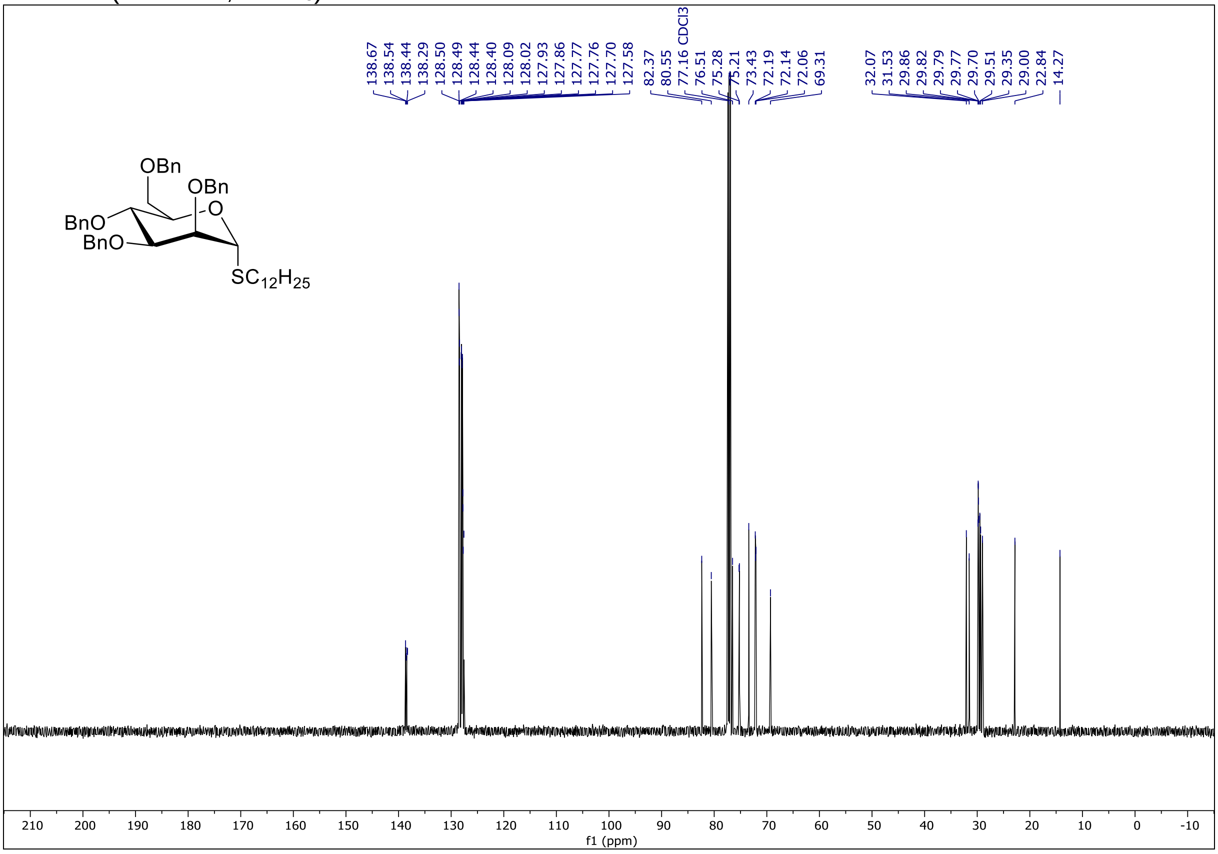

β-17c

^{1}H NMR (500 MHz, $CDCl_3$)

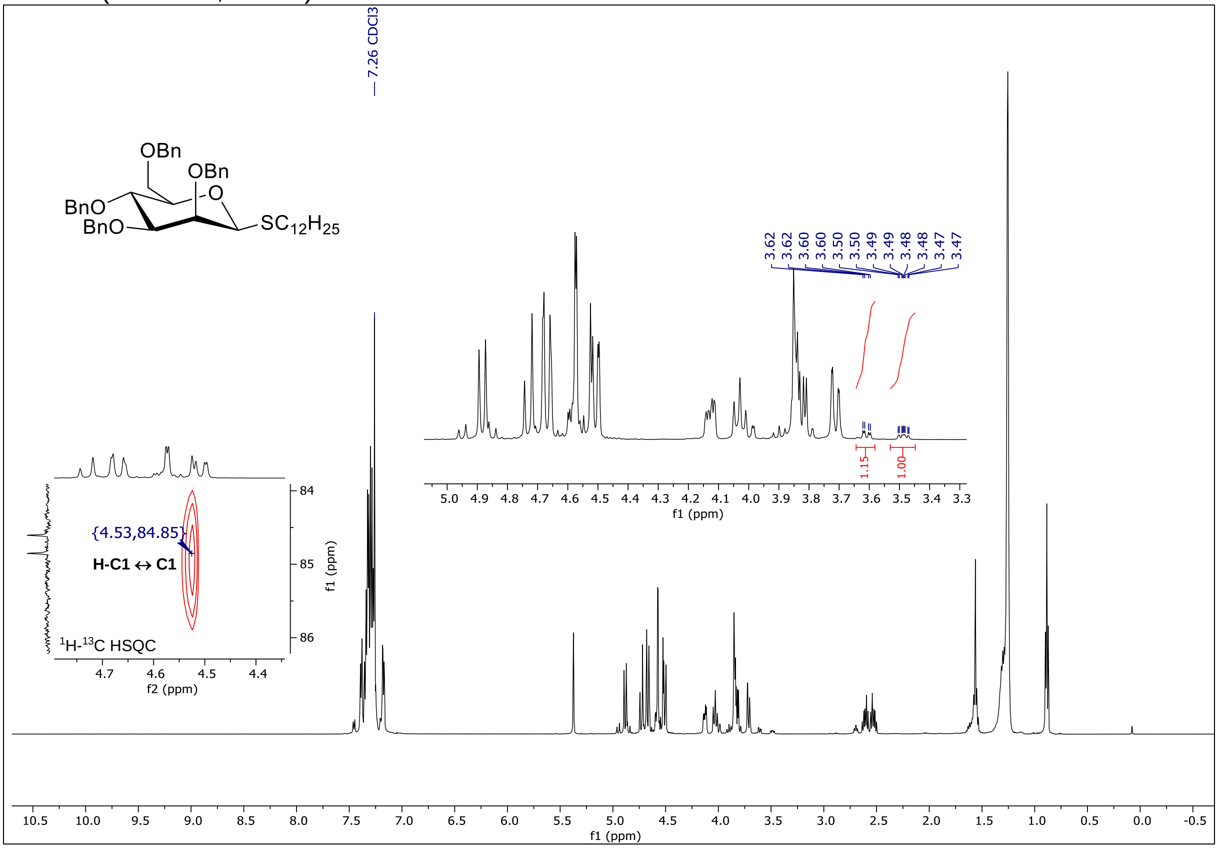

^{13}C NMR (126 MHz, $CDCl_3$)

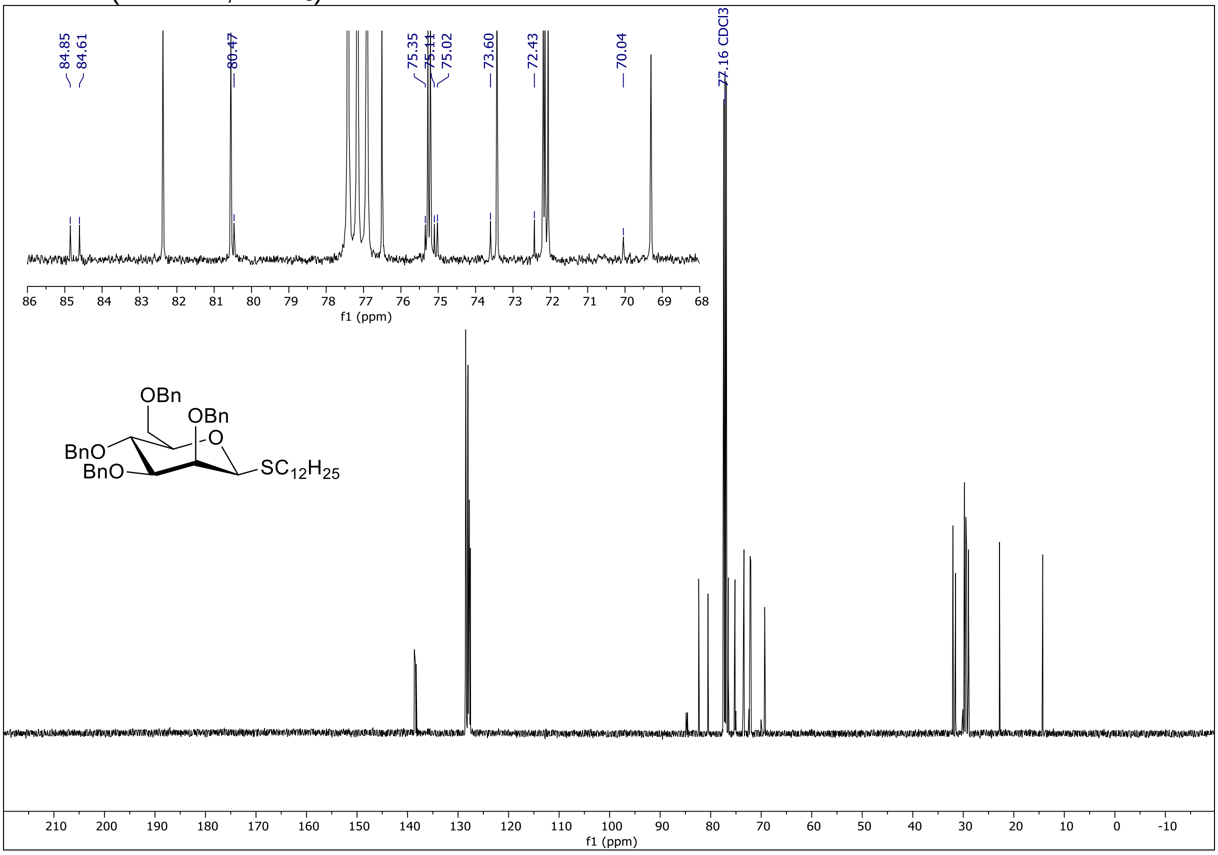

α-**17d**

^{1}H NMR (500 MHz, CDCl$_3$)

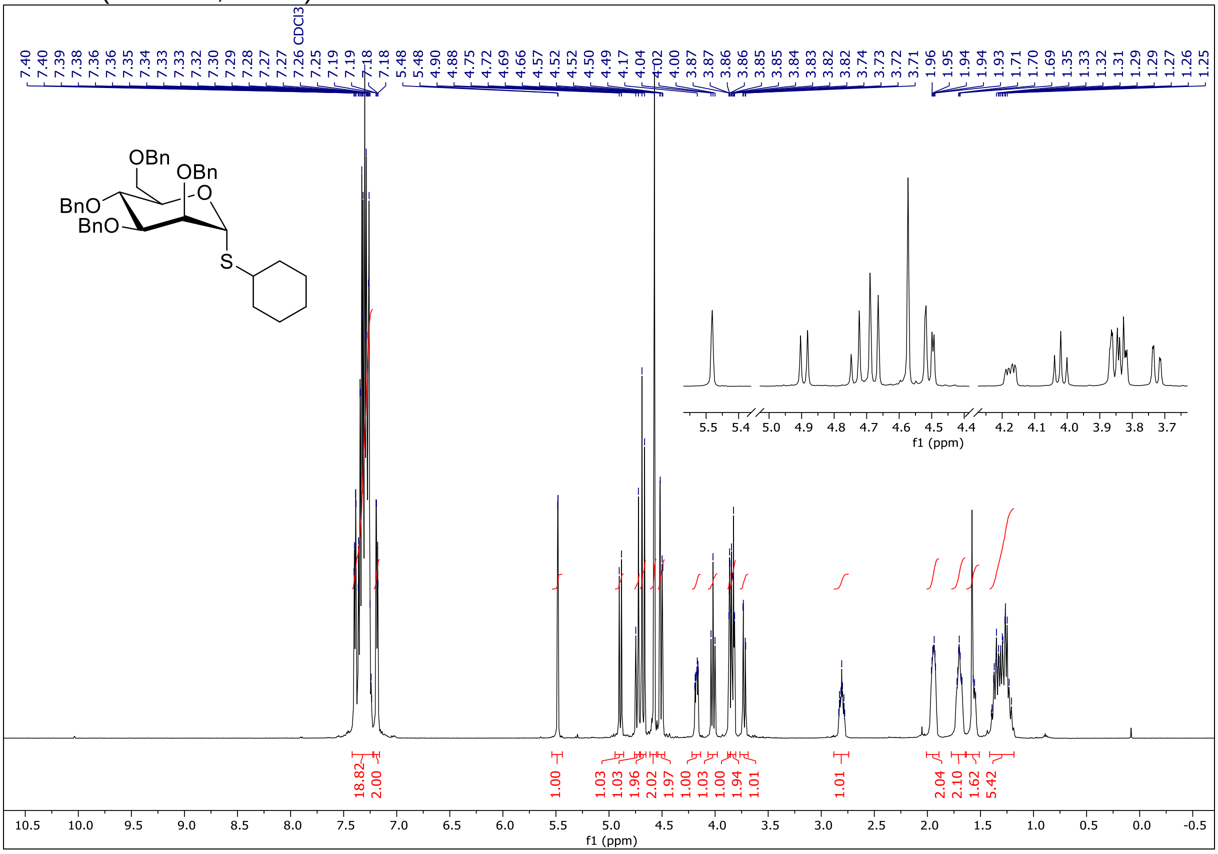

^{13}C NMR (126 MHz, CDCl$_3$)

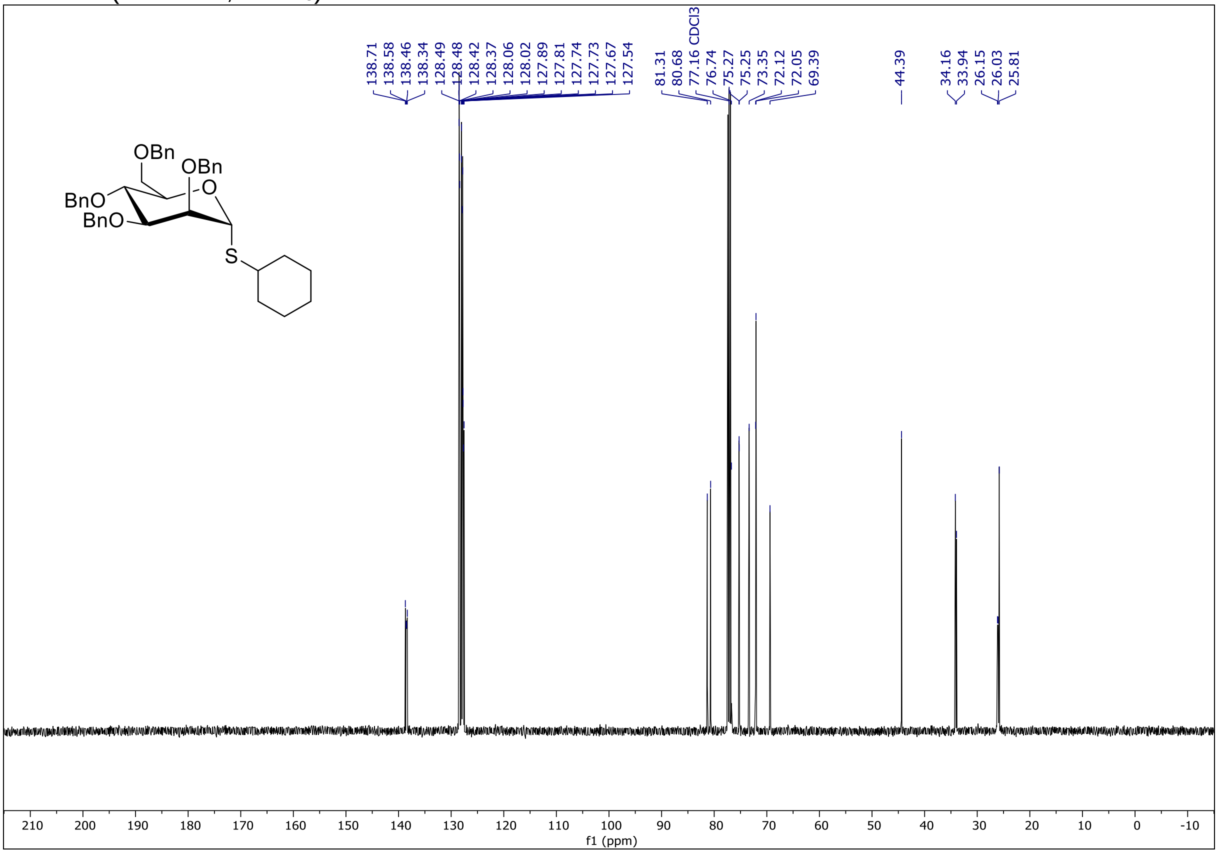

β-17d

^{1}H NMR (500 MHz, CDCl$_3$)

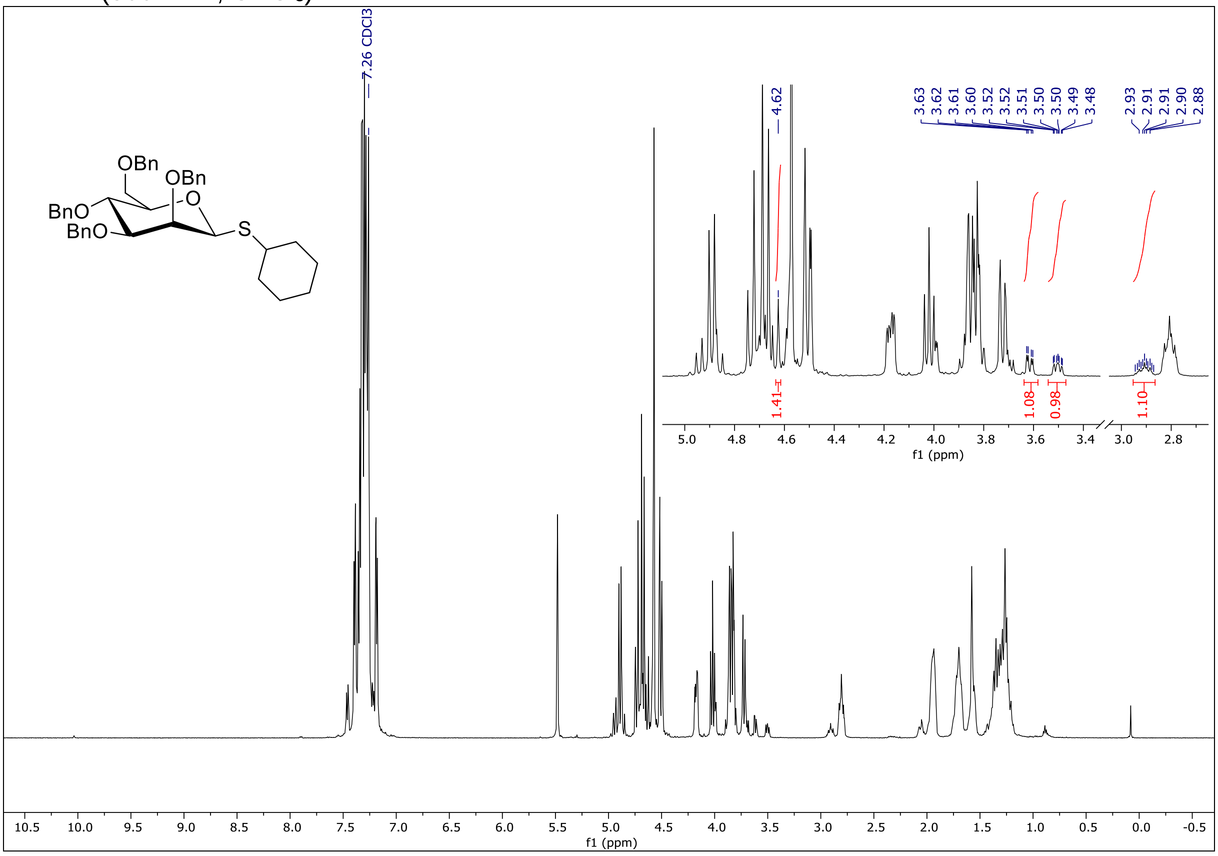

^{13}C NMR (126 MHz, CDCl$_3$)

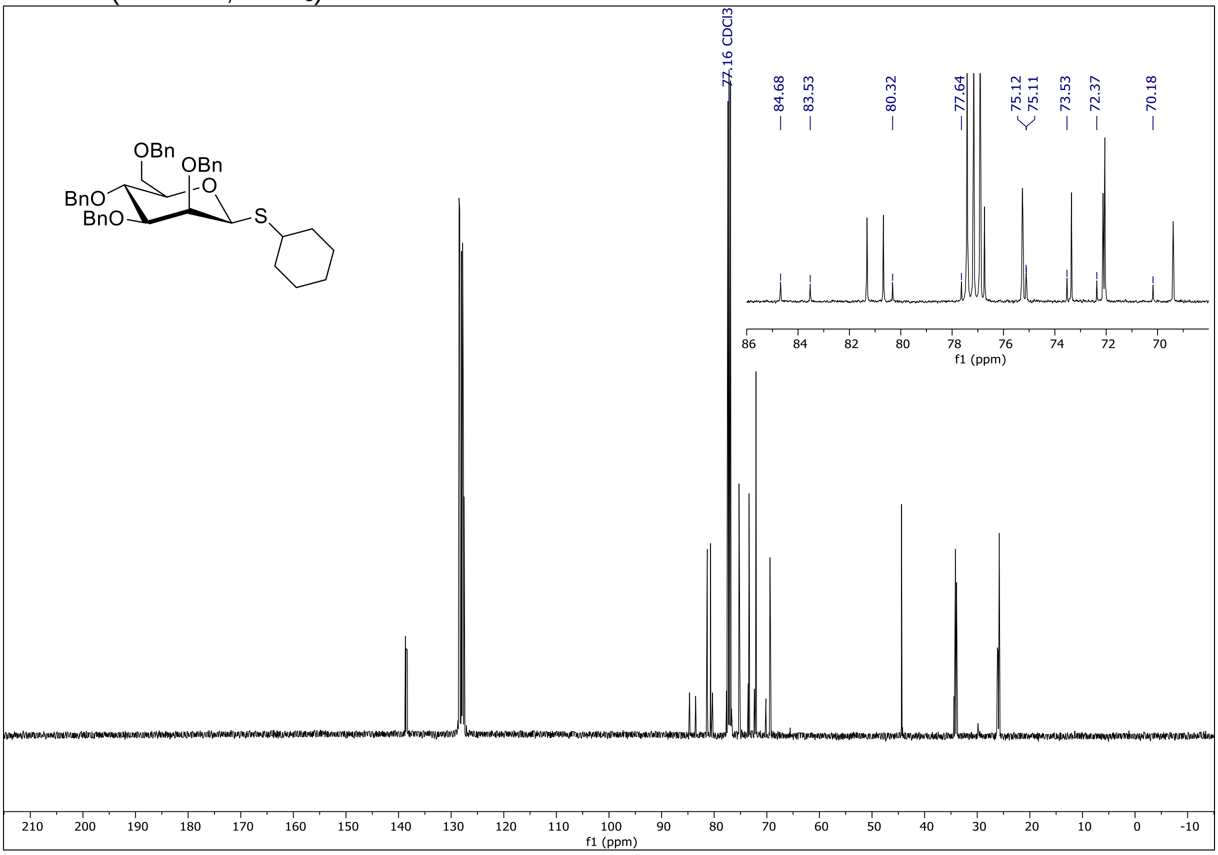

α-**17e**

^{1}H NMR (500 MHz, $CDCl_3$)

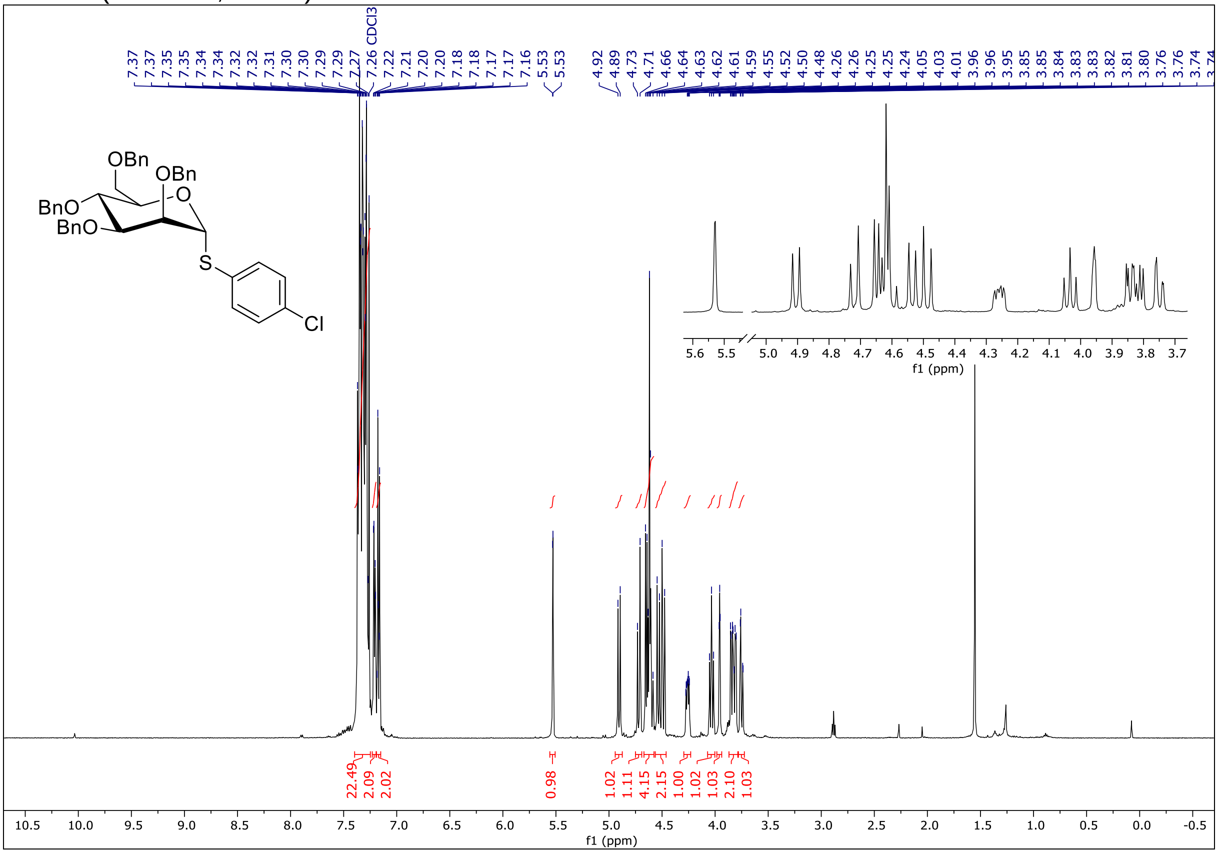

^{13}C NMR (126 MHz, $CDCl_3$)

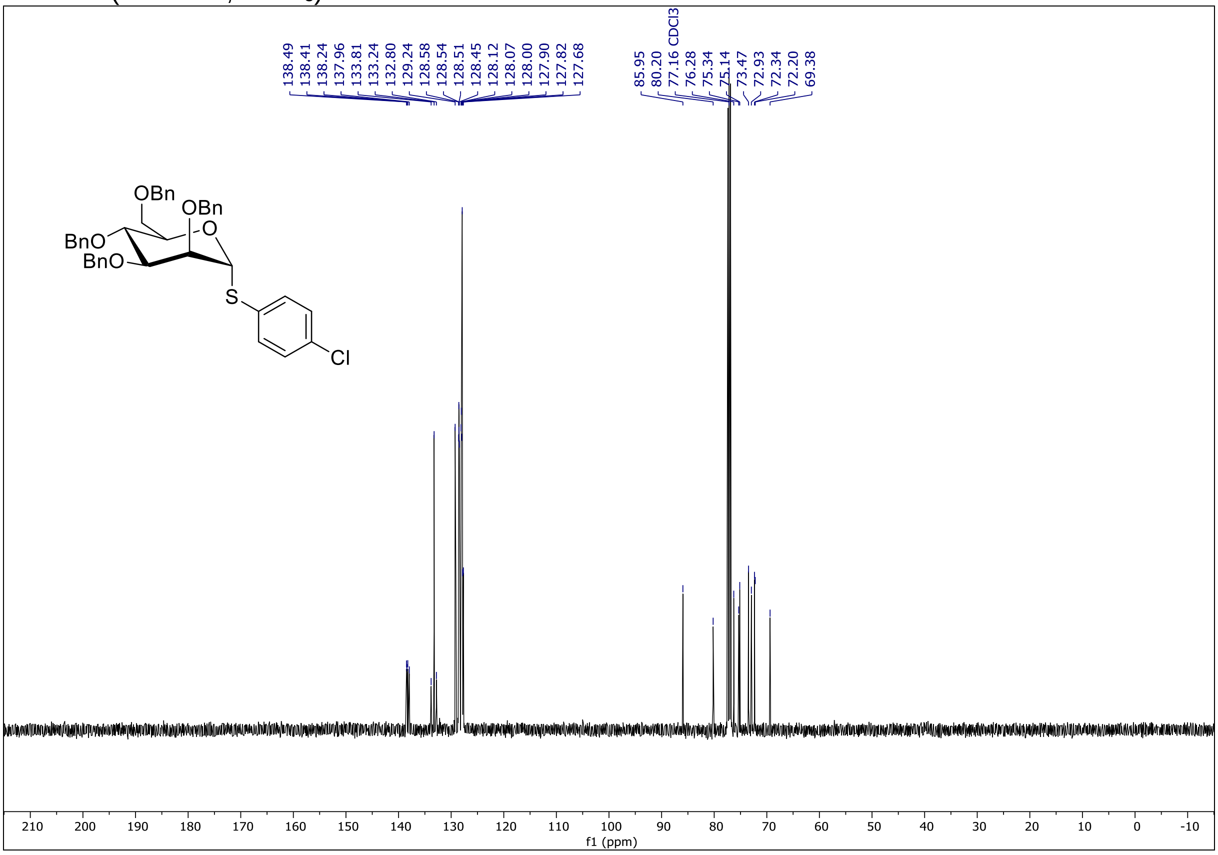

β-**17e**

^{1}H NMR (500 MHz, $CDCl_3$)

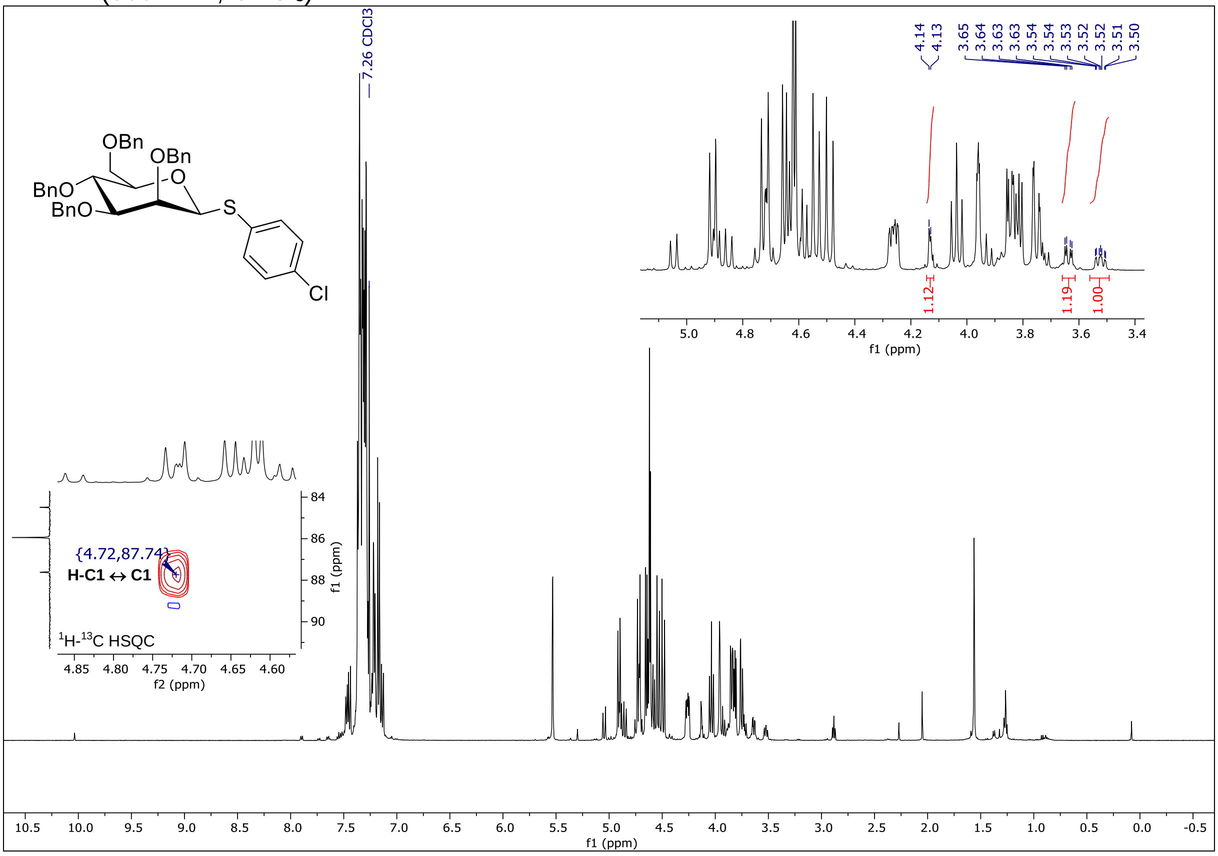

^{13}C NMR (126 MHz, $CDCl_3$)

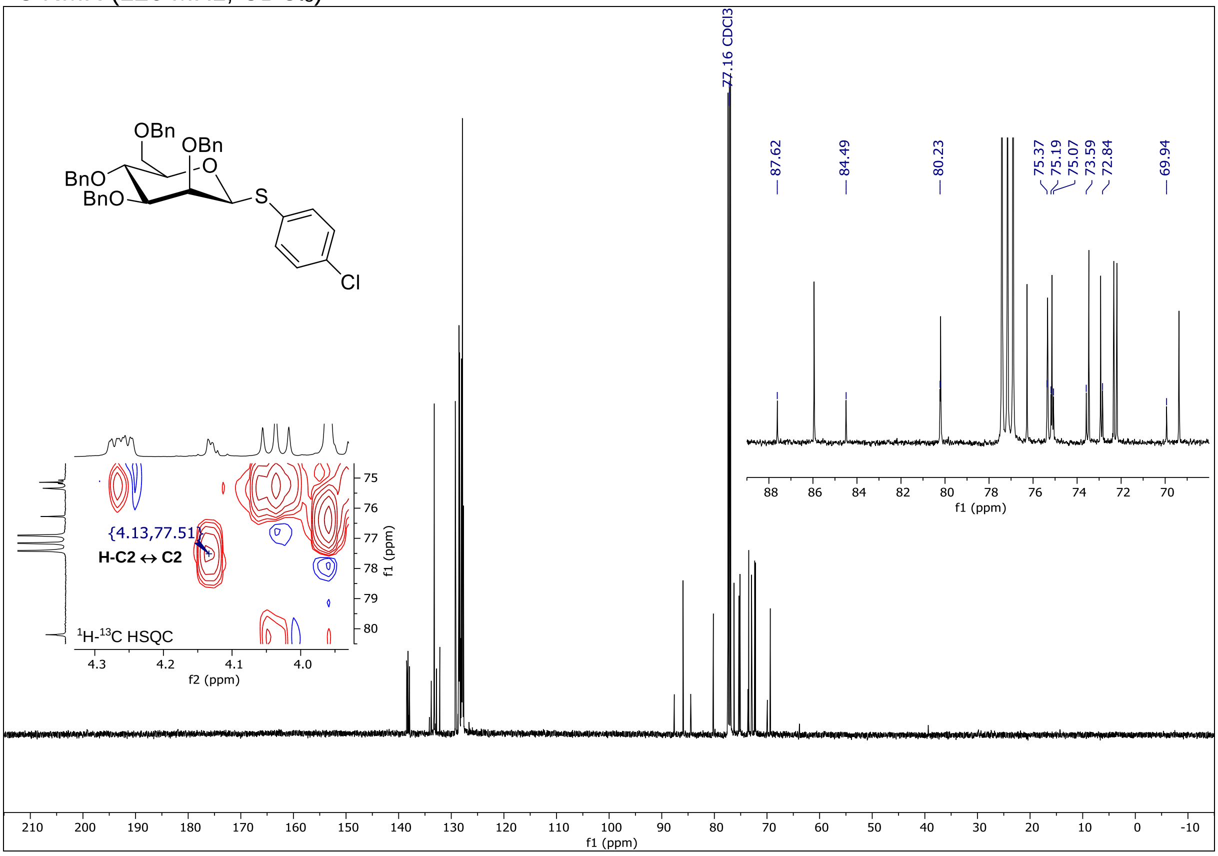

17f

^{1}H NMR (500 MHz, $CDCl_3$)

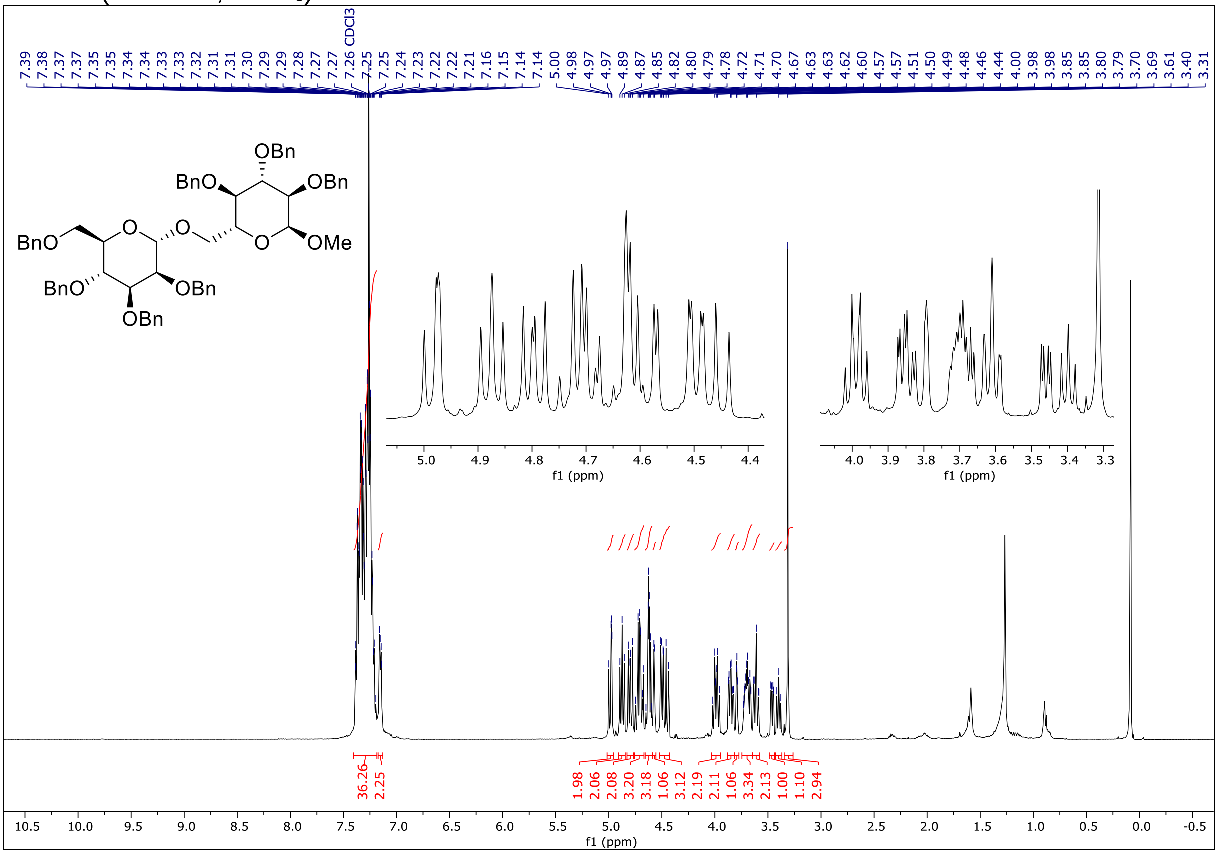

^{13}C NMR (126 MHz, $CDCl_3$)

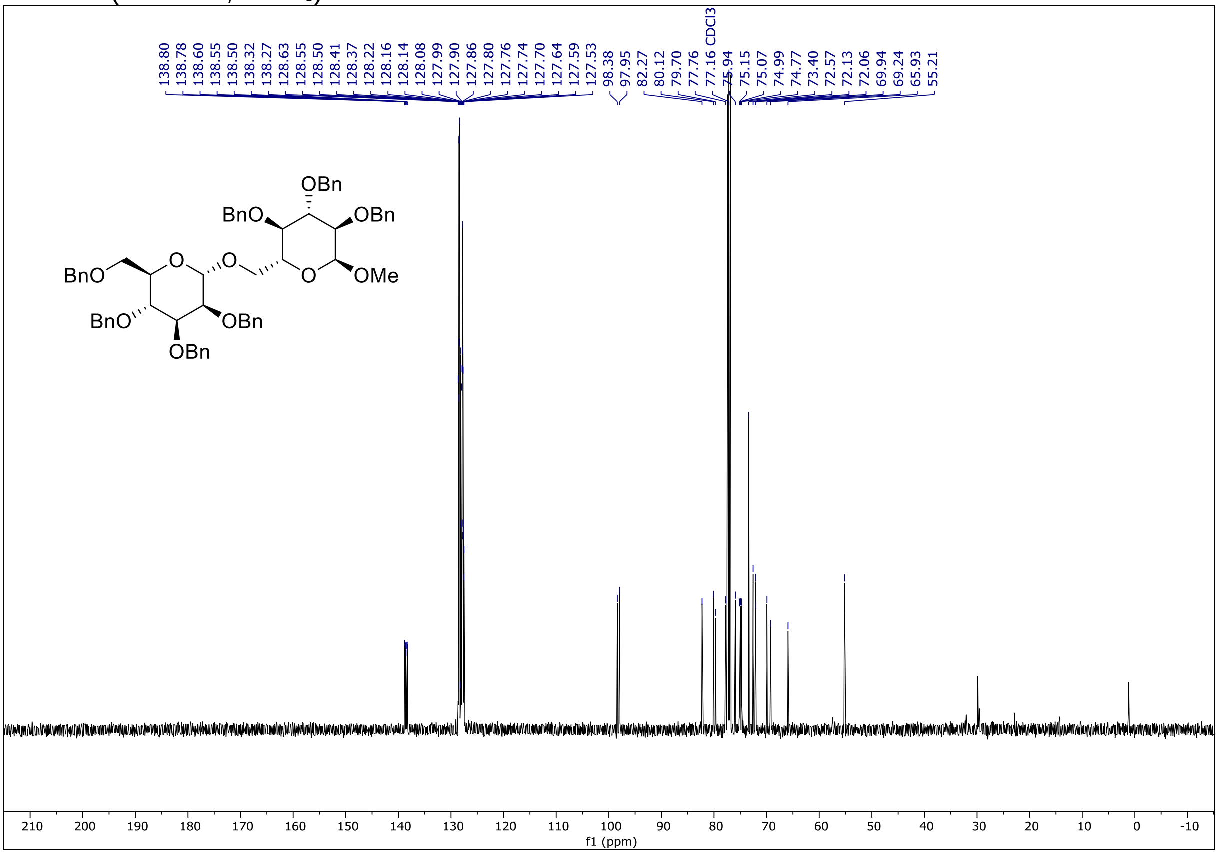

18a

^{1}H NMR (500 MHz, $CDCl_3$)

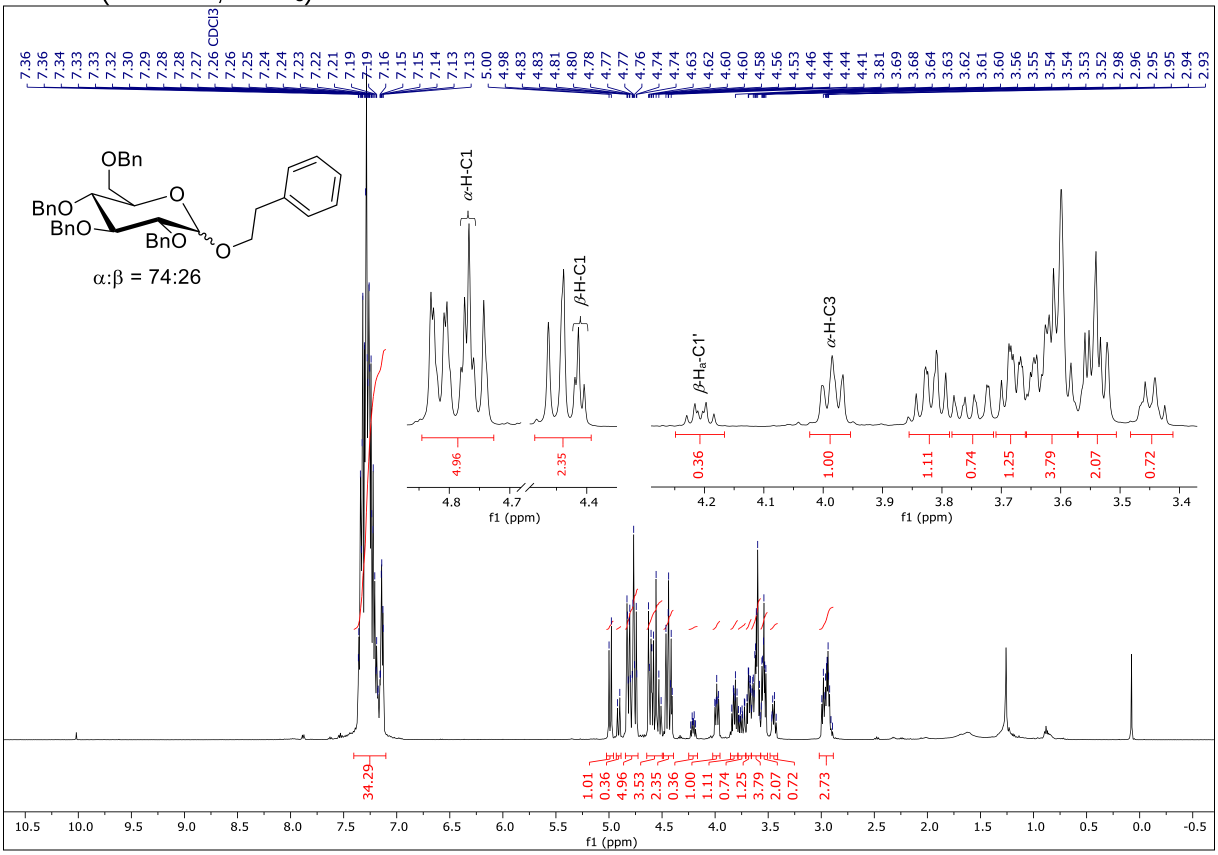

^{13}C NMR (126 MHz, $CDCl_3$)

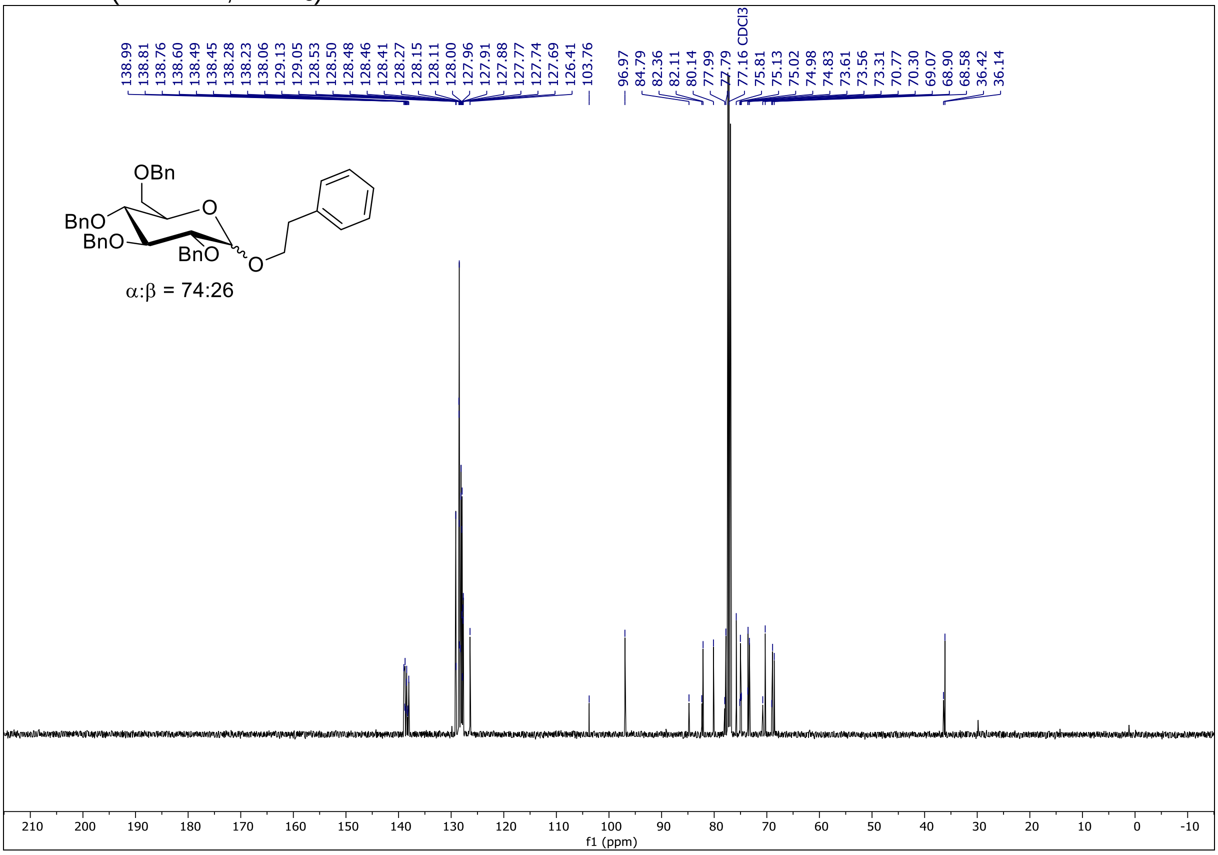

18b

^{1}H NMR (500 MHz, $CDCl_3$)

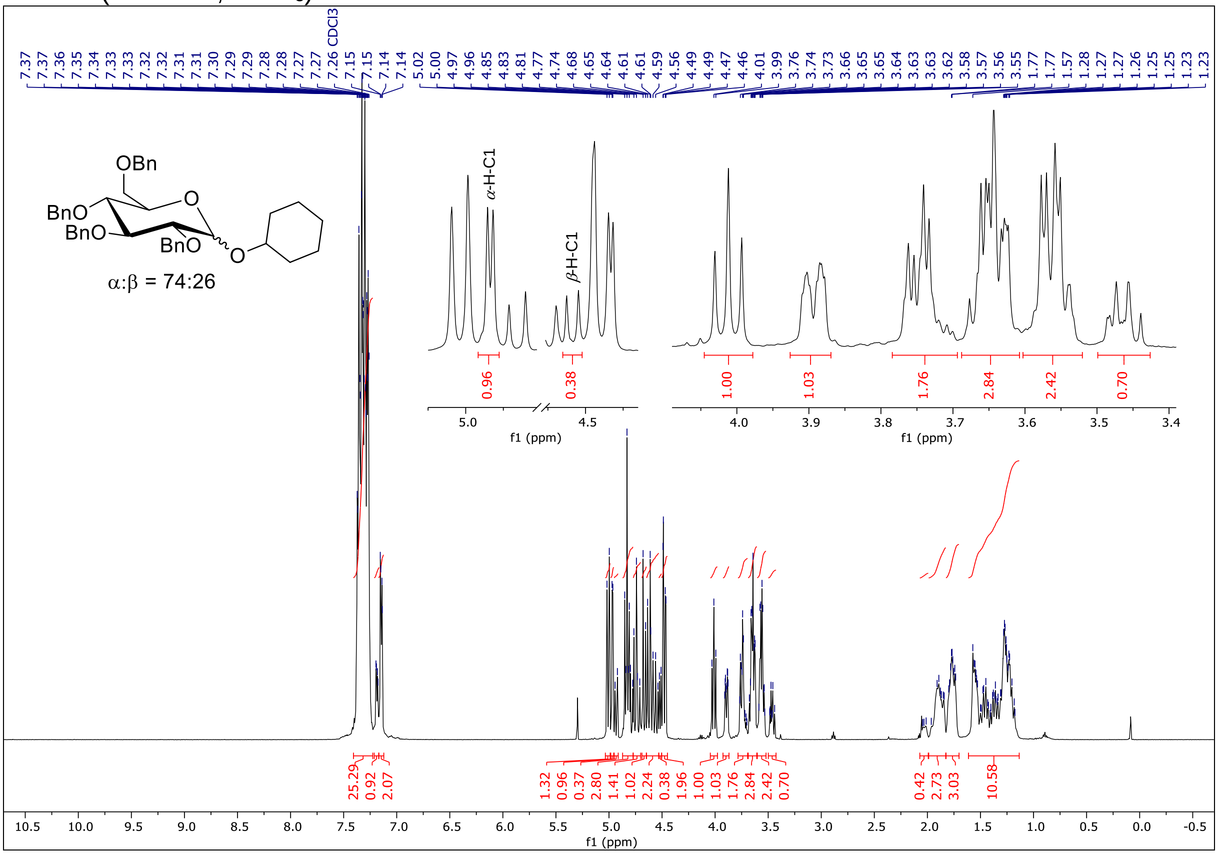

^{13}C NMR (126 MHz, $CDCl_3$)

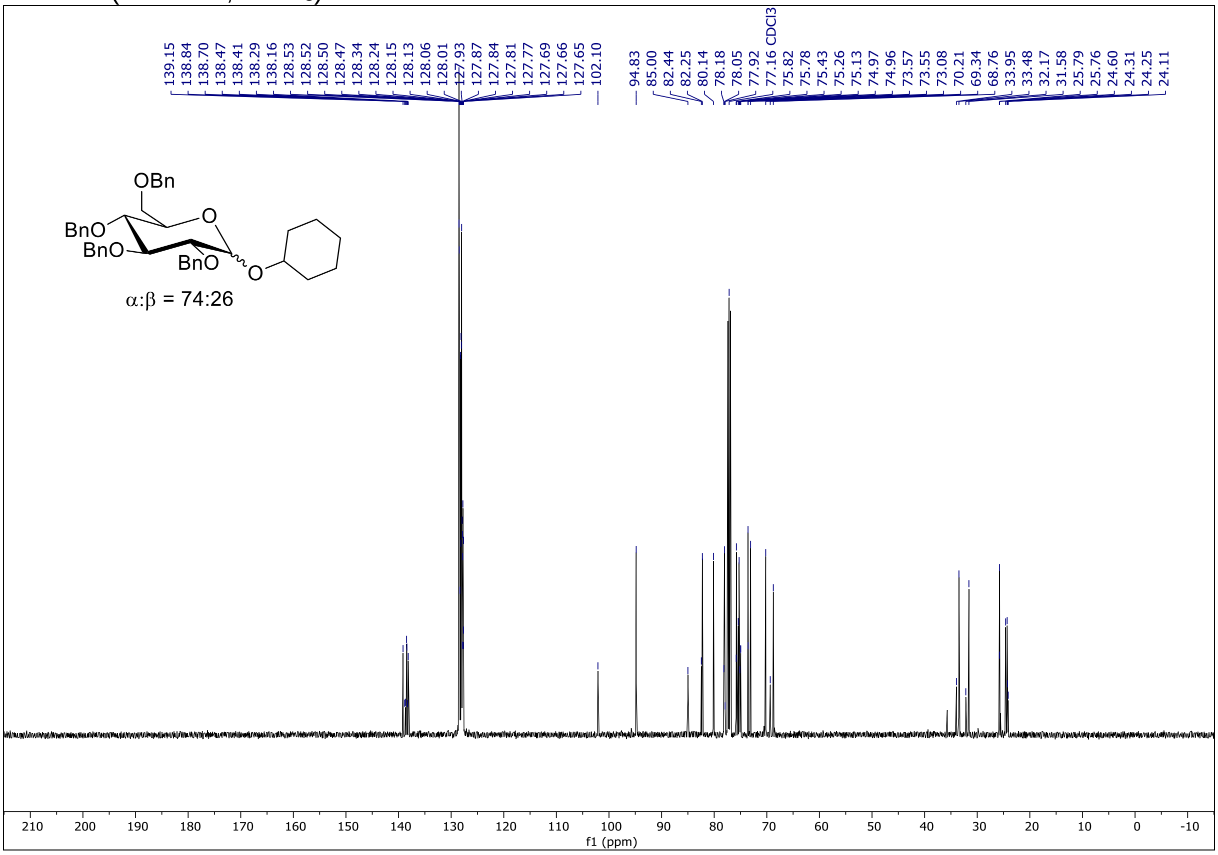

18c

^{1}H NMR (500 MHz, CDCl$_3$)

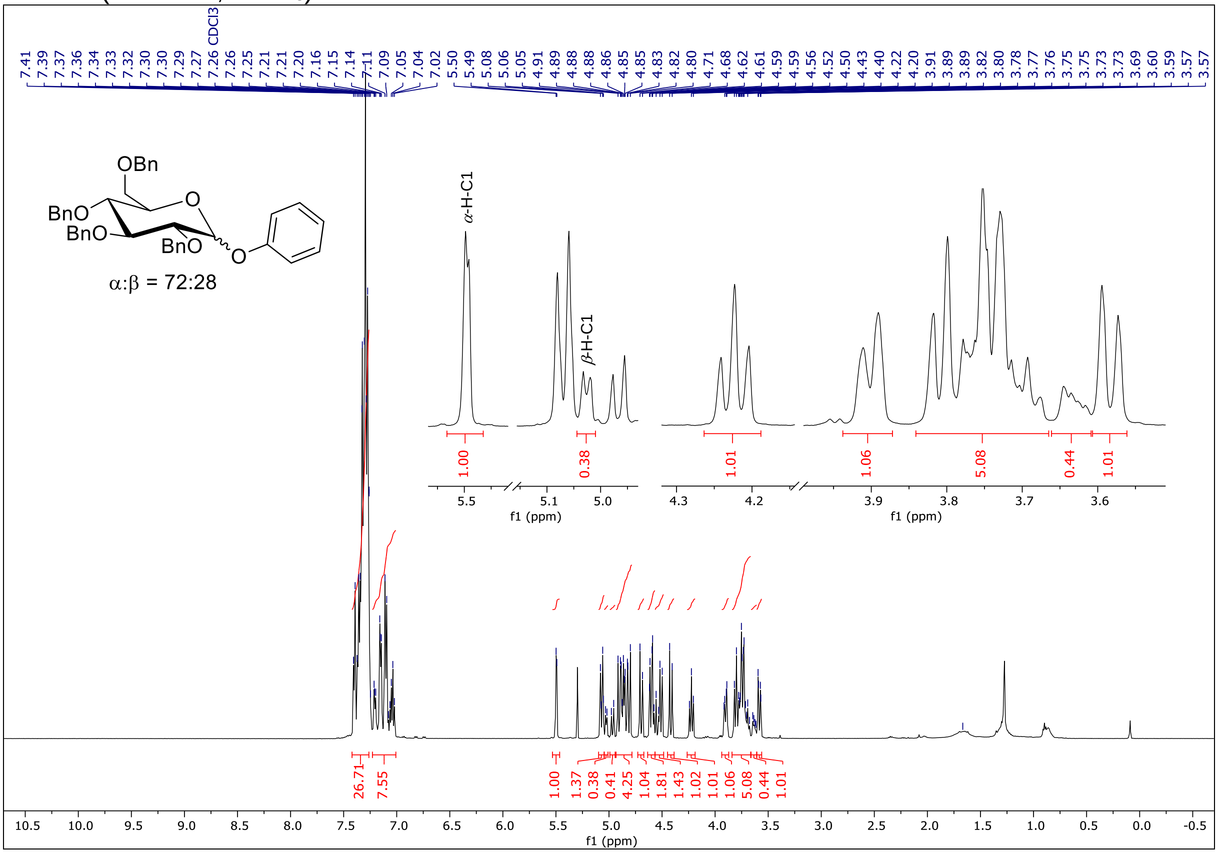

^{13}C NMR (126 MHz, CDCl$_3$)

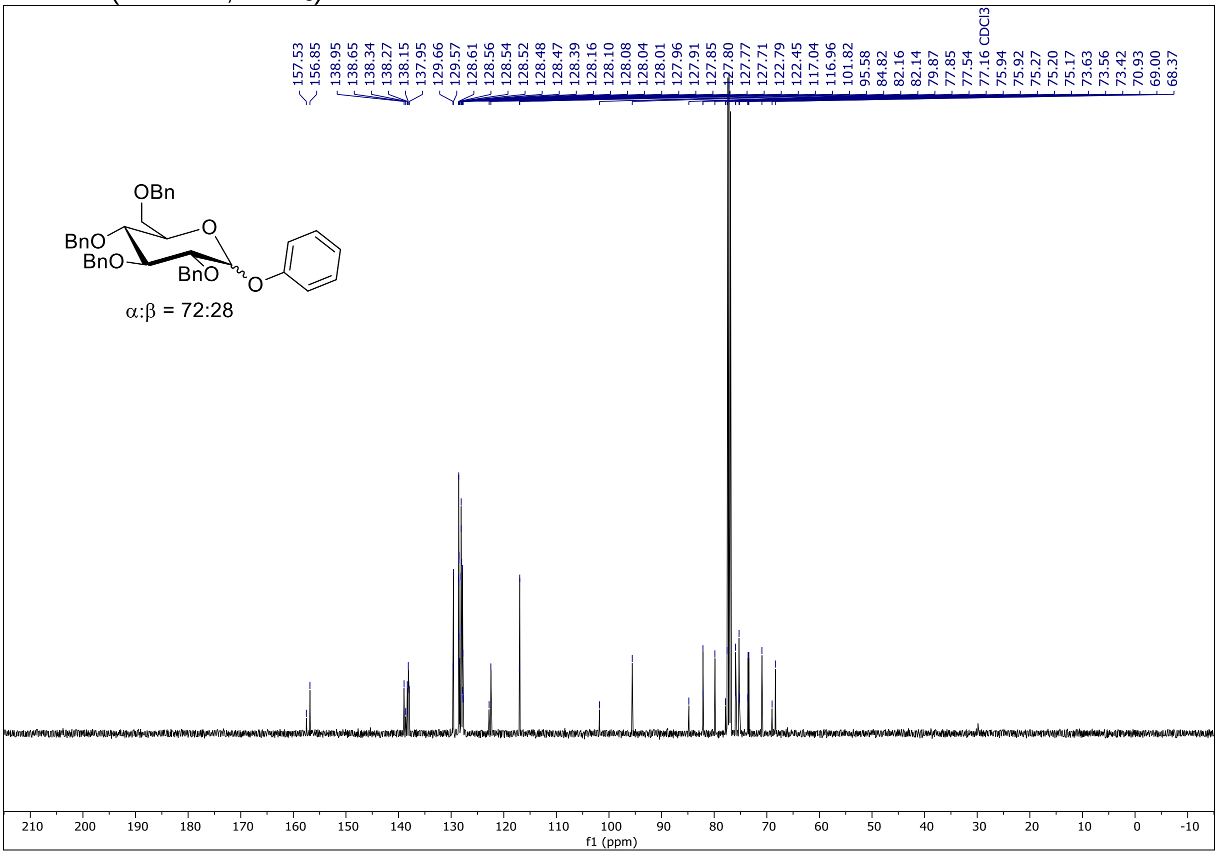

18d

^{1}H NMR (500 MHz, $CDCl_3$)

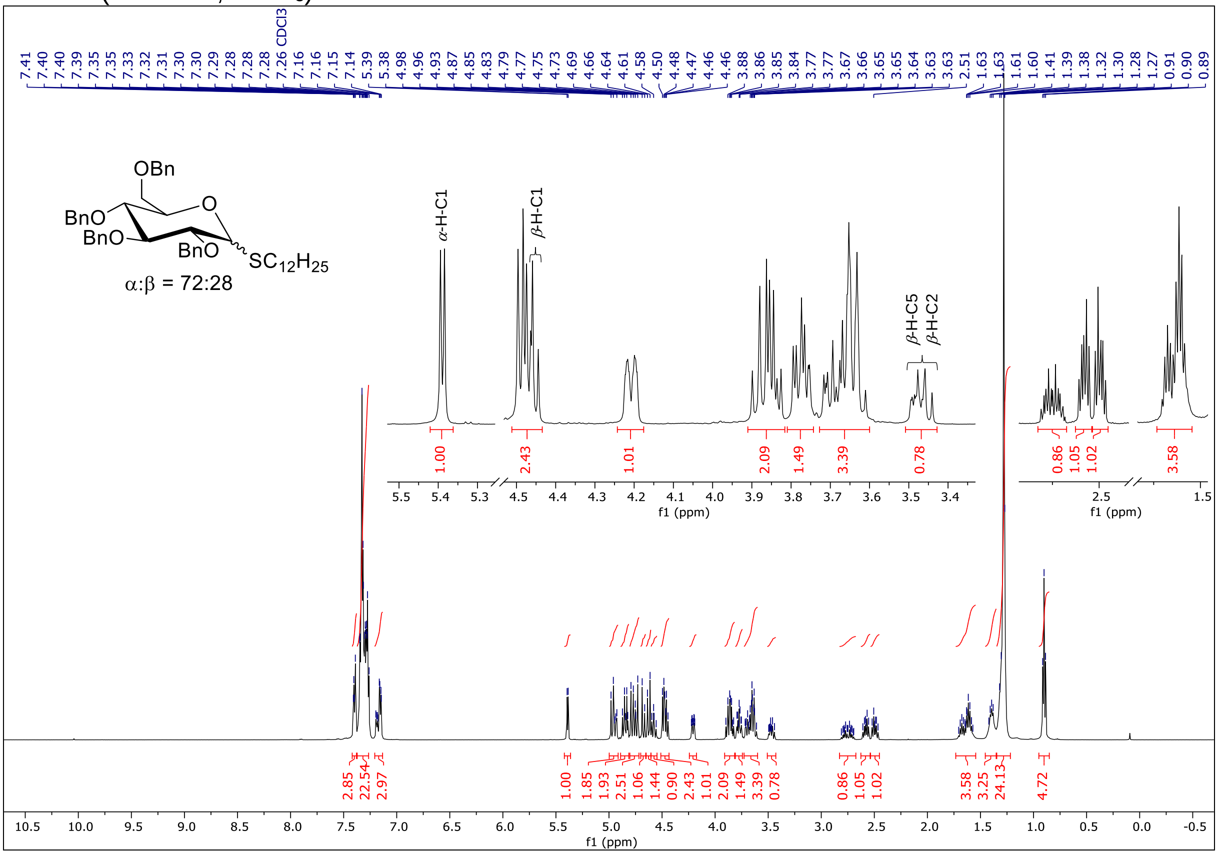

^{13}C NMR (126 MHz, $CDCl_3$)

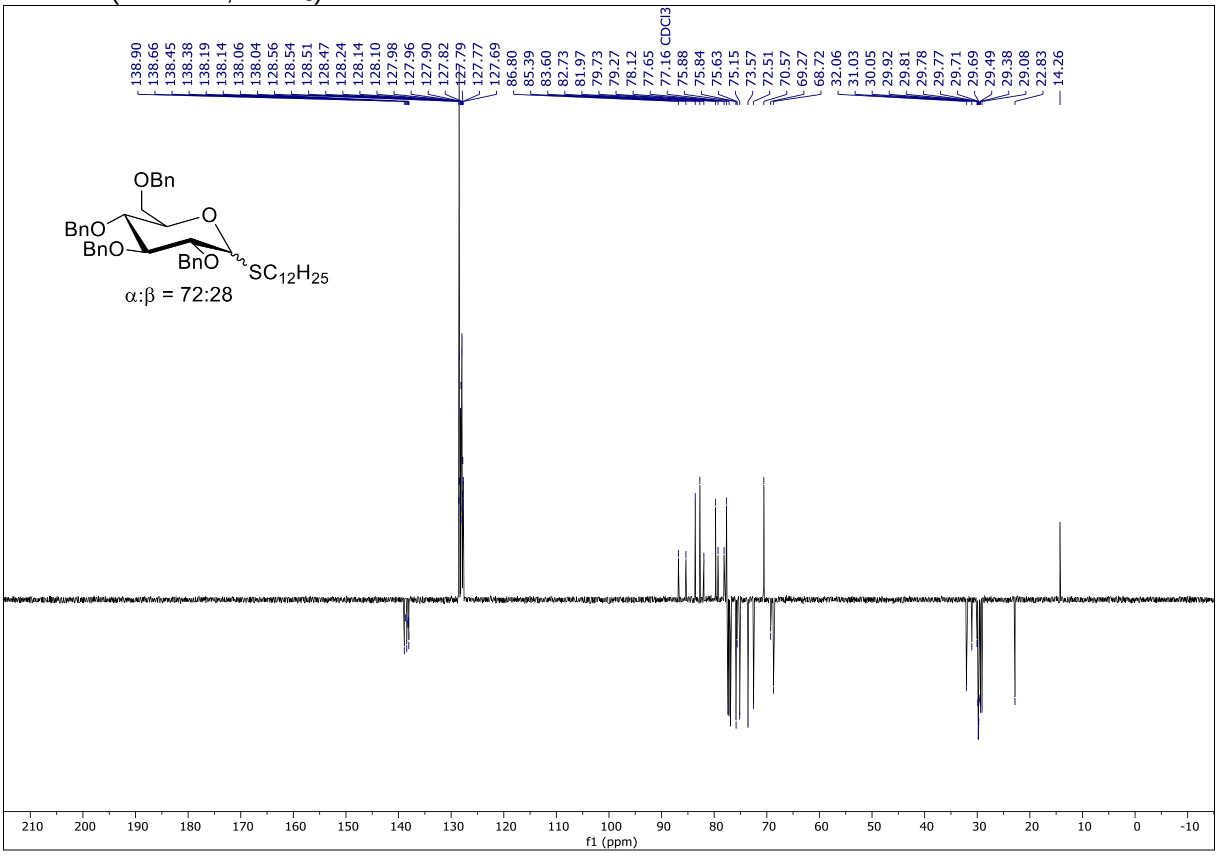

18e

^{1}H NMR (500 MHz, $CDCl_3$)

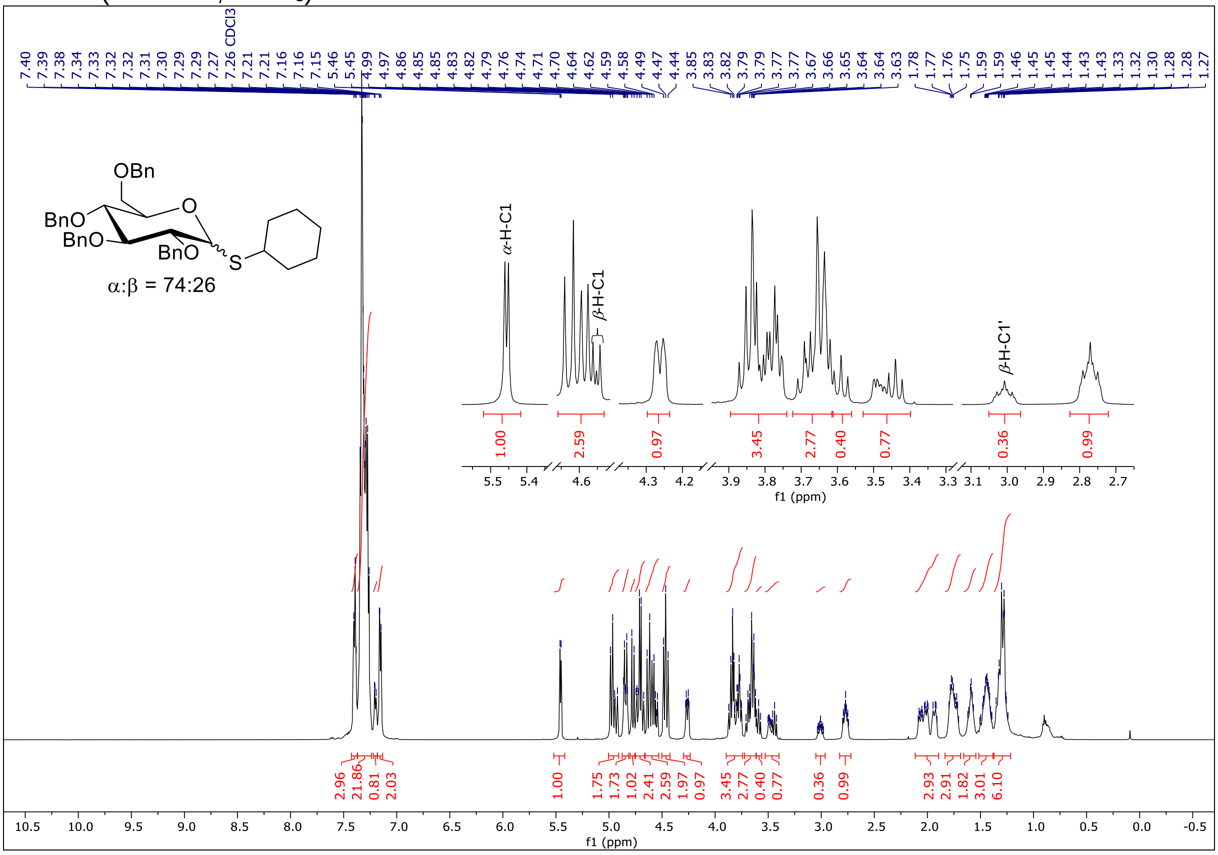

^{13}C NMR (126 MHz, $CDCl_3$)

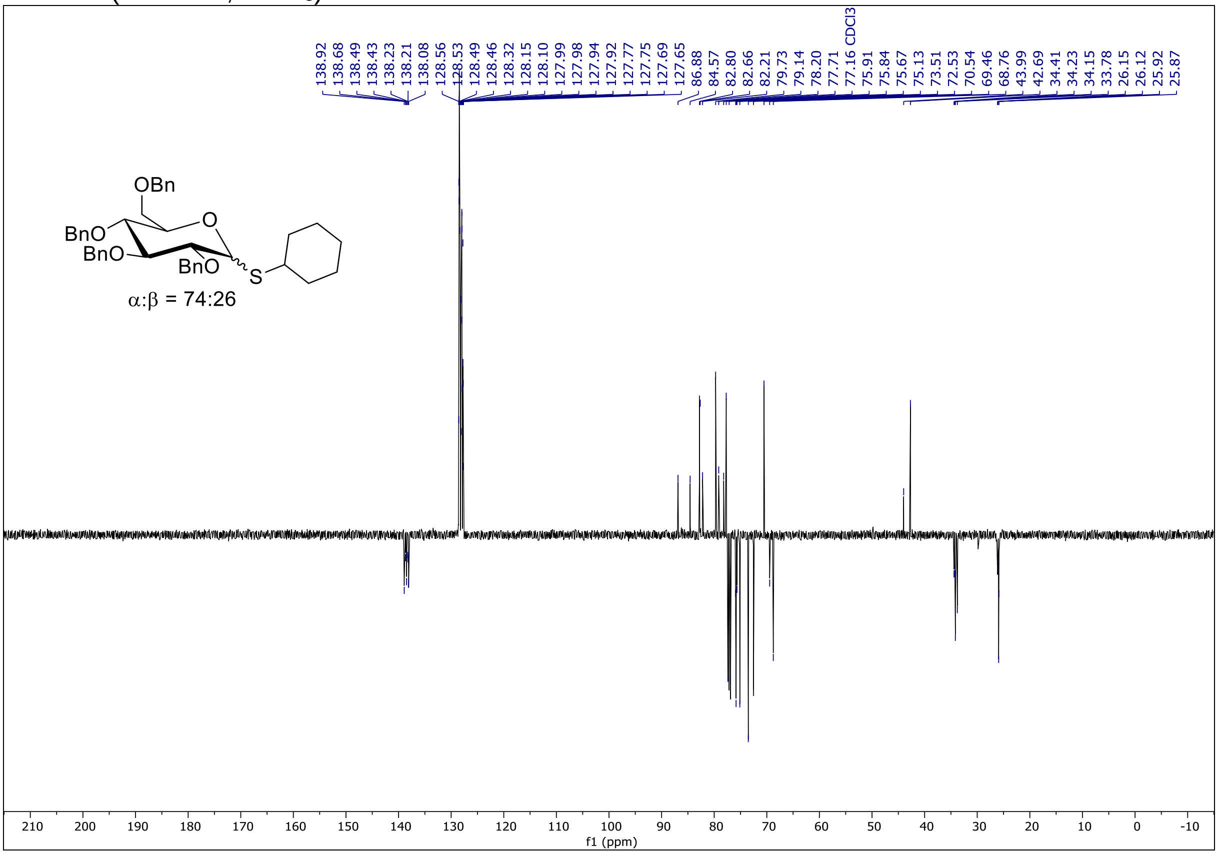

18f

^1H NMR (500 MHz, $CDCl_3$)

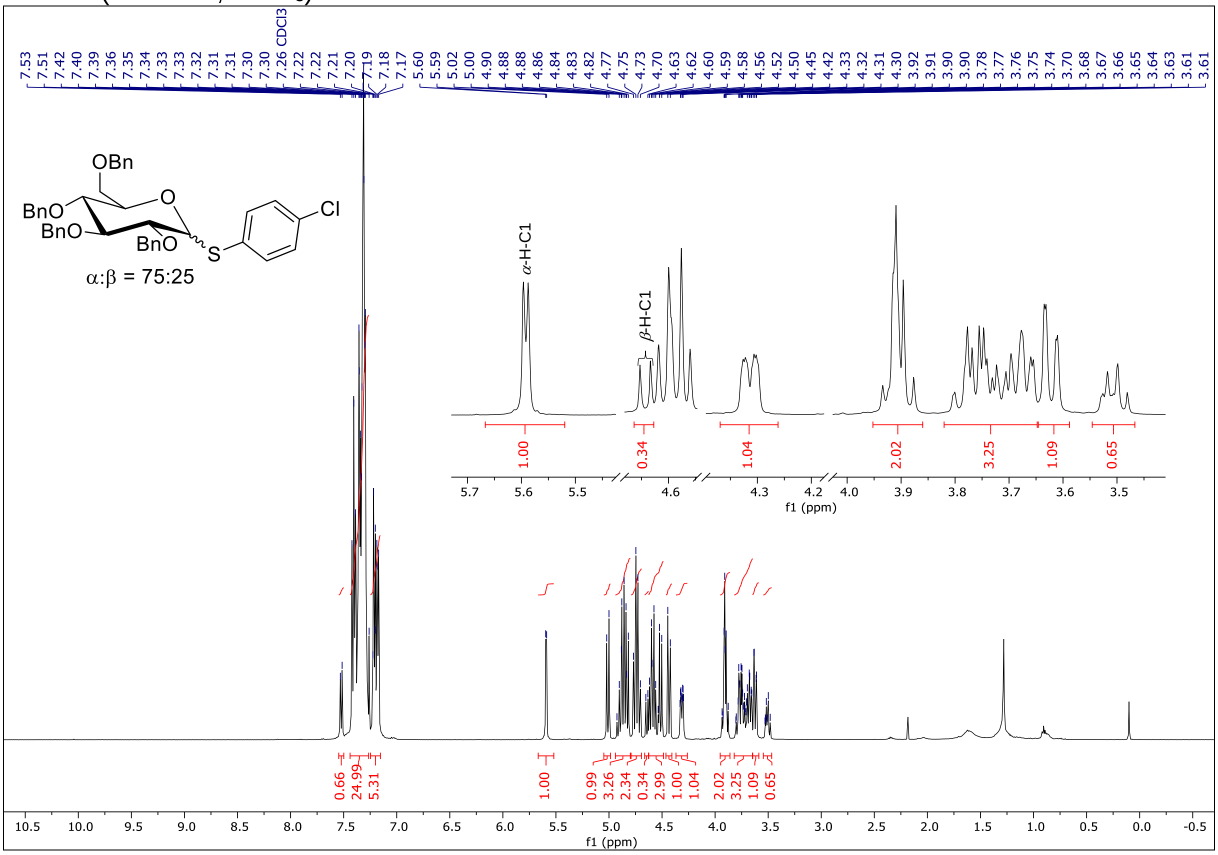

^{13}C NMR (126 MHz, $CDCl_3$)

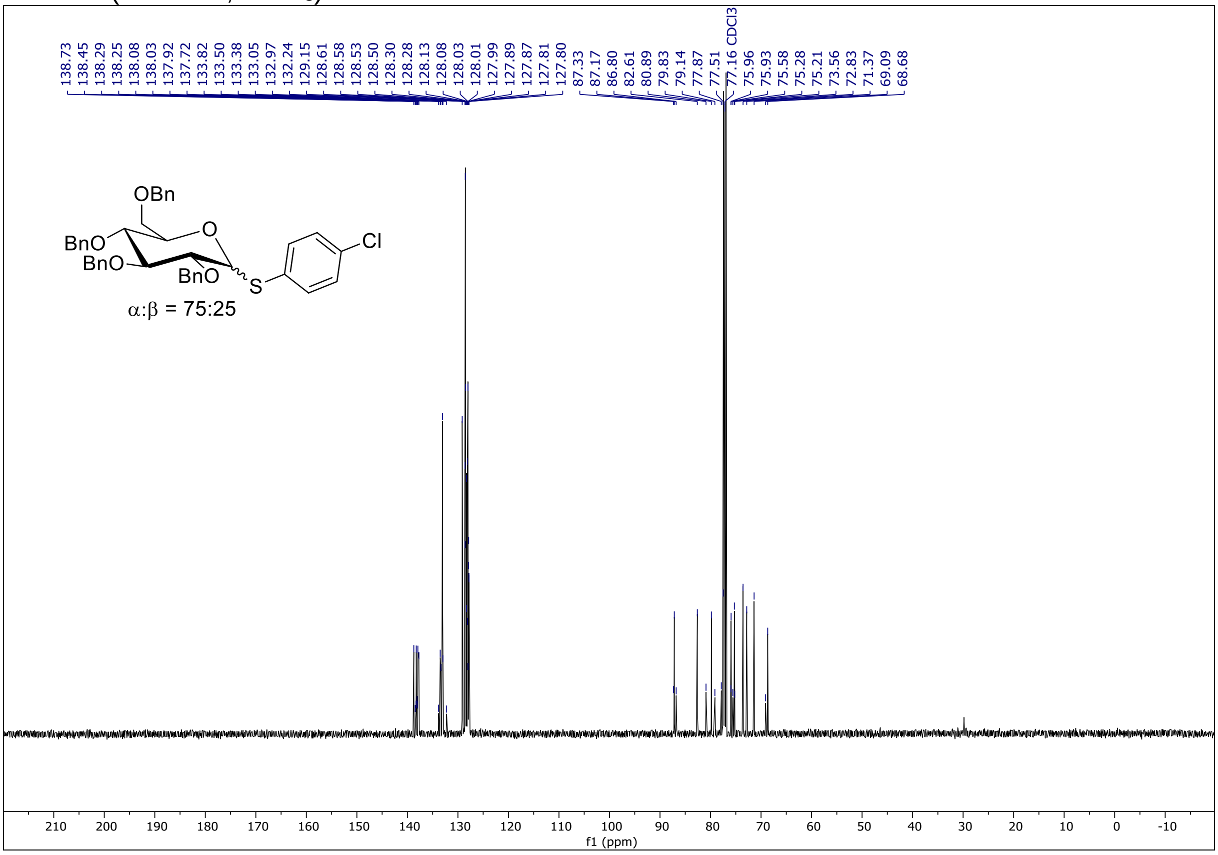

α-**20**

^{1}H NMR (500 MHz, $CDCl_3$)

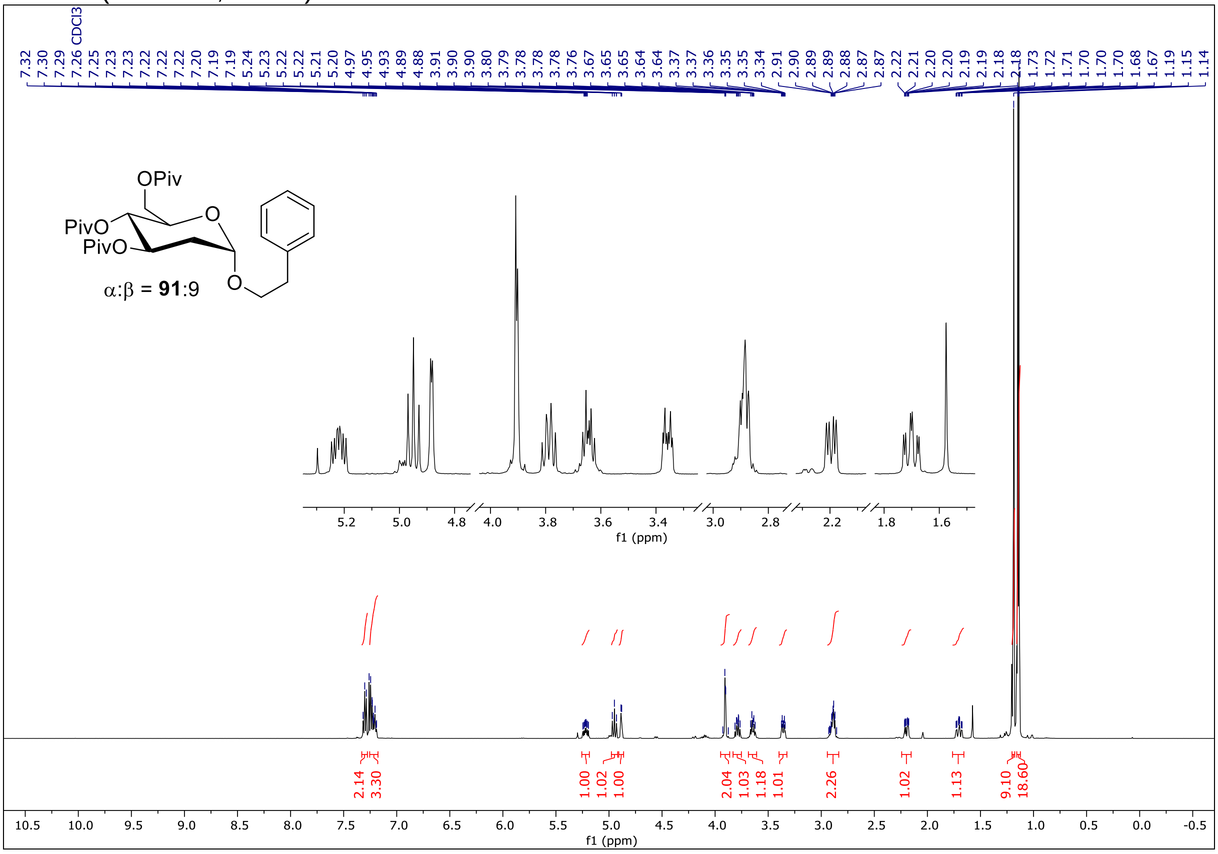

^{13}C NMR (126 MHz, $CDCl_3$)

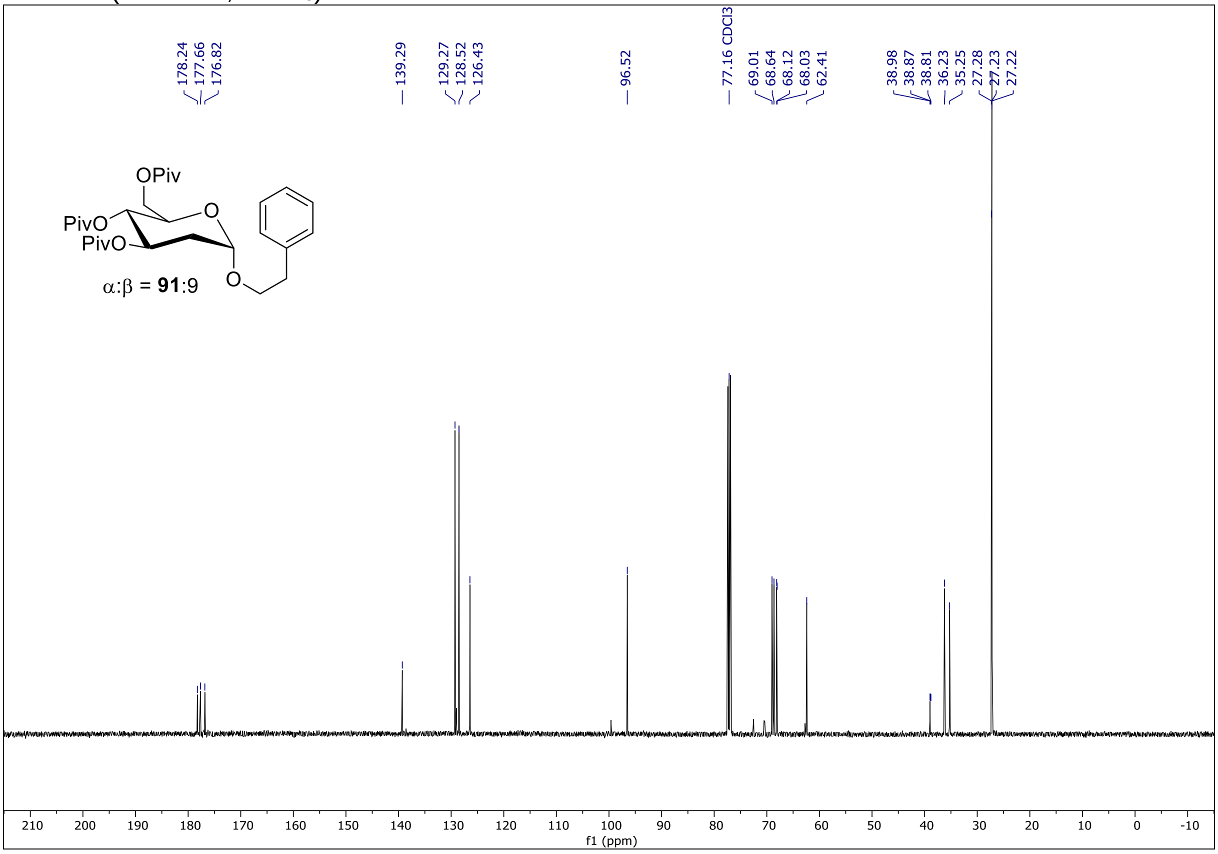

β-**20**

^{1}H NMR (500 MHz, CDCl$_3$)

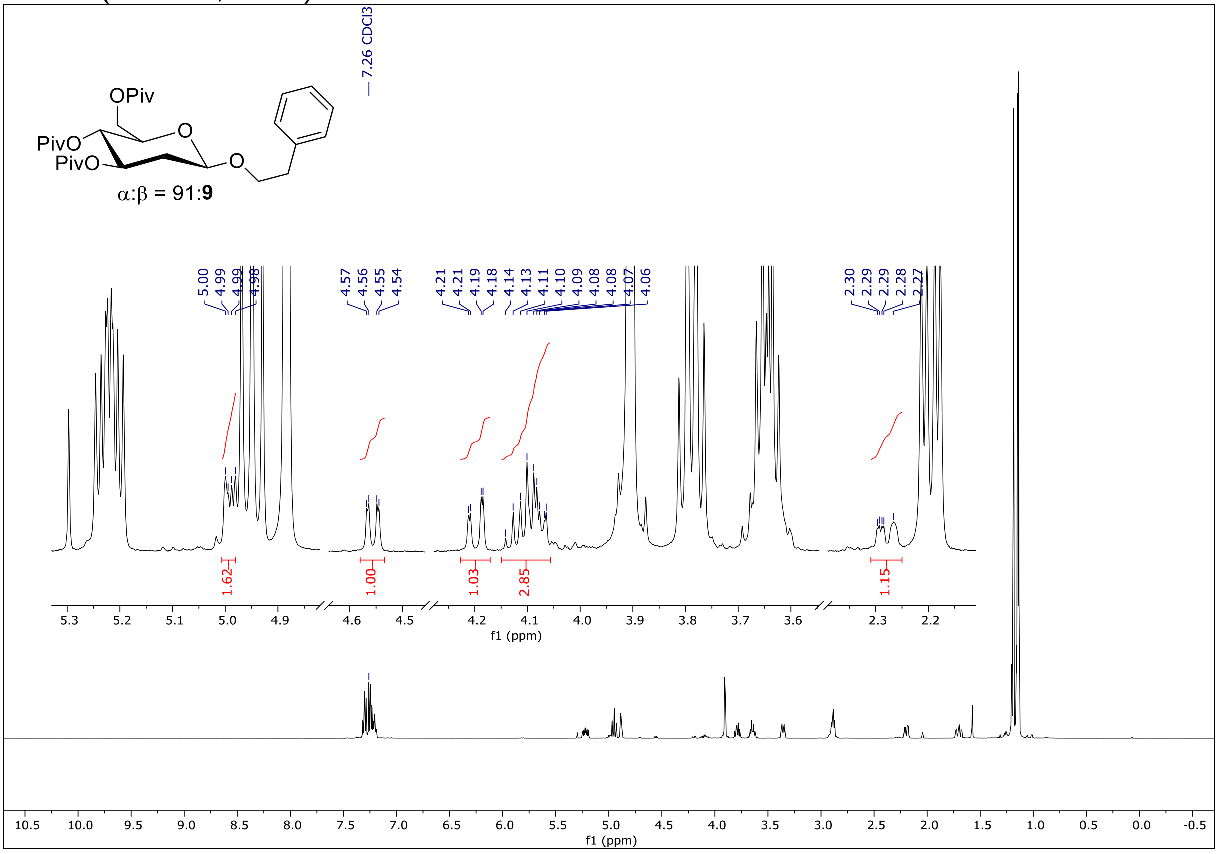

^{13}C NMR (126 MHz, CDCl$_3$)

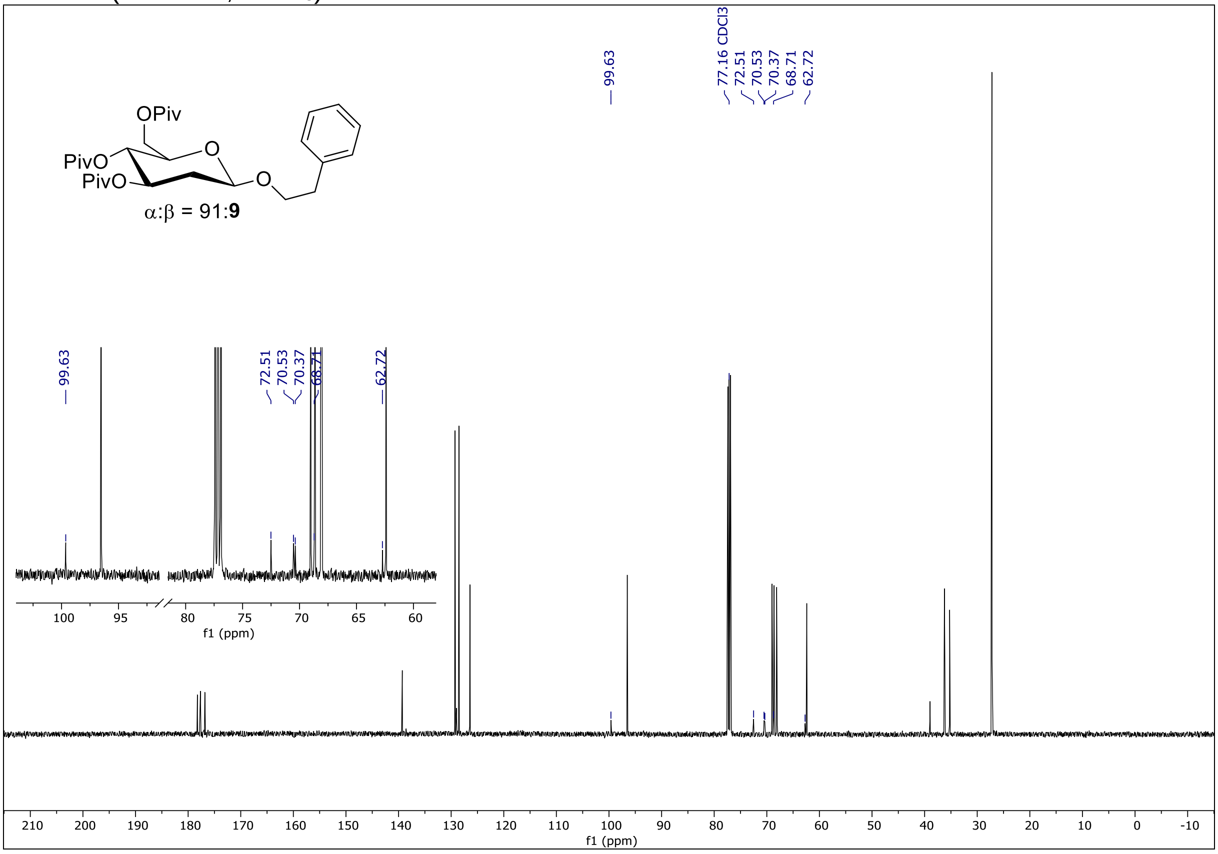

4. SIDE-PRODUCTS

α-**4**

^1H NMR (500 MHz, $CDCl_3$)

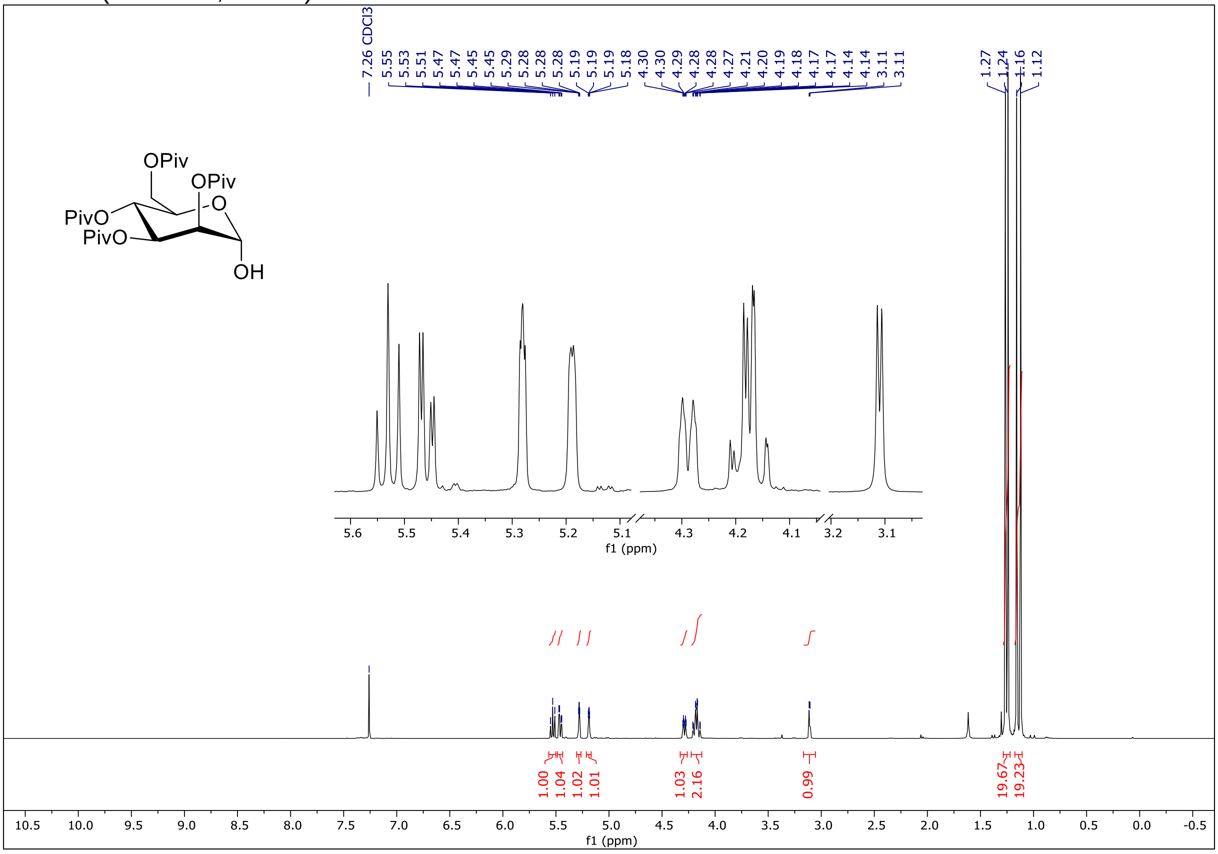

^{13}C NMR (75.5 MHz, $CDCl_3$)

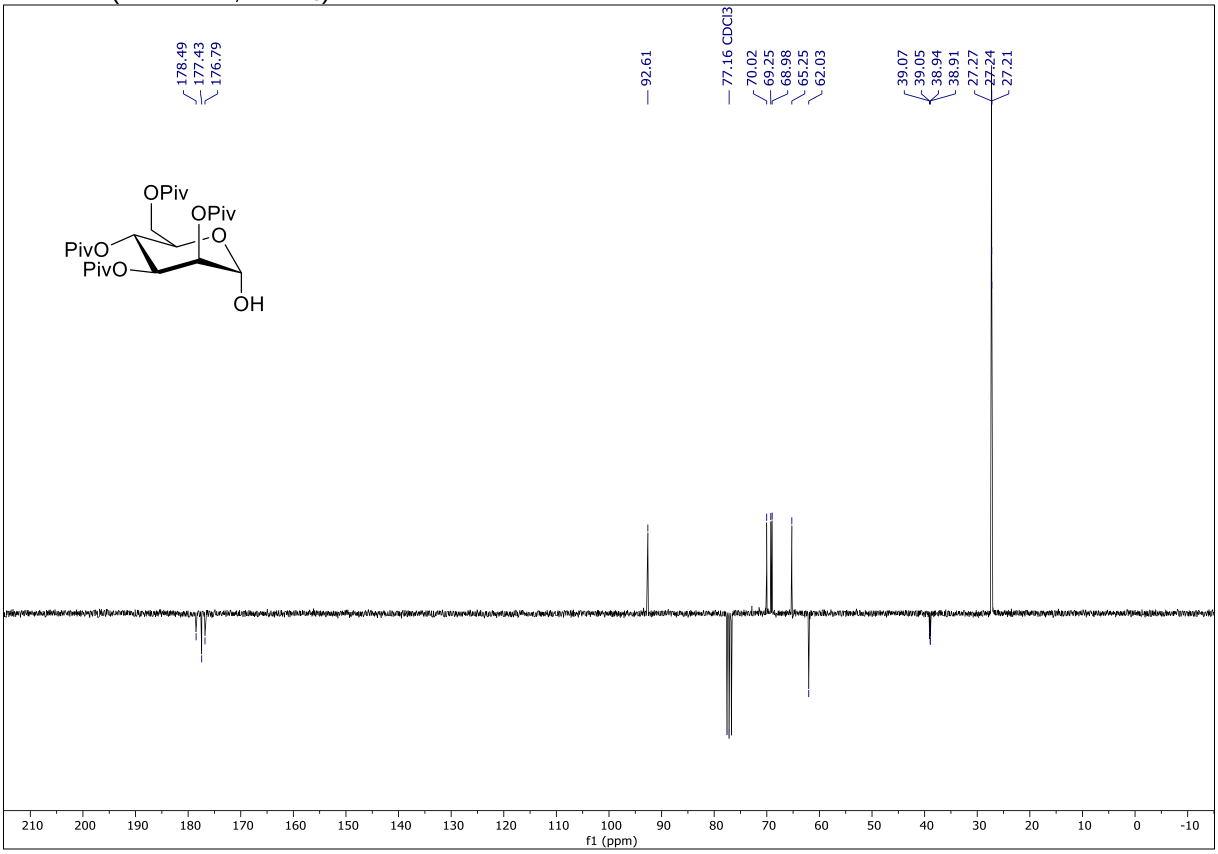

β-6

^1H NMR (500 MHz, $CDCl_3$)

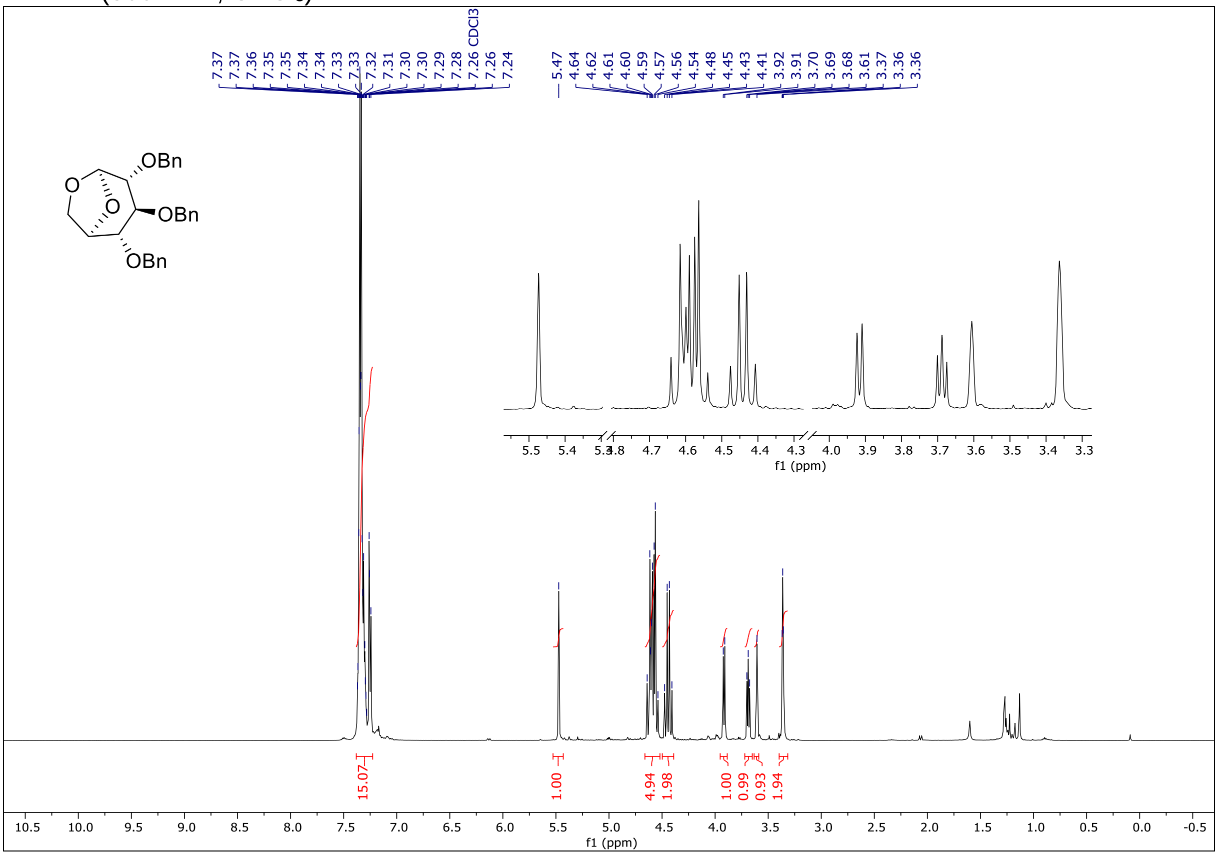

^{13}C NMR (126 MHz, $CDCl_3$)

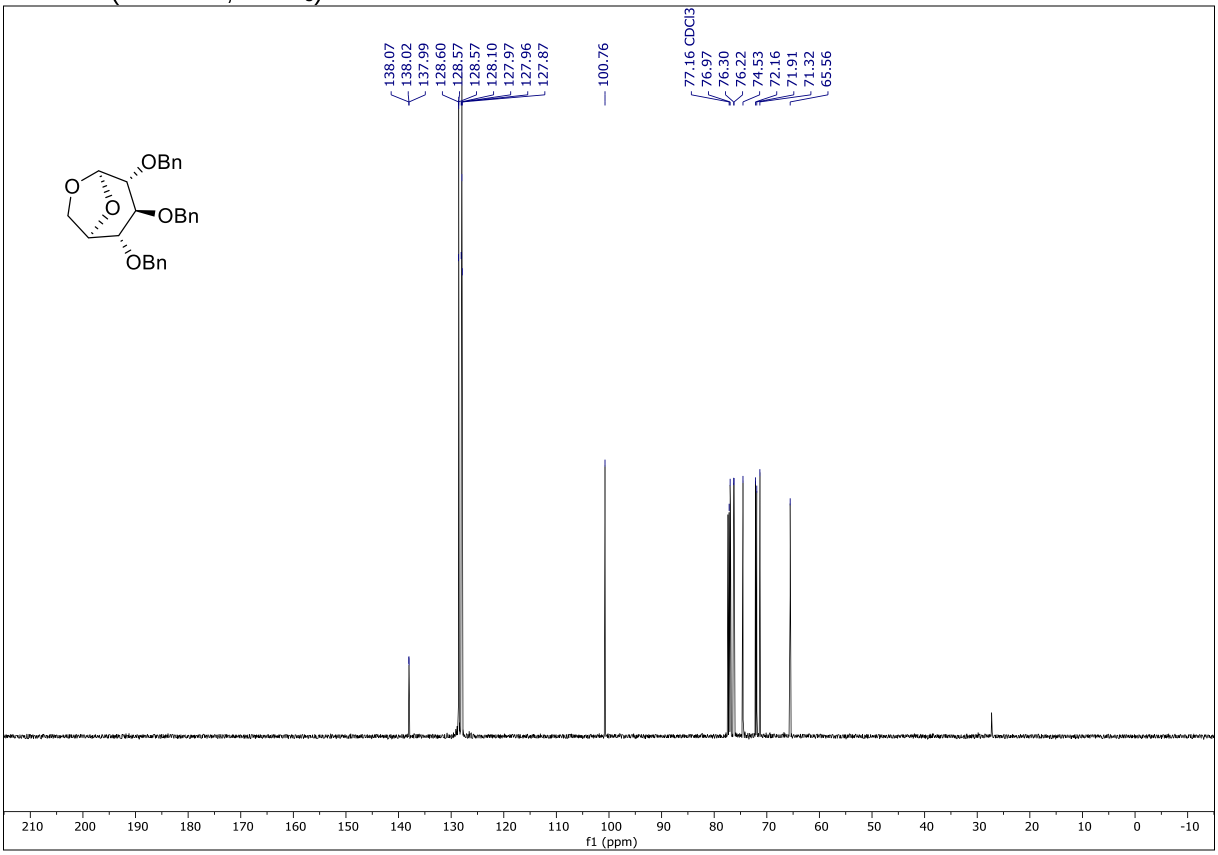

α,α-**S14**

^{1}H NMR (500 MHz, $CDCl_3$)

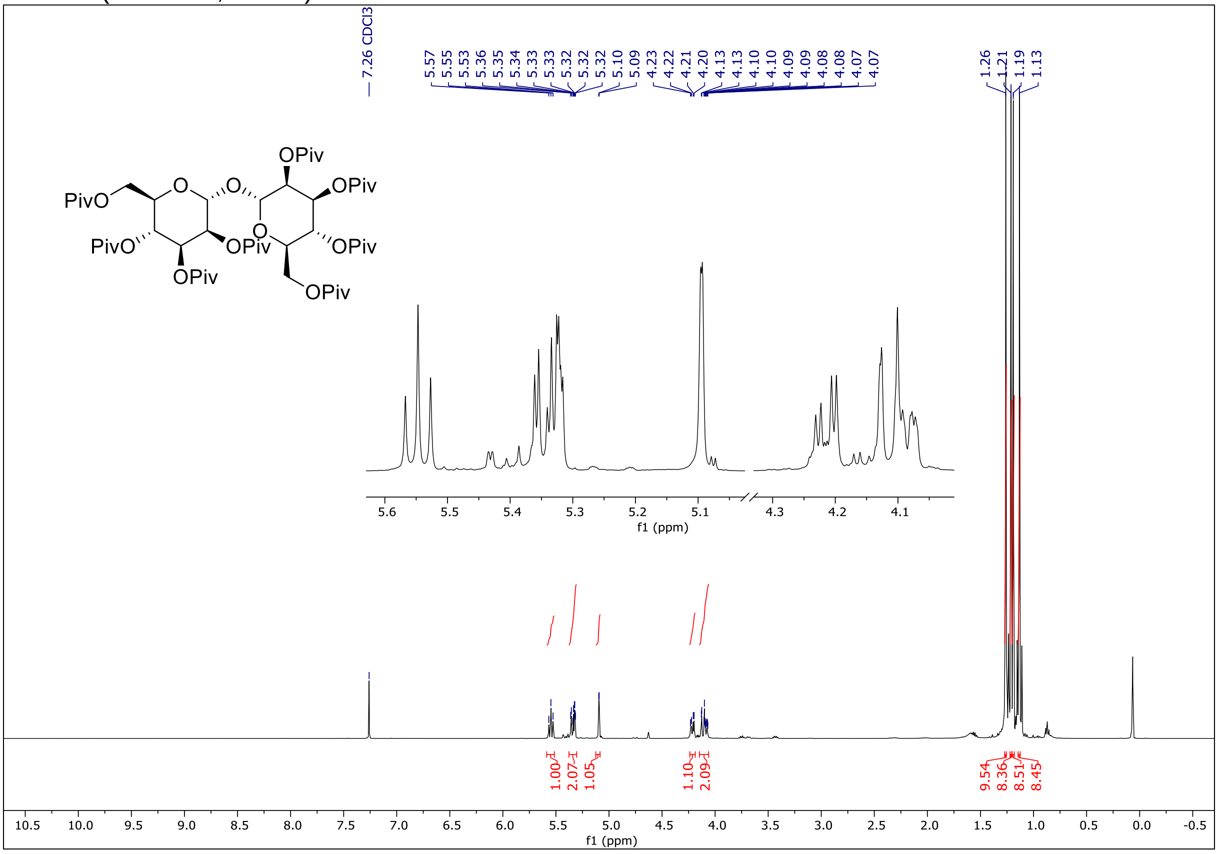

^{13}C NMR (126 MHz, $CDCl_3$)

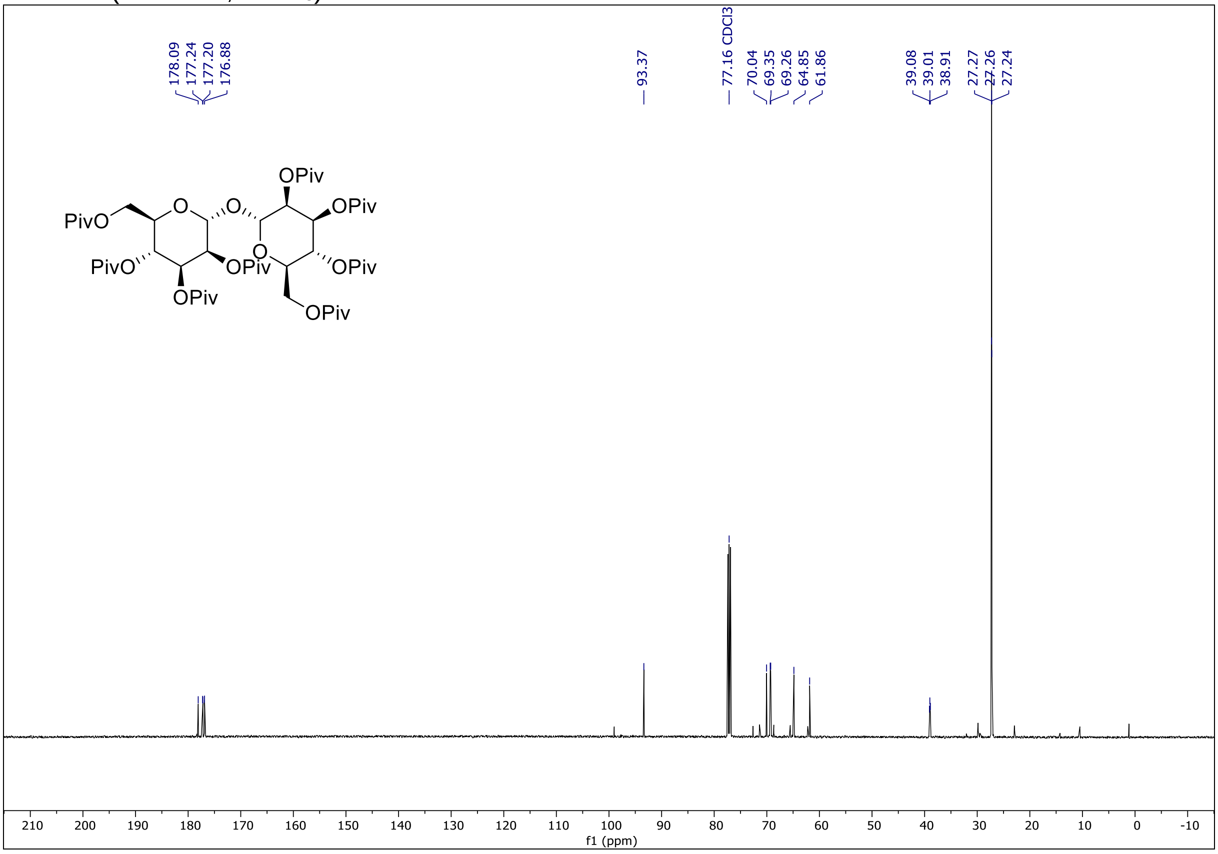

α,α-**S14**

^{1}H-^{13}C HMBC ($CDCl_3$)

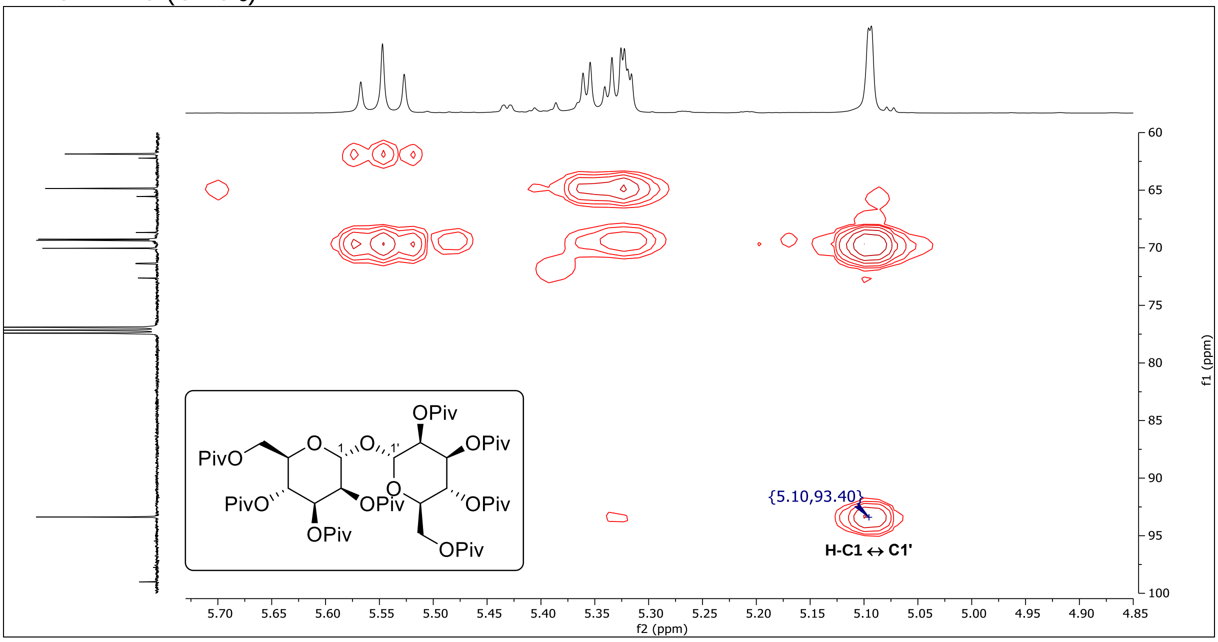

α-**S15**

^{1}H NMR (500 MHz, $CDCl_3$)

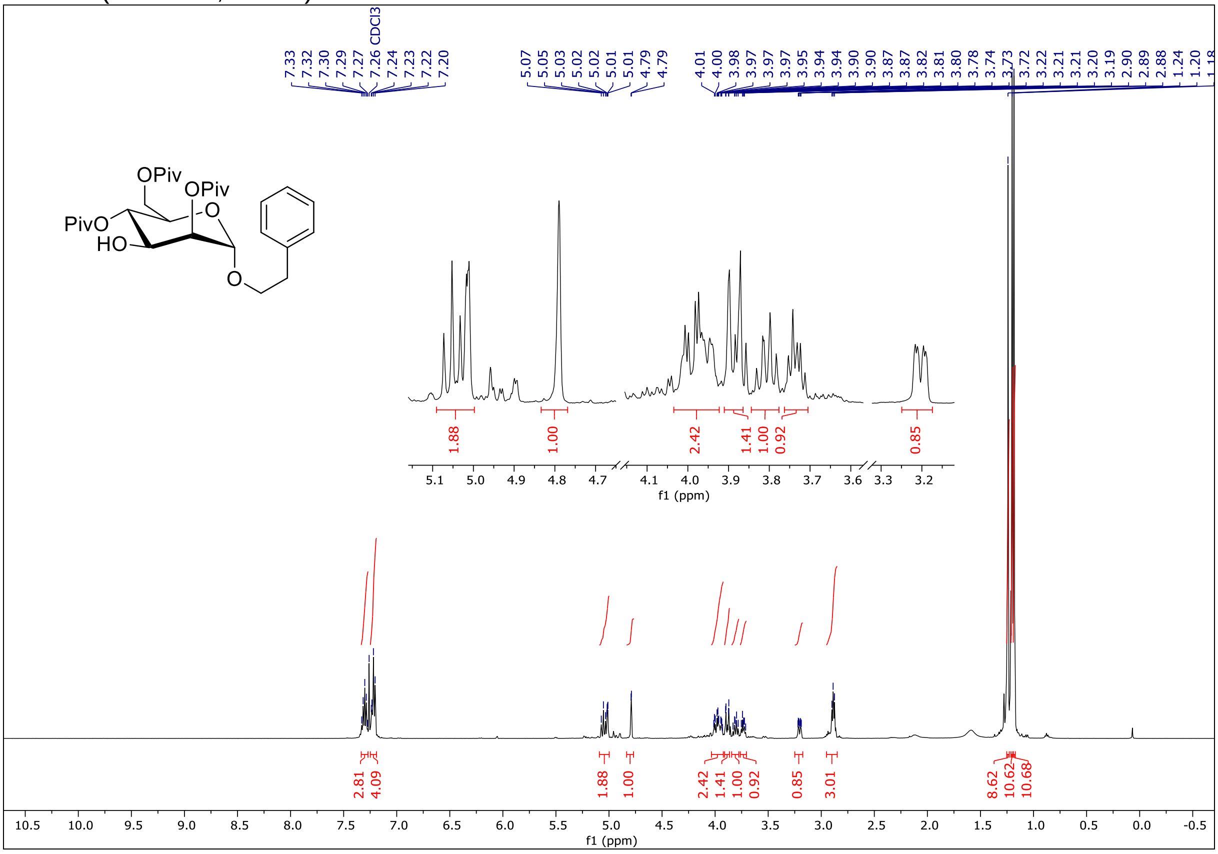

^{13}C NMR (126 MHz, $CDCl_3$)

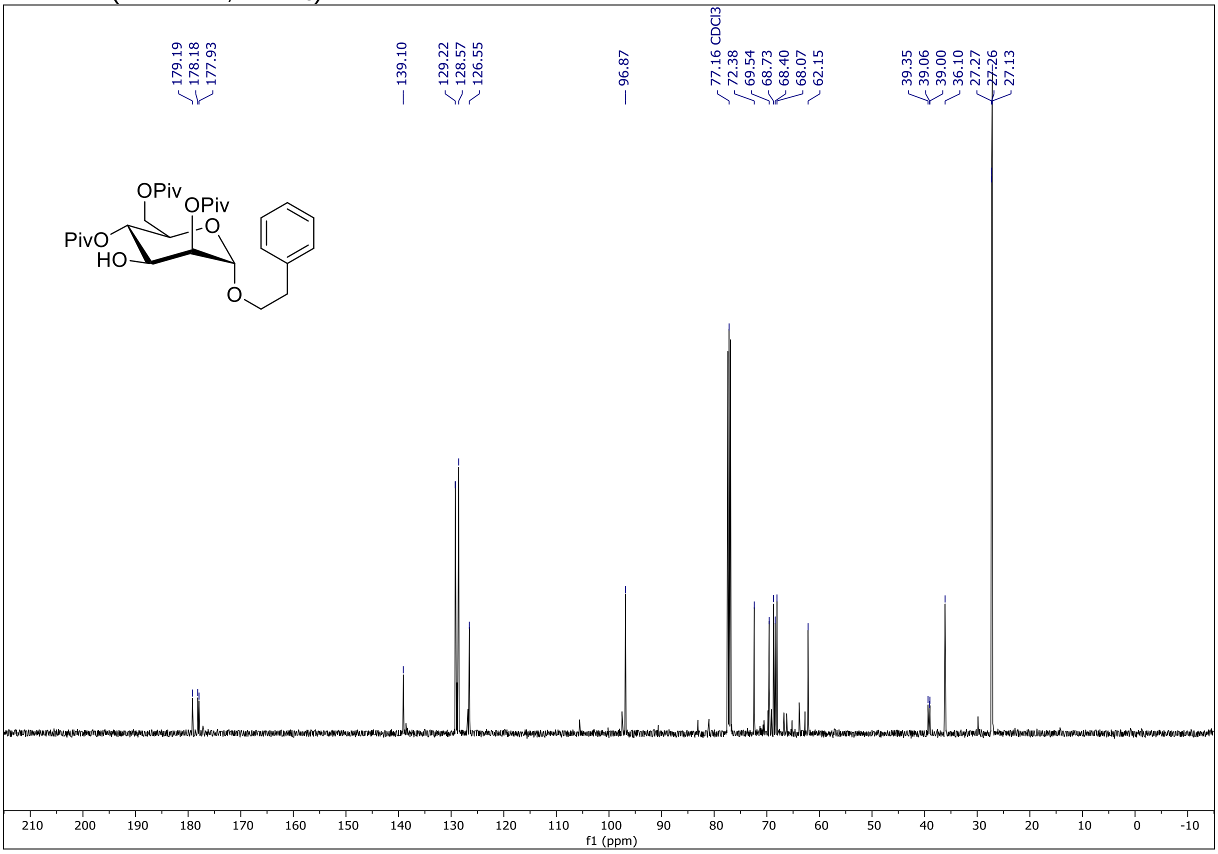

α-**S15**

^1H-^{13}C HMBC ($CDCl_3$)

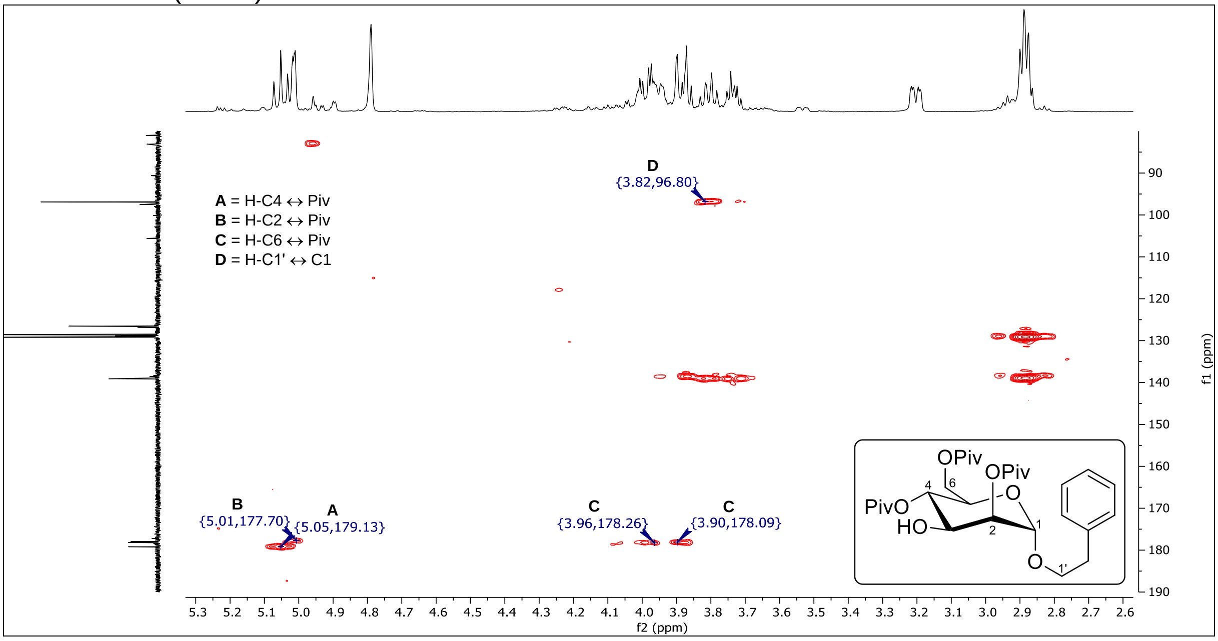

α-**S16**

^{1}H NMR (500 MHz, CDCl$_3$)

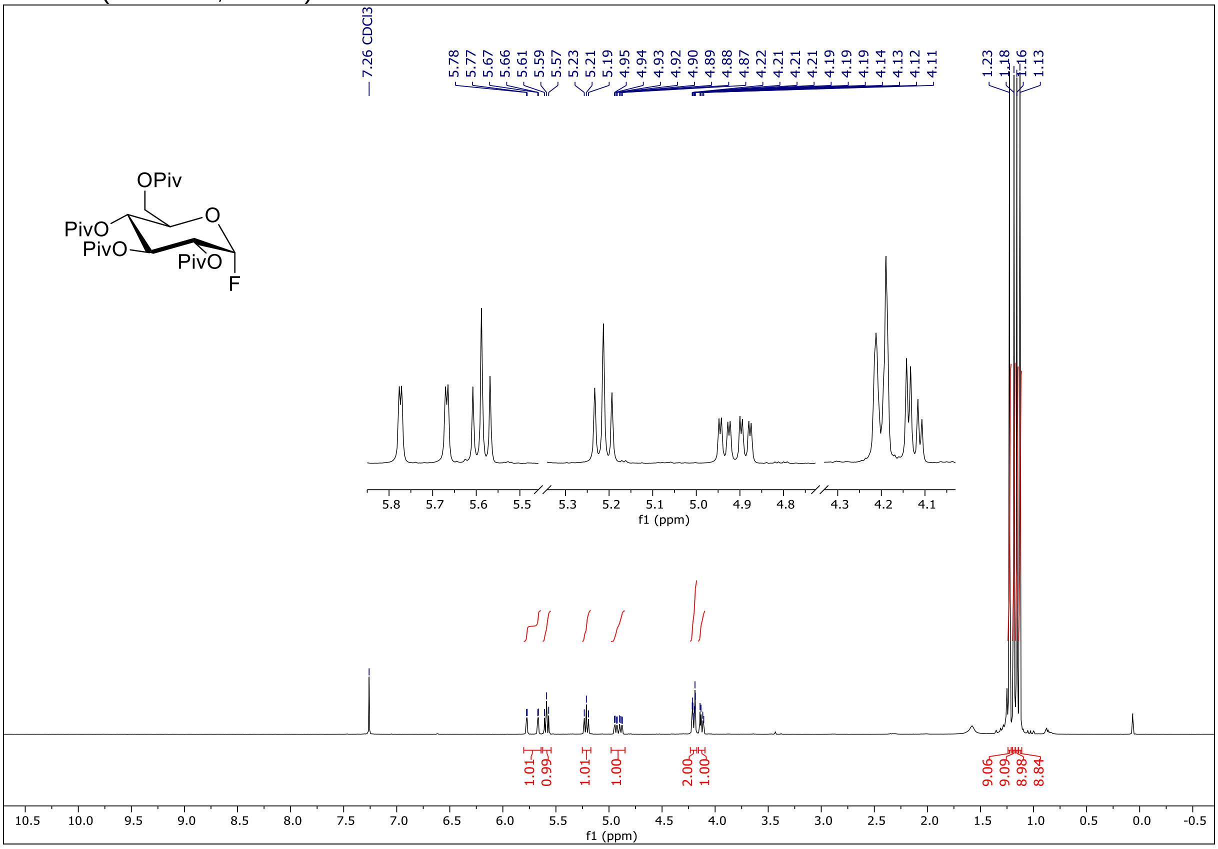

^{13}C NMR (126 MHz, CDCl$_3$)

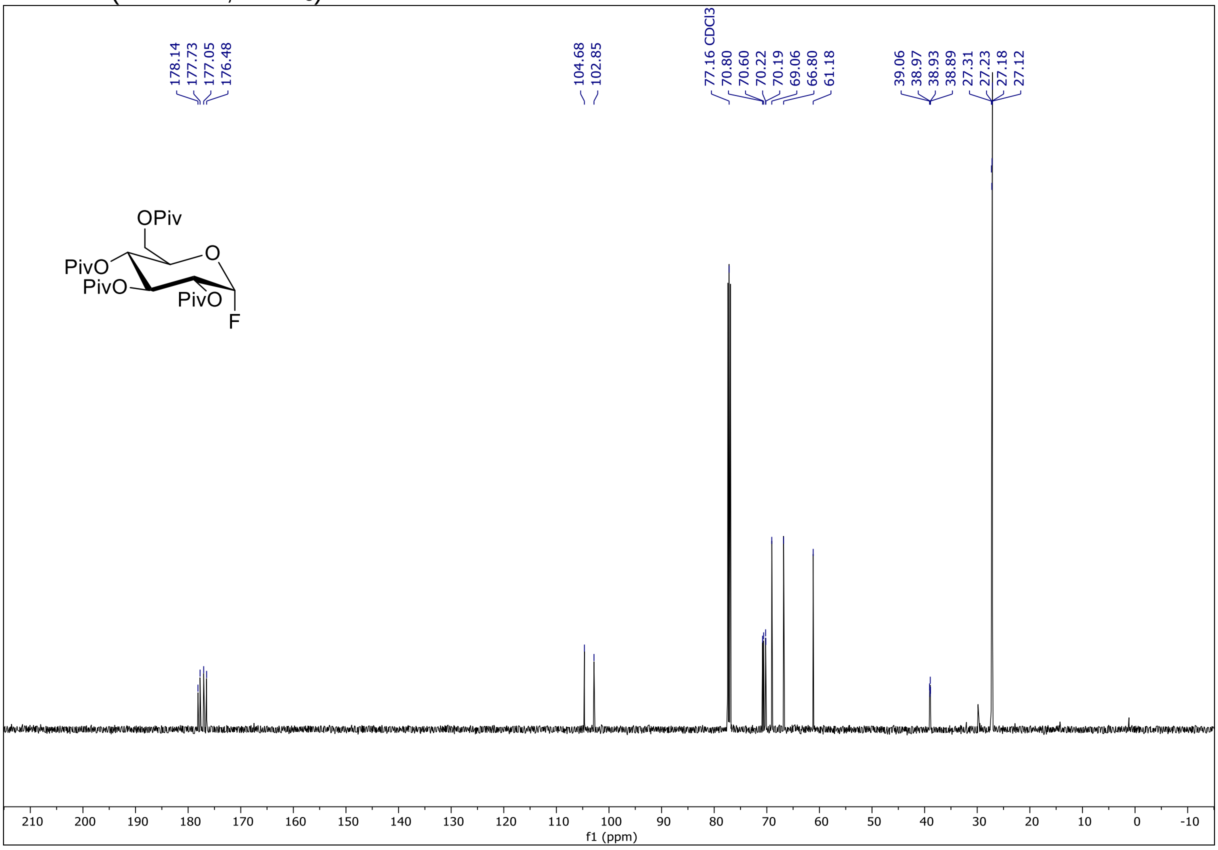

^{1}H NMR (500 MHz, $CDCl_3$)

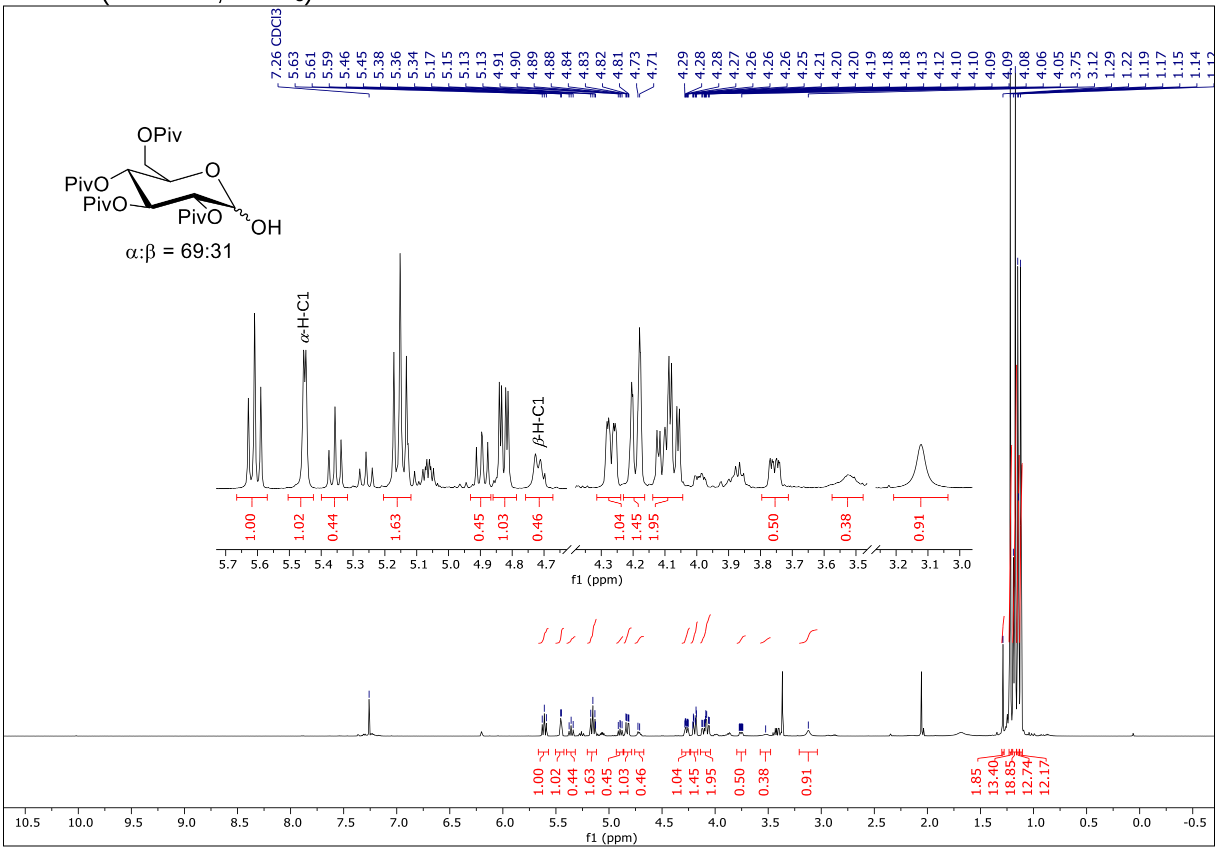

^{13}C NMR (126 MHz, $CDCl_3$)

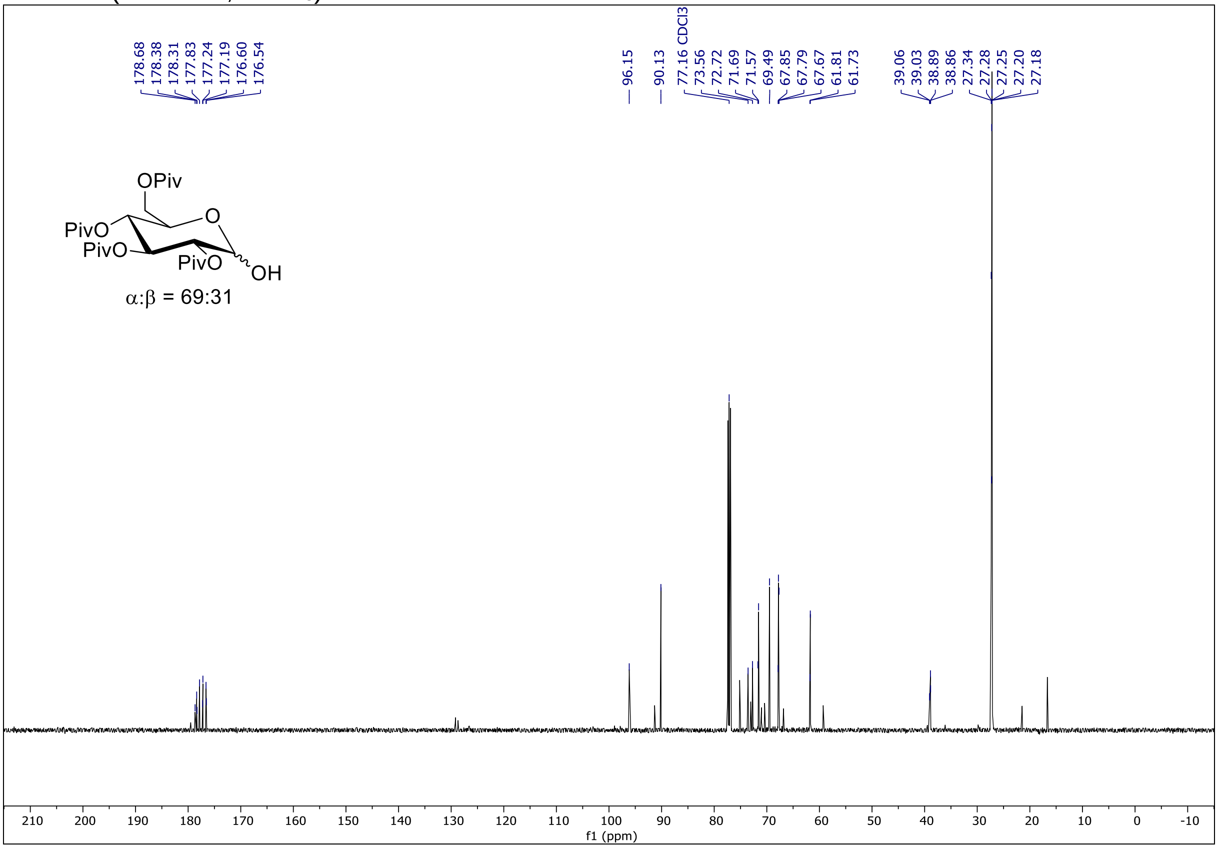

α-**S18**

^{1}H NMR (500 MHz, $CDCl_3$)

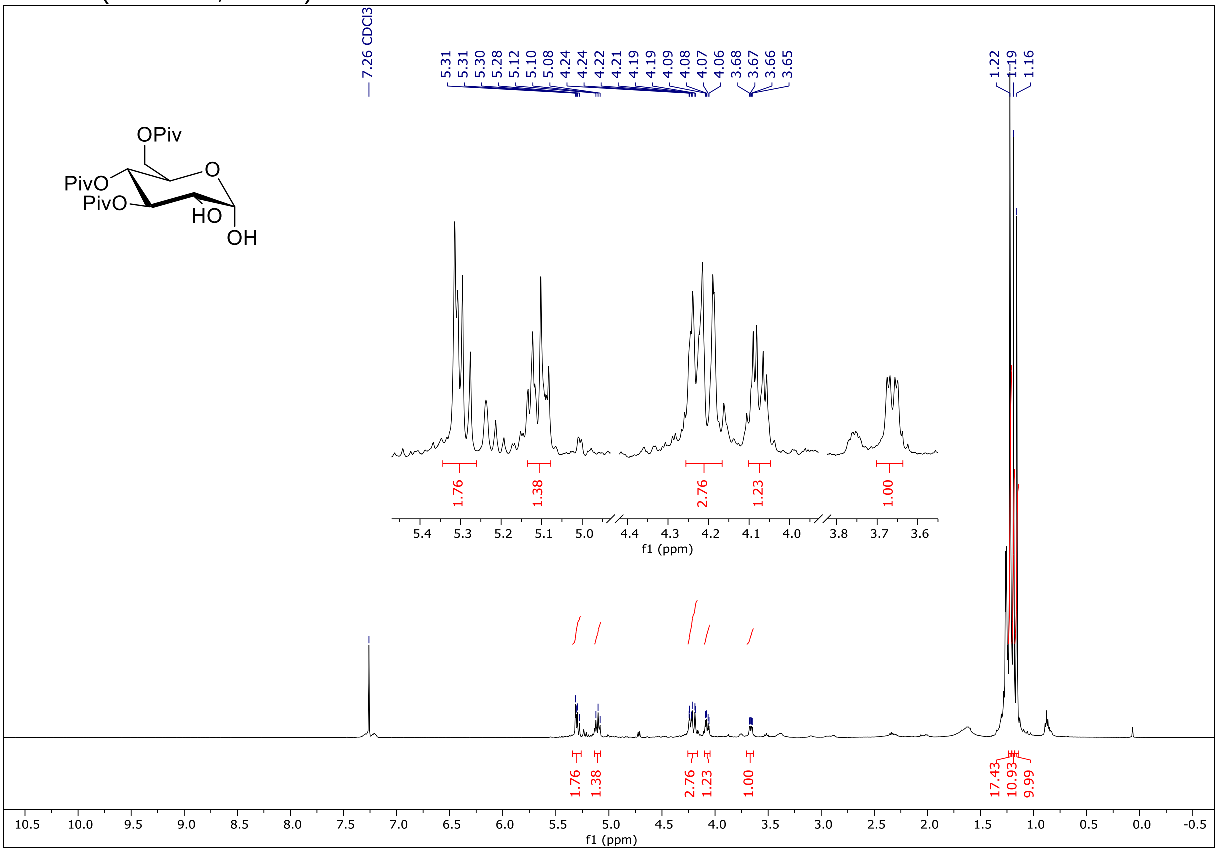

^{13}C NMR (126 MHz, $CDCl_3$)

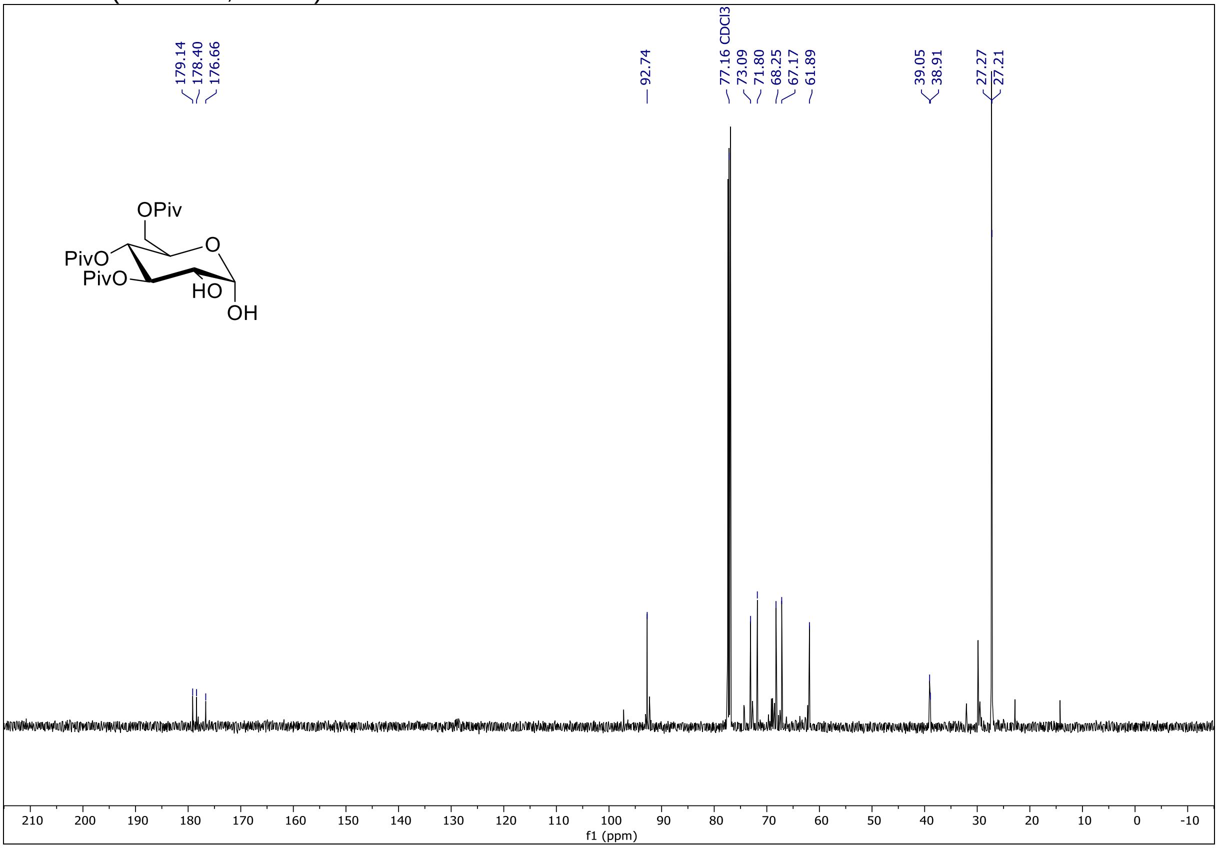

α-**S18**

^1H-^{13}C HMBC ($CDCl_3$)

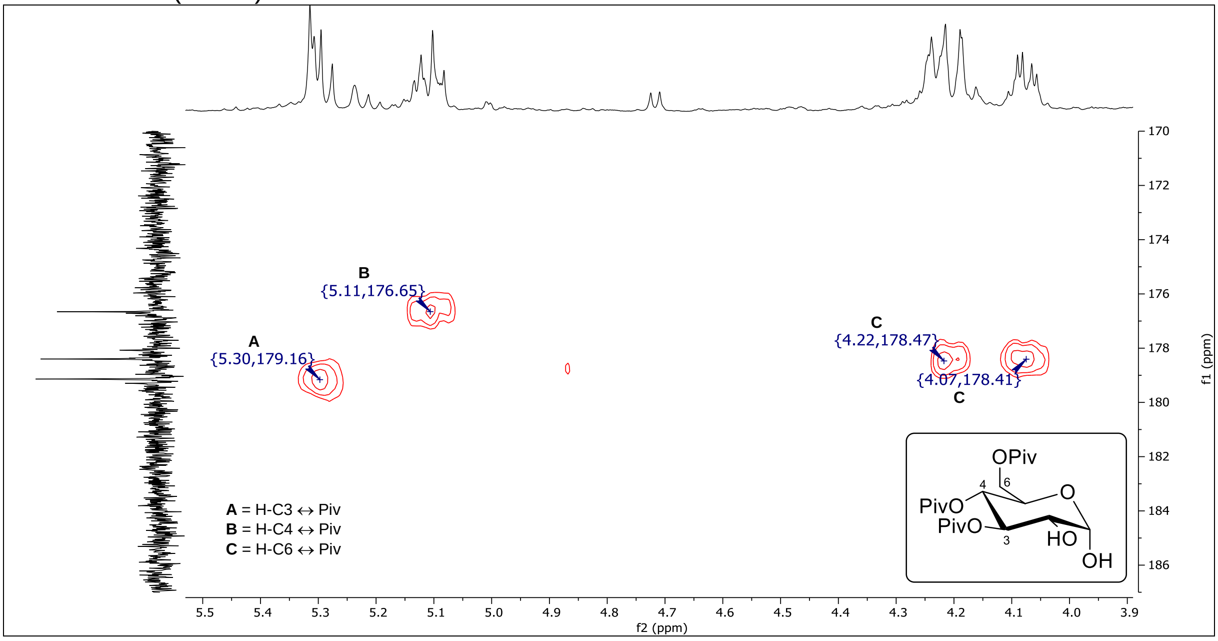

α-**S19** & β-**S20** (66:34)

^{1}H NMR (500 MHz, $CDCl_3$)

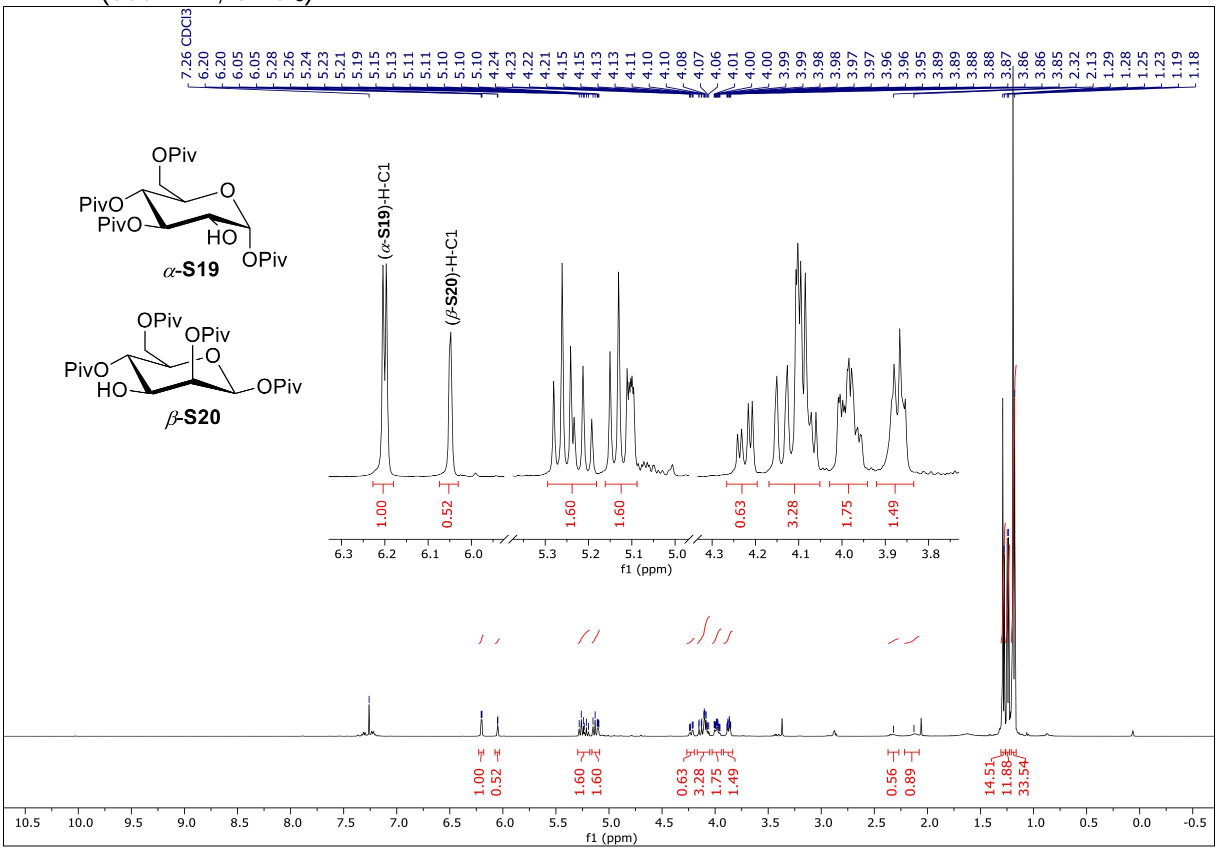

^{13}C NMR (126 MHz, $CDCl_3$)

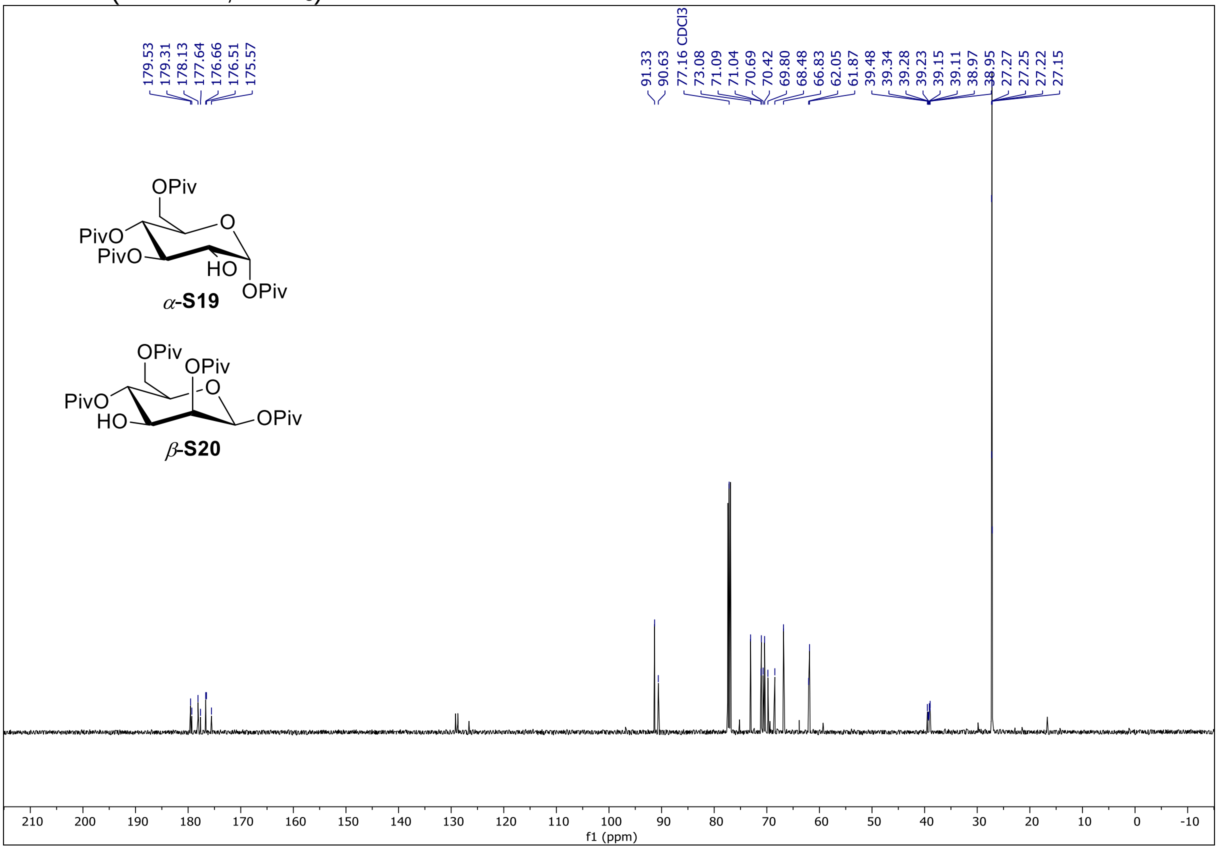

α-**S21**

^{1}H NMR (500 MHz, CDCl$_3$)

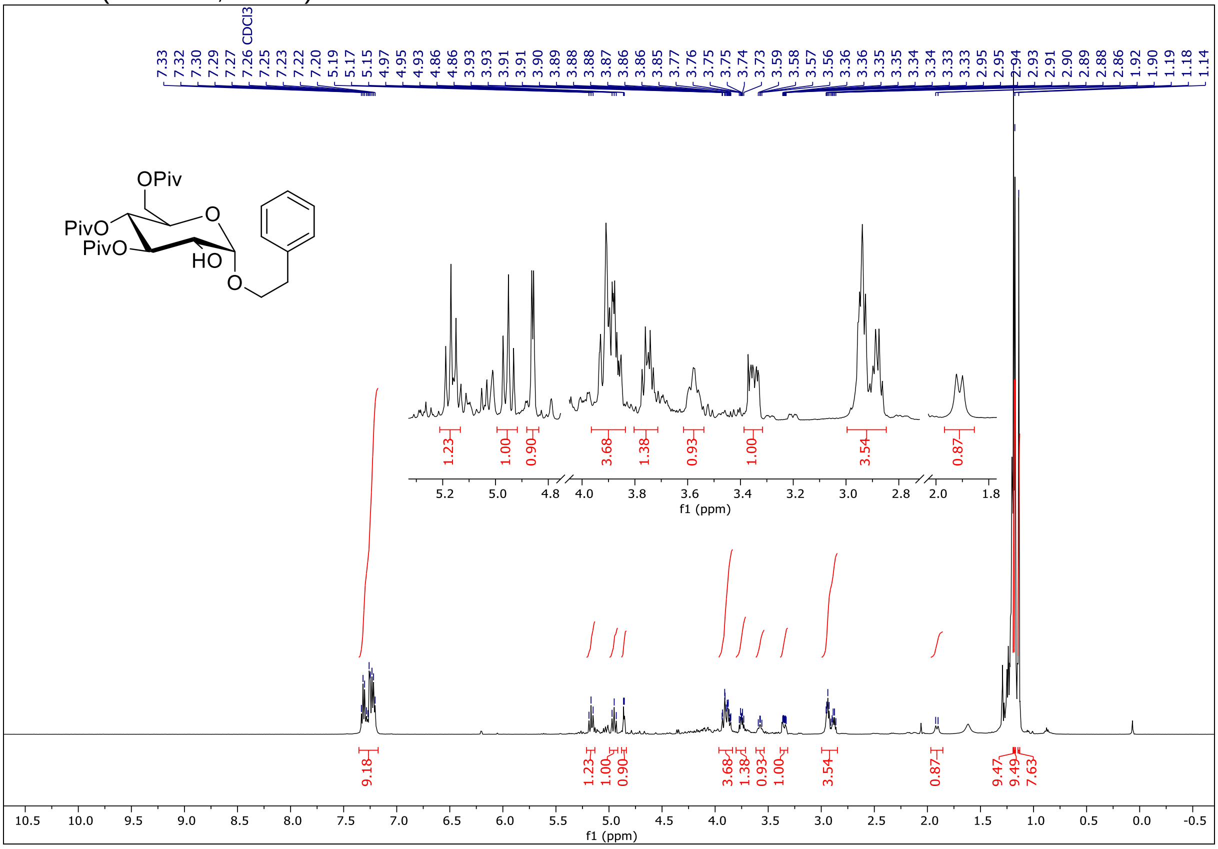

^{13}C NMR (126 MHz, CDCl$_3$)

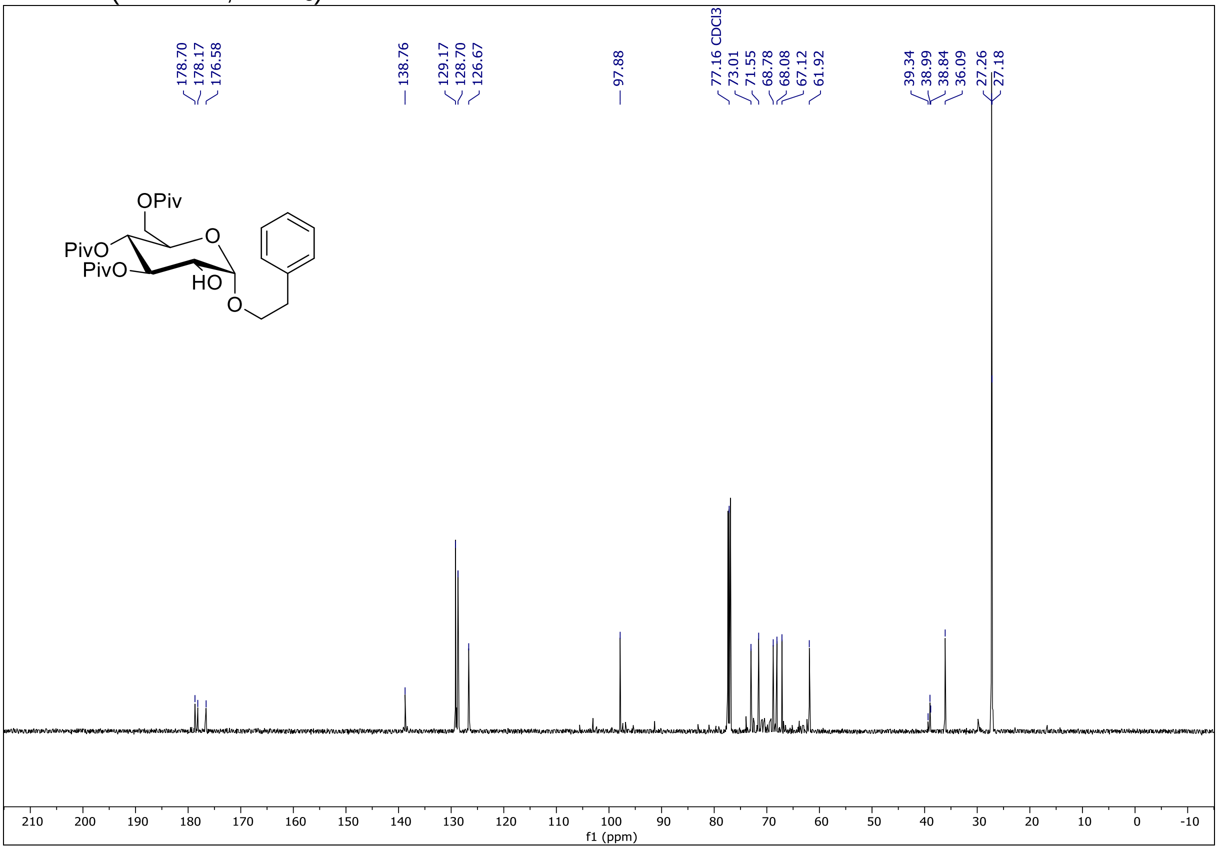

α-**S21**

^{1}H-^{13}C HMBC ($CDCl_3$)

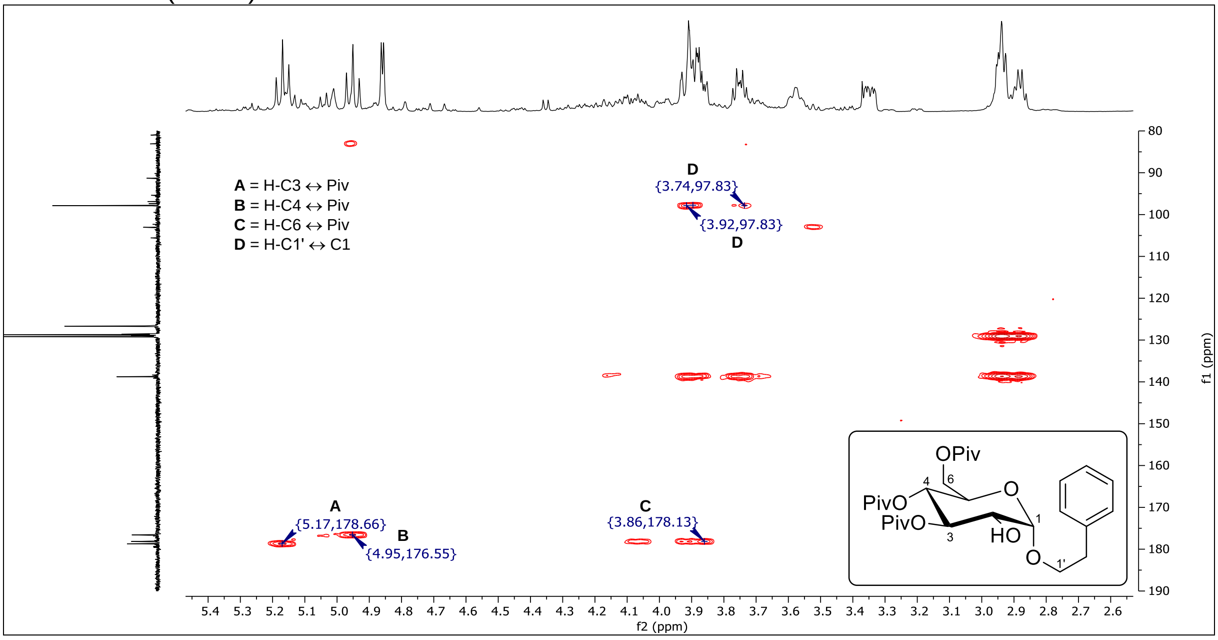

α-**S22**

^{1}H NMR (500 MHz, $CDCl_3$)

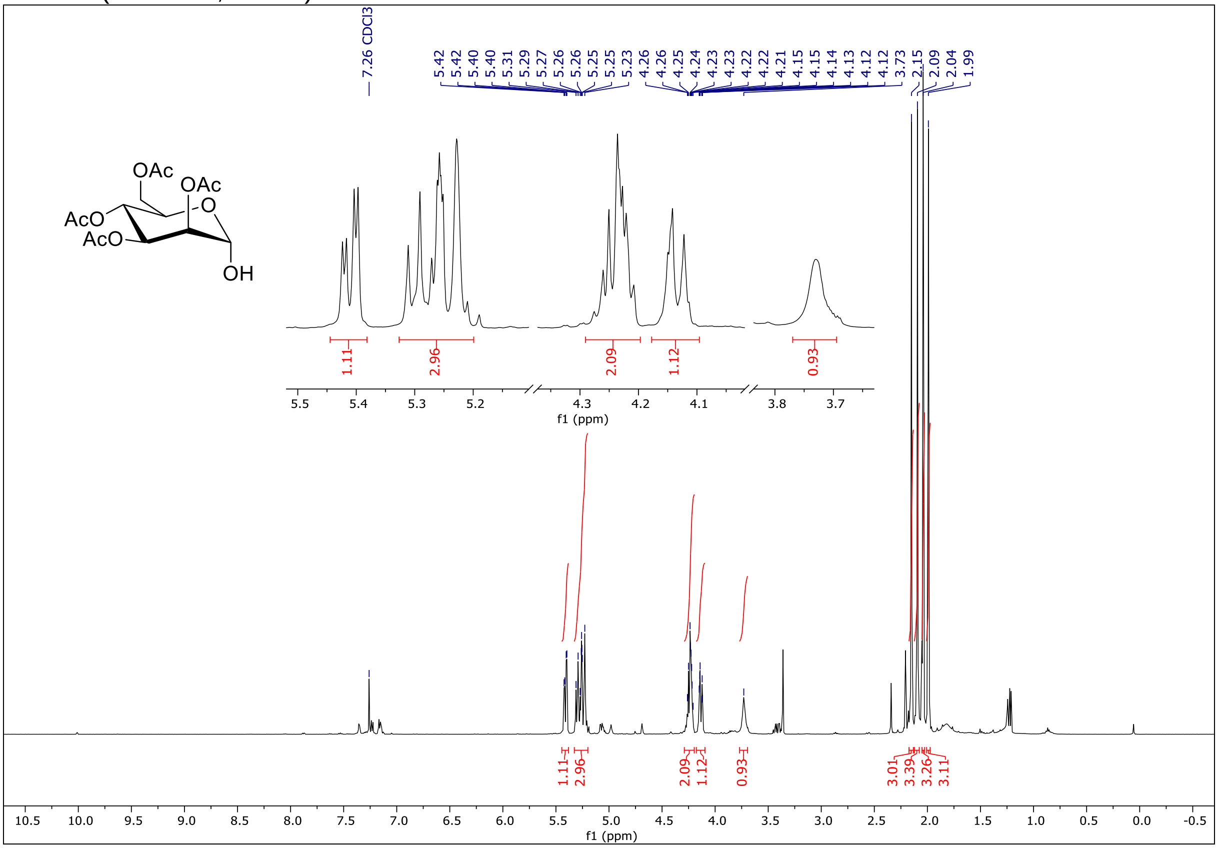

^{13}C NMR (126 MHz, $CDCl_3$)

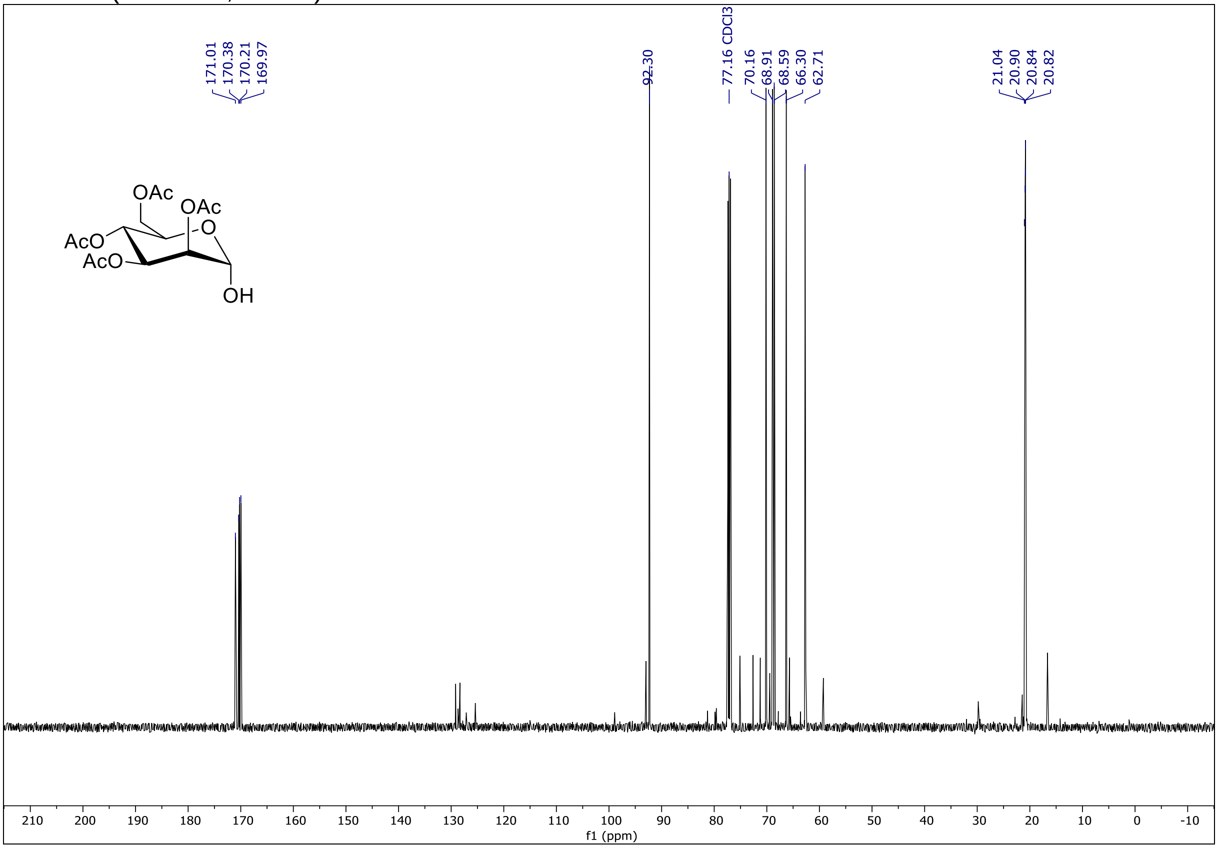

α-**S23** & α-**S24** (36:64)

^{1}H NMR (500 MHz, CDCl$_3$)

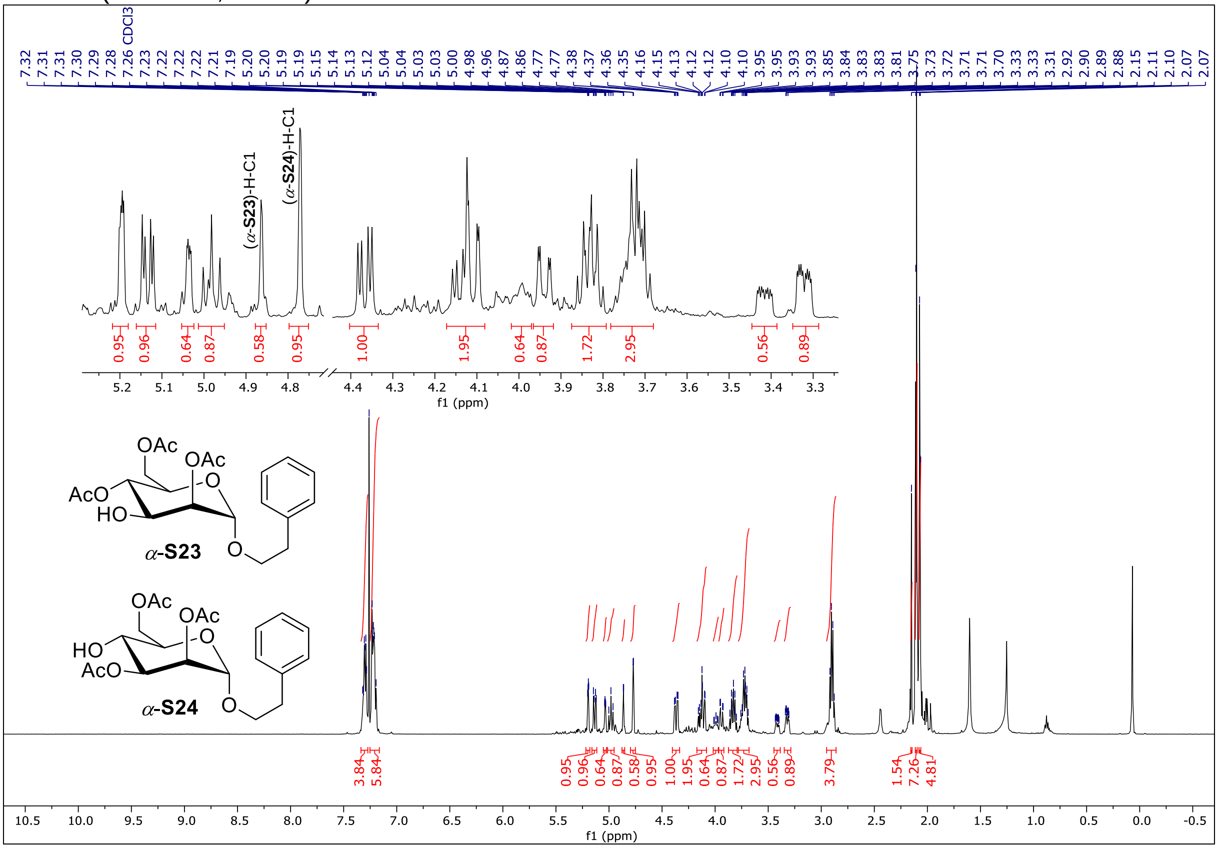

^{13}C NMR (126 MHz, CDCl$_3$)

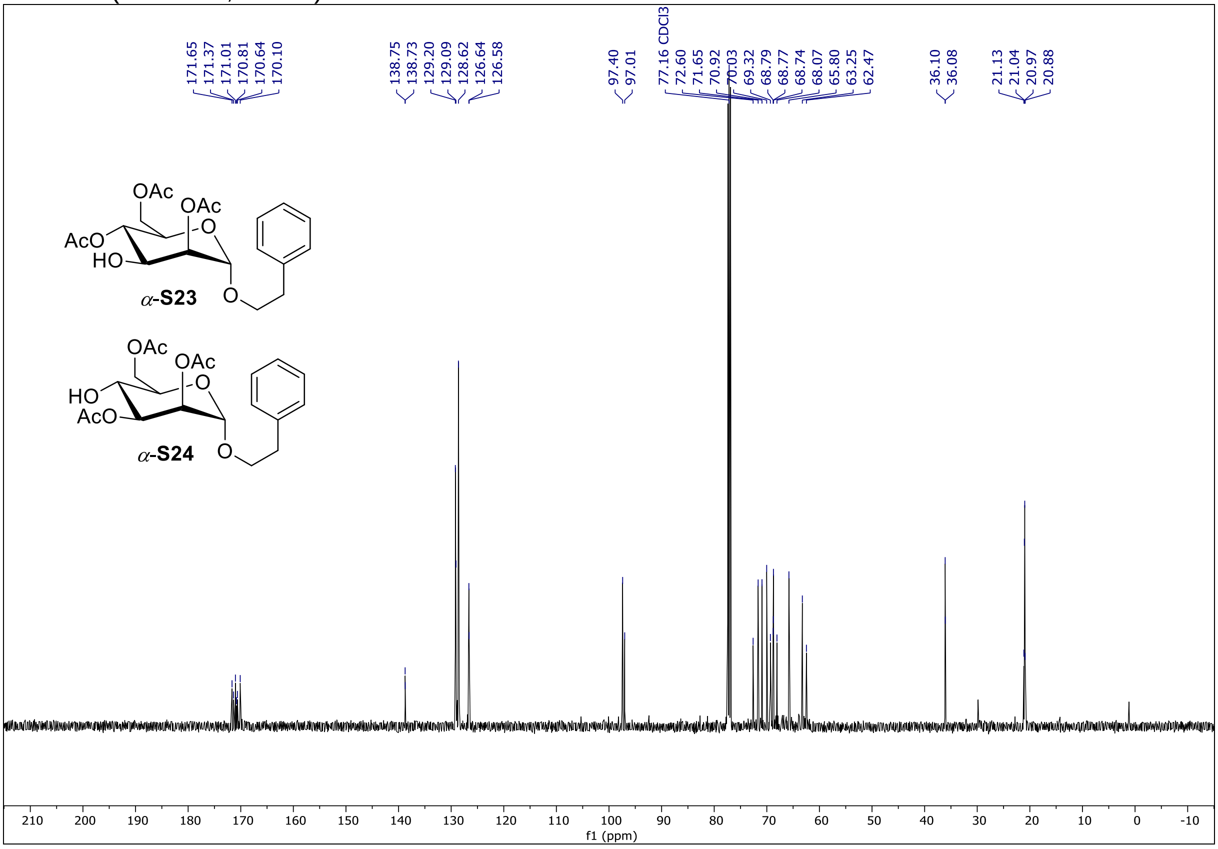

α-**S23** & α-**S24**

^{1}H-^{13}C HMBC ($CDCl_3$)

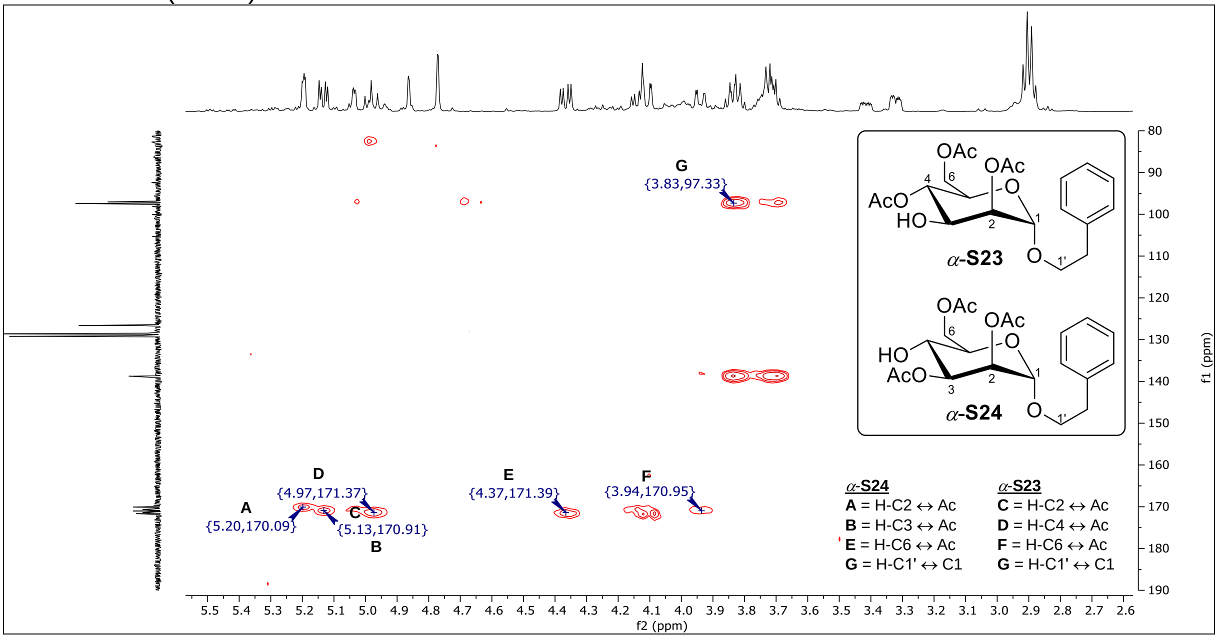

^{1}H NMR (500 MHz, $CDCl_3$)

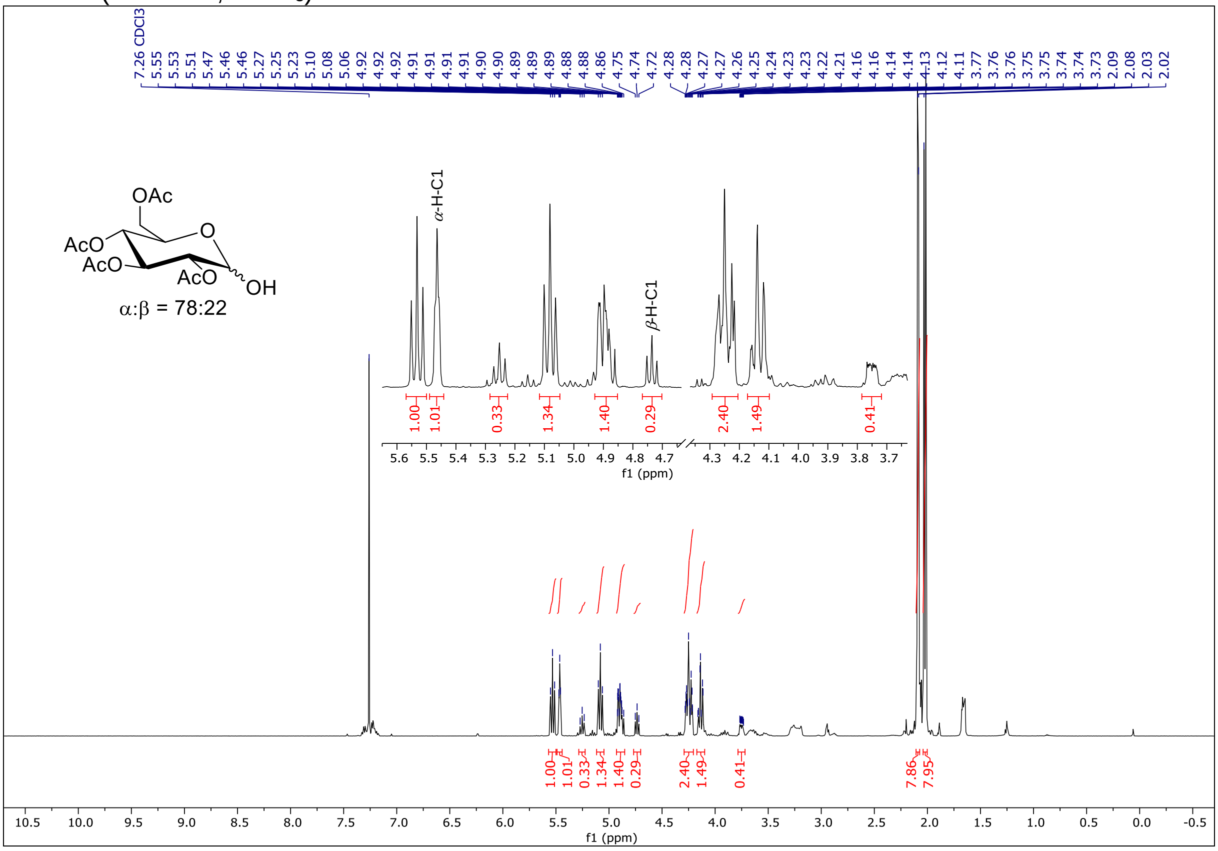

^{13}C NMR (126 MHz, $CDCl_3$)

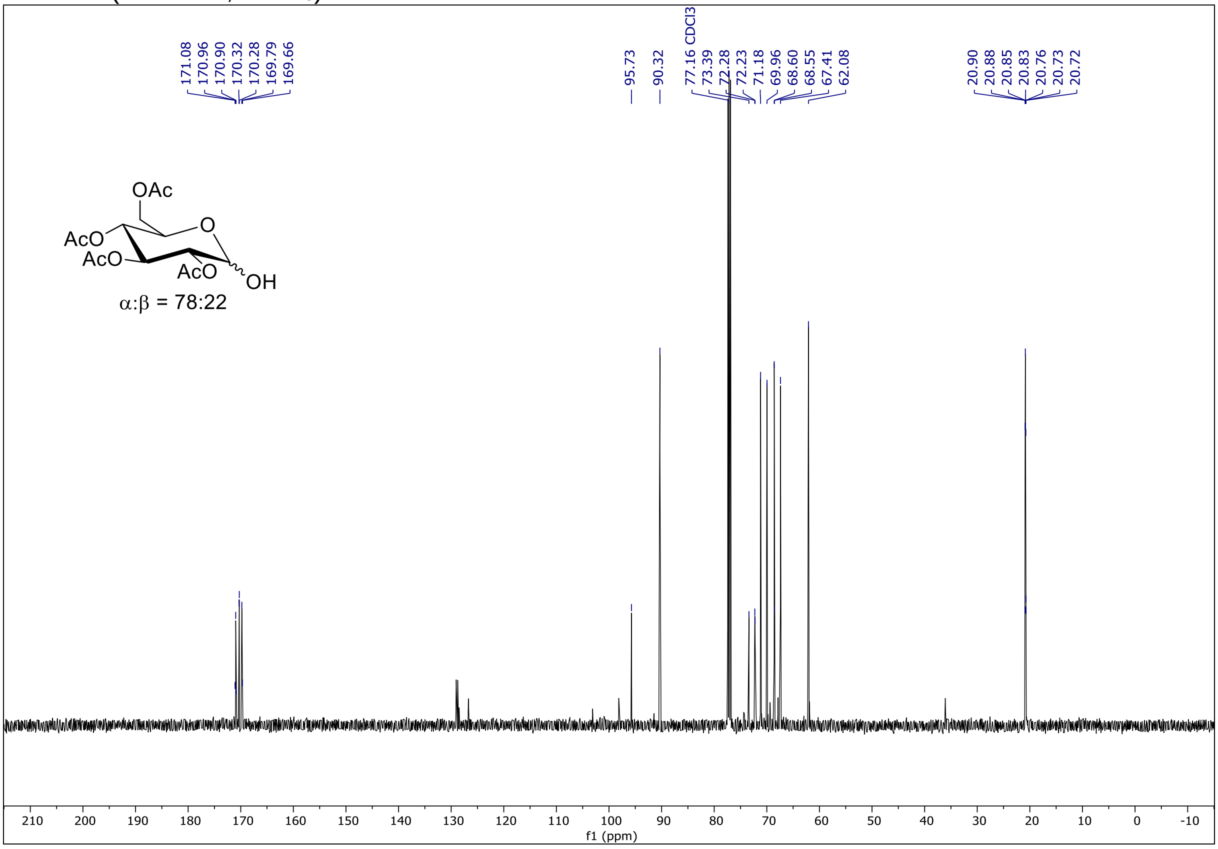

S26

^1H NMR (500 MHz, $CDCl_3$)

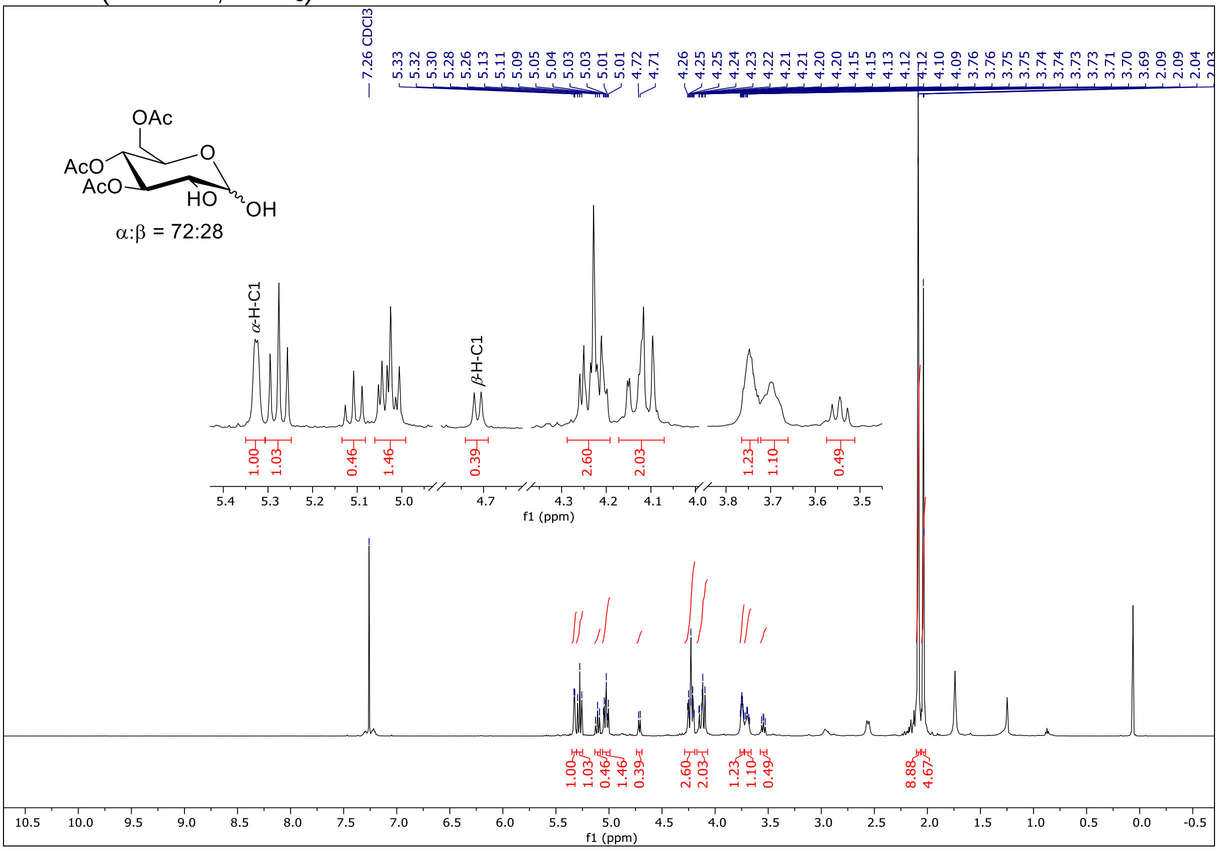

^{13}C NMR (126 MHz, $CDCl_3$)

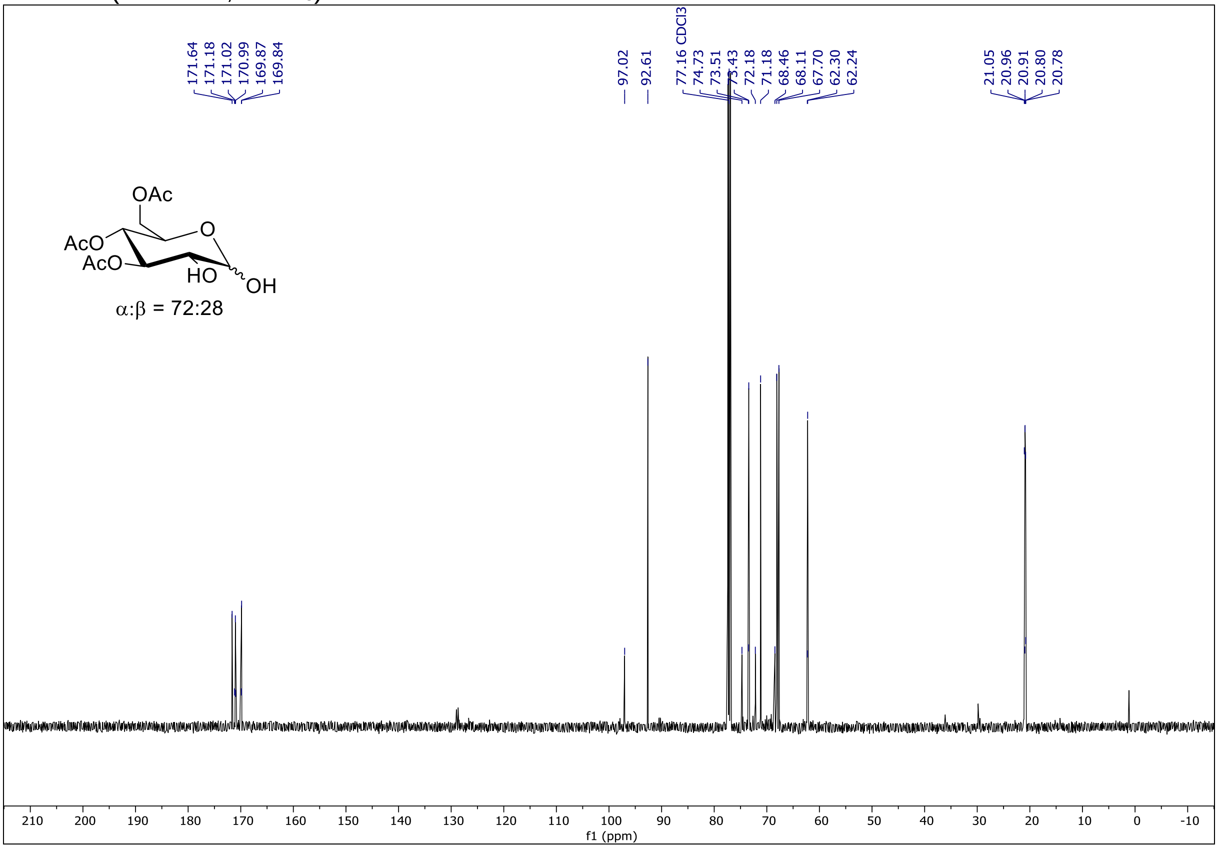

α-**S27**

^{1}H NMR (500 MHz, $CDCl_3$)

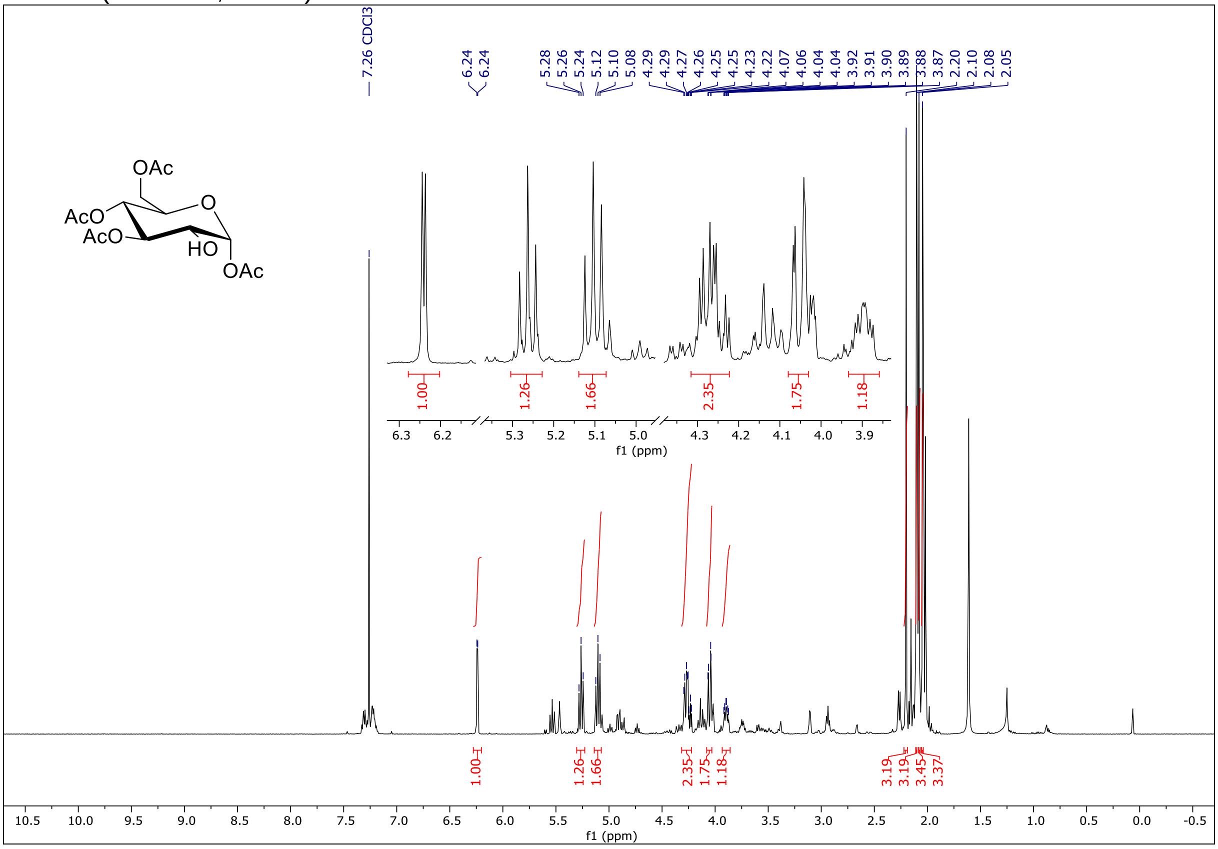

^{13}C NMR (126 MHz, $CDCl_3$)

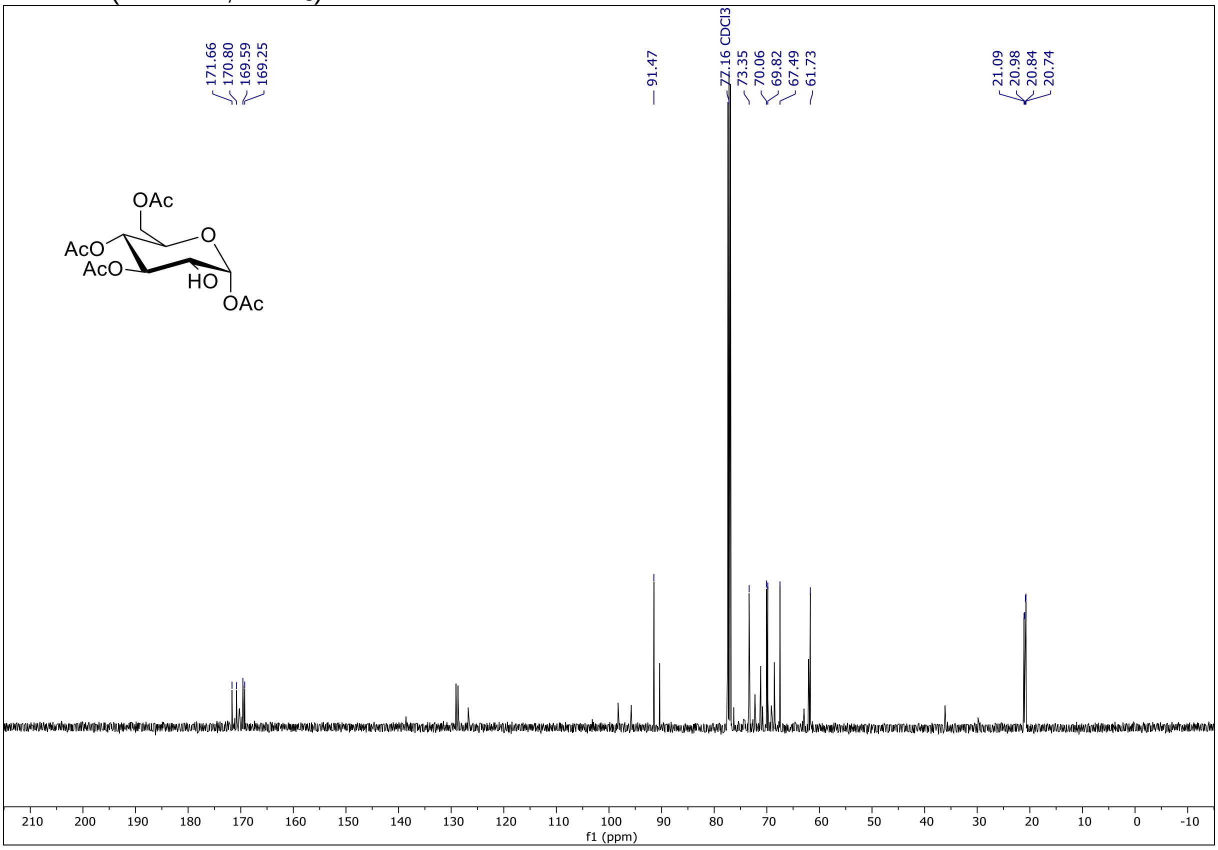

^{1}H NMR (500 MHz, $CDCl_3$)

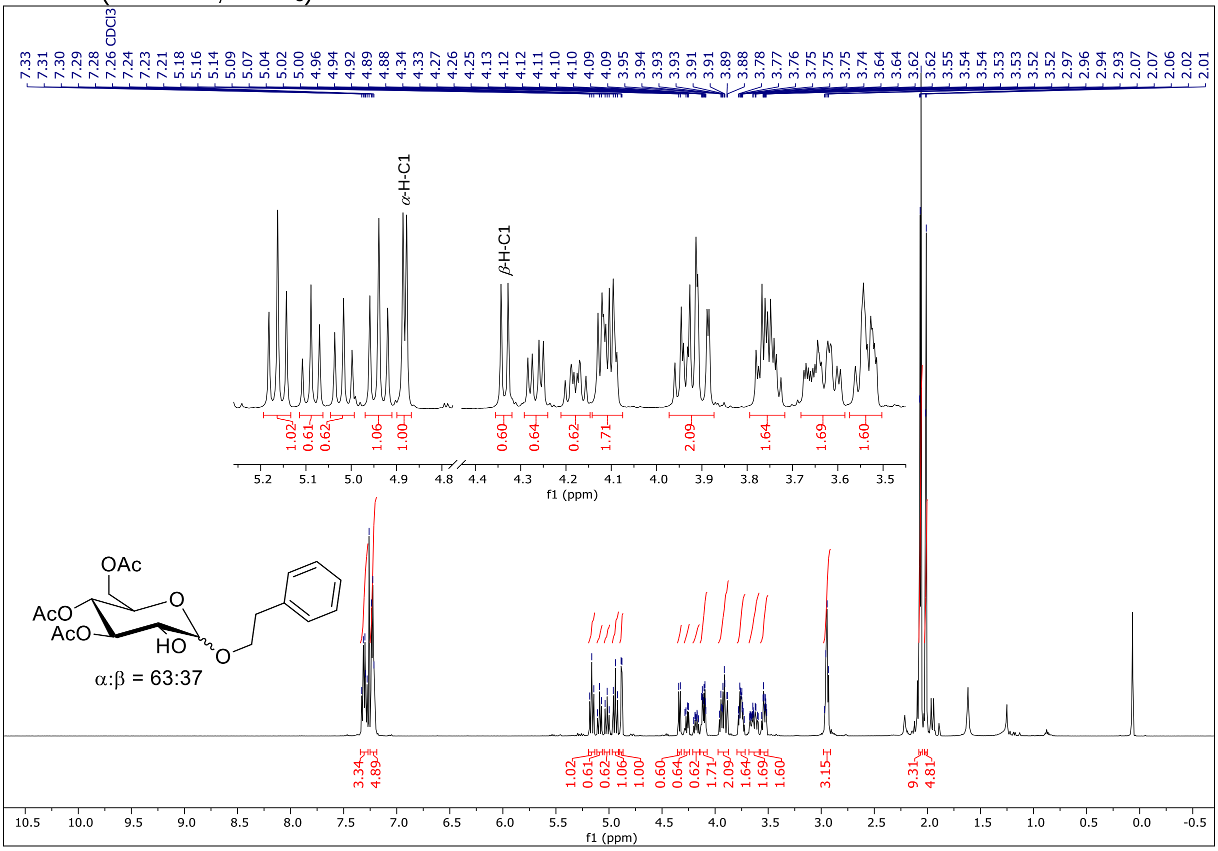

^{13}C NMR (126 MHz, $CDCl_3$)

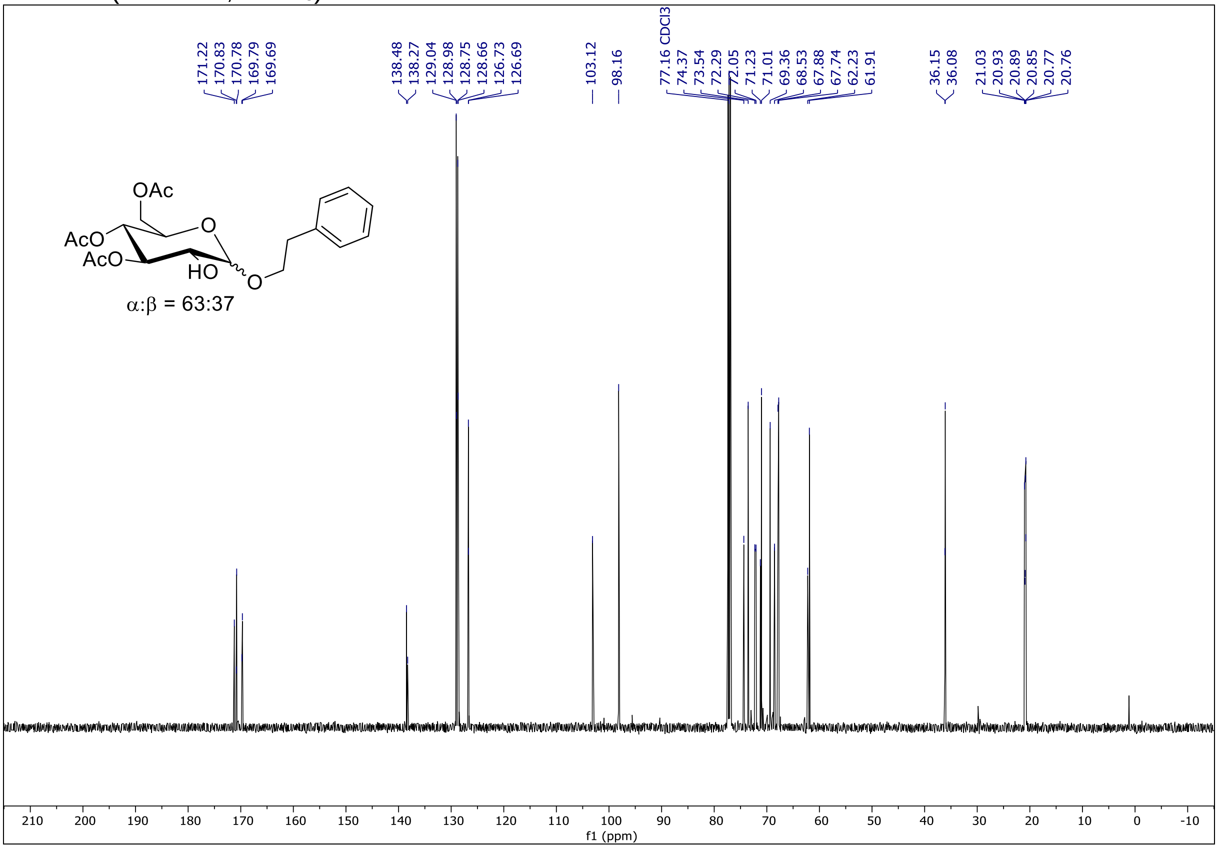

^{1}H-^{13}C HMBC ($CDCl_3$)

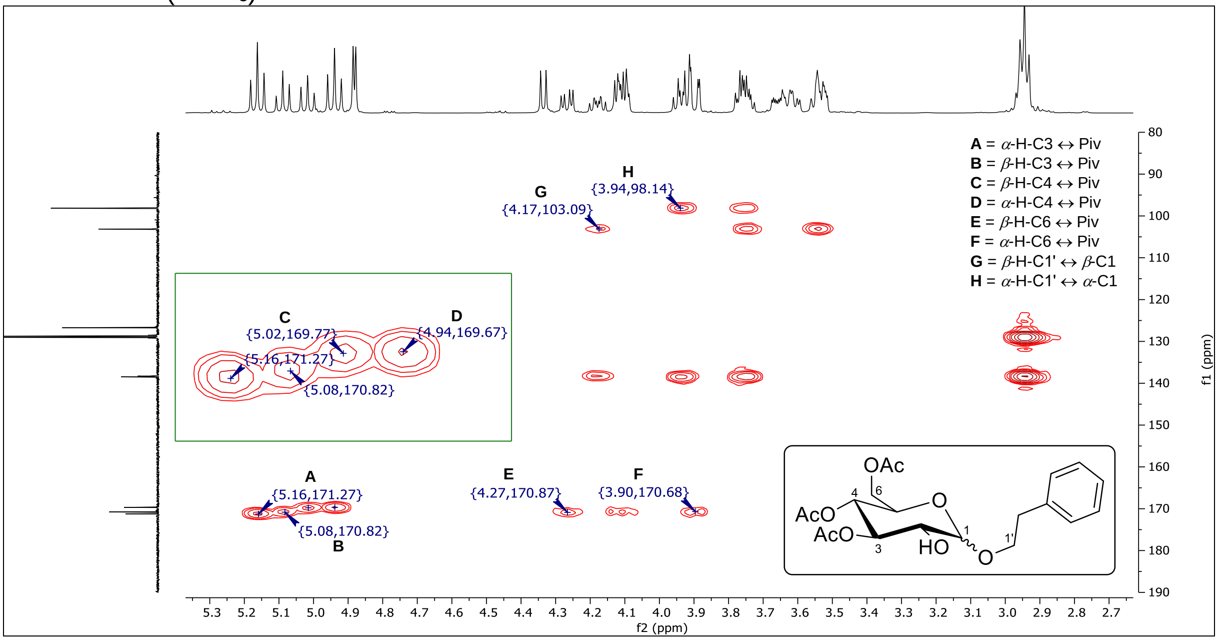

^{1}H NMR (500 MHz, $CDCl_3$)

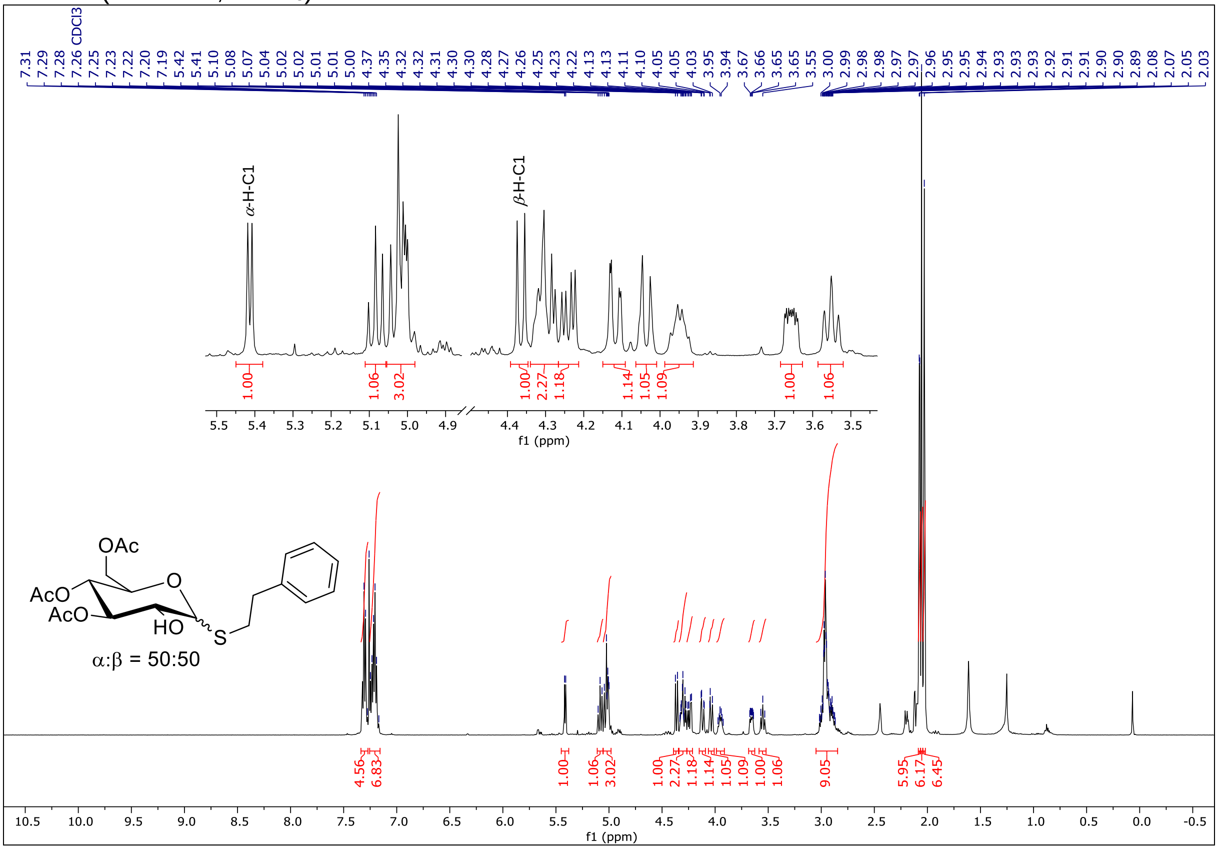

^{13}C NMR (126 MHz, $CDCl_3$)

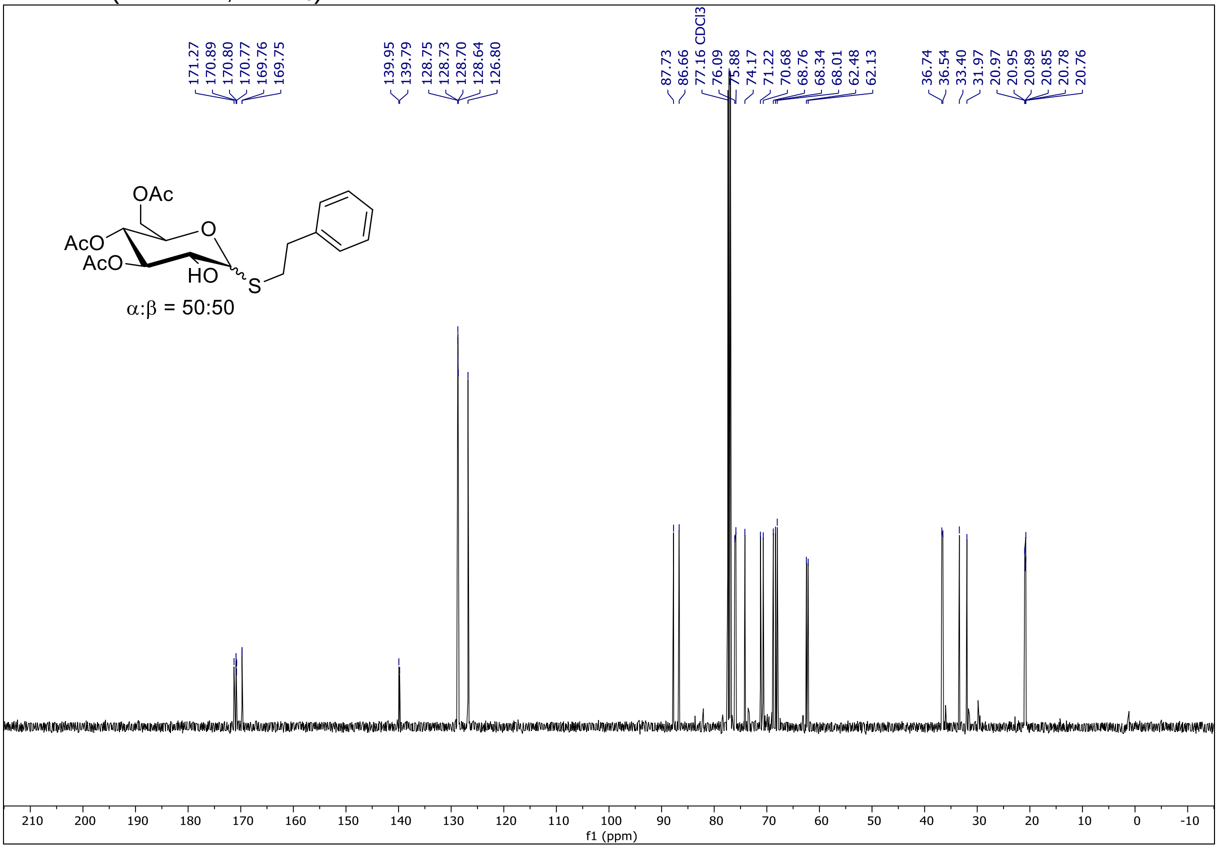

^{1}H-^{13}C HMBC ($CDCl_3$)

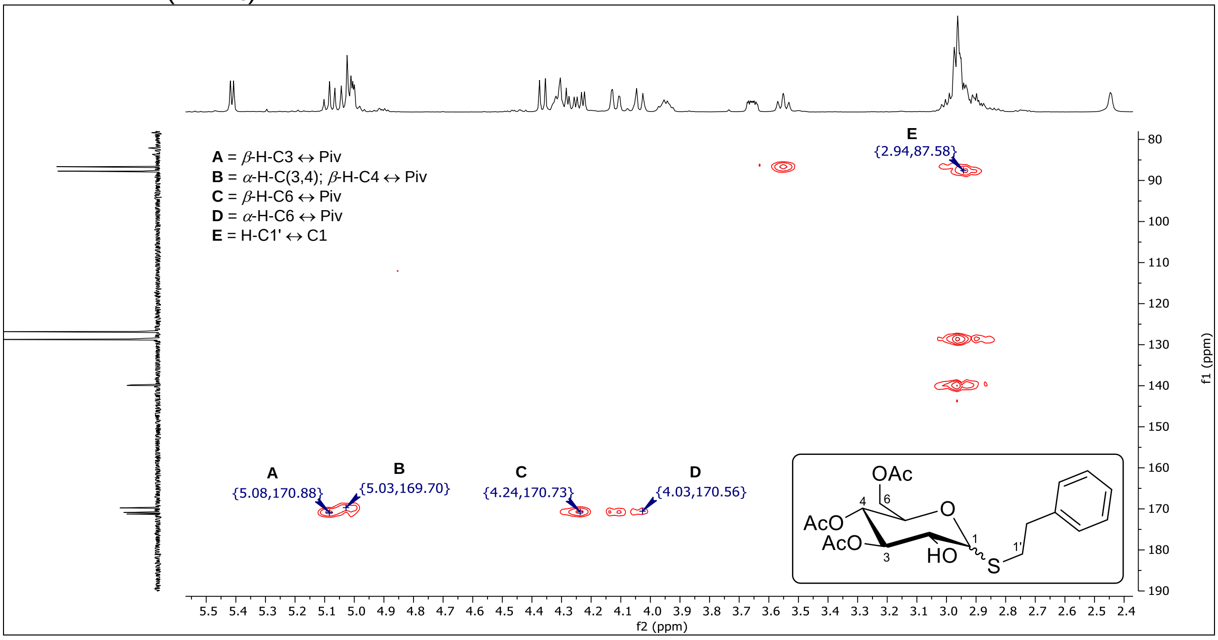

α-**S30**

^{1}H NMR (500 MHz, $CDCl_3$)

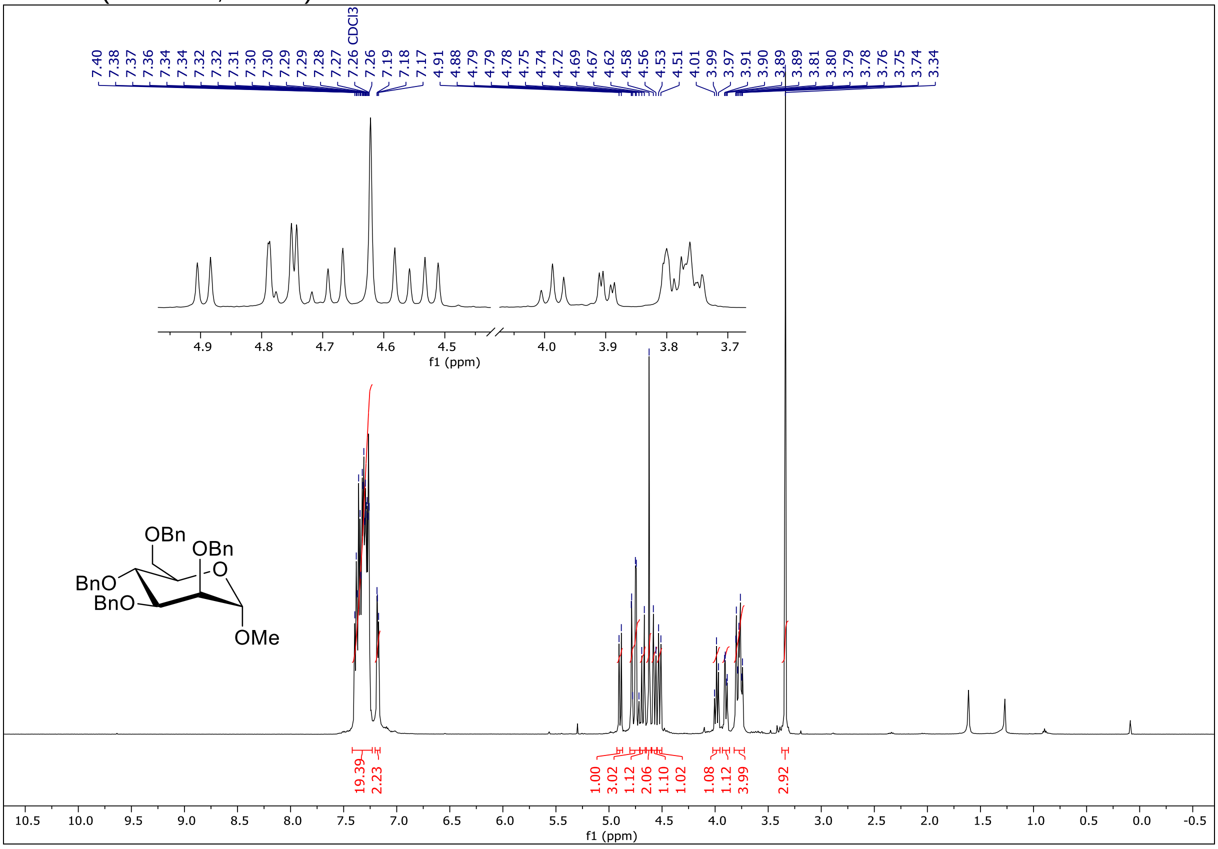

^{13}C NMR (126 MHz, $CDCl_3$)

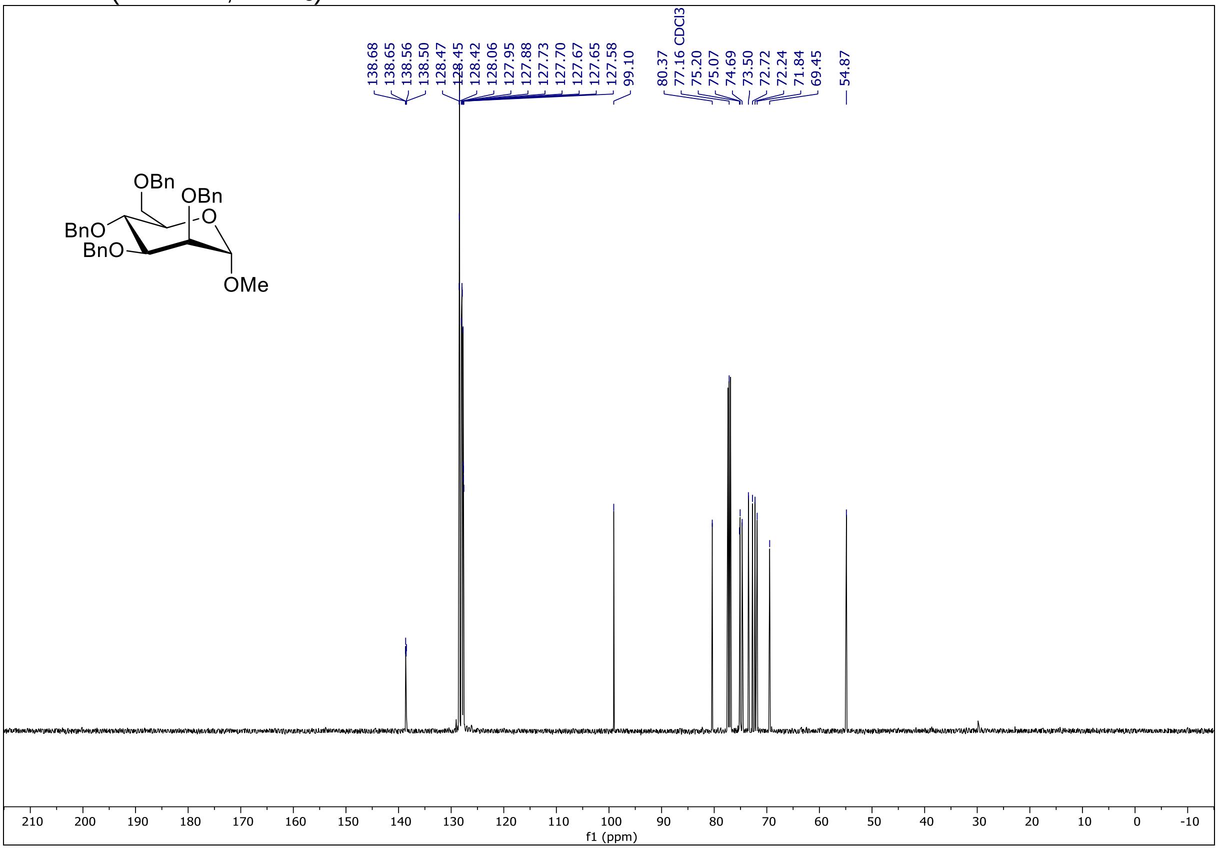

α-**S31**

^{1}H NMR (500 MHz, $CDCl_3$)

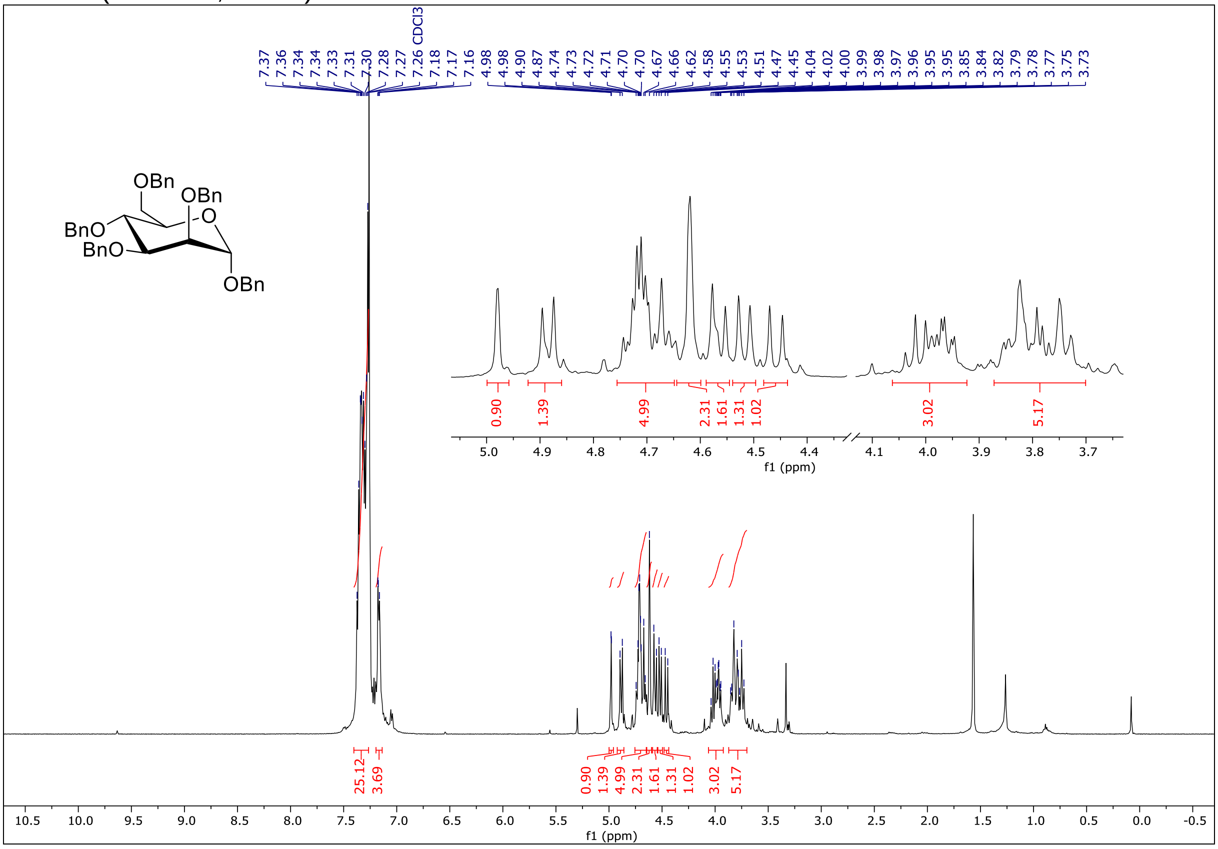

^{13}C NMR (126 MHz, $CDCl_3$)

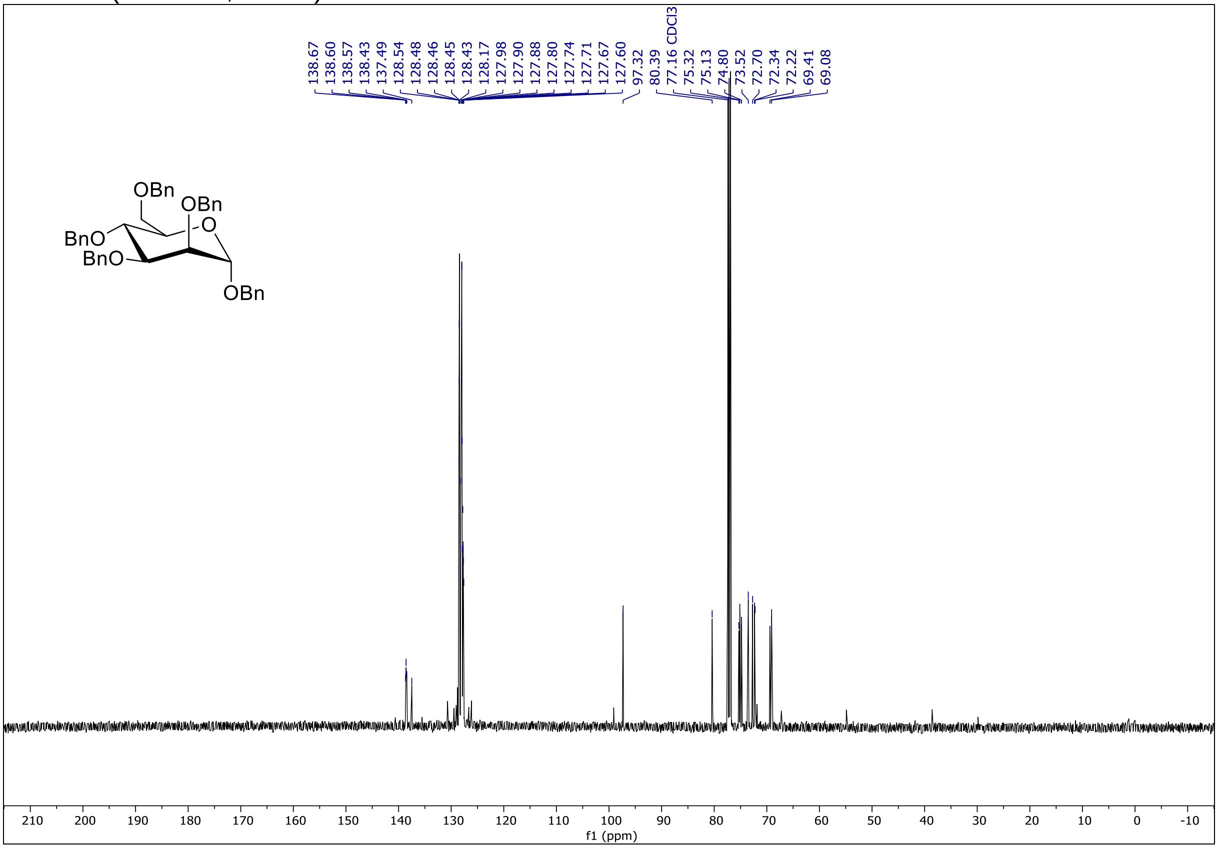

^{1}H NMR (500 MHz, $CDCl_3$)

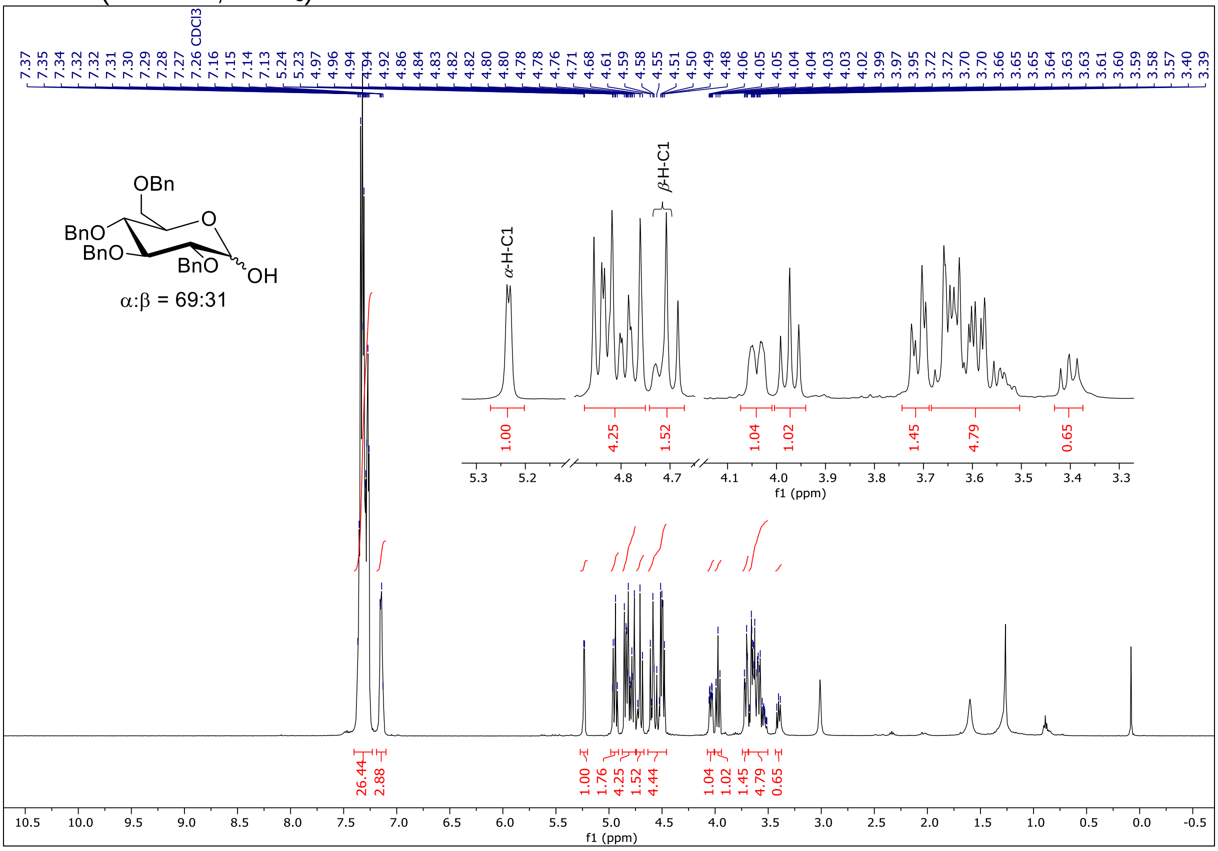

^{13}C NMR (126 MHz, $CDCl_3$)

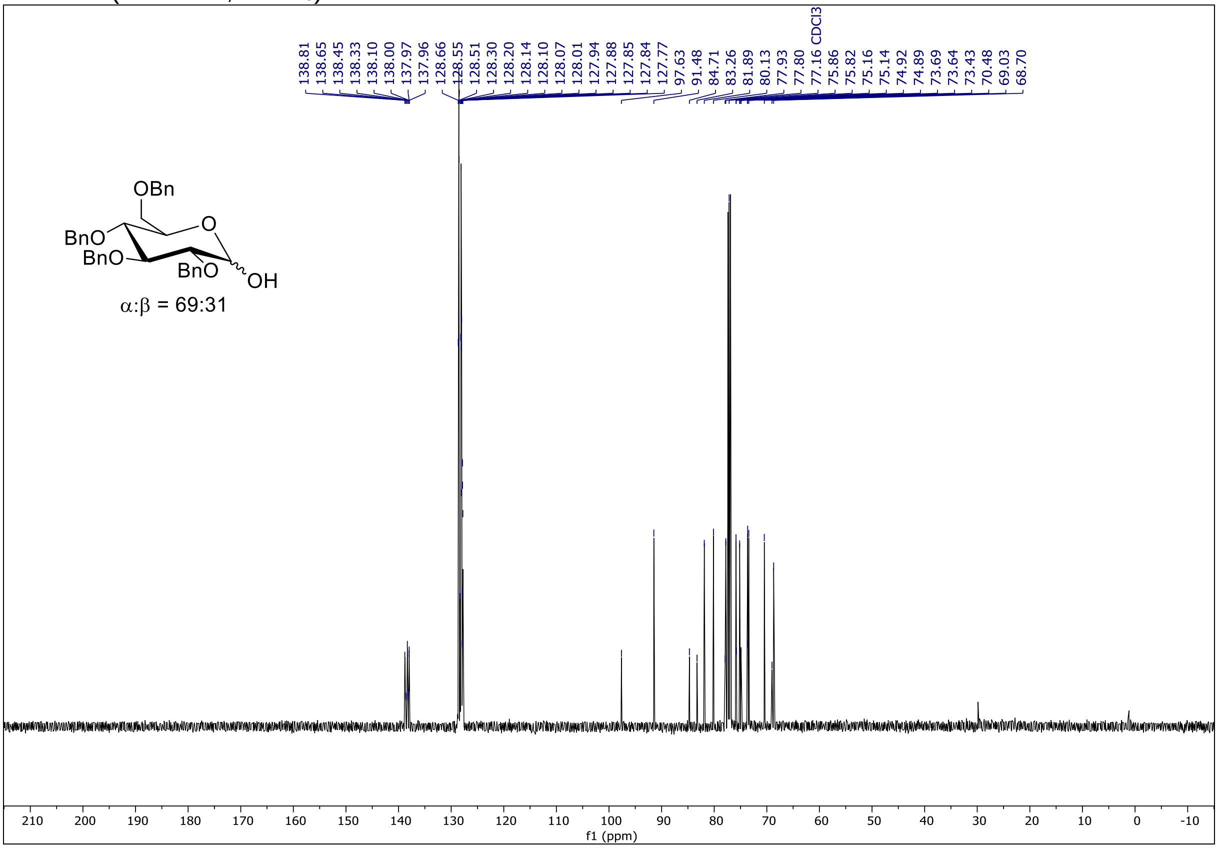

S33

^{1}H NMR (500 MHz, $CDCl_3$)

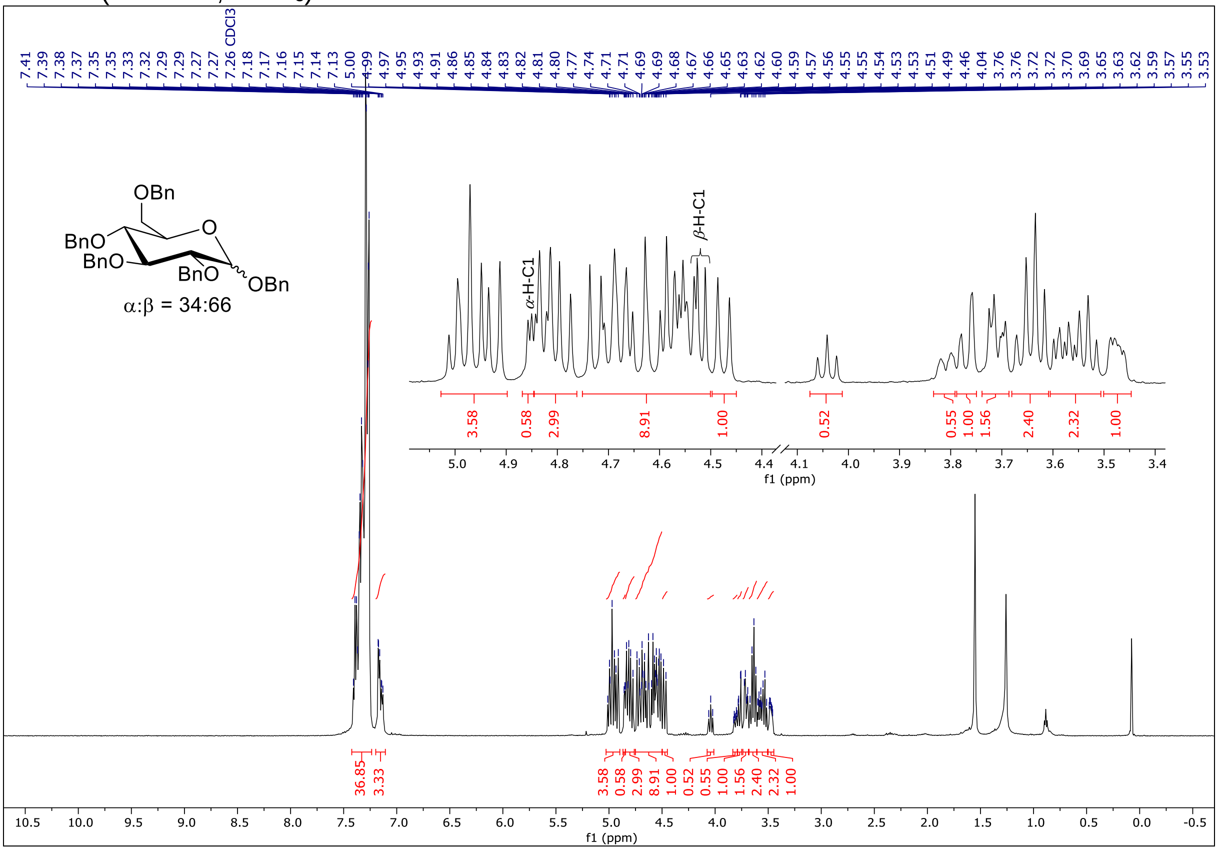

^{13}C NMR (126 MHz, $CDCl_3$)

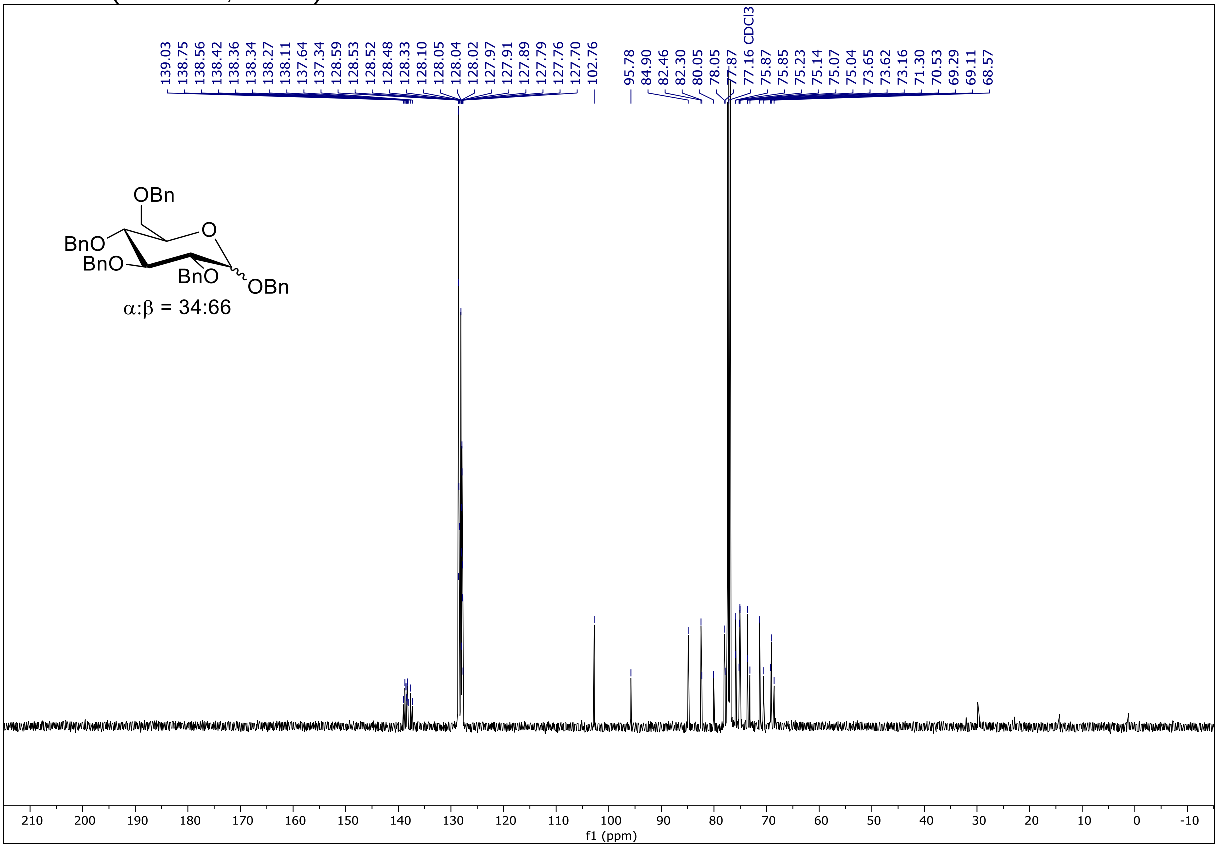

Supplement: File 2 — Copies of NMR spectra. [file Beilstein_J_Org_Chem-17-964-s002.pdf]
