# Supplementary material for: Metal-free glycosylation with glycosyl fluorides in liquid SO2
Source: Beilstein J Org Chem. 2021 Apr 29;17:964–76. doi: 10.3762/bjoc.17.78 (PMC8093551; doi:10.3762/bjoc.17.78)
Supplement: File 3 — DFT calculations. [file Beilstein_J_Org_Chem-17-964-s003.pdf]

BEILSTEIN JOURNAL OF ORGANIC CHEMISTRY

Supporting Information

for

Metal-free glycosylation with glycosyl fluorides in liquid SO_2

Krista Gulbe, Jevgeņija Lugiņina, Edijs Jansons, Artis Kinens and Māris Turks

Beilstein J. Org. Chem. **2021,** *17,* 964–976. doi:10.3762/bjoc.17.78

DFT calculations

License and Terms: This is a supporting information file under the terms of the Creative Commons Attribution License (https://creativecommons.org/licenses/by/4.0). Please note that the reuse, redistribution and reproduction in particular requires that the author(s) and source are credited and that individual graphics may be subject to special legal provisions.
The license is subject to the *Beilstein Journal of Organic Chemistry* terms and conditions: (https://www.beilstein-journals.org/bjoc/terms)

DFT calculations

All calculations were performed using Gaussian 09.[1] Transition state (TS) conformations were found by performing a relaxed potential energy surface (PES) scan for bond dissociation. Then, optimization of the TS geometries were performed using the Berny algorithm. After that, intrinsic reaction coordinates (IRC) were calculated for the TS to confirm that the saddle point connects the correct reactant and product on the PES. Optimizations of stationary points were performed without any restrictions using m052x[2] method and 6-31+g(d) basis set. For stationary points that had multiple conformations, lowest-energy conformation was identified by comparing energies of the structures optimized from different starting points. Stationary points were verified to be real minima (zero imaginary frequency) or transition states (one imaginary frequency) by performing frequency calculations at the same level of theory. For all calculations superfine integral grid was applied (Gaussian 09 keyword: integral=grid=superfine).[3]

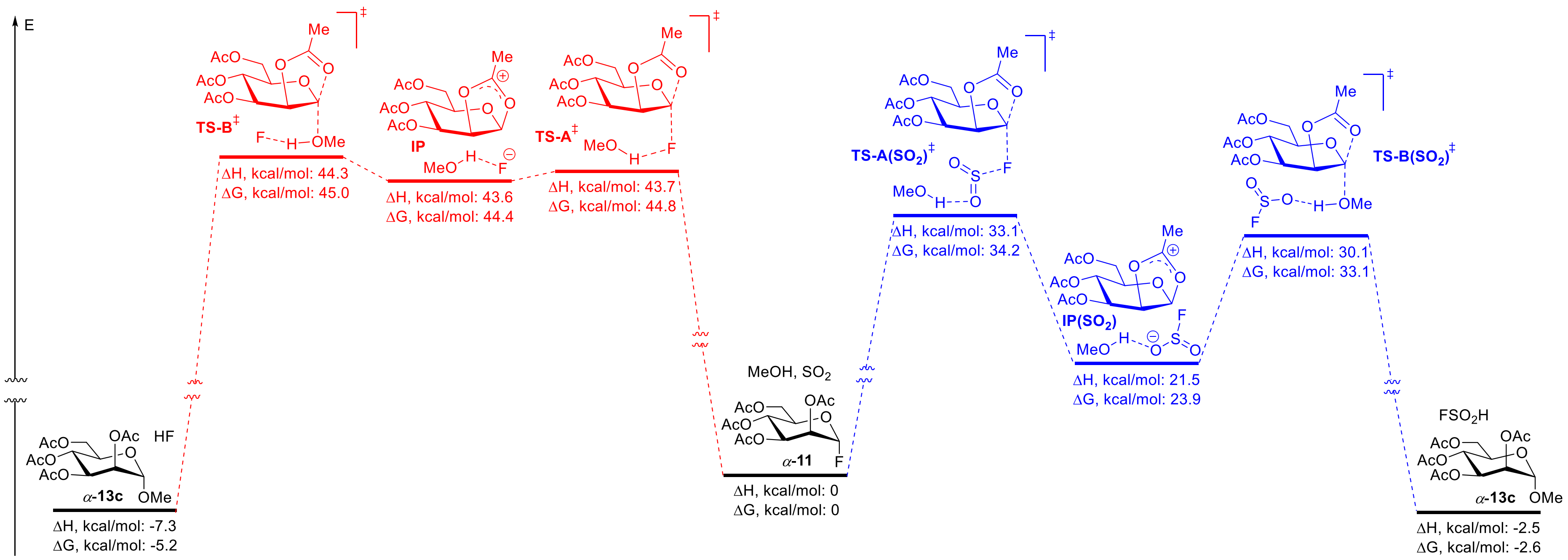

Figure: Computational study of reaction mechanism α-**11** + MeOH → α-**13c** in the presence of and in absence of SO_2 (Gaussian 09, Revision D.01; Gaussian, Inc.; m052x method and the 6-31+g(d) basis set). Gas phase enthalpy and Gibbs free energy values referenced against the starting value for the substrates and catalyst are in kcal/mol.

TS-A≠:	transition state A (conventional approach without SO_2)
TS-B≠:	transition state B (conventional approach without SO_2)
IP≠:	ion pair (conventional approach without SO_2)
TS-A(SO_2)≠:	transition state A for a reaction with participation of SO_2
TS-B(SO_2)≠:	transition state B for a reaction with participation of SO_2
IP(SO_2)≠:	ion pair for a reaction with participation of SO_2
α-**11**:	starting material: mannosyl fluoride
α-**13c**:	product methyl: mannoside

α-**11**

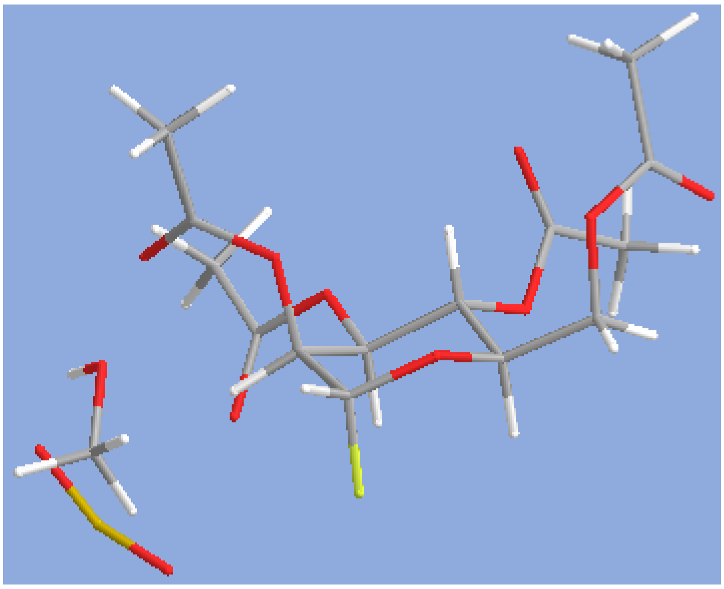

```
 Zero-point correction=                           0.407391 (Hartree/Particle)
 Thermal correction to Energy=                    0.441665
 Thermal correction to Enthalpy=                  0.442609
 Thermal correction to Gibbs Free Energy=         0.336622
 Sum of electronic and zero-point Energies=          -1985.603801
 Sum of electronic and thermal Energies=             -1985.569527
 Sum of electronic and thermal Enthalpies=           -1985.568583
 Sum of electronic and thermal Free Energies=        -1985.674570
```

```
0 1
 C                  -0.82395000   -0.63725100   -0.06944100
 C                   0.05487400    0.52597200   -0.51794700
 C                   1.51001100    0.10795500   -0.46723100
 C                   1.70251100   -1.12753100   -1.34441100
 H                  -1.87714800   -0.41073600   -0.20024300
 H                  -0.22824100    0.82281000   -1.52925800
 H                   1.80665600   -0.10512600    0.55750100
 H                   1.41425800   -0.89847100   -2.37540900
 O                   0.87612000   -2.18660300   -0.85056900
 C                  -0.47395400   -1.89810300   -0.85006500
 H                  -0.99452400   -2.76163600   -0.44821400
 F                  -0.90822100   -1.69181900   -2.15928200
 O                  -3.76257900    0.18062800   -2.11806700
 O                  -4.67518300    0.55809200    0.14663700
 H                  -3.82939900   -1.12924900    1.01665900
 C                  -4.04914100   -2.79545900    0.00949200
 H                  -3.59207900   -3.78328600    0.04837700
 H                  -5.11994100   -2.89960300    0.20410300
 H                  -3.90201400   -2.37162800   -0.98812800
 O                  -3.41482900   -2.00577000    1.00728300
 S                  -4.37613400    1.09695700   -1.17478400
 O                  -0.56068600   -0.96997800    1.29573200
 C                  -1.38709600   -0.44836300    2.22843500
 O                  -2.18378300    0.43009600    1.99193200
 C                  -1.19564000   -1.11818300    3.55380500
 H                  -1.74473500   -2.06176900    3.51873500
 H                  -0.14313400   -1.33521900    3.72841100
 H                  -1.60406900   -0.48995700    4.34100100
 O                  -0.09460100    1.64241600    0.36745700
 C                  -1.20226400    2.38626200    0.21904200
 O                  -1.99531000    2.20506000   -0.68378300
 C                  -1.32757900    3.43071200    1.28254500
 H                  -0.34739100    3.75695100    1.62483400
 H                  -1.91221100    4.26519100    0.90248400
```

H	-1.85797800	2.97000400	2.11872300
O	2.32494000	1.15170100	-1.00617300
C	3.14299500	1.81773800	-0.14881000
O	3.18675800	1.59156900	1.03556400
C	3.99185000	2.81106200	-0.88153000
H	3.43295100	3.28104900	-1.68887900
H	4.36550700	3.55088300	-0.17830700
H	4.83467800	2.26252200	-1.30827700
C	3.13473100	-1.61680800	-1.36585600
H	3.75205000	-0.94496900	-1.95684500
H	3.16023400	-2.62211200	-1.78321900
O	3.65697300	-1.69353600	-0.03276100
C	4.74569000	-0.94549900	0.25769700
O	5.31501400	-0.25274700	-0.55435900
C	5.11436500	-1.06236900	1.70576500
H	4.90279900	-2.06144600	2.08187900
H	6.16381300	-0.80705500	1.82997500
H	4.50496100	-0.34032000	2.25409600

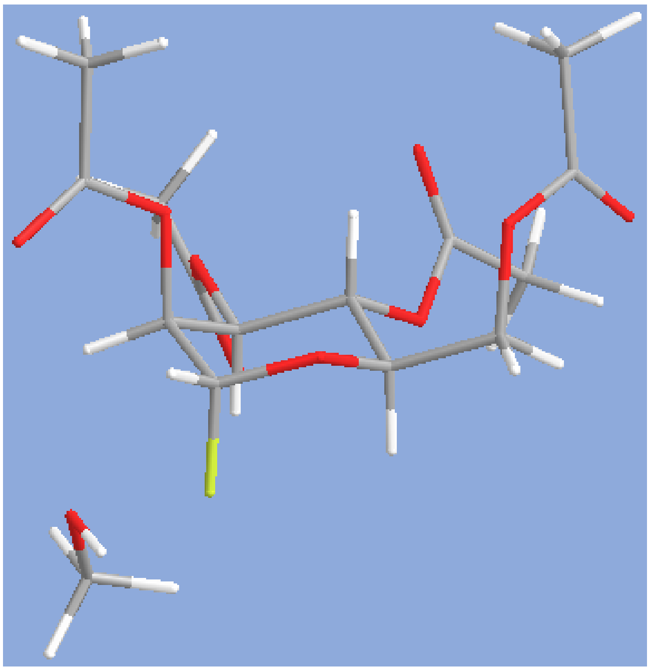

```
Zero-point correction=                           0.397984 (Hartree/Particle)
Thermal correction to Energy=                    0.428253
Thermal correction to Enthalpy=                  0.429197
Thermal correction to Gibbs Free Energy=         0.332015
Sum of electronic and zero-point Energies=          -1437.049066
Sum of electronic and thermal Energies=             -1437.018797
Sum of electronic and thermal Enthalpies=           -1437.017853
Sum of electronic and thermal Free Energies=        -1437.115036
```

0 1

C	-1.68133000	-0.89542600	-0.04303800
C	-0.88917300	0.39950600	-0.07587300
C	0.57403900	0.09484800	-0.35027400
C	0.69389500	-0.70439700	-1.64668000
H	-2.74350400	-0.69207000	0.07516300
H	-1.29625000	1.05198700	-0.84663700
H	1.00333500	-0.45091300	0.48743100
H	0.32898400	-0.09674000	-2.48048700
O	-0.09016500	-1.90129500	-1.56776000
C	-1.42770300	-1.69956400	-1.31492300
H	-1.90977900	-2.67076000	-1.28996500
F	-2.00582000	-0.97399000	-2.37326700
H	-3.54904400	0.62584600	-2.04419700
C	-3.78365400	2.54481400	-1.66371000
H	-3.95759600	3.12087100	-0.75835000

H	-2.84635100	2.88444300	-2.11132100
H	-4.61018700	2.71299500	-2.35897500
O	-3.70908800	1.17823800	-1.26920400
O	-1.19142400	-1.66347700	1.06444400
C	-2.00797900	-2.63346800	1.53465100
O	-3.06741600	-2.90394600	1.02329500
C	-1.41209200	-3.29865700	2.74066200
H	-0.44179400	-3.72287200	2.47943500
H	-1.25107500	-2.55572000	3.52265600
H	-2.08458100	-4.07749800	3.08908000
O	-1.04430300	1.02023900	1.20101400
C	-1.08180200	2.37111700	1.24626000
O	-1.03131400	3.07744800	0.26815500
C	-1.16209900	2.84473100	2.66824800
H	-0.22327900	2.59441600	3.16643800
H	-1.31876700	3.91985300	2.68233100
H	-1.96830500	2.32938300	3.18998700
O	1.30139800	1.30384600	-0.57886400
C	2.04349100	1.80550300	0.43759800
O	2.09929300	1.29240900	1.53060100
C	2.75954400	3.04685300	0.00457100
H	2.04706700	3.74281400	-0.43906700
H	3.25544200	3.49323200	0.86208800
H	3.49743300	2.76975400	-0.75014300
C	2.12475700	-1.10244100	-1.95260600
H	2.68611600	-0.23999600	-2.30255500
H	2.12370500	-1.89006900	-2.70367700
O	2.75822400	-1.63725300	-0.78210300
C	3.76999800	-0.92160200	-0.23759000
O	4.21642900	0.08740400	-0.73137800
C	4.21925600	-1.51687500	1.06333600
H	4.09613700	-2.59795200	1.06299400
H	5.25332600	-1.23615700	1.24788300
H	3.59339700	-1.08749300	1.84988600

TS-A(SO_2)‡

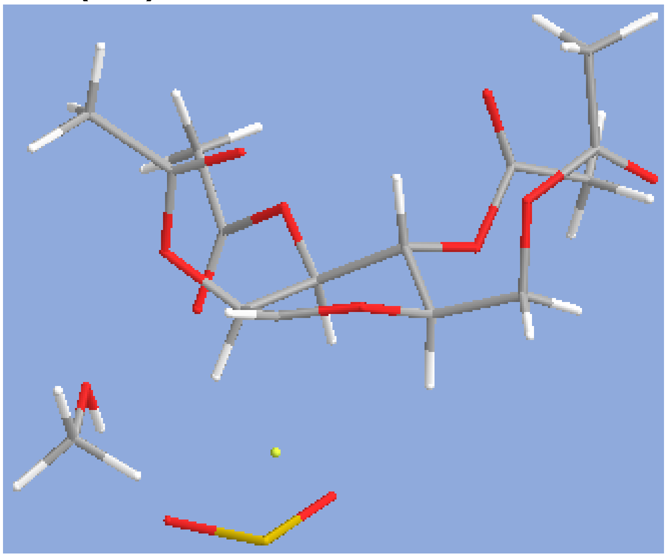

```
Zero-point correction=                           0.405649 (Hartree/Particle)
Thermal correction to Energy=                    0.439382
Thermal correction to Enthalpy=                  0.440327
Thermal correction to Gibbs Free Energy=         0.336095
Sum of electronic and zero-point Energies=          -1985.550556
Sum of electronic and thermal Energies=             -1985.516823
Sum of electronic and thermal Enthalpies=           -1985.515879
Sum of electronic and thermal Free Energies=        -1985.620111
```

0 1

C	-1.29819800	0.38467000	0.07597800
C	-0.00963200	0.82061800	-0.68245700
C	1.20908600	0.01239600	-0.28847800
C	0.83232700	-1.46135000	-0.28006900
H	-2.09932200	0.26132400	-0.64460200
H	-0.20334500	0.68150000	-1.74596300
H	1.58882400	0.31493300	0.68566700
H	0.30120300	-1.74616200	-1.19284200
O	-0.11046500	-1.64713300	0.82304300
C	-1.14216000	-0.90412500	0.86720900
H	-1.83961800	-1.09028700	1.66961700
F	-2.30048000	-2.17384400	-0.20076700
O	-1.61919500	-1.29623100	-2.46540900
O	-3.86038800	-0.74455200	-1.58458200
H	-4.13407000	-0.34251300	0.37825200
C	-4.83288100	-1.56134500	1.72603400
H	-4.77495600	-1.63727000	2.81108800
H	-5.88272400	-1.46412700	1.43797100
H	-4.41544800	-2.46693200	1.27839200
O	-4.08571800	-0.41212500	1.34674300
S	-2.91188600	-1.81075300	-1.96853500
O	-1.78292600	1.40802100	0.94394100
C	-1.14280800	1.53804900	2.11770100
O	-0.26708000	0.76680600	2.44692600
C	-1.67729000	2.67793100	2.92369800
H	-1.67121200	3.58997800	2.32669300
H	-2.71446900	2.45780600	3.18337600
H	-1.07848300	2.79873500	3.82169500
O	0.30560300	2.18397900	-0.42194100
C	-0.46116700	3.09750200	-1.07742700
O	-1.36946600	2.78107000	-1.79966000
C	-0.00169500	4.49465000	-0.77879600
H	0.99199300	4.64420600	-1.20432400
H	-0.70307300	5.20151300	-1.21308000
H	0.07717600	4.64082400	0.29873200
O	2.21100100	0.18889600	-1.28831400
C	3.36129700	0.82475200	-0.92604700
O	3.55831300	1.24484300	0.18643200
C	4.32270800	0.87776600	-2.07253700
H	3.79947300	1.05773200	-3.01007400
H	5.06516200	1.64802900	-1.88084700
H	4.81776000	-0.09439600	-2.12691200
C	1.98696600	-2.39886300	-0.03556300
H	2.63130000	-2.41732000	-0.91171000
H	1.60755000	-3.39831100	0.17261500
O	2.72562000	-1.96412700	1.10846700
C	4.04125100	-1.67930900	0.93720700
O	4.61031800	-1.80559600	-0.12120600
C	4.65115800	-1.16694400	2.20506800
H	4.25172400	-1.69716200	3.06775400
H	5.73210300	-1.26441000	2.14706200
H	4.38822400	-0.11024100	2.29026100

IP(SO_2)

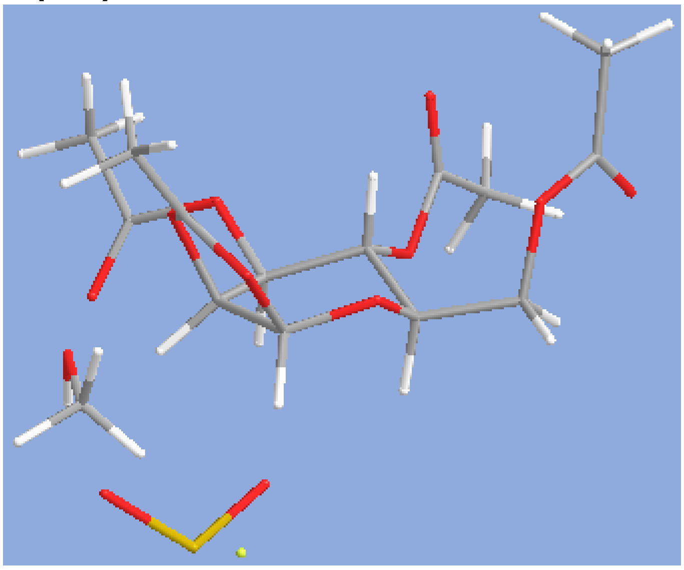

```
 Zero-point correction=                           0.408861 (Hartree/Particle)
 Thermal correction to Energy=                    0.441903
 Thermal correction to Enthalpy=                  0.442847
 Thermal correction to Gibbs Free Energy=         0.340620
 Sum of electronic and zero-point Energies=          -1985.568312
 Sum of electronic and thermal Energies=             -1985.535270
 Sum of electronic and thermal Enthalpies=           -1985.534326
 Sum of electronic and thermal Free Energies=        -1985.636553
```

```
0 1
 C                 -1.11411500    0.35877900    0.27145700
 C                  0.13710400    1.01599600   -0.31919500
 C                  1.37152100    0.14228300   -0.21666200
 C                  1.01651400   -1.27488800   -0.64921000
 H                 -2.00744700    0.73741100   -0.21272600
 H                 -0.09157200    1.20610400   -1.36783300
 H                  1.76108600    0.12612700    0.80041500
 H                  0.51175300   -1.27459200   -1.62012300
 O                  0.12376200   -1.78217700    0.35822300
 C                 -1.12211300   -1.18162500    0.34242800
 H                 -1.81775300   -1.64869400   -0.34430200
 F                 -3.28051400   -2.05830900   -1.89822200
 O                 -1.55825400   -0.37053100   -2.36113100
 O                 -3.75276500    0.31909200   -1.48855200
 H                 -4.01913600    0.24636300    0.28578600
 C                 -4.74780200   -1.27162000    1.26095300
 H                 -4.56796900   -1.73790900    2.23106600
 H                 -5.81741400   -1.07797700    1.15689100
 H                 -4.42550800   -1.95051500    0.46885800
 O                 -4.00950500   -0.05202400    1.22076000
 S                 -3.04031600   -0.47080200   -2.54334700
 O                 -1.26125900    0.72123000    1.70107300
 C                 -1.70811100   -0.30412300    2.31858000
 O                 -1.64980500   -1.42223300    1.69885700
 C                 -2.21008300   -0.20817200    3.70007000
 H                 -1.62238300    0.52209400    4.25300100
 H                 -3.24048600    0.14766200    3.62106200
 H                 -2.18498600   -1.18451900    4.17632700
 O                  0.45123300    2.23960900    0.34902900
 C                 -0.36995600    3.29146900    0.09948500
 O                 -1.35412800    3.19566200   -0.58760700
 C                  0.11946700    4.53051700    0.79018800
 H                  1.12522200    4.76947800    0.44272200
 H                 -0.56097600    5.34989400    0.57638500
 H                  0.17413200    4.35176700    1.86469800
 O                  2.36009400    0.66769800   -1.10274000
```

C	3.49553400	1.17762300	-0.55612100
O	3.69399200	1.22315100	0.63366800
C	4.45054900	1.61818700	-1.62217700
H	3.91851600	2.06883500	-2.45819800
H	5.17124000	2.30996400	-1.19381300
H	4.97322900	0.72710000	-1.97711800
C	2.21079300	-2.19628800	-0.71808800
H	2.84021500	-1.92731300	-1.56286100
H	1.86712600	-3.22488100	-0.81455800
O	2.96903300	-2.10704800	0.49649400
C	4.27416400	-1.76385300	0.39882300
O	4.82799800	-1.54538700	-0.65364000
C	4.91059900	-1.65813800	1.75181700
H	4.51019100	-2.41052000	2.42886100
H	5.98840800	-1.75512700	1.64842200
H	4.67664600	-0.66635000	2.14500500

TS-B(SO_2)‡

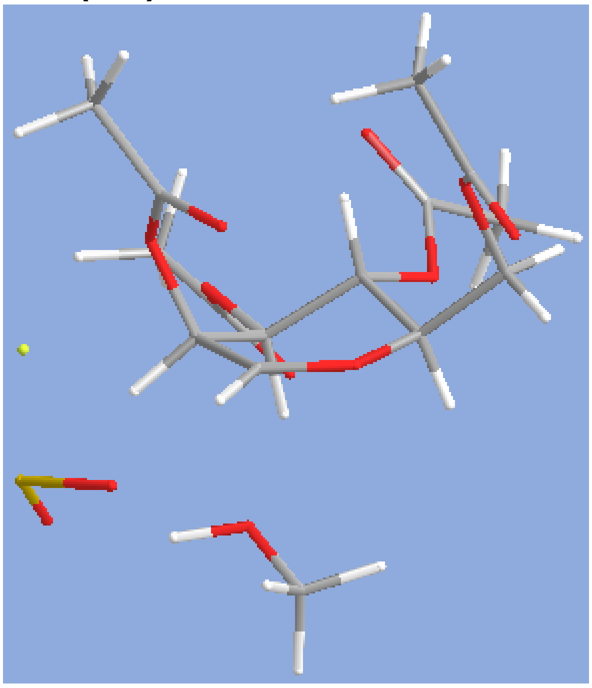

```
Zero-point correction=                           0.406840 (Hartree/Particle)
Thermal correction to Energy=                    0.439562
Thermal correction to Enthalpy=                  0.440506
Thermal correction to Gibbs Free Energy=         0.339289
Sum of electronic and zero-point Energies=          -1985.554340
Sum of electronic and thermal Energies=             -1985.521618
Sum of electronic and thermal Enthalpies=           -1985.520674
Sum of electronic and thermal Free Energies=        -1985.621891
```

0 1

C	0.55275800	-0.85499100	0.24498100
C	-0.86650100	-1.10259100	-0.25206100
C	-1.59129800	0.23442100	-0.32038800
C	-0.89290300	1.12178900	-1.35112100
H	1.17809600	-1.73599300	0.12400300
H	-0.83254700	-1.56240100	-1.24025400
H	-1.63983800	0.70241900	0.66374100
H	-1.07736100	0.70266700	-2.34243900
O	0.54804000	1.16077700	-1.20147400
C	1.21178700	0.29617100	-0.51619000
H	2.28946500	0.38344000	-0.57332900
F	3.22201800	-1.13782700	1.41534500
O	4.22599000	-0.30915500	-0.68689600
O	3.16680500	-2.51910900	-0.62862700

H	2.14232300	-1.75100400	-1.75302500
C	2.12122600	-0.45642300	-3.25576600
H	3.08028000	-0.05129600	-2.92935400
H	2.26670900	-1.14149300	-4.09217600
H	1.46407300	0.35416600	-3.56758000
O	1.47964800	-1.14256500	-2.17731300
S	4.17398300	-1.61168800	0.02518900
O	0.50464800	-0.61460100	1.66267400
C	1.08299500	0.49946800	2.08829100
O	1.43384100	1.35966100	1.29563800
C	1.25127600	0.56042300	3.56755300
H	2.13850700	-0.03175100	3.80434400
H	1.40701000	1.58962300	3.87939300
H	0.39228500	0.11729100	4.06774800
O	-1.45282500	-1.99661200	0.68696300
C	-2.43463300	-2.82406100	0.25462200
O	-2.83475400	-2.83678000	-0.88404300
C	-2.94953000	-3.66181000	1.38536100
H	-2.11960400	-4.11519700	1.92640300
H	-3.48629200	-3.00648000	2.07447000
H	-3.61896800	-4.42273300	0.99379900
O	-2.90475600	0.10712300	-0.86788400
C	-3.93353800	-0.15463300	-0.01871100
O	-3.79620800	-0.22134600	1.17679300
C	-5.20420600	-0.36872000	-0.78316600
H	-5.34216600	0.41961000	-1.52271600
H	-5.11897400	-1.31999400	-1.31253200
H	-6.04017900	-0.39911900	-0.08982900
C	-1.35046100	2.56455500	-1.31013400
H	-2.42815900	2.60710600	-1.45850300
H	-0.83900000	3.14590300	-2.07429800
O	-1.05919100	3.09917400	-0.01941200
C	-0.12903300	4.09584900	0.04411600
O	0.38464400	4.57885400	-0.93116800
C	0.15338000	4.47660400	1.46625900
H	-0.75393600	4.42957600	2.06644100
H	0.87509500	3.76004900	1.86280900
H	0.58633300	5.47338700	1.48787800

α-13c

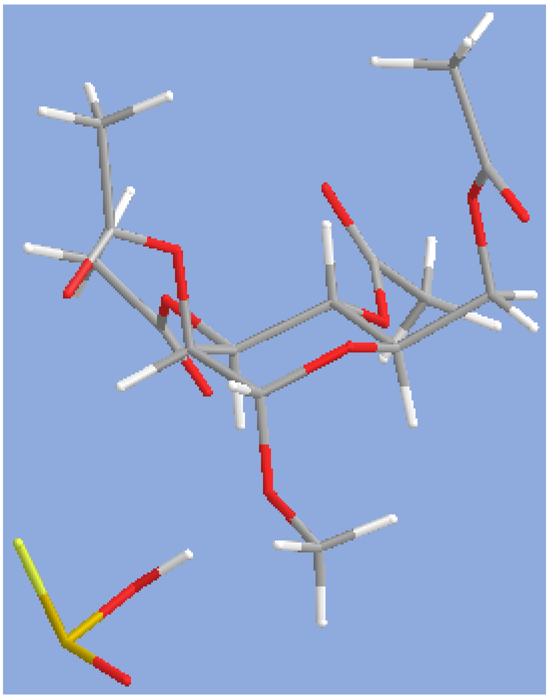

Zero-point correction= 0.408028 (Hartree/Particle)
Thermal correction to Energy= 0.441143

```
 Thermal correction to Enthalpy=                 0.442087
 Thermal correction to Gibbs Free Energy=        0.335985
 Sum of electronic and zero-point Energies=         -1985.606595
 Sum of electronic and thermal Energies=            -1985.573480
 Sum of electronic and thermal Enthalpies=          -1985.572536
 Sum of electronic and thermal Free Energies=       -1985.678638
```

0 1			
C	-0.67150600	-0.24272100	0.74967200
C	-0.02185600	0.98227900	0.12536000
C	1.34537900	0.61305700	-0.44326100
C	1.21866400	-0.59904900	-1.37629000
H	-1.67308800	-0.01498200	1.11348200
H	-0.65094700	1.37958300	-0.67304300
H	2.05226200	0.42269800	0.36240000
H	0.65042600	-0.29335600	-2.26190600
O	0.56580900	-1.68847200	-0.73133700
C	-0.71512900	-1.39263000	-0.25207600
H	-1.07926900	-2.30408100	0.22148900
F	-4.37573300	-0.46537900	0.82663800
O	-4.91513600	-1.07992400	-1.48176700
O	-3.47028700	0.87232200	-0.96431700
H	-2.74812300	0.20555500	-1.14674600
C	-1.91492300	-2.08748600	-2.18708000
H	-2.43261100	-2.86992100	-1.63116700
H	-2.58285600	-1.67669100	-2.93796800
H	-1.00592500	-2.47664500	-2.64455200
O	-1.58226000	-1.00725500	-1.29853100
S	-4.84579700	0.11099900	-0.64324600
O	0.16253900	-0.63936600	1.84432400
C	-0.42120900	-1.39657600	2.80316300
O	-1.56605600	-1.76964100	2.73651600
C	0.54530400	-1.69961300	3.91084700
H	0.99958900	-0.77656400	4.27063900
H	0.01975100	-2.20983900	4.71323800
H	1.34202200	-2.33821000	3.52511200
O	0.07958500	1.95085700	1.17278900
C	0.02453100	3.25918400	0.83561800
O	-0.14881100	3.64700100	-0.29544700
C	0.24422500	4.12709900	2.03738000
H	-0.32221800	3.75319000	2.88918400
H	1.30713700	4.08188900	2.28461700
H	-0.03680900	5.14969400	1.80020300
O	1.83692300	1.65655400	-1.29737800
C	2.57919900	2.64483800	-0.74578800
O	2.89733600	2.66456300	0.41872600
C	2.90299000	3.69744600	-1.76276700
H	3.19391800	3.24388400	-2.70945600
H	1.99554300	4.28263300	-1.92725100
H	3.69160000	4.33927800	-1.37920600
C	2.56879800	-1.11696900	-1.82955100
H	3.11260300	-0.33507500	-2.35515200
H	2.43968500	-1.98967700	-2.46615400
O	3.34572200	-1.47108500	-0.68091600
C	3.51781700	-2.78948600	-0.42023700
O	3.16282000	-3.67339400	-1.15804400
C	4.22001800	-2.97326900	0.89534400
H	5.17489200	-2.44670300	0.87946200
H	3.61349800	-2.53481000	1.68935700
H	4.37397700	-4.03332600	1.07665700

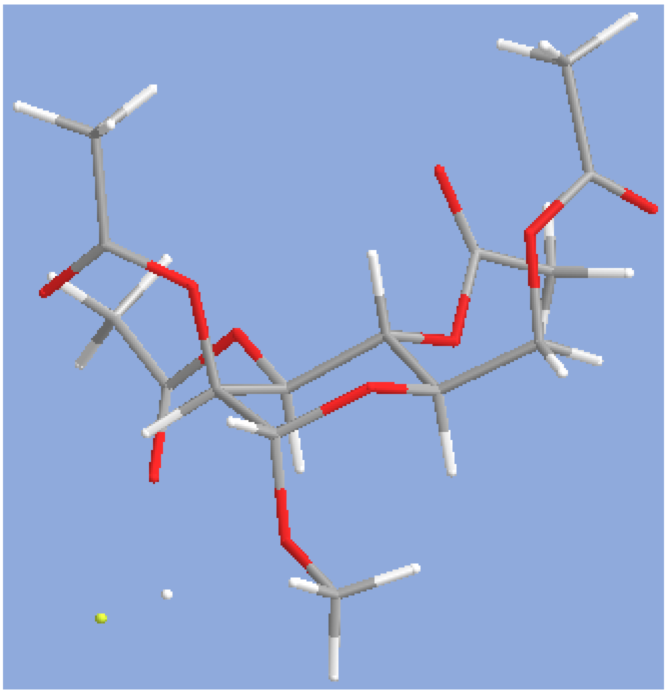

Zero-point correction= 0.398048 (Hartree/Particle)
Thermal correction to Energy= 0.427402
Thermal correction to Enthalpy= 0.428346
Thermal correction to Gibbs Free Energy= 0.334558
Sum of electronic and zero-point Energies= -1437.060407
Sum of electronic and thermal Energies= -1437.031053
Sum of electronic and thermal Enthalpies= -1437.030109
Sum of electronic and thermal Free Energies= -1437.123897

0 1

C	1.68927500	0.34788400	0.33862000
C	0.77341800	-0.32337300	-0.68380400
C	-0.66238700	0.07471100	-0.40737200
C	-0.77490300	1.59536700	-0.46666500
H	2.73618100	0.13581400	0.12201200
H	1.06930300	-0.02396200	-1.68997600
H	-0.97796700	-0.28853200	0.56786300
H	-0.46472900	1.94432200	-1.45870800
O	0.07955600	2.16512500	0.52884000
C	1.43364900	1.85228800	0.37139700
H	1.94284700	2.30992000	1.22165900
H	3.63014100	2.15458100	-0.83292300
O	1.35528300	-0.12127100	1.64983400
C	2.03973100	-1.19219300	2.12388700
O	2.94392700	-1.72096700	1.52713500
C	1.50091300	-1.61250200	3.45963600
H	1.38403100	-0.74626400	4.10994800
H	0.51476400	-2.05724400	3.31091200
H	2.17182500	-2.34287500	3.90345500
O	0.85849900	-1.74122900	-0.56016600
C	1.89922400	-2.32937600	-1.21148500
O	2.64743600	-1.71402600	-1.92835700
C	1.97940000	-3.79095500	-0.89376600
H	0.98377800	-4.22493100	-0.81458100
H	2.56851500	-4.29209000	-1.65753100
H	2.47991600	-3.88755400	0.07207800
O	-1.50849500	-0.46339800	-1.42570900
C	-2.37586900	-1.44609400	-1.06612600
O	-2.44858500	-1.88833400	0.05444000
C	-3.23705300	-1.85017300	-2.22322200
H	-2.66775300	-1.84840500	-3.15111400
H	-3.66516000	-2.82960800	-2.02620500
H	-4.04130500	-1.11498200	-2.30102600

C	-2.18337500	2.09438100	-0.22277000
H	-2.81024800	1.88553200	-1.08615100
H	-2.15445500	3.16555200	-0.02610700
O	-2.74356300	1.46334300	0.93508000
C	-3.87478800	0.73913700	0.76625100
O	-4.44682600	0.63575000	-0.29419400
C	-4.28551100	0.06779900	2.04151900
H	-4.03689500	0.68358800	2.90368300
H	-5.35007000	-0.14979000	2.00479900
H	-3.73226700	-0.87204300	2.10440100
O	1.94775600	2.36955200	-0.84468000
C	1.83318700	3.79713300	-0.93900900
H	2.33057700	4.26548200	-0.08675700
H	2.33057000	4.08155300	-1.86200400
H	0.78479800	4.09057000	-0.96559200
F	4.55158100	2.21564500	-0.60249600

TS-A‡

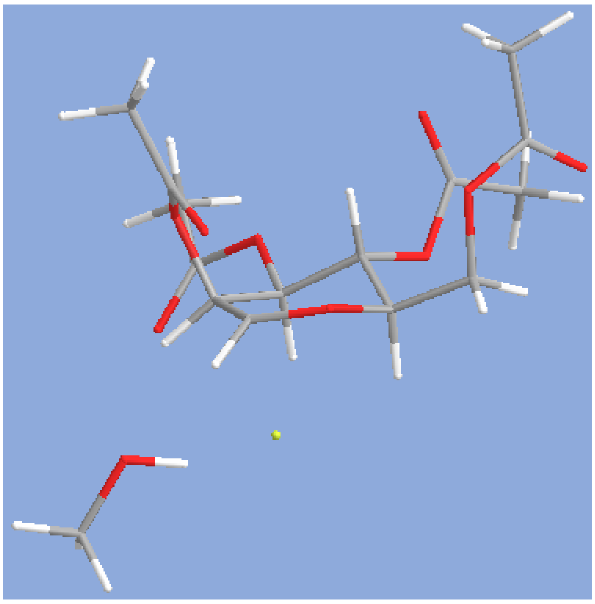

```
Zero-point correction=                           0.396755 (Hartree/Particle)
Thermal correction to Energy=                    0.426230
Thermal correction to Enthalpy=                  0.427175
Thermal correction to Gibbs Free Energy=         0.331709
Sum of electronic and zero-point Energies=          -1436.979104
Sum of electronic and thermal Energies=             -1436.949629
Sum of electronic and thermal Enthalpies=           -1436.948685
Sum of electronic and thermal Free Energies=        -1437.044151
```

0 1

C	-1.69228100	-0.09680700	0.22694800
C	-0.68791000	0.83392300	-0.45832300
C	0.67493400	0.17335500	-0.47071300
C	0.59489900	-1.16374100	-1.19298300
H	-2.72108600	0.14773600	-0.00537100
H	-1.05117500	1.02871900	-1.46347200
H	1.03911400	0.03418000	0.54834400
H	0.15101300	-1.03656100	-2.18049500
O	-0.27854300	-2.05070200	-0.46376200
C	-1.44748200	-1.59284700	-0.02557500
H	-2.30381200	-2.20958900	-0.23882600
F	-2.00146700	-0.88457000	-2.01745300
H	-3.50967100	-1.11413100	-1.58292600
C	-5.49566700	-1.05922500	-1.68433700
H	-6.31898300	-1.23771200	-0.99232600
H	-5.56736600	-0.03104000	-2.05376300

H	-5.59925000	-1.74537800	-2.53131100
O	-4.28819100	-1.27660200	-0.98495000
O	-1.54025800	0.12346600	1.68492100
C	-1.39713700	-0.99608300	2.32023500
O	-1.35433400	-2.05180100	1.66375800
C	-1.30435600	-0.94340500	3.80285000
H	-0.62390700	-0.14483200	4.09526100
H	-2.29469700	-0.71288000	4.20025600
H	-0.96907000	-1.90389600	4.18304000
O	-0.51528100	2.05788000	0.26562800
C	-1.55138900	2.92679500	0.24831300
O	-2.60693800	2.67747800	-0.27824100
C	-1.19642000	4.20083100	0.95978000
H	-0.38359100	4.69740400	0.42777700
H	-2.06974600	4.84611400	0.99480300
H	-0.84425200	3.97521500	1.96663700
O	1.59306500	0.99844900	-1.18675700
C	2.59265700	1.58743700	-0.48086100
O	2.73179300	1.45188100	0.71111700
C	3.49914400	2.36515900	-1.38424100
H	2.93313400	2.85489700	-2.17463800
H	4.05991300	3.08623200	-0.79510400
H	4.19293500	1.65314400	-1.83712500
C	1.93188600	-1.85398700	-1.30934600
H	2.55387700	-1.33741100	-2.03574700
H	1.77761000	-2.88822900	-1.61248300
O	2.59581800	-1.87257800	-0.03525600
C	3.82713300	-1.31874600	0.03856100
O	4.39673500	-0.82922000	-0.90927000
C	4.36012000	-1.35995500	1.43950400
H	4.03559700	-2.26313800	1.95295100
H	5.44480300	-1.29166700	1.41141600
H	3.96209600	-0.48828400	1.96381100

IP

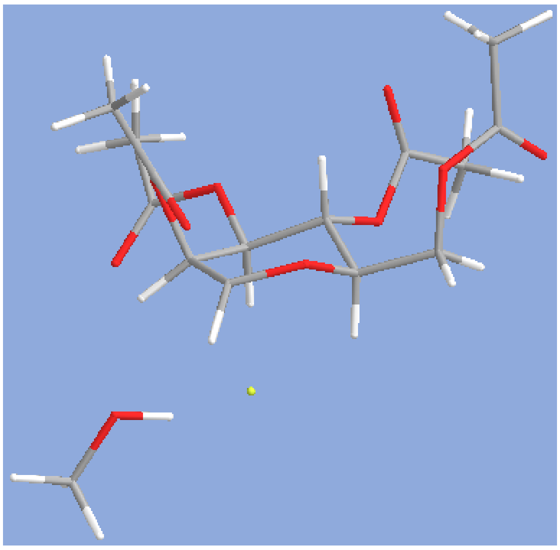

```
Zero-point correction=                           0.397567 (Hartree/Particle)
 Thermal correction to Energy=                    0.427359
 Thermal correction to Enthalpy=                  0.428303
 Thermal correction to Gibbs Free Energy=         0.332269
 Sum of electronic and zero-point Energies=          -1436.979616
 Sum of electronic and thermal Energies=             -1436.949824
 Sum of electronic and thermal Enthalpies=           -1436.948880
 Sum of electronic and thermal Free Energies=        -1437.044914
```

0 1

C	-1.70217200	-0.12888000	0.19409500
C	-0.64637200	0.83392700	-0.35137400

C	0.72010500	0.17946400	-0.38452700
C	0.63640300	-1.16737700	-1.09003000
H	-2.71333900	0.15584600	-0.07388700
H	-0.97070000	1.11739200	-1.34896300
H	1.10325400	0.04415500	0.62739300
H	0.15130900	-1.06329900	-2.06154400
O	-0.17668600	-2.02828500	-0.26411700
C	-1.46790600	-1.62968500	-0.03891000
H	-2.21311200	-2.11081400	-0.65206100
F	-1.82983400	-0.63321100	-2.22118800
H	-3.31007500	-0.83868800	-1.86270600
C	-5.28342800	-0.69765500	-2.11280600
H	-6.17071500	-0.89713200	-1.51053900
H	-5.28914200	0.35866200	-2.40158500
H	-5.33613600	-1.30878300	-3.02007500
O	-4.14813600	-1.01734900	-1.33785500
O	-1.65262700	-0.03509300	1.70104700
C	-1.74598500	-1.21078700	2.18531100
O	-1.74150500	-2.18150300	1.36668000
C	-1.88546300	-1.42727000	3.64227000
H	-1.52594900	-0.55468300	4.18109500
H	-2.94626300	-1.57464800	3.85894000
H	-1.34365000	-2.32580800	3.92959100
O	-0.49630900	1.98909100	0.48761000
C	-1.51989400	2.87163000	0.50712700
O	-2.56916300	2.67215200	-0.05292000
C	-1.16170400	4.09357600	1.30363700
H	-0.37240000	4.63968200	0.78443300
H	-2.04104000	4.72297500	1.40886700
H	-0.77459200	3.80193800	2.27995200
O	1.61125200	1.02364000	-1.11558200
C	2.62822800	1.61000400	-0.43598400
O	2.80257400	1.47115200	0.75144900
C	3.50778300	2.39441100	-1.36078200
H	2.92018800	2.87885600	-2.13855400
H	4.07690200	3.12005600	-0.78529300
H	4.19623700	1.68705000	-1.82884100
C	1.97904400	-1.83790600	-1.24111300
H	2.57202100	-1.31514100	-1.98723300
H	1.83306600	-2.87591000	-1.53526600
O	2.68247200	-1.84236200	0.01336500
C	3.90909700	-1.27742200	0.04756100
O	4.44811200	-0.78925600	-0.91908800
C	4.48303400	-1.30459300	1.43294600
H	4.19304200	-2.21411200	1.95601400
H	5.56474400	-1.21313900	1.37383500
H	4.08036500	-0.44168500	1.96790300

TS-B‡

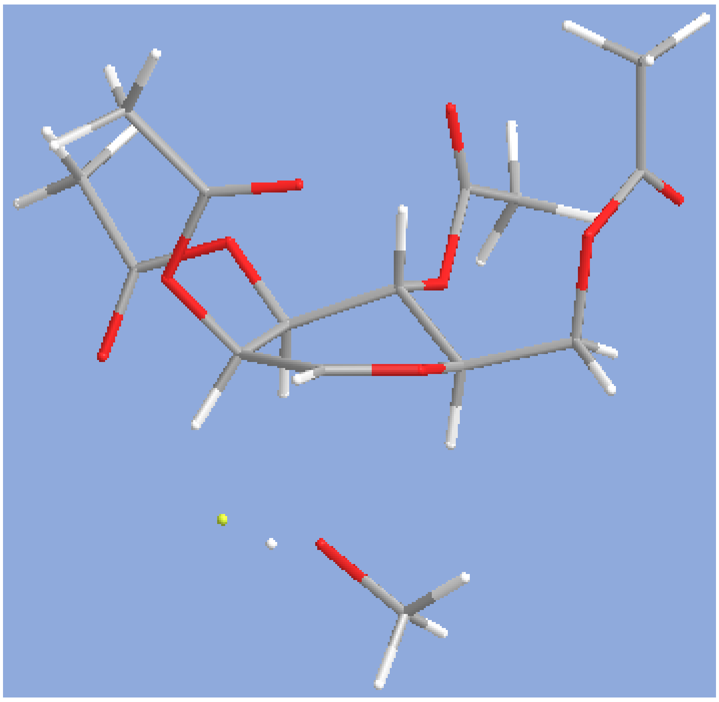

```
 Zero-point correction=                           0.393520 (Hartree/Particle)
 Thermal correction to Energy=                    0.422660
 Thermal correction to Enthalpy=                  0.423604
 Thermal correction to Gibbs Free Energy=         0.330698
 Sum of electronic and zero-point Energies=          -1436.971940
 Sum of electronic and thermal Energies=             -1436.942800
 Sum of electronic and thermal Enthalpies=           -1436.941856
 Sum of electronic and thermal Free Energies=        -1437.034763

0 1
 C                  1.92470000    0.32528700    0.07191700
 C                  0.86517500   -0.43617700   -0.76276500
 C                 -0.55124900   -0.04527400   -0.38976800
 C                 -0.67386200    1.47052200   -0.42546300
 H                  2.74442000    0.65083100   -0.55940900
 H                  1.04473100   -0.20025000   -1.81150500
 H                 -0.82424400   -0.43311800    0.58914400
 H                 -0.29849600    1.86773600   -1.36908200
 O                  0.20740400    2.02657800    0.60049600
 C                  1.37453200    1.55457800    0.75033300
 H                  1.99445300    2.06864300    1.46940800
 H                  2.71055900    2.69028400   -0.77898300
 O                  2.55057400   -0.51662500    1.04905900
 C                  1.84231500   -0.74150700    2.16844600
 O                  0.82365200   -0.12557400    2.40087300
 C                  2.45280800   -1.79655400    3.03407300
 H                  2.18401700   -2.77214000    2.62281400
 H                  3.53786100   -1.70760600    3.02877100
 H                  2.05498900   -1.71103000    4.04149000
 O                  0.96118800   -1.84258800   -0.55721300
 C                  2.01352100   -2.45051800   -1.16823600
 O                  2.80876200   -1.84527800   -1.83861100
 C                  2.03893900   -3.92057200   -0.86931800
 H                  1.03191700   -4.33440700   -0.88896100
 H                  2.68285100   -4.41874600   -1.58917900
 H                  2.44718200   -4.06503200    0.13341900
 O                 -1.44244500   -0.54712900   -1.38409300
 C                 -2.30438800   -1.53750000   -1.01199300
 O                 -2.32708700   -2.00475400    0.09861900
 C                 -3.21144500   -1.90591400   -2.14427700
 H                 -2.67426400   -1.89745100   -3.09114400
```

H	-3.64943900	-2.88112300	-1.94854100
H	-4.00438000	-1.15562800	-2.18229400
C	-2.06146100	1.99007400	-0.14355100
H	-2.70573900	1.78610600	-0.99581900
H	-2.01675800	3.06210300	0.04473700
O	-2.58832800	1.36284100	1.02785100
C	-3.72338500	0.63119700	0.89167300
O	-4.31654200	0.52432900	-0.15560100
C	-4.09424600	-0.03413300	2.18062600
H	-3.87409600	0.61267200	3.02783200
H	-5.14607300	-0.30666800	2.15322000
H	-3.49044400	-0.94062800	2.26498400
O	1.88226300	2.48334400	-1.49248800
C	1.31893500	3.71245800	-1.87719200
H	0.66837600	4.12703400	-1.08952000
H	2.09358100	4.45597900	-2.08658700
H	0.71961400	3.57766800	-2.78365200
F	3.44761300	2.76753500	0.22007800

References

1. Frisch, M. J.; Trucks, G. W.; Schlegel, H. B.; Scuseria, G. E.; Robb, M. A.; Cheeseman, J. R.; Scalmani, G.; Barone, V.; Mennucci, B.; Petersson, G. A.; Nakatsuji, H.; Caricato, M.; Li, X.; Hratchian, H. P.; Izmaylov, A. F.; Bloino, J.; Zheng, G.; Sonnenberg, J. L.; Hada, M.; Ehara, M.; Toyota, K.; Fukuda, R.; Hasegawa, J.; Ishida, M.; Nakajima, T.; Honda, Y.; Kitao, O.; Nakai, H.; Vreven, T.; Jr., J. A. M.; Peralta, J. E.; Ogliaro, F.; Bearpark, M.; Heyd, J. J.; Brothers, E.; Kudin, K. N.; Staroverov, V. N.; Keith, T.; Kobayashi, R.; Normand, J.; Raghavachari, K.; Rendell, A.; Burant, J. C.; Iyengar, S. S.; Tomasi, J.; Cossi, M.; Rega, N.; Millam, J. M.; Klene, M.; Knox, J. E.; Cross, J. B.; Bakken, V.; Adamo, C.; Jaramillo, J.; Gomperts, R.; Stratmann, R. E.; Yazyev, O.; Austin, A. J.; Cammi, R.; Pomelli, C.; Ochterski, J. W.; Martin, R. L.; Morokuma, K.; Zakrzewski, V. G.; Voth, G. A.; Salvador, P.; Dannenberg, J. J.; Dapprich, S.; Daniels, A. D.; Farkas, O.; Foresman, J. B.; Ortiz, J. V.; Cioslowski, J.; Fox, D. J. *Gaussian 09, Revision D.01*; Gaussian, Inc., Wallingford CT, 2013.
2. Kóňa, J.; Tvaroška, I. Comparative DFT Study on the α-Glycosidic Bond in Reactive Species of Galactosyl Diphosphates. *Chemical Papers* **2009**, *63* (5). https://doi.org/10.2478/s11696-009-0060-4.
3. Bootsma, A. N.; Wheeler, S. *Popular Integration Grids Can Result in Large Errors in DFT-Computed Free Energies*; preprint; 2019. https://doi.org/10.26434/chemrxiv.8864204.v4.
